# Supplementary material for: Cyclin-dependent kinase inhibitor p18 regulates lineage transitions of excitatory neurons, astrocytes, and interneurons in the mouse cortex
Source: EMBO J. 2024 Dec 12;44(2):382–412. doi: 10.1038/s44318-024-00325-9 (PMC11730326; doi:10.1038/s44318-024-00325-9)
Supplement: Supplementary file 1 — Appendix [file 44318_2024_325_MOESM1_ESM.pdf]

APPENDIX

Cyclin-dependent kinase inhibitor p18 regulates lineage transitions of excitatory neurons, astrocytes, and interneurons in the mouse cortex

Wonyoung Lee, Byunghee Kang, Hyo-Min Kim, Tsuyoshi Ishida, Minkyung Shin, Misato Iwashita, Masahiro Nitta, Aki Shiraishi, Hiroshi Kiyonari, Koichiro Shimoya, Kazuto Masamoto, Tae-Young Roh, and Yoichi Kosodo

TABLE OF CONTENTS

Appendix Figure S1. Characterizations of the knockdown (KD) of p18 and p27 Page 2

Appendix Figure S2. Effects of p18 OE introduced at E12.5 on cellular differentiations Page 5

Appendix Figure S3. Generation and characterization of p18-P2A-mKO2 Tg mice Page 7

Appendix Figure S4. Validation of the results of RNAseq by p18 OE using IUE and confirmation of Dlx2 KD Page 9

Appendix Figure S5. The expression level of marker genes in p18 and p27 double KD tissue Page 11

Appendix Fig. S1

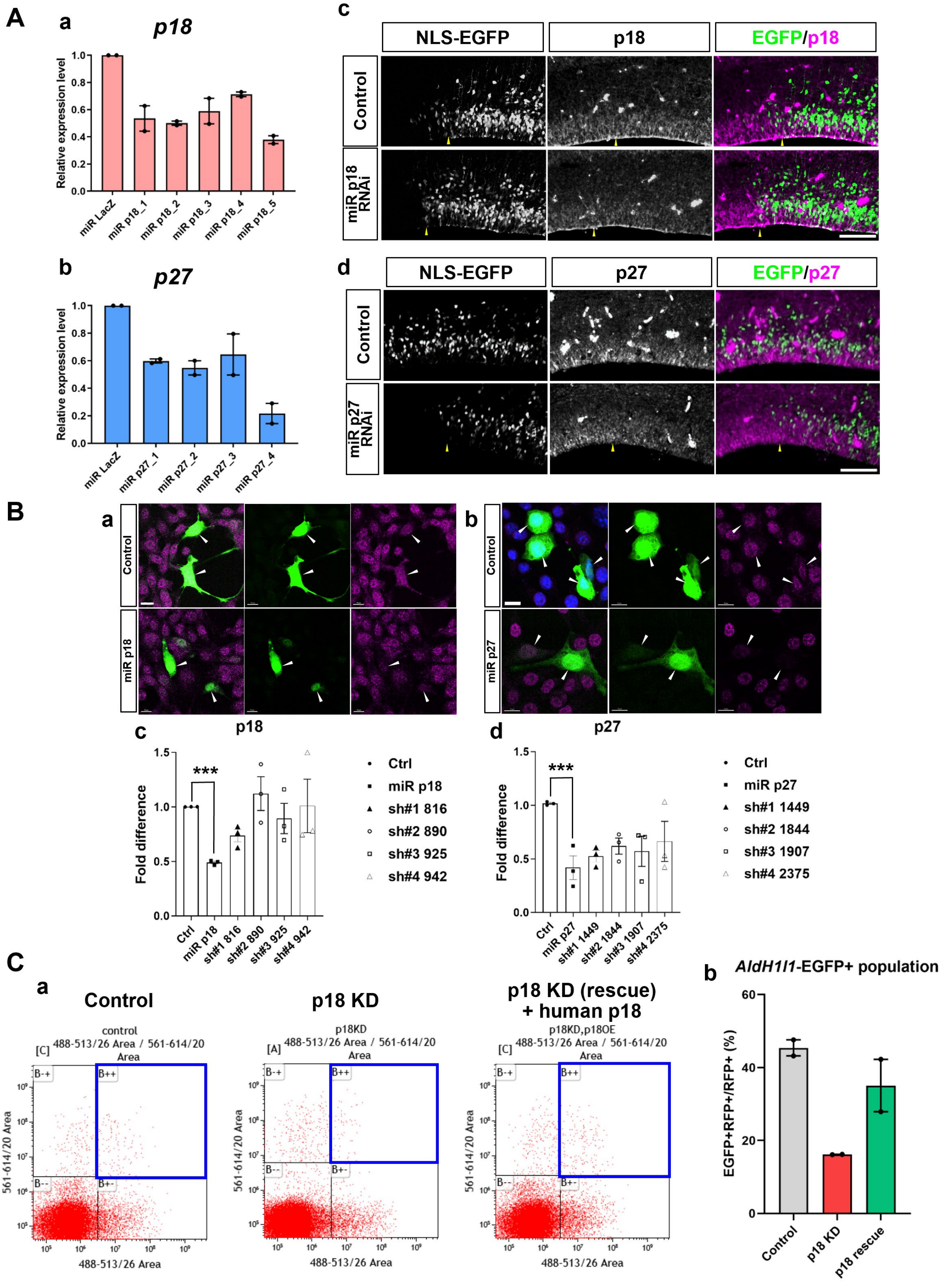

Appendix Figure S1. Characterizations of the knockdown (KD) of p18 and p27

(A) Confirmation of the efficiency of p18 and p27 KD *in vitro* and *in vivo*. **(a**, **b)** To select KD constructs for p18 and p27, plasmids containing miRNAs for control (LacZ), p18 (a, #1-5), or p27 (b, #1-4) mixed with pCAG-H2B-TagRFP were transfected into NS cells by electroporation. After 1 d, the cells were detached from the dish, and H2B-TagRFP-positive cells were collected by a fluorescence-activated cell sorting (FACS) system. RNA was extracted, and qPCR was performed to compare the KD efficiency among constructs. **(c**, **d)** Plasmids to induce KD of mouse p18 (#5 in a) and p27 (#4 in b) mixed with pCAG-NLS-EGFP were delivered at E15.5 of the *AldH1l1*-EGFP mice and then fixed at E16.5. Arrowheads; the border between electroporated and non-electroporated regions. Bar, 100 µm.

(B) Comparison of p18 and p27 KD efficiency between miRNA and shRNA. Knock-down constructs of p18 (**a, c**) and p27 (**b, d**) selected in **A**, as well as the constructs using the other vector and sequences, were electroporated into MEFs. Subsequently, immunostaining using p18 (a) and p27 (b) antibodies and qPCR (c, d) was performed to confirm the KD efficiencies. Arrowheads; EGFP positive cells. Bar, 10 µm.

(C) Rescue experiment of p18 KD. **(a)** To confirm that the KD of p18 caused the decreased expression of *AldH1l1*-EGFP, plasmids to induce overexpression (OE) of human p18 and KD of mouse p18 with pCAG-H2B-TagRFP were delivered at E15.5 of the *AldH1l1*-EGFP mice. After dissection at E16.5, cells in the electroporated region were dissociated and analyzed by FACS. **(b)** Percentage of *Aldh1l1*-EGFP-positive populations among H2B-TagRFP-positive cells (blue square in a).

(A, C) Error bars show mean ± SEM. **(B)** One-way ANOVA with tukey's test, $*p < 0.05$, $**p < 0.01$, $***p < 0.001$. Error bars show mean ± SEM.

Appendix Fig. S2

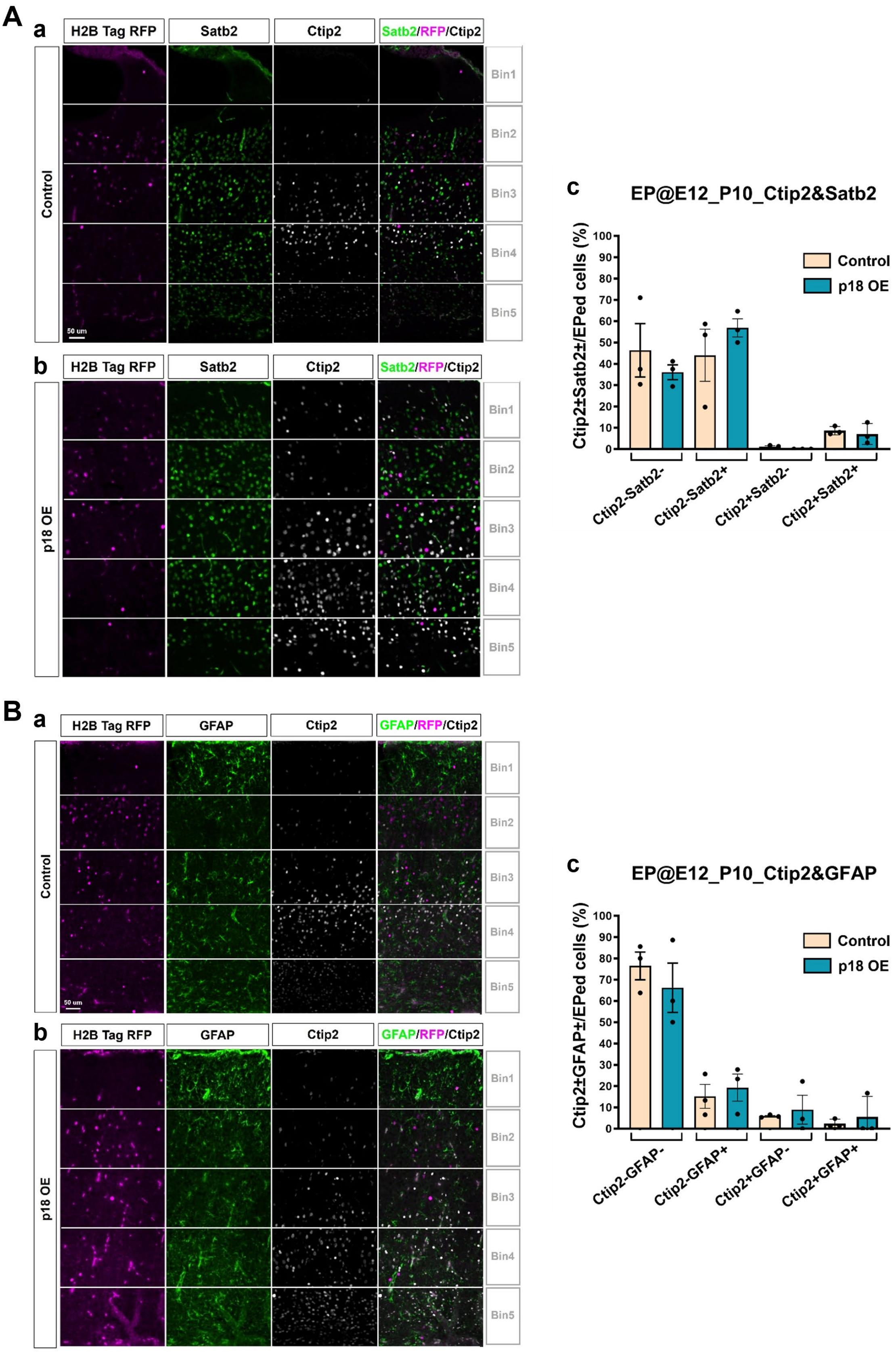

Appendix Figure S2. Effects of p18 OE introduced at E12.5 on cellular differentiations

(**A**) (**a, b**) Plasmids to induce p18 OE with pCAG-H2B-TagRFP were delivered at E12.5 and then fixed at P10. Sections were stained using Satb2 and Ctip2 antibodies. Bar, 50 µm. (**c**) Quantification of Satb2- and Ctip2-positive cells among H2B-TagRFP-positive cells (n = 3 brains). No significant changes were observed between Control and p18 OE.

(**B**) (**a, b**) Plasmids to induce p18 OE with pCAG-H2B-TagRFP were delivered at E12.5 and then fixed at P10. Sections were stained using Ctip2 and GFAP antibodies. Bar, 50 µm. (**c**) Quantification of Ctip2- and GFAP-positive cells among H2B-TagRFP-positive cells (n = 3 brains). No significant changes were observed between Control and p18 OE.

(A, B) Two-tailed *t*-test, no statistically significant changes were detected. Error bars show mean ± SEM.

Appendix Fig. S3

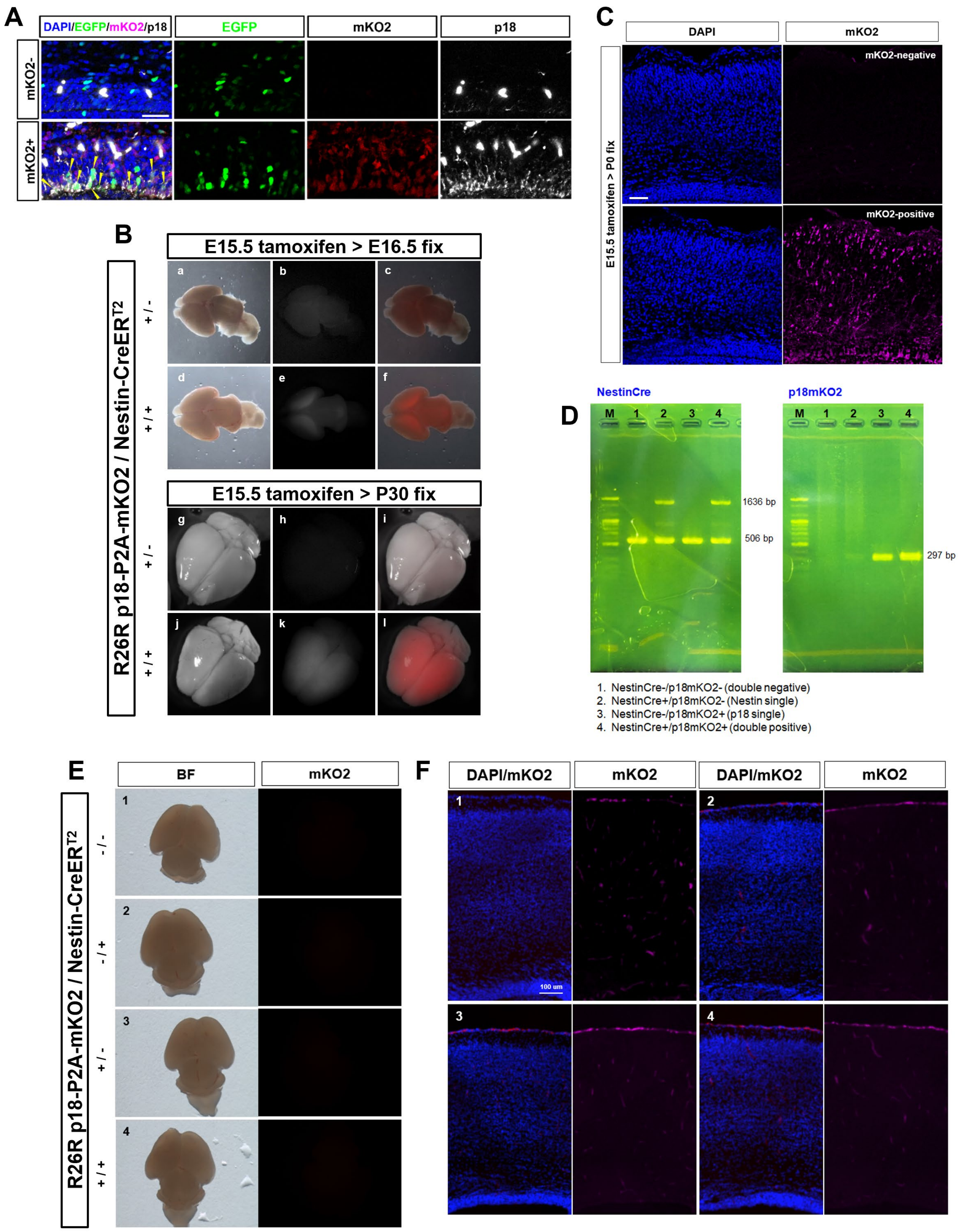

Appendix Figure S3. Generation and characterization of p18-P2A-mKO2 Tg mice

(**A**) pCAG-Cre mixed with pCAG-NLS-EGFP was delivered at E16.5 of the R26R-p18-P2A-mKO2 mice (+/- and -/-) and then fixed at P0. Sections were stained using EGFP and p18 antibodies to confirm mKO2-P2A-p18 expression as a result of recombination. Bar, 50 μm.

(**B**) Confirmation of mKO2-P2A-p18 expression after tamoxifen administration to the R26R-p18-P2A-mKO2/Nes-CreER^{T2} mice at E15.5. Brains of fixed mice were dissected and observed at E16.5 (**a-f**) and P30 (**g-l**).

(**C**) Confirmation of mKO2-P2A-p18 expression after tamoxifen administration to the R26R-p18-P2A-mKO2/Nes-CreER^{T2} mice at E15.5. Brain sections of mice fixed at P0 were observed. Bar, 100 μm.

(**D**) Confirmation of genotypes derived from the R26R-p18-P2A-mKO2/Nes-CreER^{T2} mice used in **E** and **F**.

(**E**) Whole brain images confirming the absence of mKO2 expressions without tamoxifen administration in all genotypes.

(**F**) Brain tissue images confirming the absence of mKO2 expressions without tamoxifen administration in all genotypes.

Appendix Fig. S4

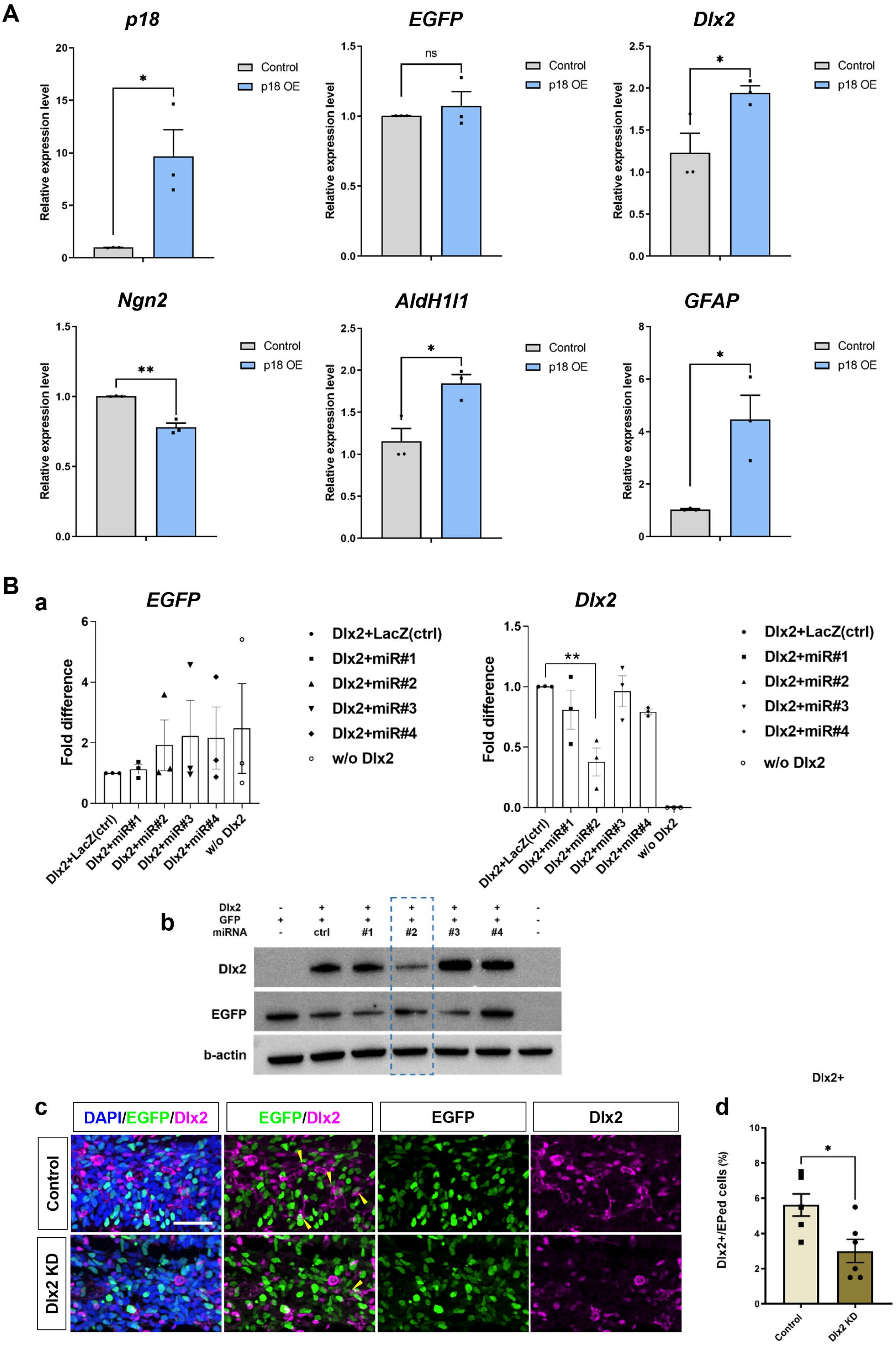

Appendix Figure S4. Validation of the results of RNAseq by p18 OE using IUE and confirmation of Dlx2 KD

(A) qPCR of tissue fragments to confirm genes upregulated by p18 OE. Plasmids to induce p18 OE mixed with pCAG-NLS-EGFP were delivered at E15.5, and then brains were dissected at E16.5. RNA was prepared from VZ isolated by LMD, followed by qPCR analysis to evaluate the expression of landmark genes.

(B) Confirmation of the efficiency of Dlx2 KD. **(a)** To select KD constructs for Dlx2, plasmids containing miRNAs for control (LacZ) or Dlx2 (#1-4) mixed with pCAG-NLS-EGFP and pCAG-Dlx2 were transfected into HEK293T cells. Subsequently, qPCR analysis using RNAs from total cell lysates was performed (n = 3 culture). **(b)** Plasmids containing miRNAs for control (LacZ) or Dlx2 (#1-4) mixed with pCAG-NLS-EGFP and pCAG-Dlx2 were transfected into HEK293T cells. Protein levels were examined by western blotting. **(c)** Plasmids containing miRNAs for control (LacZ) or Dlx2#2 mixed with pCAG-NLS-EGFP were delivered at E15.5 and then fixed at E17.5. Sections were stained using Dlx2 antibody. Arrowheads; double-positive cells for Dlx2 and EGFP. Bar, 100 µm. **(d)** Quantification of the images shown in **c** (n = 6 sections from 3 brains each).

(A, B(d)) Two-tailed *t*-test, **(B(a))** one-way ANOVA with tukey's test, *$p < 0.05$, **$p < 0.01$, ***$p < 0.001$. Error bars show mean ± SEM.

Appendix Fig. S5

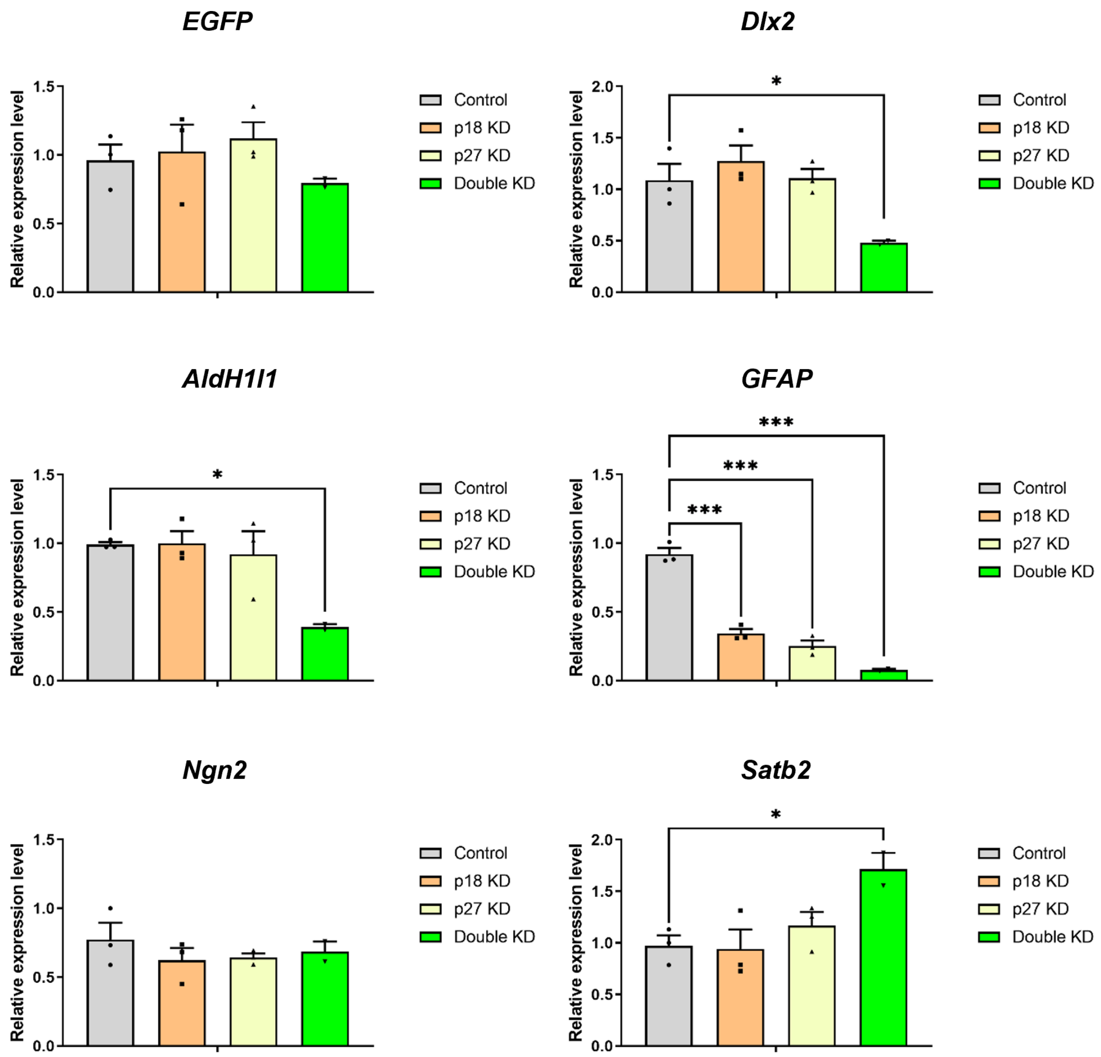

Appendix Figure S5. The expression level of marker genes in p18 and p27 double KD tissue

Plasmids to induce KD of p18 and p27 mixed with pCAG-NLS-EGFP were delivered at E15.5, followed by dissection at E17.5. Subsequently, qPCR analysis was performed using RNAs prepared from the VZ isolated by LMD. One-way ANOVA with tukey's test, $*p < 0.05$, $**p < 0.01$, $***p < 0.001$. Error bars show mean ± SEM.
