# Supplementary material for: Cyclin-dependent kinase inhibitor p18 regulates lineage transitions of excitatory neurons, astrocytes, and interneurons in the mouse cortex
Source: EMBO J. 2024 Dec 12;44(2):382–412. doi: 10.1038/s44318-024-00325-9 (PMC11730326; doi:10.1038/s44318-024-00325-9)
Supplement: Supplementary file 3 — Source data Fig. 1 [file 44318_2024_325_MOESM3_ESM.zip › 1B.pptx]

## Slide 1
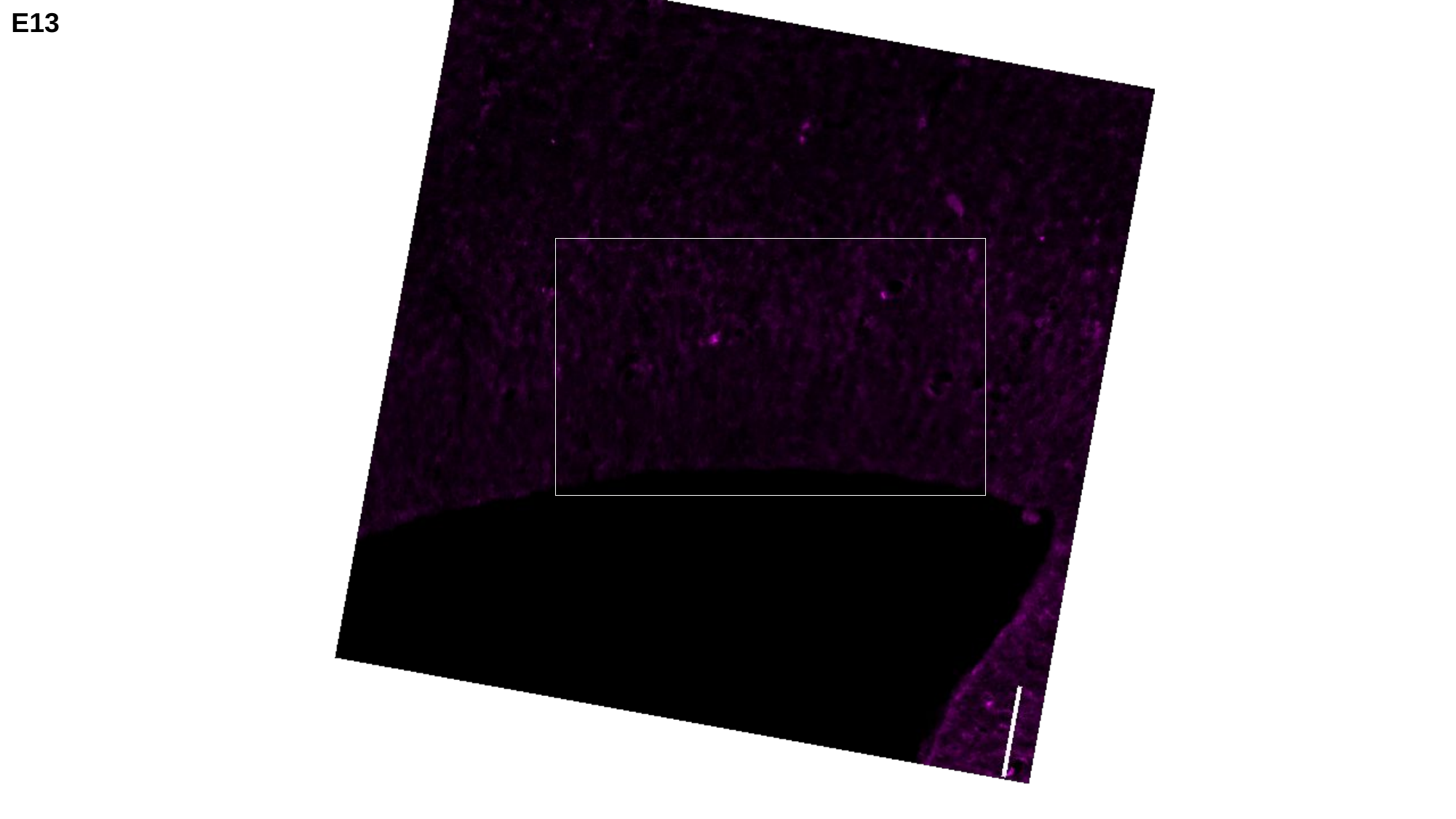

E13

## Slide 2
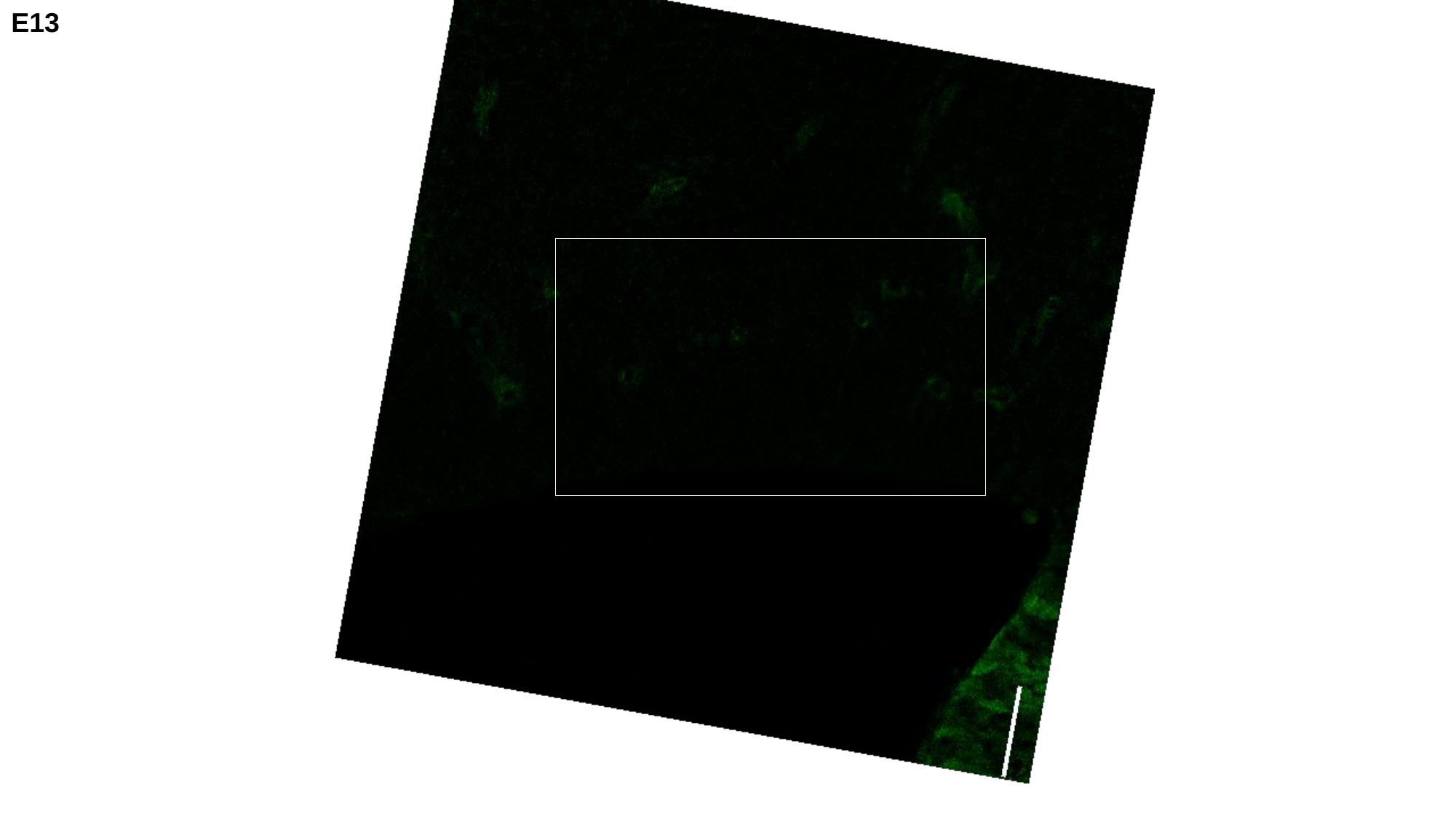

E13

## Slide 3
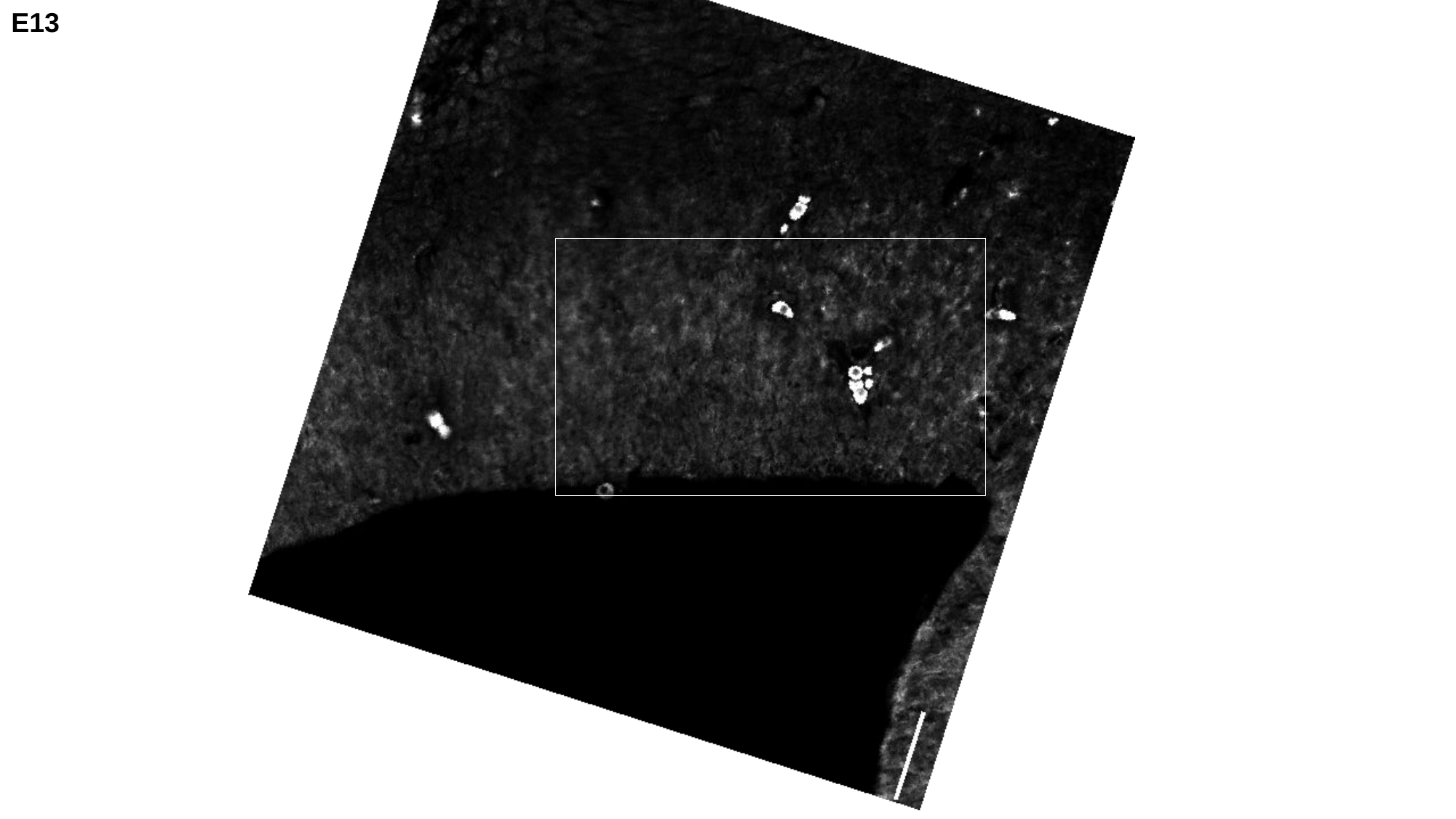

E13

## Slide 4
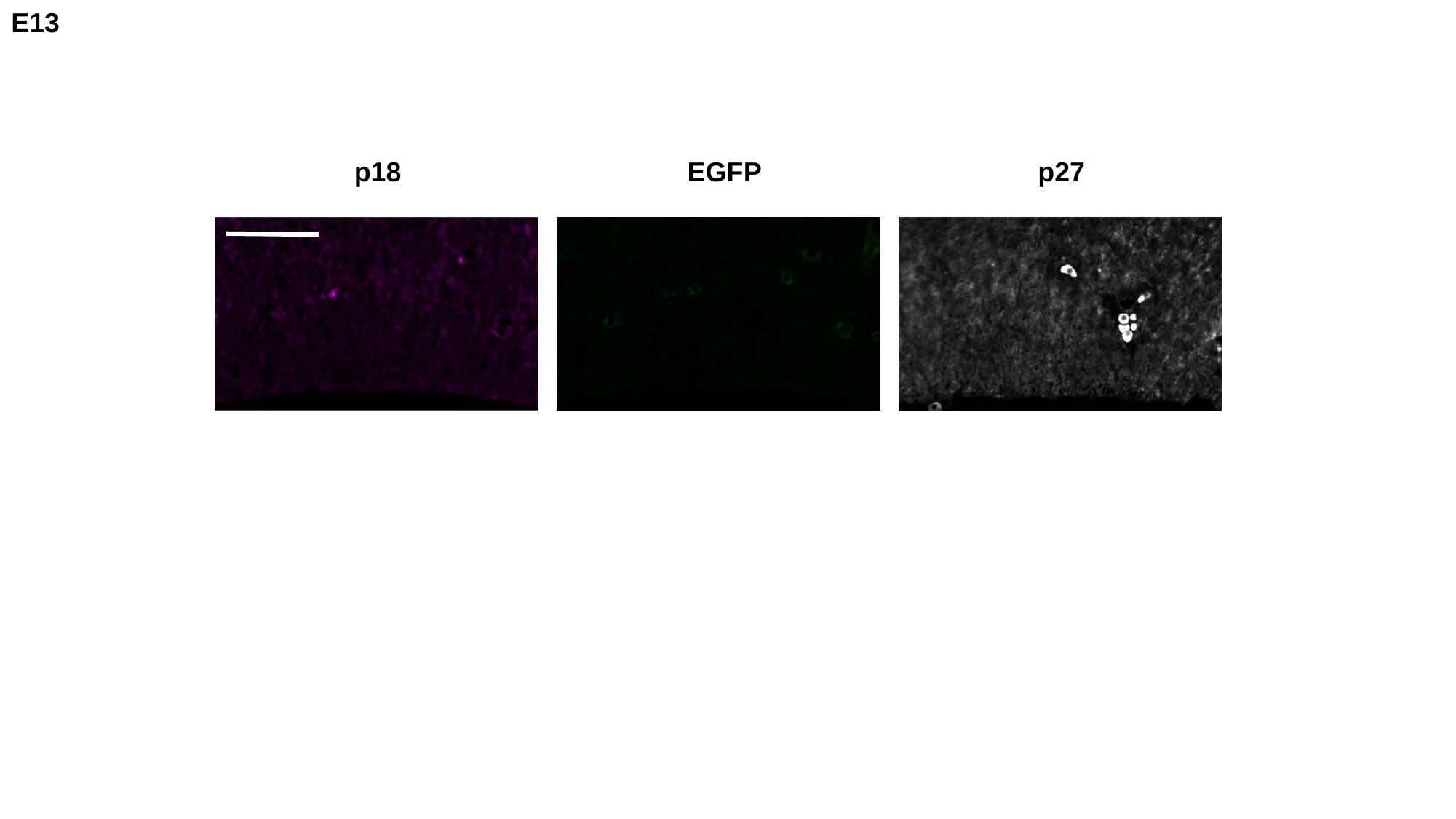

E13
p18
EGFP
p27

## Slide 5
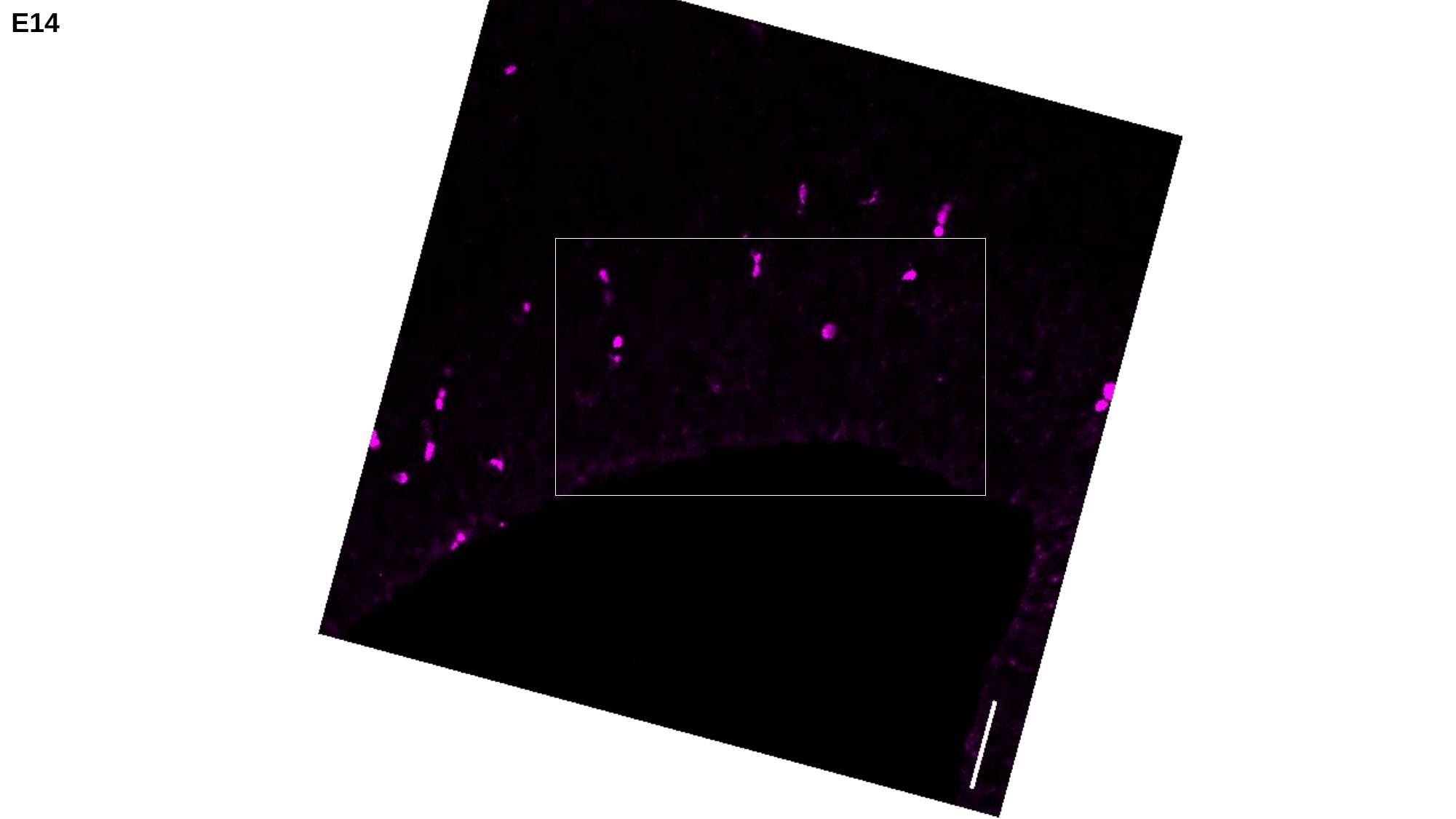

E14

## Slide 6
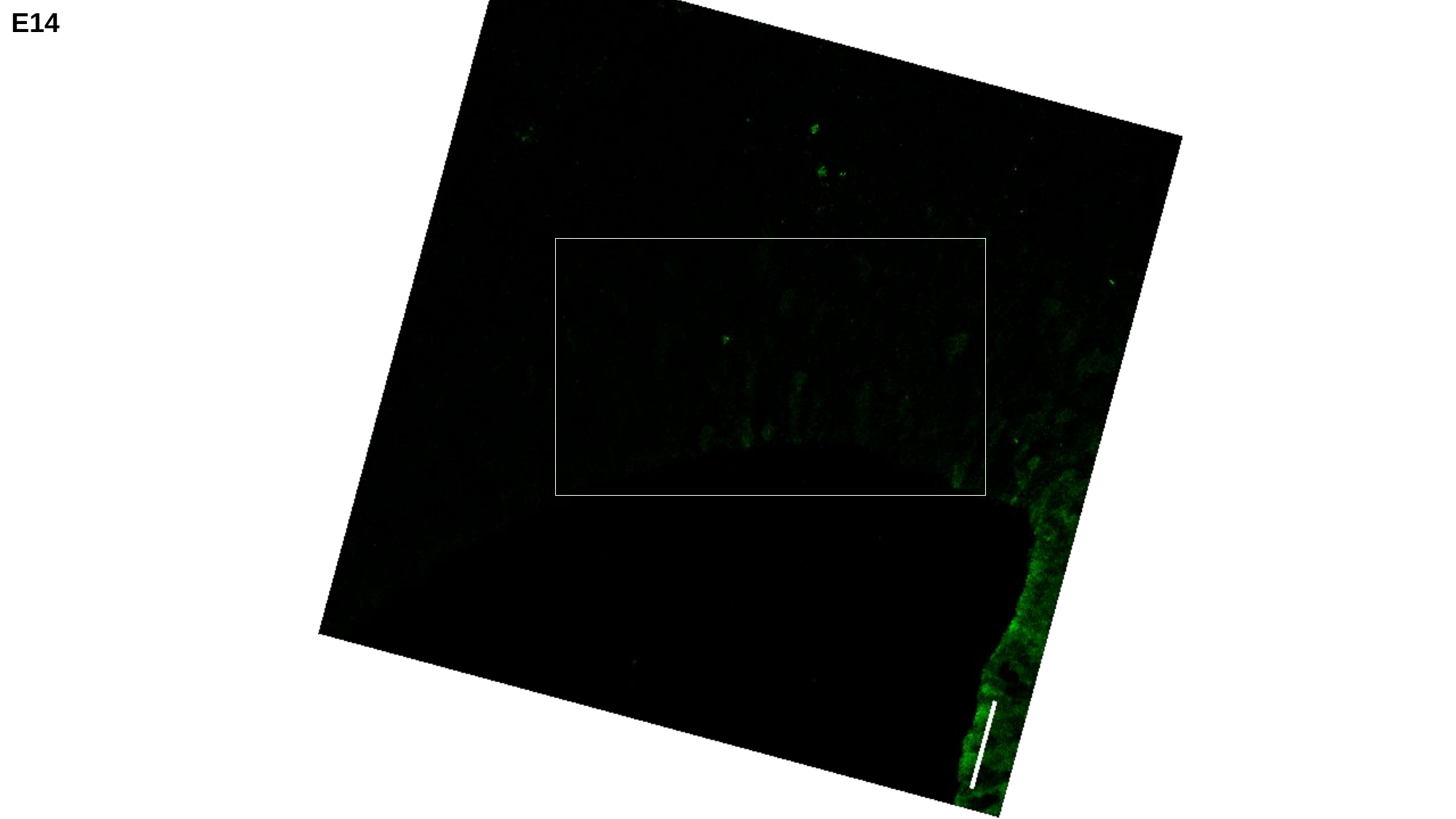

E14

## Slide 7
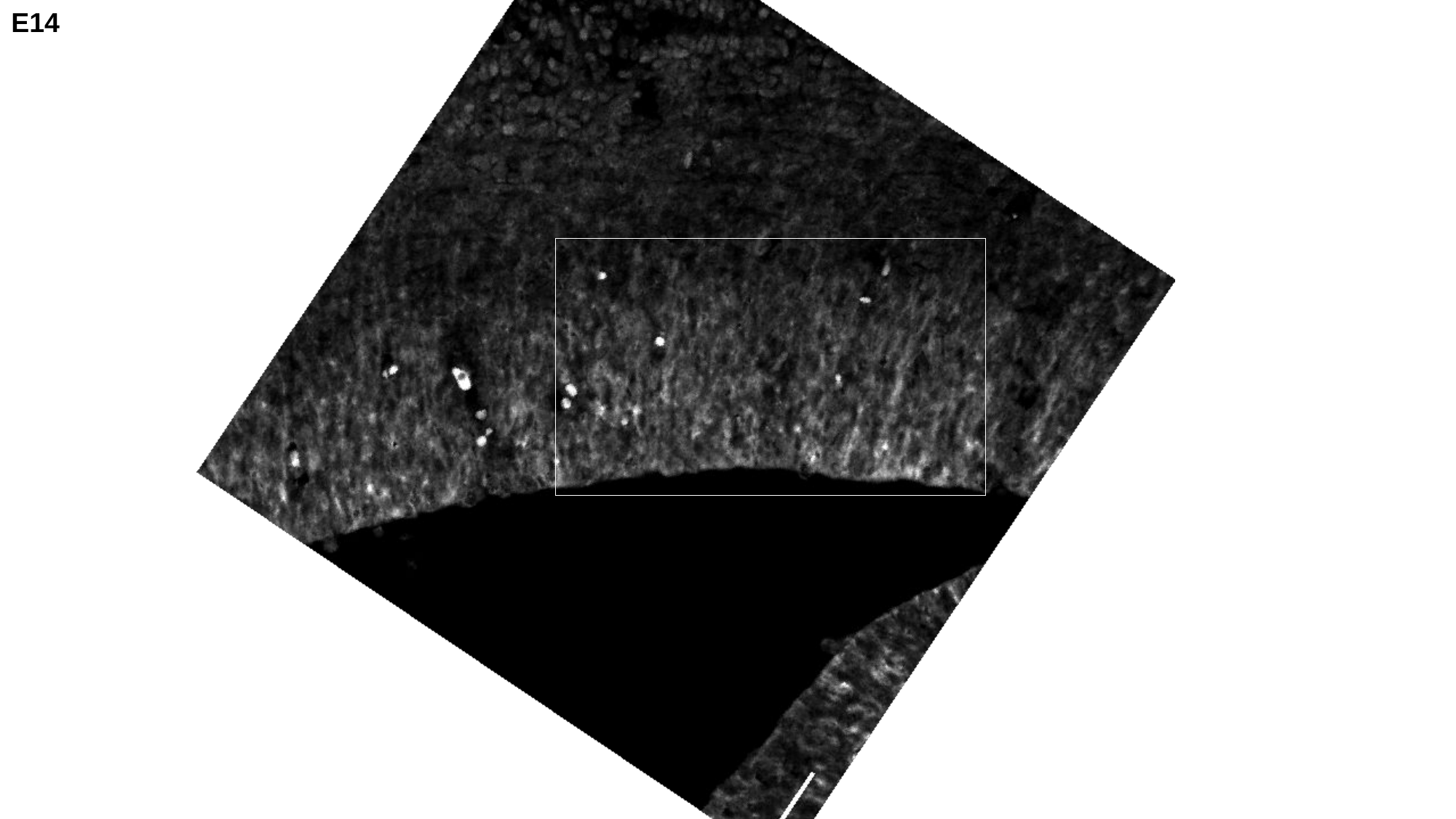

E14

## Slide 8
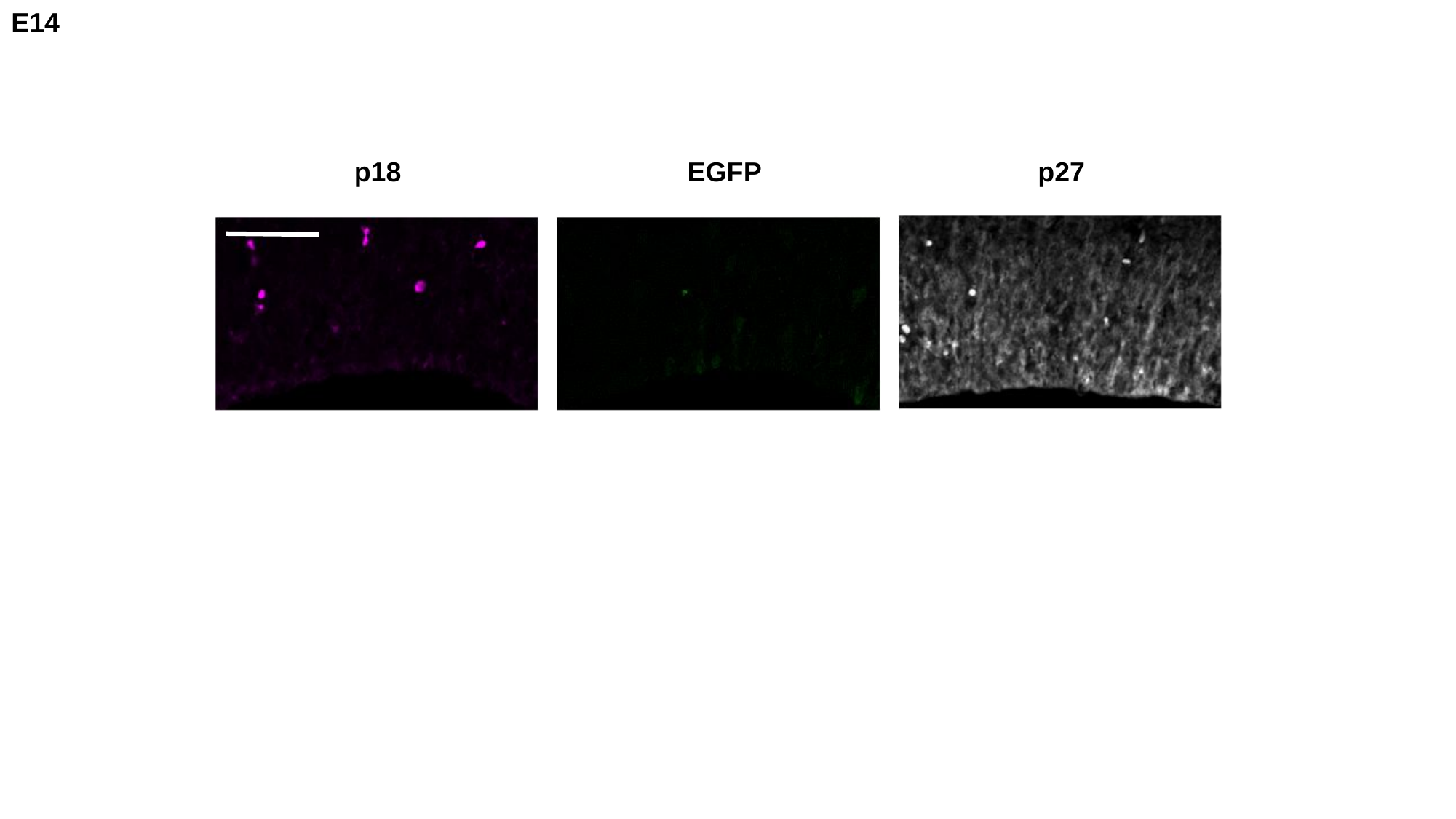

E14
p18
EGFP
p27

## Slide 9
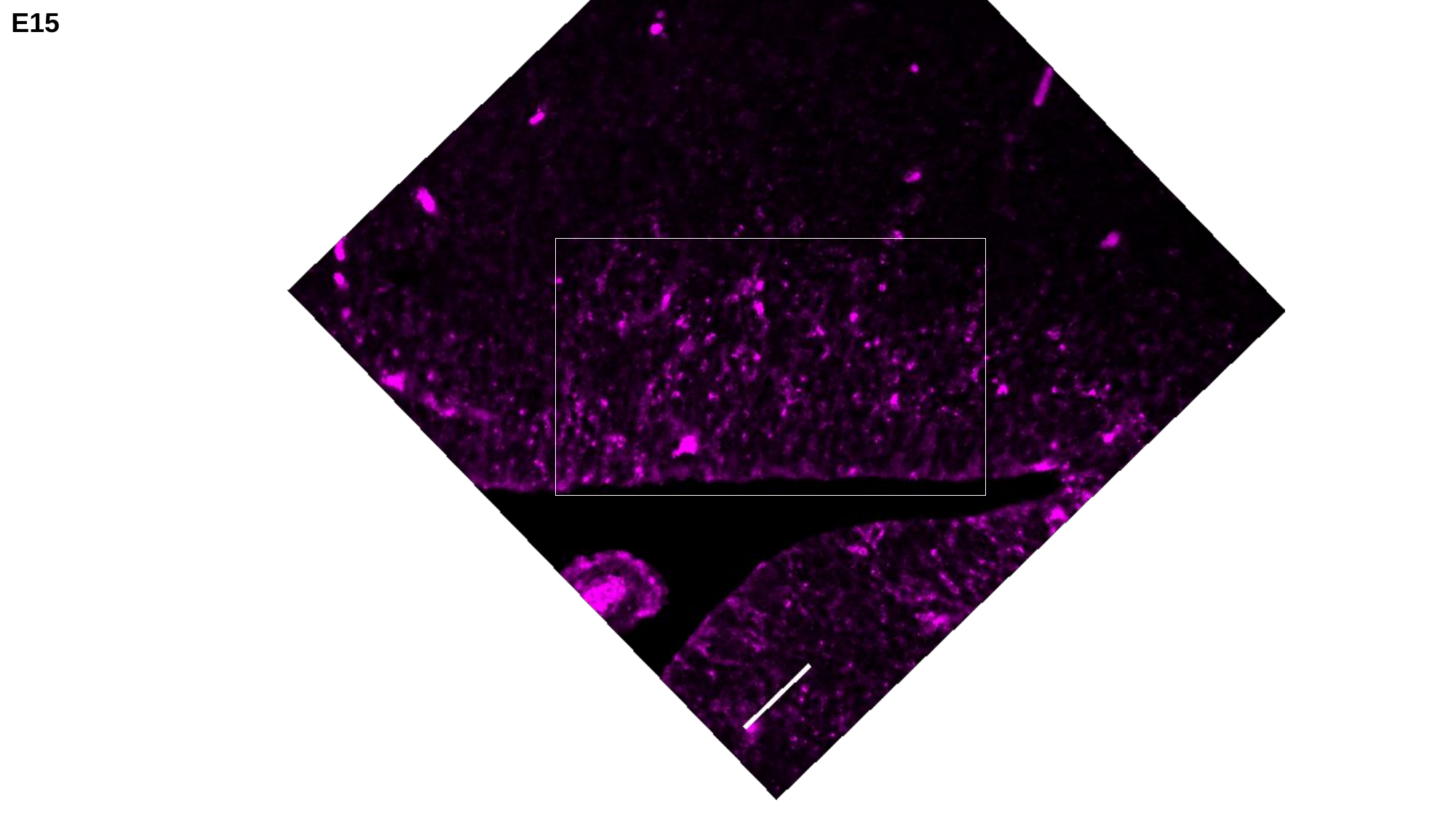

E15

## Slide 10
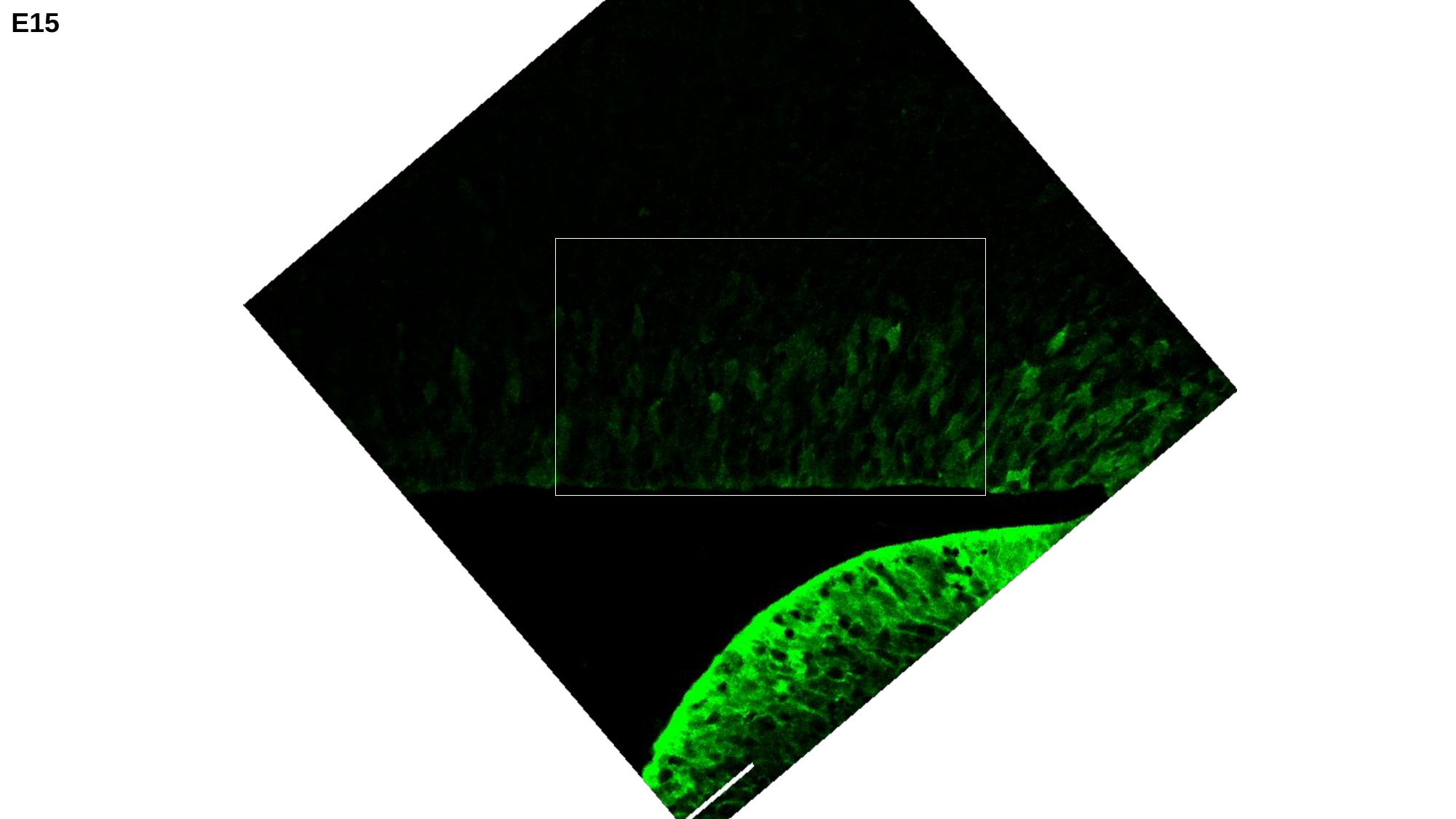

E15

## Slide 11
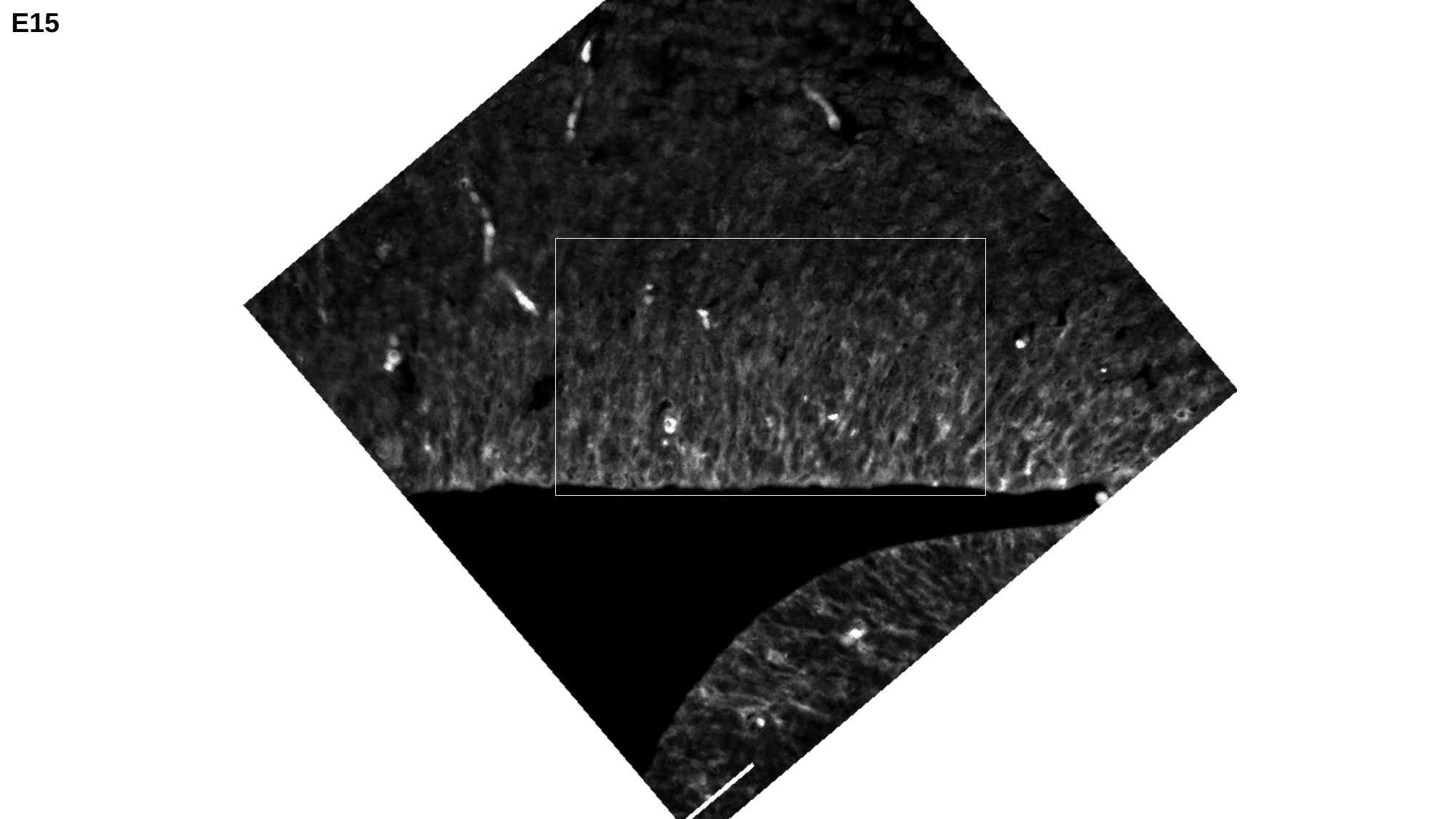

E15

## Slide 12
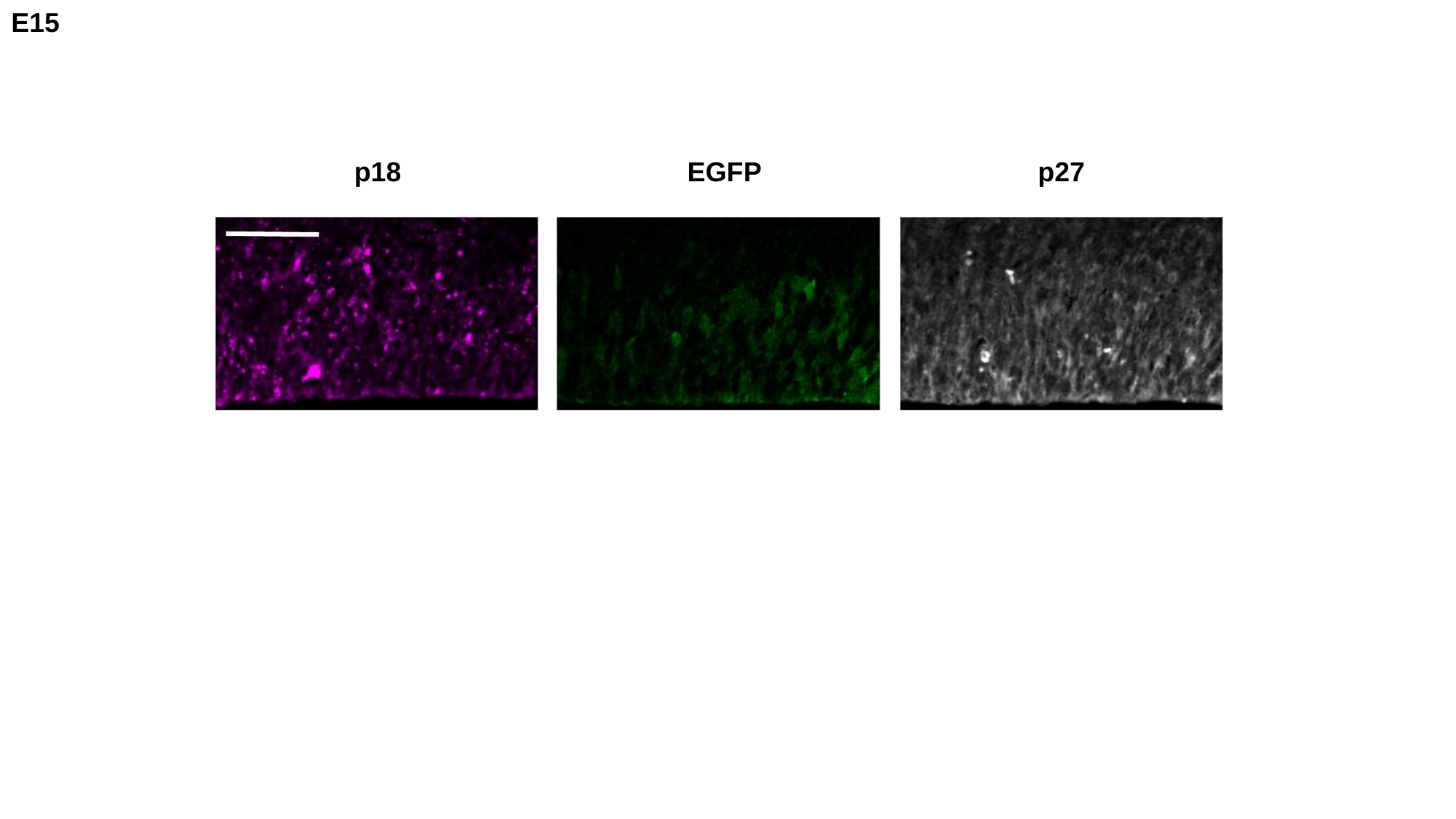

E15
p18
EGFP
p27

## Slide 13
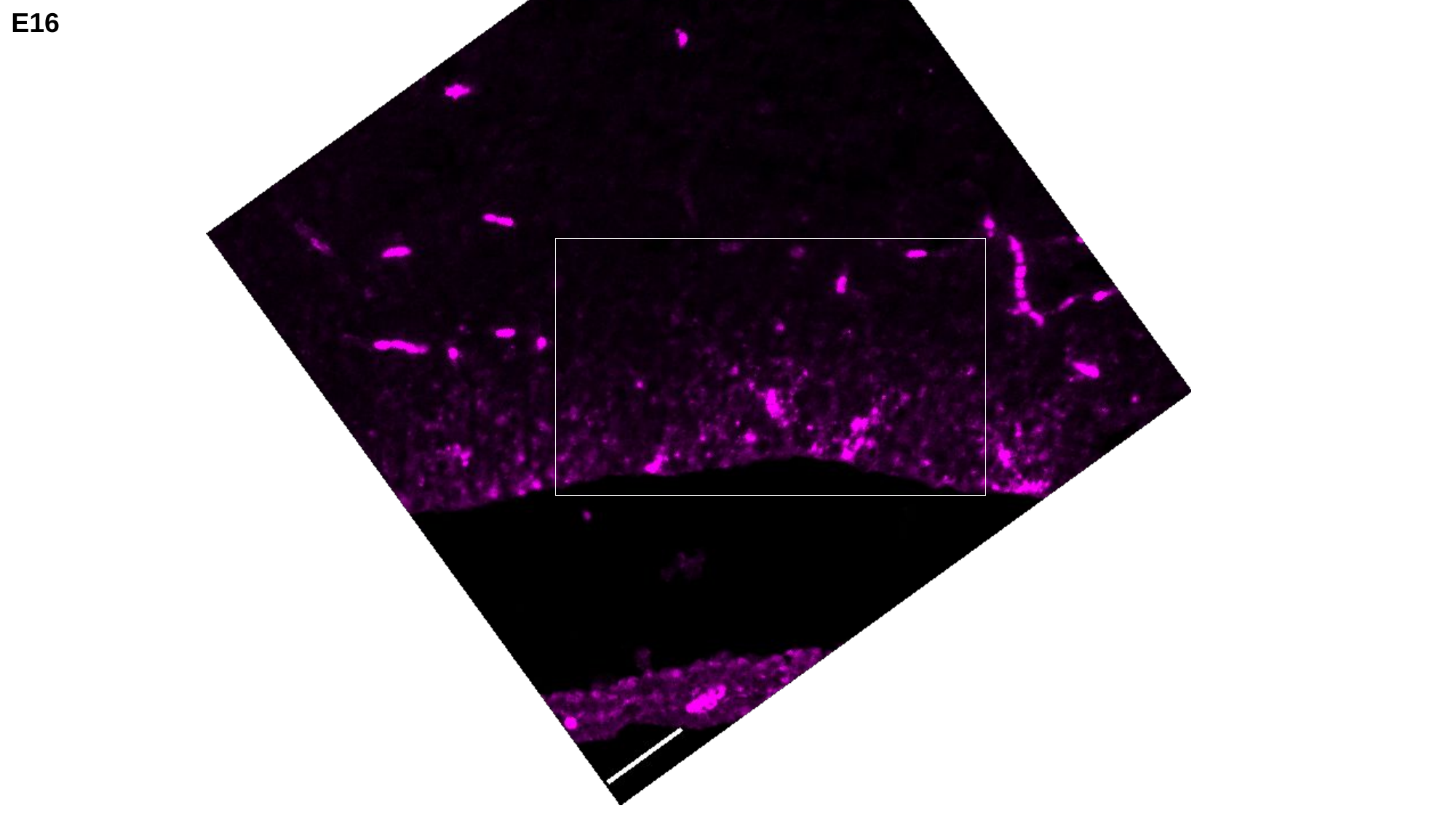

E16

## Slide 14
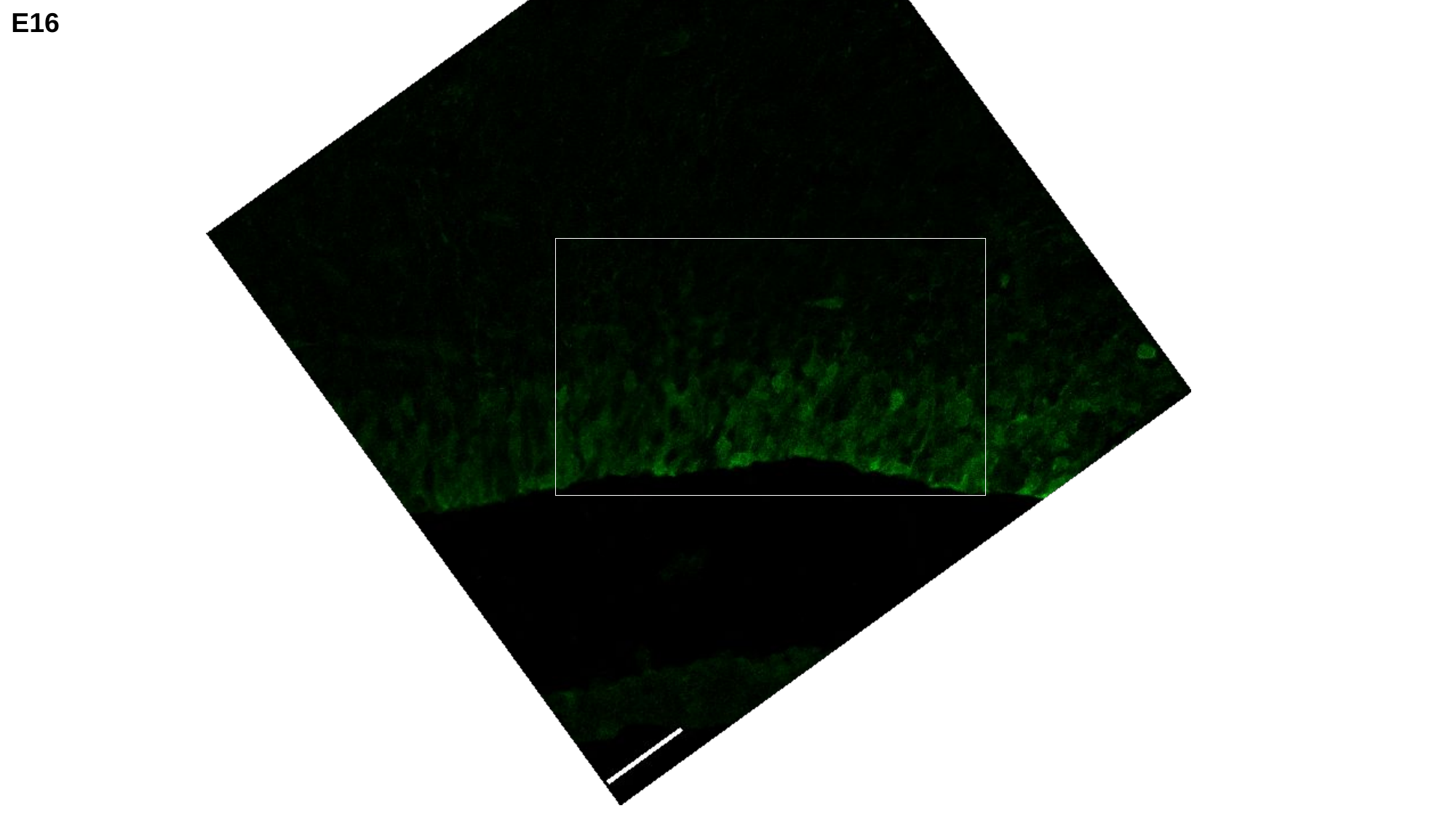

E16

## Slide 15
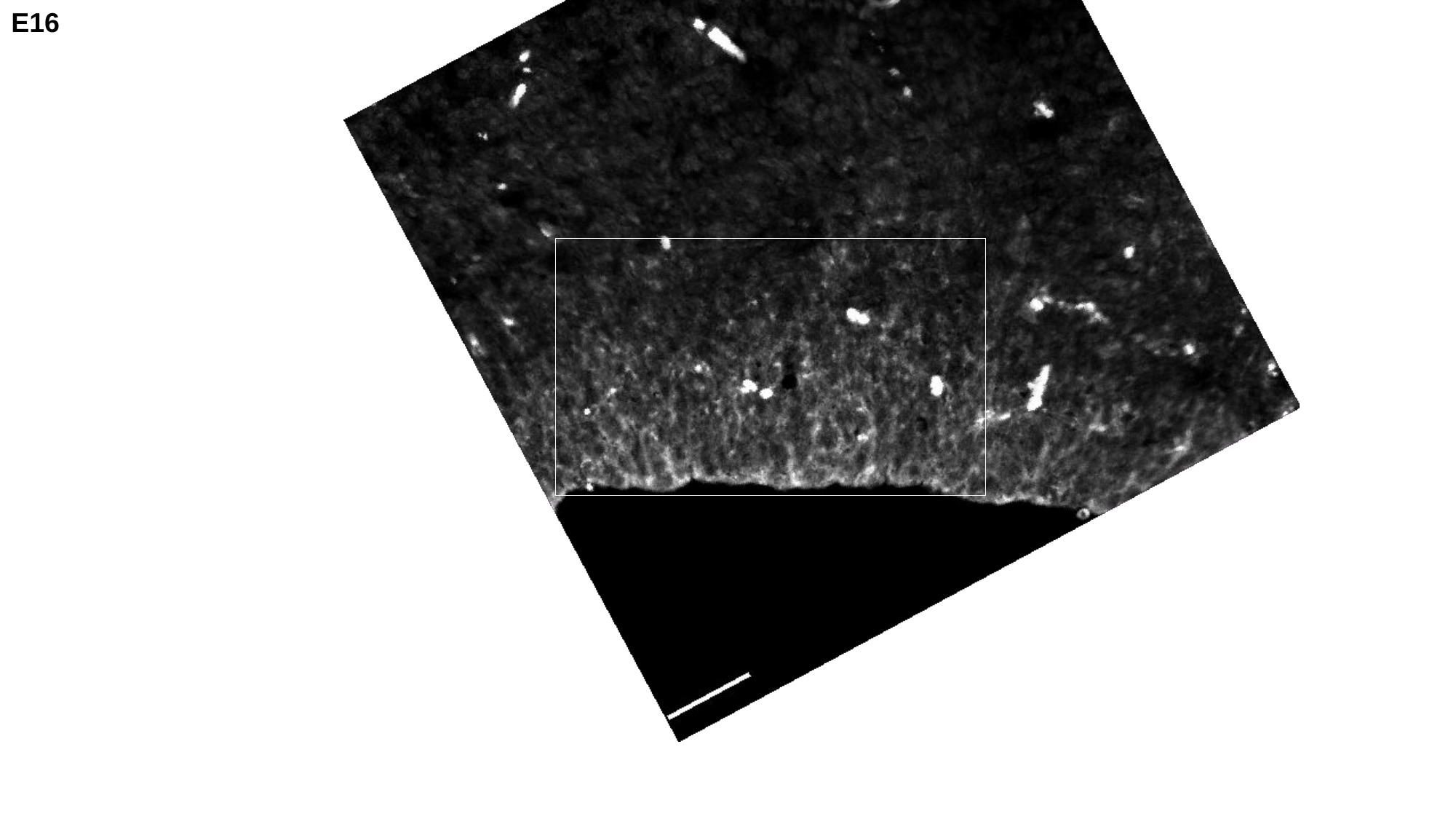

E16

## Slide 16
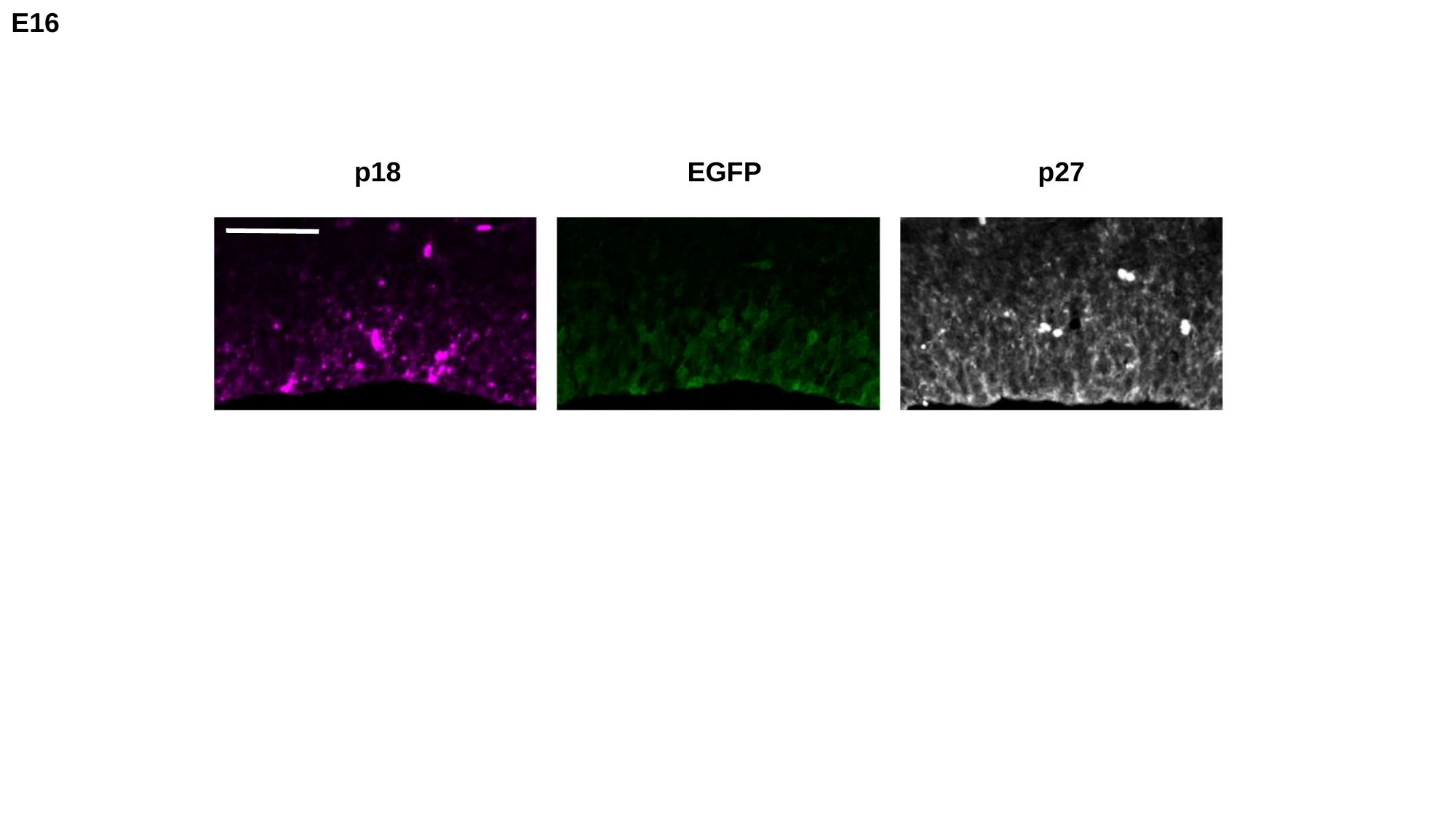

E16
p18
EGFP
p27

## Slide 17
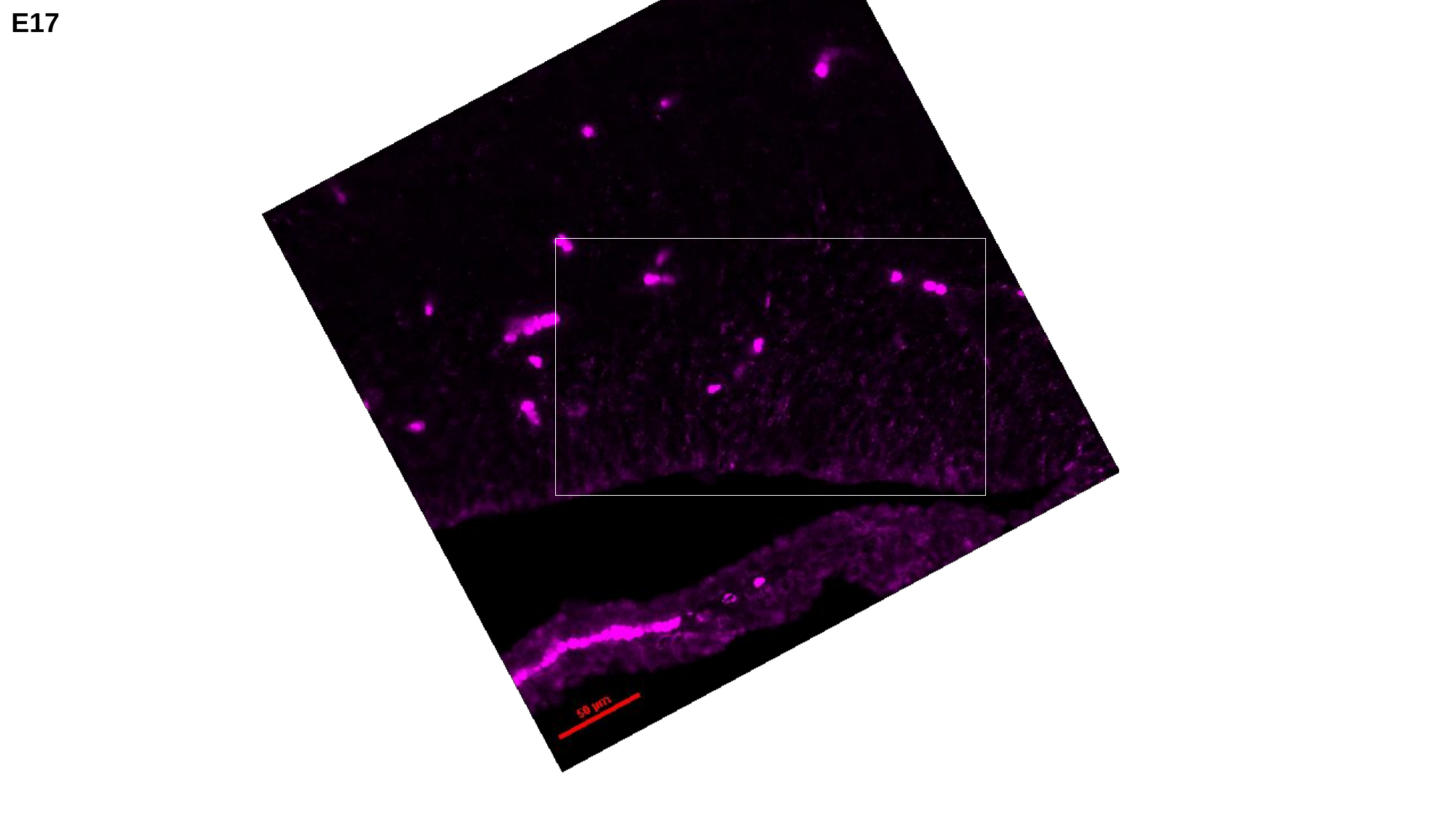

E17

## Slide 18
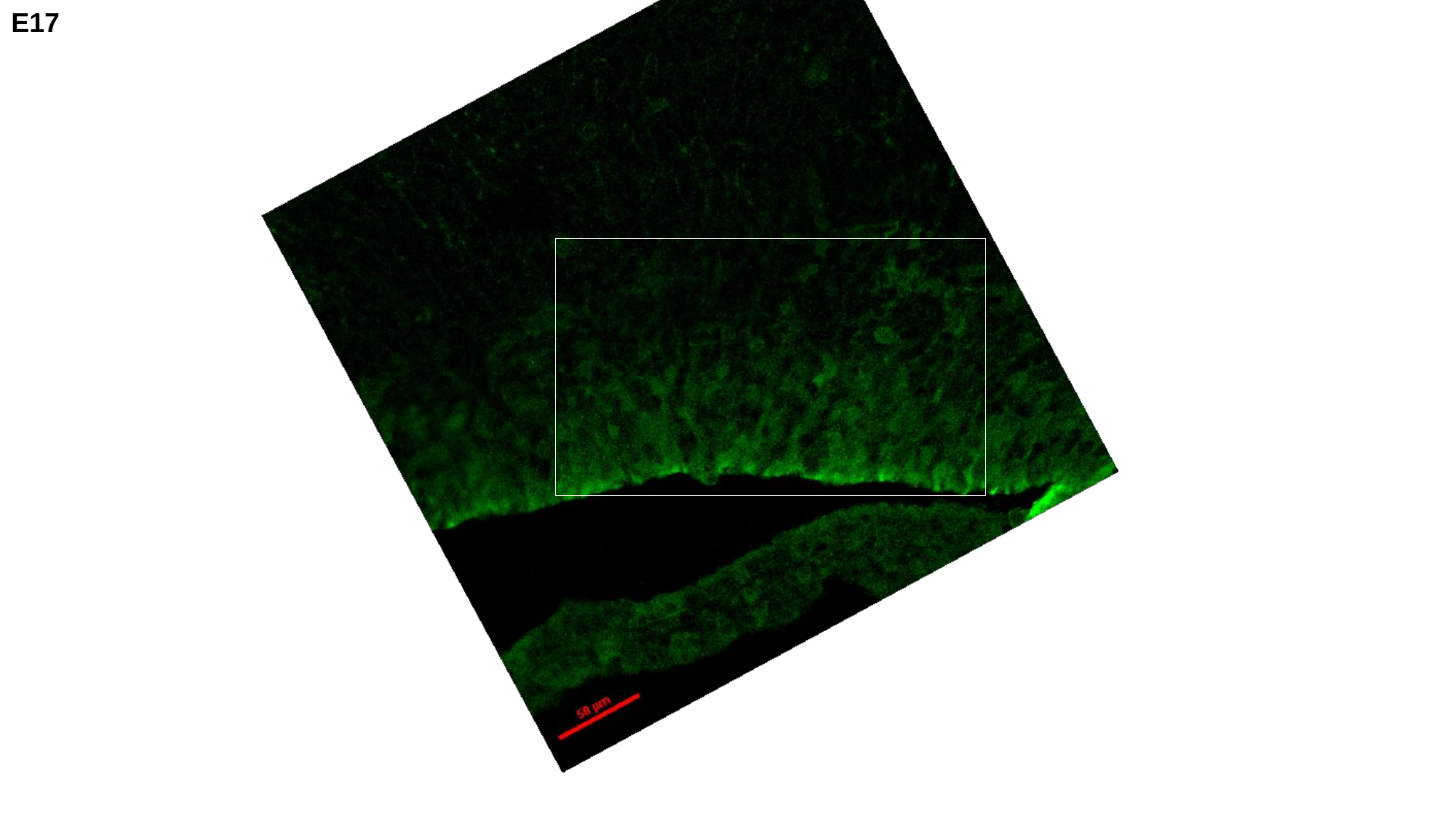

E17

## Slide 19
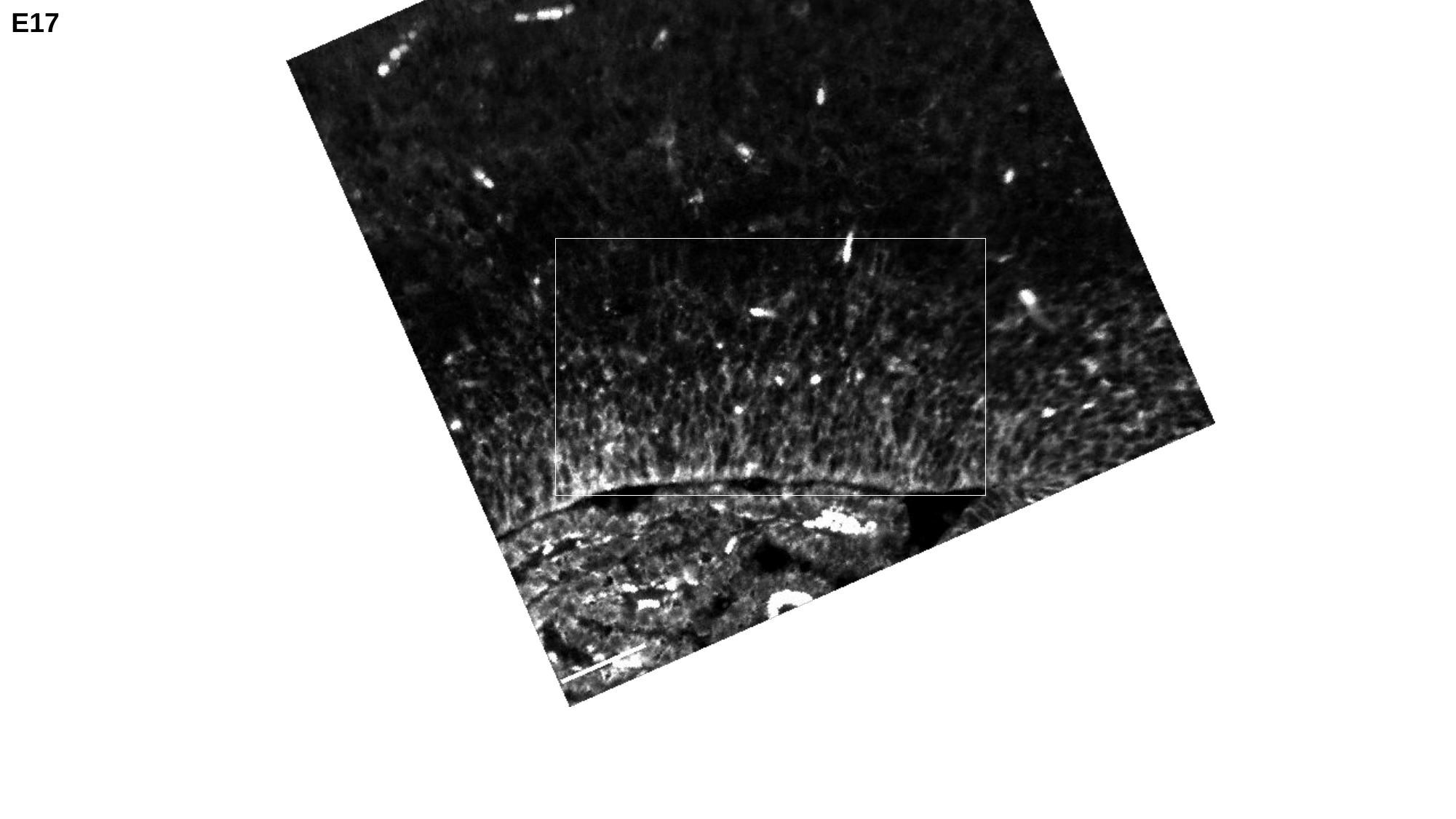

E17

## Slide 20
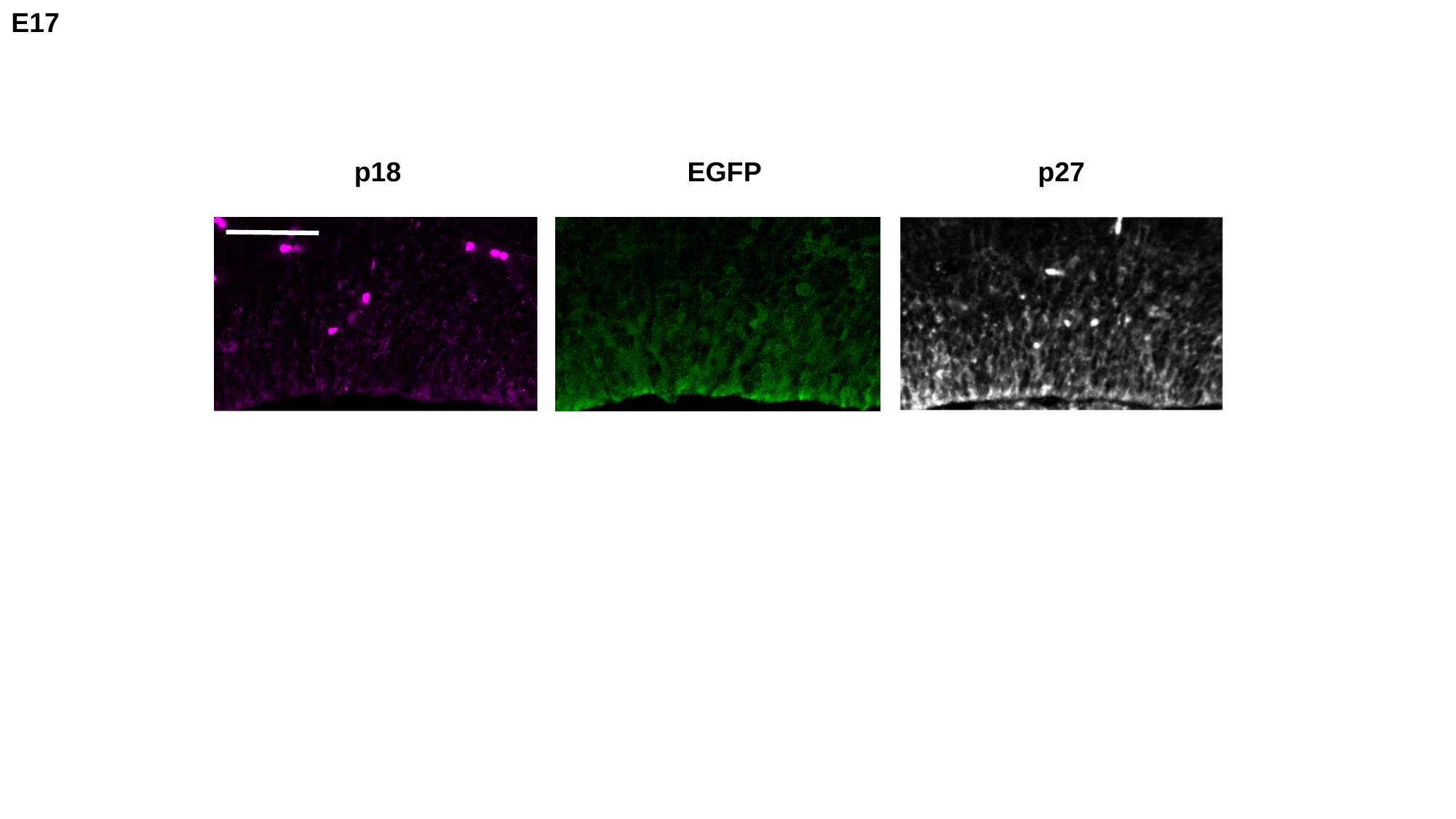

E17
p18
EGFP
p27
