# Supplementary figures and images for: Cyclin-dependent kinase inhibitor p18 regulates lineage transitions of excitatory neurons, astrocytes, and interneurons in the mouse cortex
Source: EMBO J. 2024 Dec 12;44(2):382–412. doi: 10.1038/s44318-024-00325-9 (PMC11730326; doi:10.1038/s44318-024-00325-9)

## Slide 1
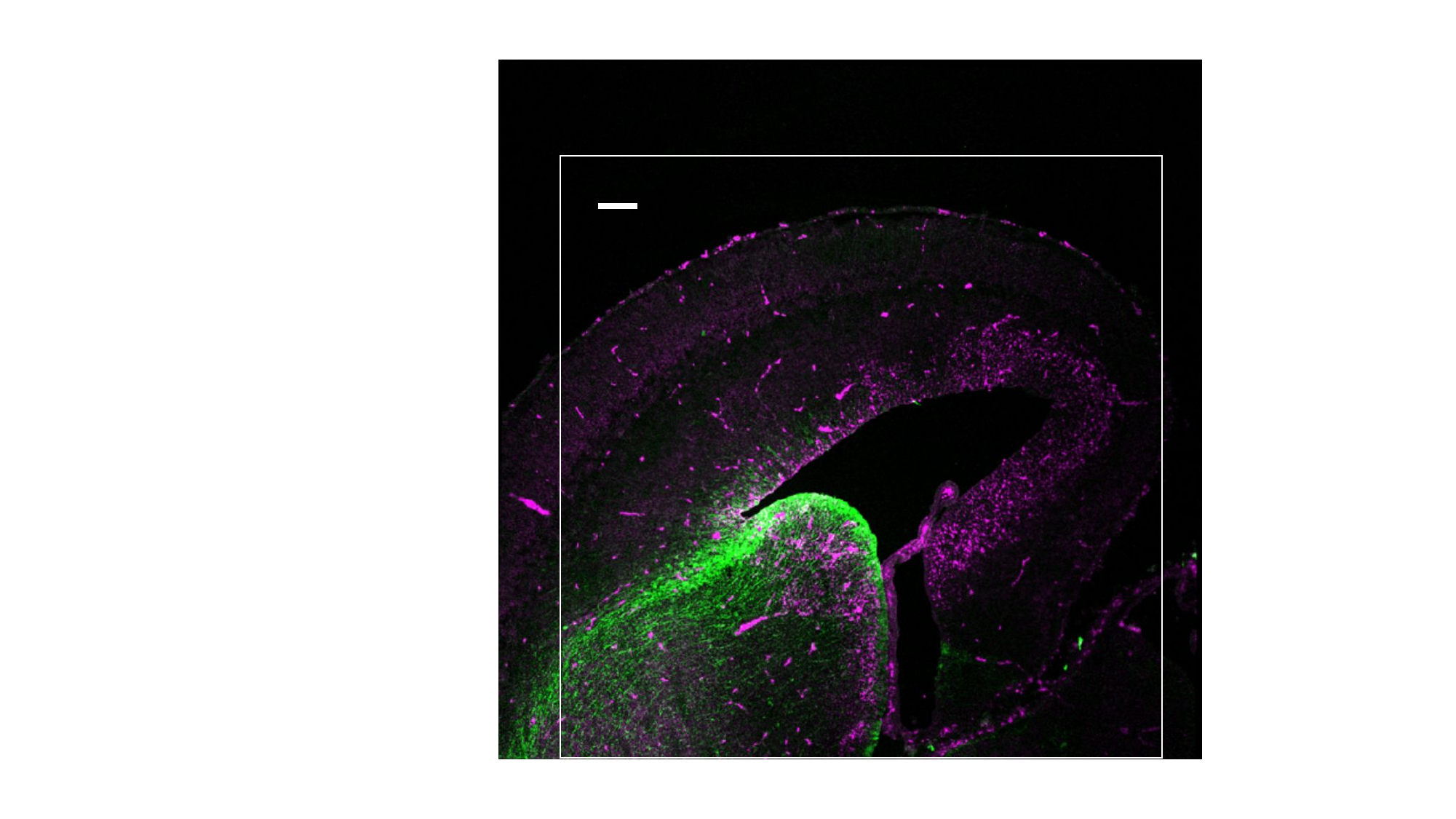

## Slide 2
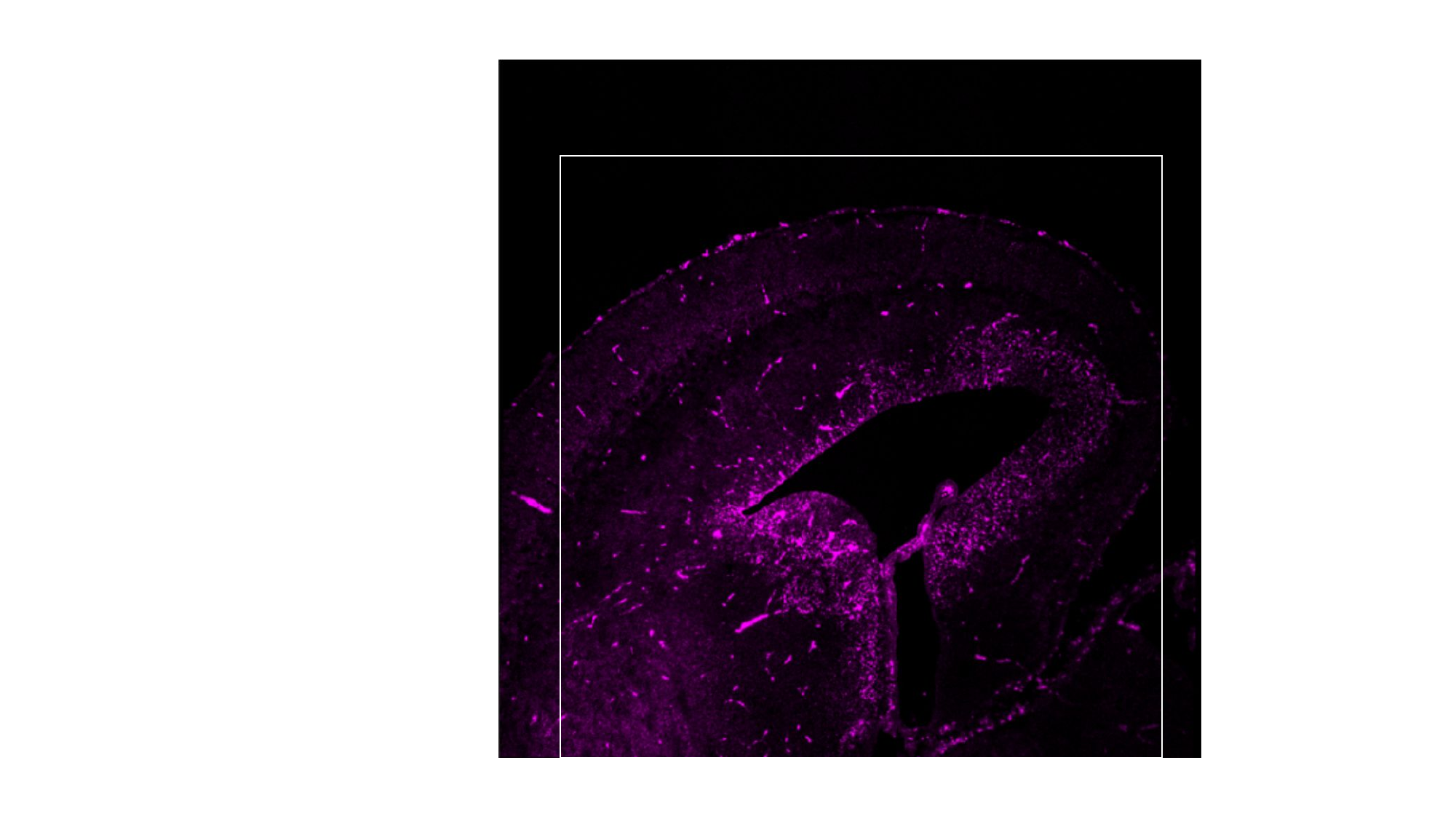

## Slide 3
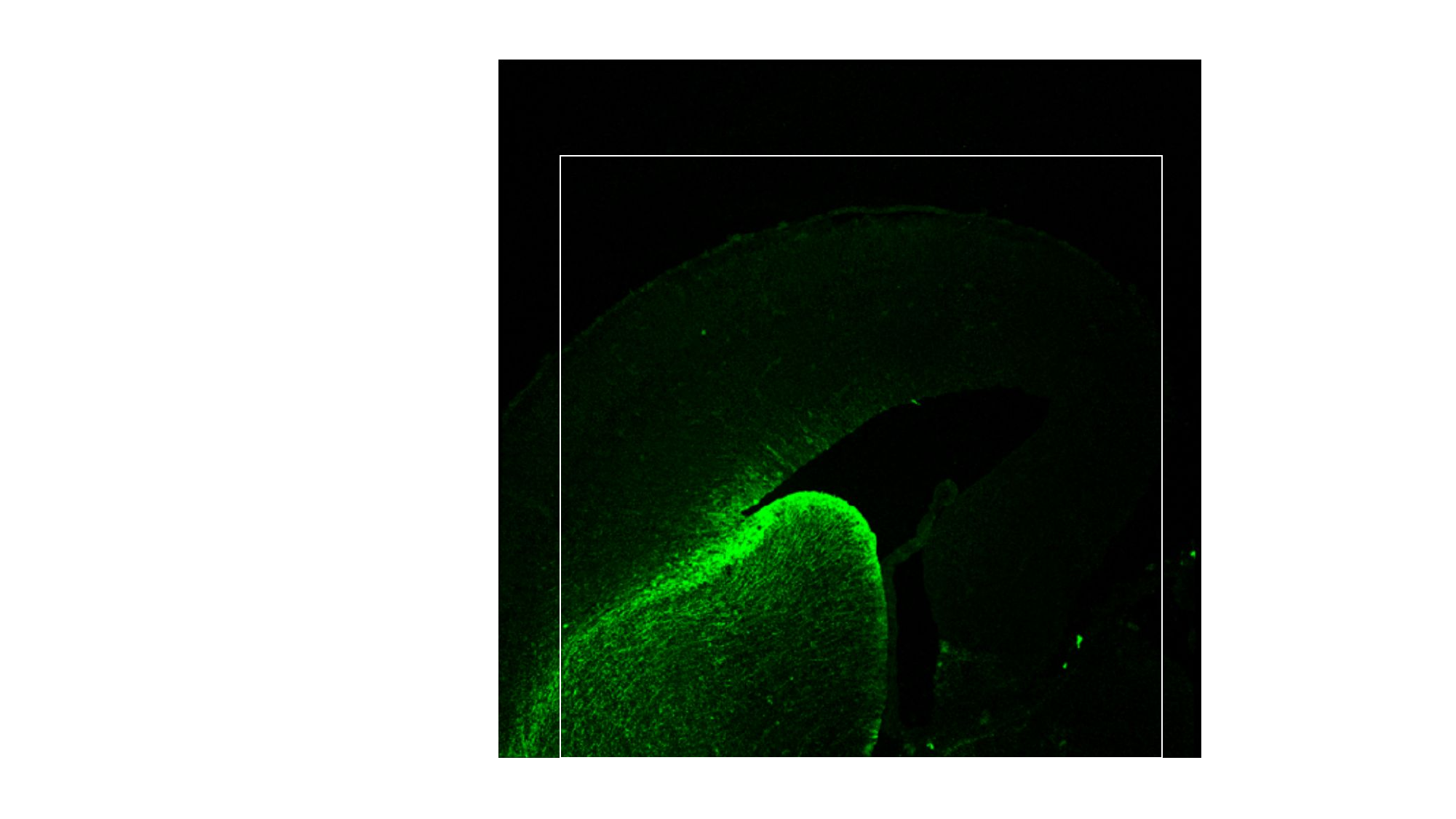

## Slide 4
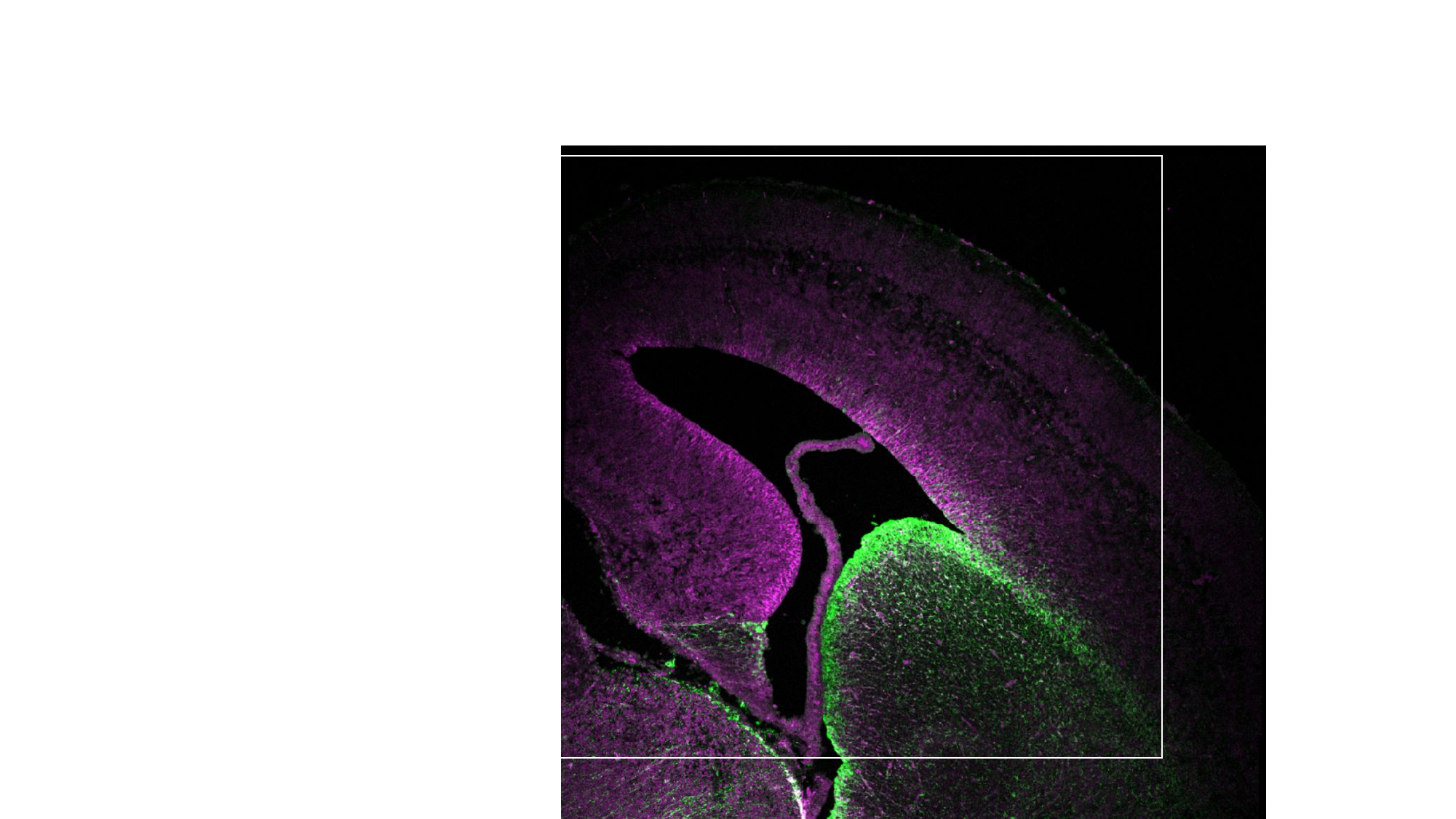

## Slide 5
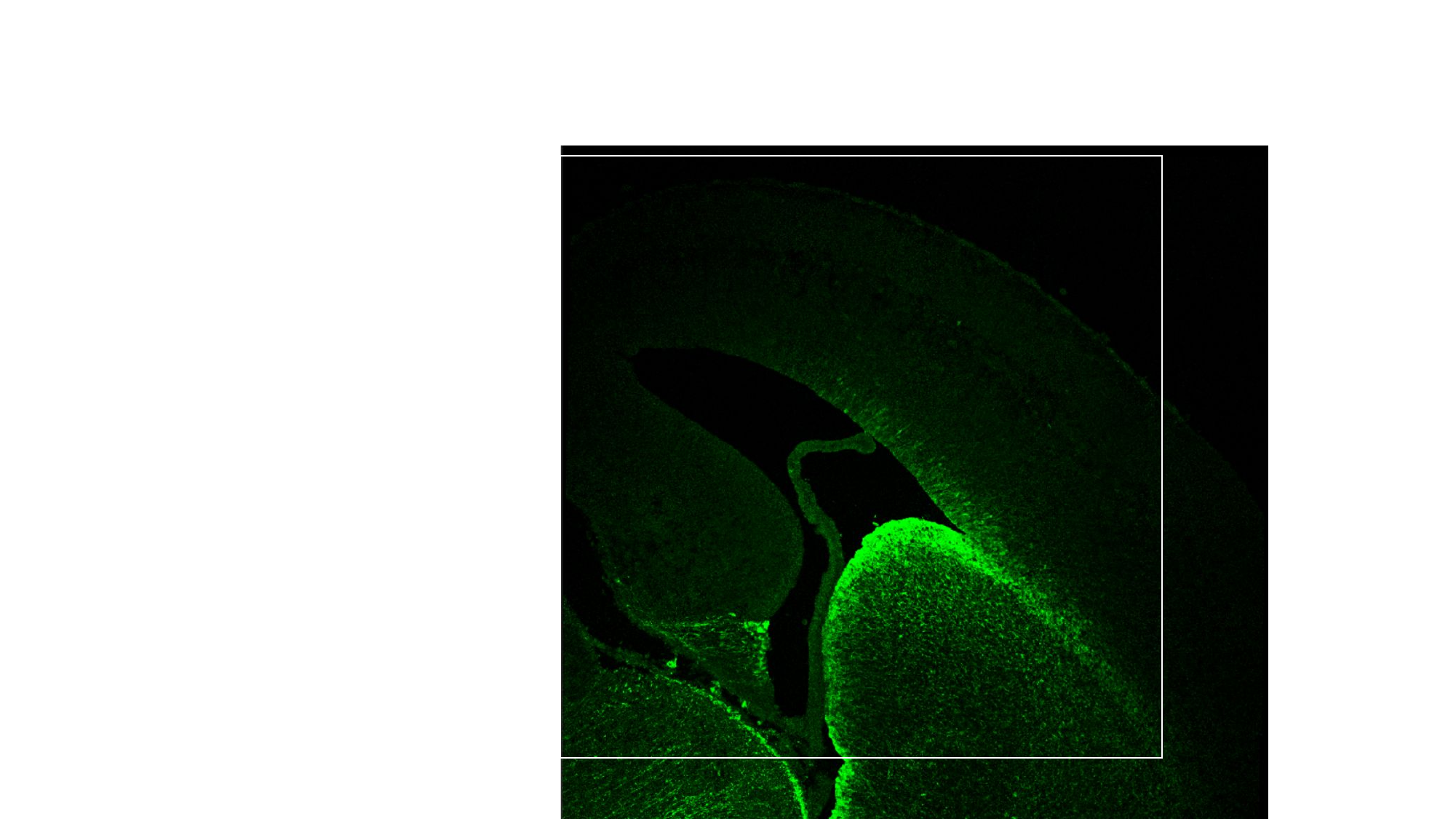

## Slide 6
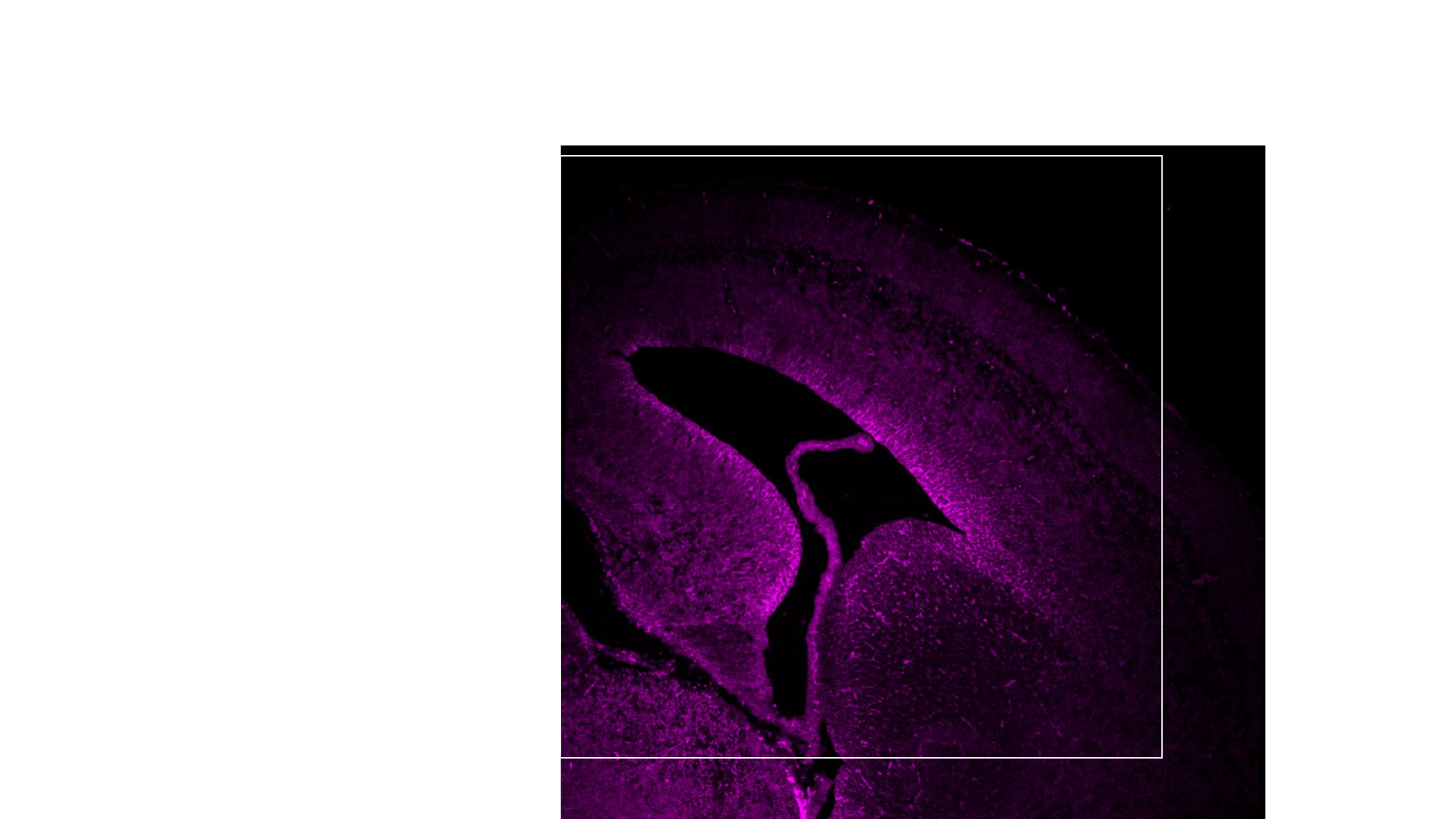

## Slide 7
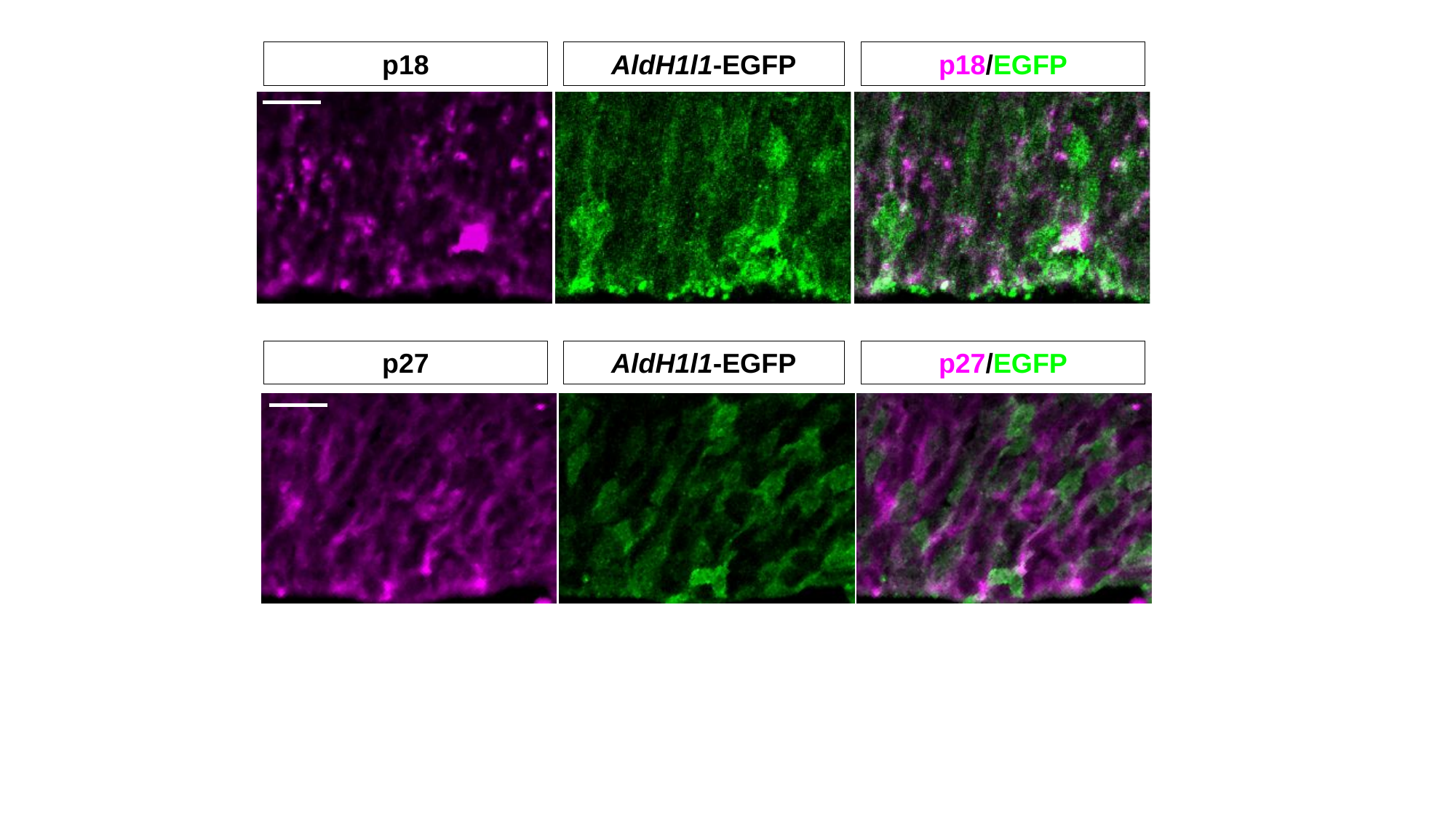

p18
AldH1l1-EGFP
p18/EGFP
p27
AldH1l1-EGFP
p27/EGFP

Supplement: Supplementary file 3 — Source data Fig. 1 [file 44318_2024_325_MOESM3_ESM.zip › 1A.pptx]

## Slide 1
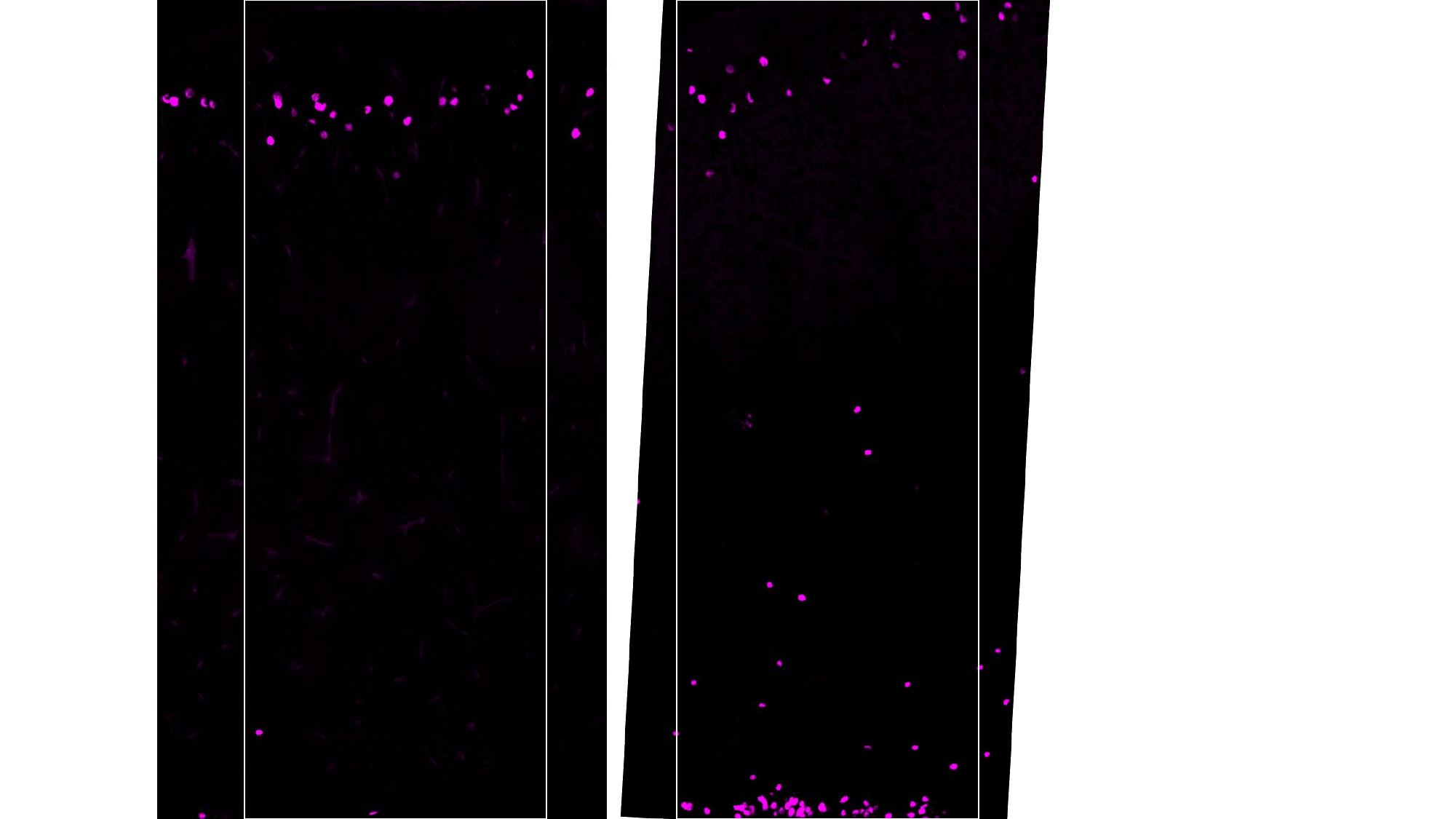

## Slide 2
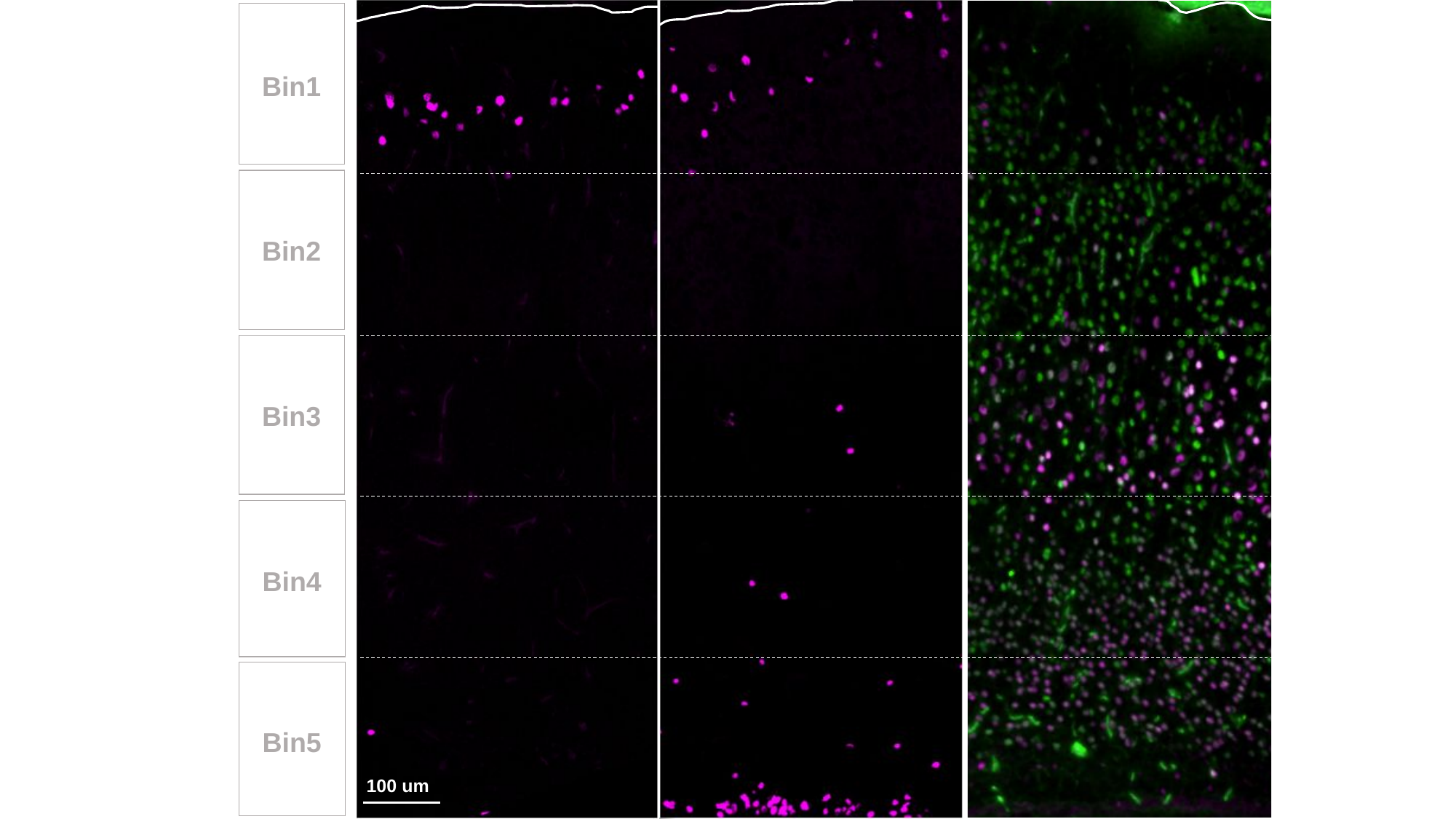

Satb2/Ctip2
Control
p18
Bin1
Bin2
Bin3
Bin4
Bin5
100 um

Supplement: Supplementary file 5 — Source data Fig. 3 [file 44318_2024_325_MOESM5_ESM.zip › 3C.pptx]

## Slide 1
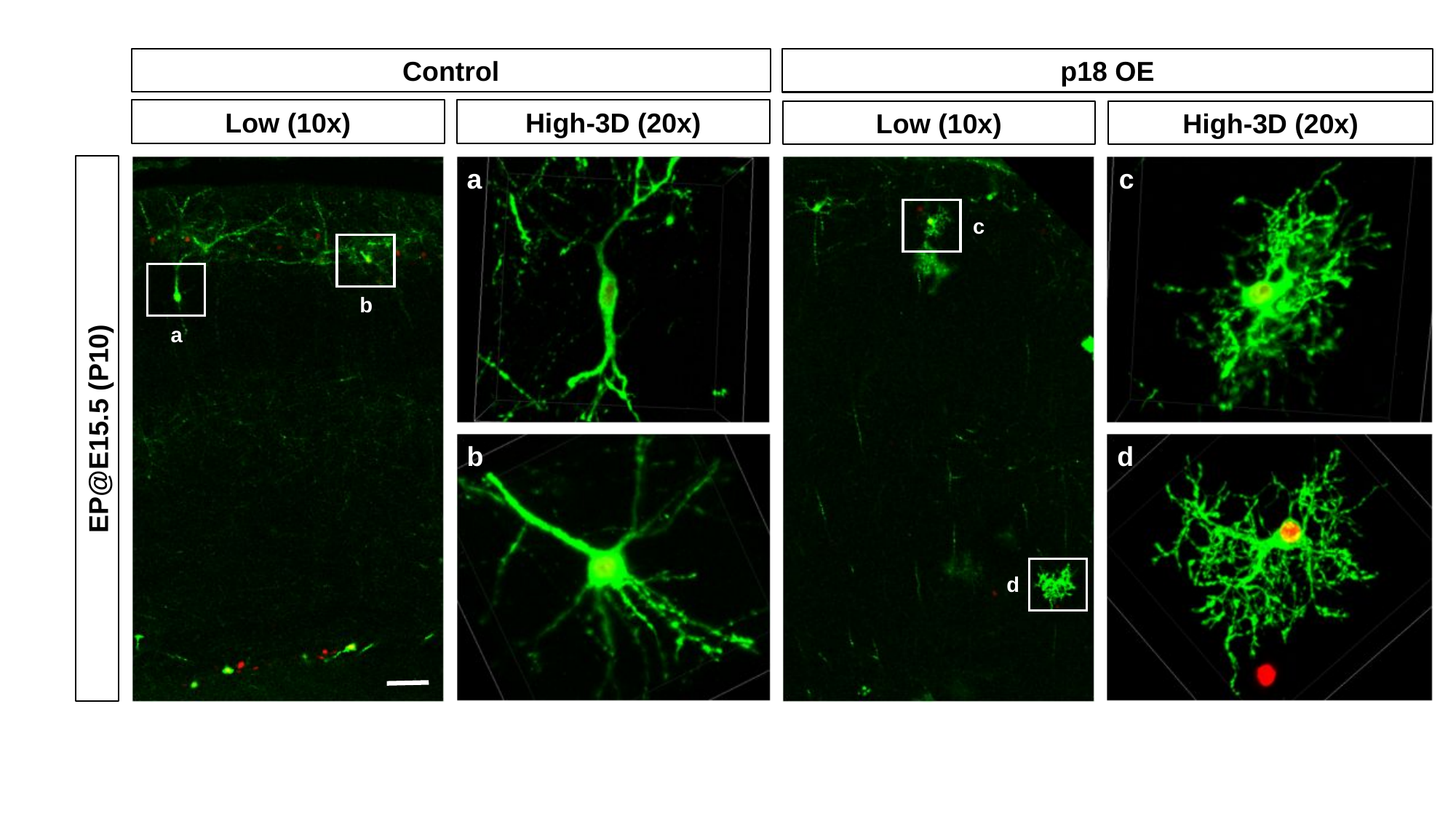

Control
p18 OE
Low (10x)
High-3D (20x)
Low (10x)
High-3D (20x)
a
c
c
b
a
EP@E15.5 (P10)
b
d
d

## Slide 2
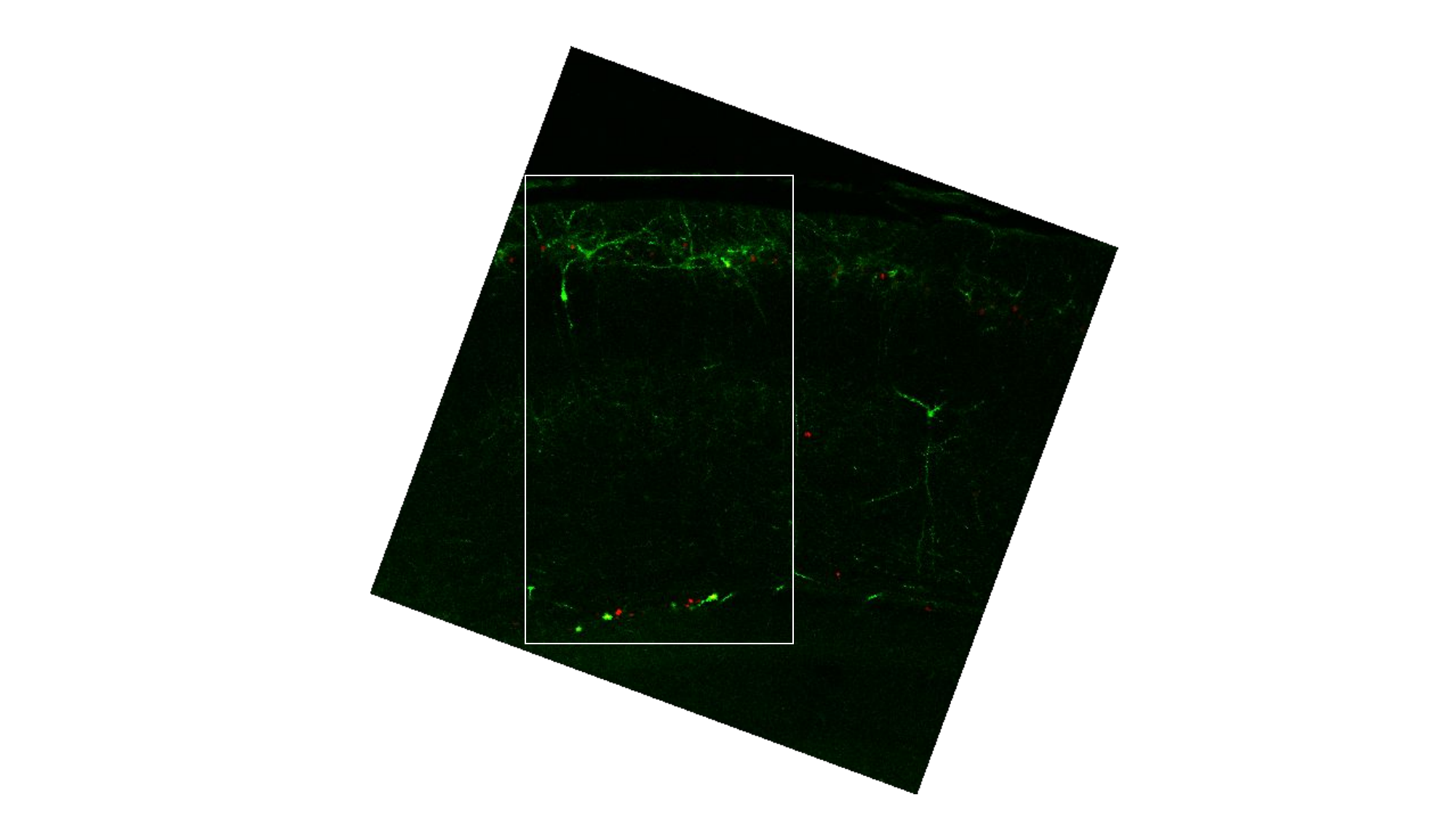

## Slide 3
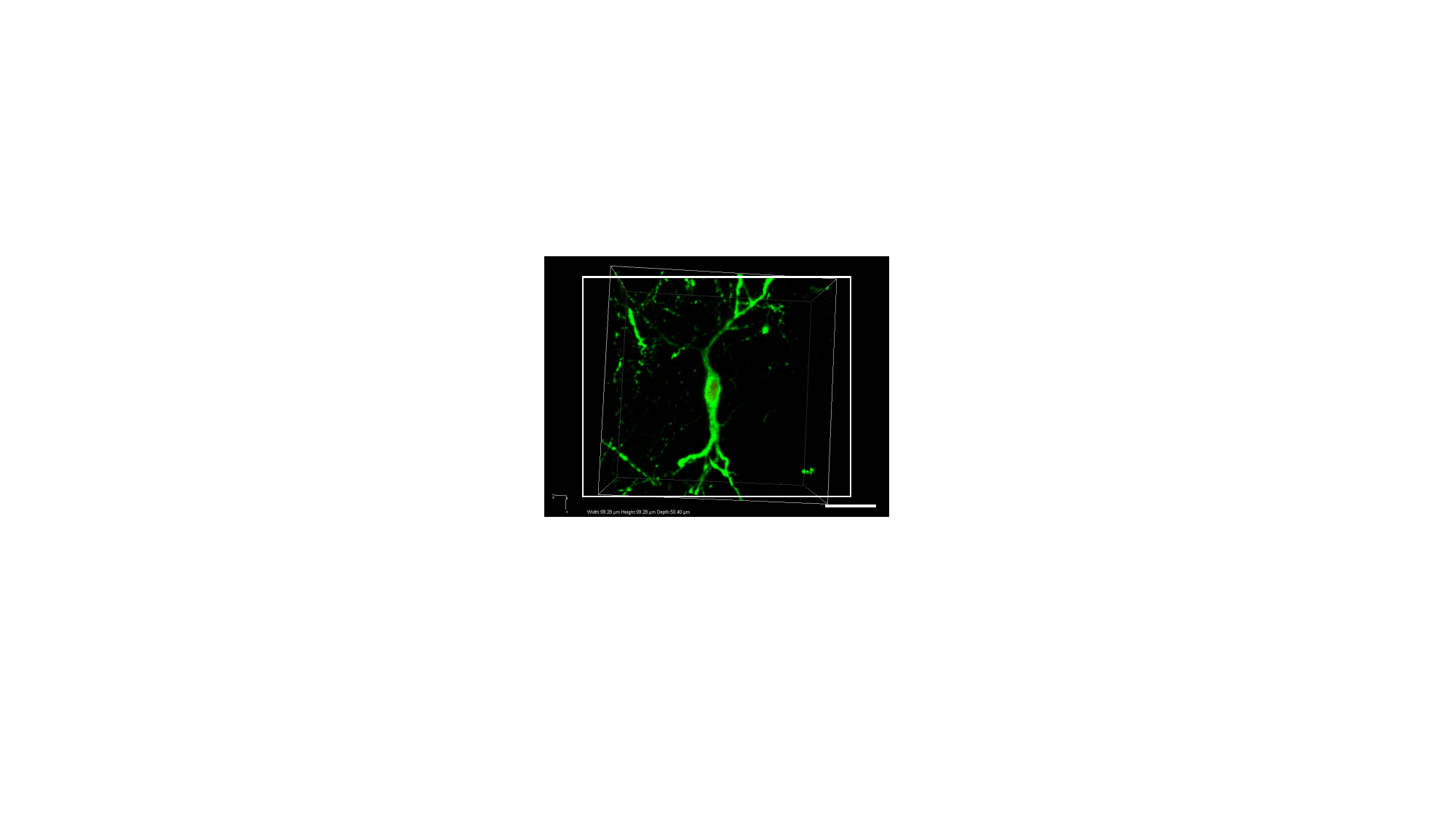

## Slide 4
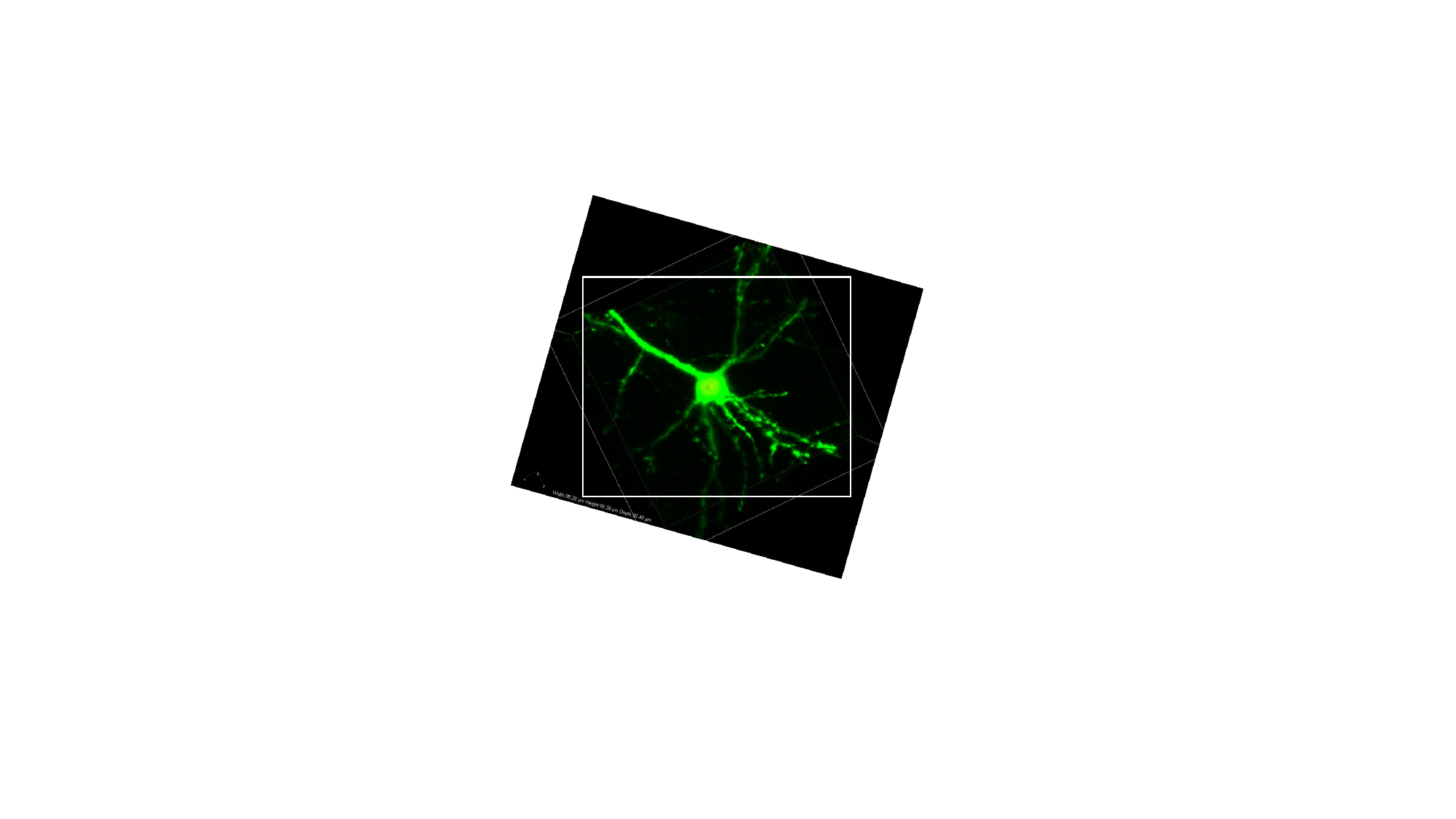

## Slide 5
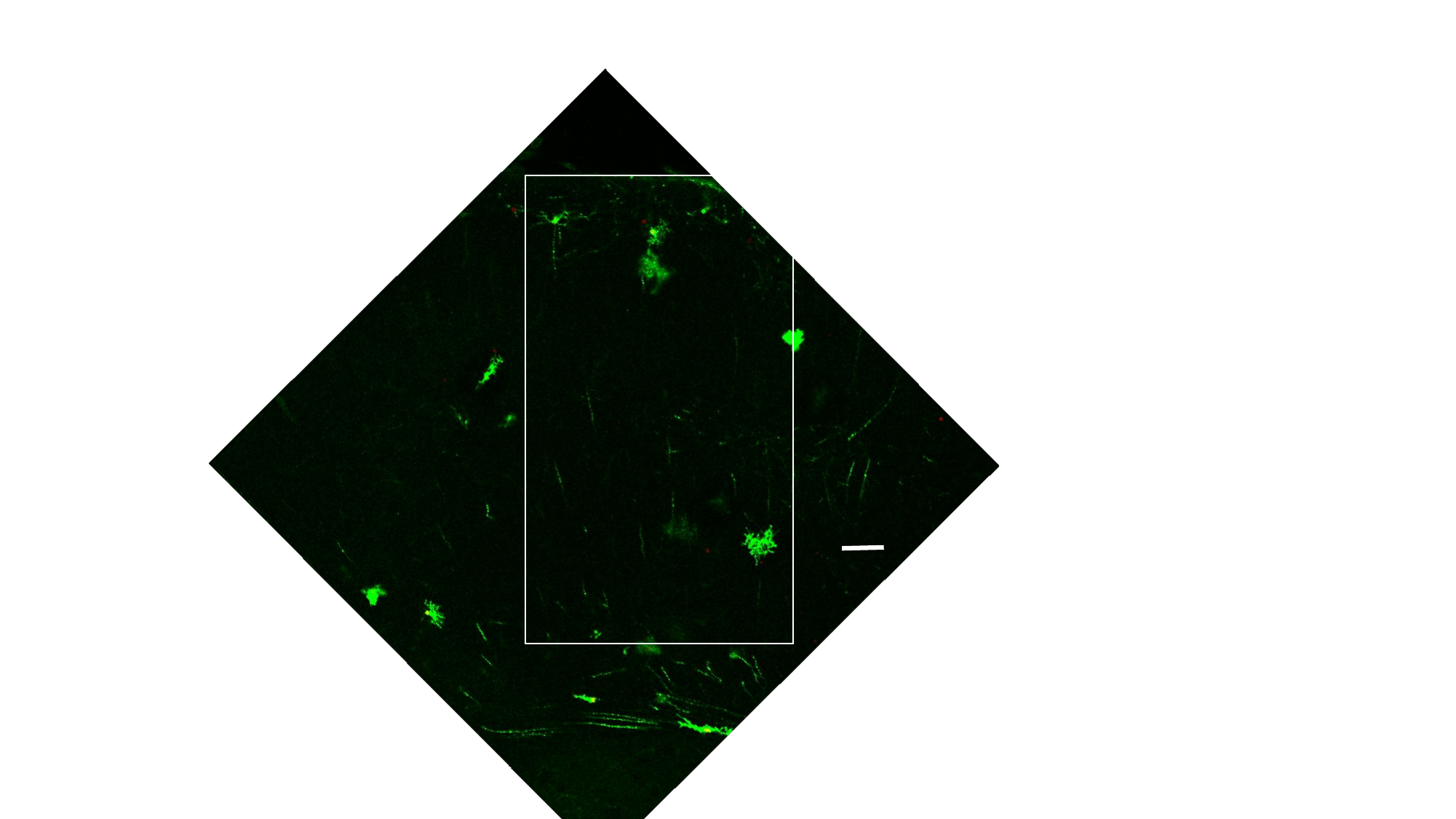

## Slide 6
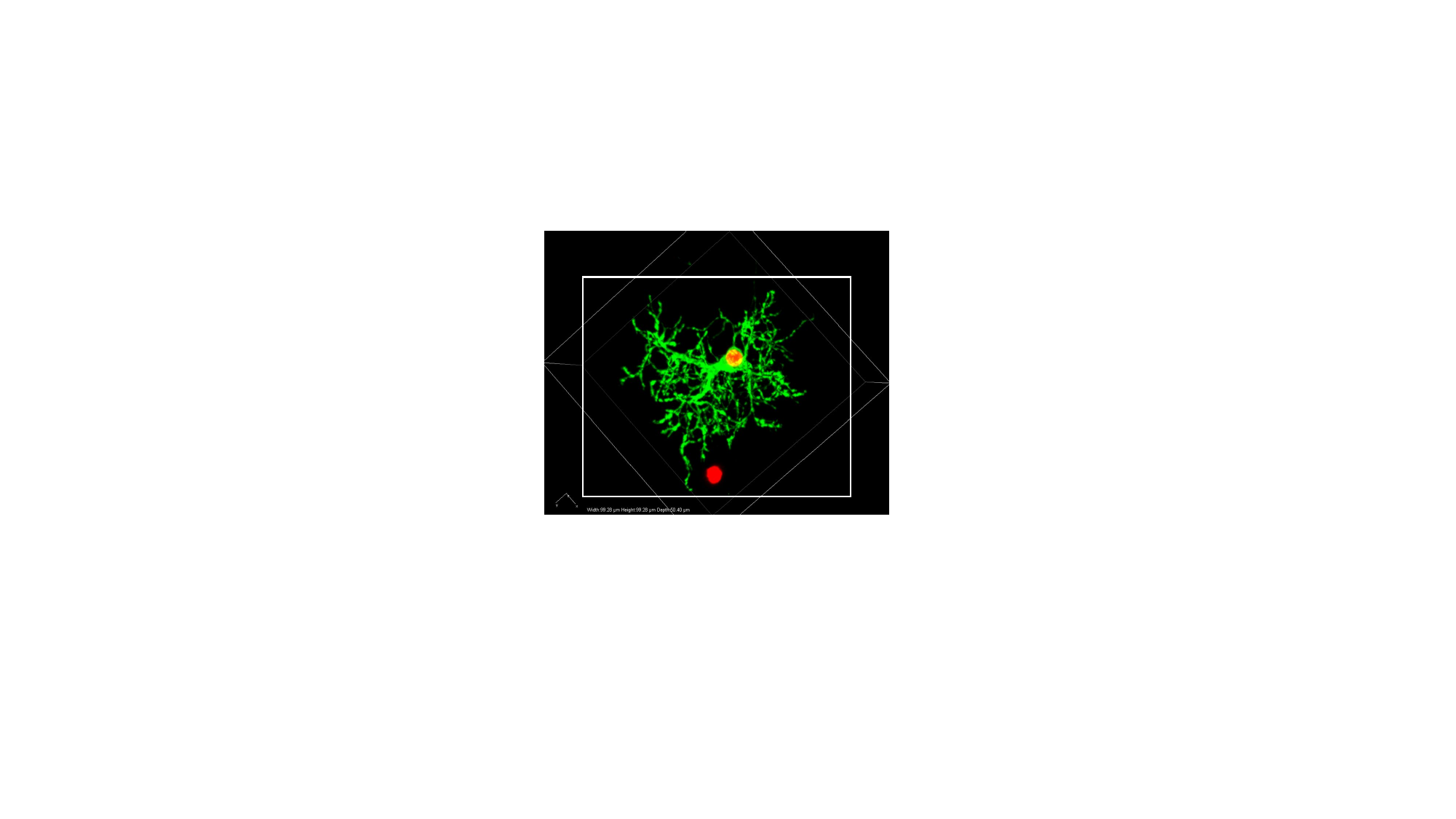

## Slide 7
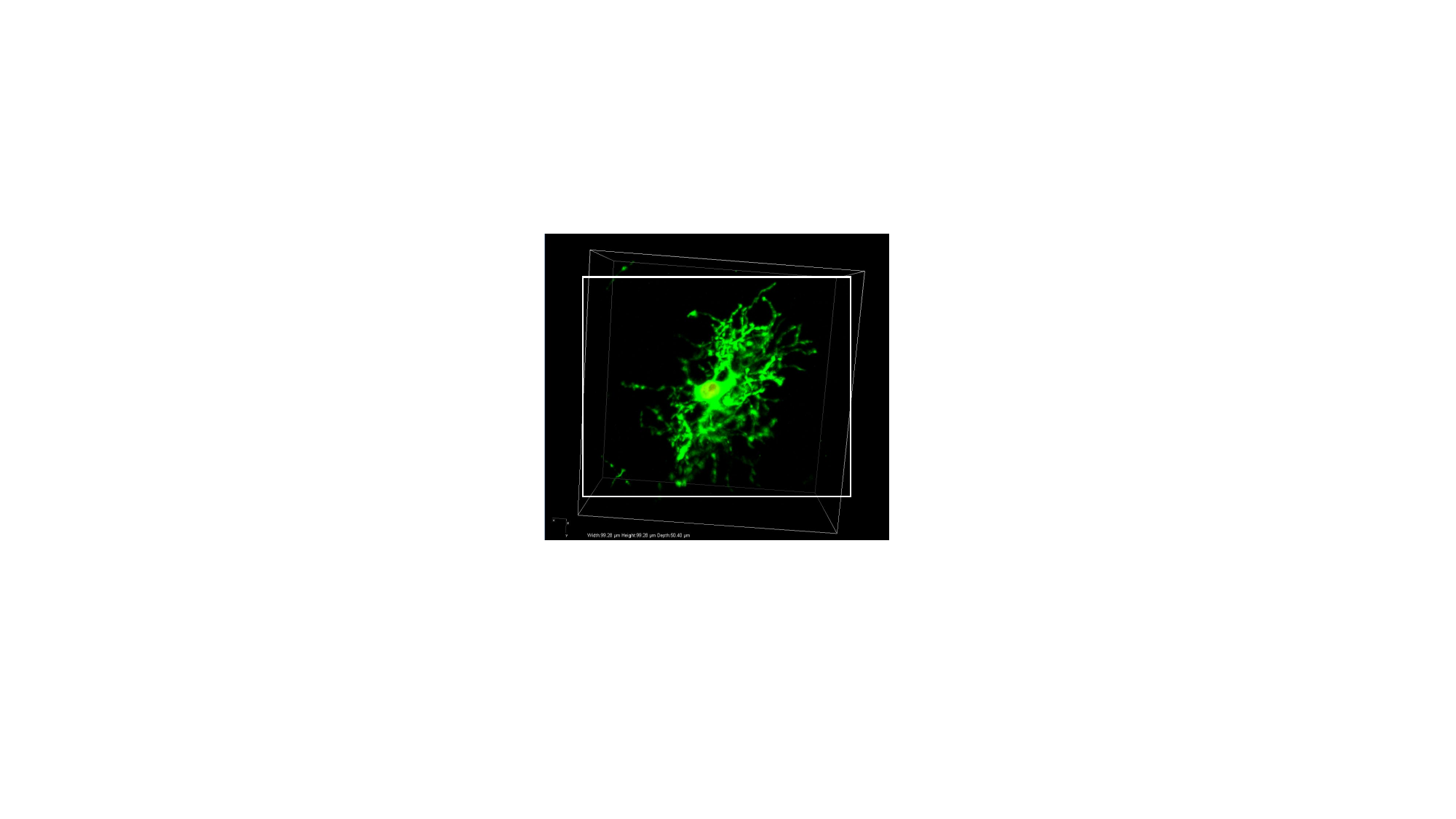

Supplement: Supplementary file 6 — Source data Fig. 4 [file 44318_2024_325_MOESM6_ESM.zip › 4A.pptx]

## Slide 1
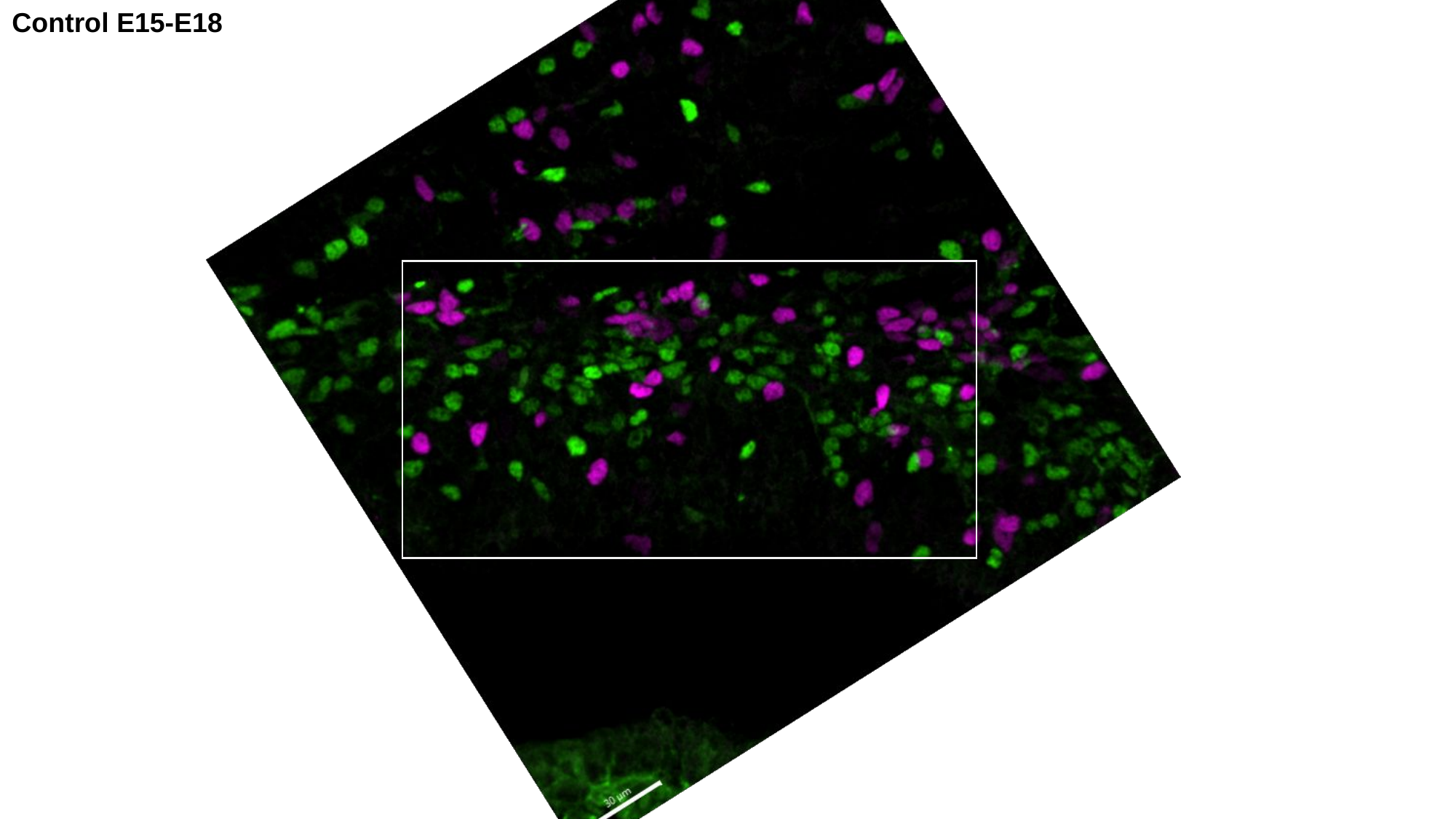

Control E15-E18

## Slide 2
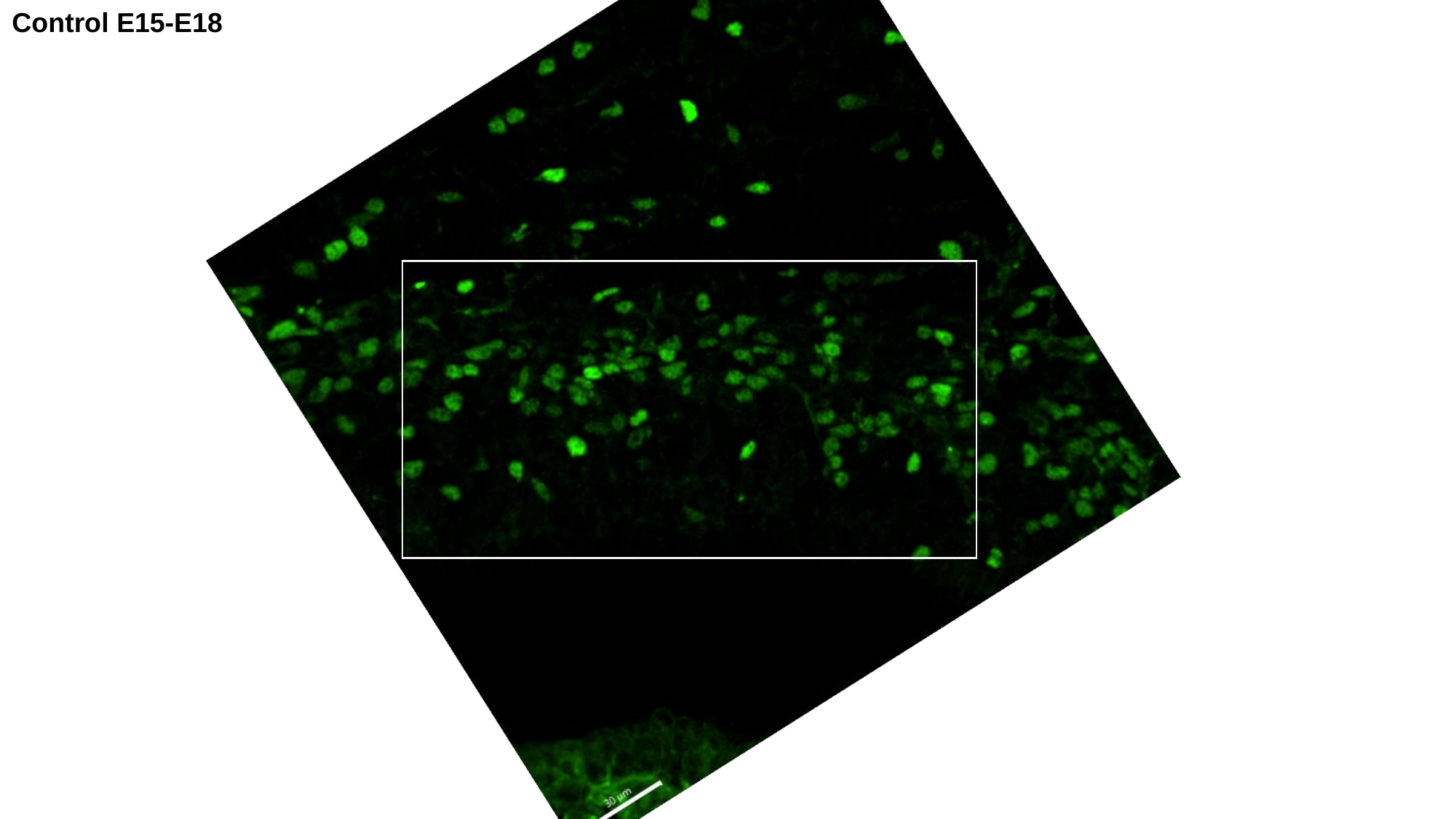

Control E15-E18

## Slide 3
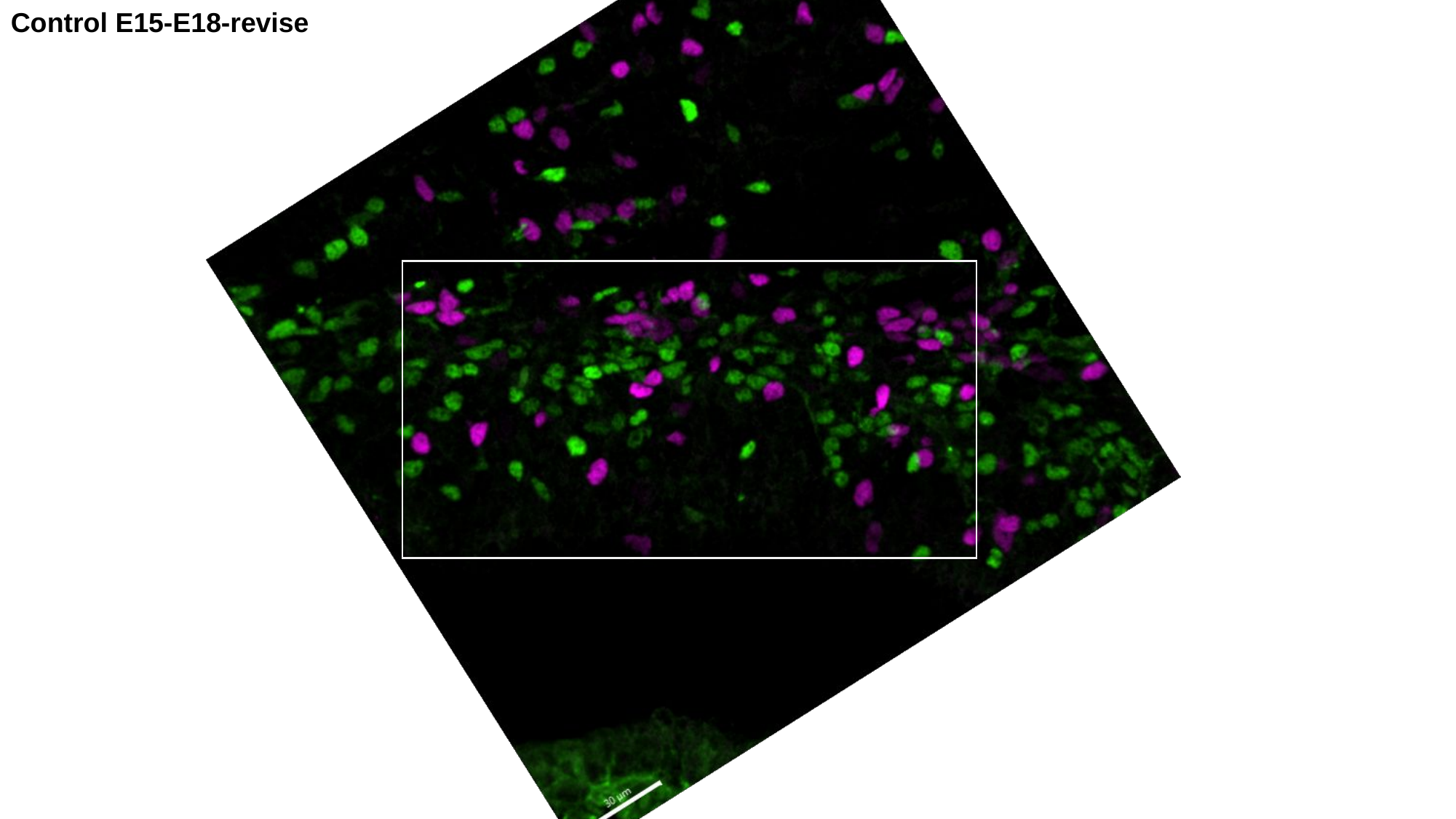

Control E15-E18-revise

## Slide 4
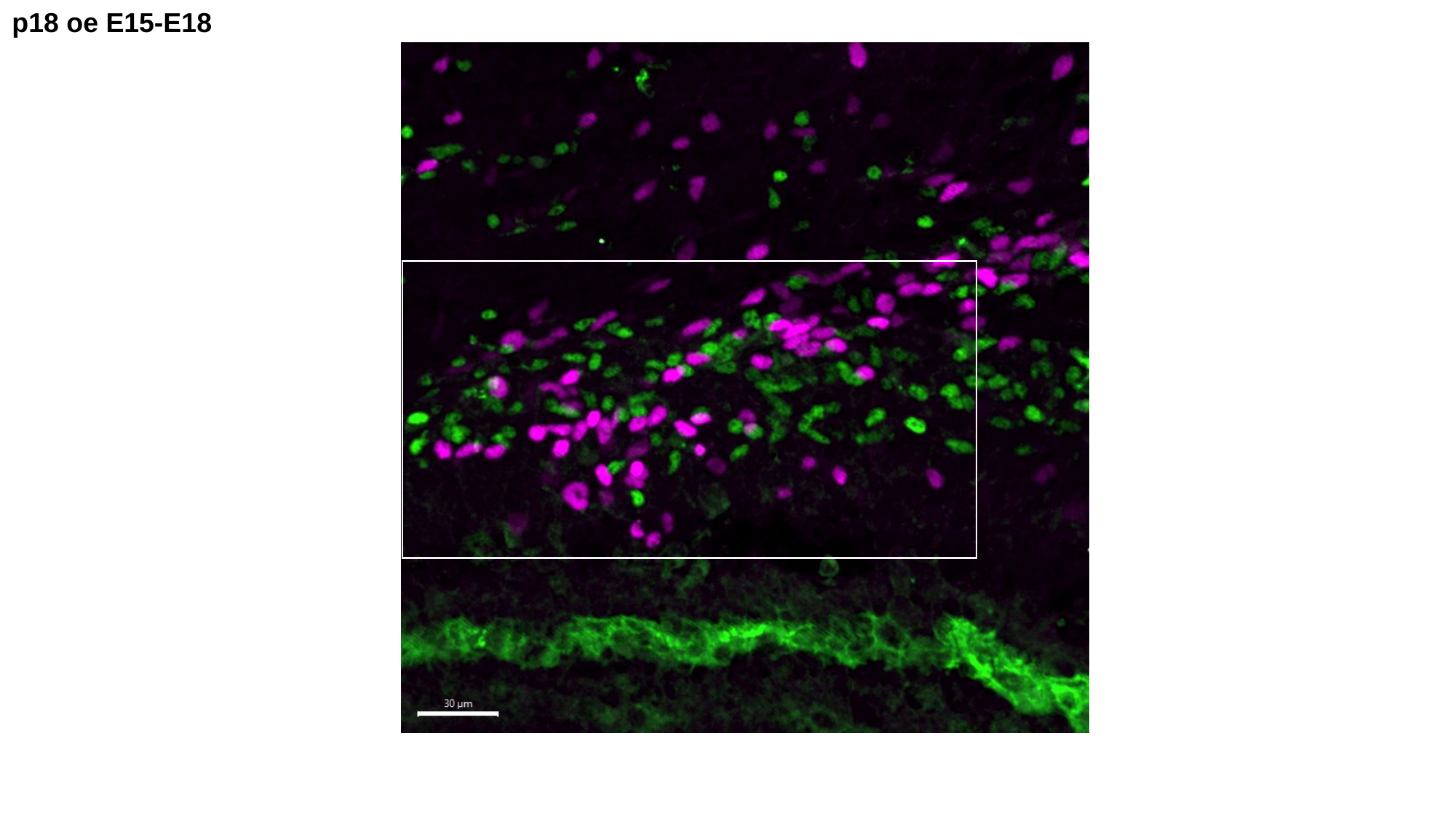

p18 oe E15-E18

## Slide 5
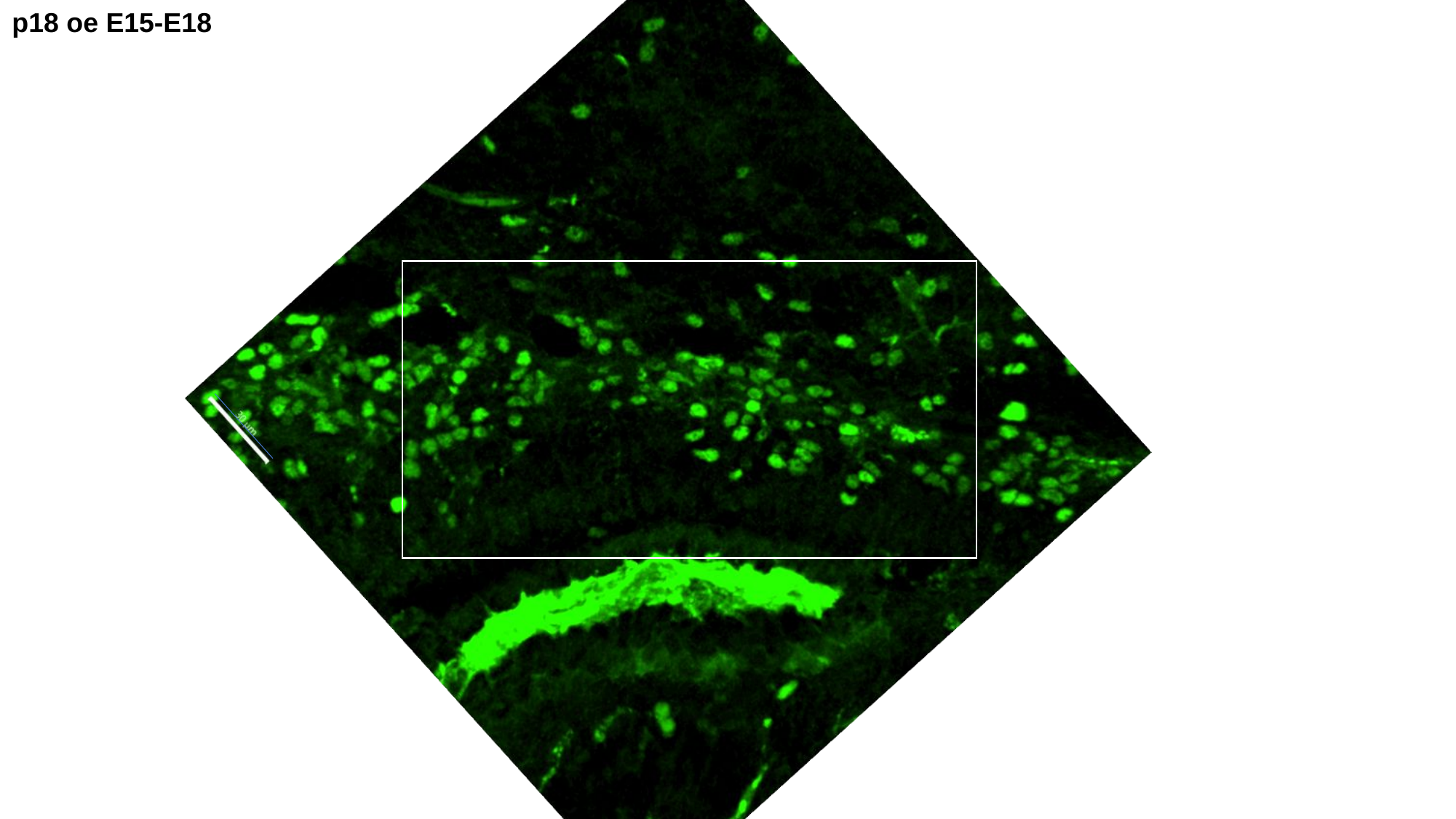

p18 oe E15-E18

## Slide 6
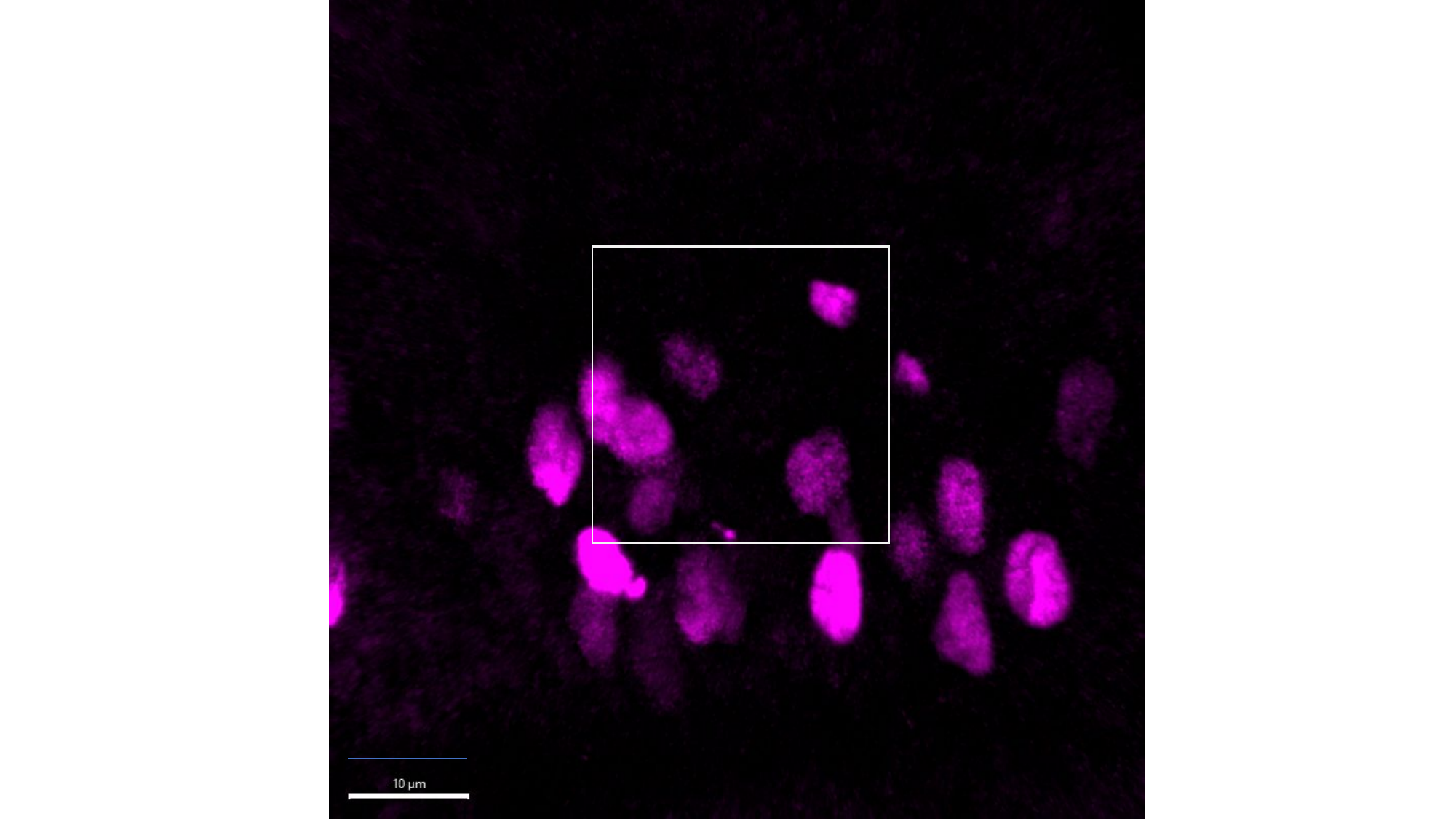

Supplement: Supplementary file 9 — Source data Fig. 7 [file 44318_2024_325_MOESM9_ESM.zip › 7B.pptx]

## Slide 1
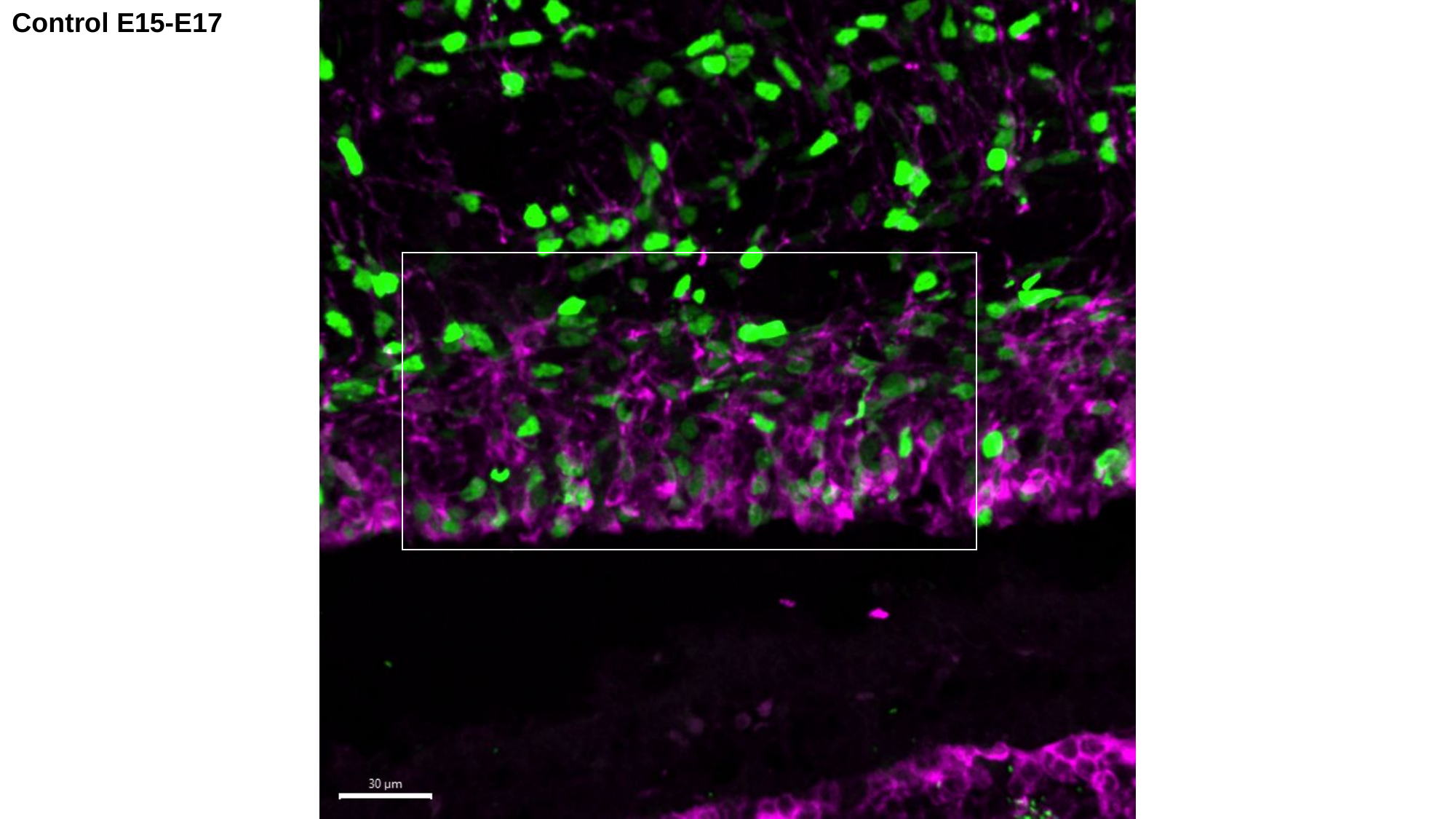

Control E15-E17

## Slide 2
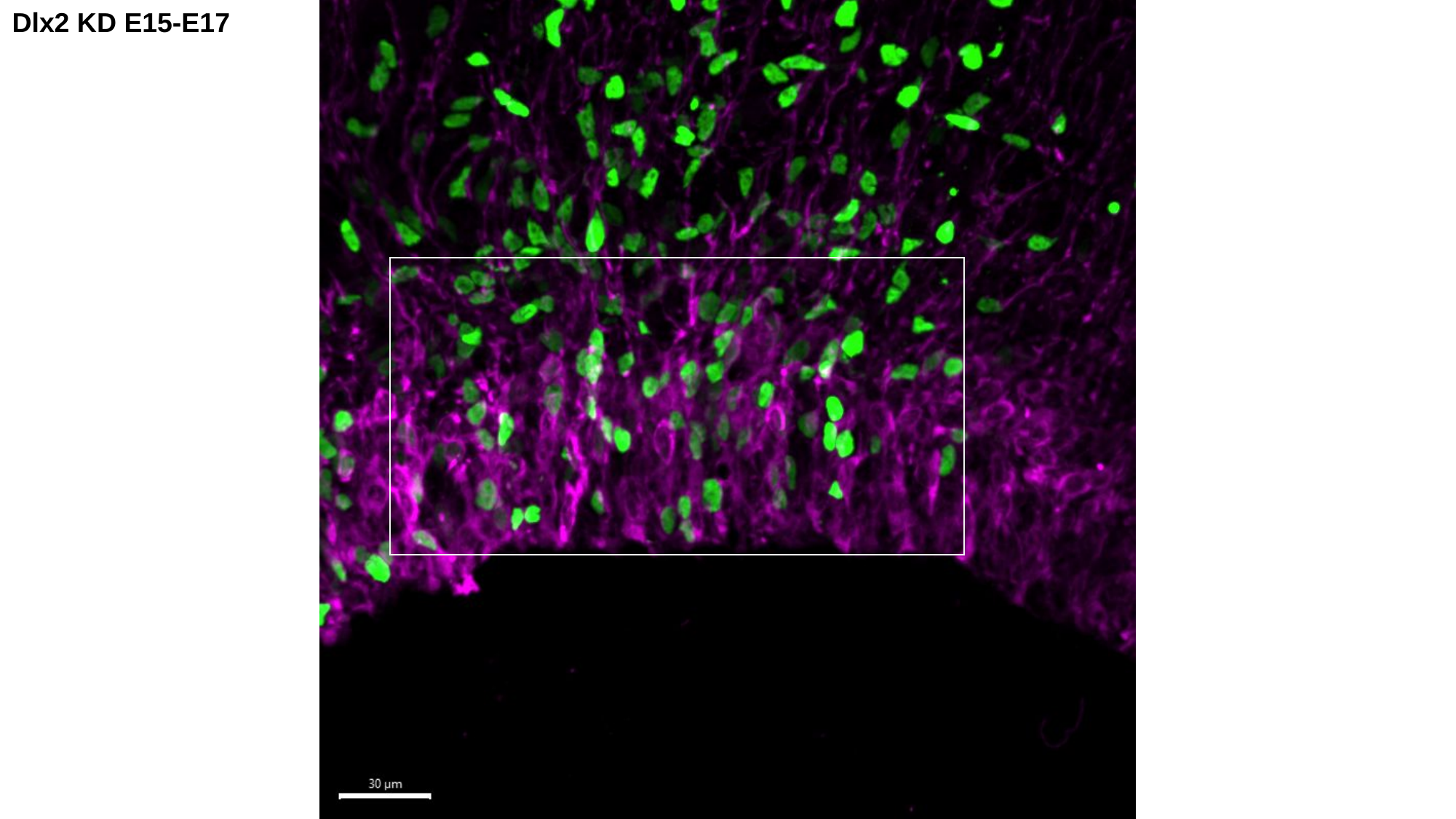

Dlx2 KD E15-E17

## Slide 3
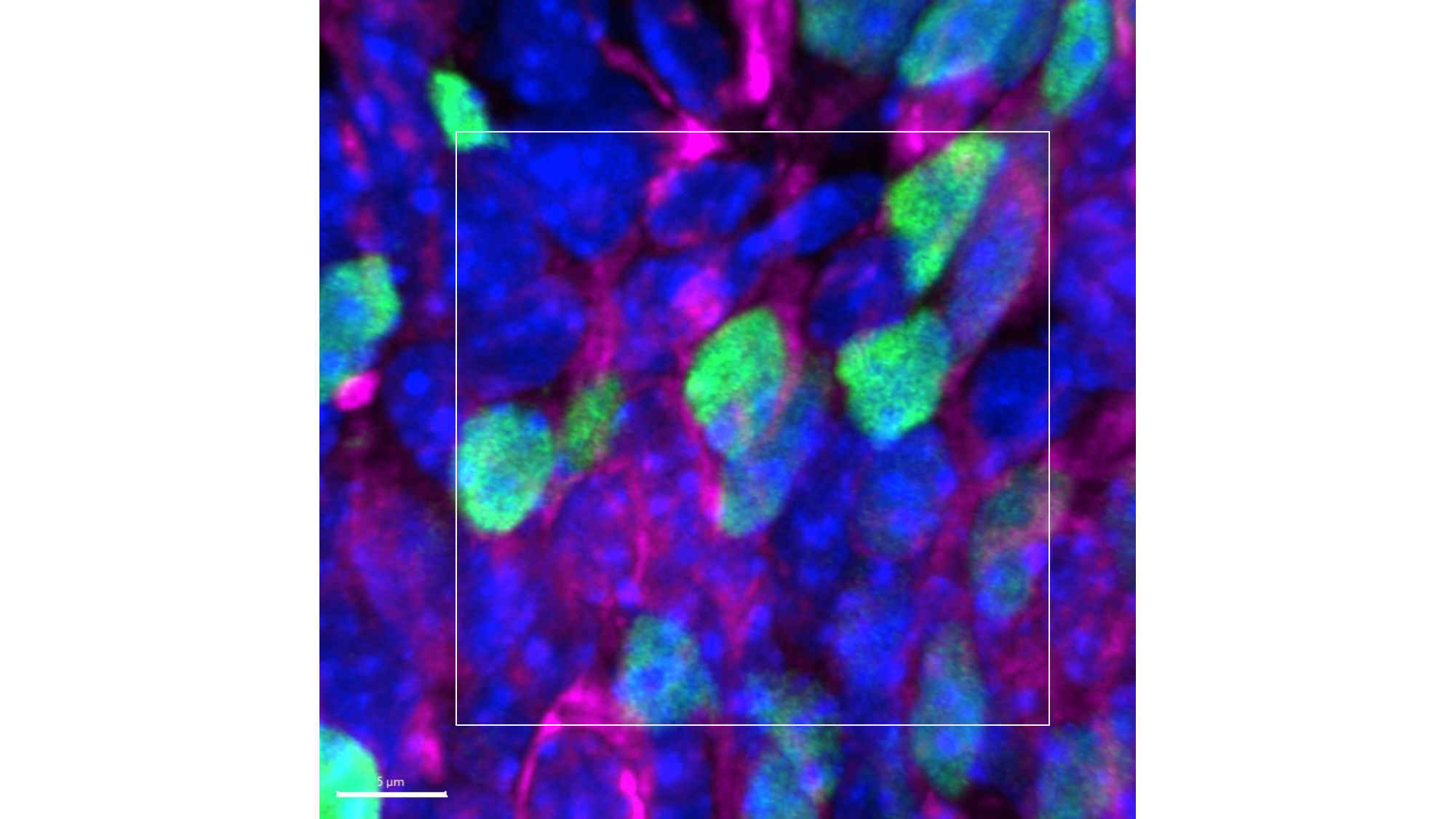

Supplement: Supplementary file 9 — Source data Fig. 7 [file 44318_2024_325_MOESM9_ESM.zip › 7G.pptx]

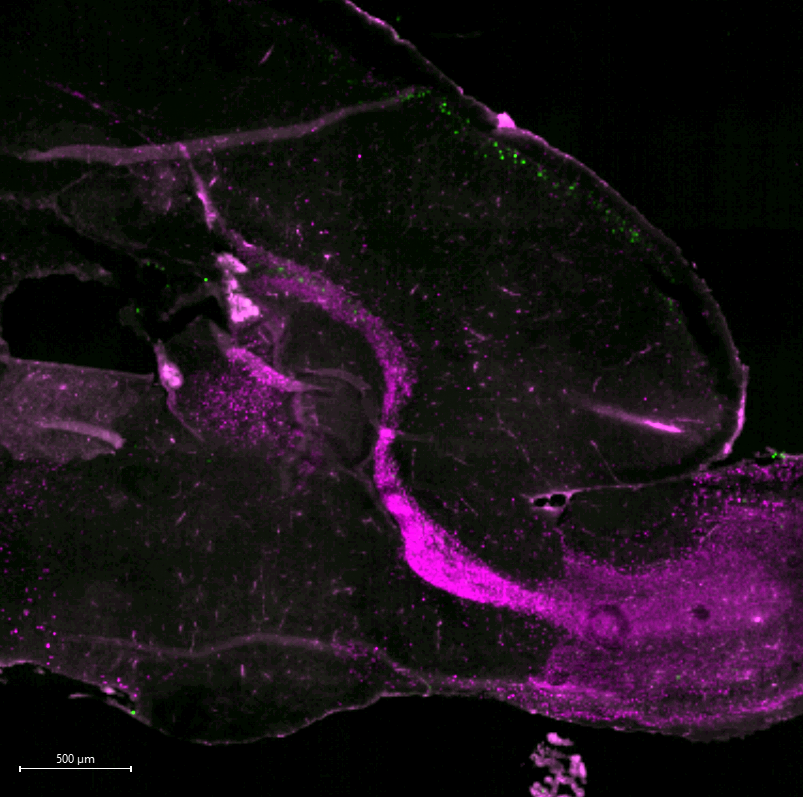

Supplement: Supplementary file 10 — Source data Fig. 8 [file 44318_2024_325_MOESM10_ESM.zip › 8D/8D_a.png]

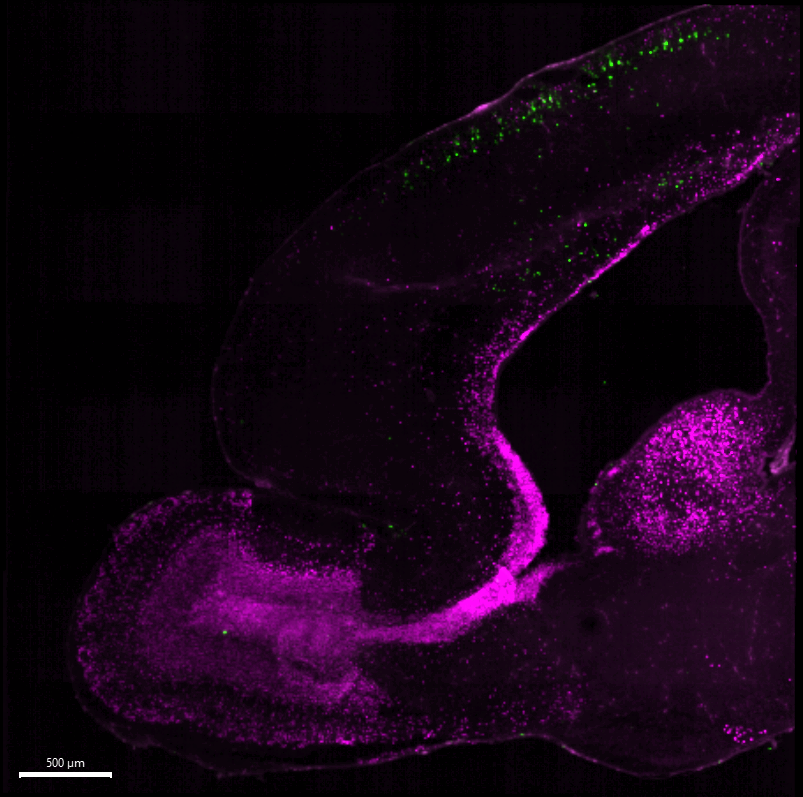

Supplement: Supplementary file 10 — Source data Fig. 8 [file 44318_2024_325_MOESM10_ESM.zip › 8D/8D_b.png]

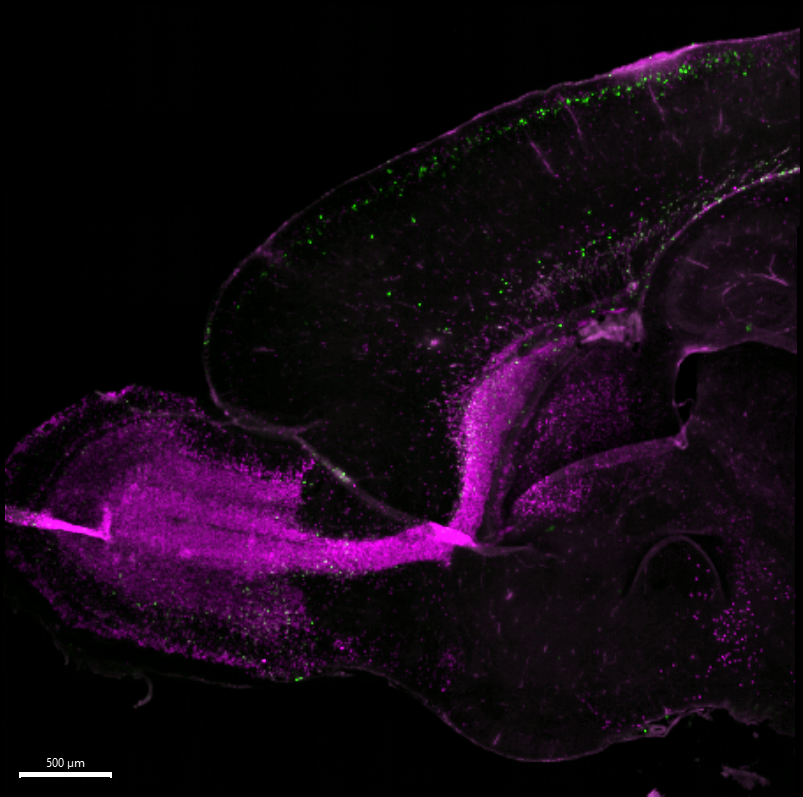

Supplement: Supplementary file 10 — Source data Fig. 8 [file 44318_2024_325_MOESM10_ESM.zip › 8D/8D_c.png]

## Slide 1
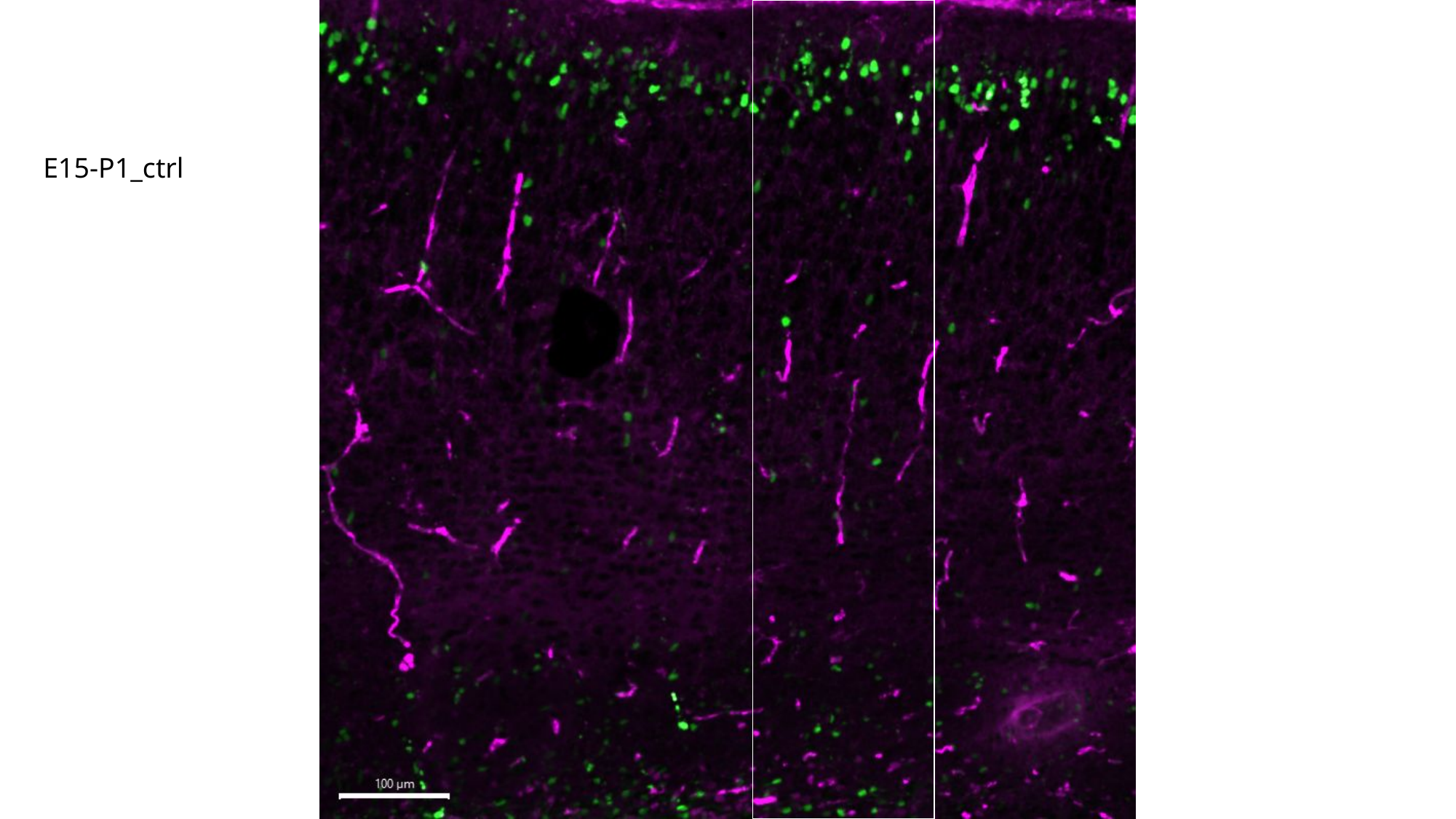

E15-P1_ctrl

## Slide 2
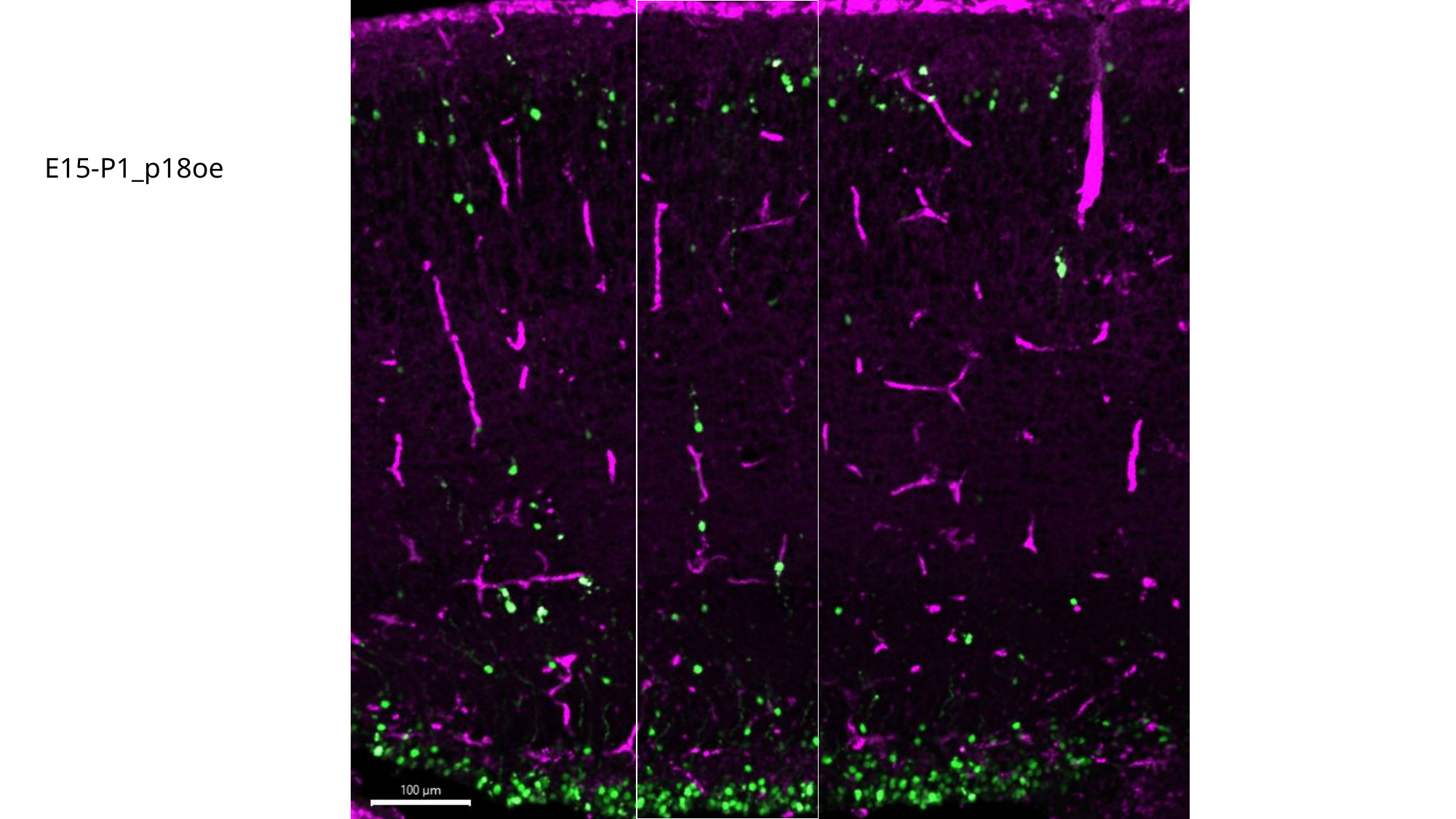

E15-P1_p18oe

## Slide 3
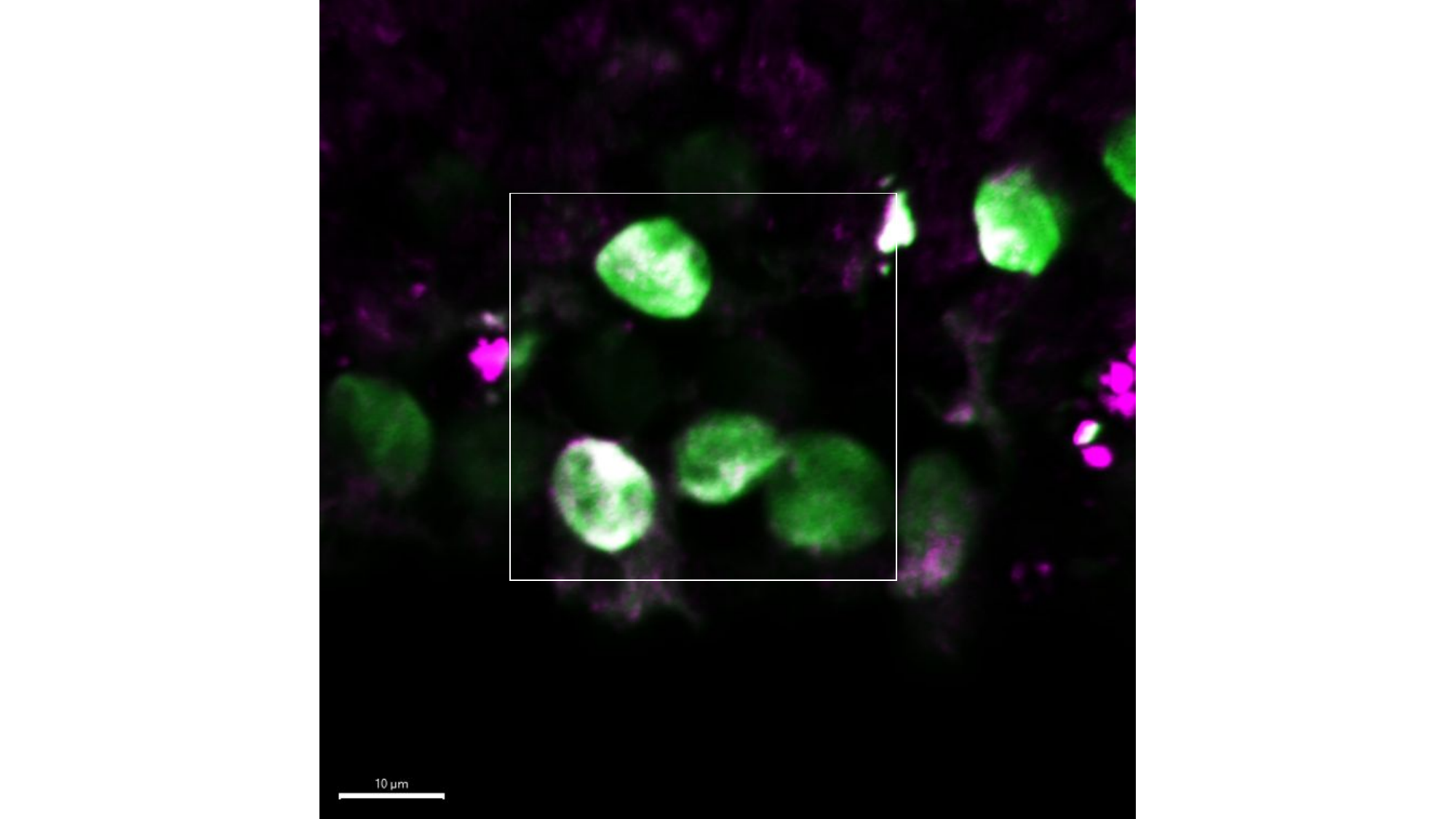

Supplement: Supplementary file 10 — Source data Fig. 8 [file 44318_2024_325_MOESM10_ESM.zip › 8C.pptx]

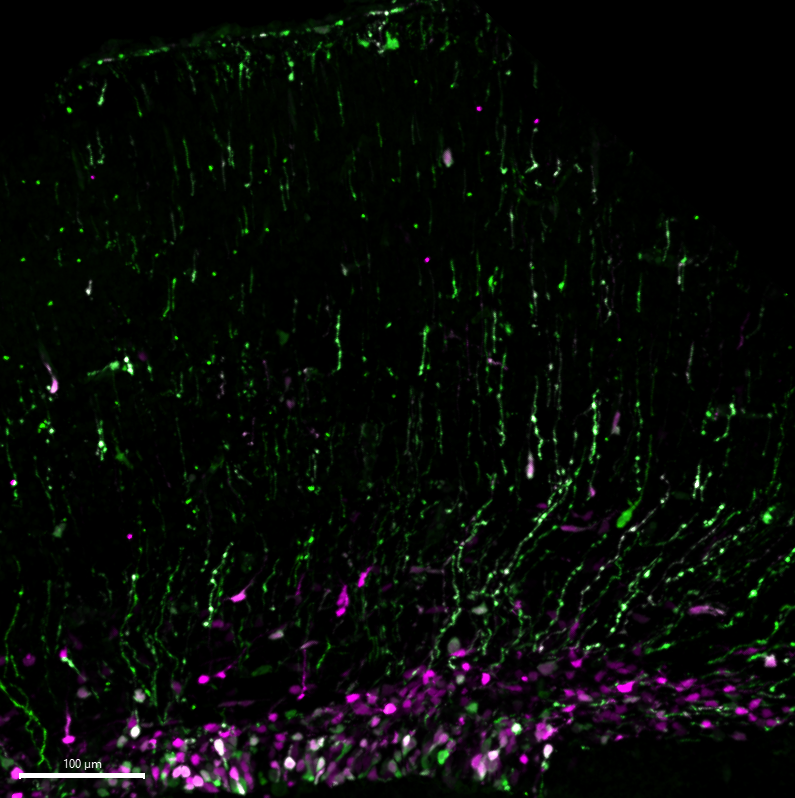

Supplement: Supplementary file 11 — Source data Fig. 9 [file 44318_2024_325_MOESM11_ESM.zip › 9A/9A_a-1.png]

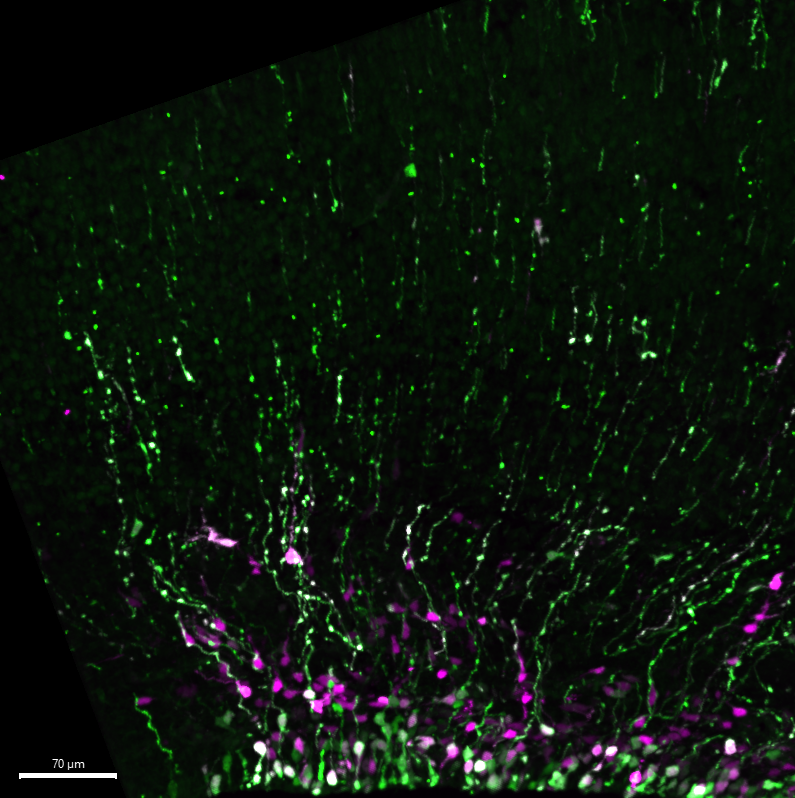

Supplement: Supplementary file 11 — Source data Fig. 9 [file 44318_2024_325_MOESM11_ESM.zip › 9A/9A_a-2.png]

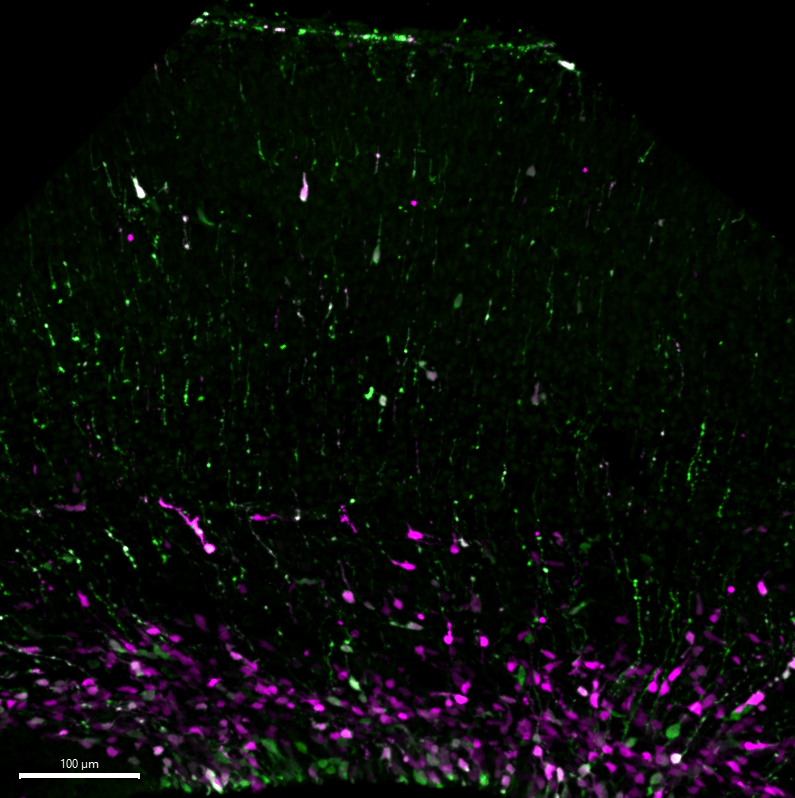

Supplement: Supplementary file 11 — Source data Fig. 9 [file 44318_2024_325_MOESM11_ESM.zip › 9A/9A_a-3.png]

## Slide 1
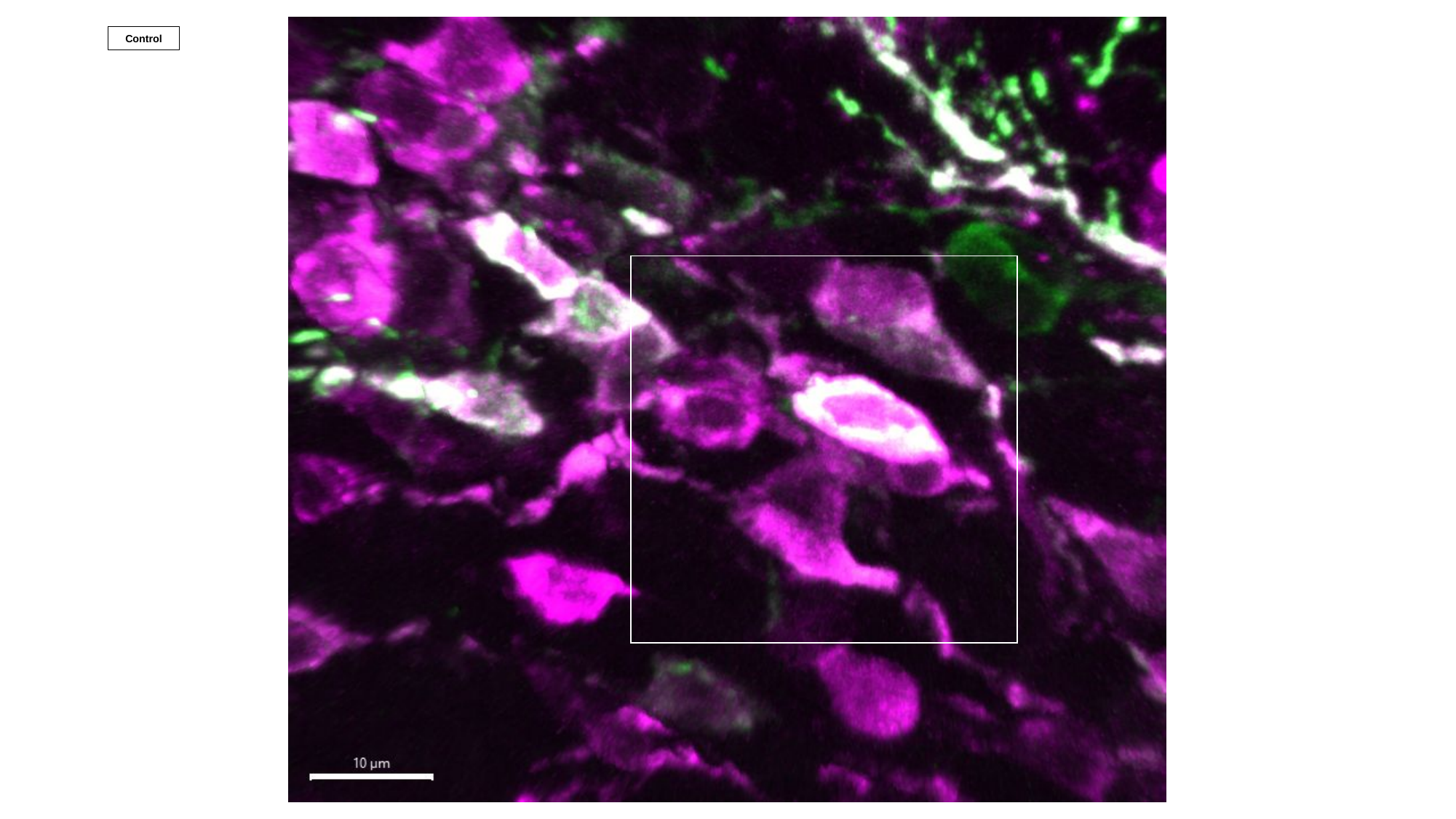

Control

## Slide 2
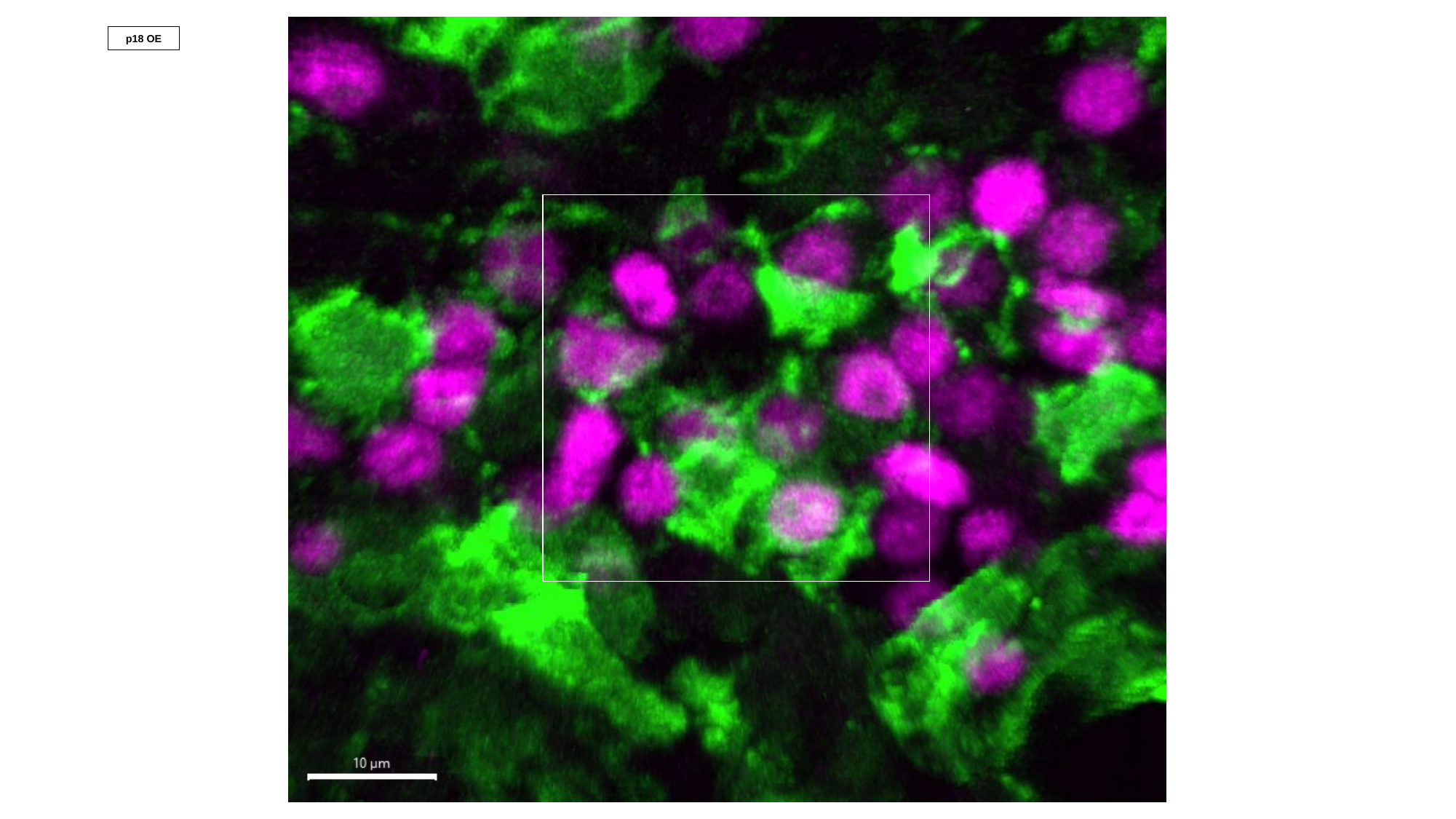

p18 OE

## Slide 3
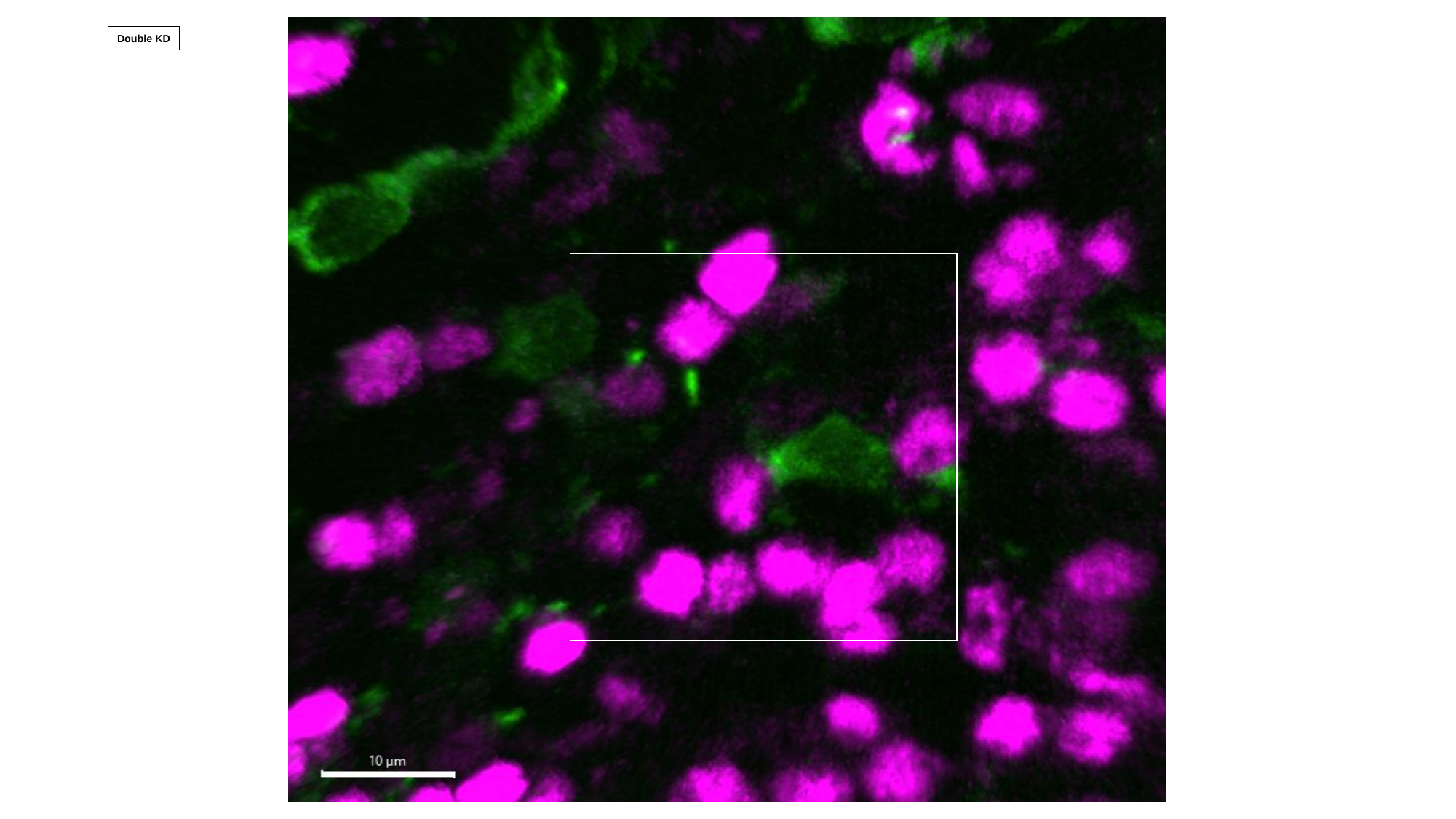

Double KD

Supplement: Supplementary file 11 — Source data Fig. 9 [file 44318_2024_325_MOESM11_ESM.zip › 9D/9D_c.pptx]
