# Supplementary material for: Cyclin-dependent kinase inhibitor p18 regulates lineage transitions of excitatory neurons, astrocytes, and interneurons in the mouse cortex
Source: EMBO J. 2024 Dec 12;44(2):382–412. doi: 10.1038/s44318-024-00325-9 (PMC11730326; doi:10.1038/s44318-024-00325-9)
Supplement: Supplementary file 4 — Source data Fig. 2 [file 44318_2024_325_MOESM4_ESM.zip › 2A.pptx]

## Slide 1
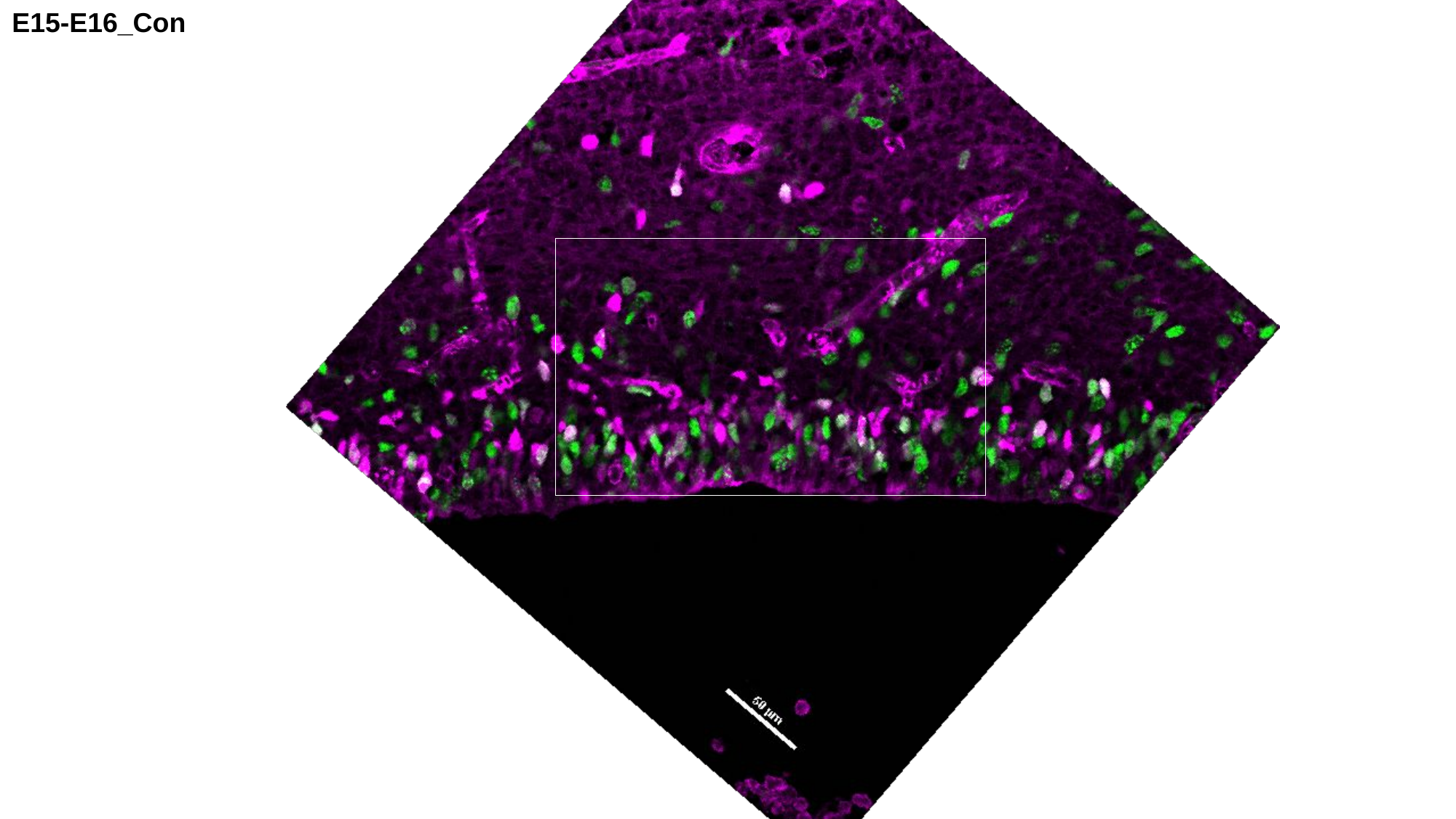

E15-E16_Con

## Slide 2
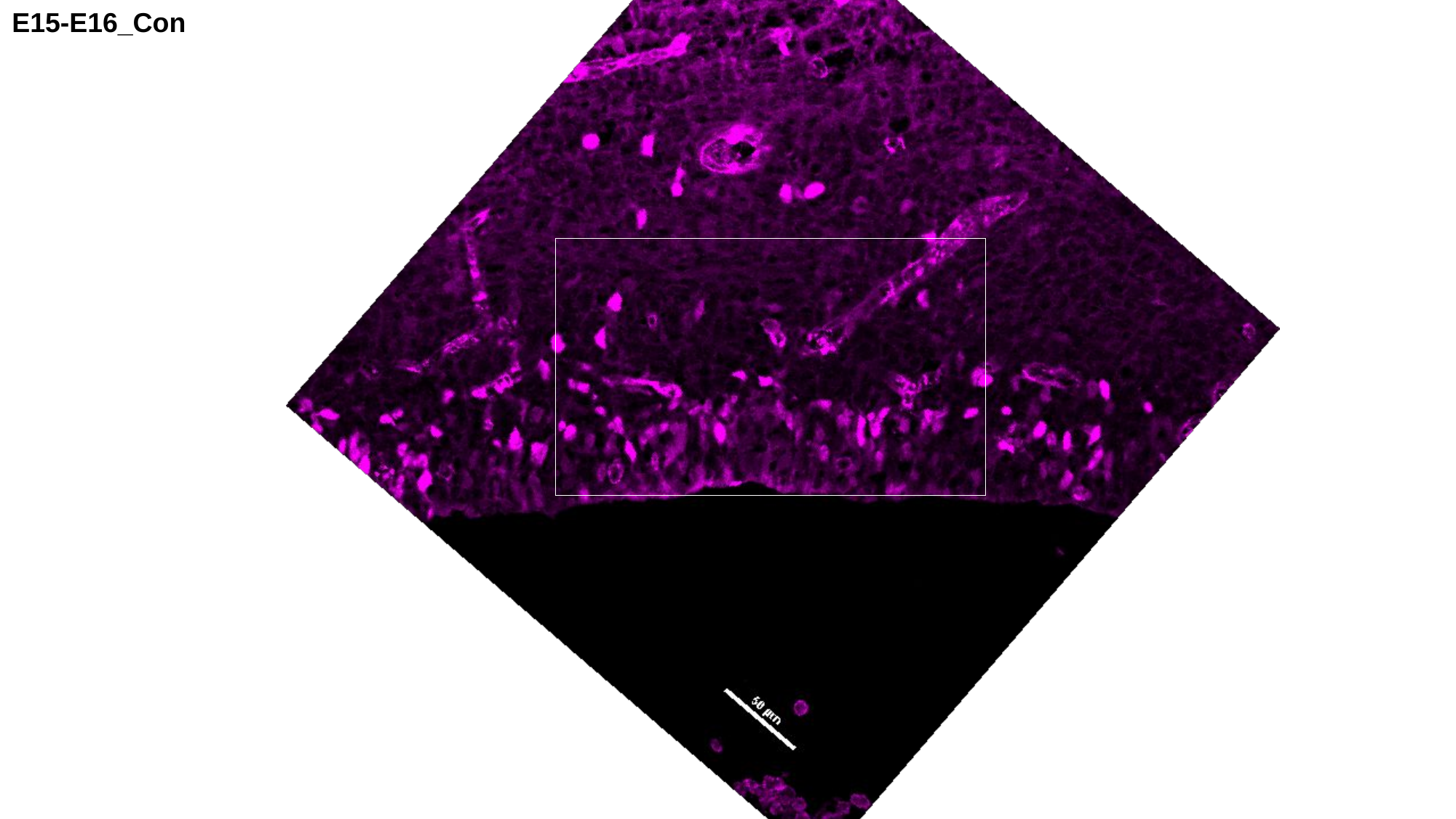

E15-E16_Con

## Slide 3
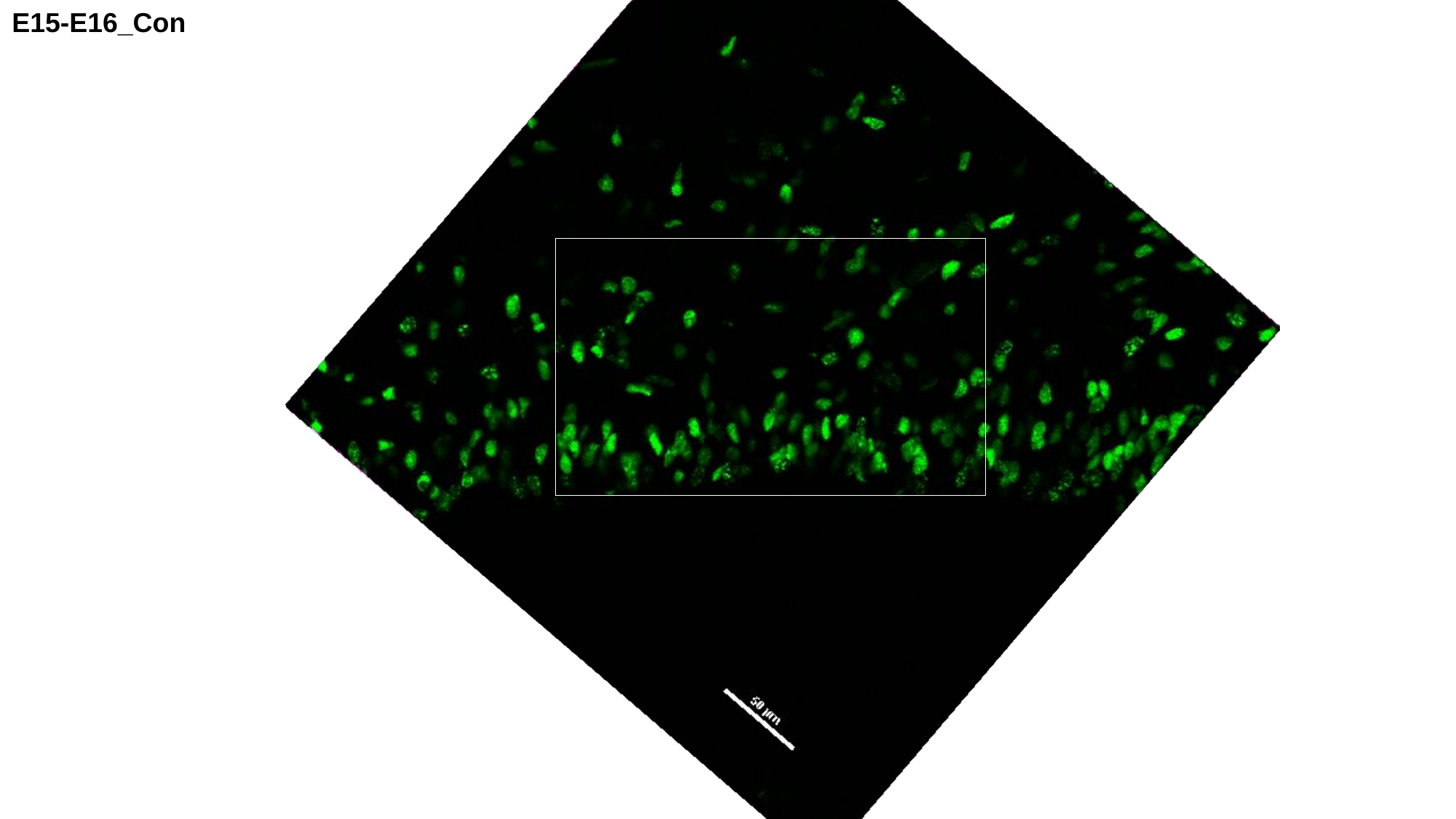

E15-E16_Con

## Slide 4
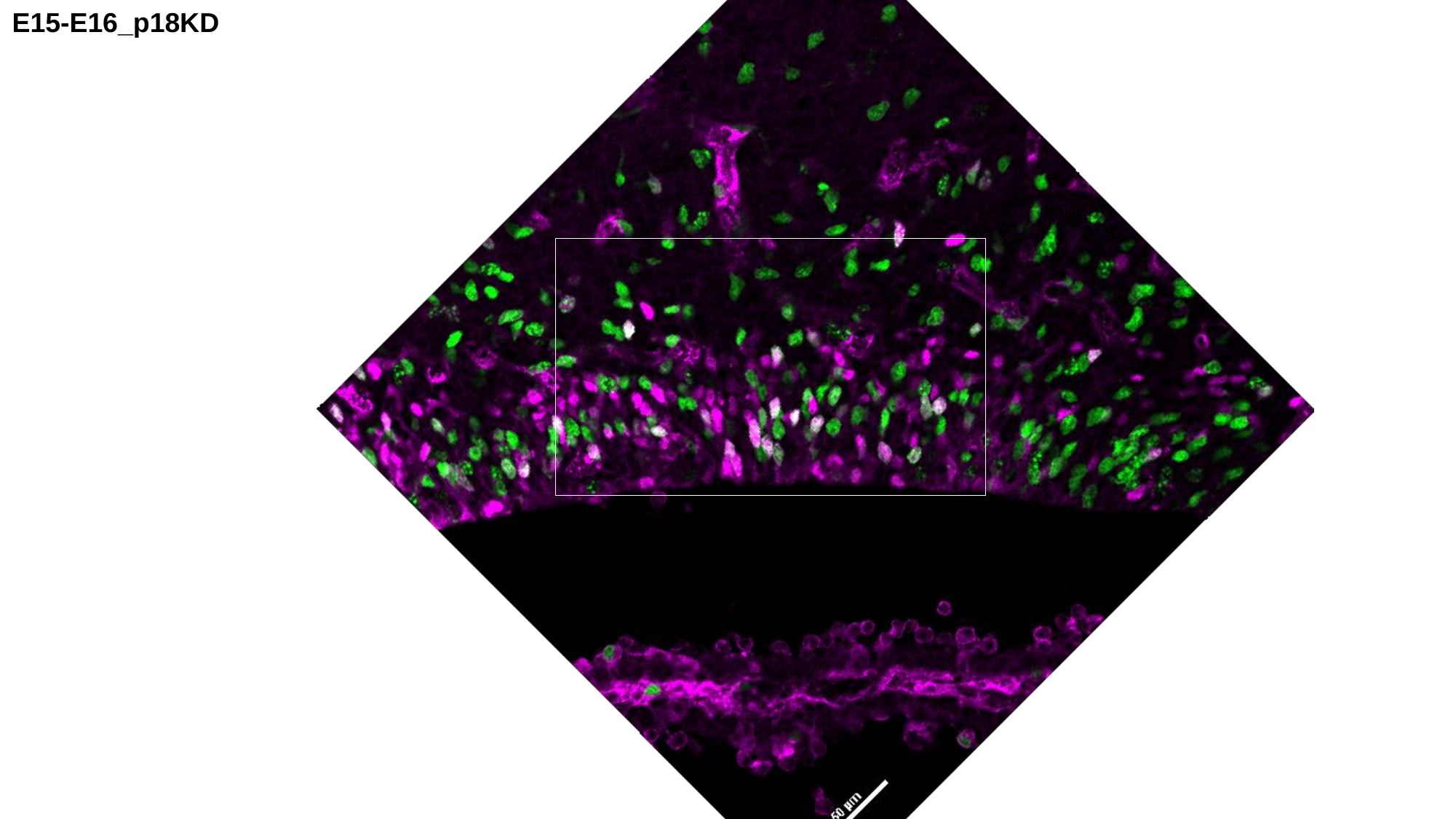

E15-E16_p18KD

## Slide 5
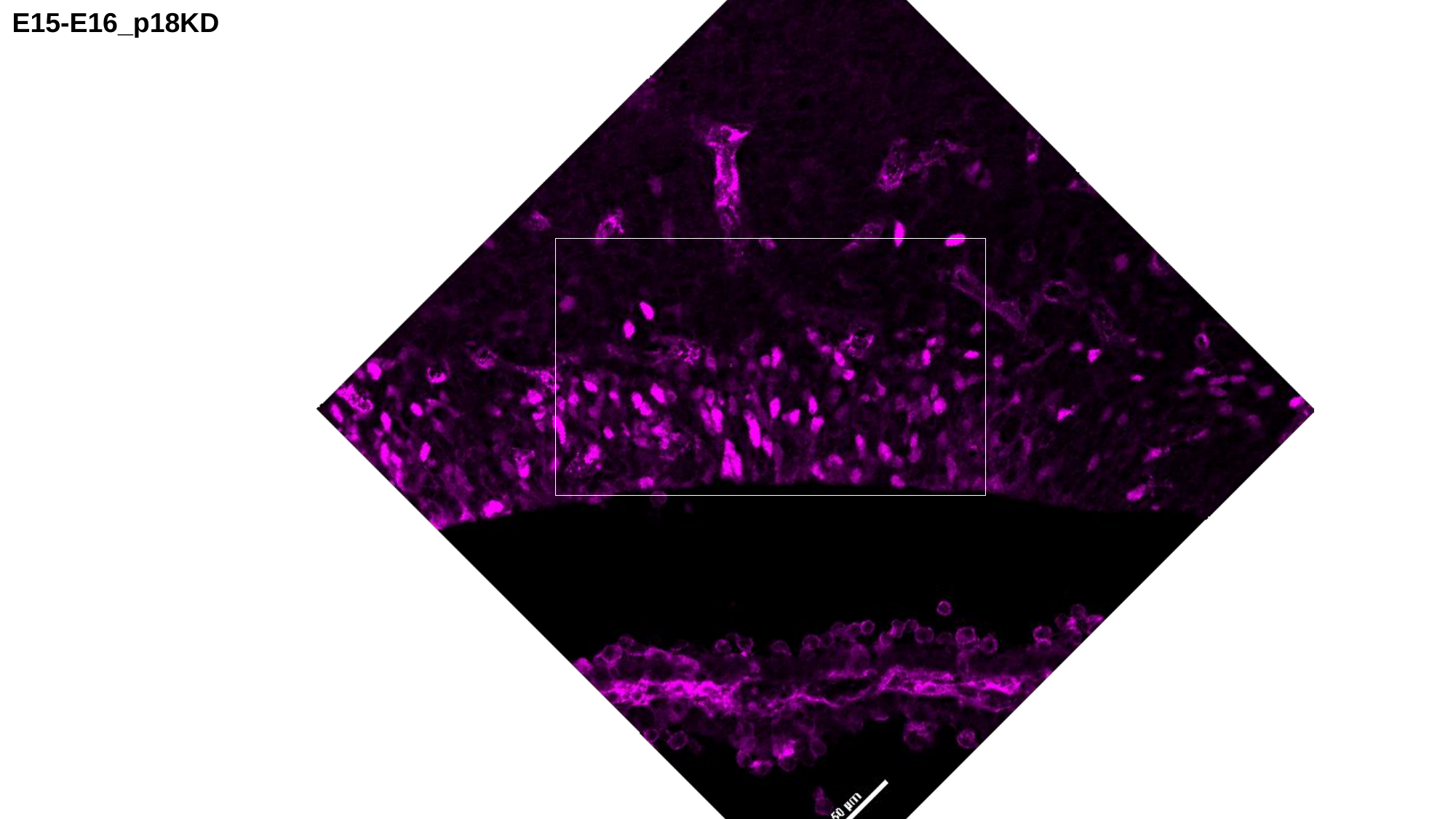

E15-E16_p18KD

## Slide 6
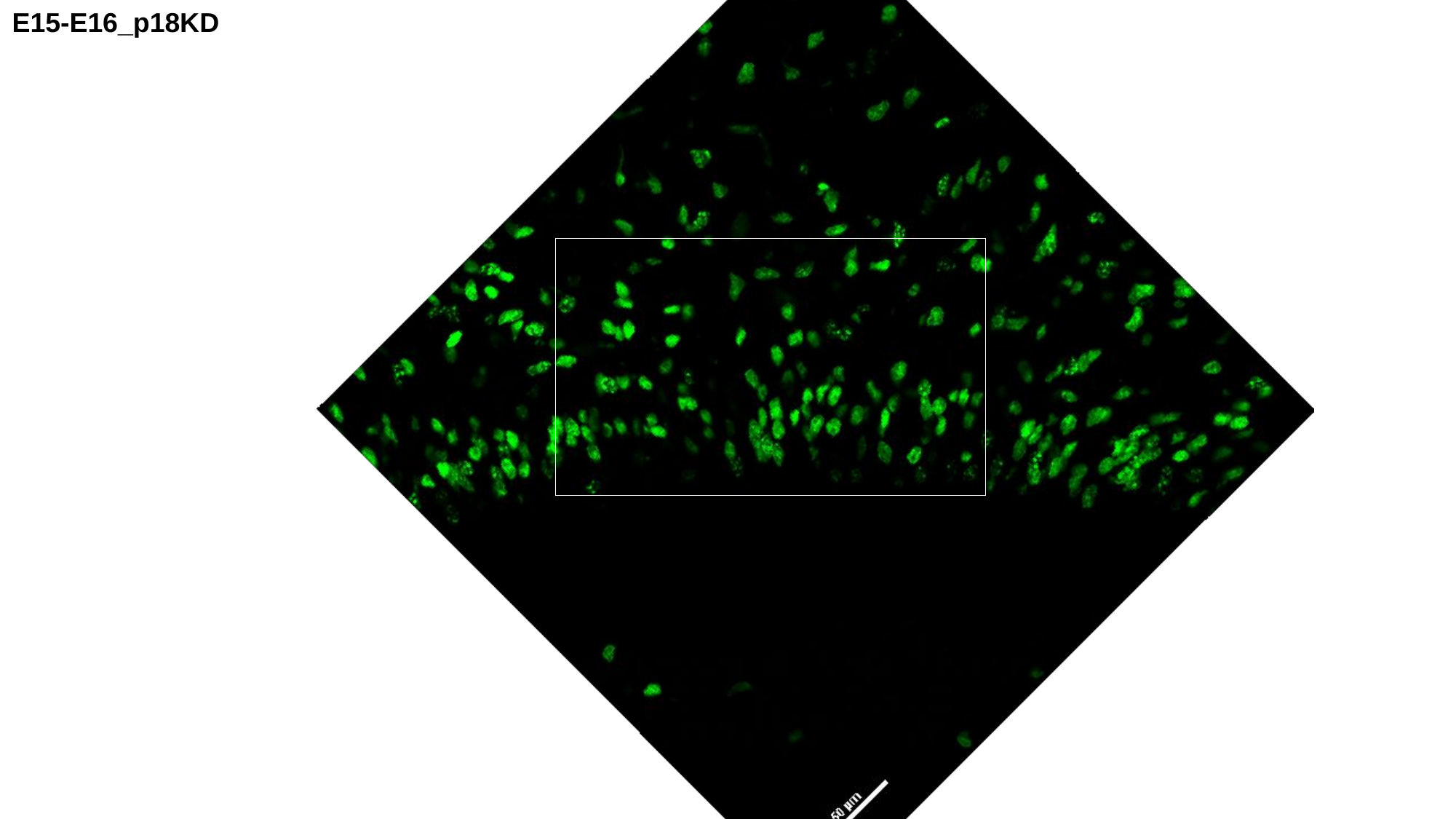

E15-E16_p18KD

## Slide 7
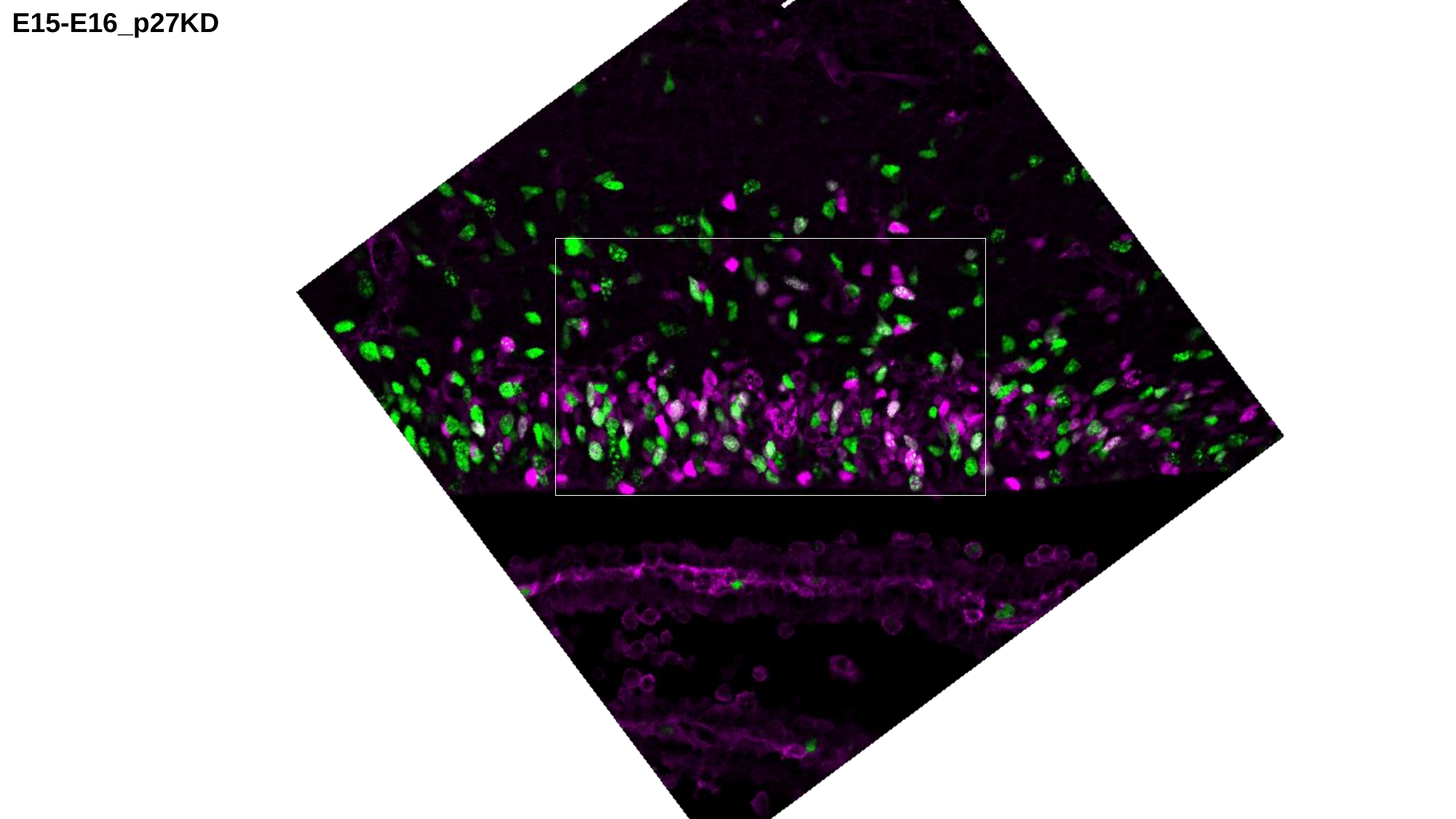

E15-E16_p27KD

## Slide 8
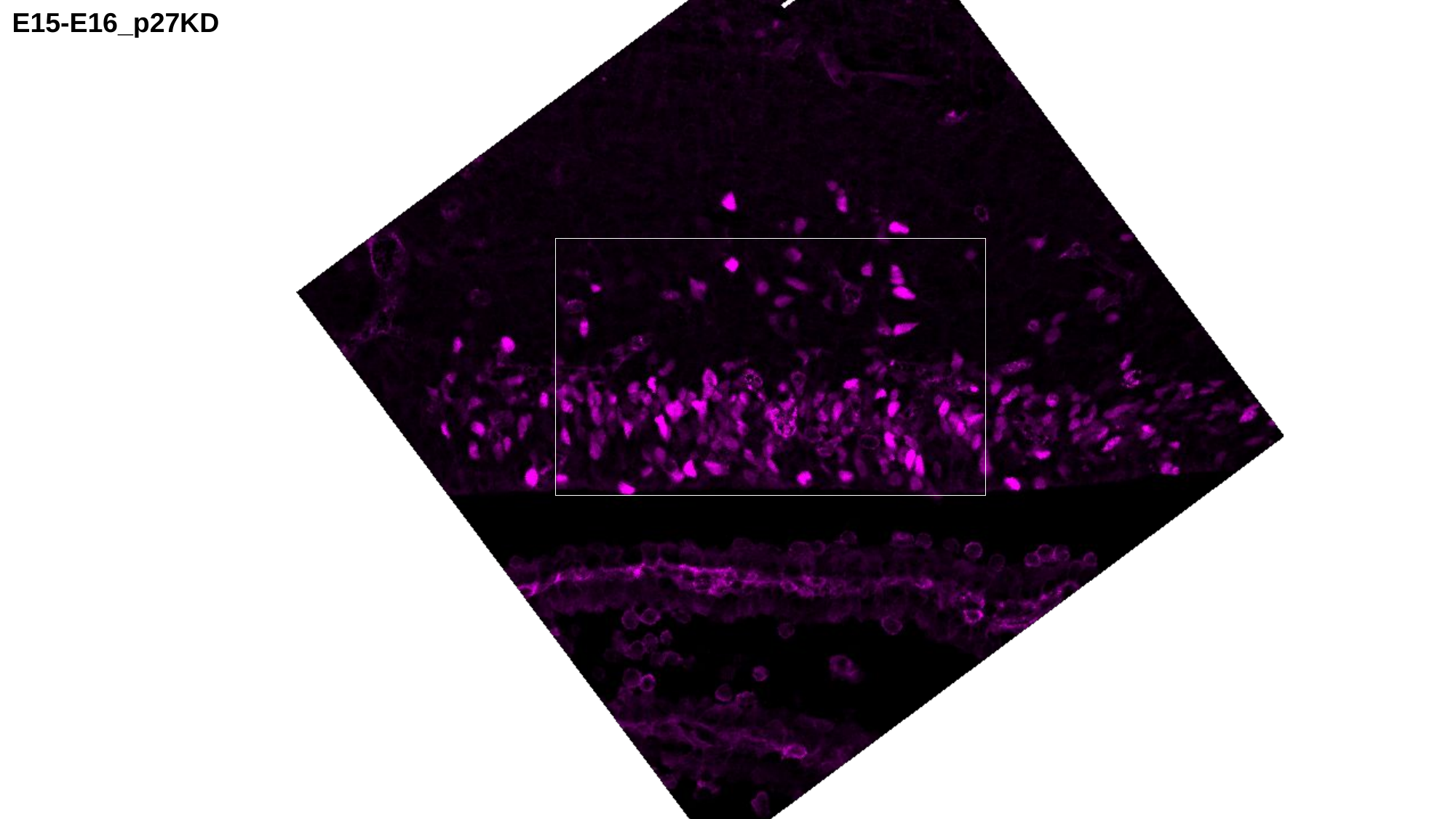

E15-E16_p27KD

## Slide 9
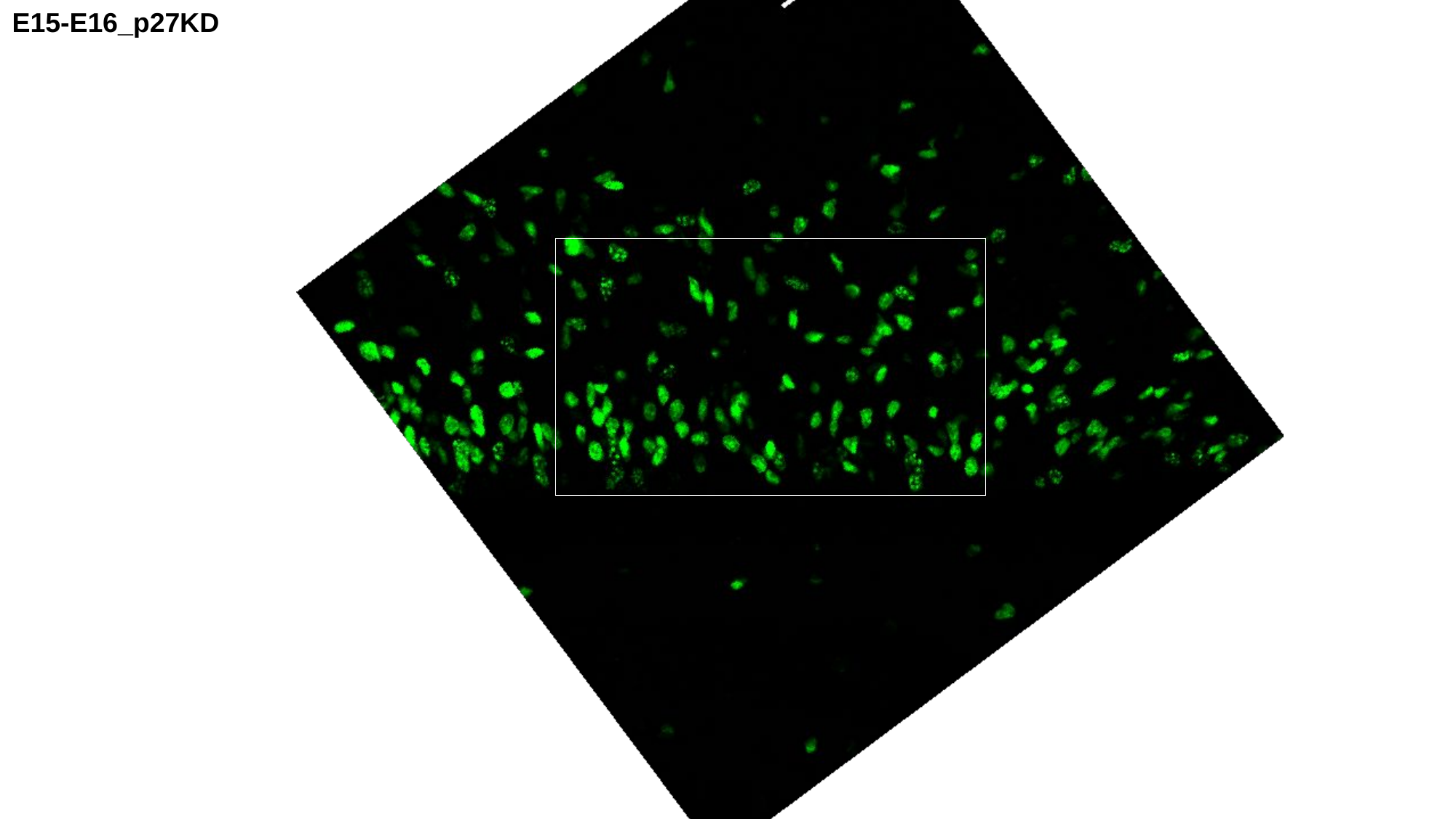

E15-E16_p27KD

## Slide 10
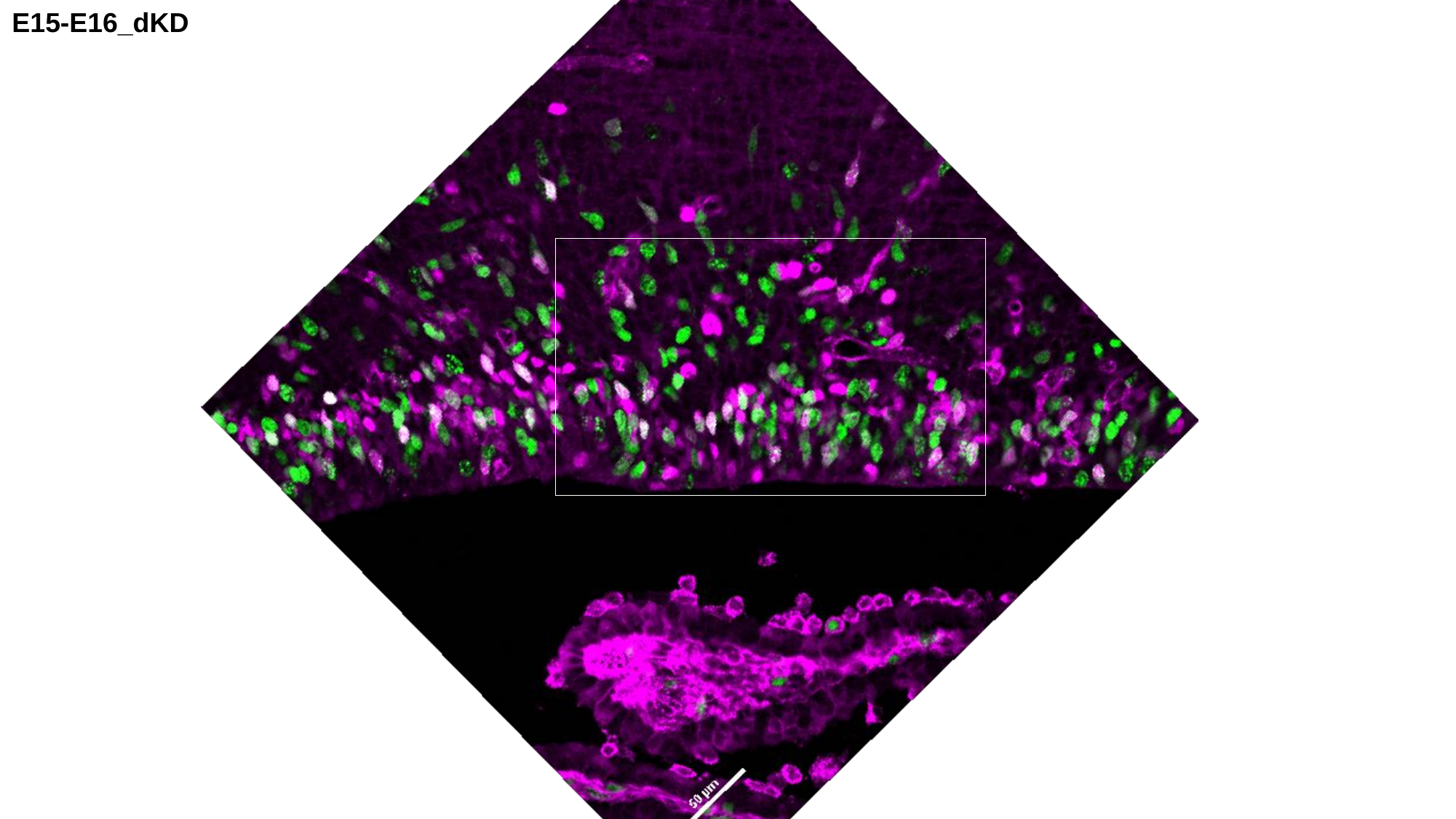

E15-E16_dKD

## Slide 11
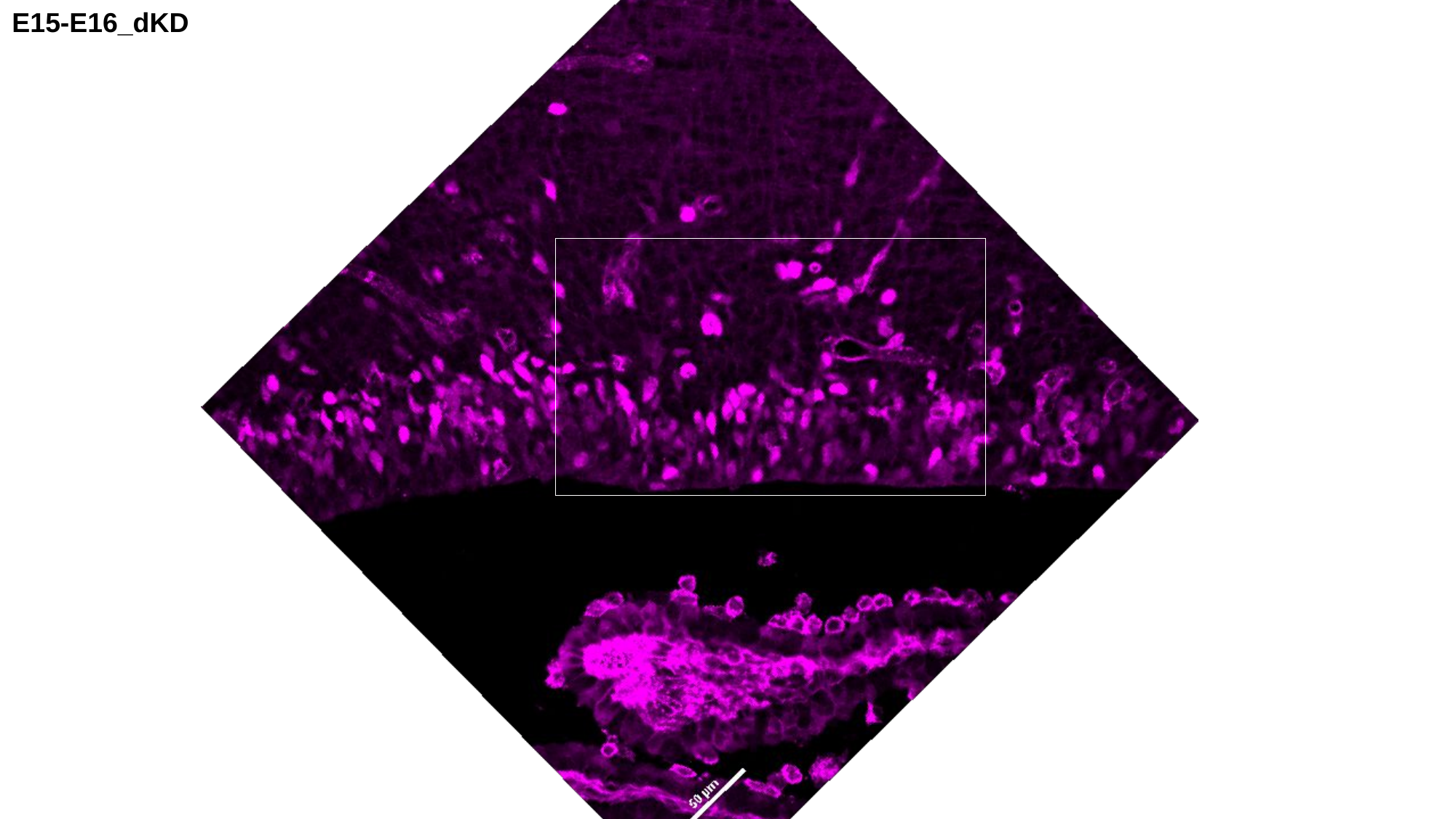

E15-E16_dKD

## Slide 12
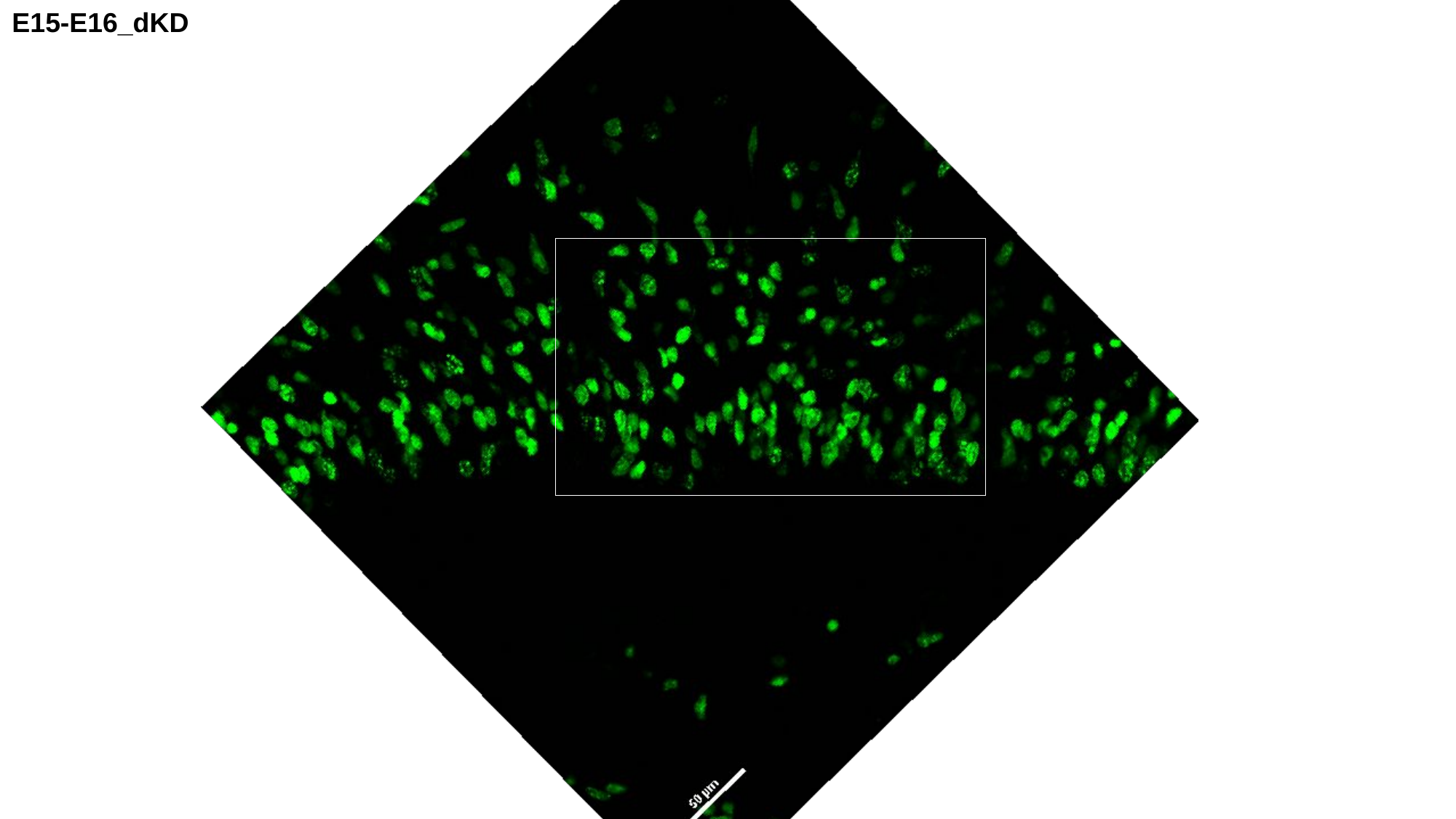

E15-E16_dKD

## Slide 13
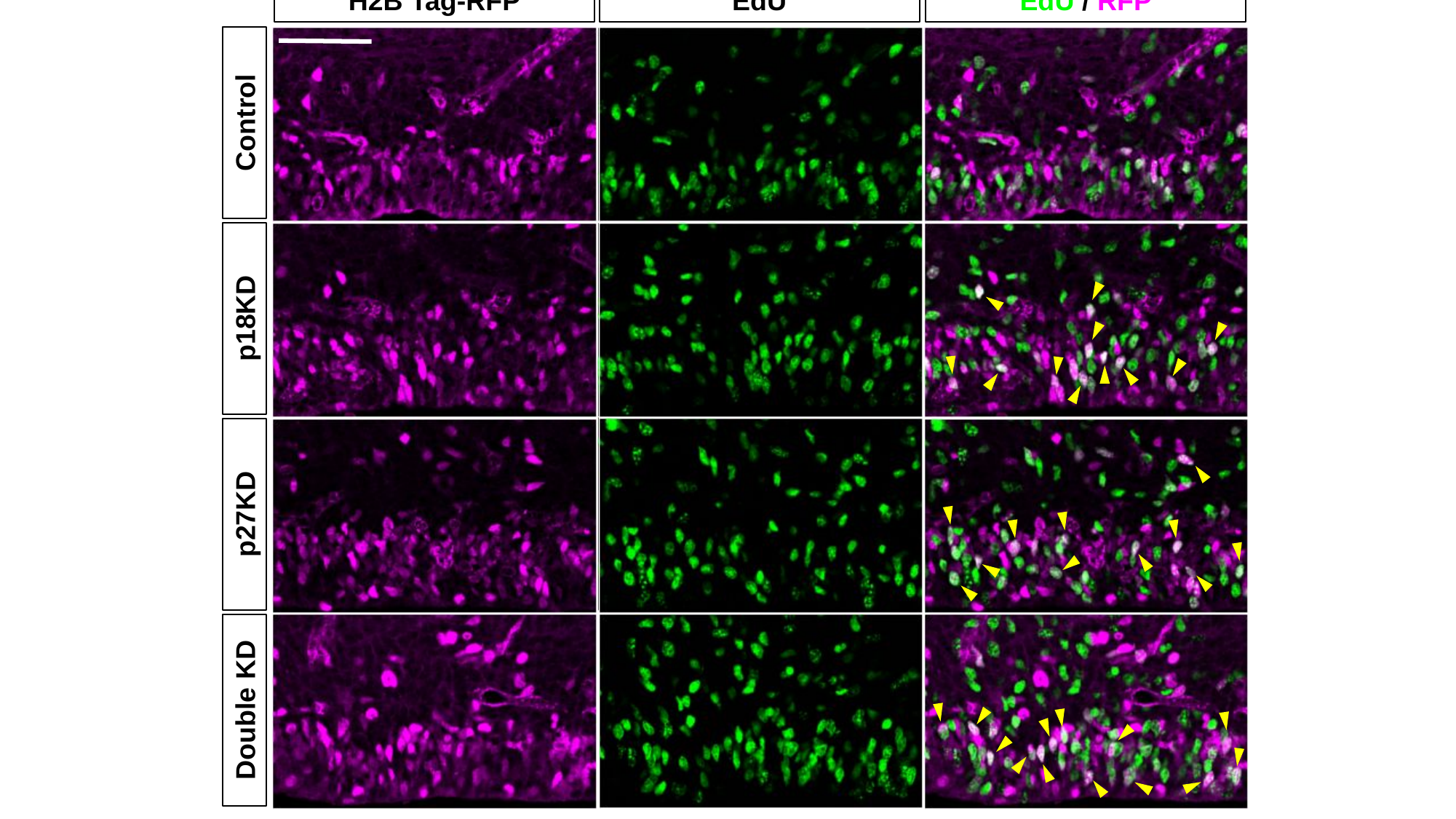

H2B Tag-RFP
EdU
EdU / RFP
Control
p18KD
p27KD
Double KD
