# Supplementary material for: Cyclin-dependent kinase inhibitor p18 regulates lineage transitions of excitatory neurons, astrocytes, and interneurons in the mouse cortex
Source: EMBO J. 2024 Dec 12;44(2):382–412. doi: 10.1038/s44318-024-00325-9 (PMC11730326; doi:10.1038/s44318-024-00325-9)
Supplement: Supplementary file 4 — Source data Fig. 2 [file 44318_2024_325_MOESM4_ESM.zip › 2D.pptx]

## Slide 1
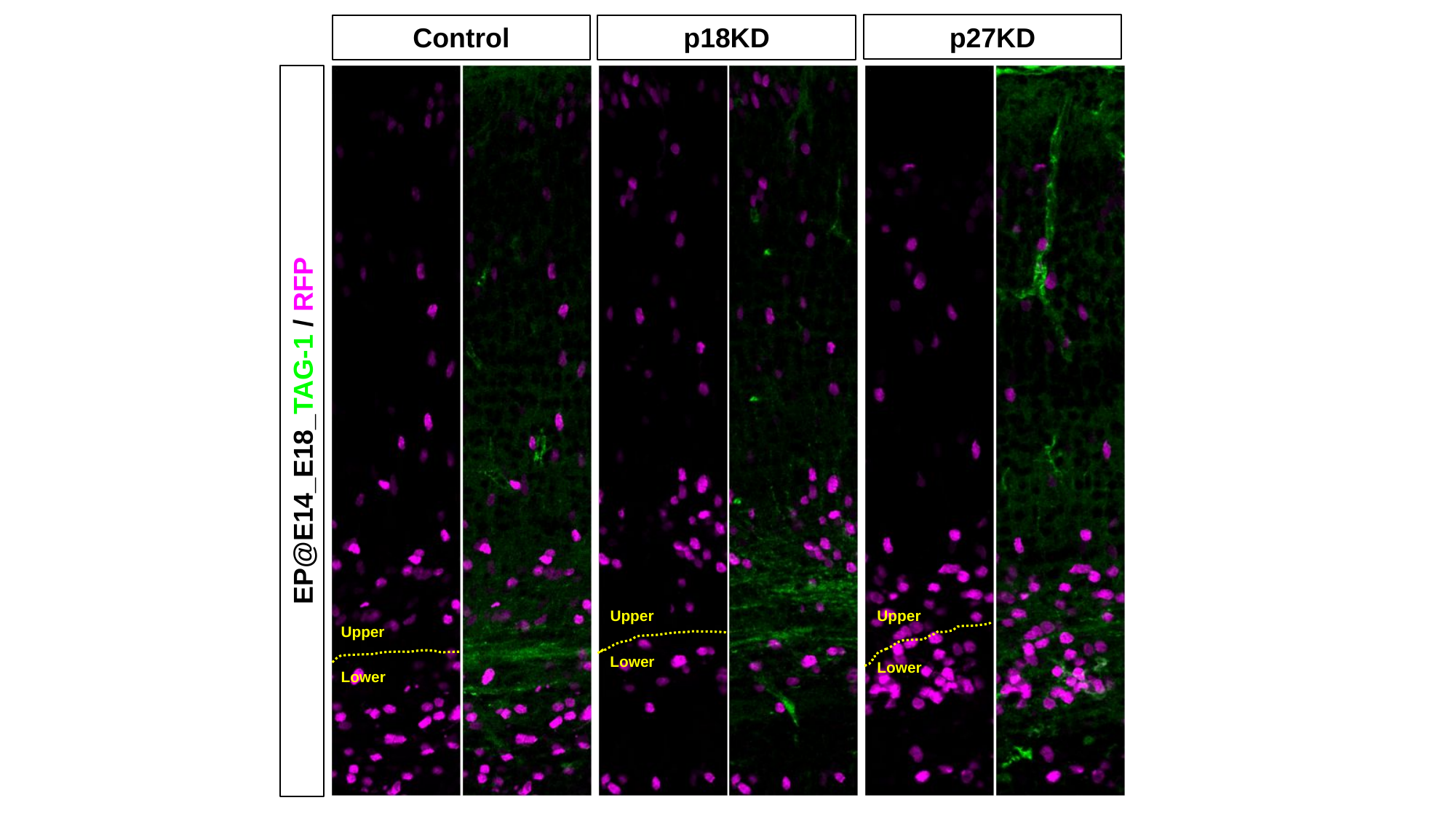

p27KD
Control
p18KD
EP@E14_E18_TAG-1 / RFP
Upper
Upper
Upper
Lower
Lower
Lower

## Slide 2
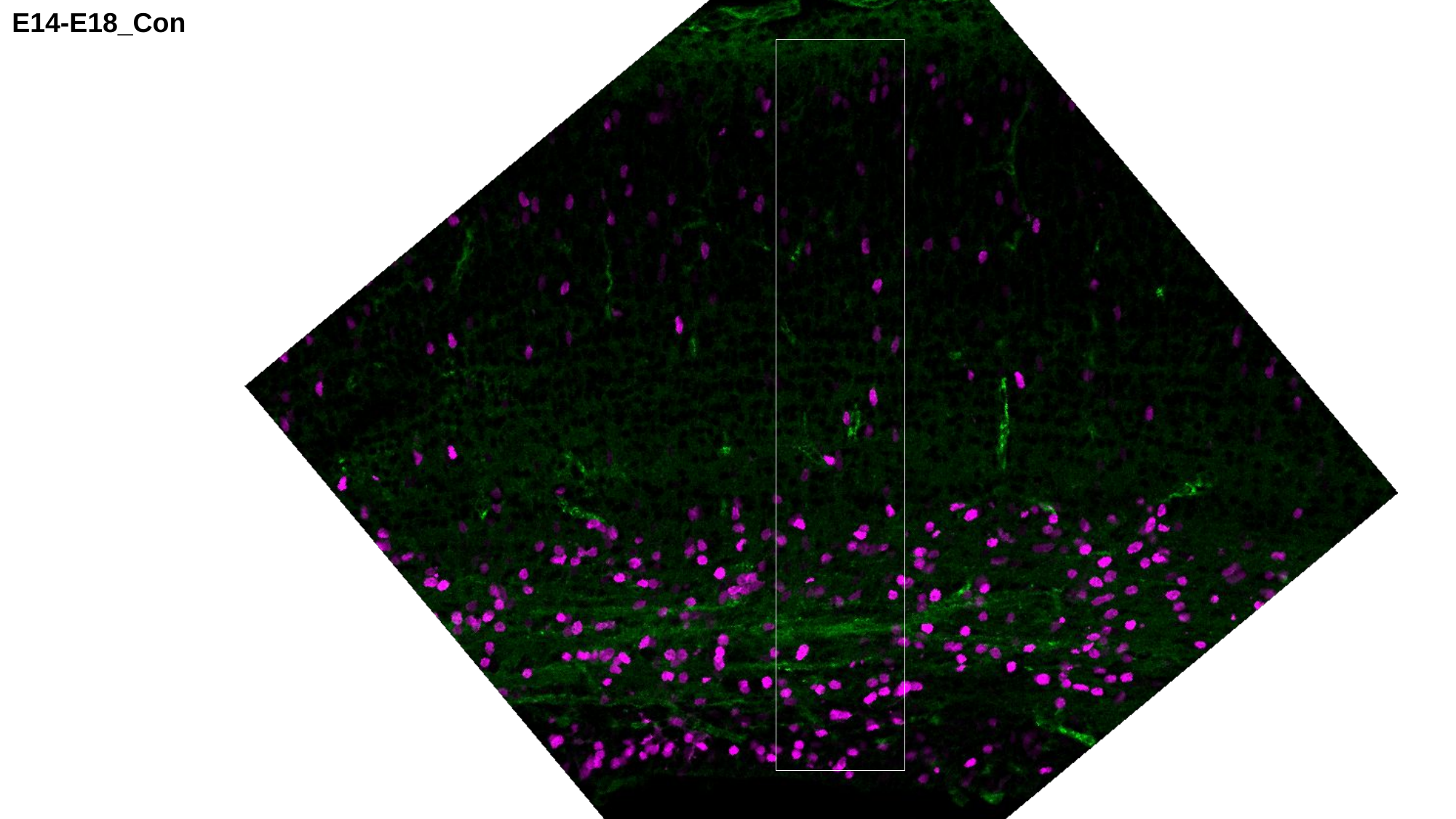

E14-E18_Con

## Slide 3
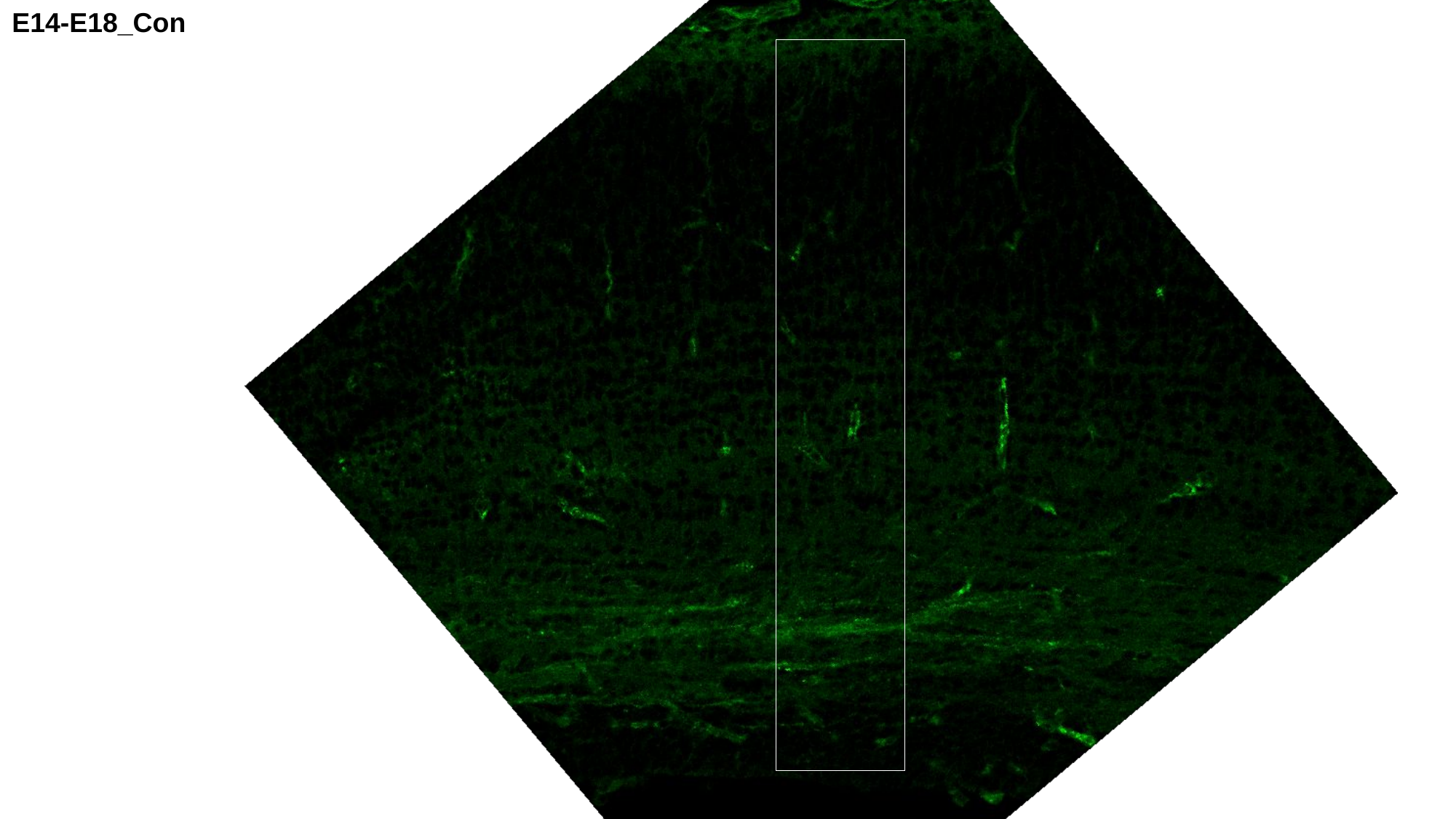

E14-E18_Con

## Slide 4
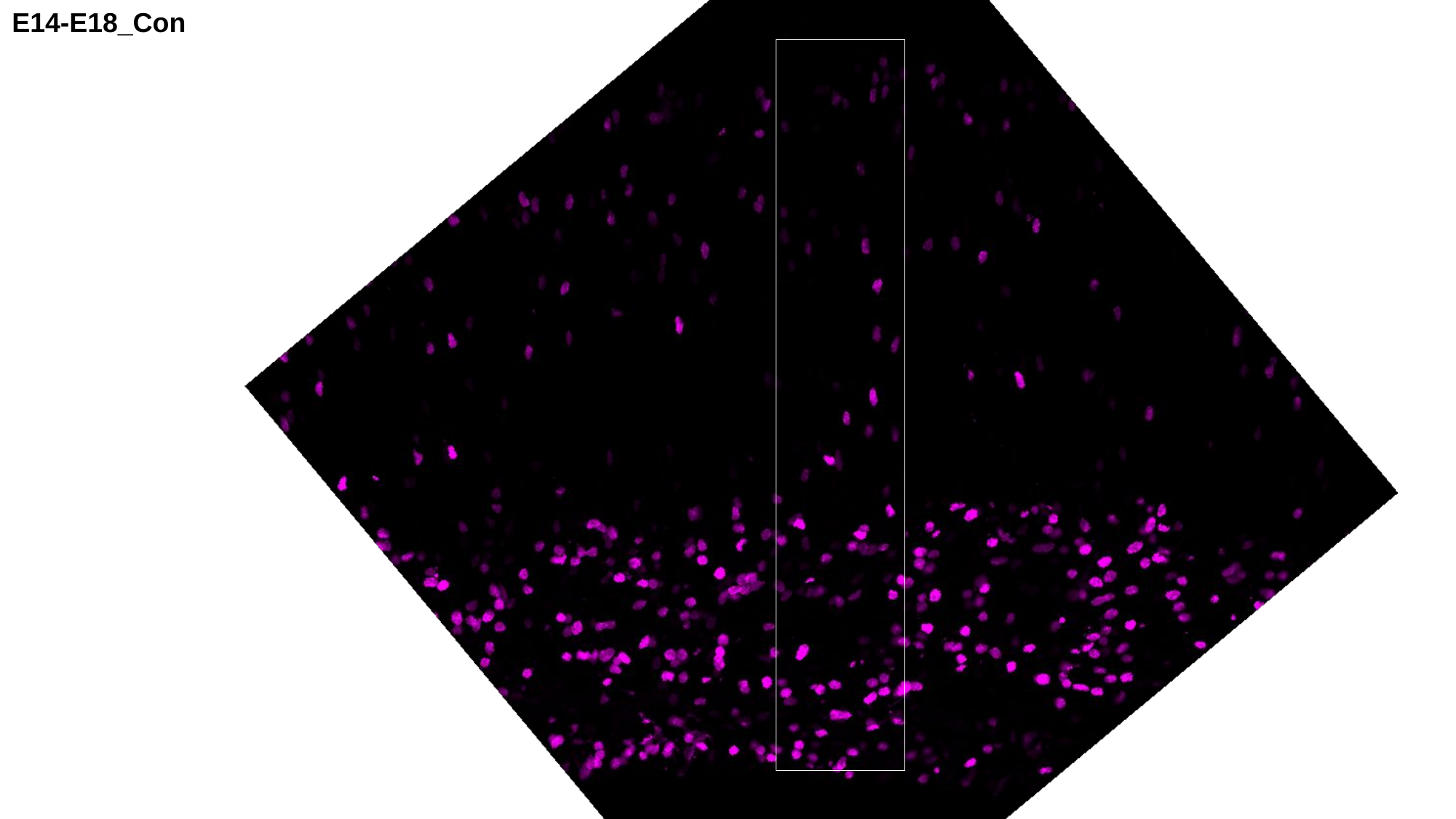

E14-E18_Con

## Slide 5
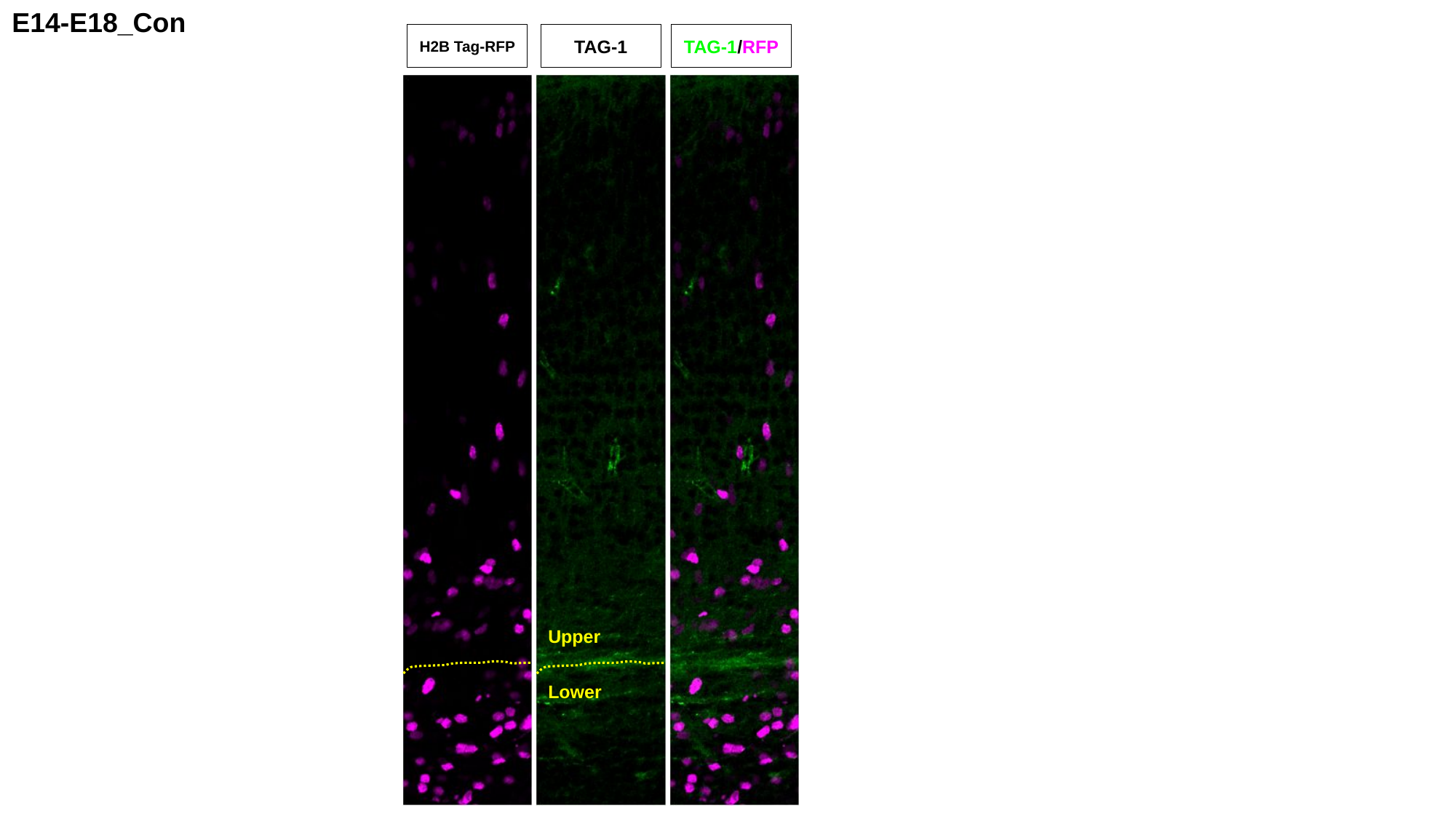

E14-E18_Con
H2B Tag-RFP
TAG-1
TAG-1/RFP
Upper
Lower

## Slide 6
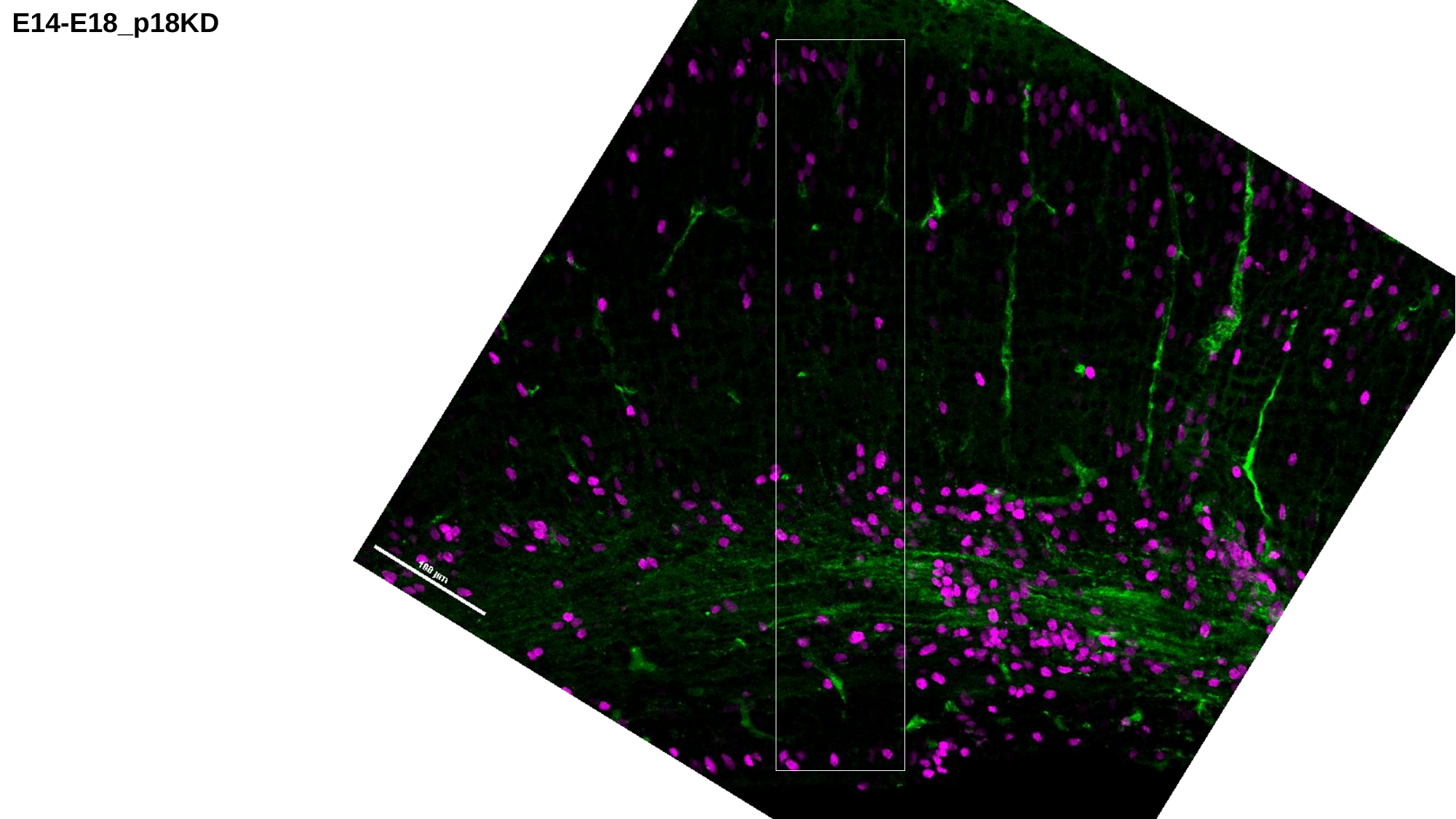

E14-E18_p18KD

## Slide 7
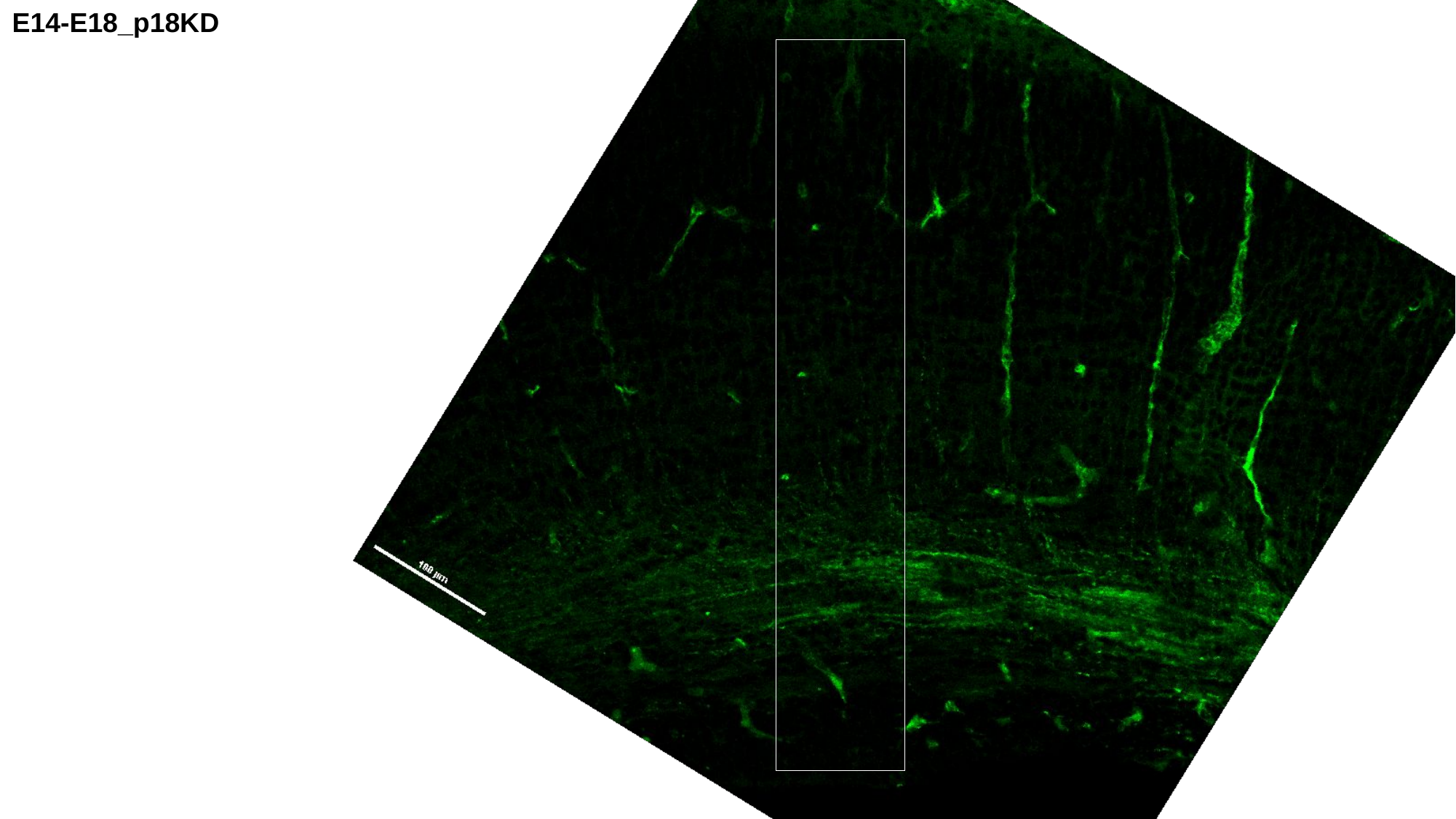

E14-E18_p18KD

## Slide 8
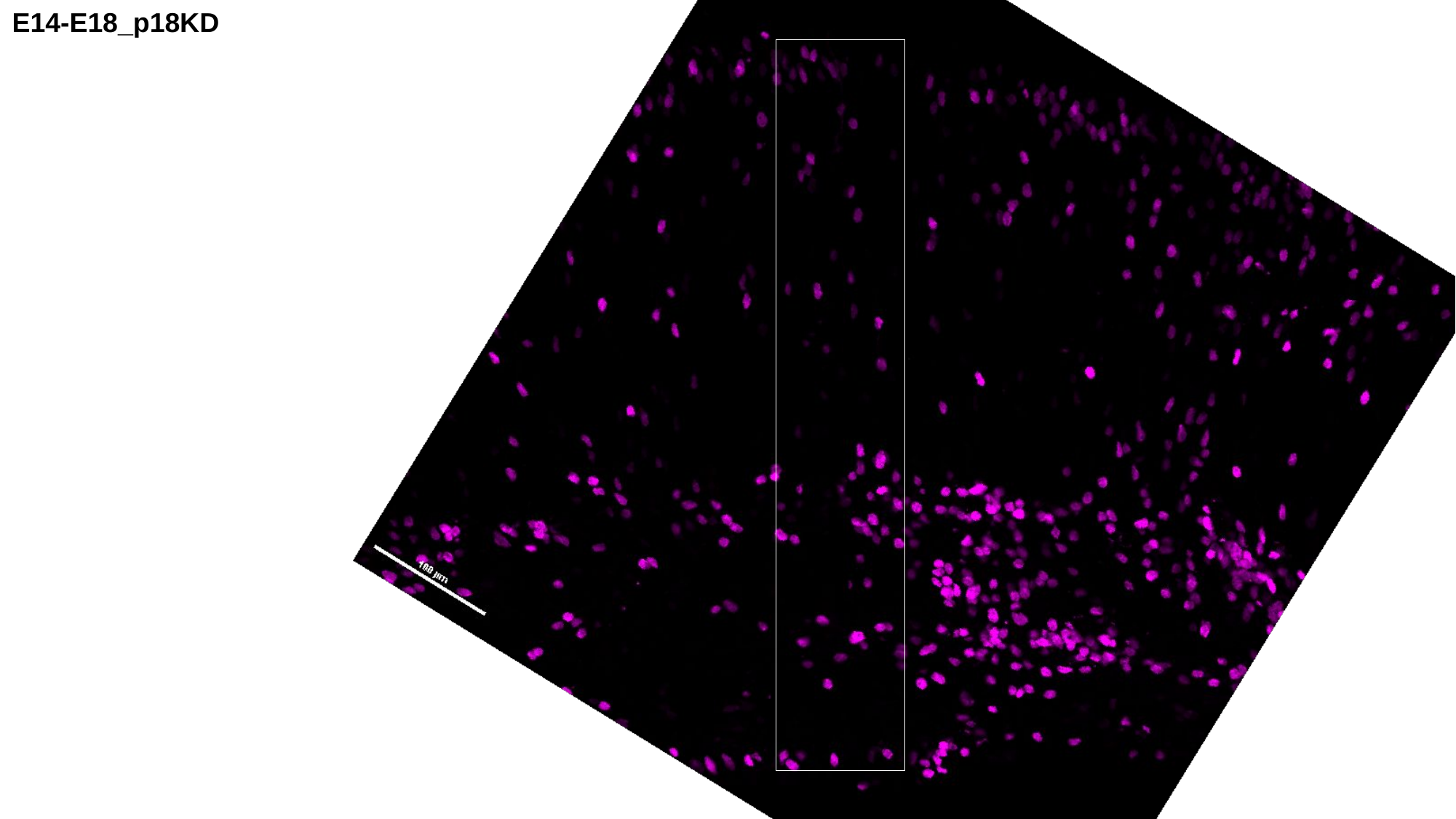

E14-E18_p18KD

## Slide 9
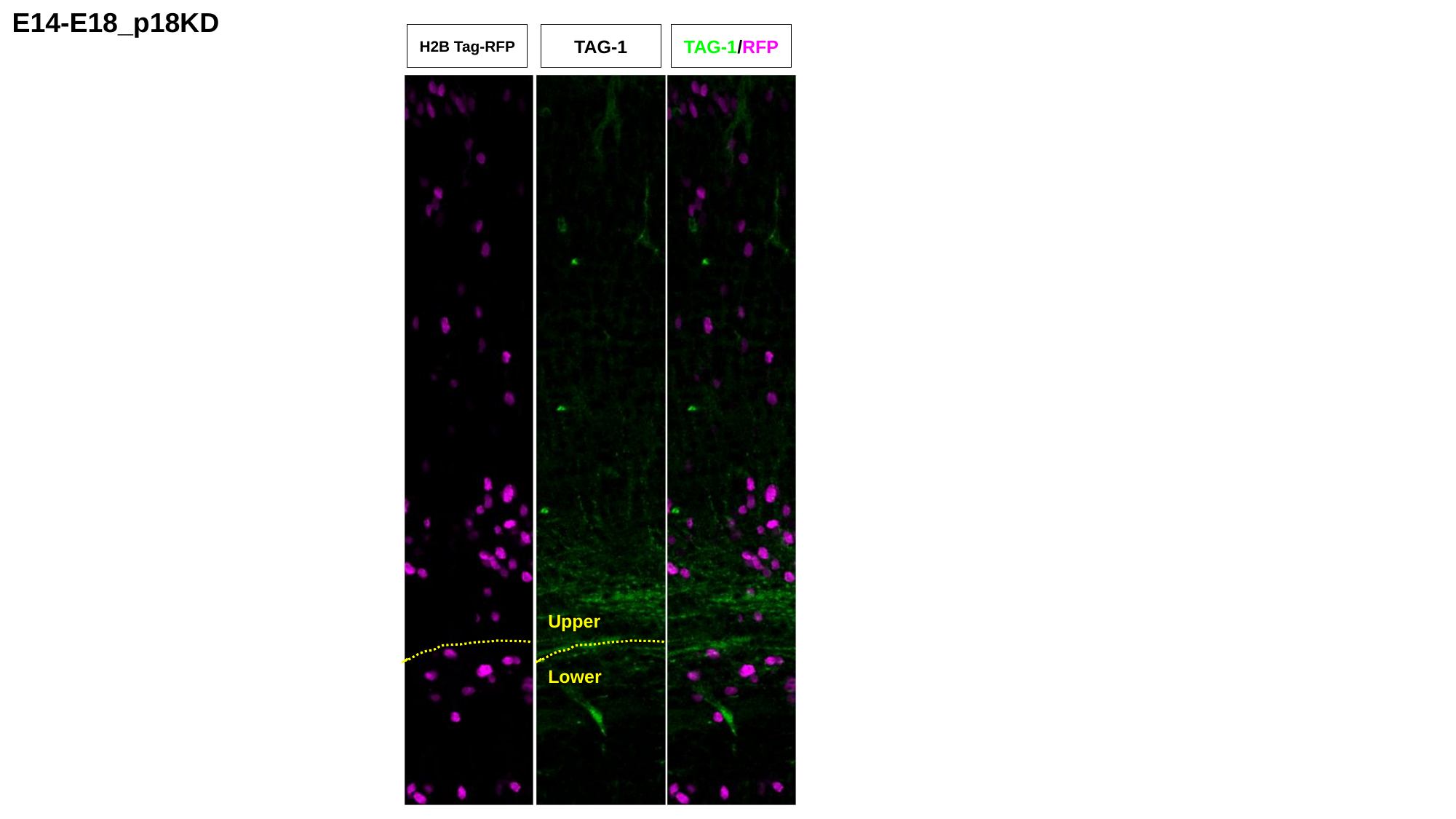

E14-E18_p18KD
H2B Tag-RFP
TAG-1
TAG-1/RFP
Upper
Lower

## Slide 10
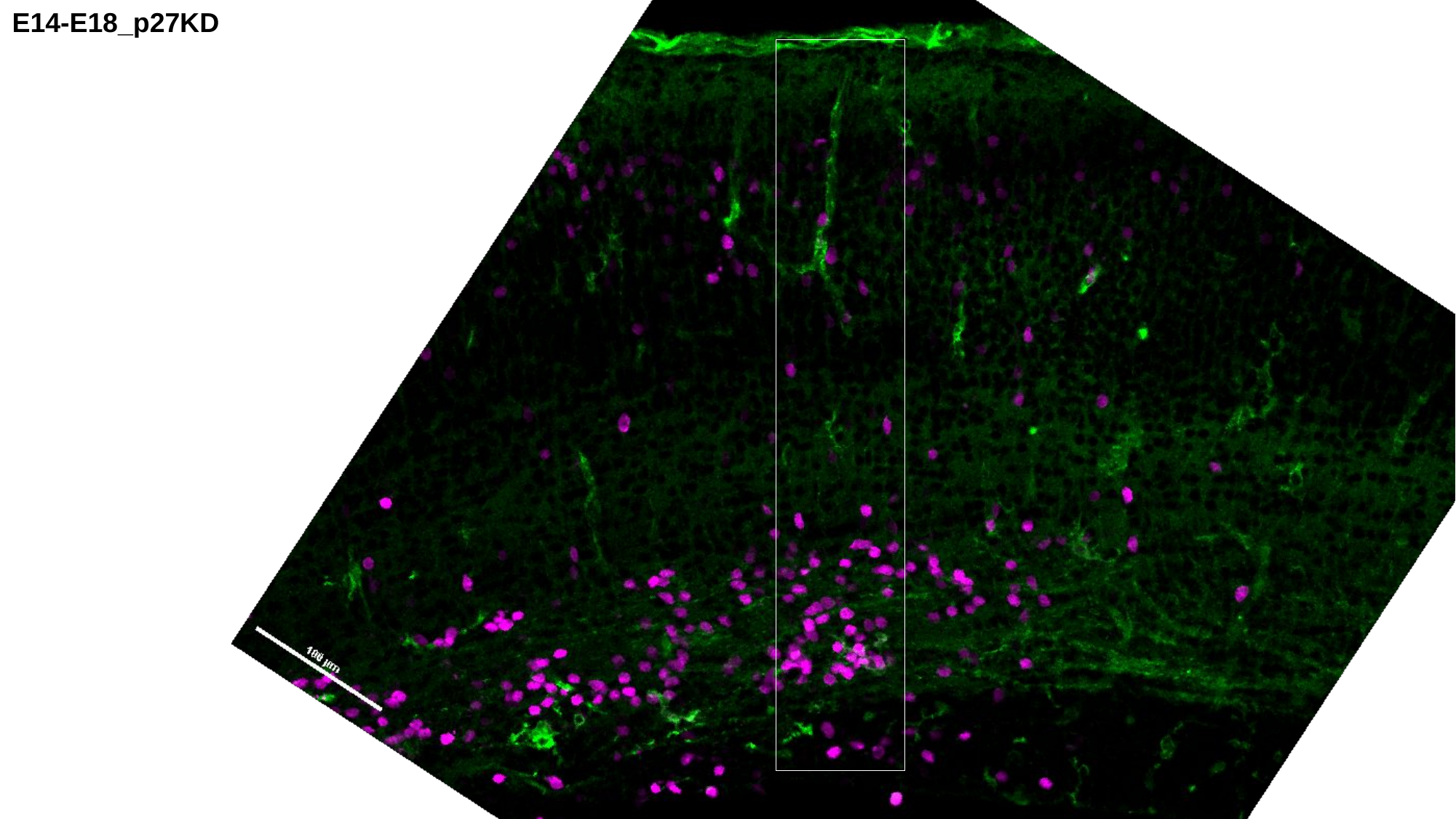

E14-E18_p27KD

## Slide 11
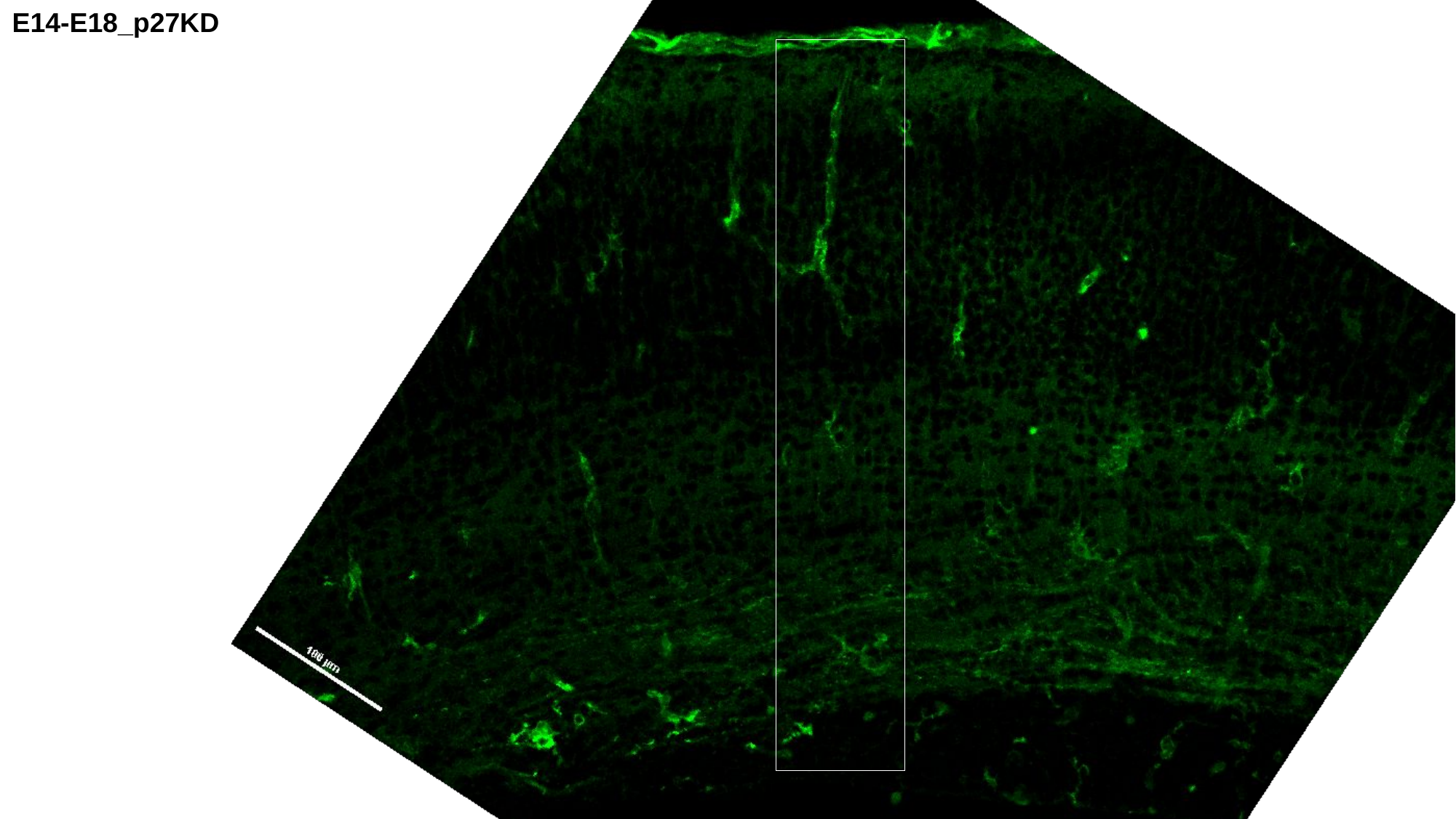

E14-E18_p27KD

## Slide 12
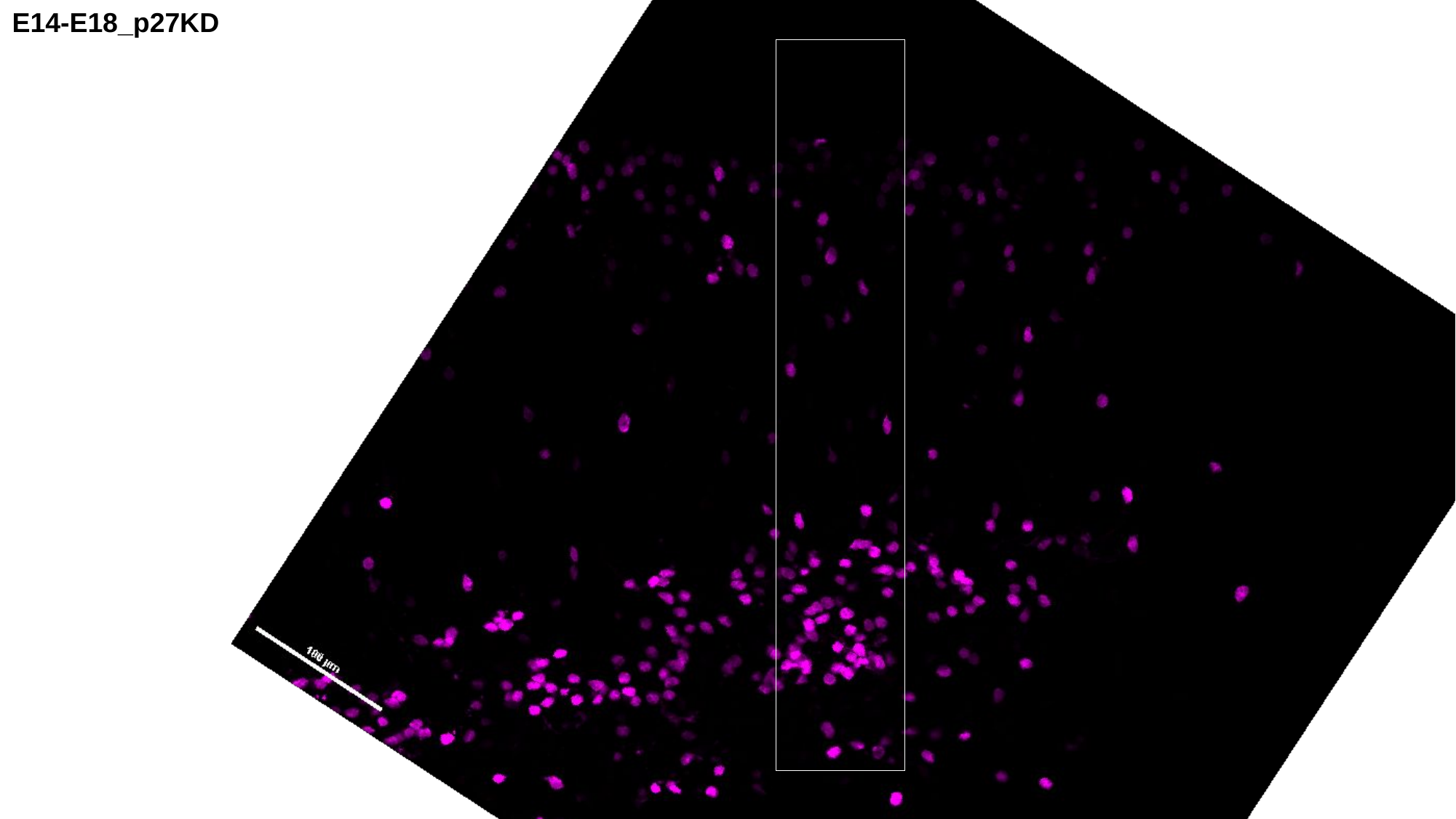

E14-E18_p27KD

## Slide 13
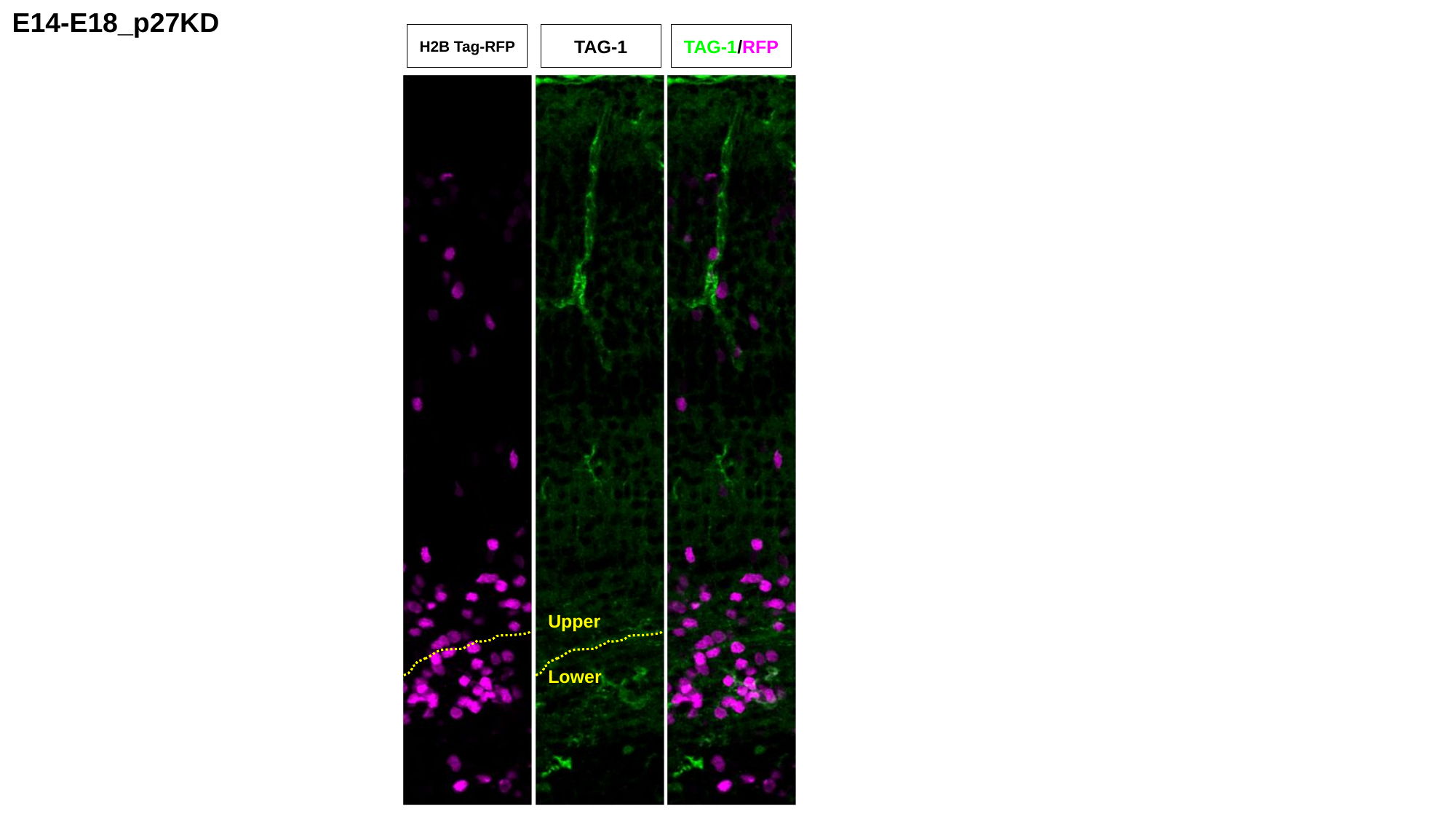

E14-E18_p27KD
H2B Tag-RFP
TAG-1
TAG-1/RFP
Upper
Lower

## Slide 14
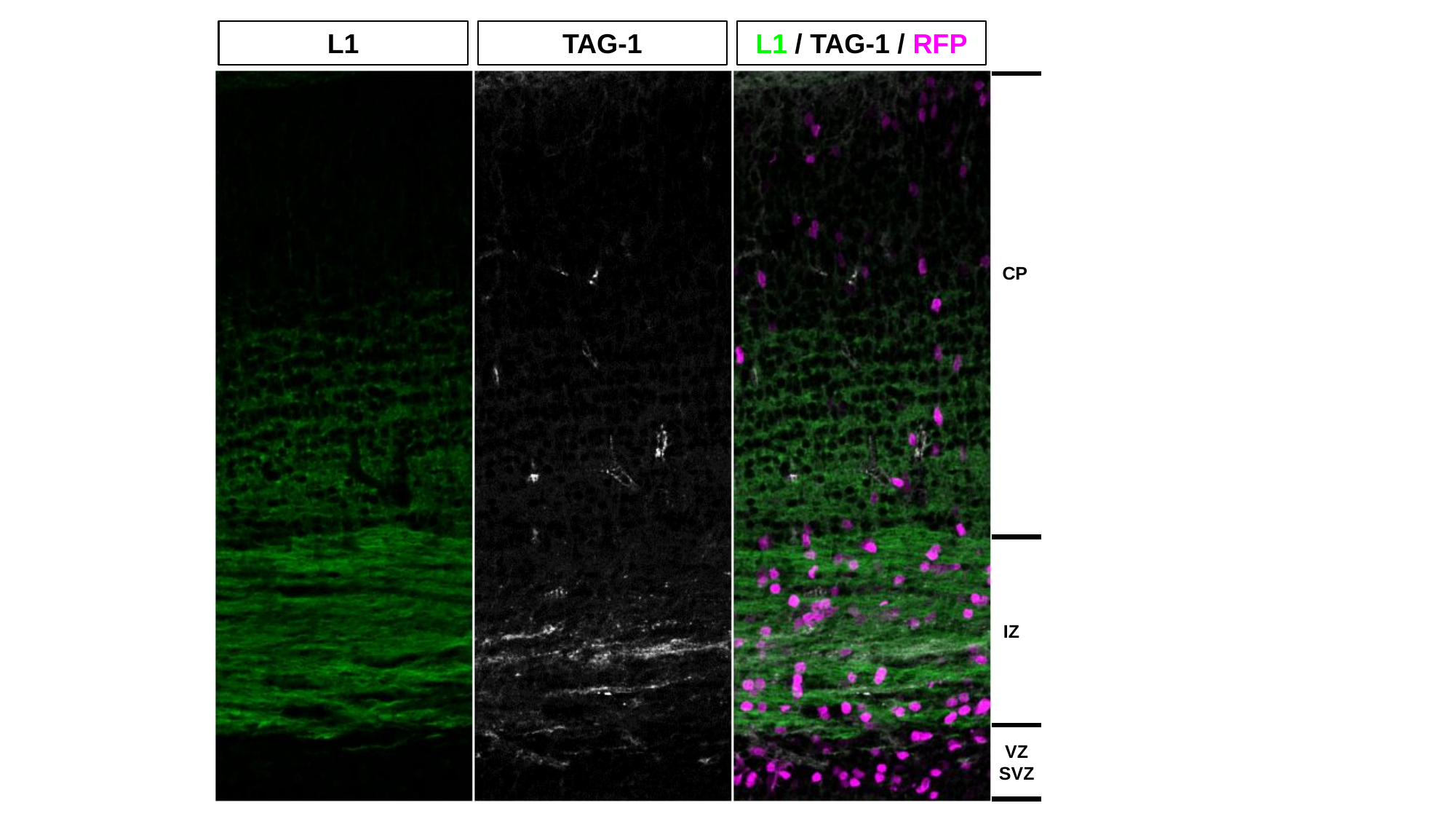

L1
TAG-1
L1 / TAG-1 / RFP
CP
IZ
VZ
SVZ
