# Supplementary material for: Cyclin-dependent kinase inhibitor p18 regulates lineage transitions of excitatory neurons, astrocytes, and interneurons in the mouse cortex
Source: EMBO J. 2024 Dec 12;44(2):382–412. doi: 10.1038/s44318-024-00325-9 (PMC11730326; doi:10.1038/s44318-024-00325-9)
Supplement: Supplementary file 4 — Source data Fig. 2 [file 44318_2024_325_MOESM4_ESM.zip › 2F.pptx]

## Slide 1
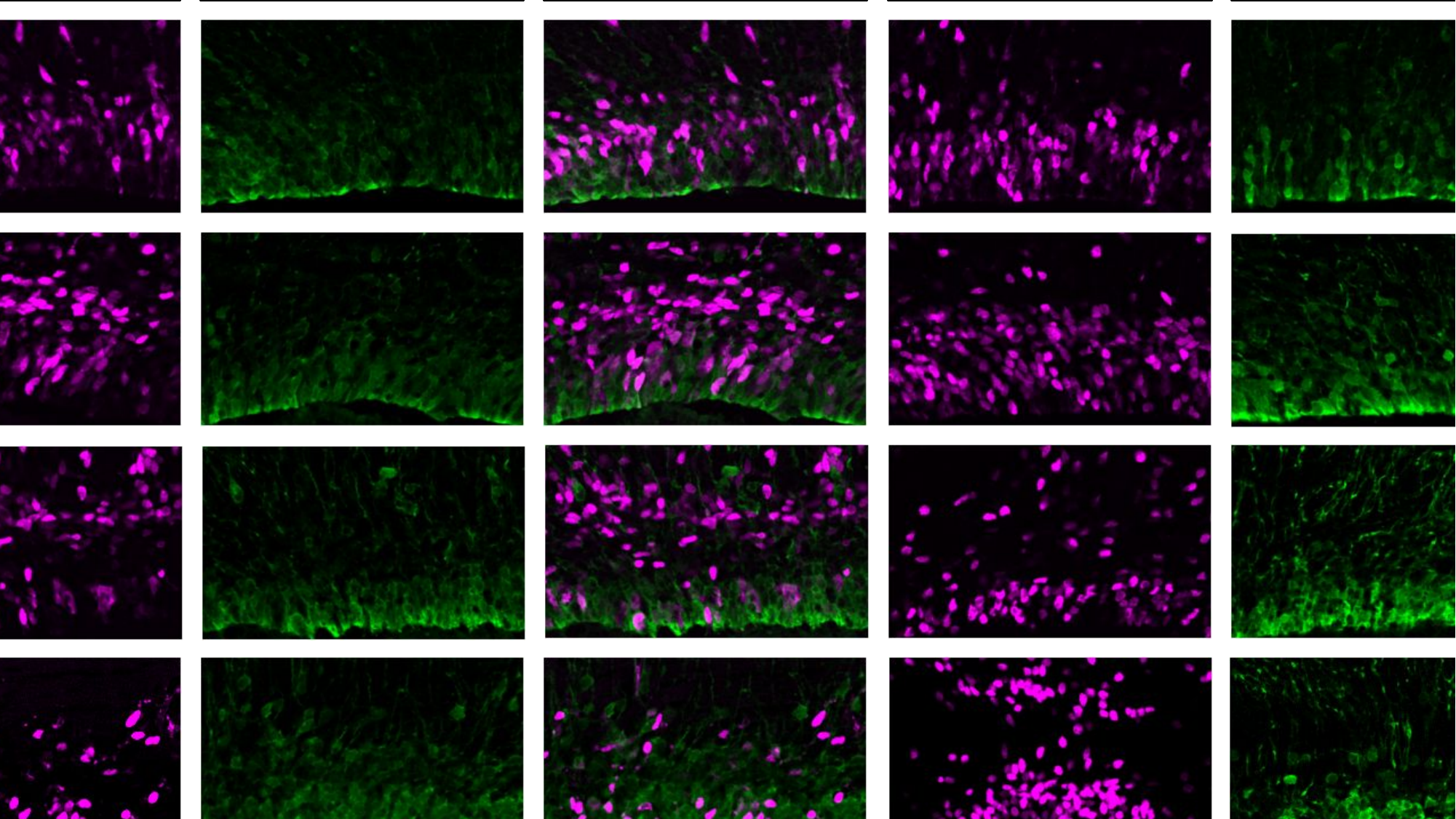

Empty vector (Control)
p18Ink4c
H2B Tag-RFP
Aldh1l1-EGFP
EGFP / RFP
H2B Tag-RFP
Aldh1l1-EGFP
EGFP / RFP
E16.5
E17.5
EPed at E15.5
E18.5
P0

## Slide 2
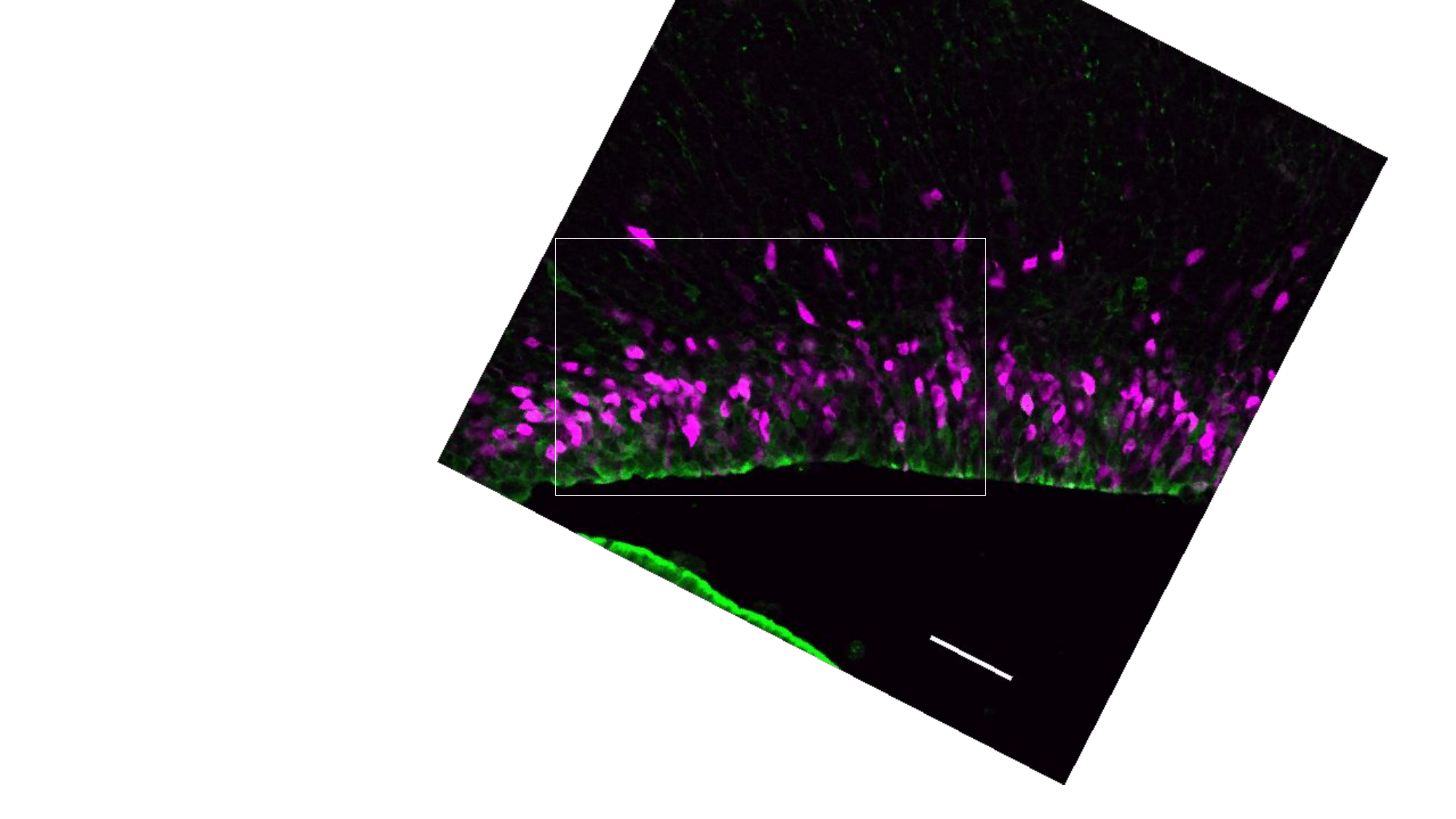

## Slide 3
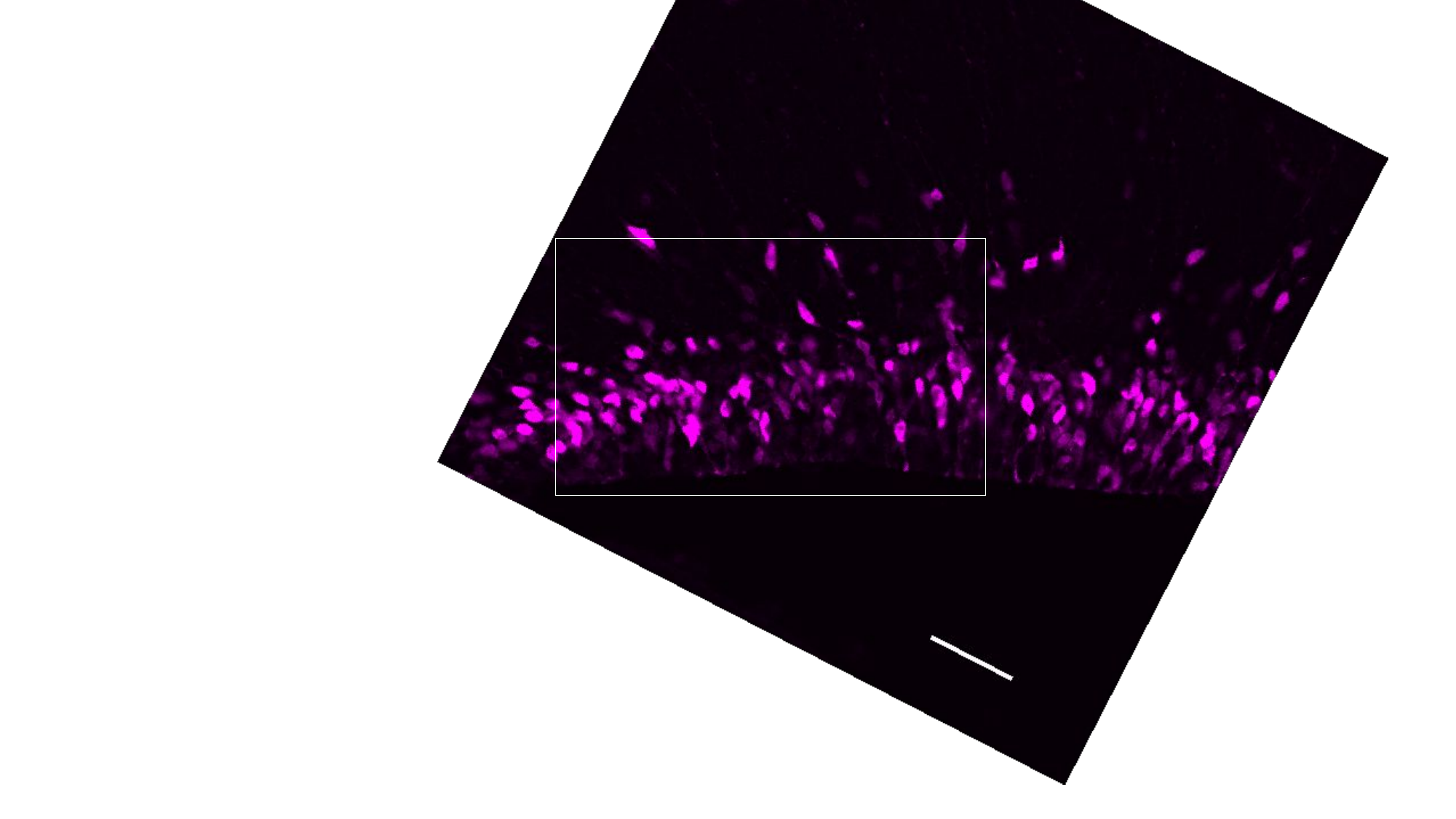

## Slide 4
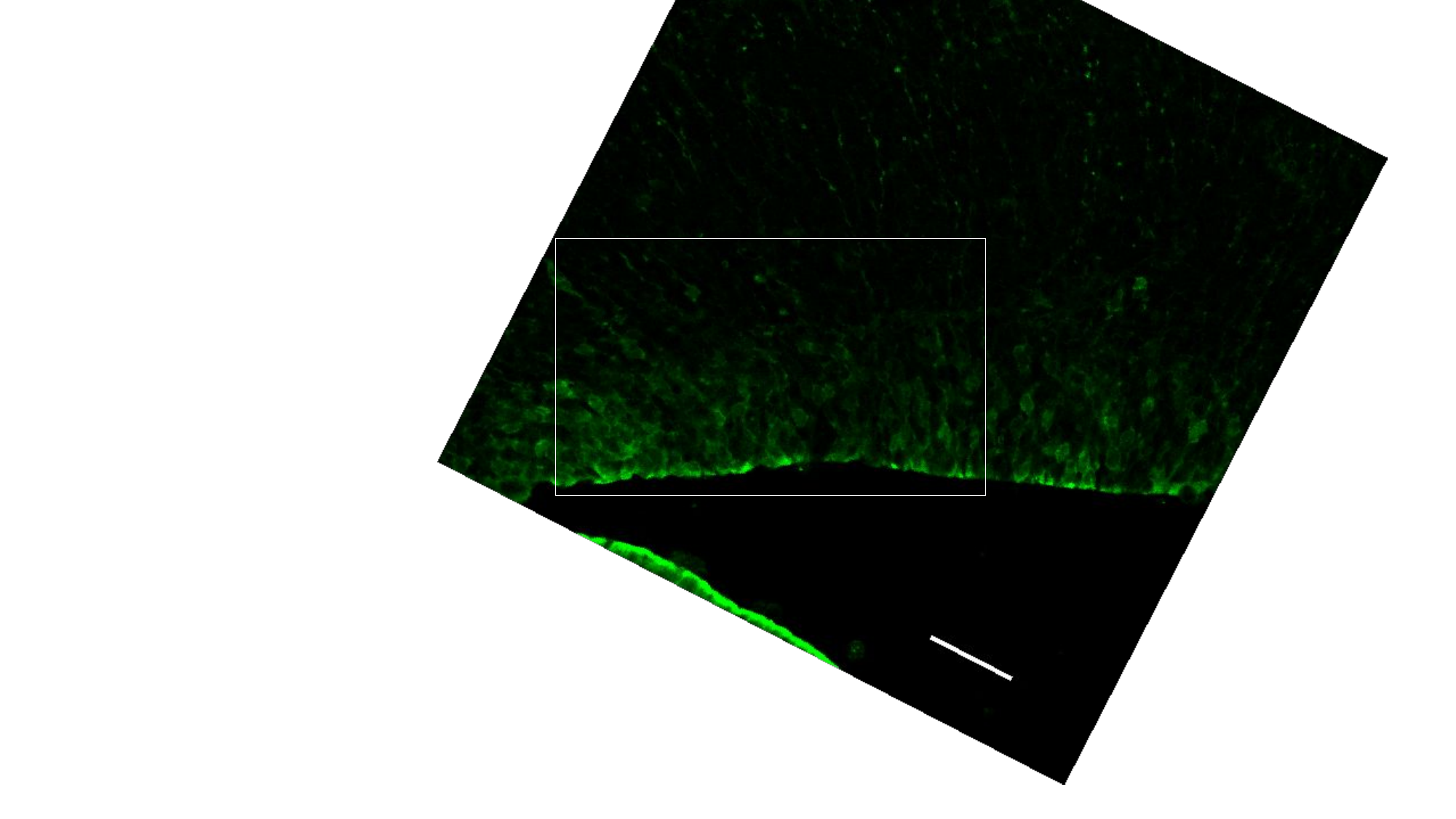

## Slide 5
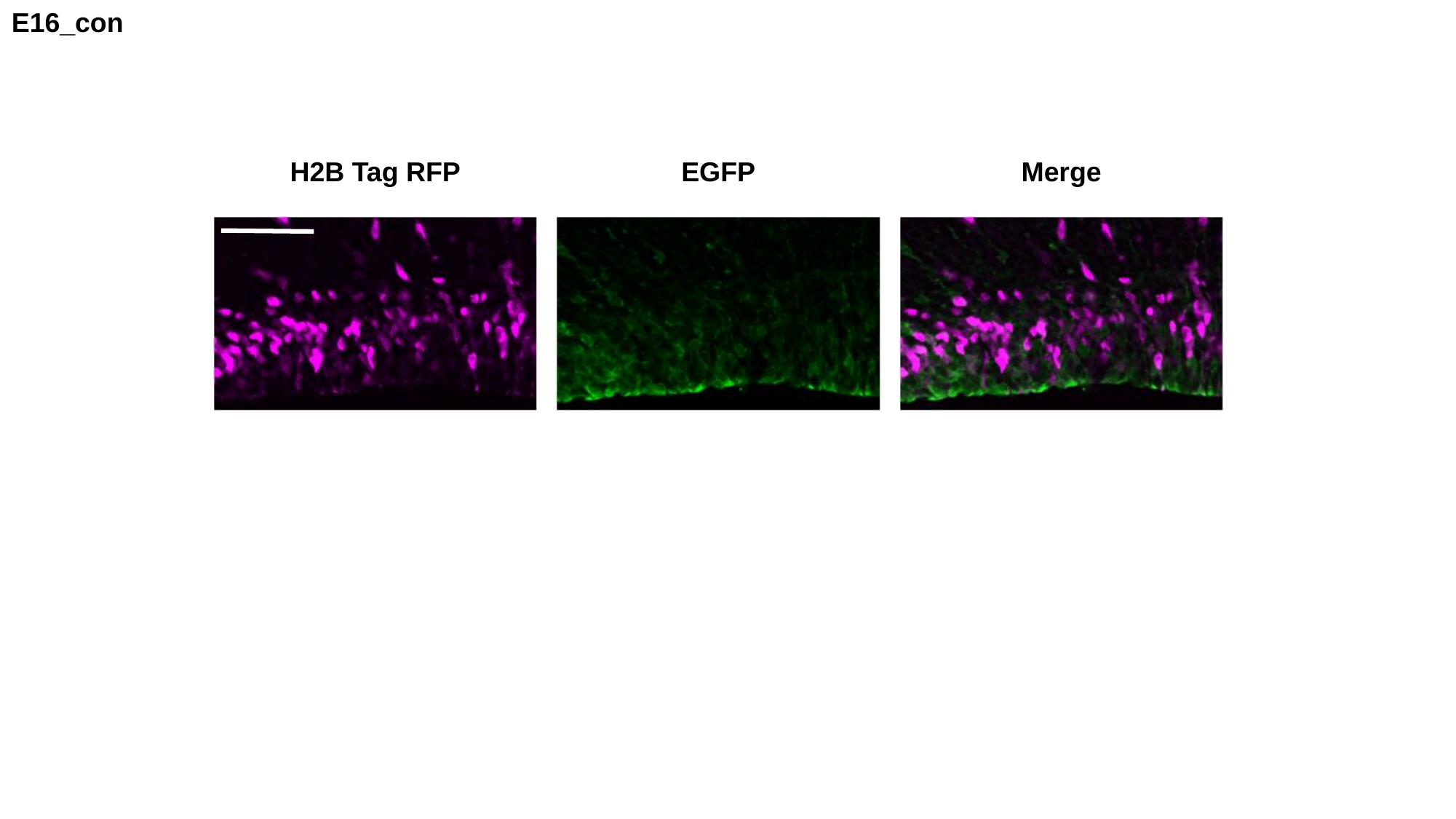

E16_con
H2B Tag RFP
EGFP
Merge

## Slide 6
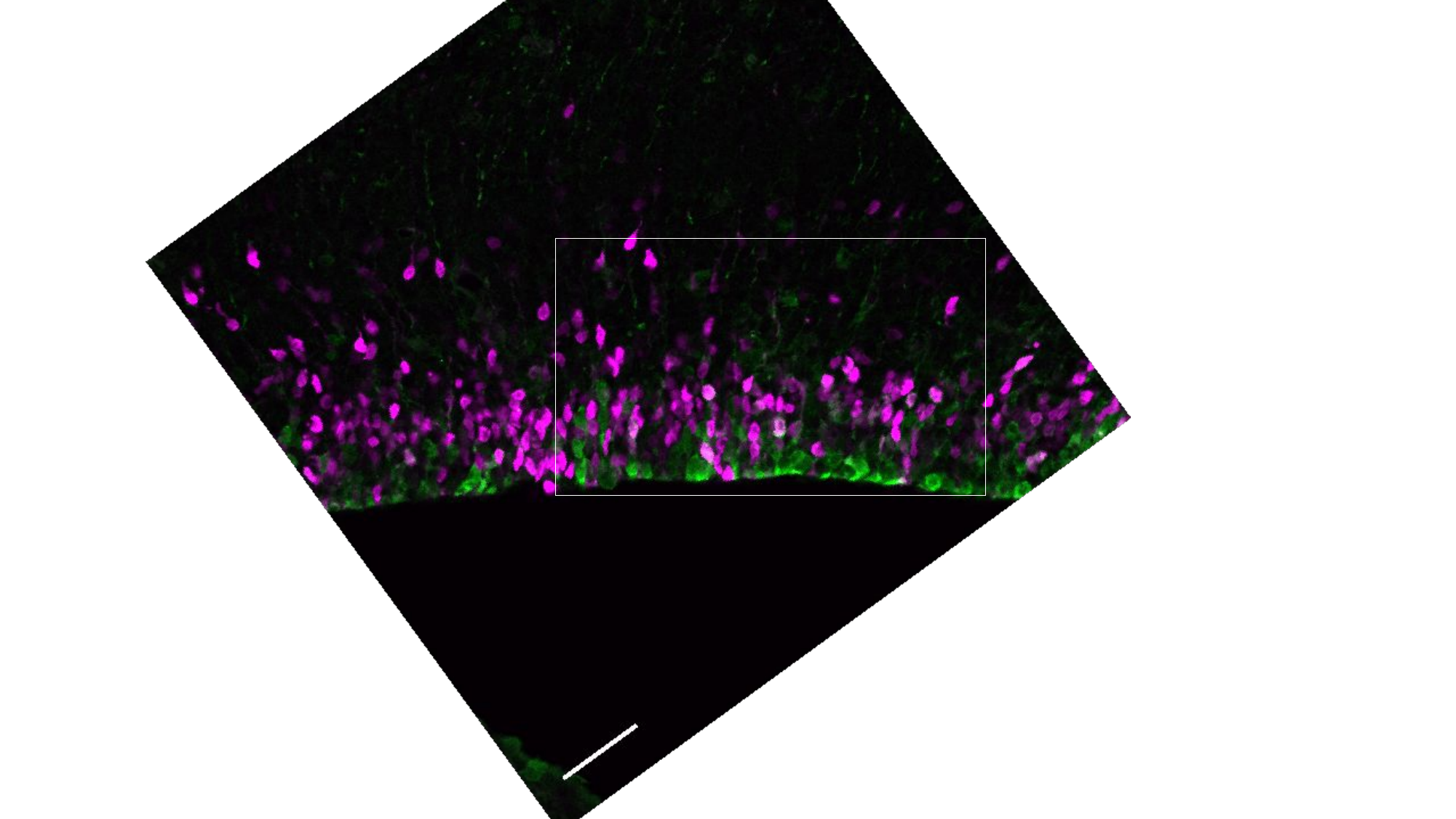

## Slide 7
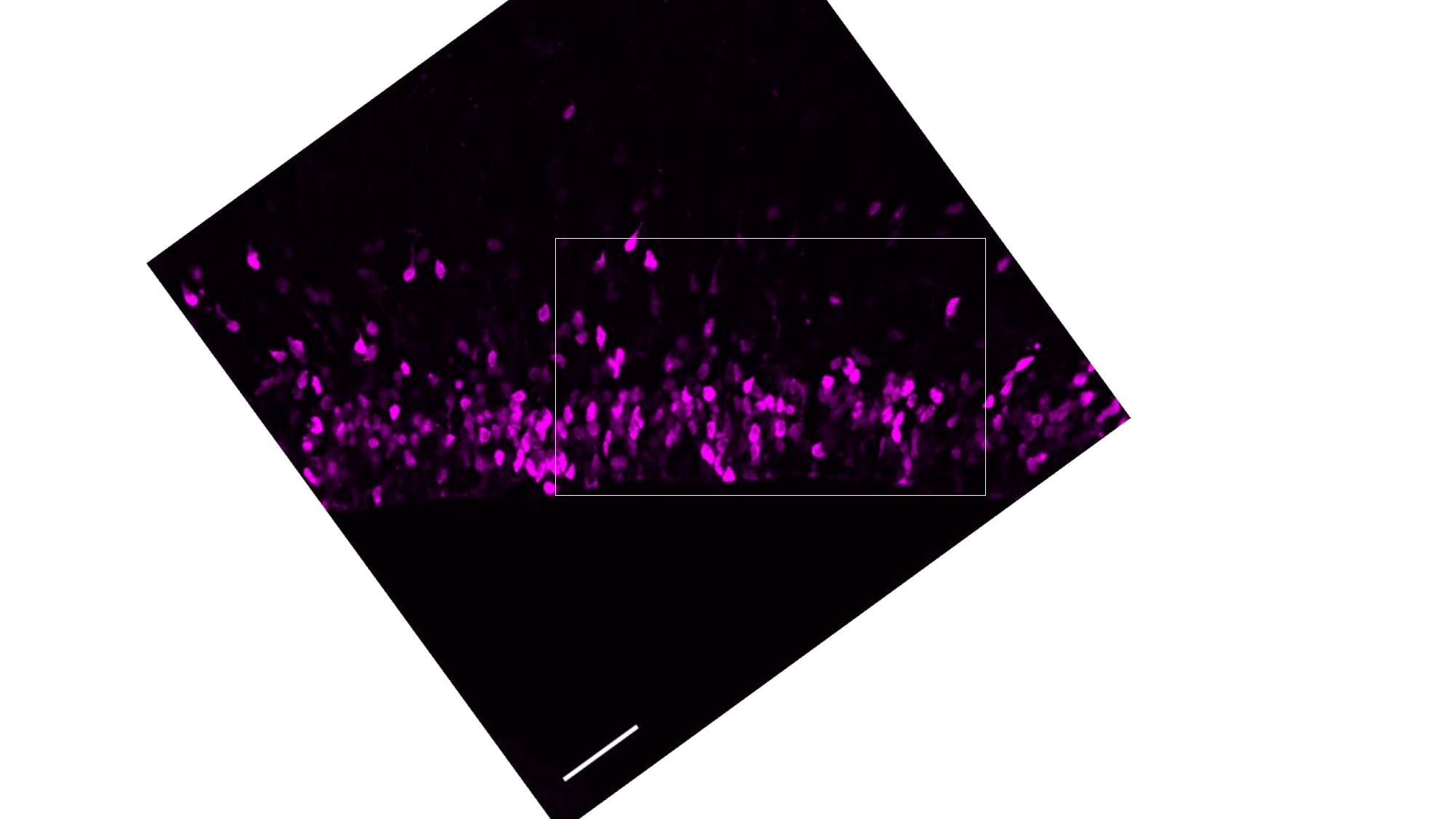

## Slide 8
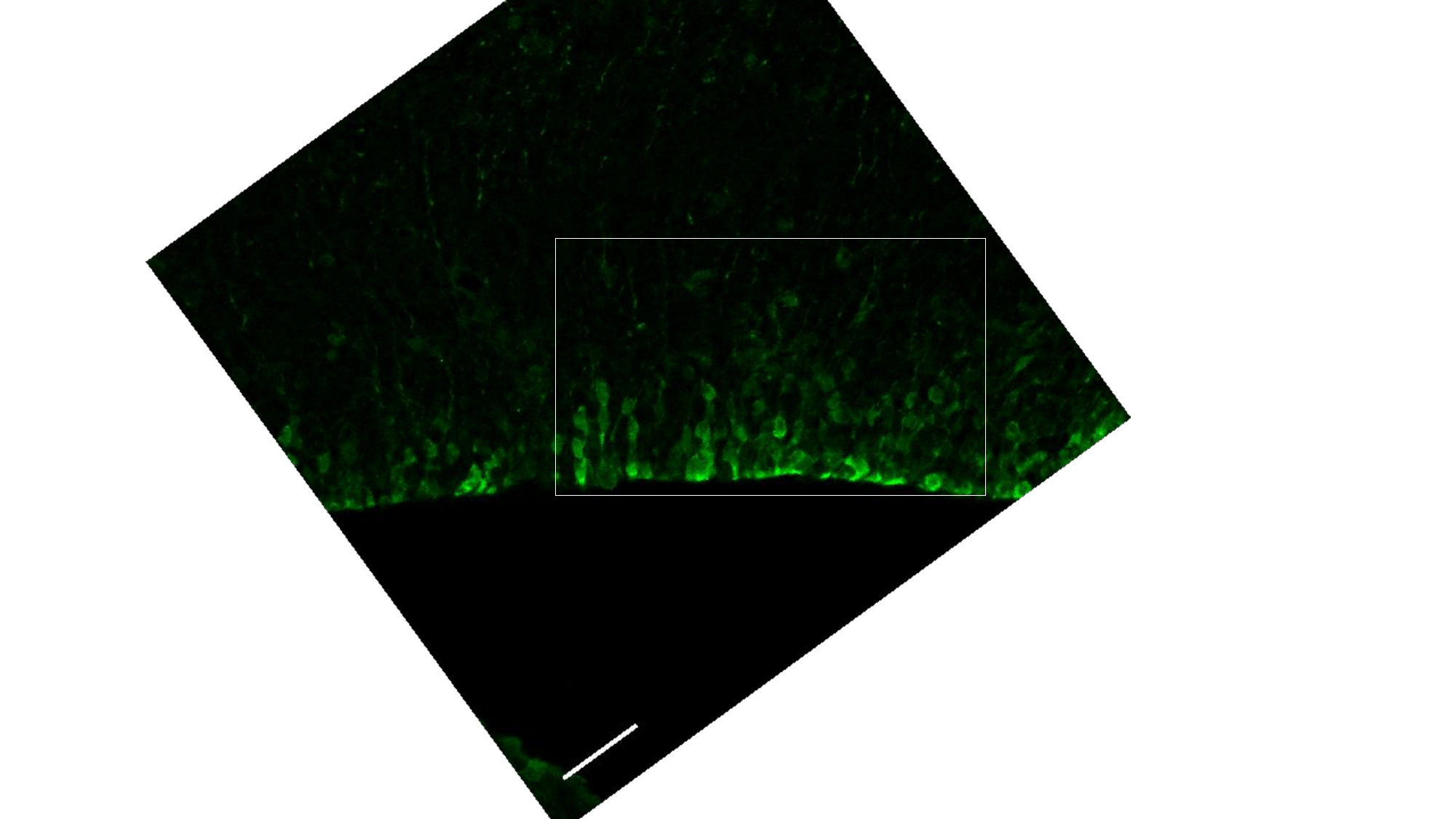

## Slide 9
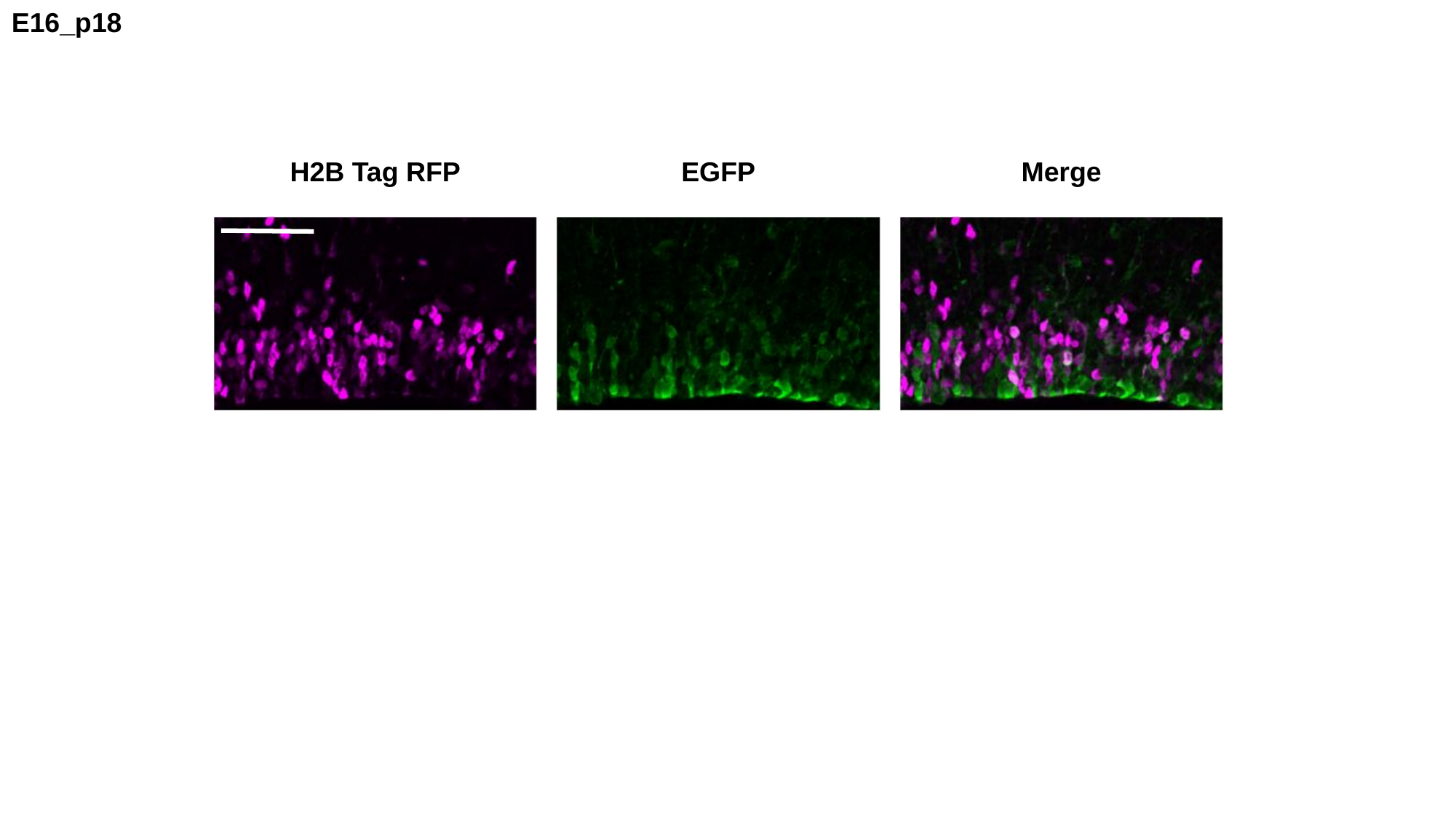

E16_p18
H2B Tag RFP
EGFP
Merge

## Slide 10
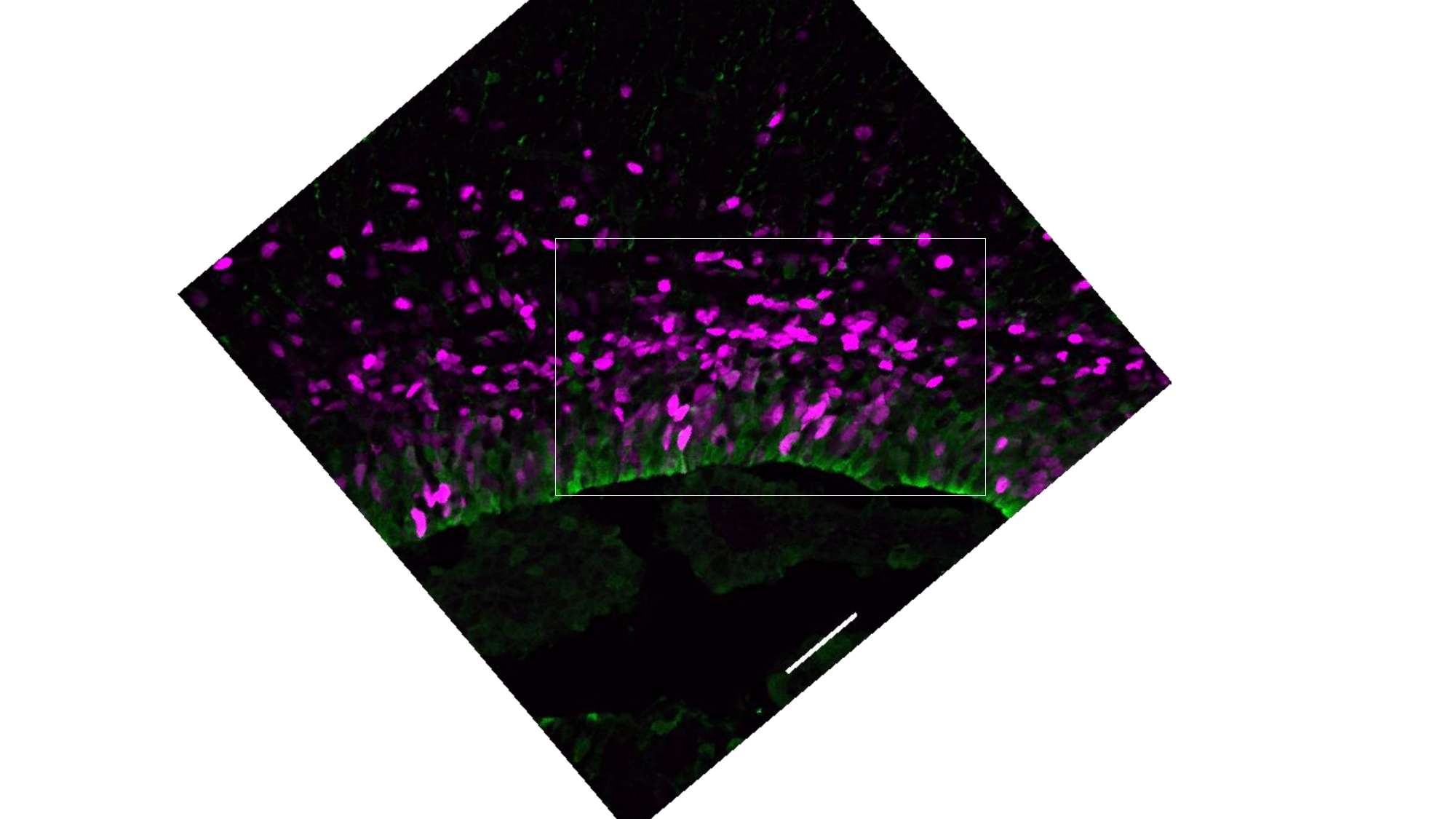

## Slide 11
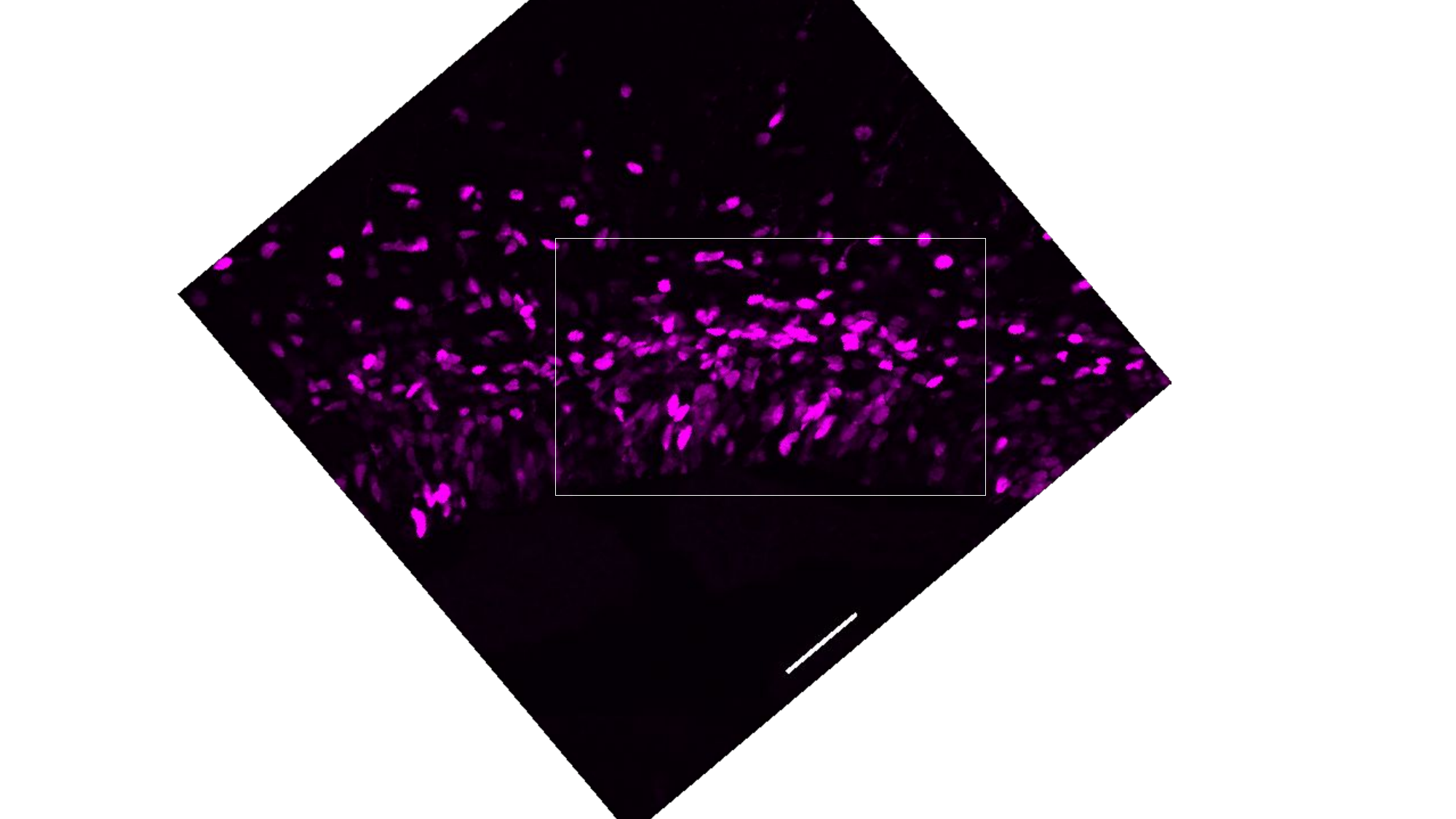

## Slide 12
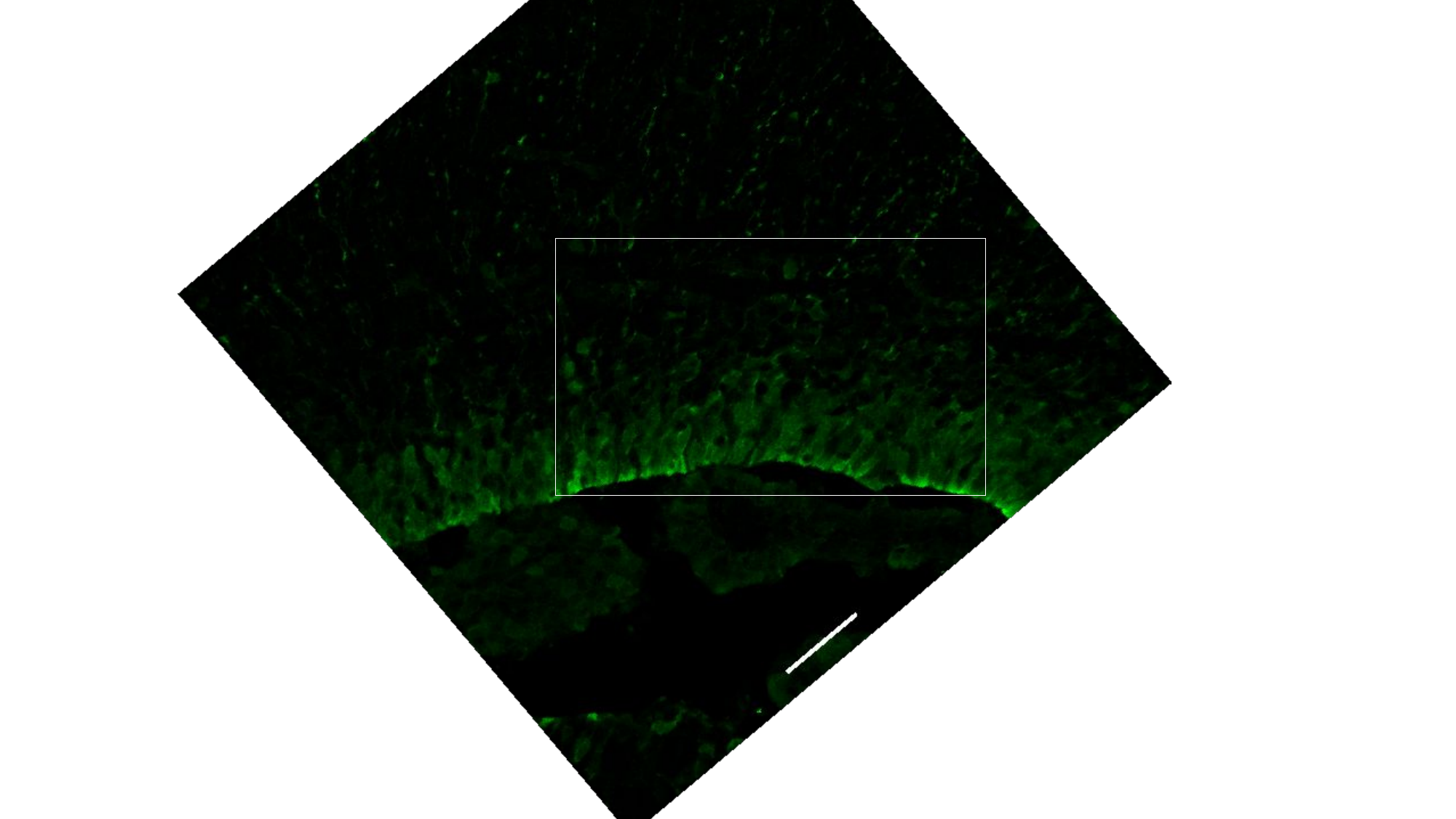

## Slide 13
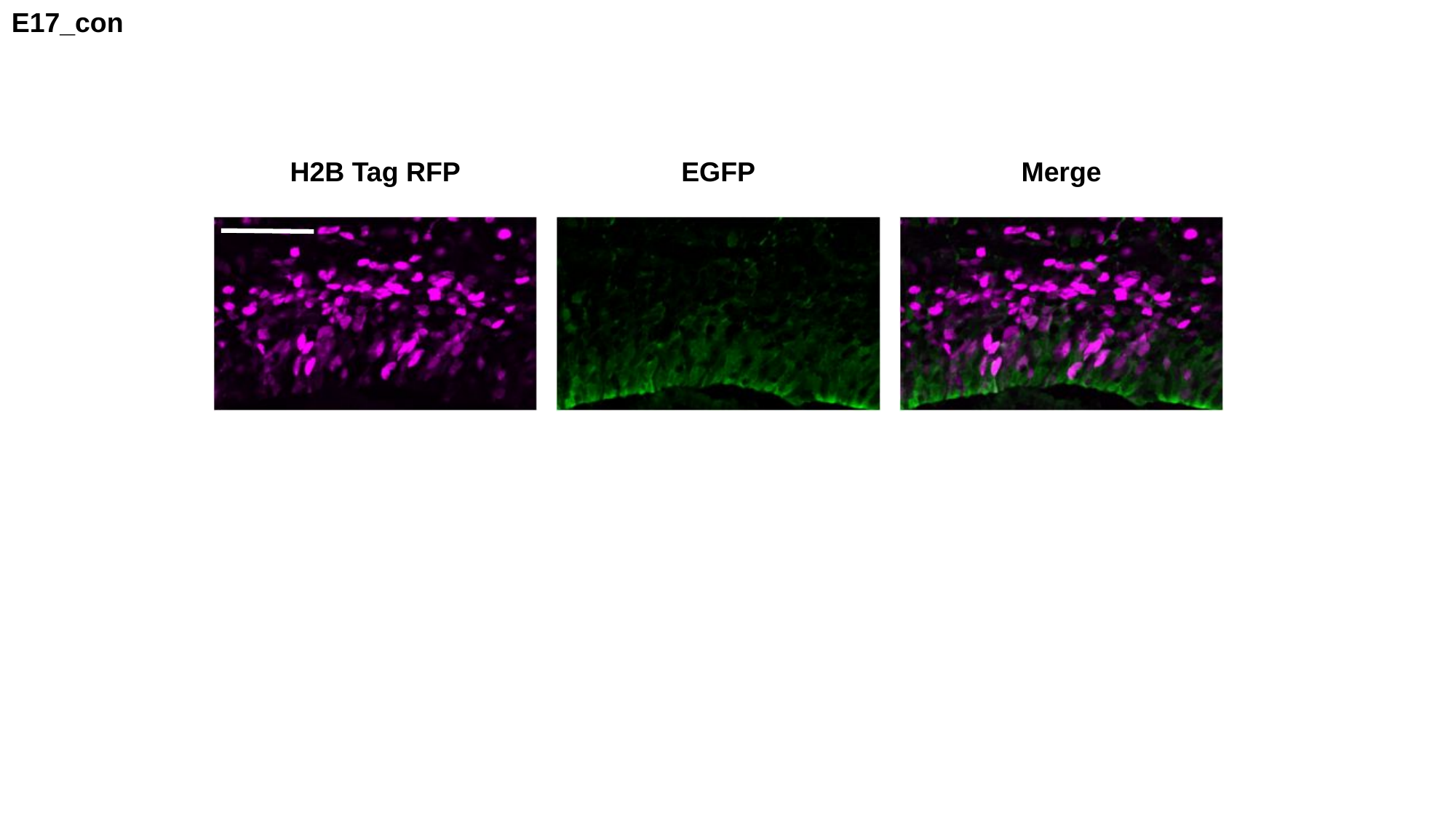

E17_con
H2B Tag RFP
EGFP
Merge

## Slide 14
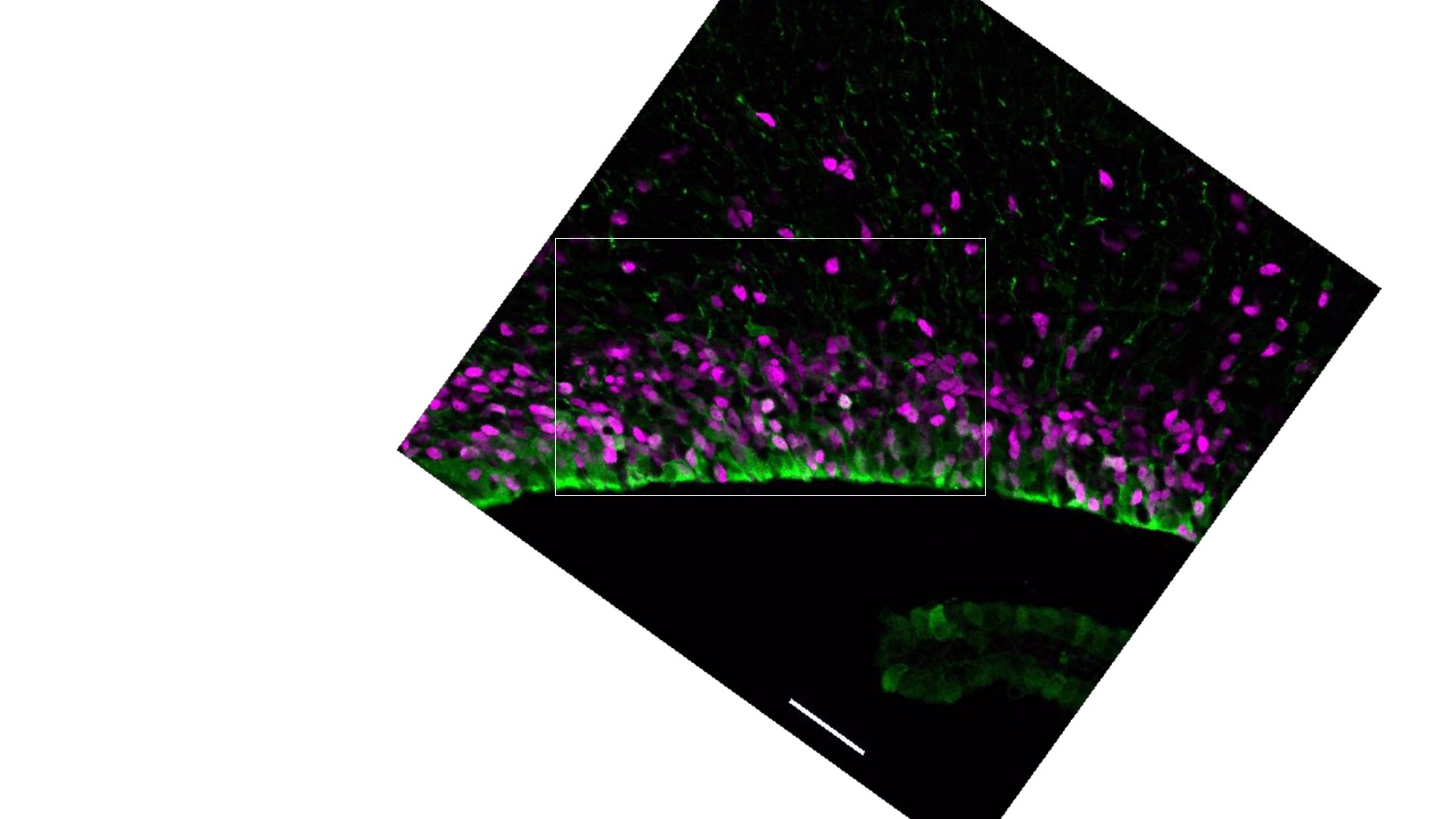

## Slide 15
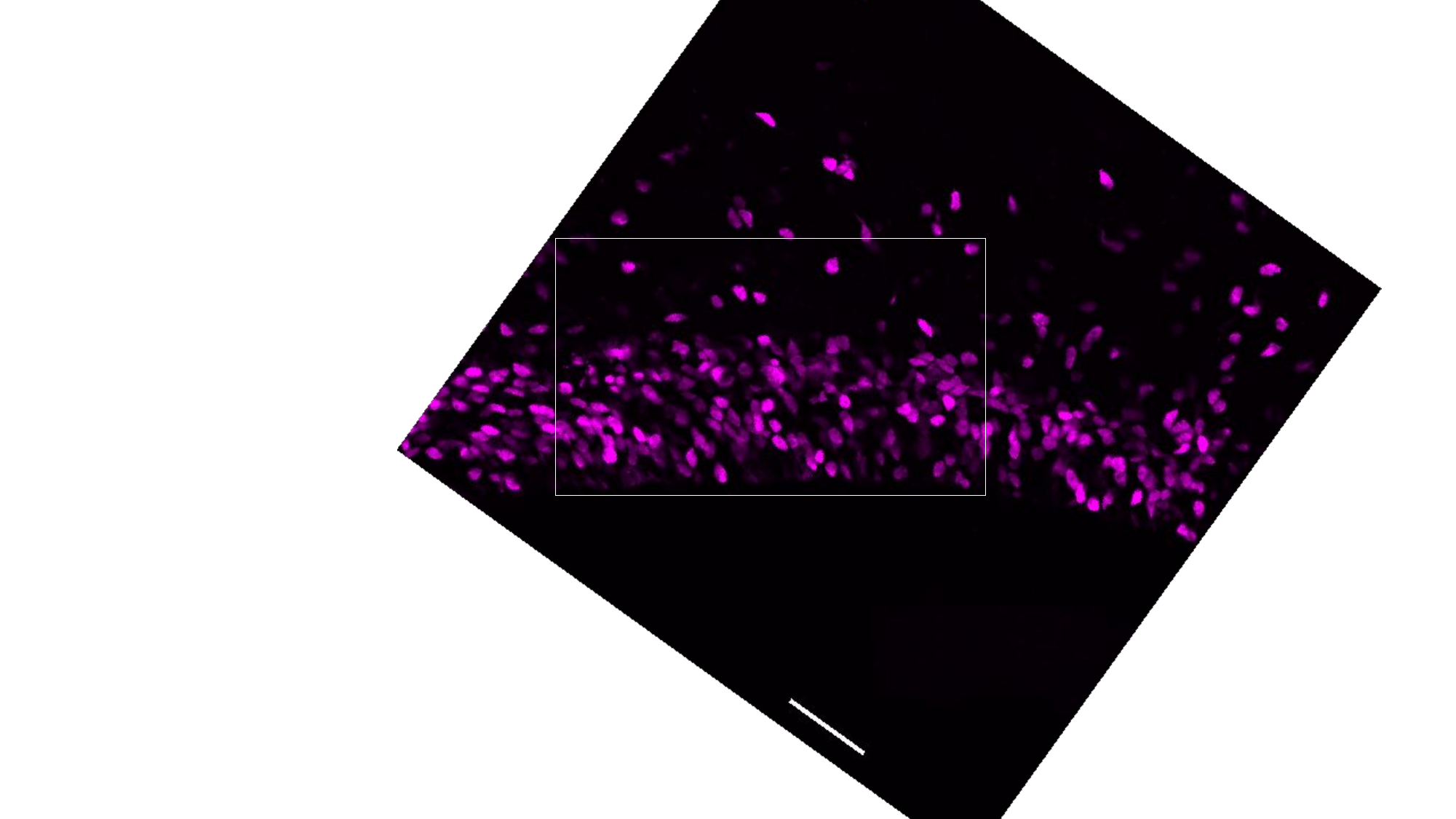

## Slide 16
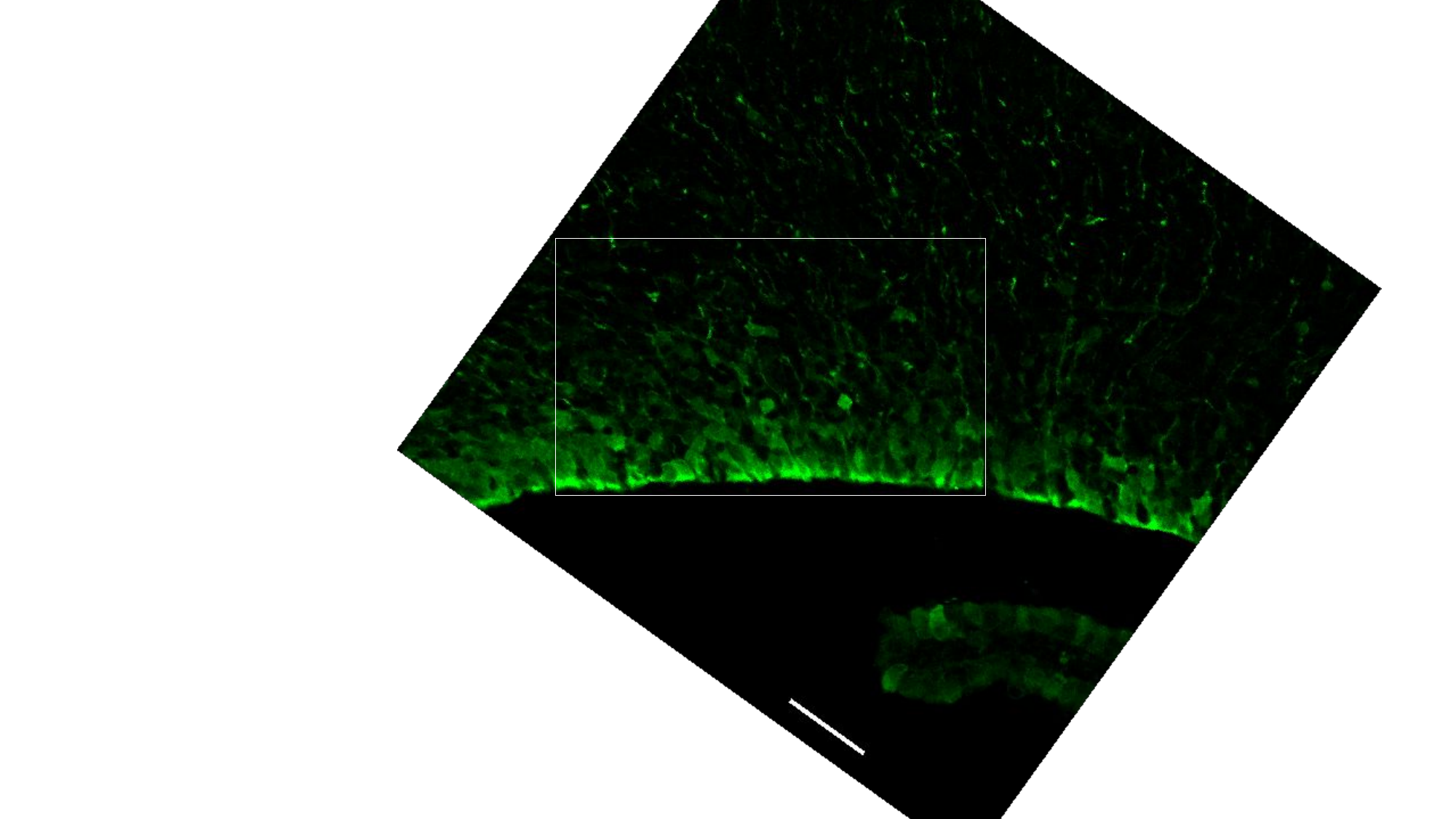

## Slide 17
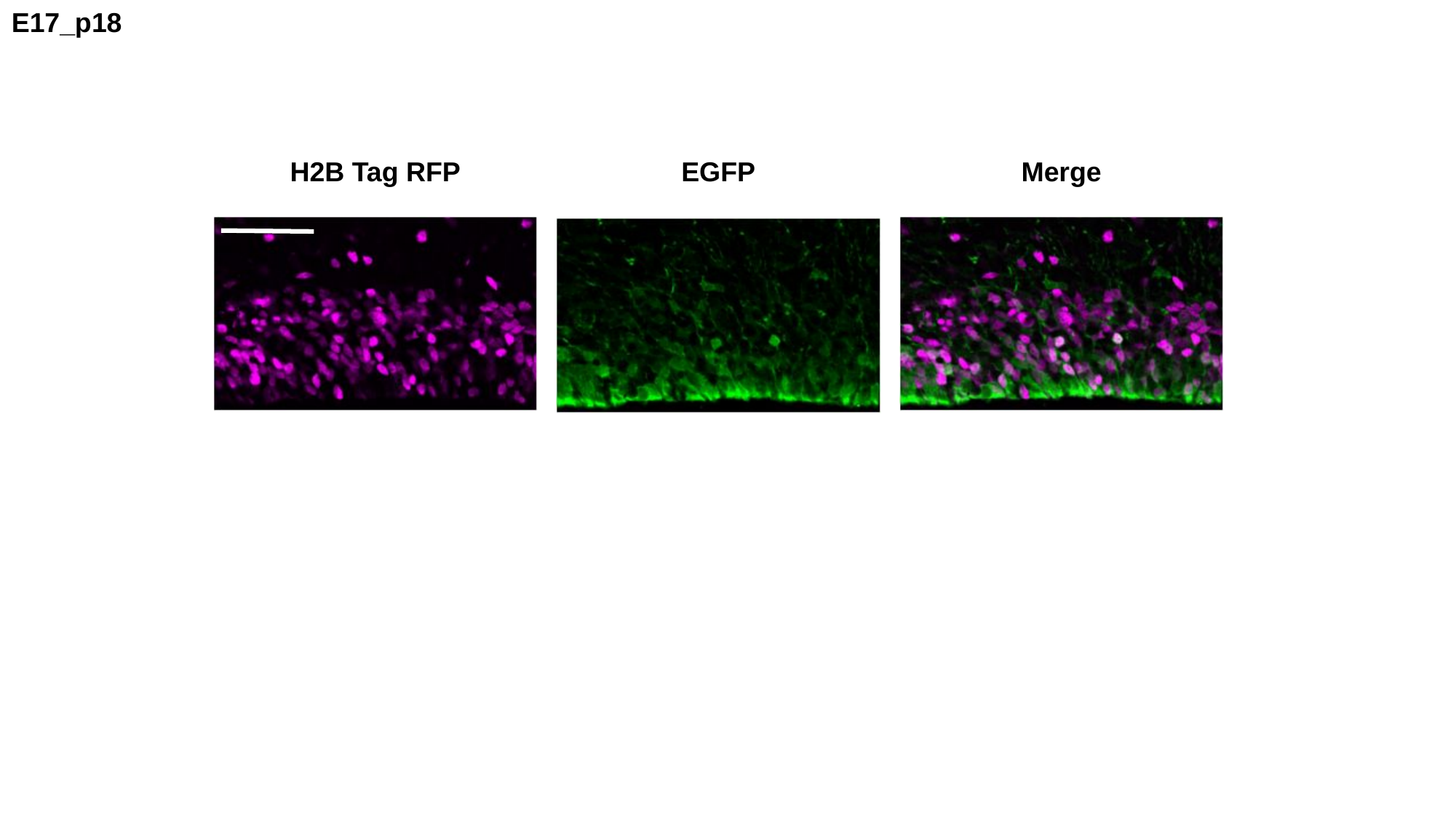

E17_p18
H2B Tag RFP
EGFP
Merge

## Slide 18
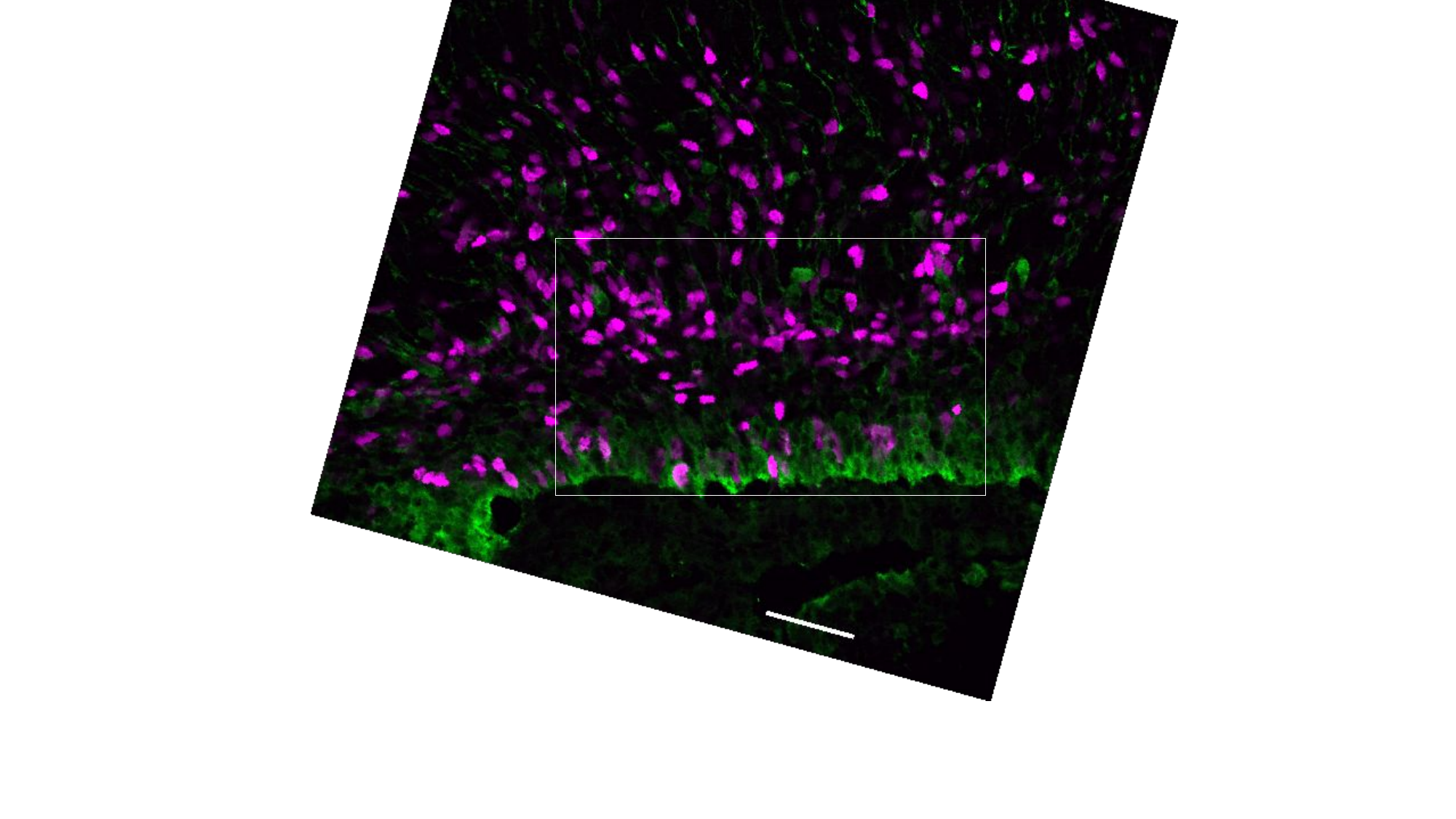

## Slide 19
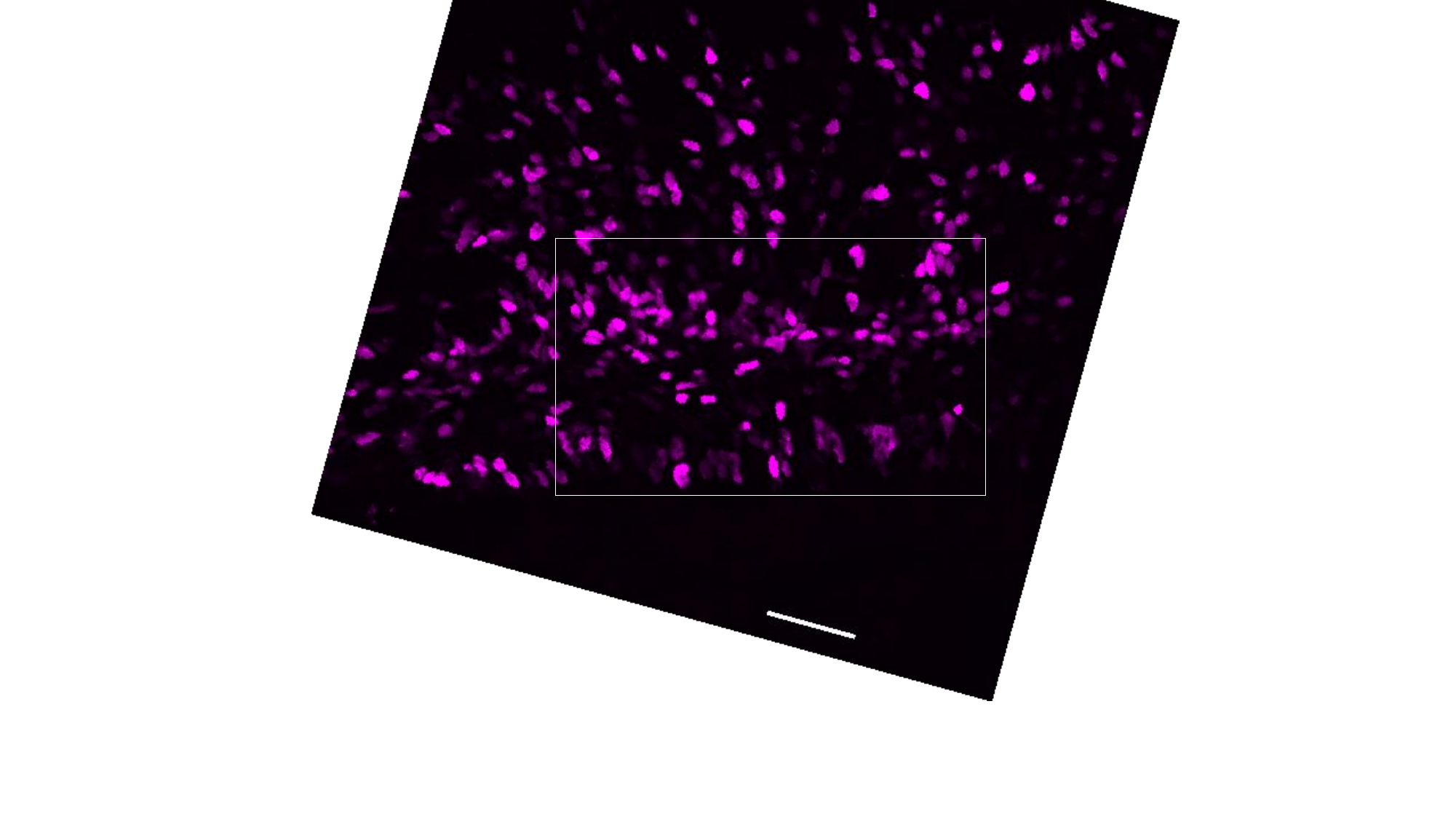

## Slide 20
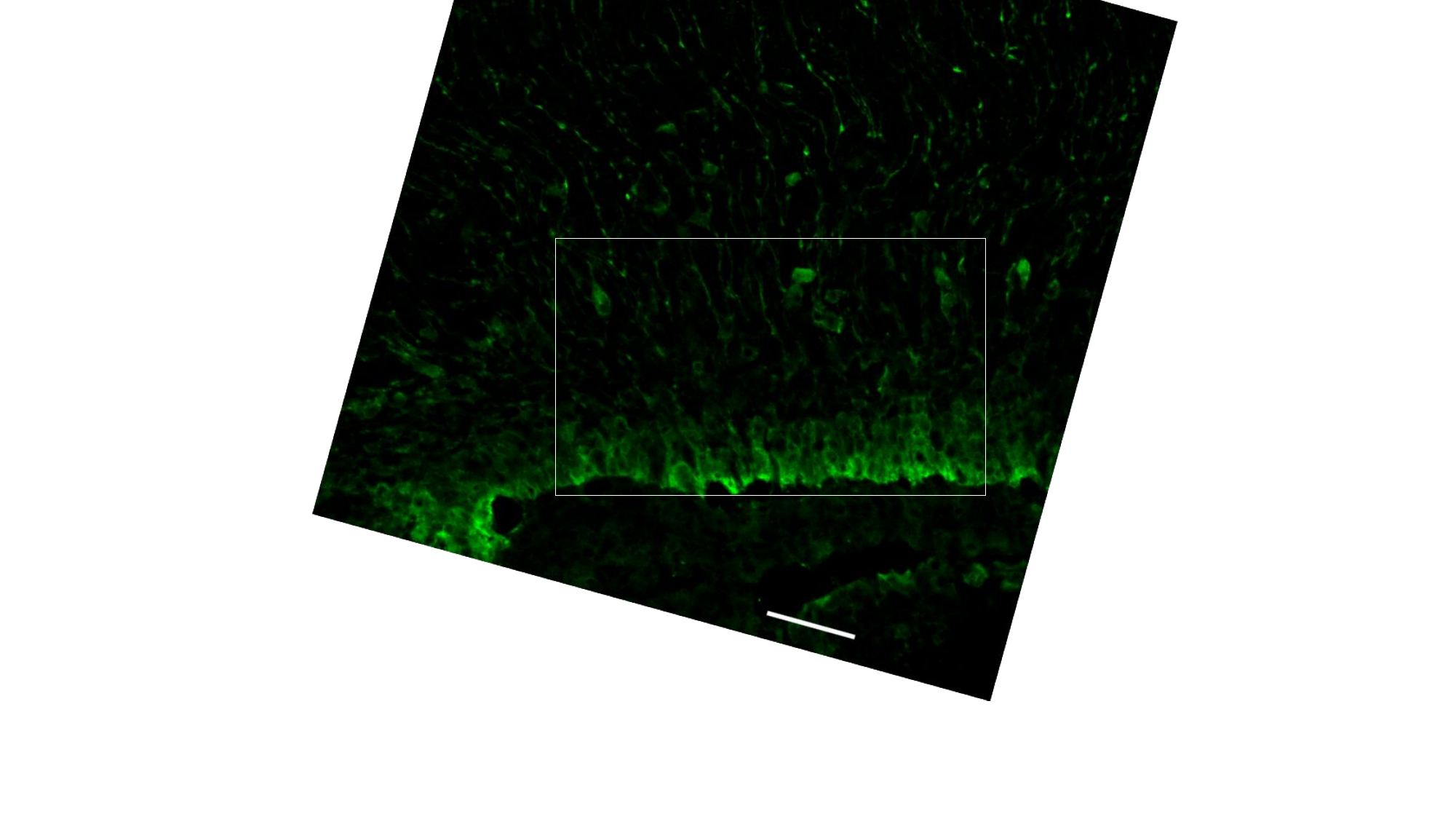

## Slide 21
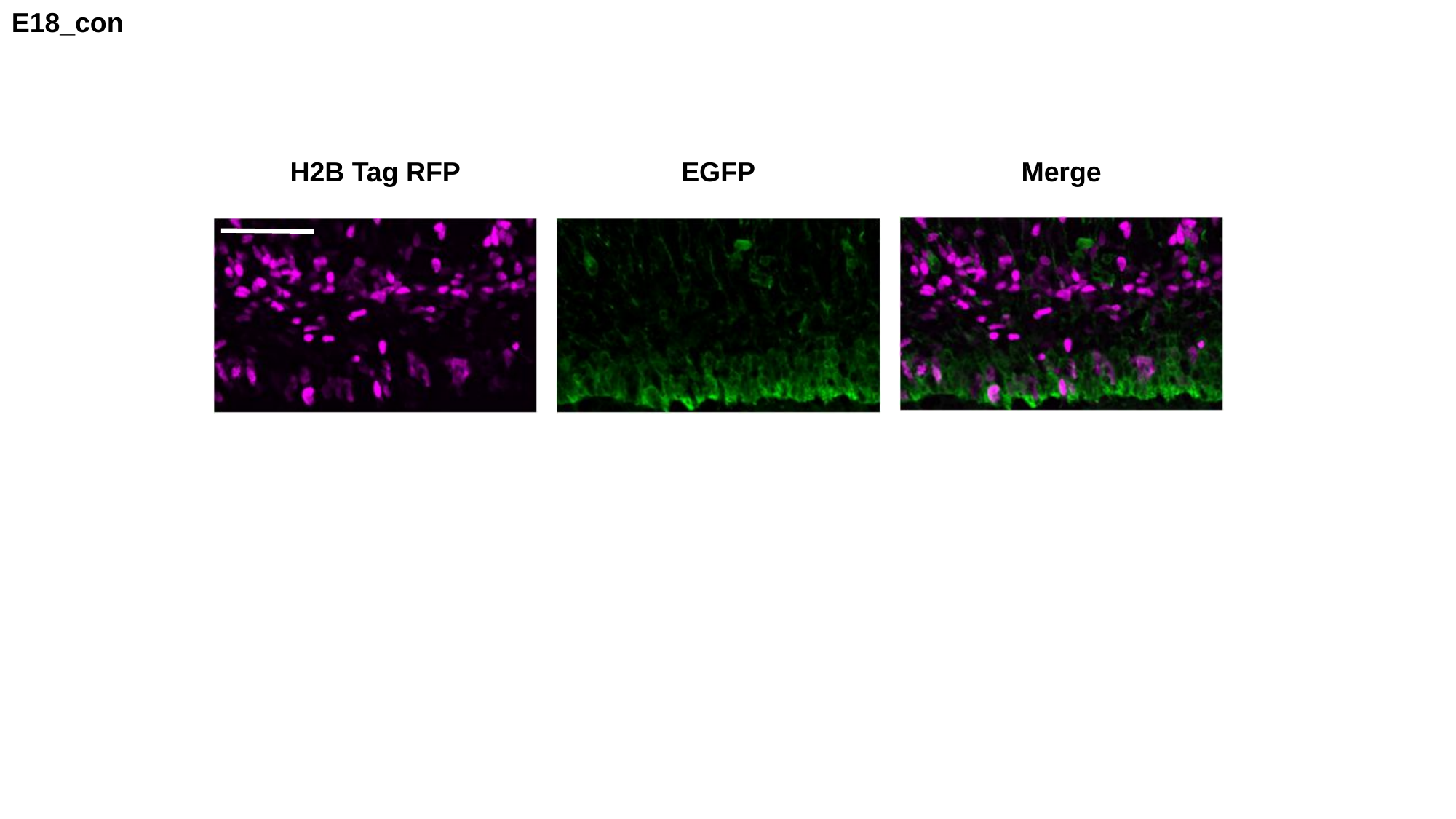

E18_con
H2B Tag RFP
EGFP
Merge

## Slide 22
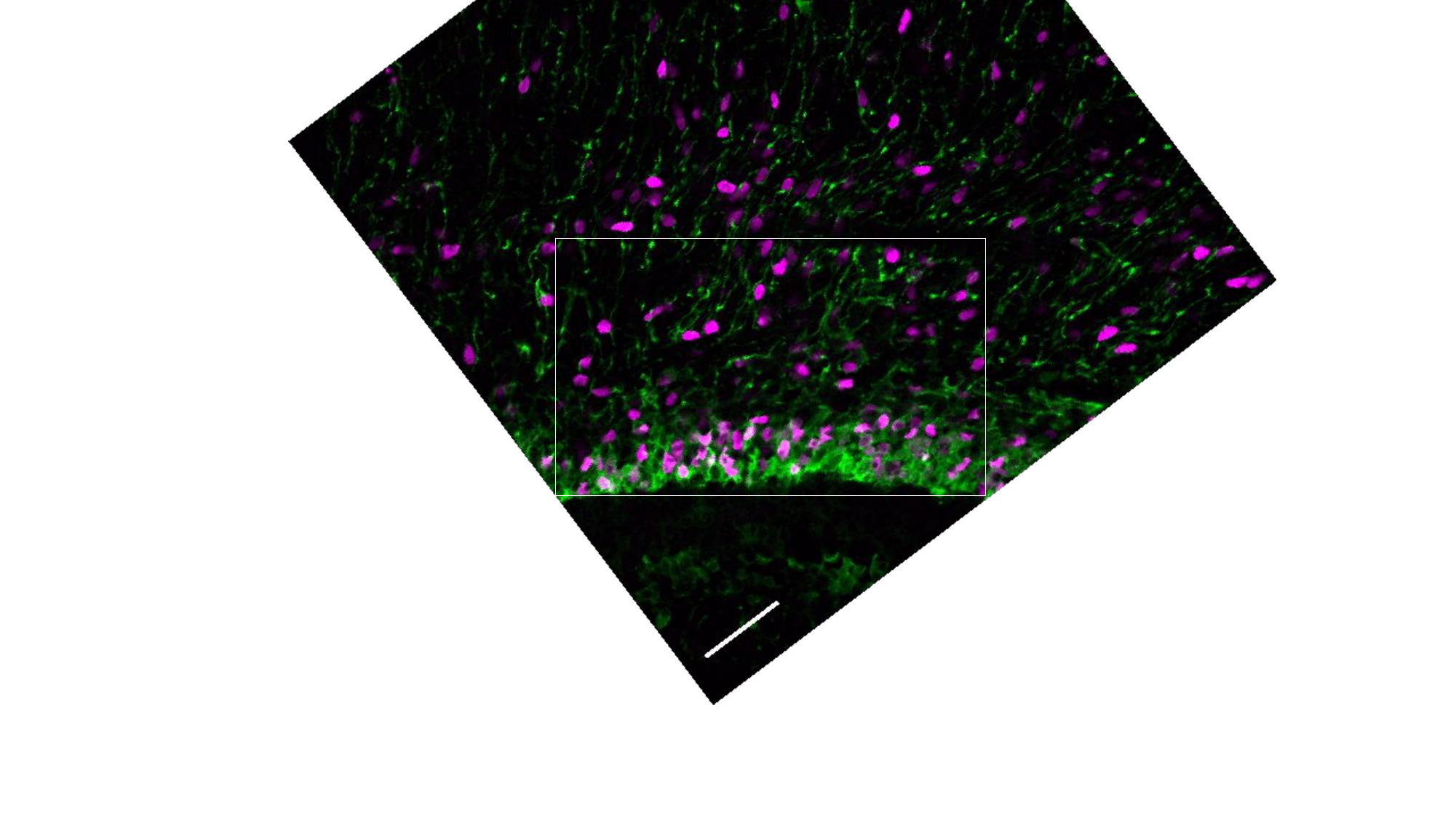

## Slide 23
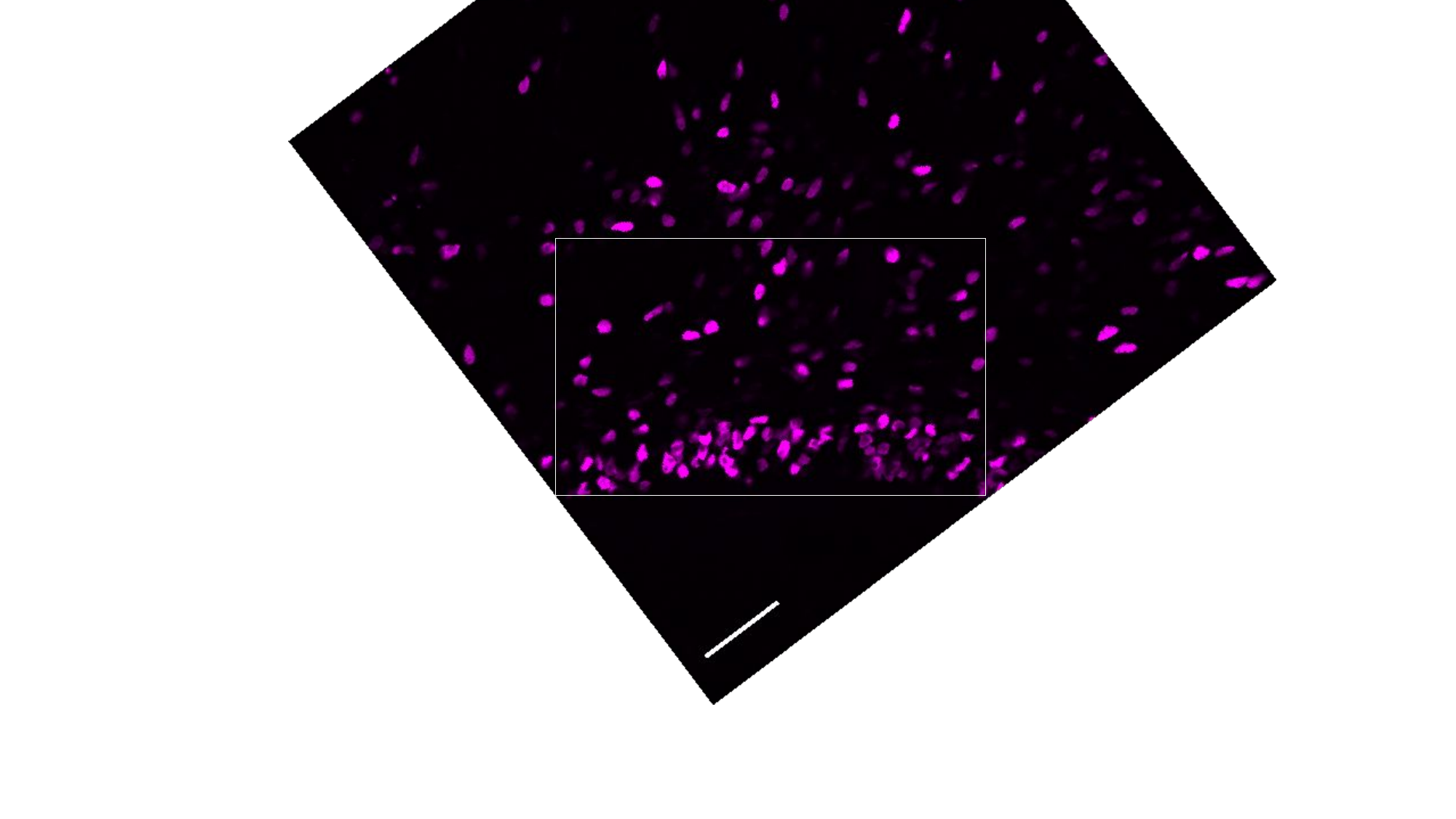

## Slide 24
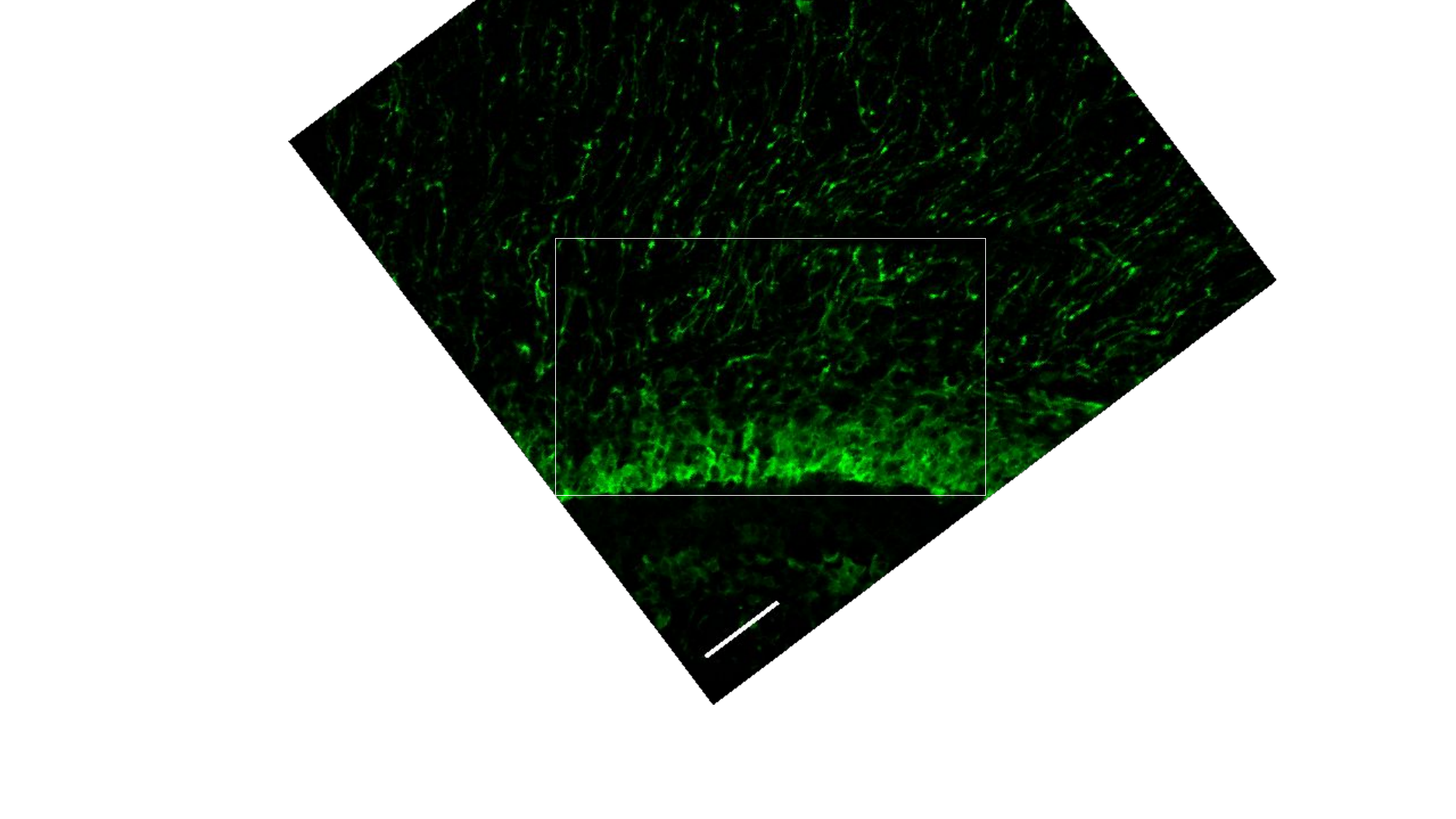

## Slide 25
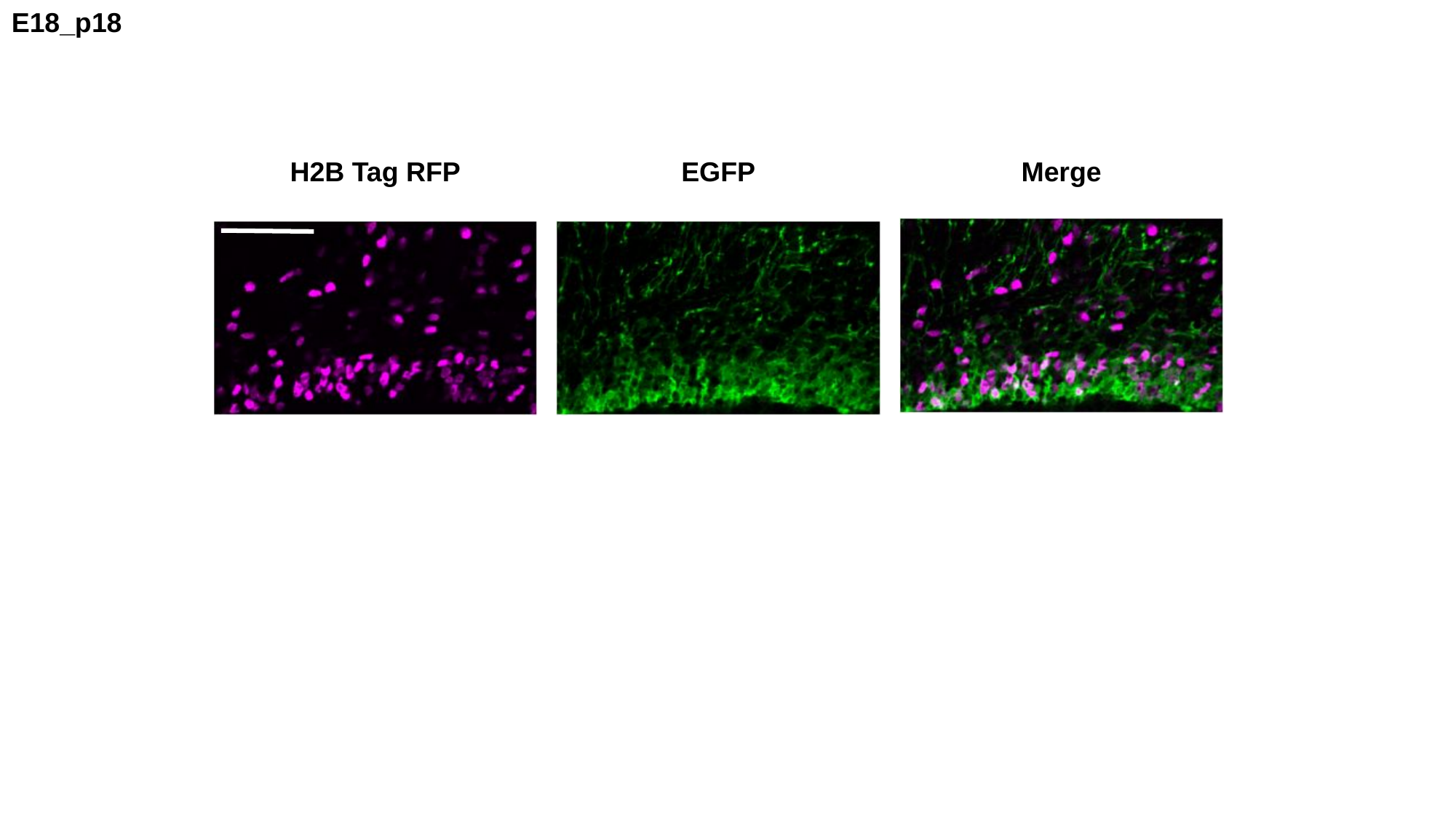

E18_p18
H2B Tag RFP
EGFP
Merge

## Slide 26
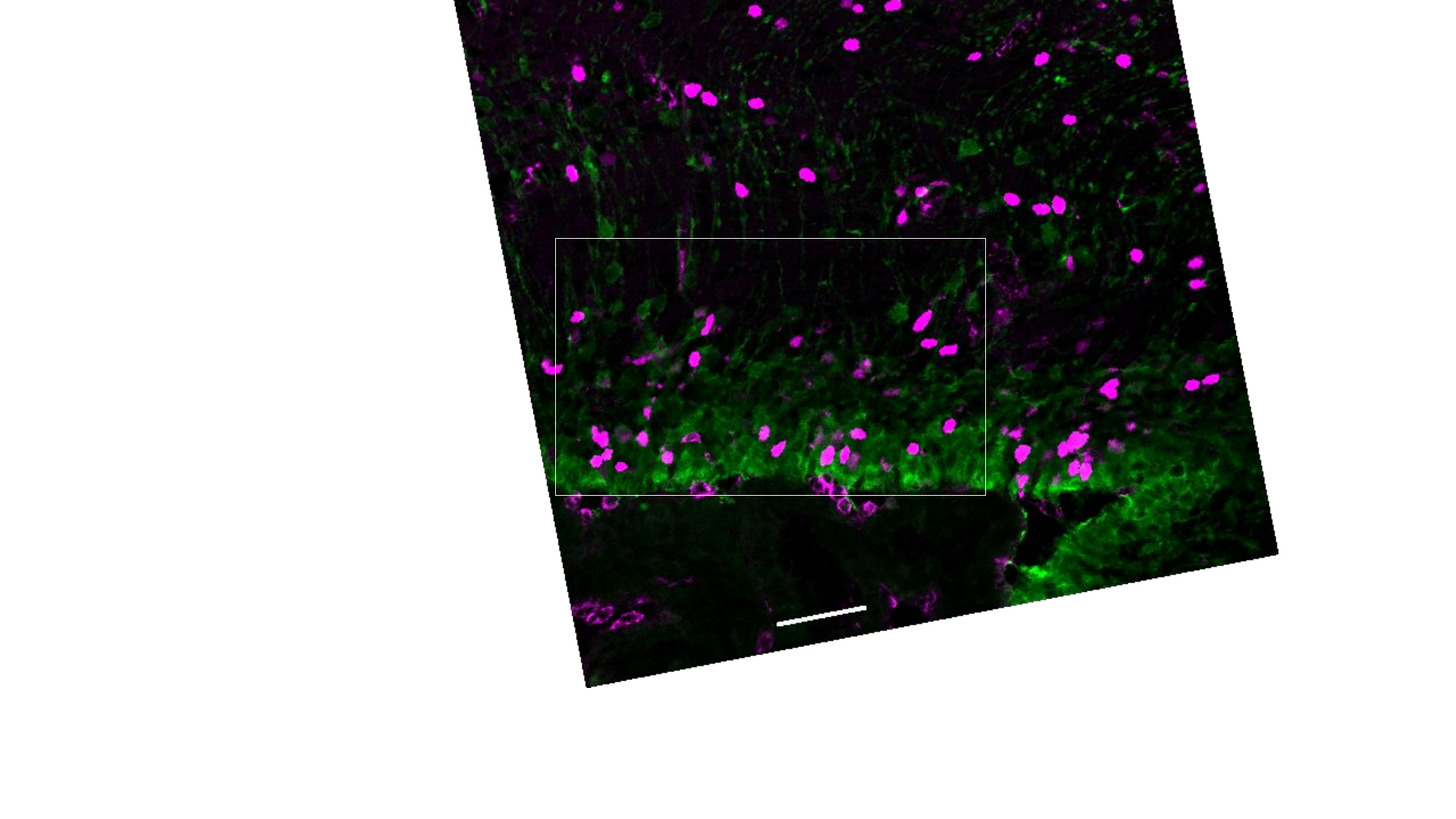

## Slide 27
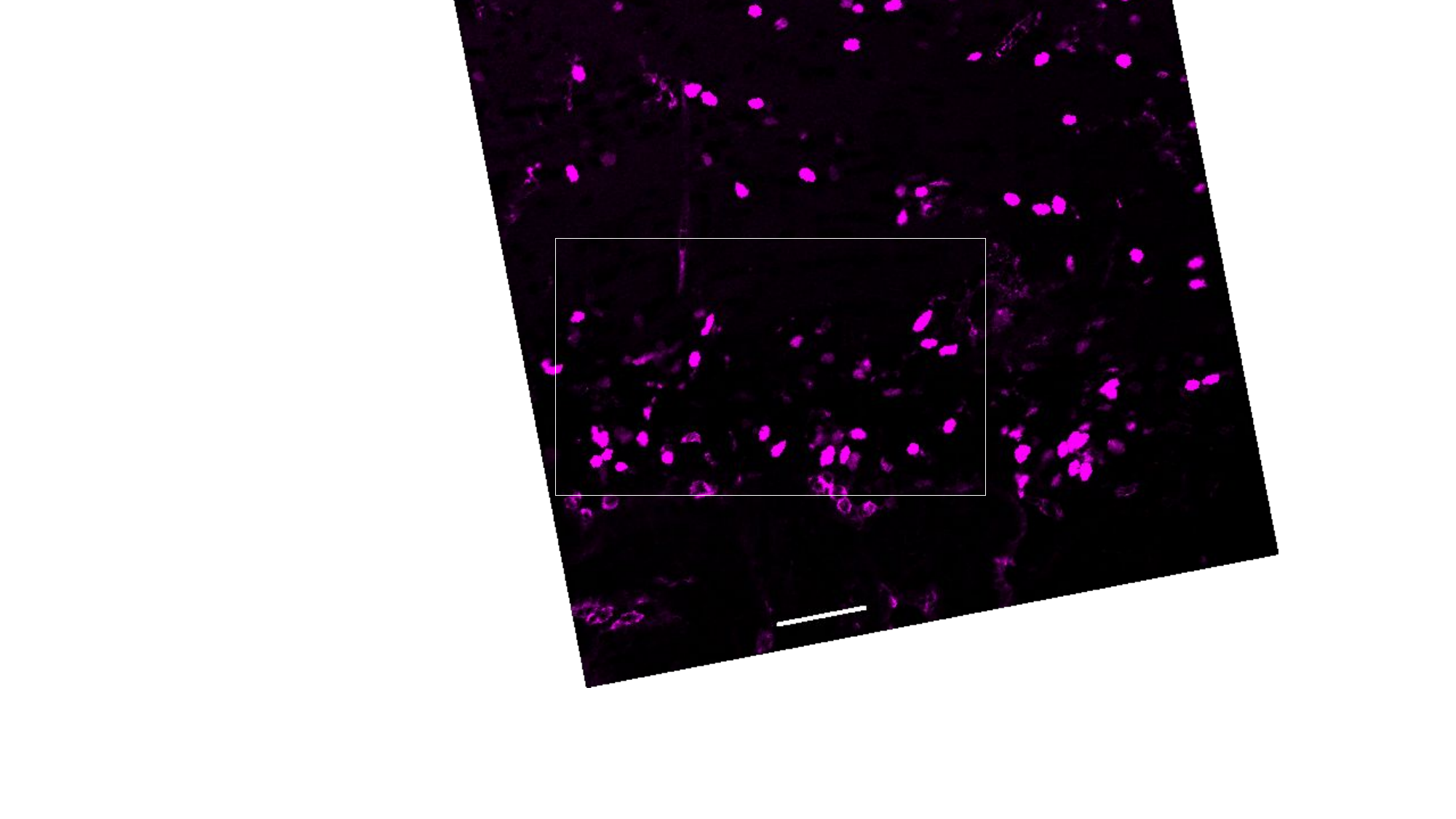

## Slide 28
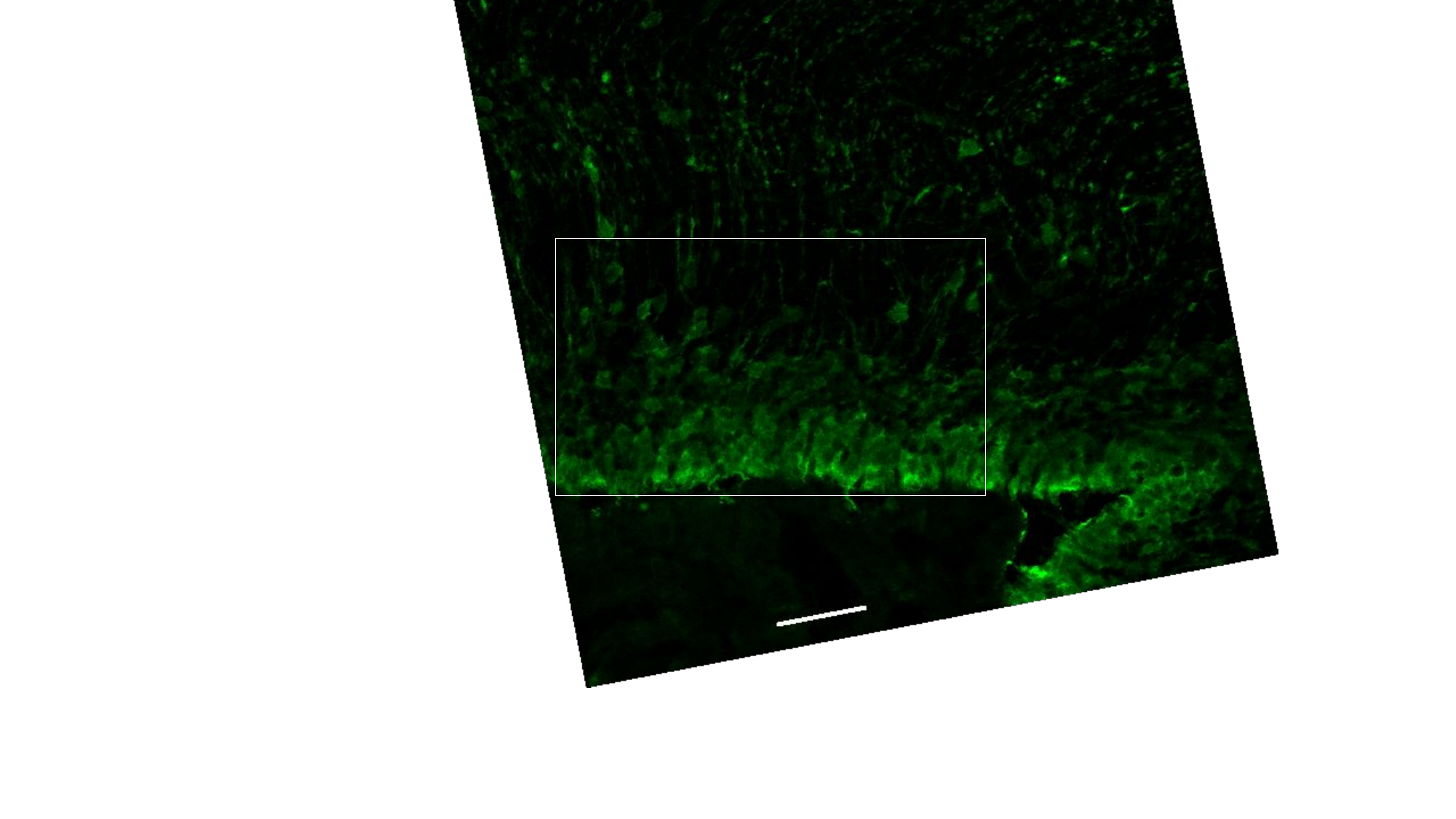

## Slide 29
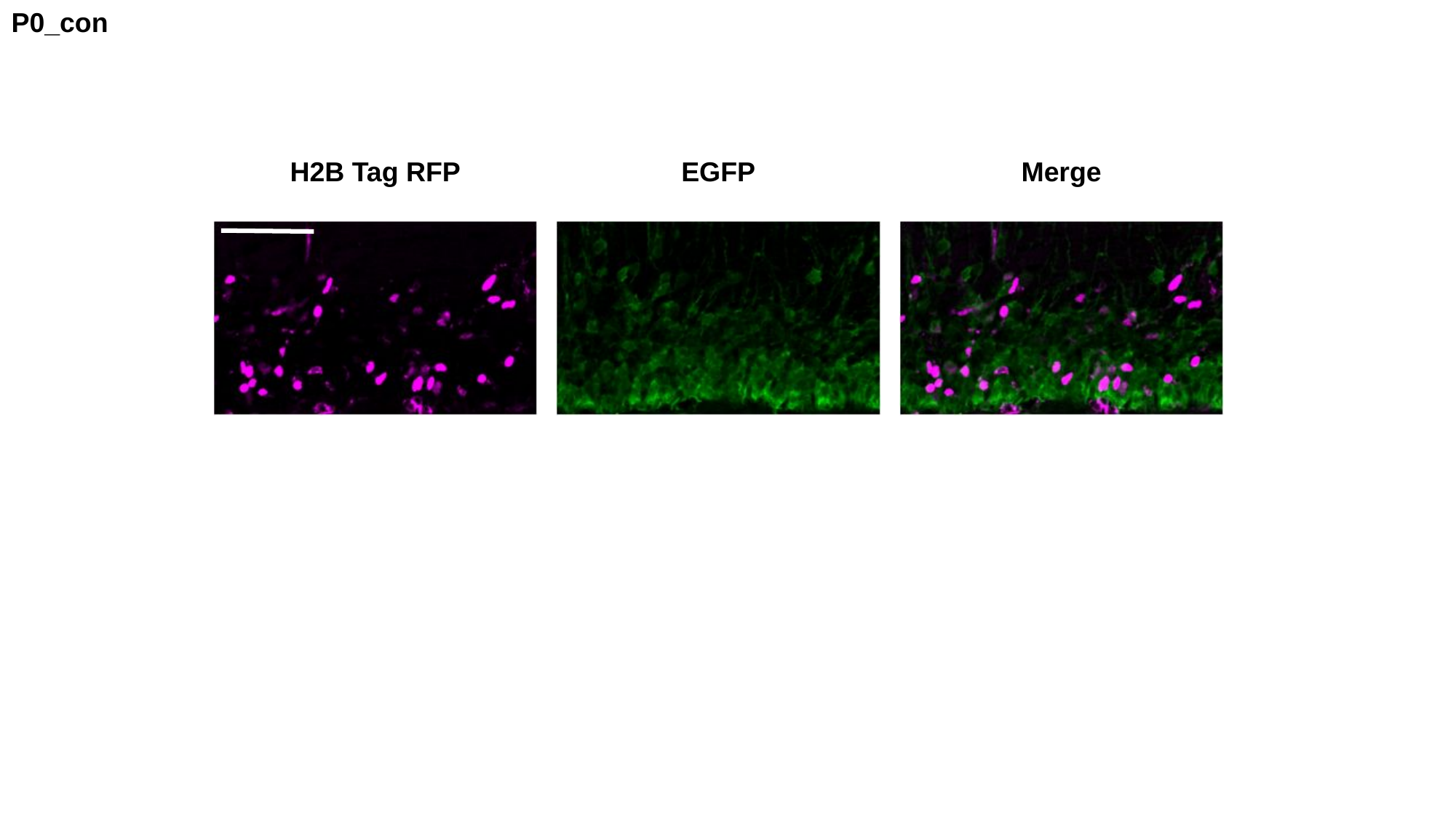

P0_con
H2B Tag RFP
EGFP
Merge

## Slide 30
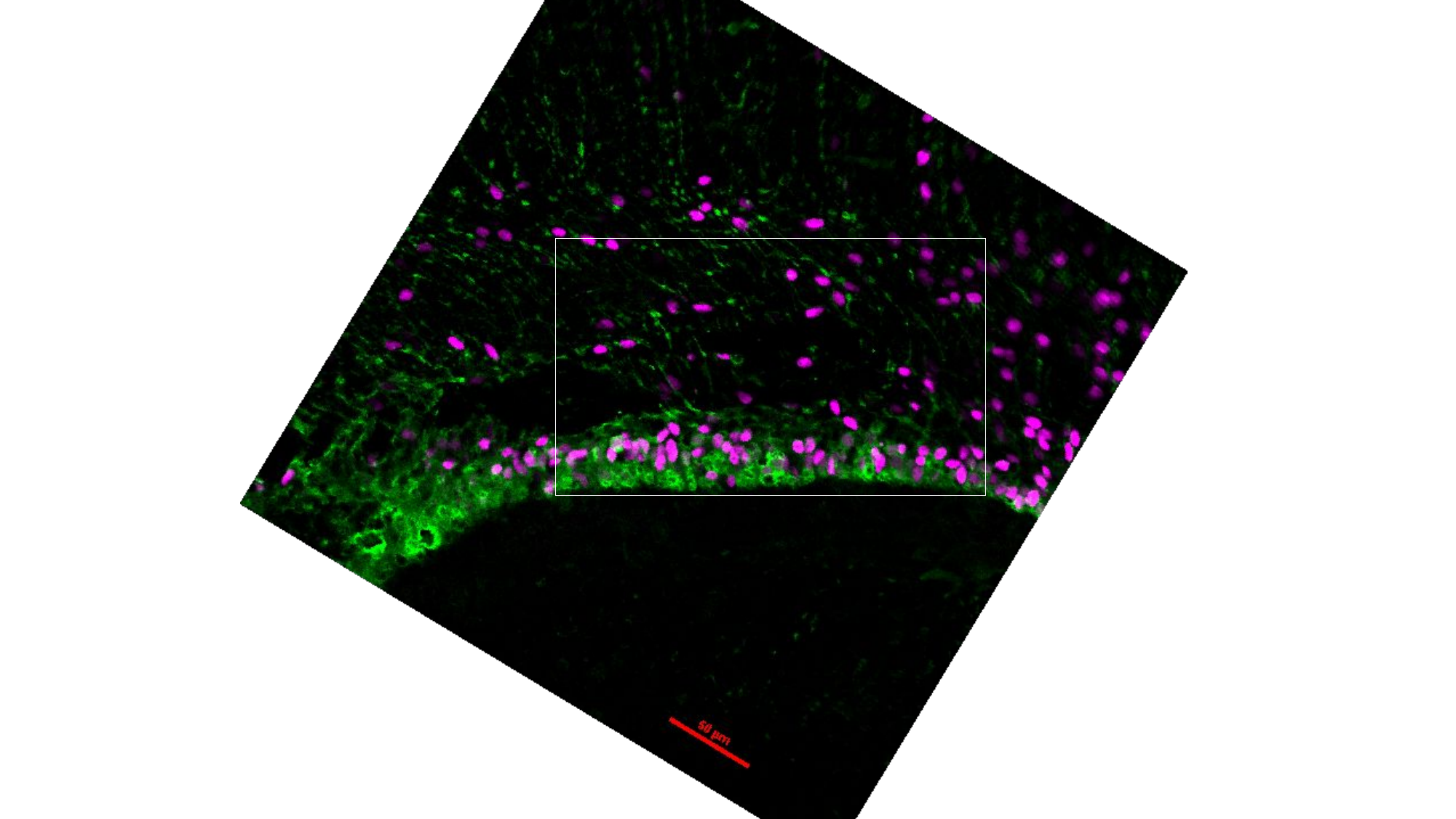

## Slide 31
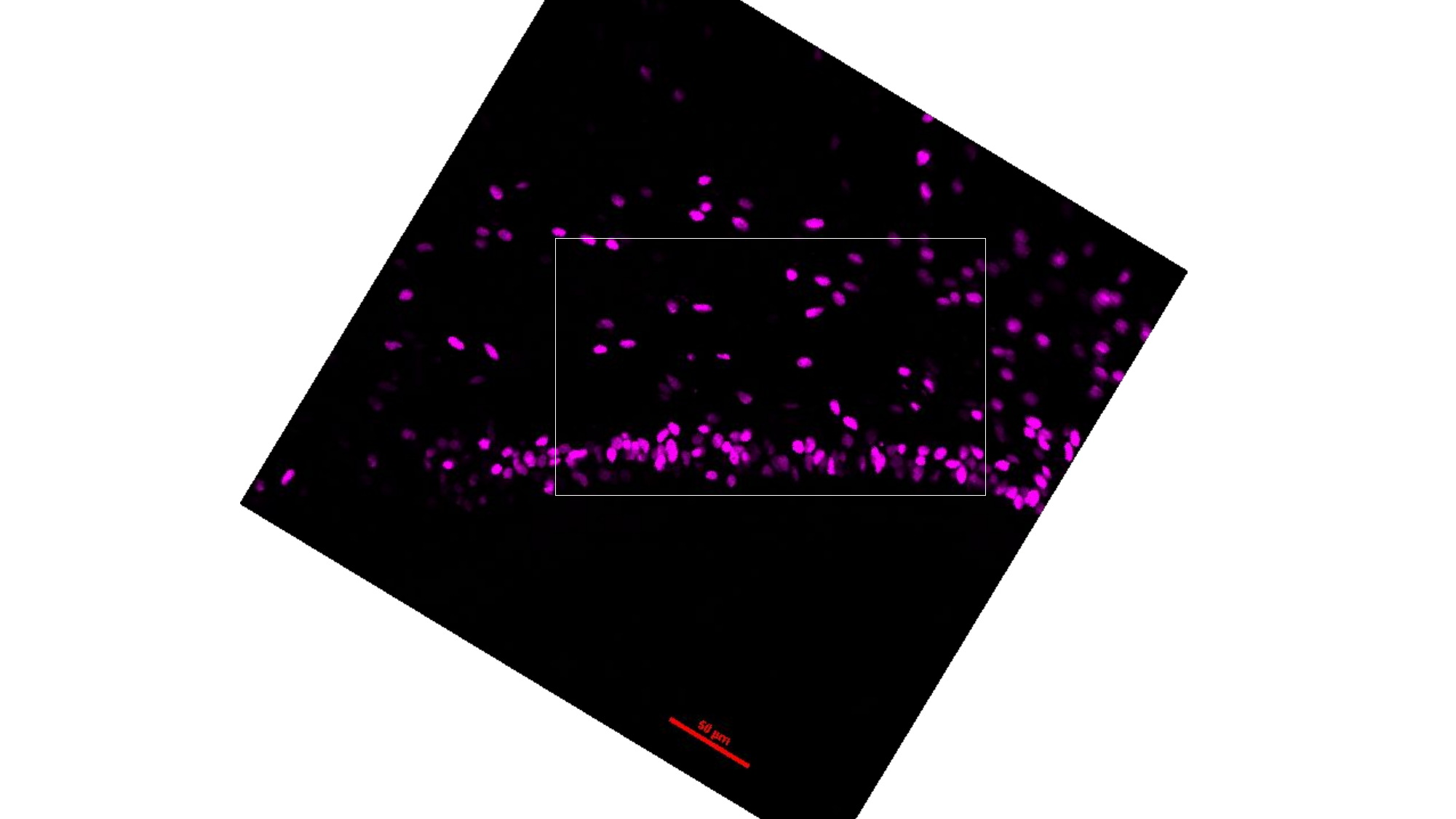

## Slide 32
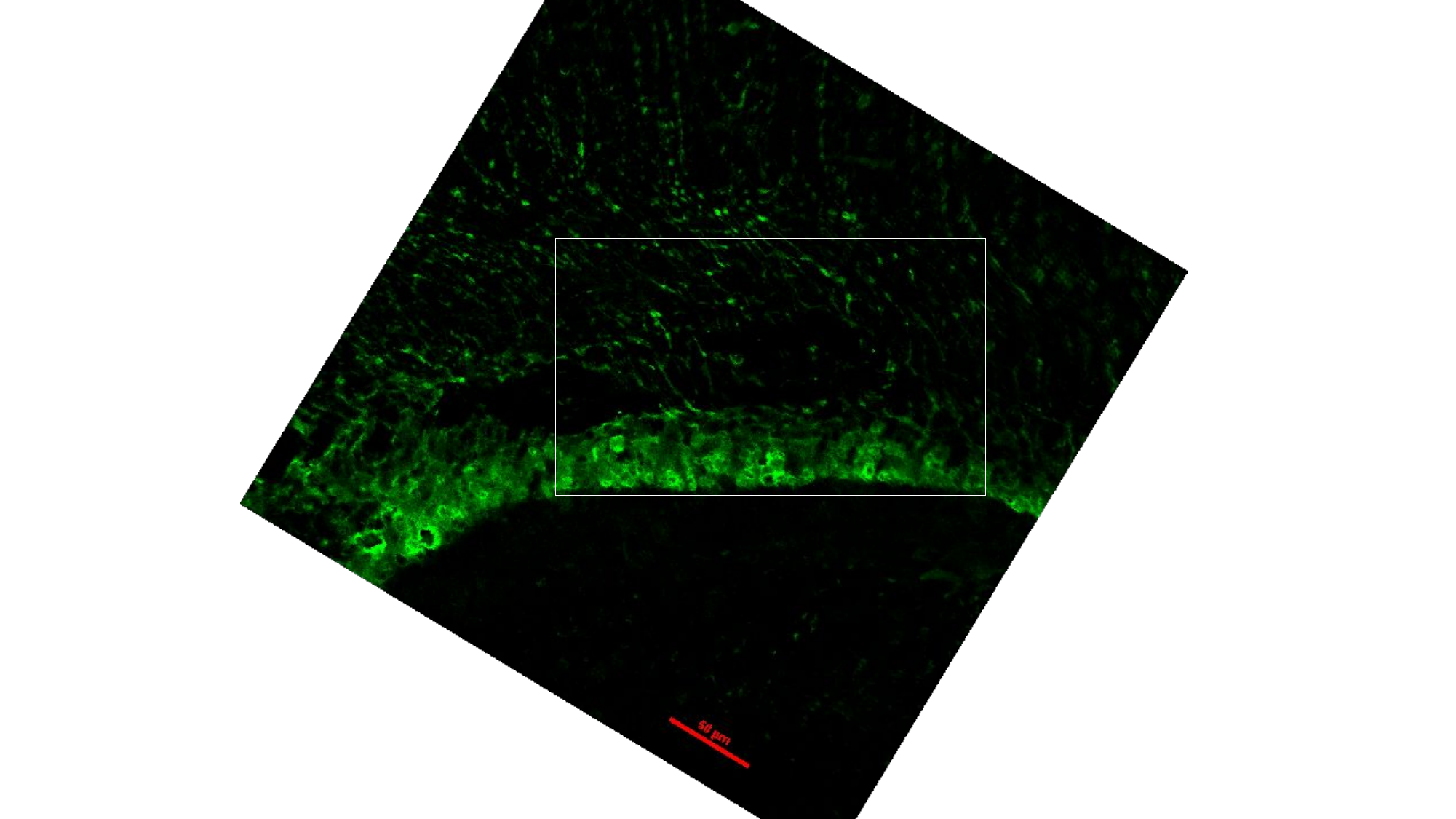

## Slide 33
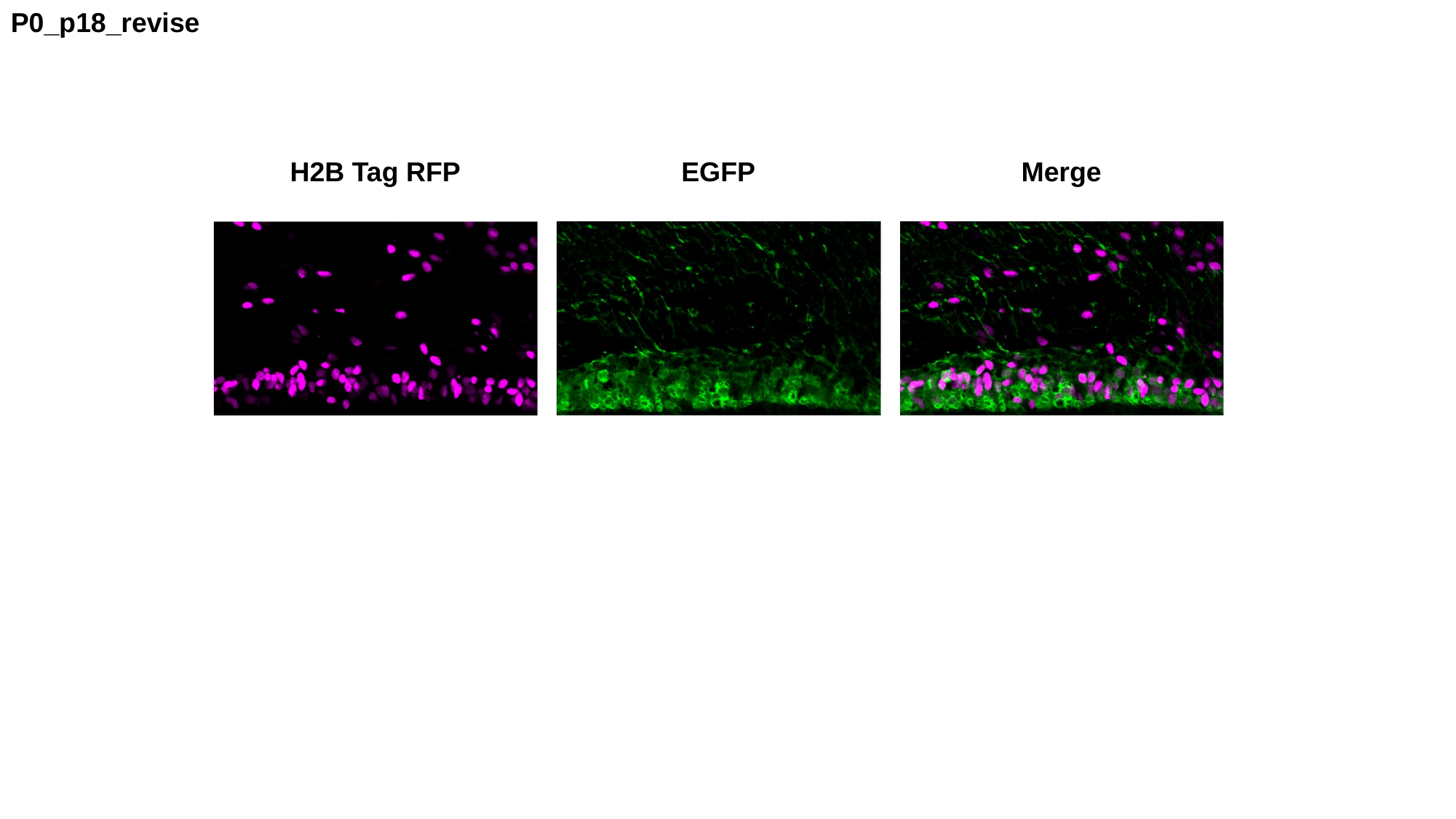

P0_p18_revise
H2B Tag RFP
EGFP
Merge
