# Supplementary material for: Cyclin-dependent kinase inhibitor p18 regulates lineage transitions of excitatory neurons, astrocytes, and interneurons in the mouse cortex
Source: EMBO J. 2024 Dec 12;44(2):382–412. doi: 10.1038/s44318-024-00325-9 (PMC11730326; doi:10.1038/s44318-024-00325-9)
Supplement: Supplementary file 4 — Source data Fig. 2 [file 44318_2024_325_MOESM4_ESM.zip › 2H.pptx]

## Slide 1
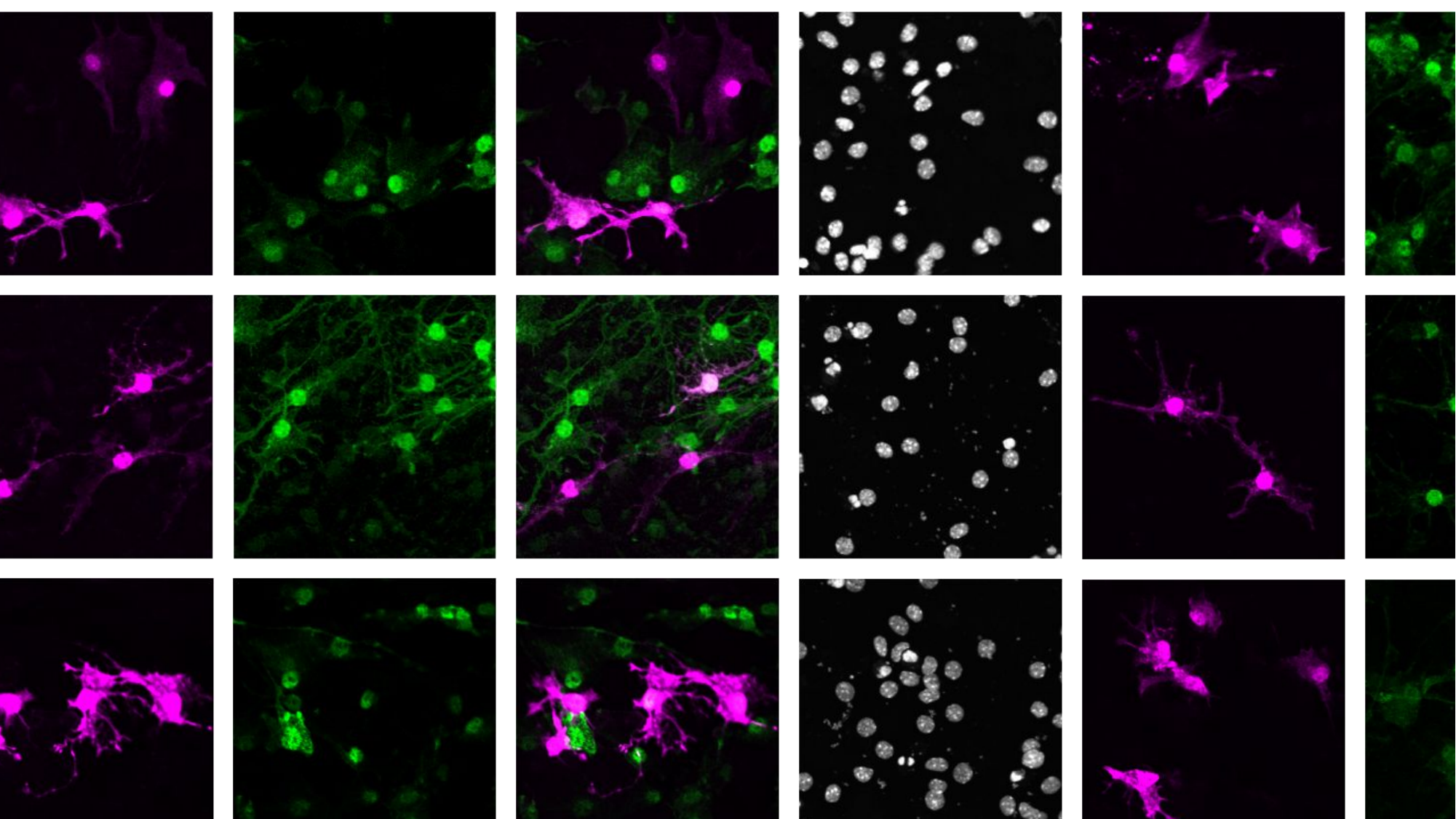

FBS
BMP4
LIF
mKO2 (Control)
p18-2A-mKO2
DAPI
mKO2
Aldh1l1-EGFP
EGFP / mKO2
DAPI
mKO2
Aldh1l1-EGFP
EGFP / mKO2
+
+
+
-
-
-
-
+
+

## Slide 2
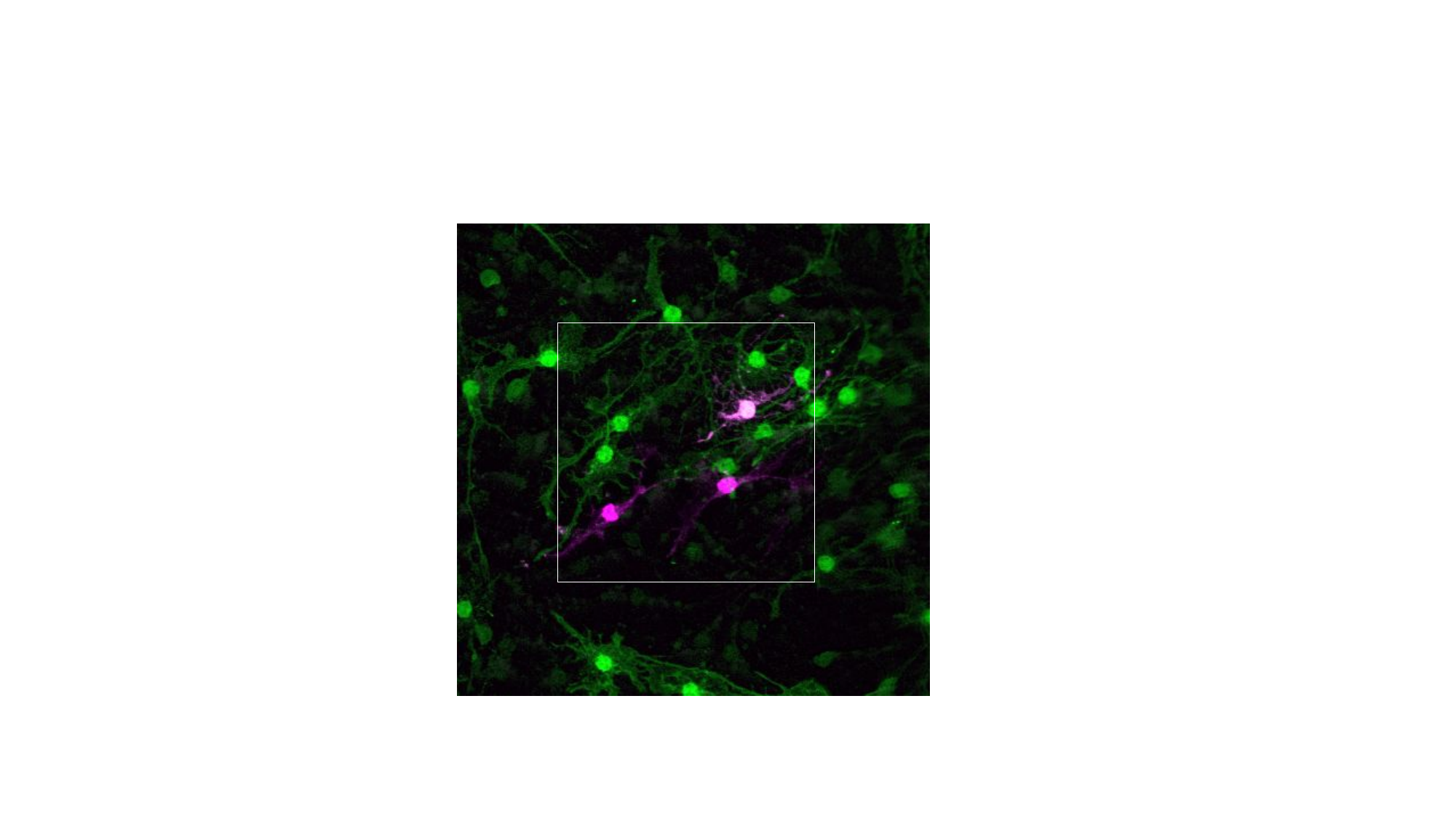

## Slide 3
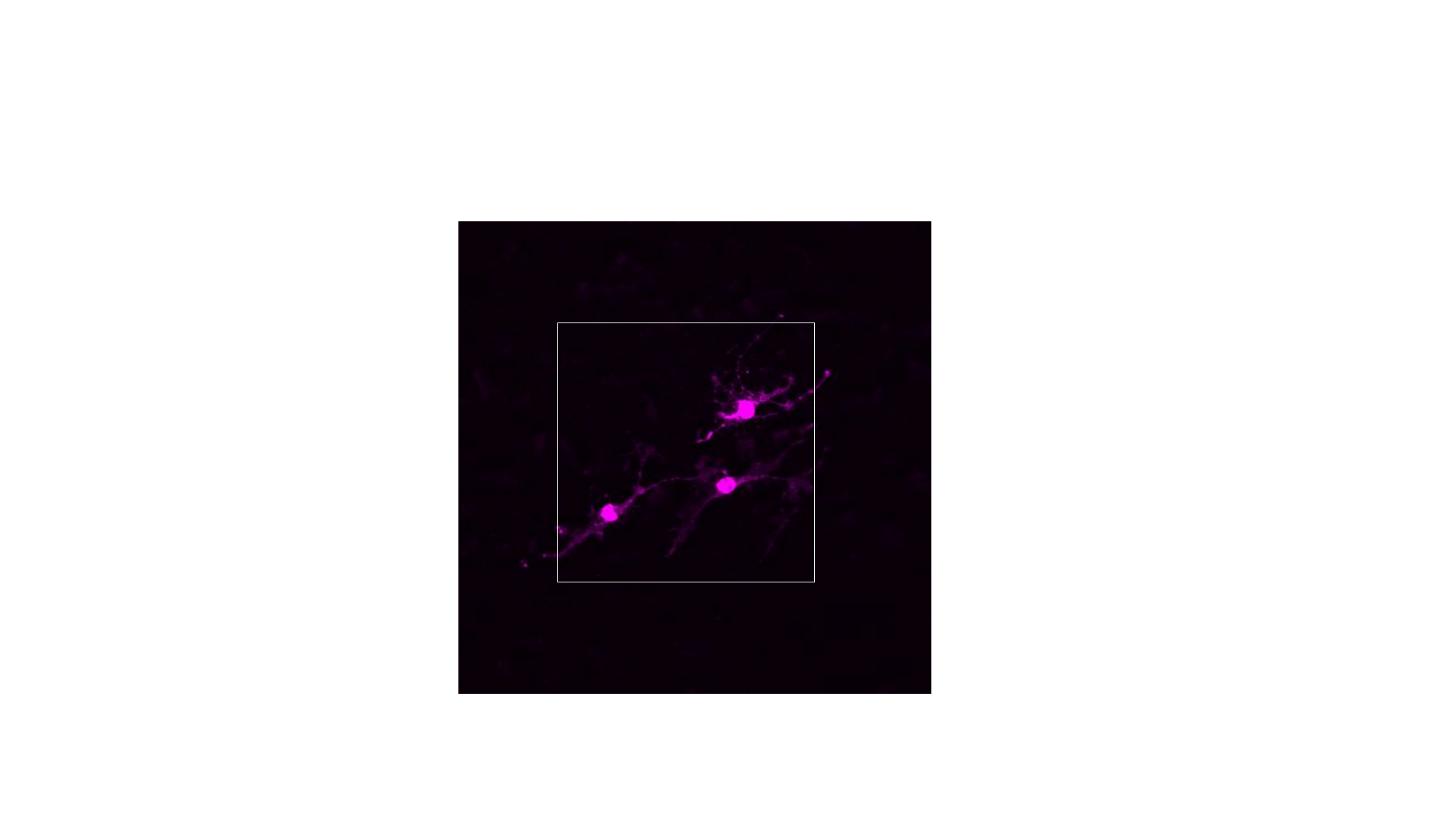

## Slide 4
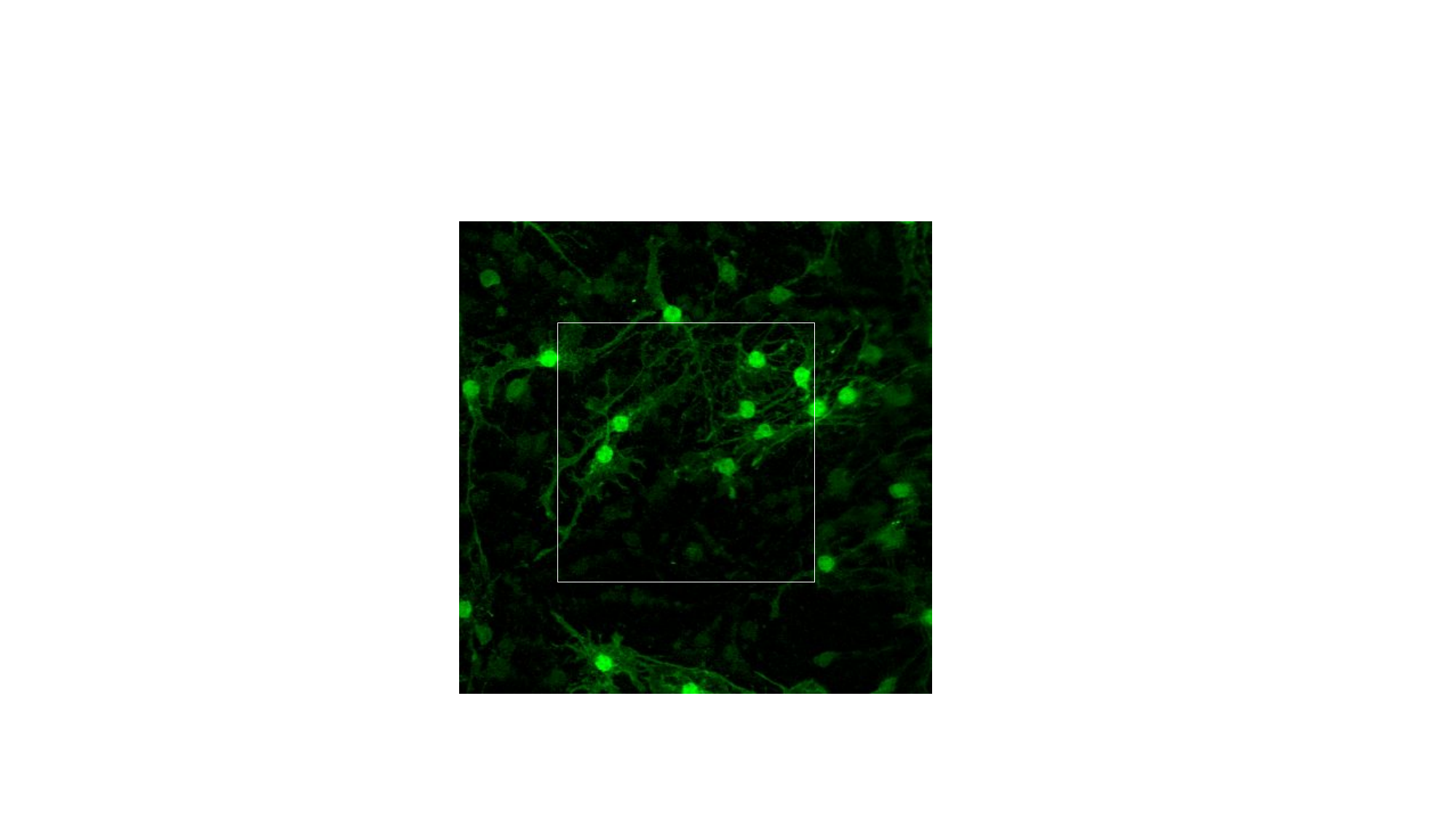

## Slide 5
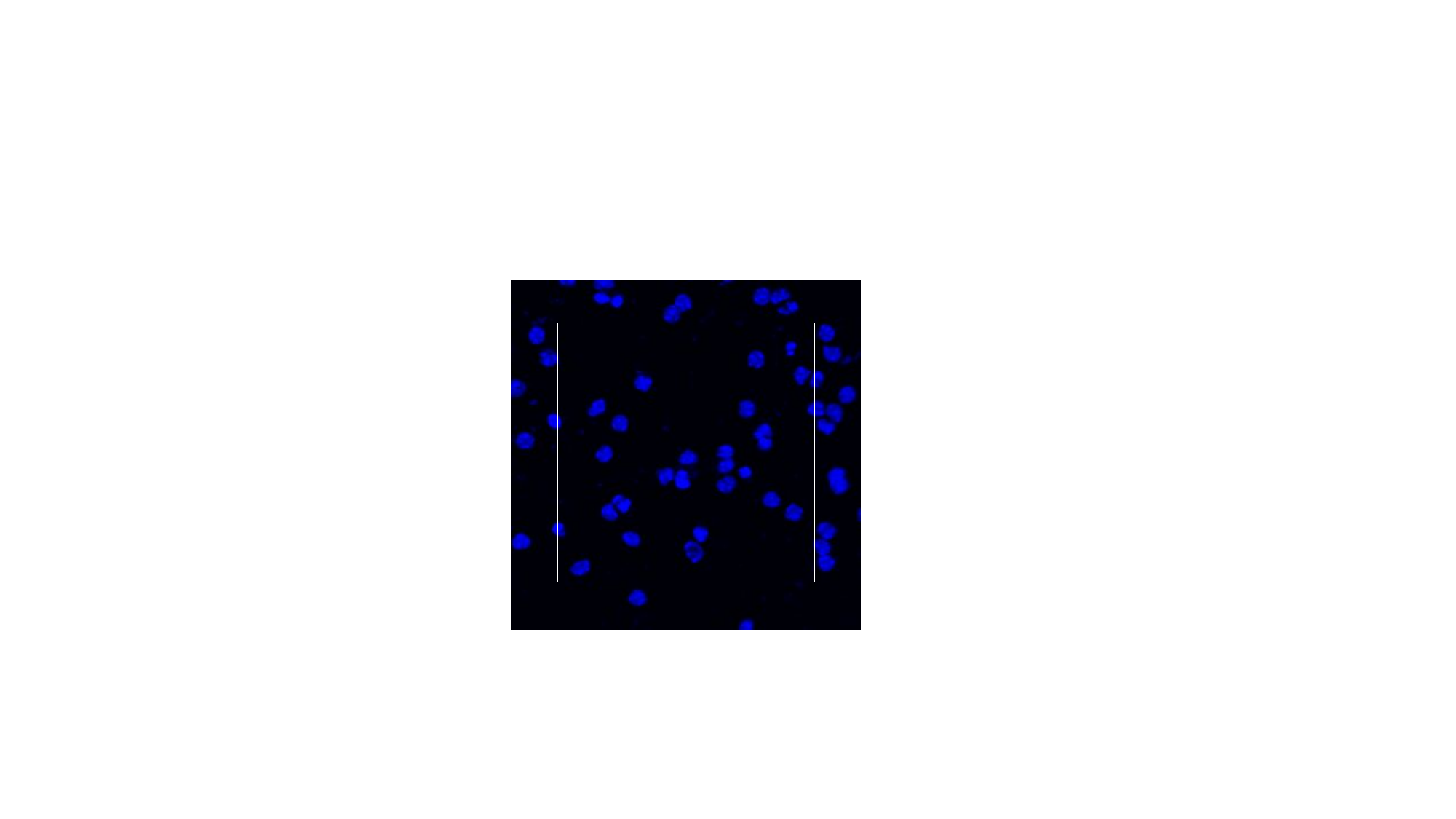

## Slide 6
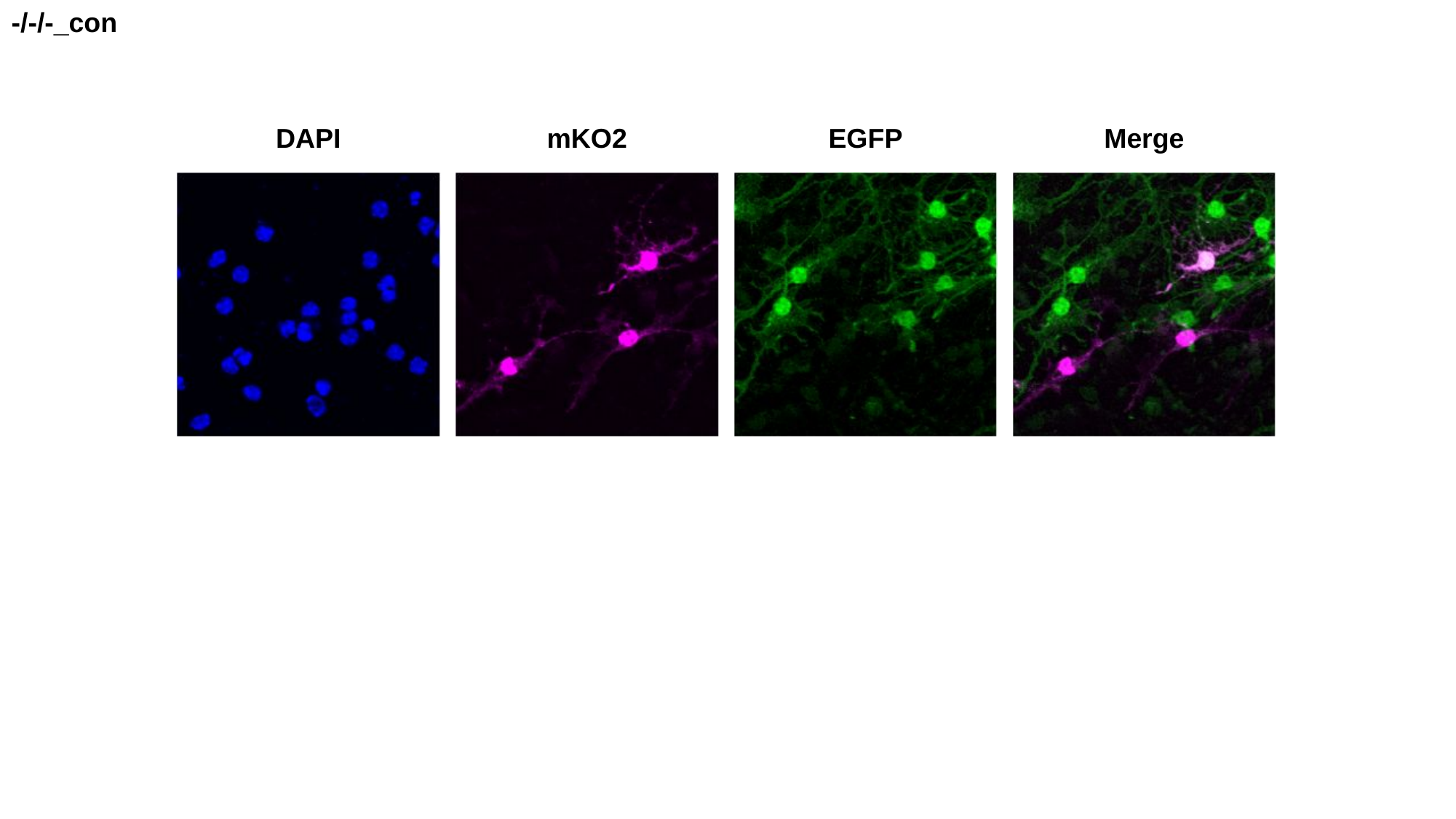

-/-/-_con
DAPI
mKO2
EGFP
Merge

## Slide 7
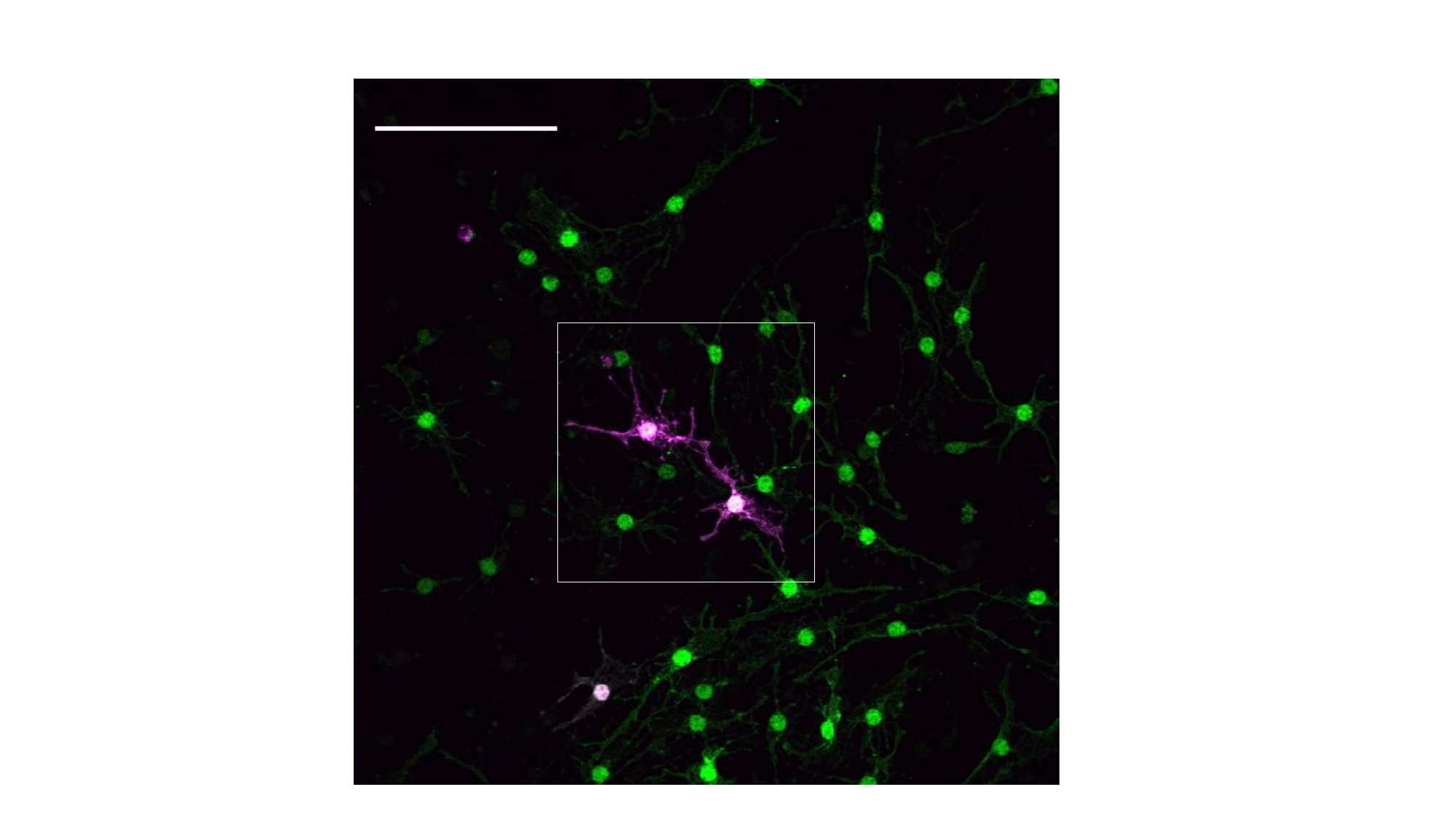

## Slide 8
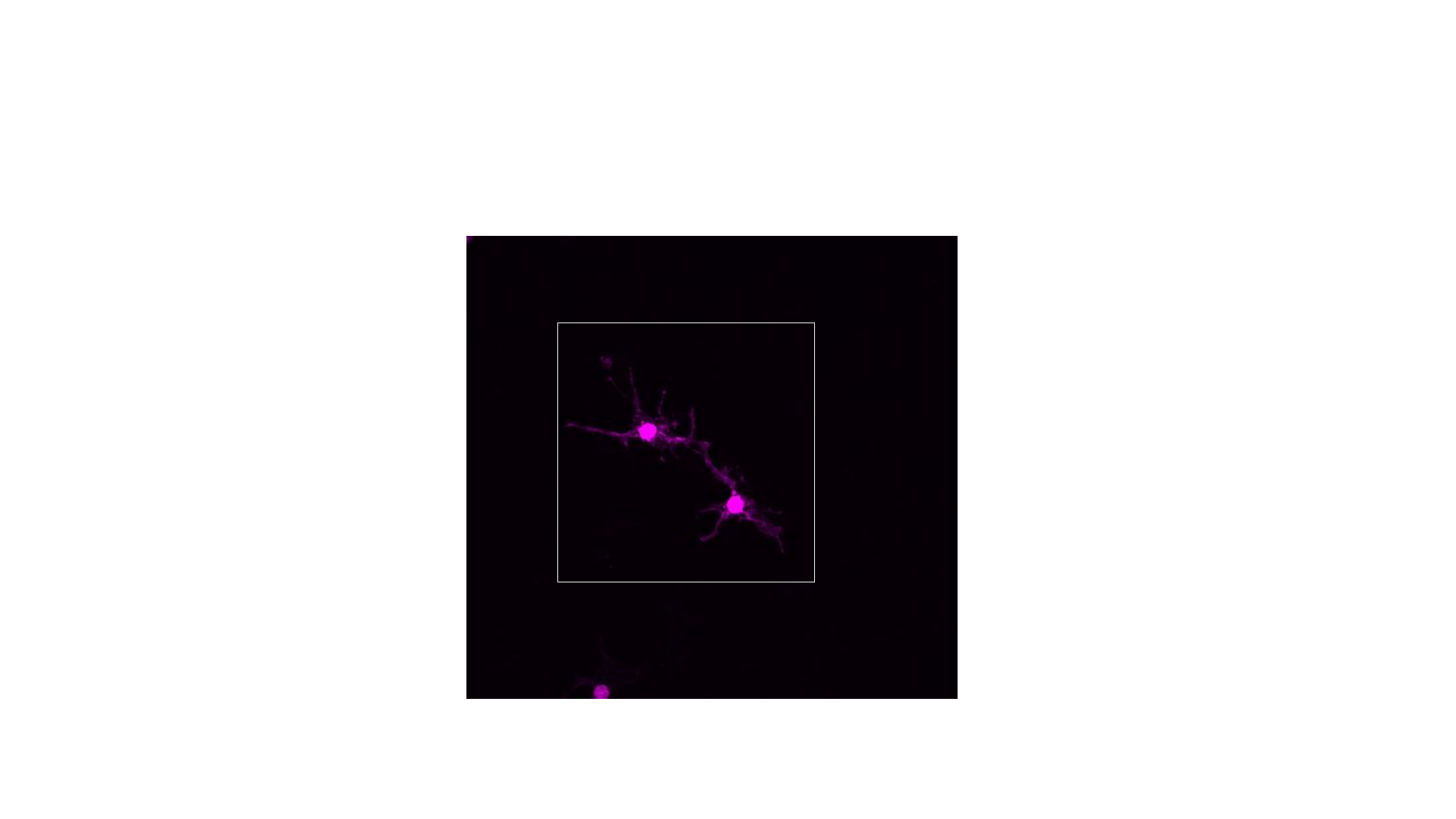

## Slide 9
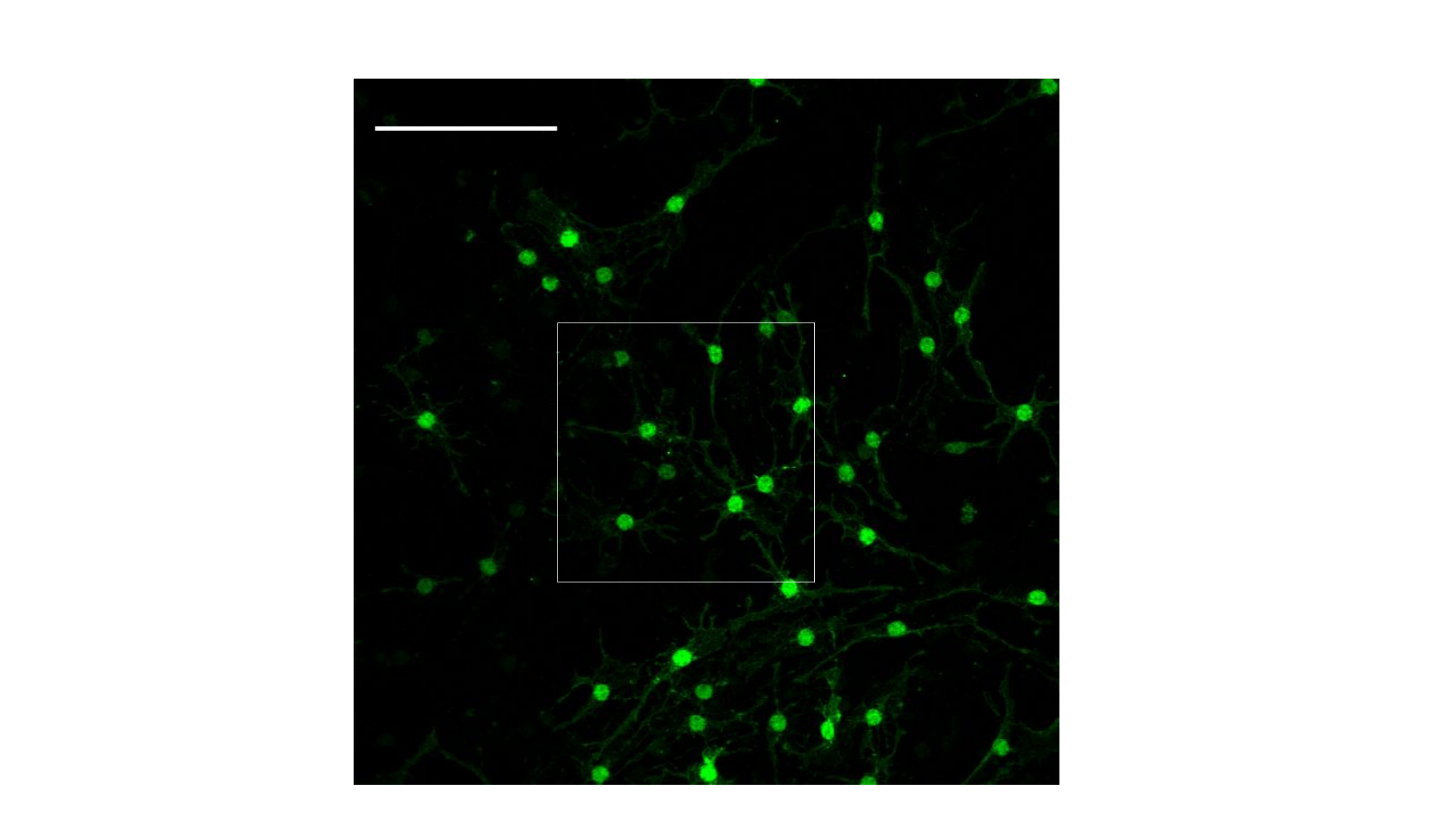

## Slide 10
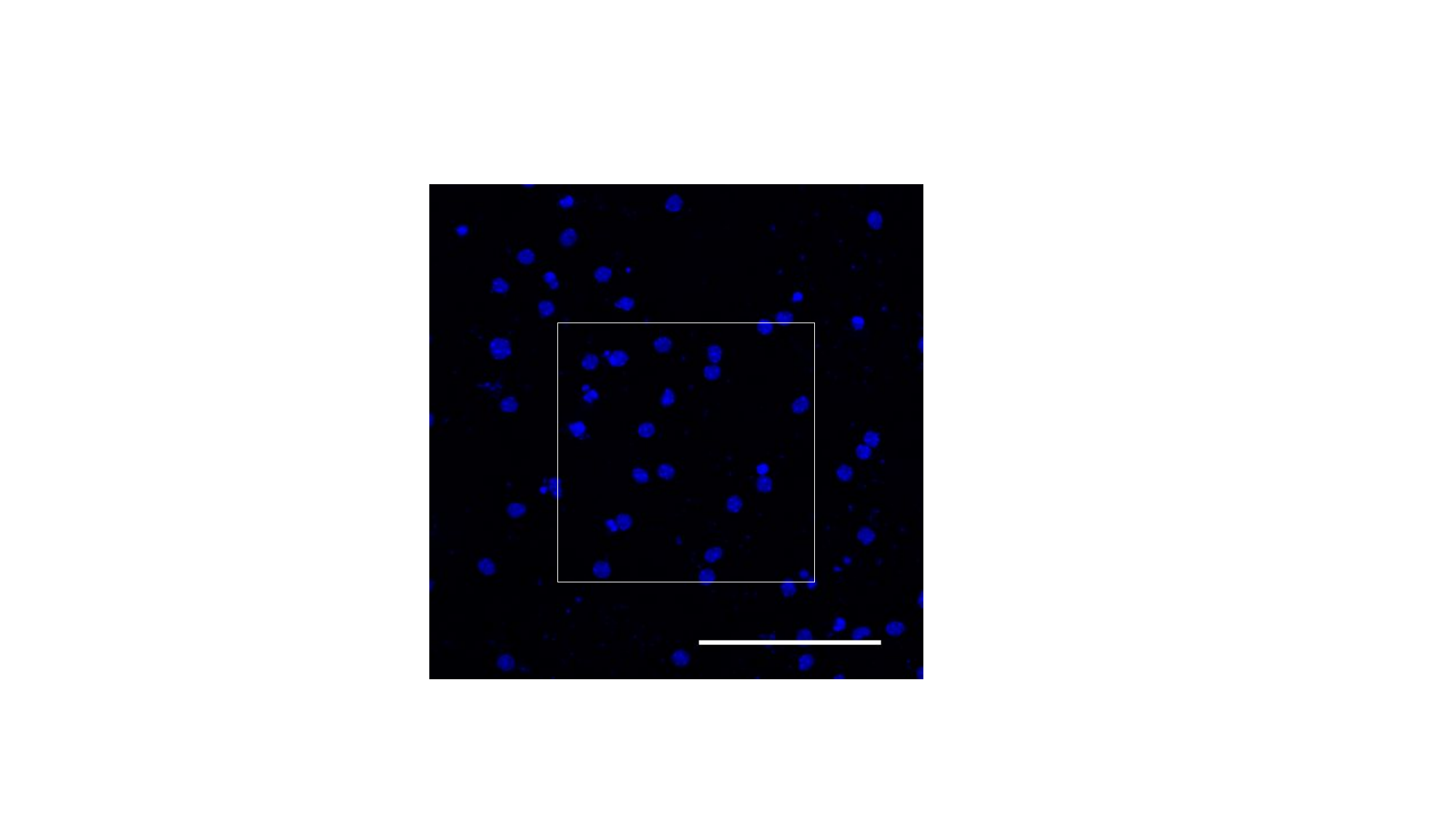

## Slide 11
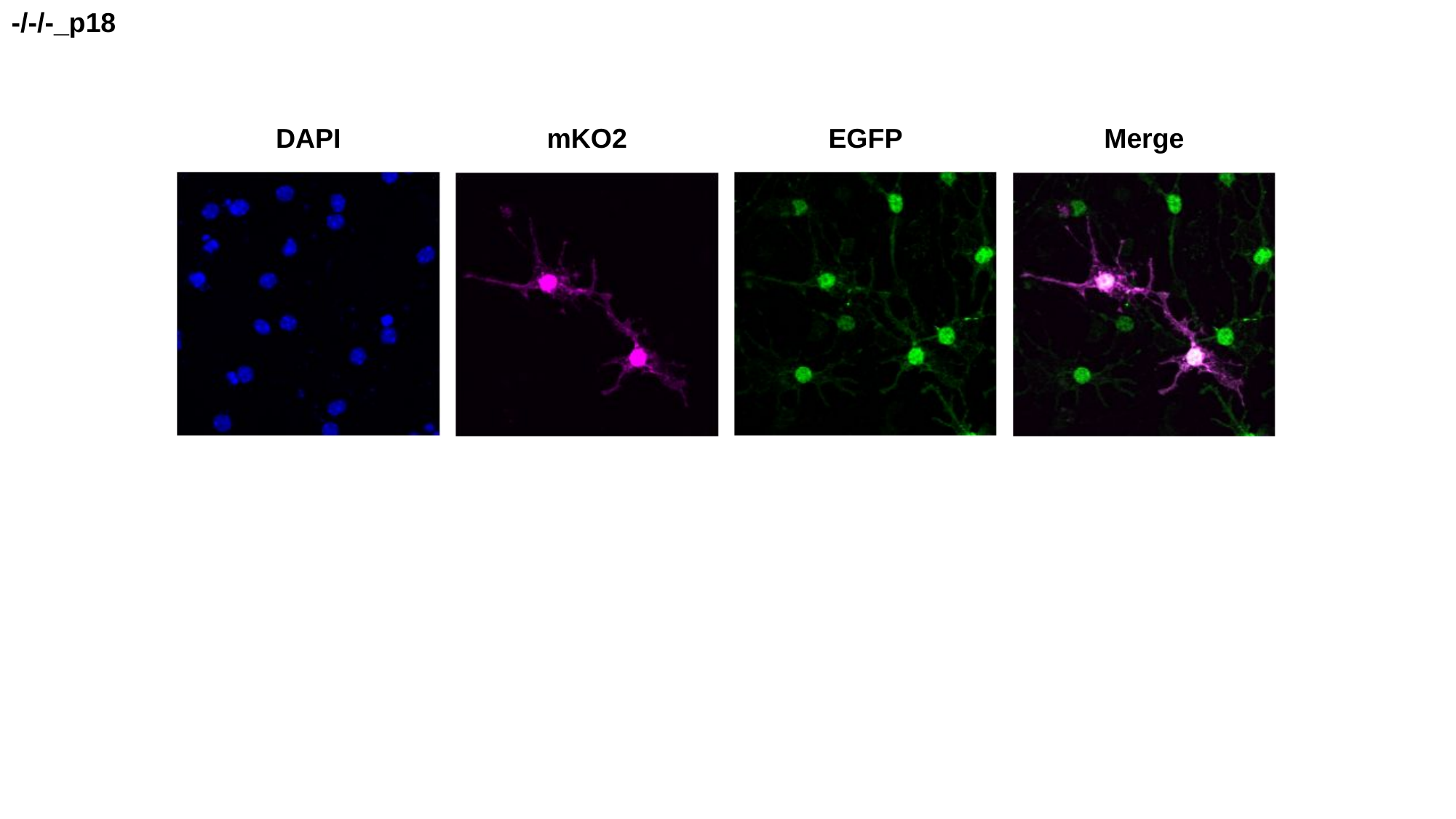

-/-/-_p18
DAPI
mKO2
EGFP
Merge

## Slide 12
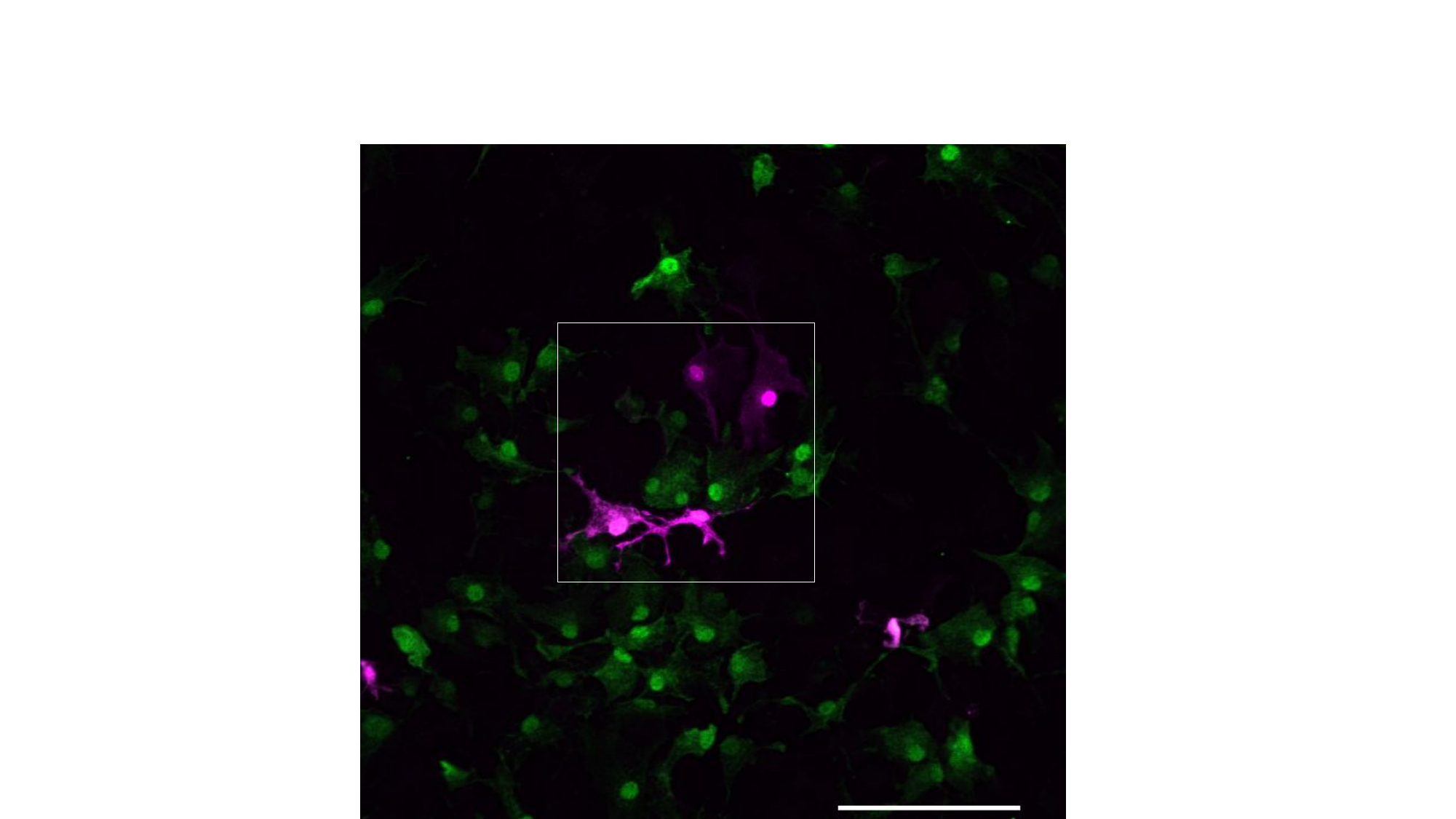

## Slide 13
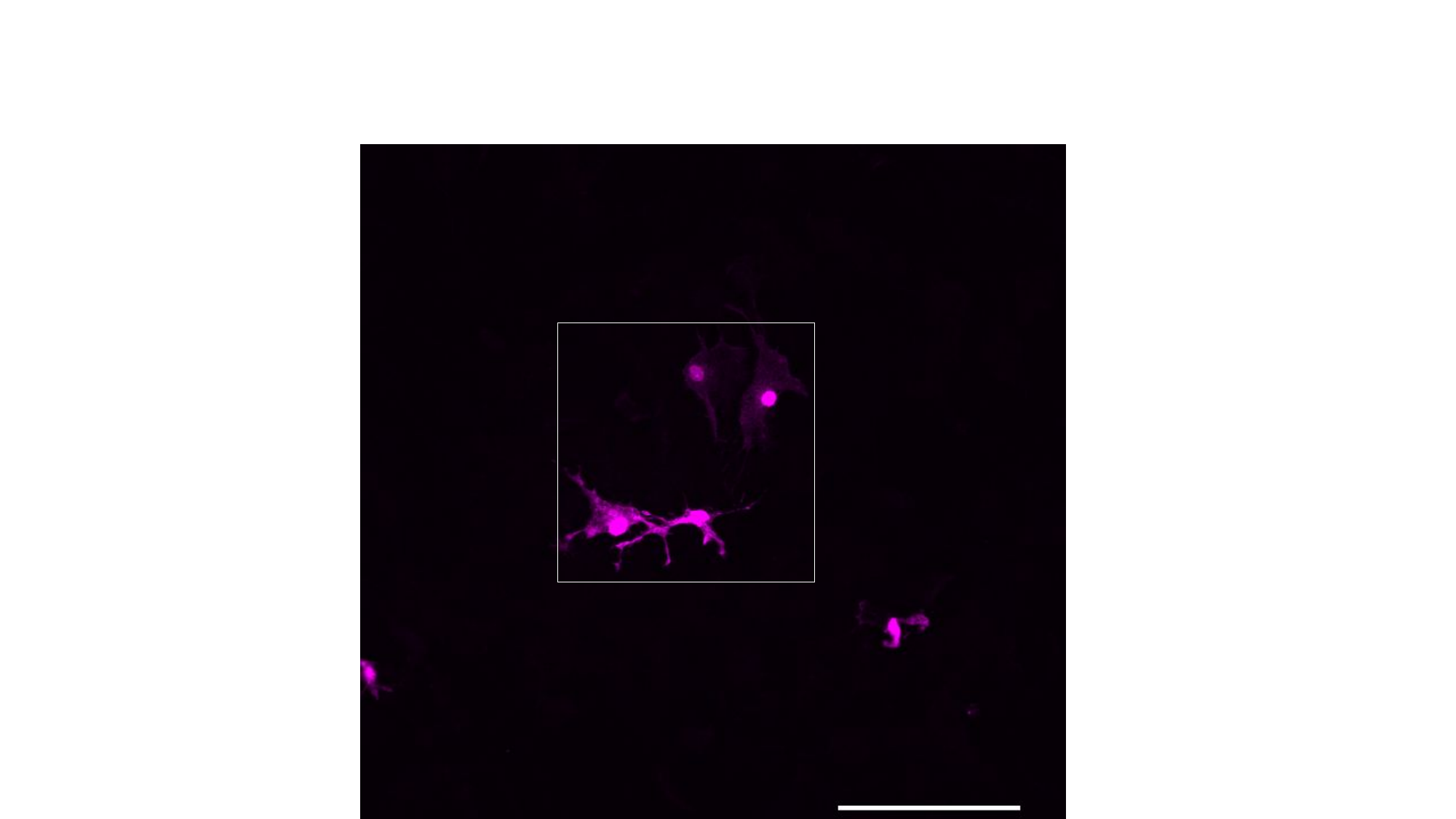

## Slide 14
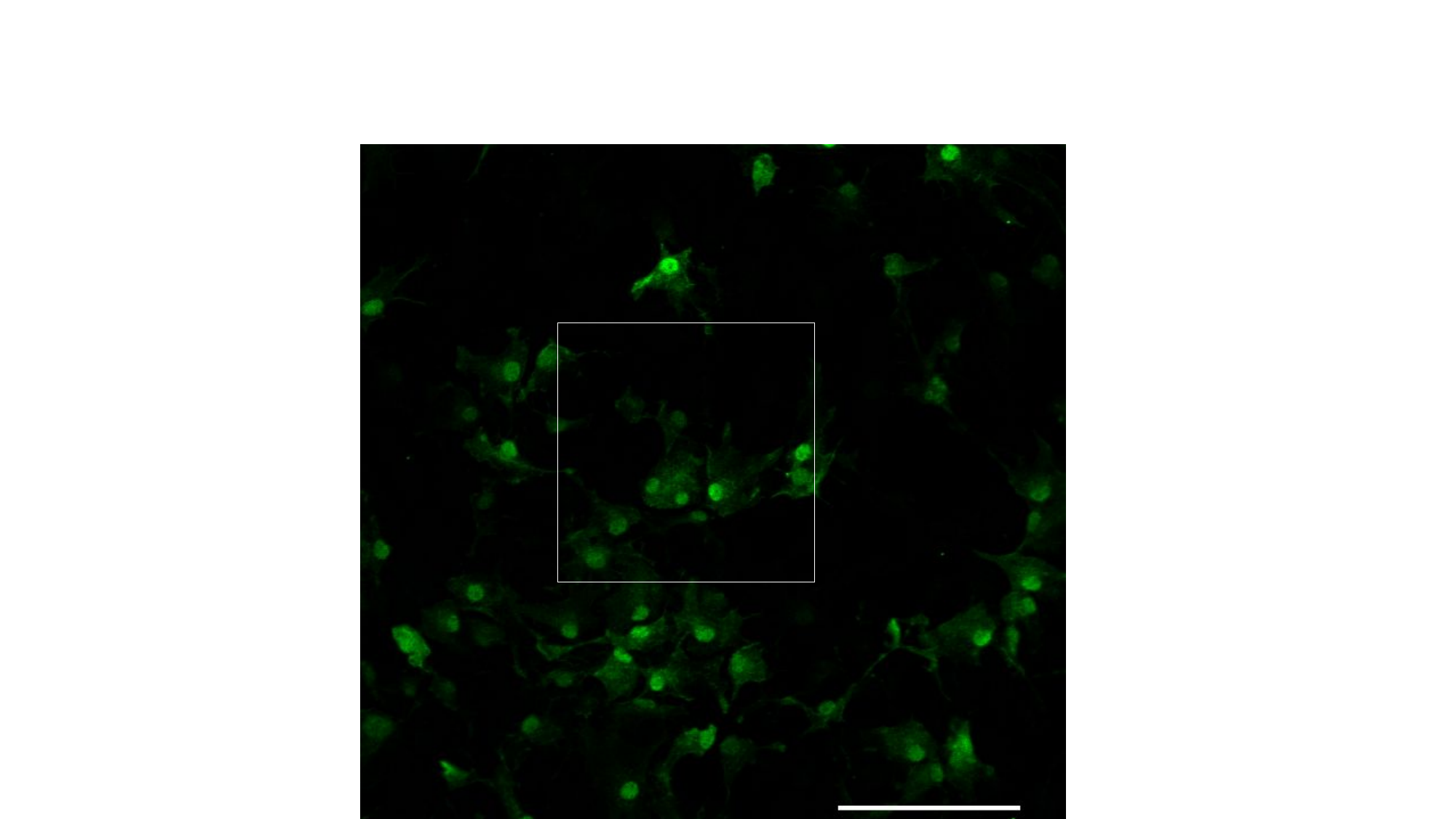

## Slide 15
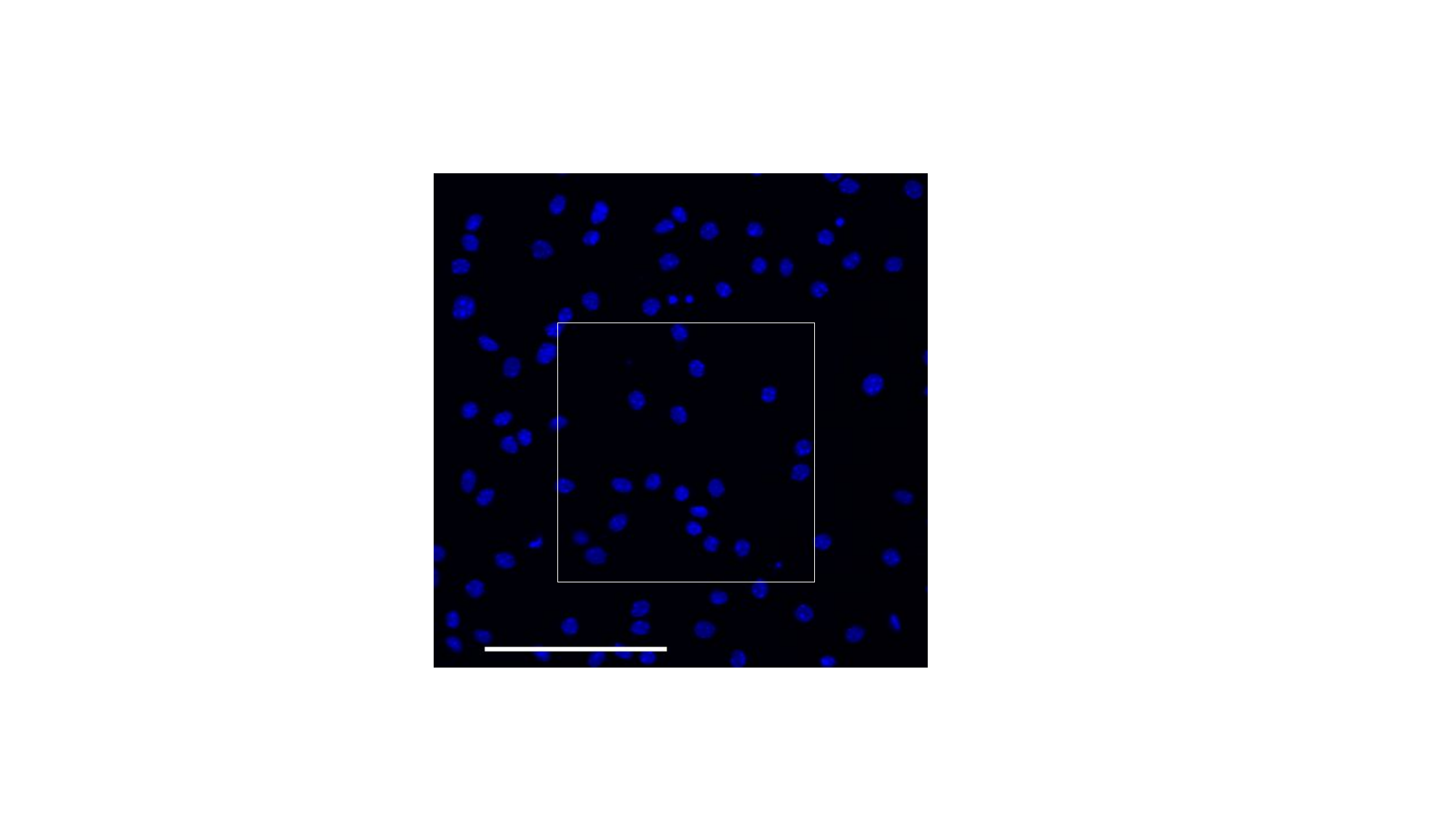

## Slide 16
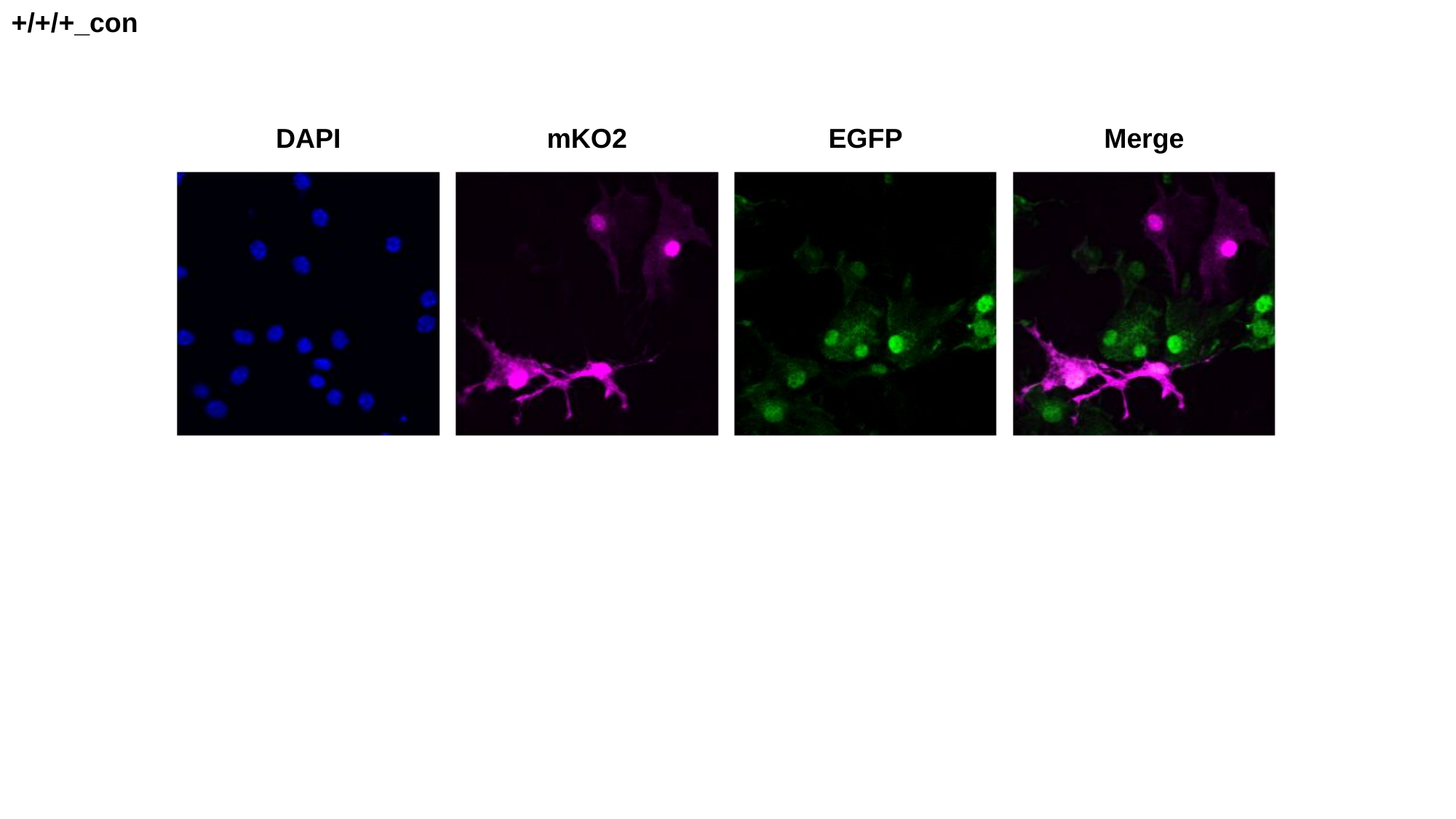

+/+/+_con
DAPI
mKO2
EGFP
Merge

## Slide 17
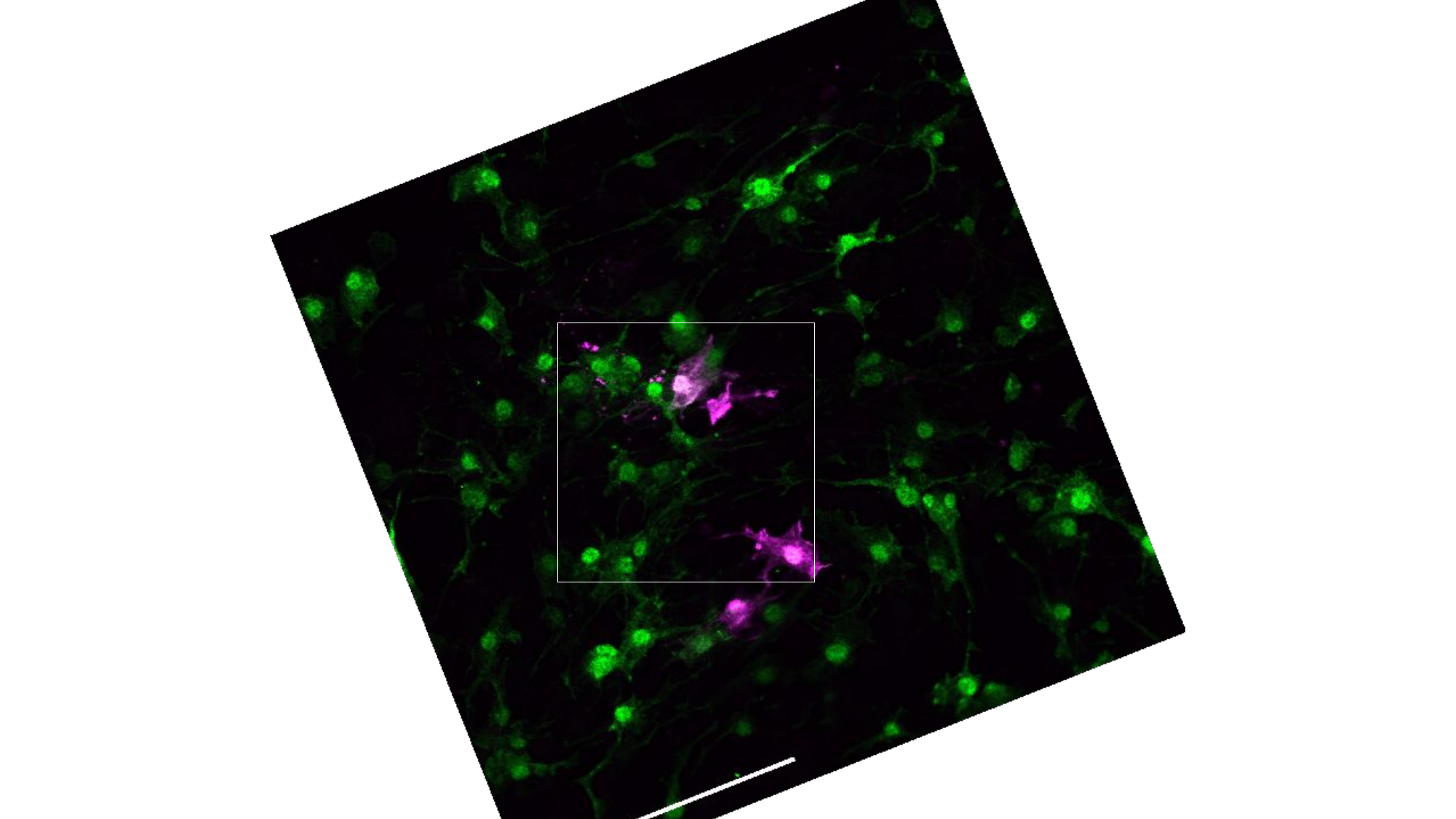

## Slide 18
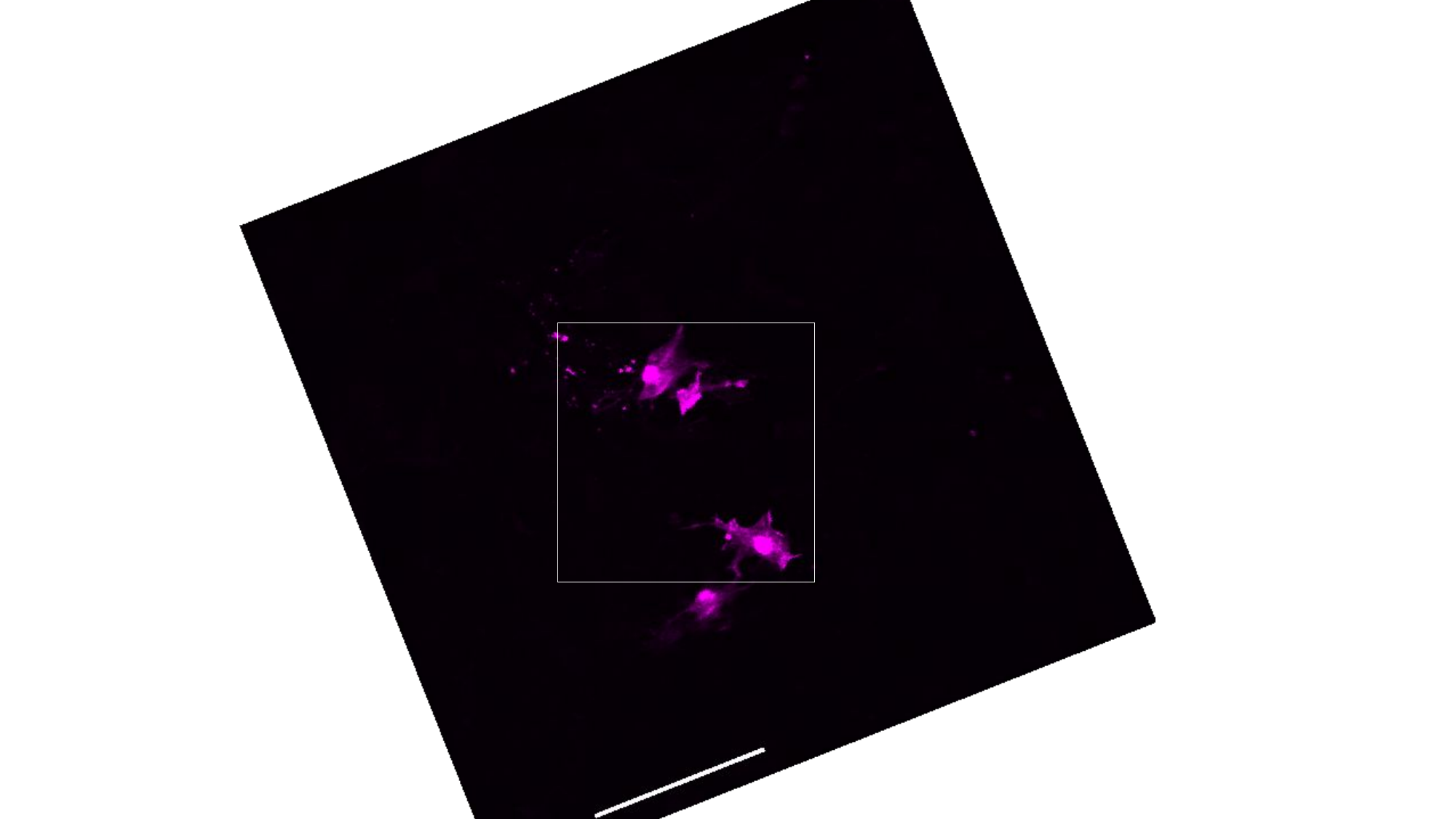

## Slide 19
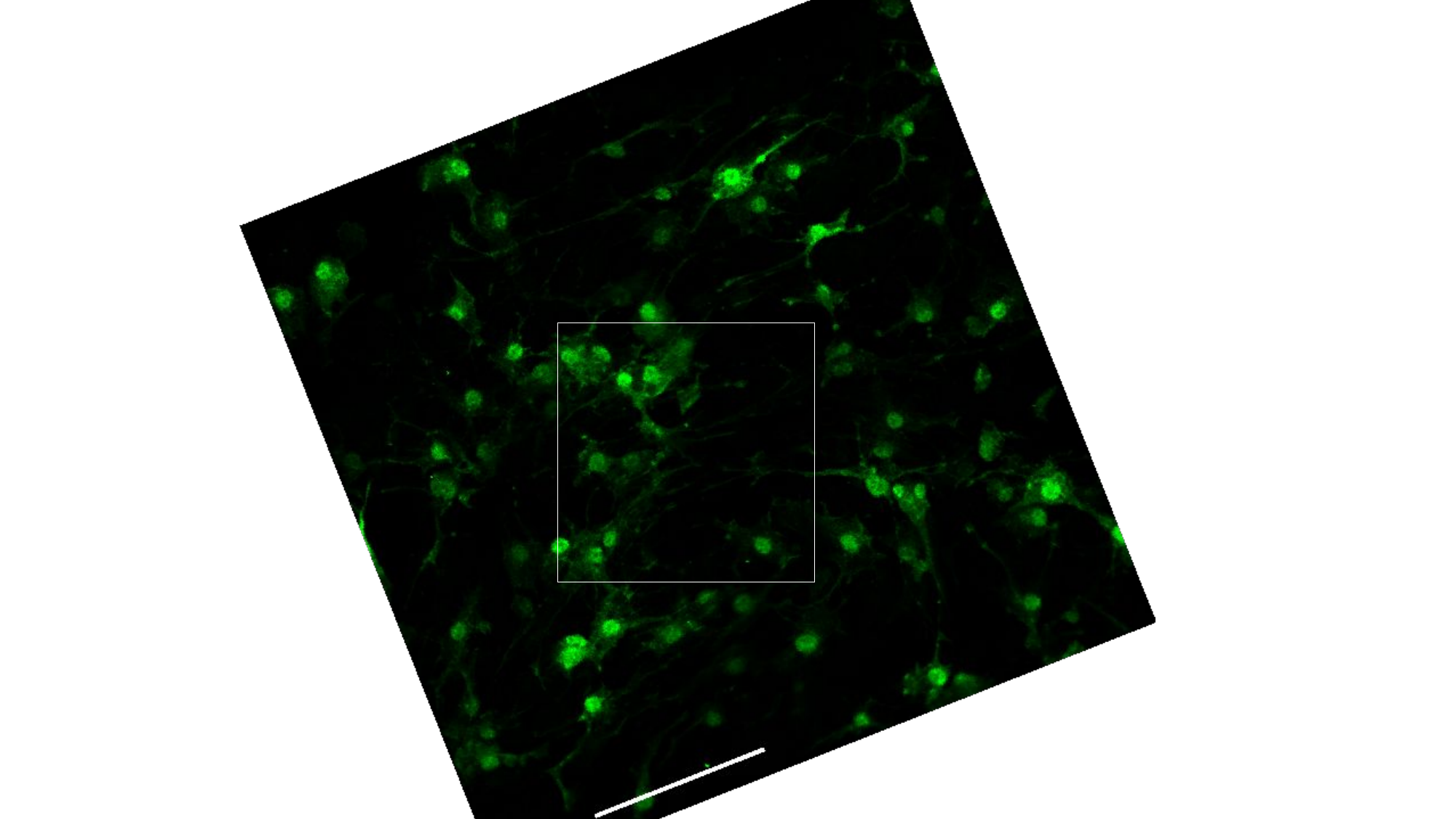

## Slide 20
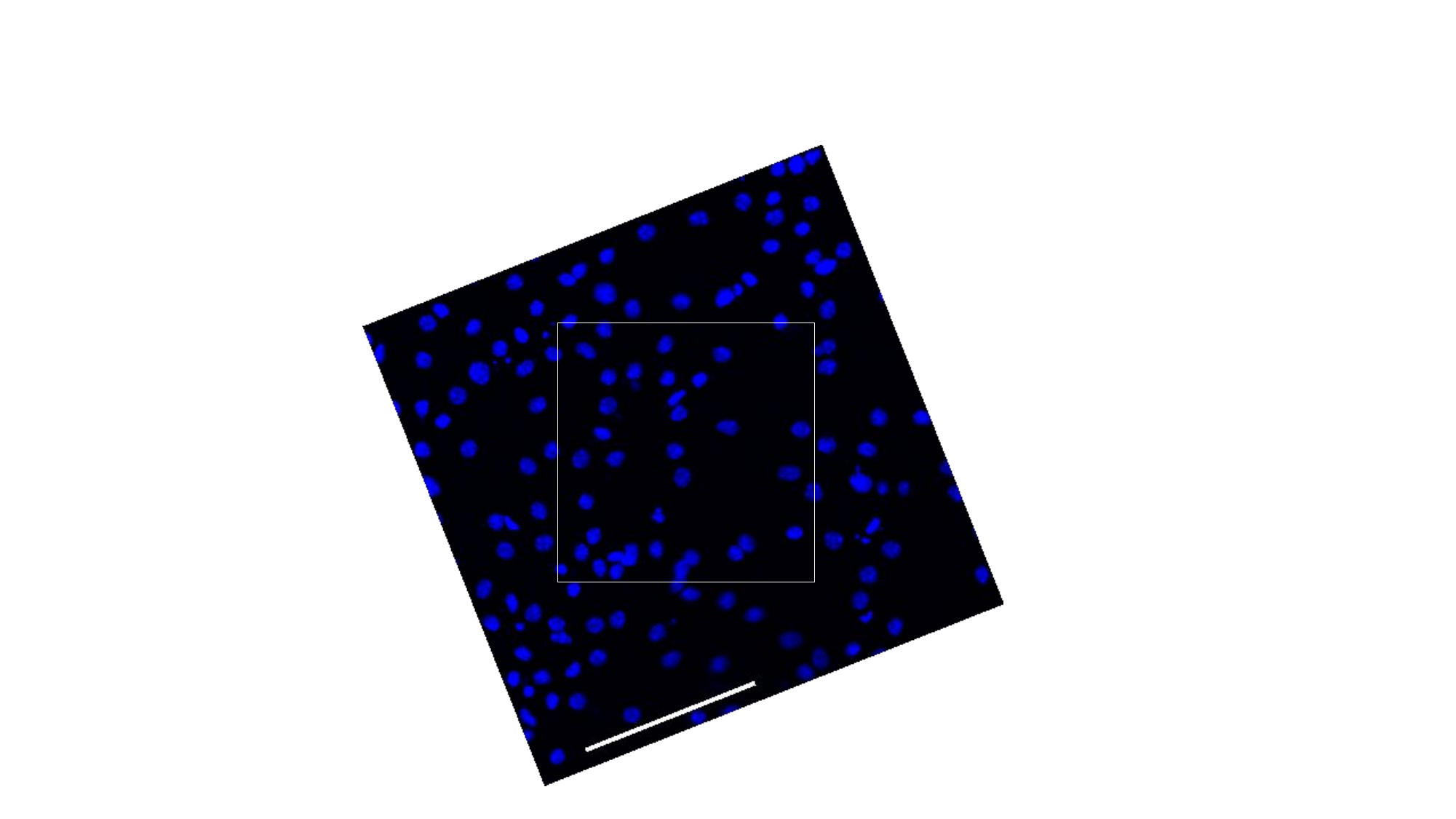

## Slide 21
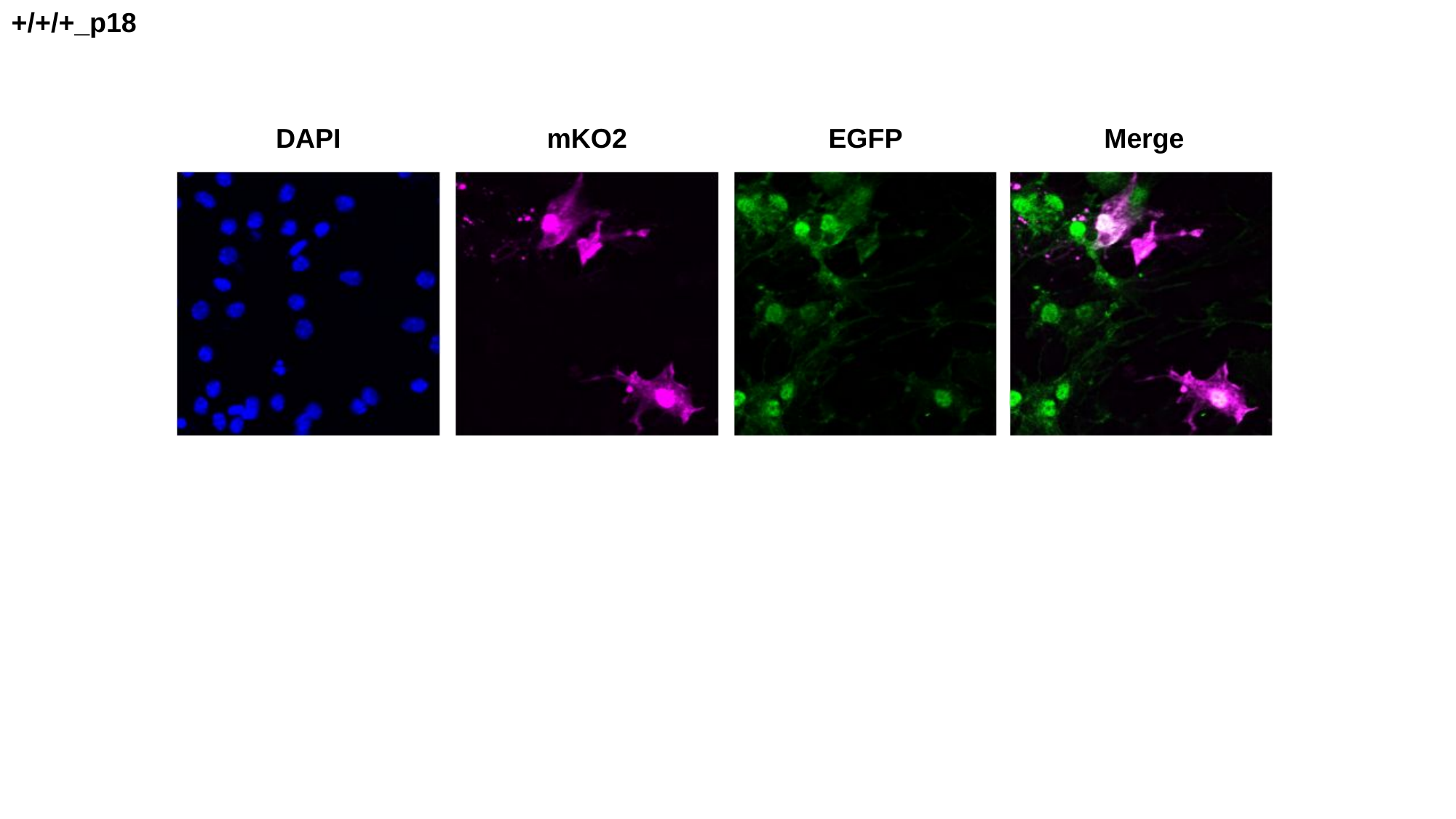

+/+/+_p18
DAPI
mKO2
EGFP
Merge

## Slide 22
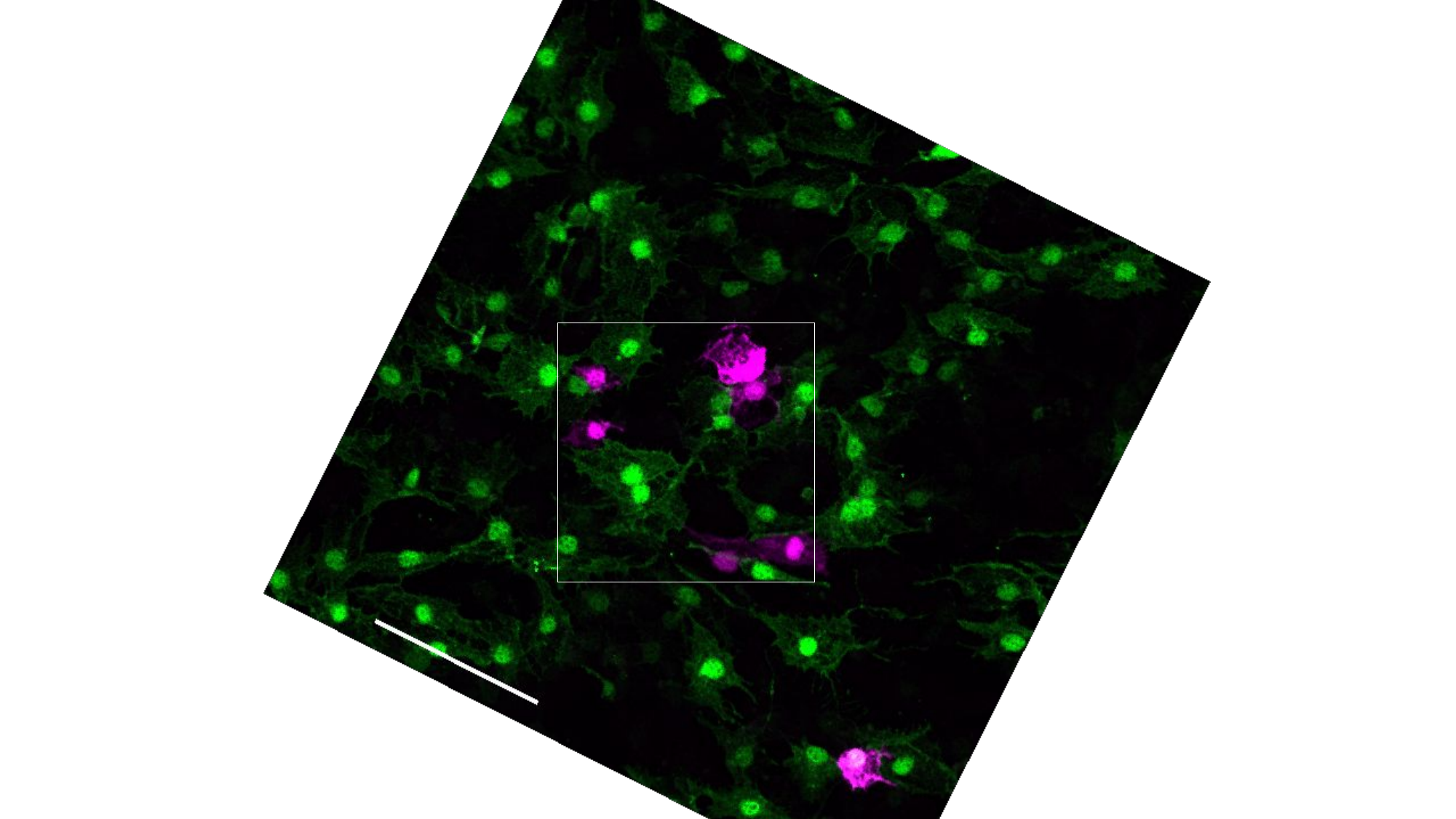

## Slide 23
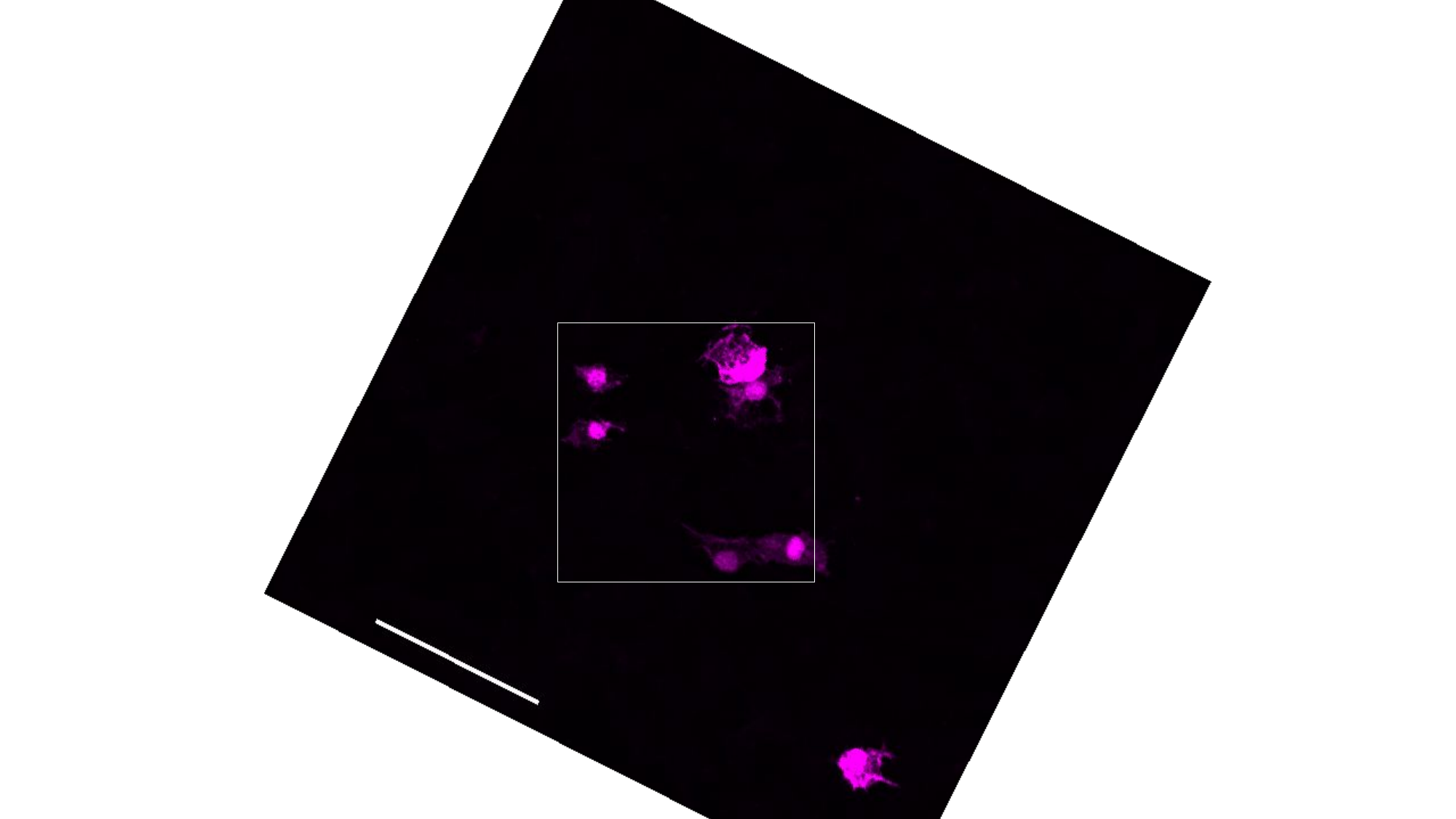

## Slide 24
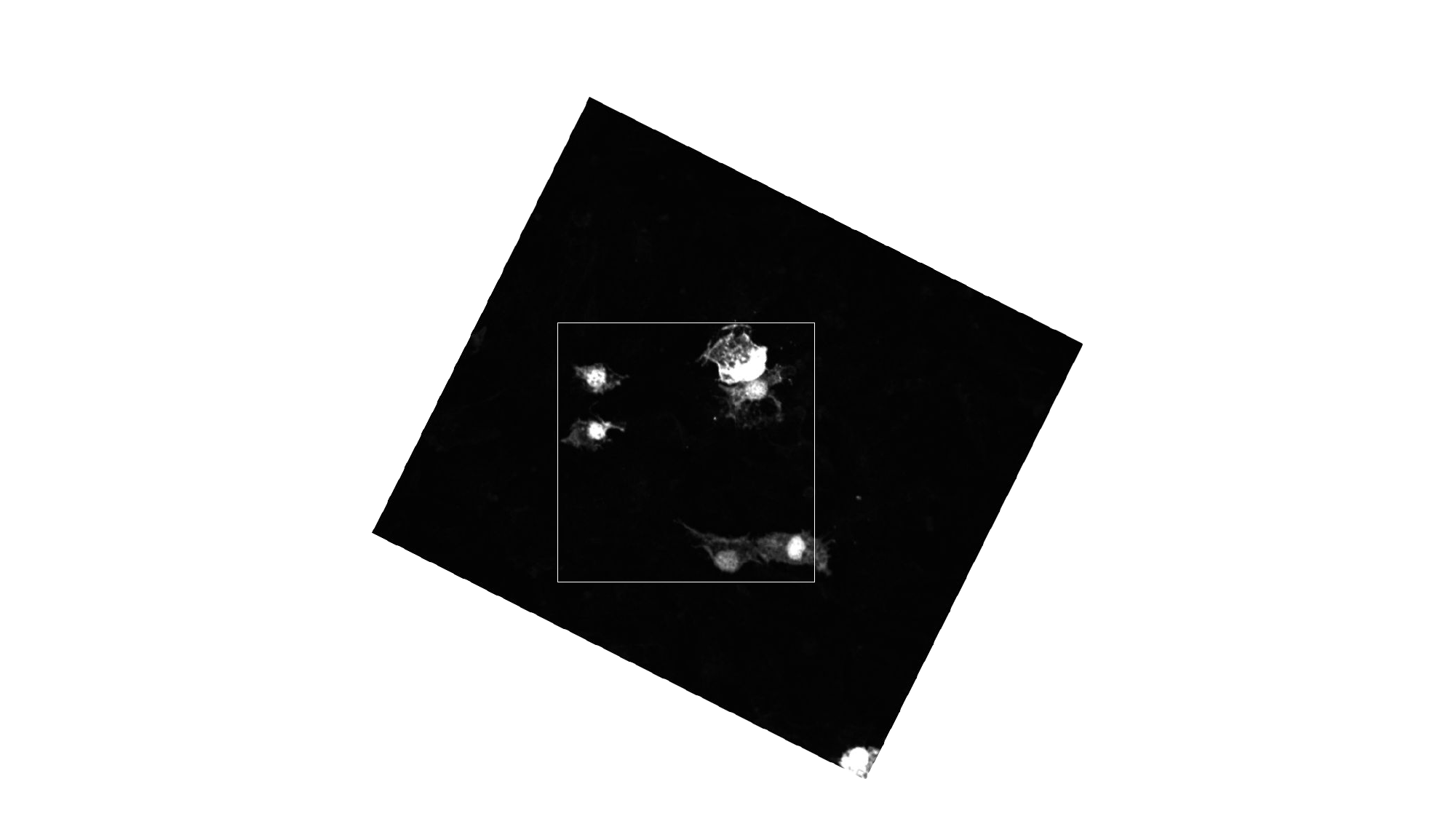

## Slide 25
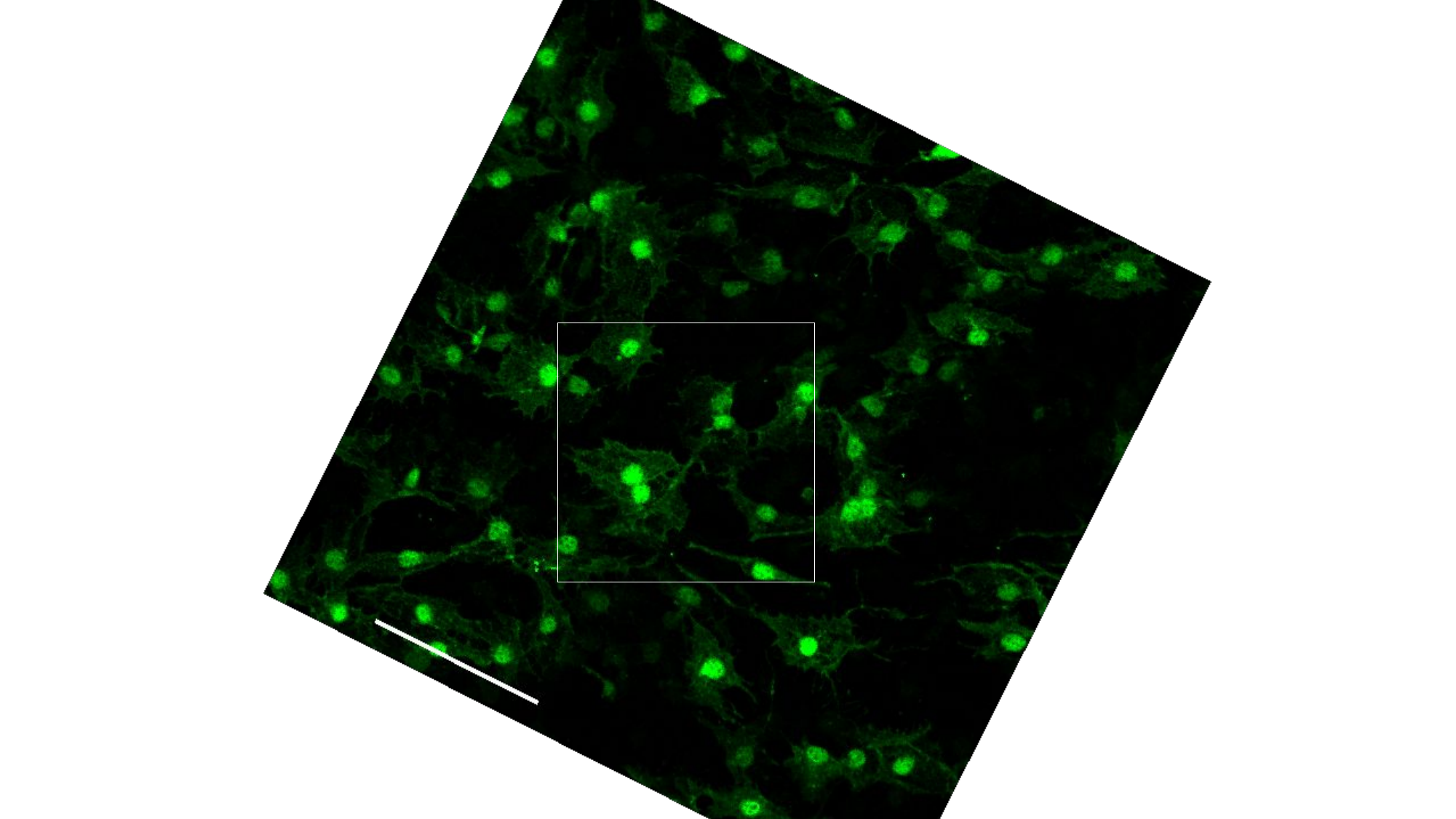

## Slide 26
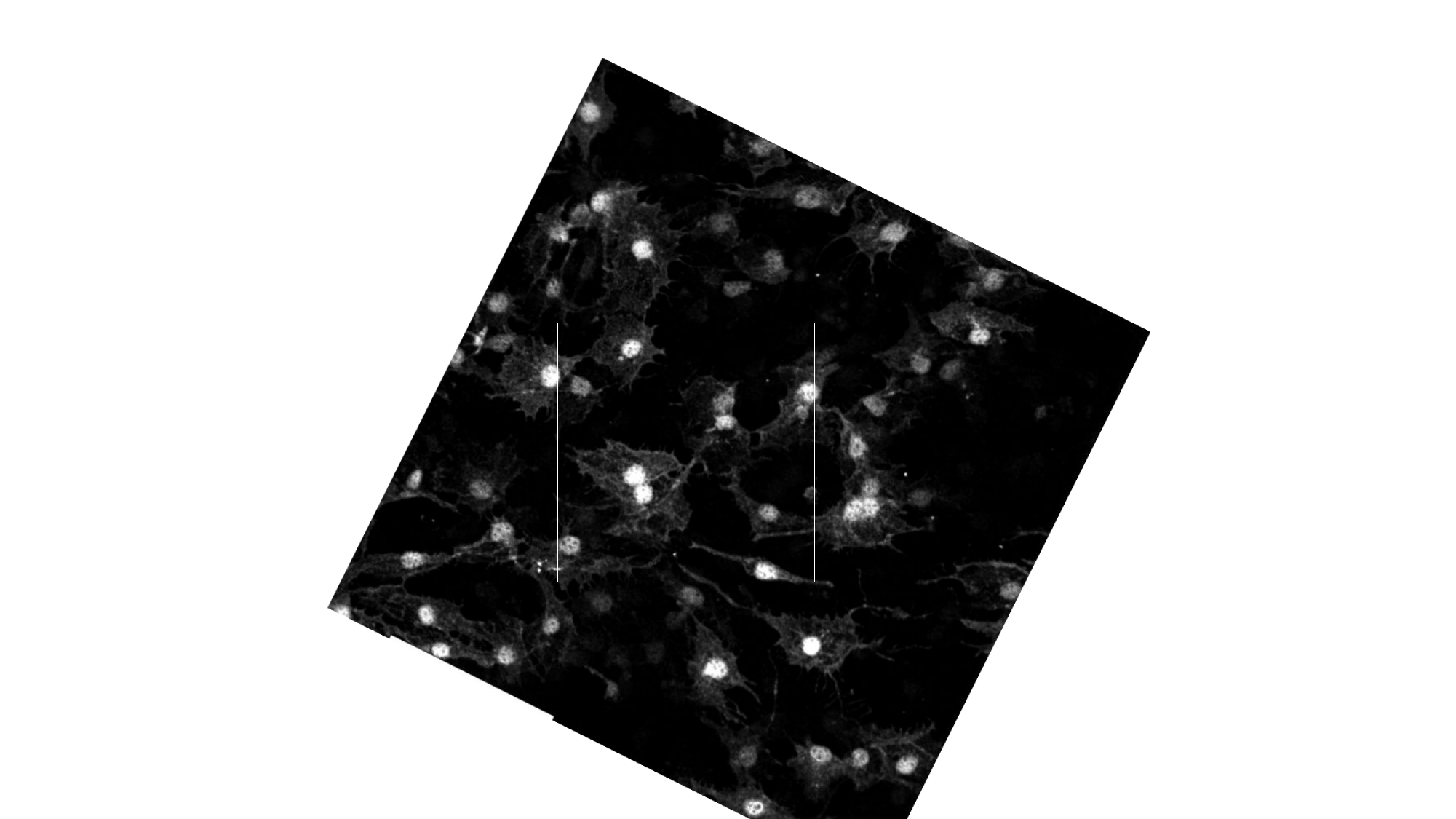

## Slide 27
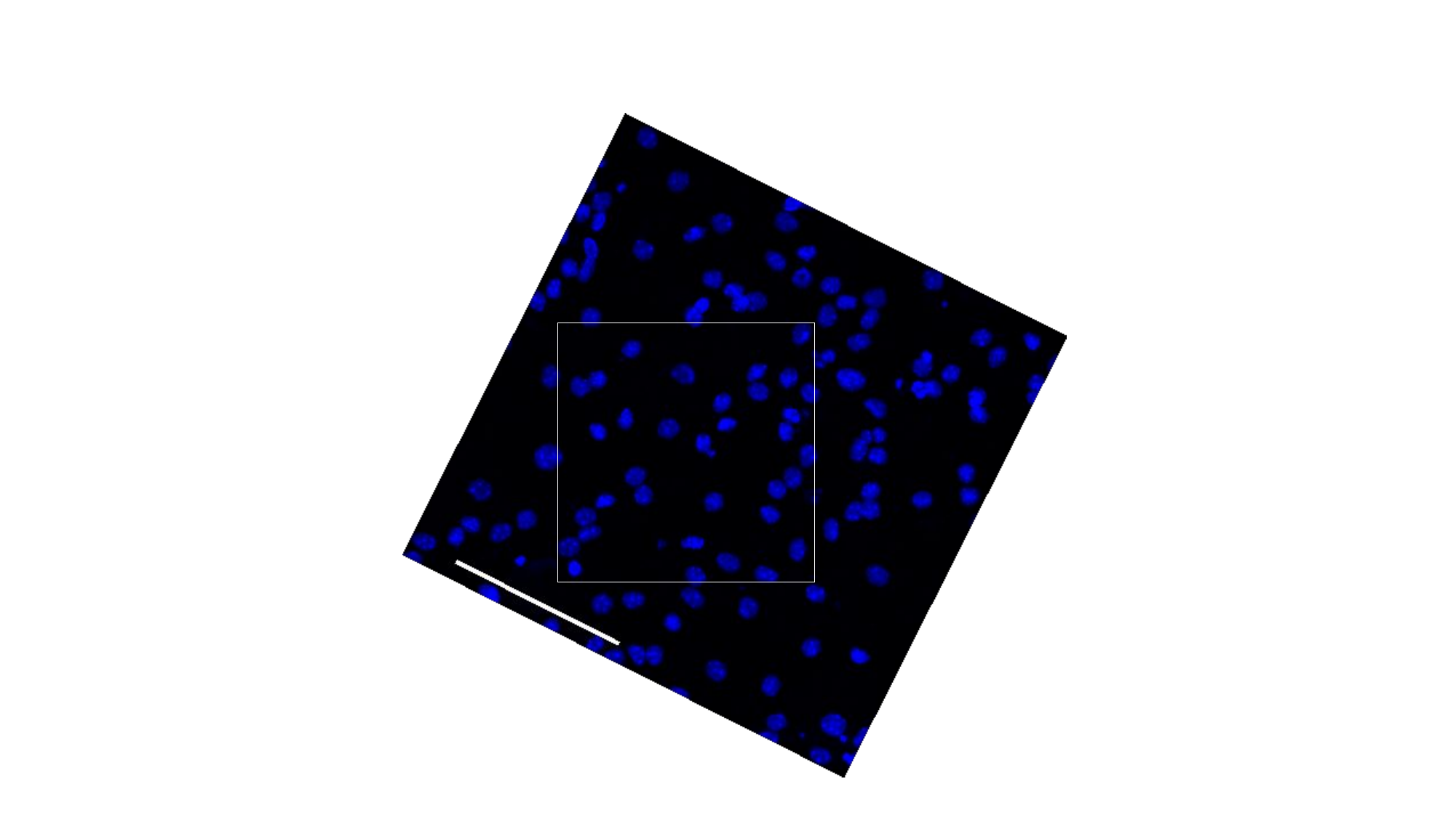

## Slide 28
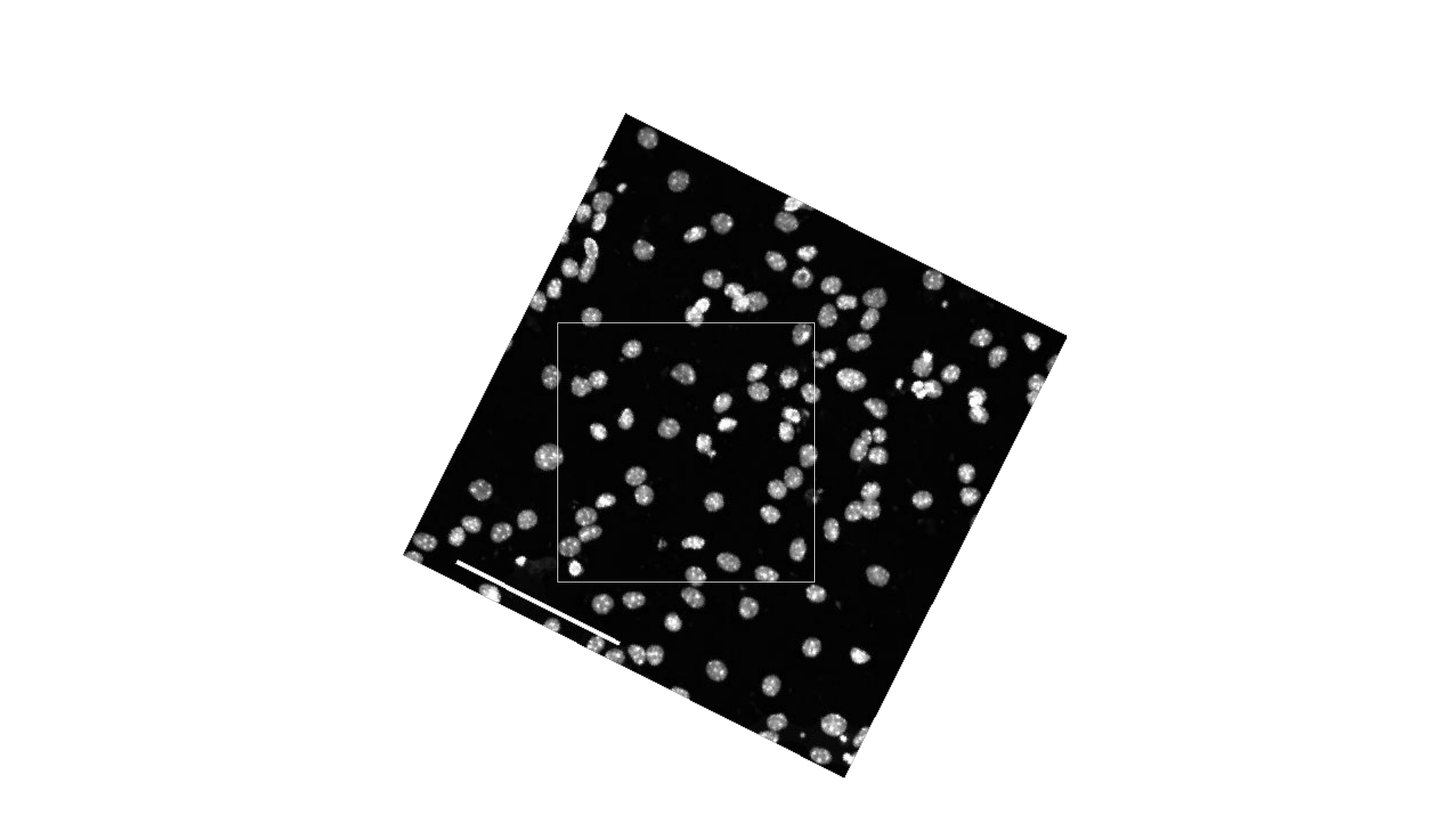

## Slide 29
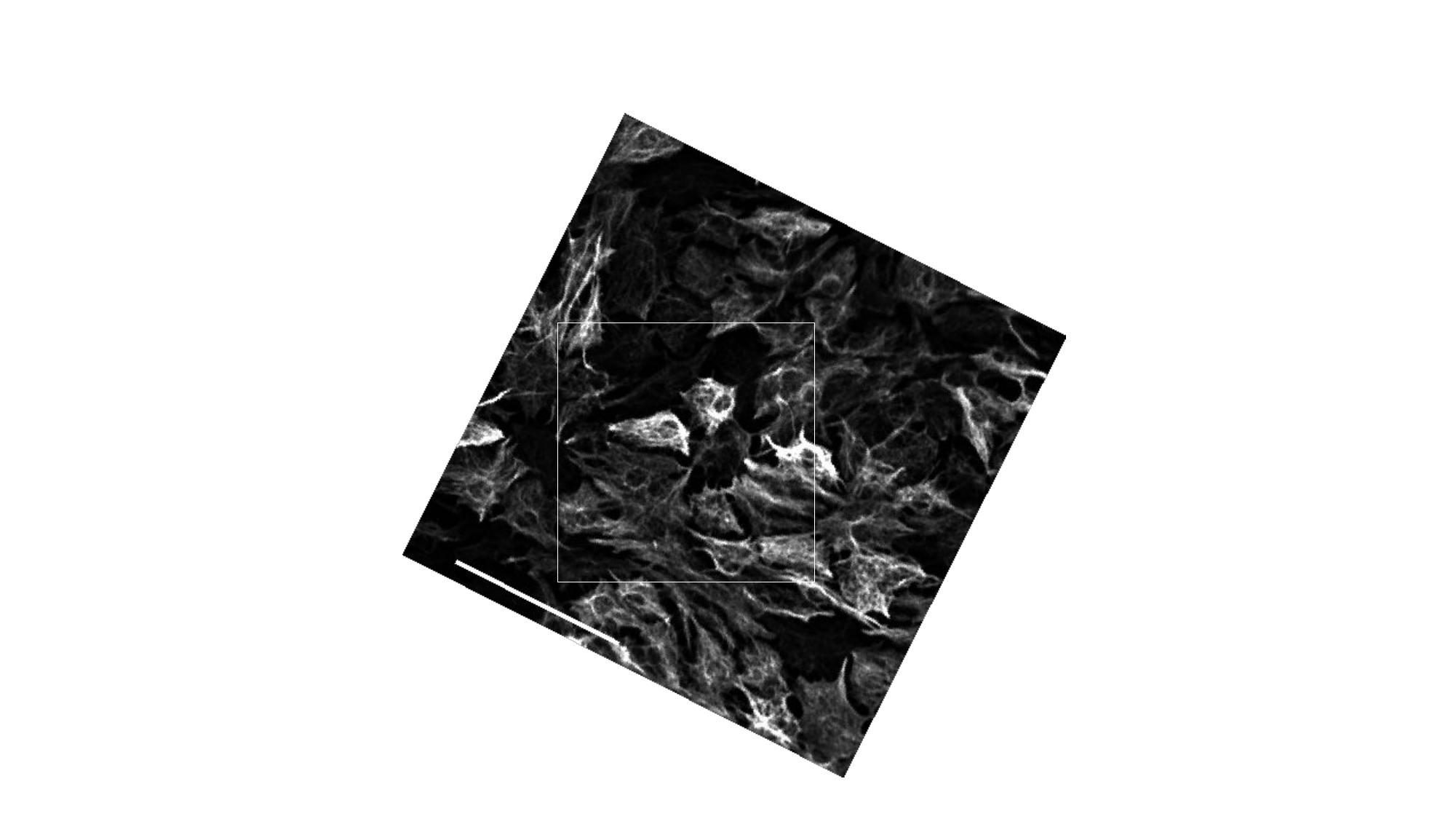

## Slide 30
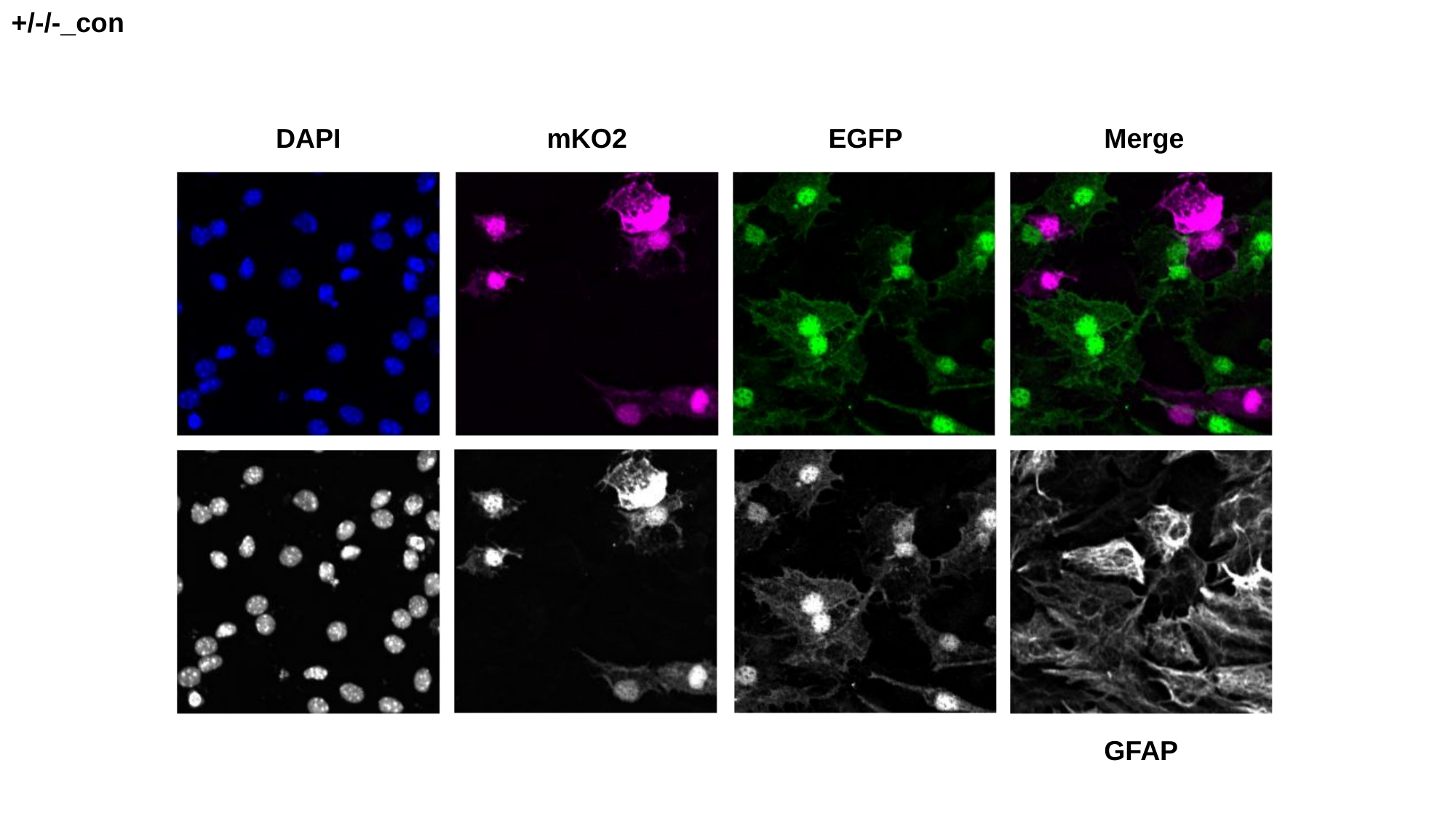

+/-/-_con
DAPI
mKO2
EGFP
Merge
GFAP

## Slide 31
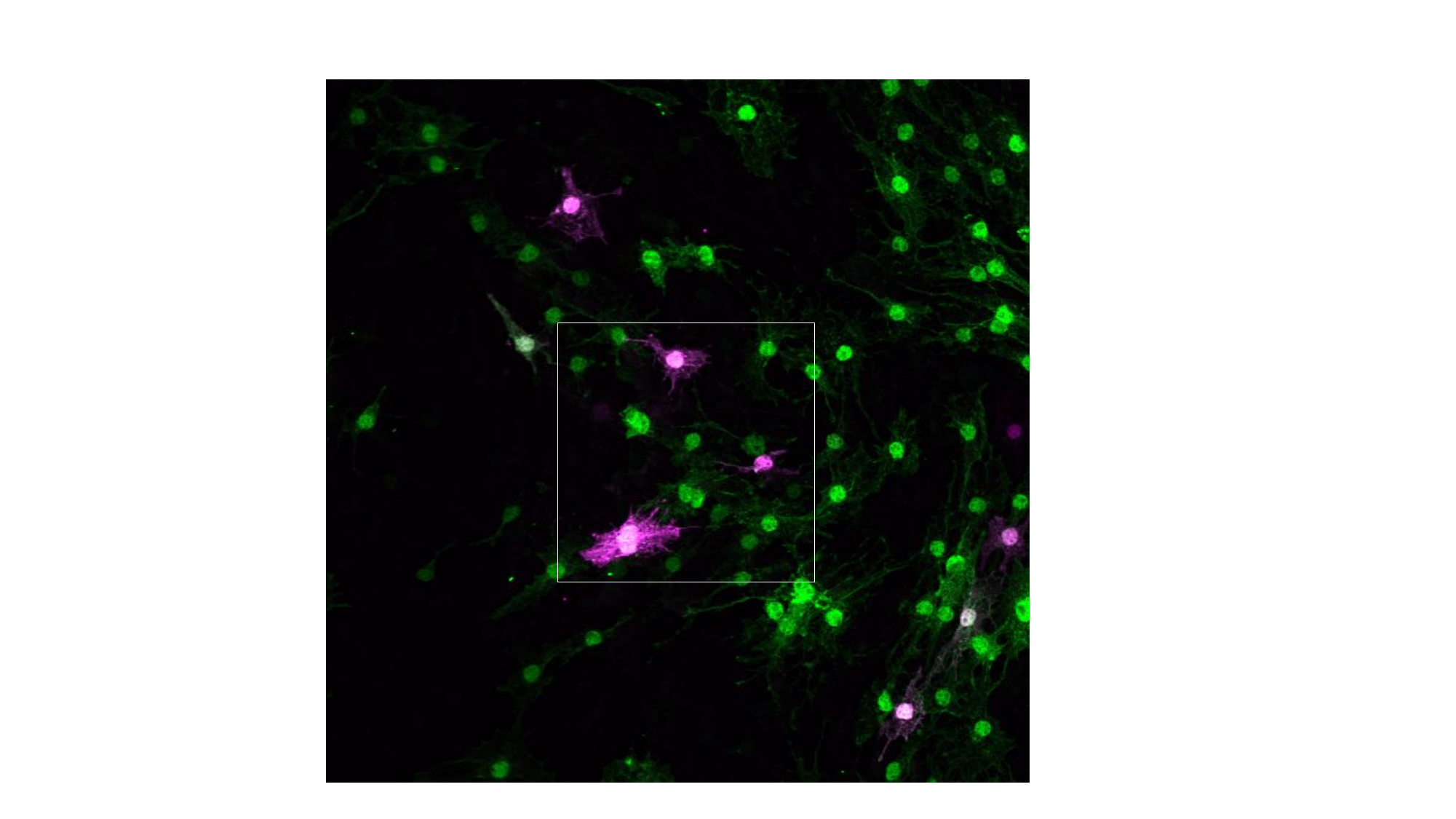

## Slide 32
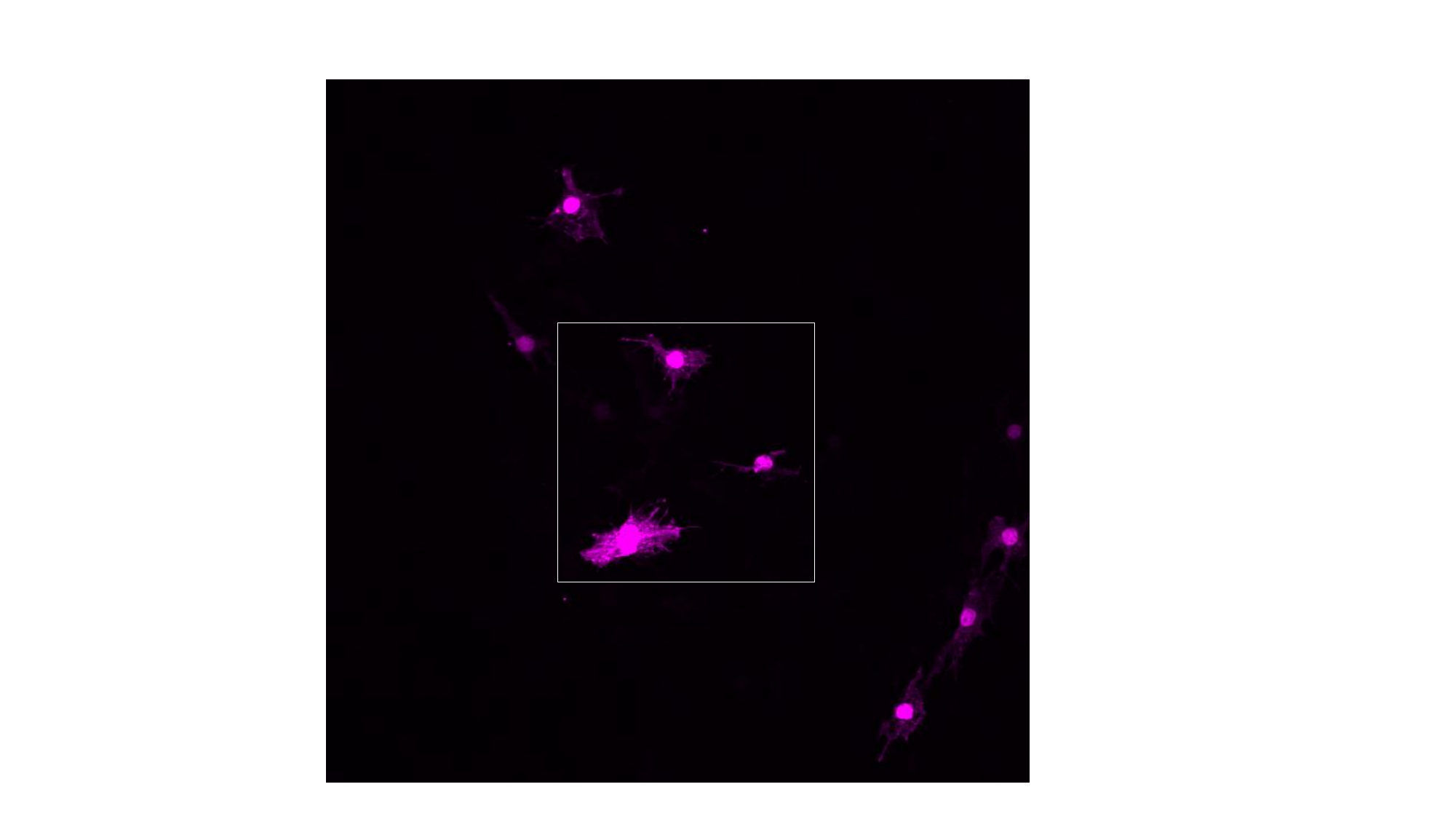

## Slide 33
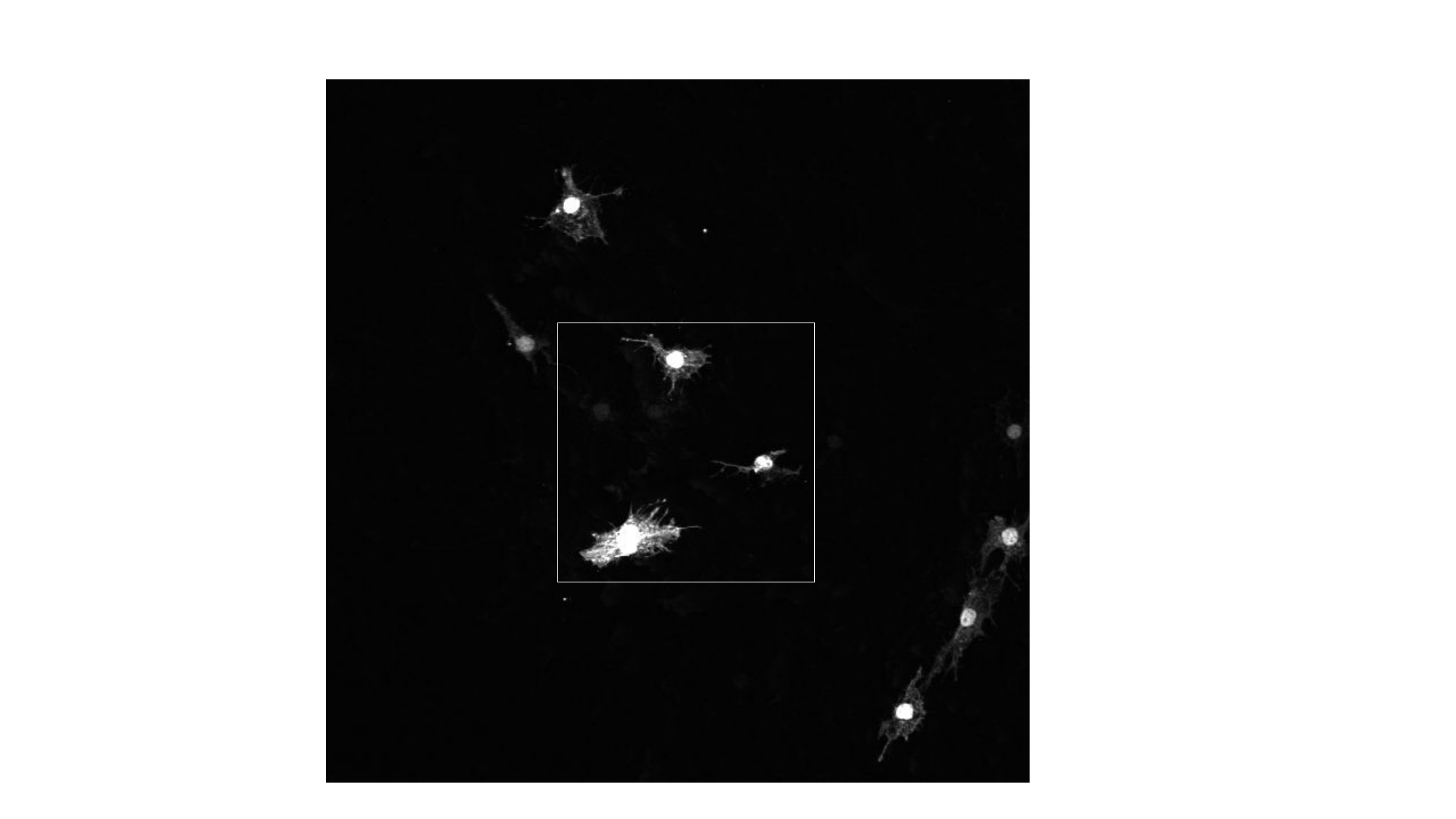

## Slide 34
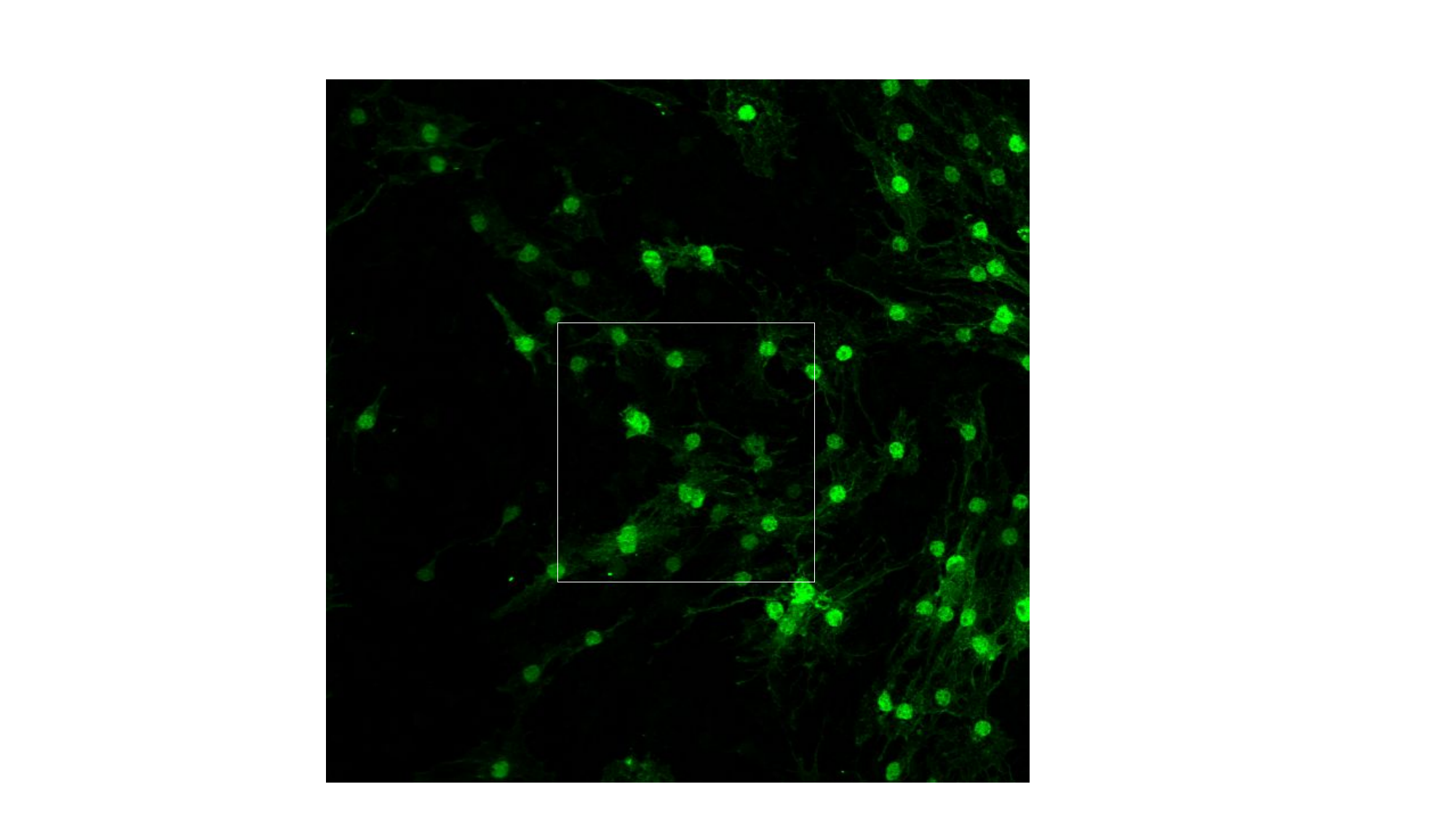

## Slide 35
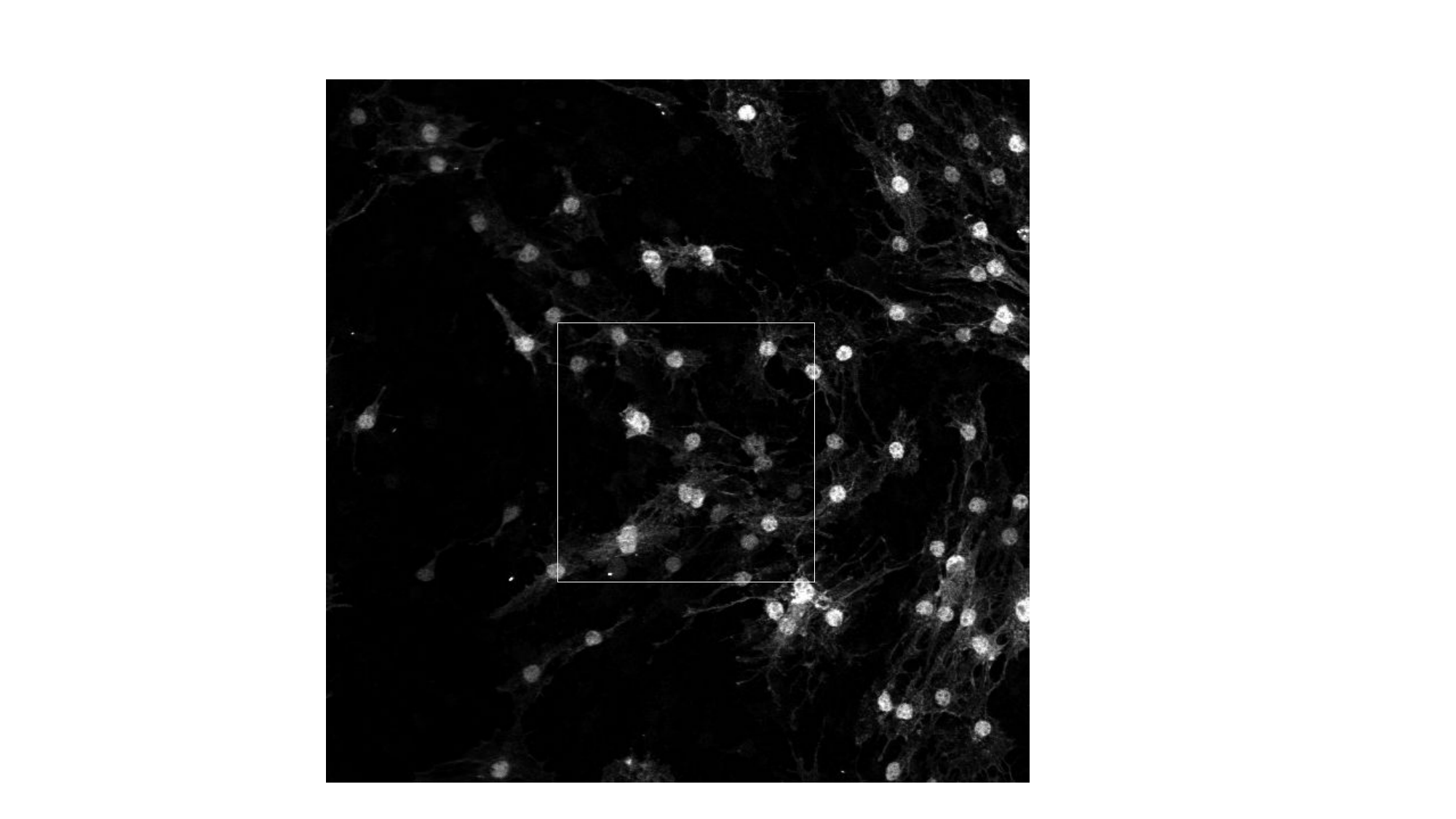

## Slide 36
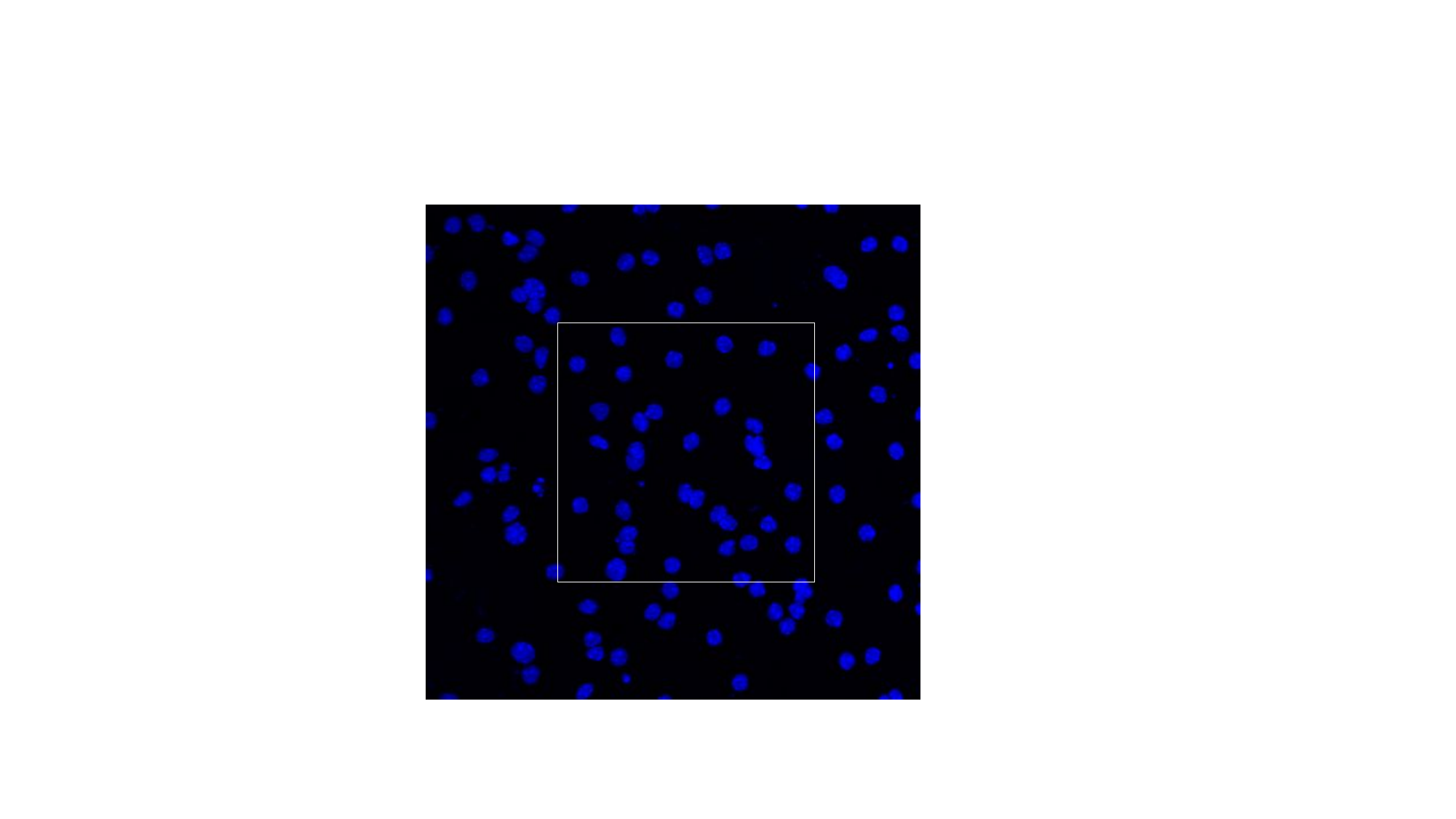

## Slide 37
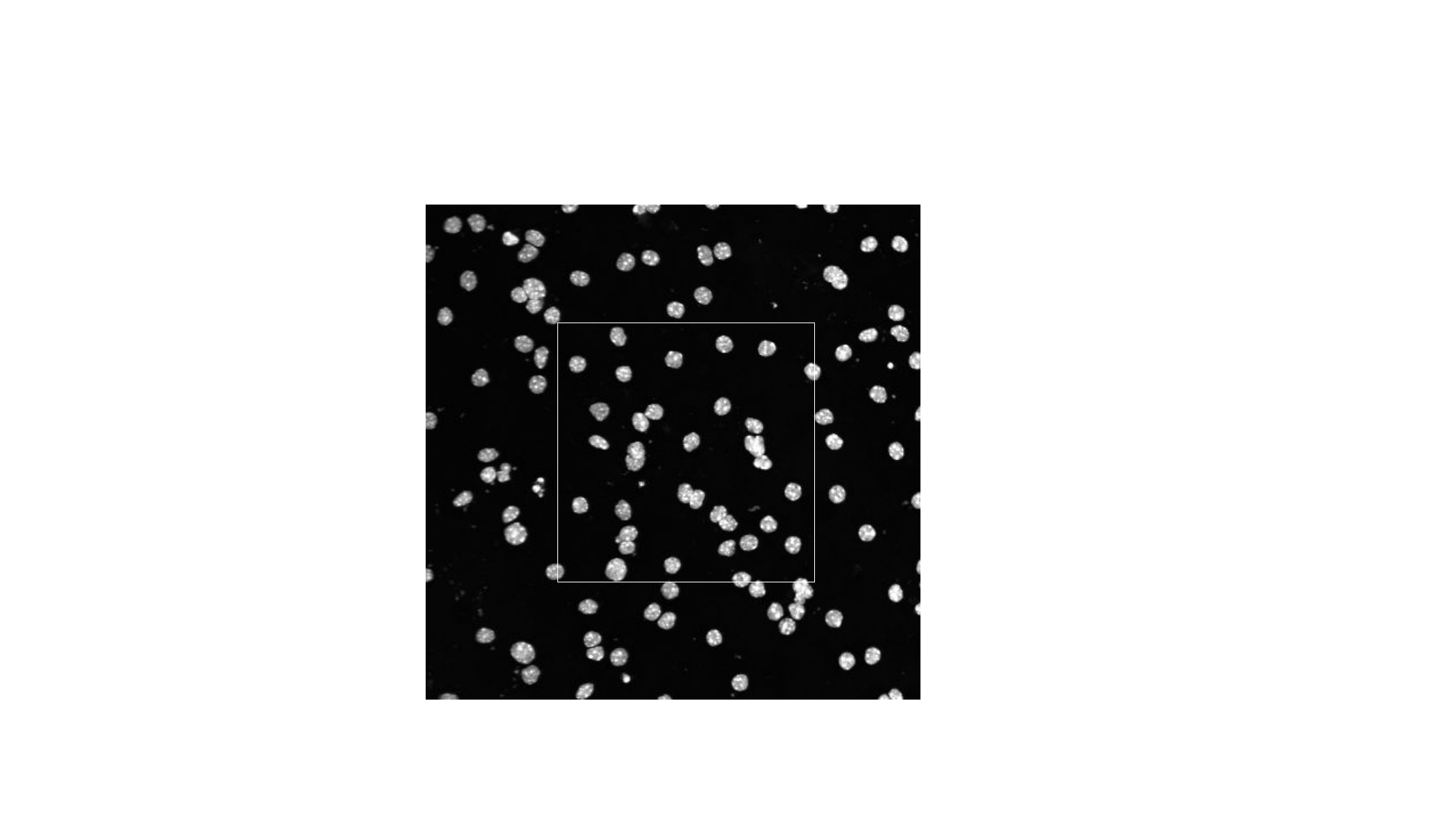

## Slide 38
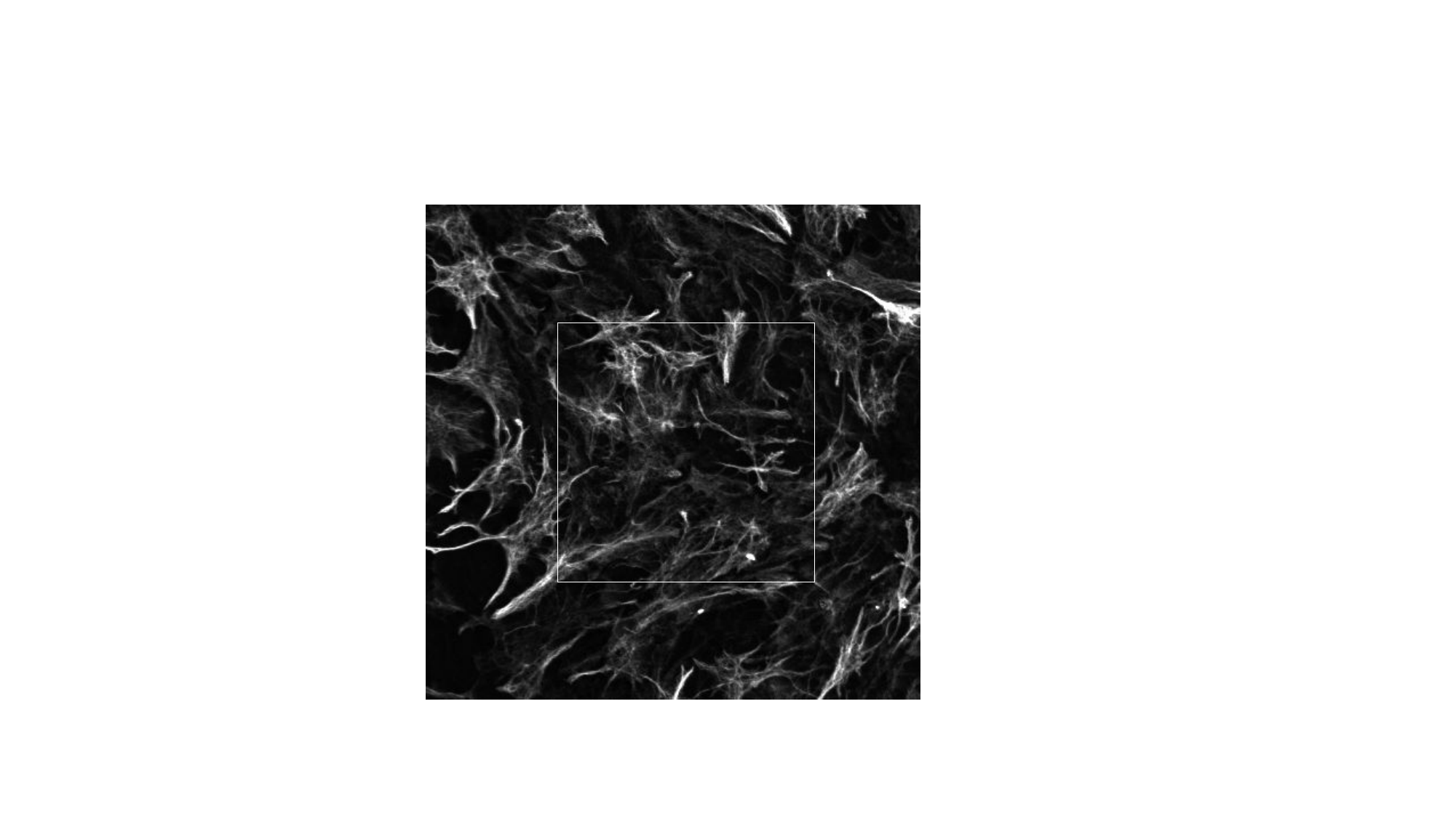

## Slide 39
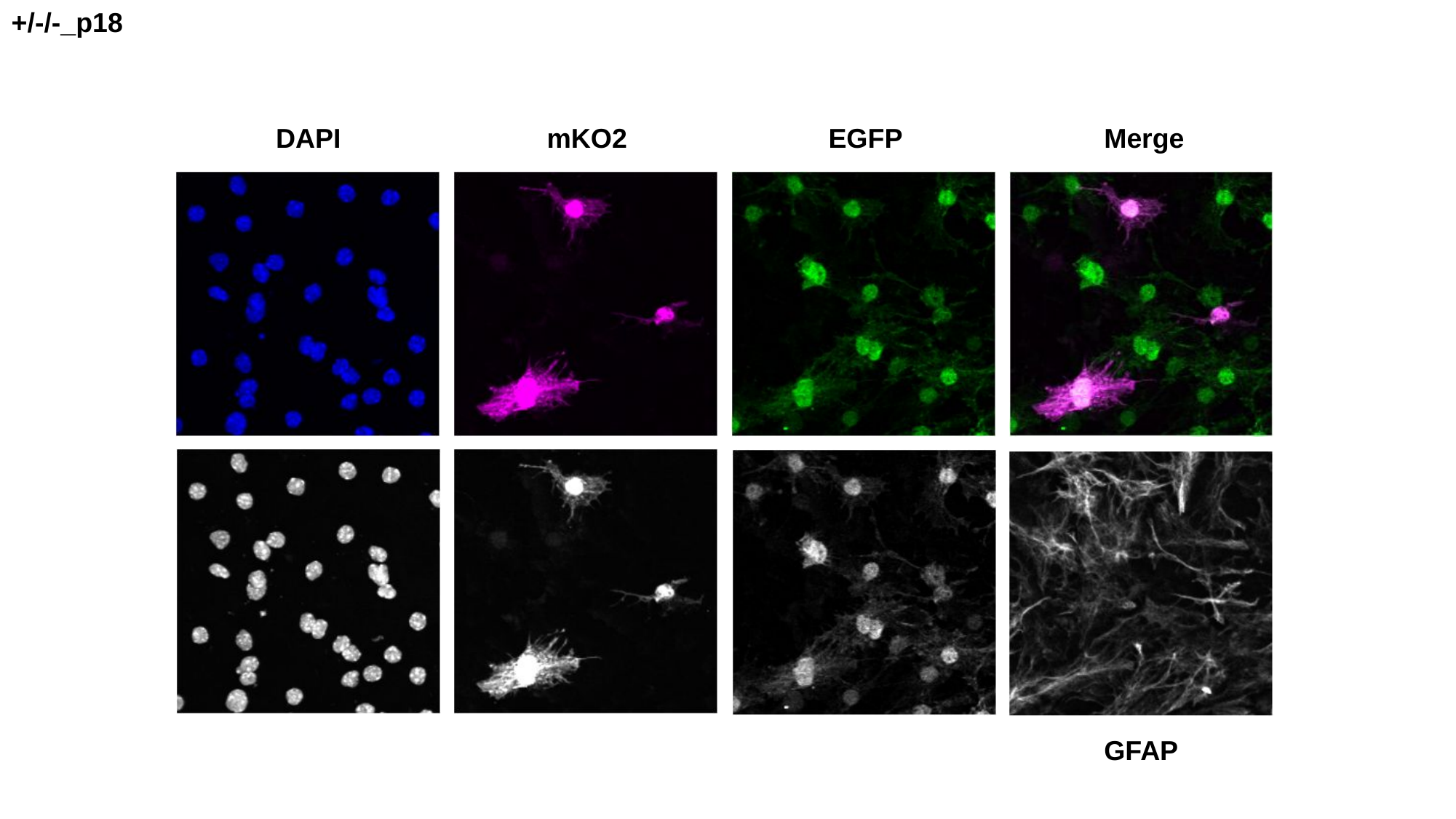

+/-/-_p18
DAPI
mKO2
EGFP
Merge
GFAP

## Slide 40
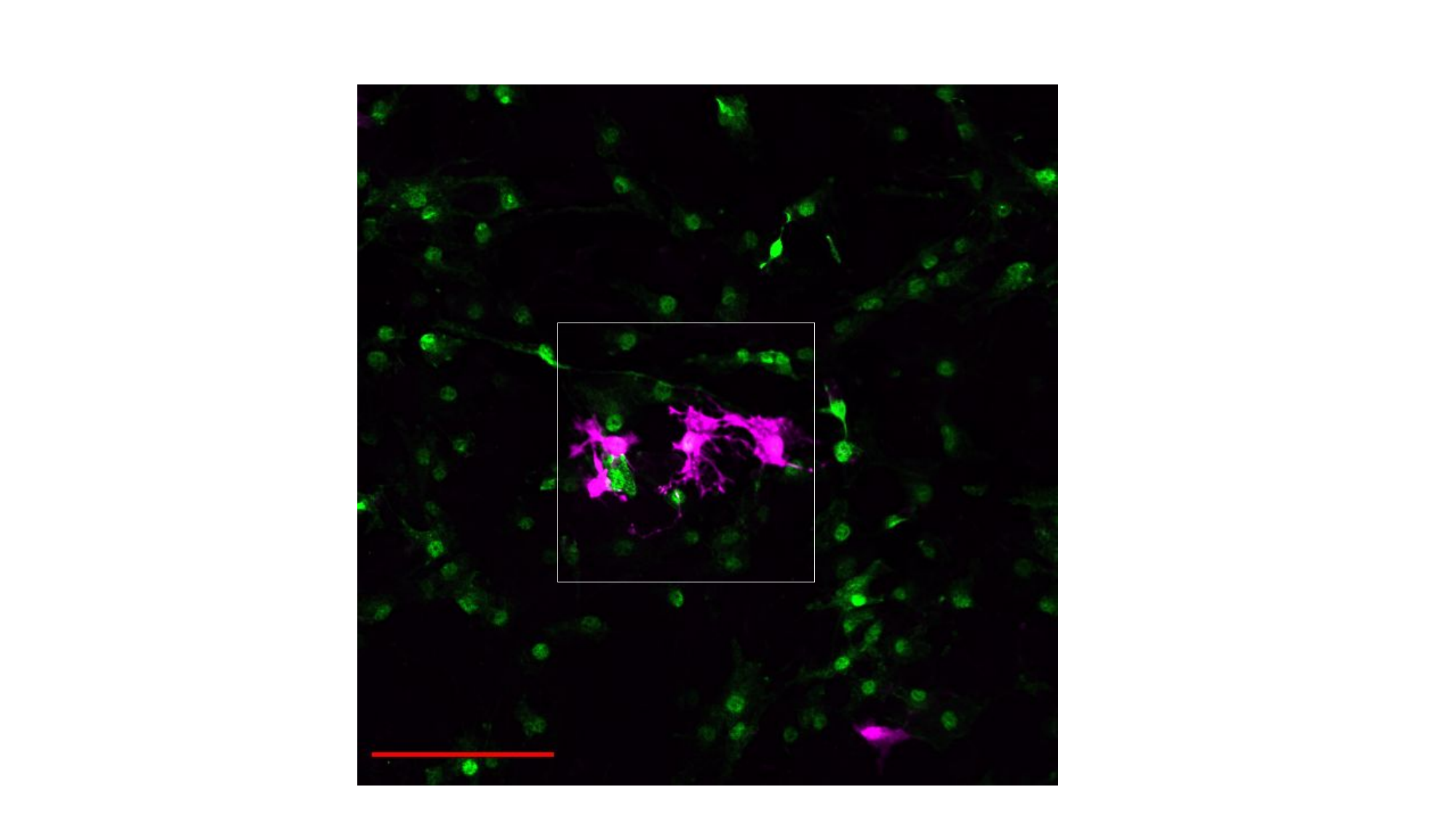

## Slide 41
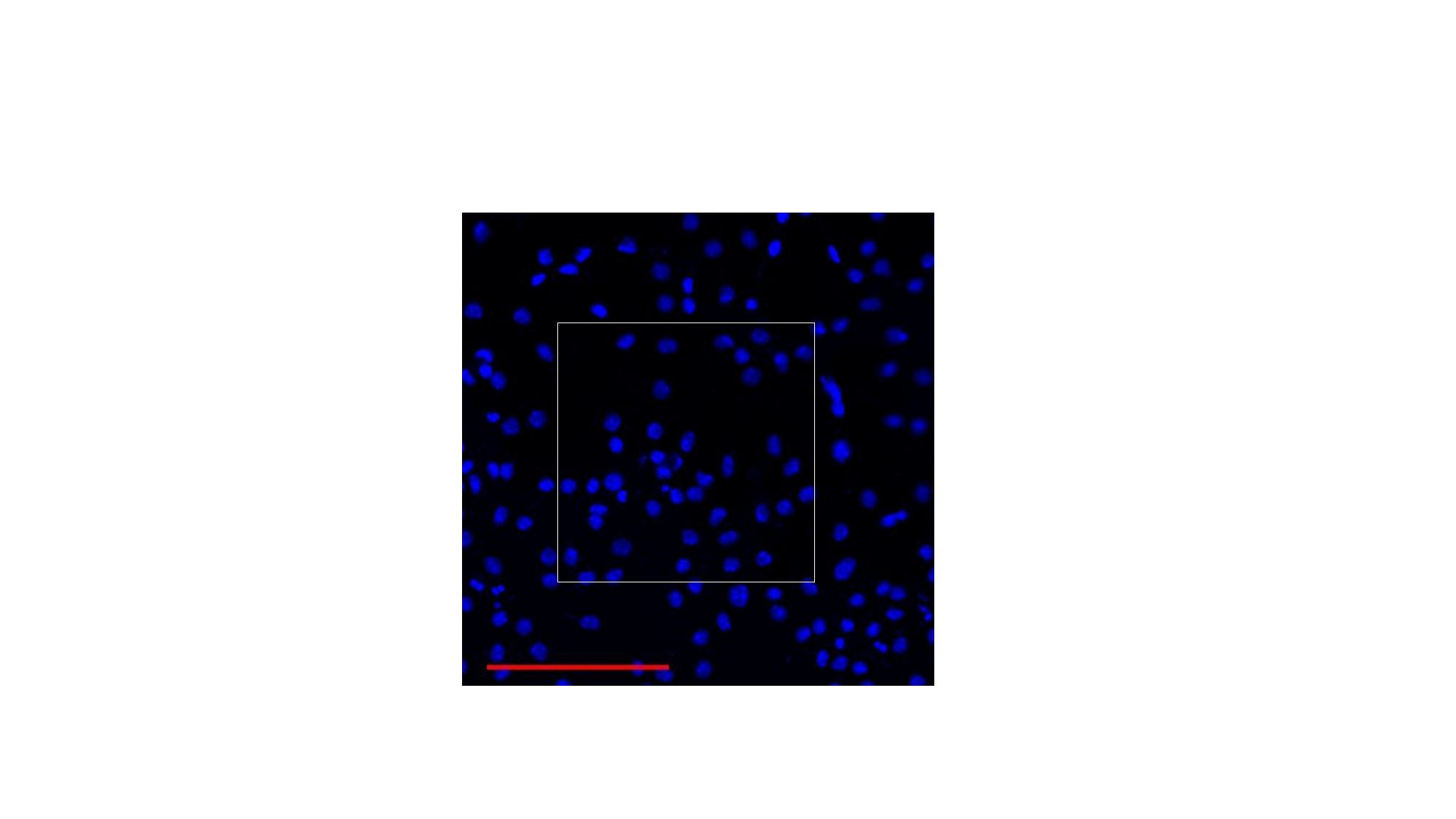

## Slide 42
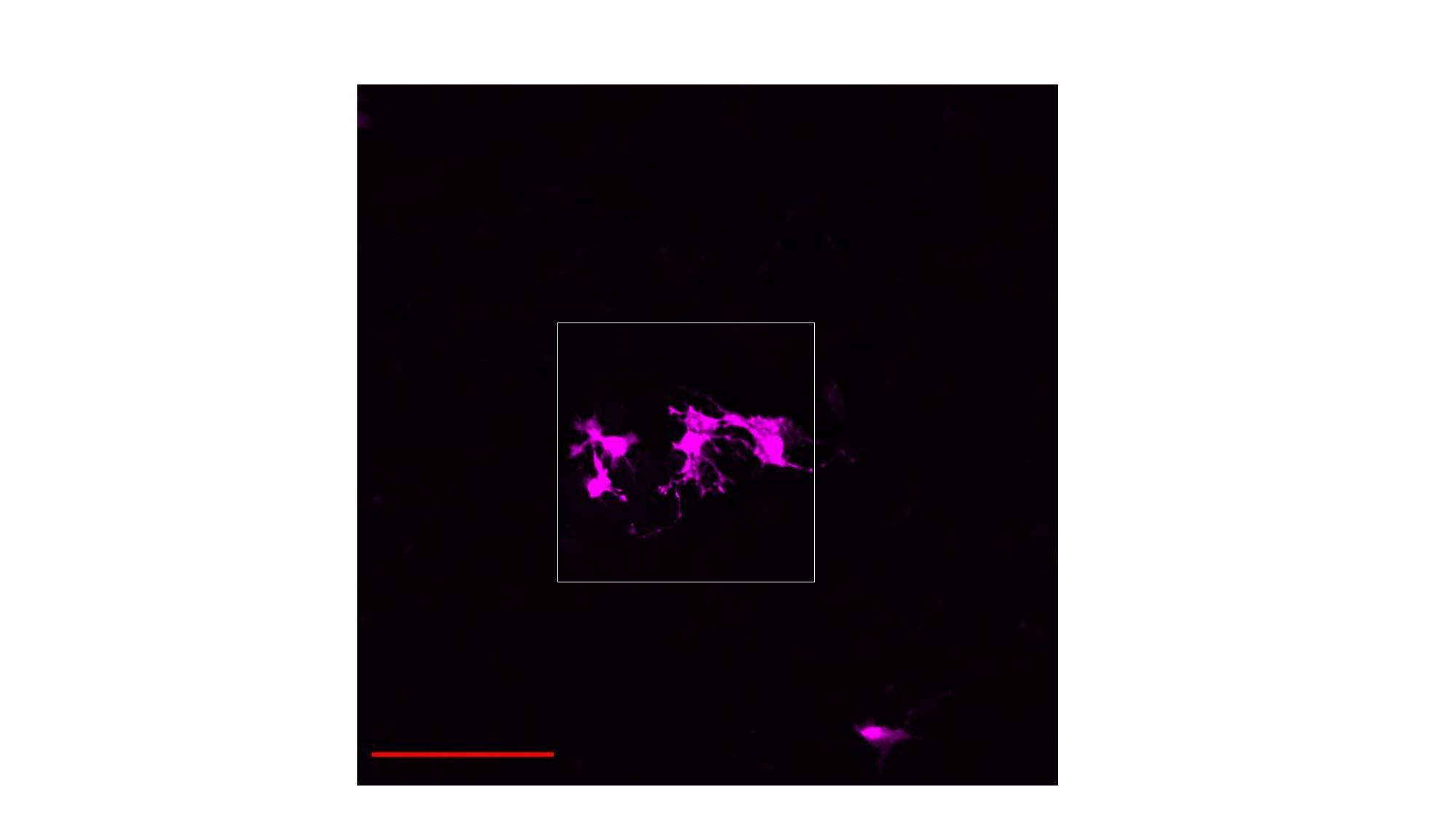

## Slide 43
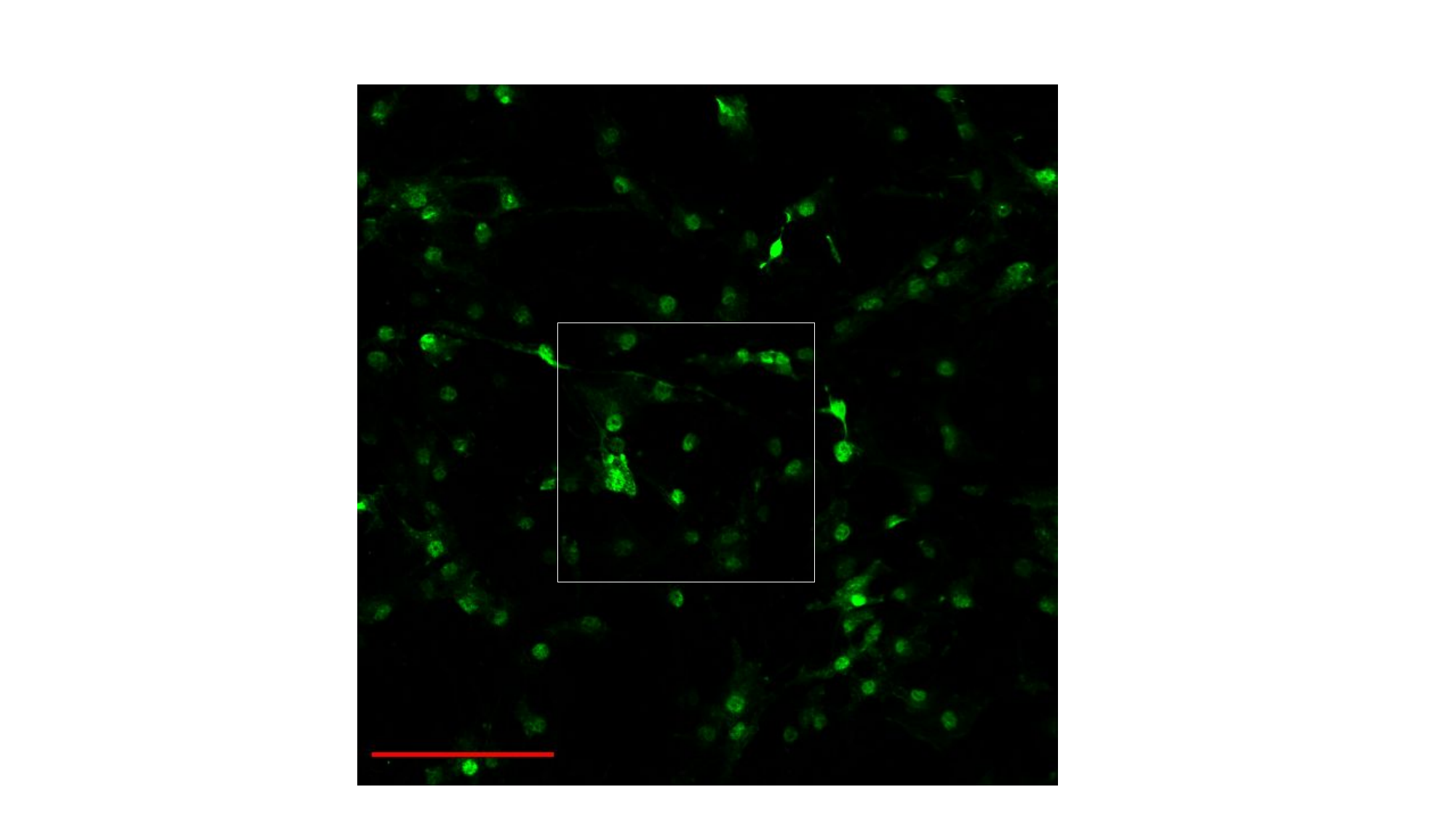

## Slide 44
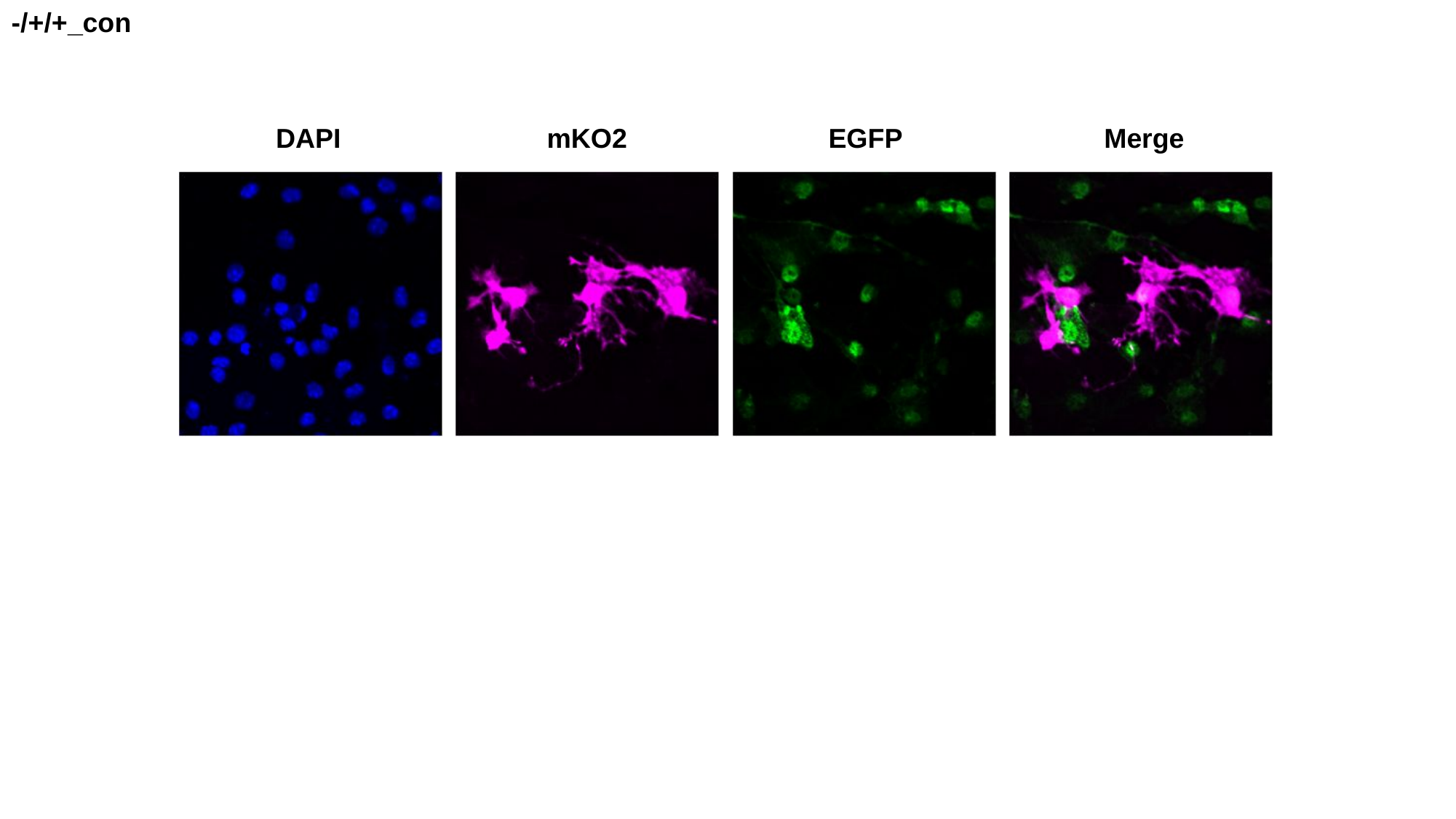

-/+/+_con
DAPI
mKO2
EGFP
Merge

## Slide 45
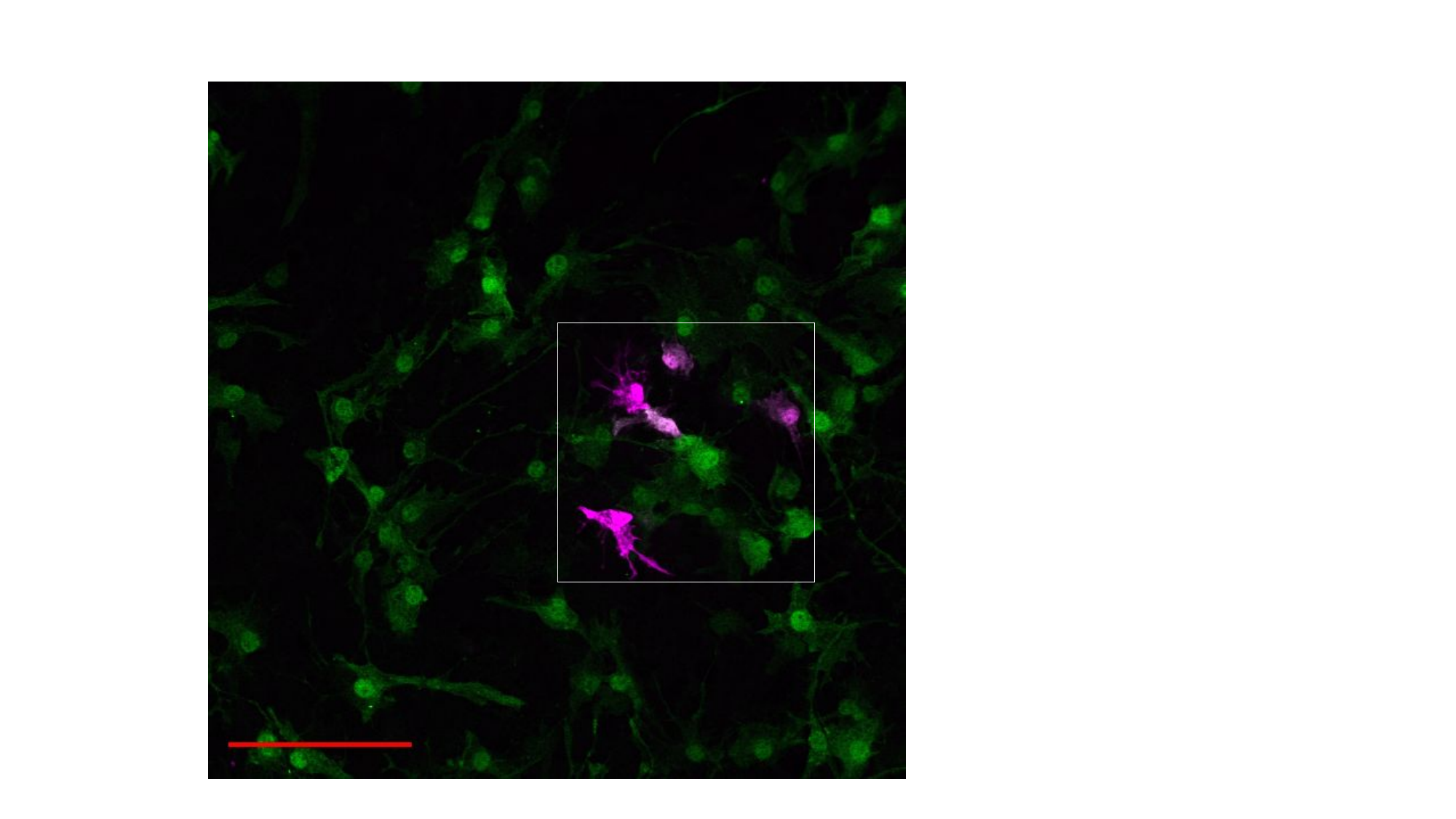

## Slide 46
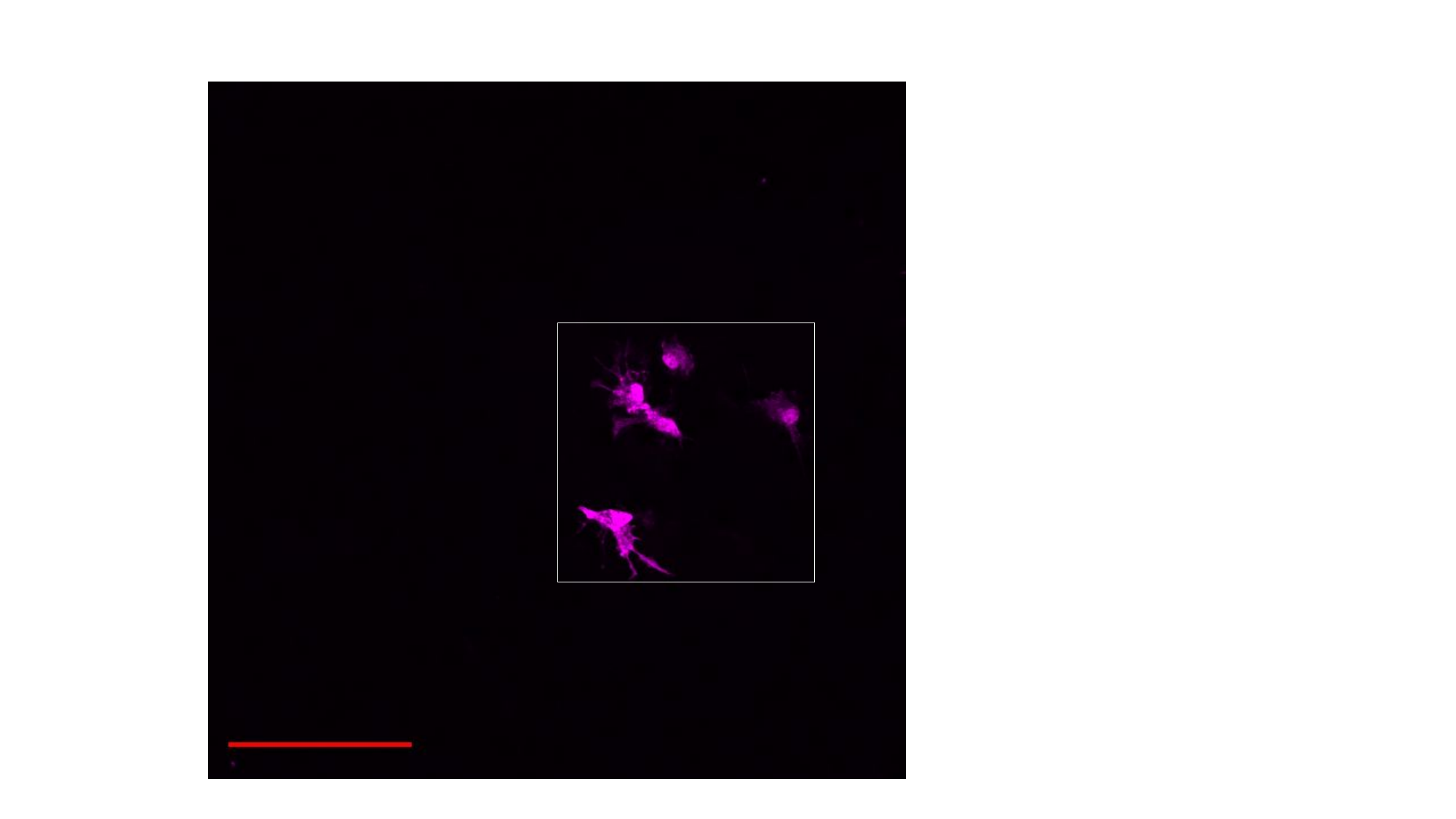

## Slide 47
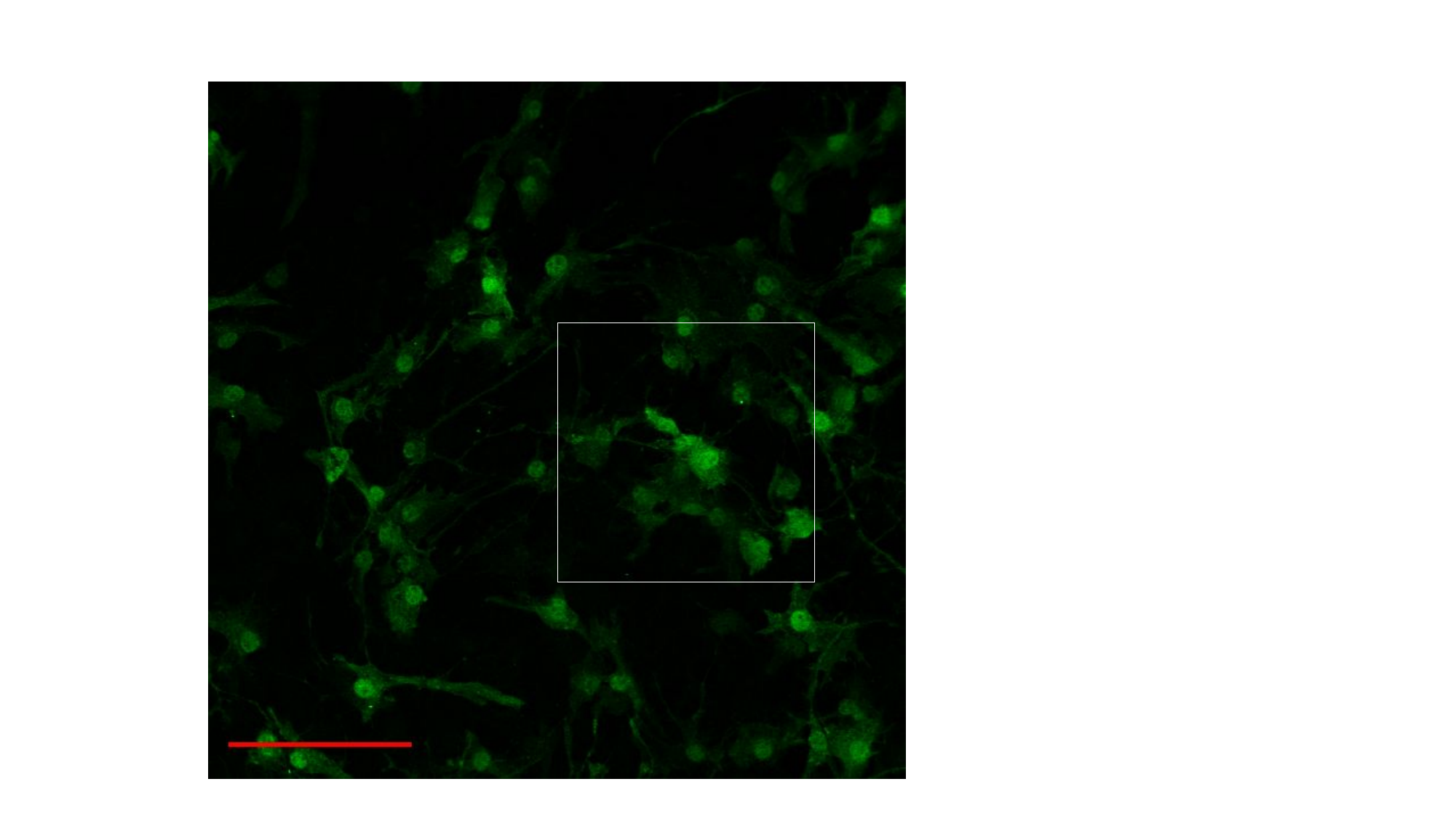

## Slide 48
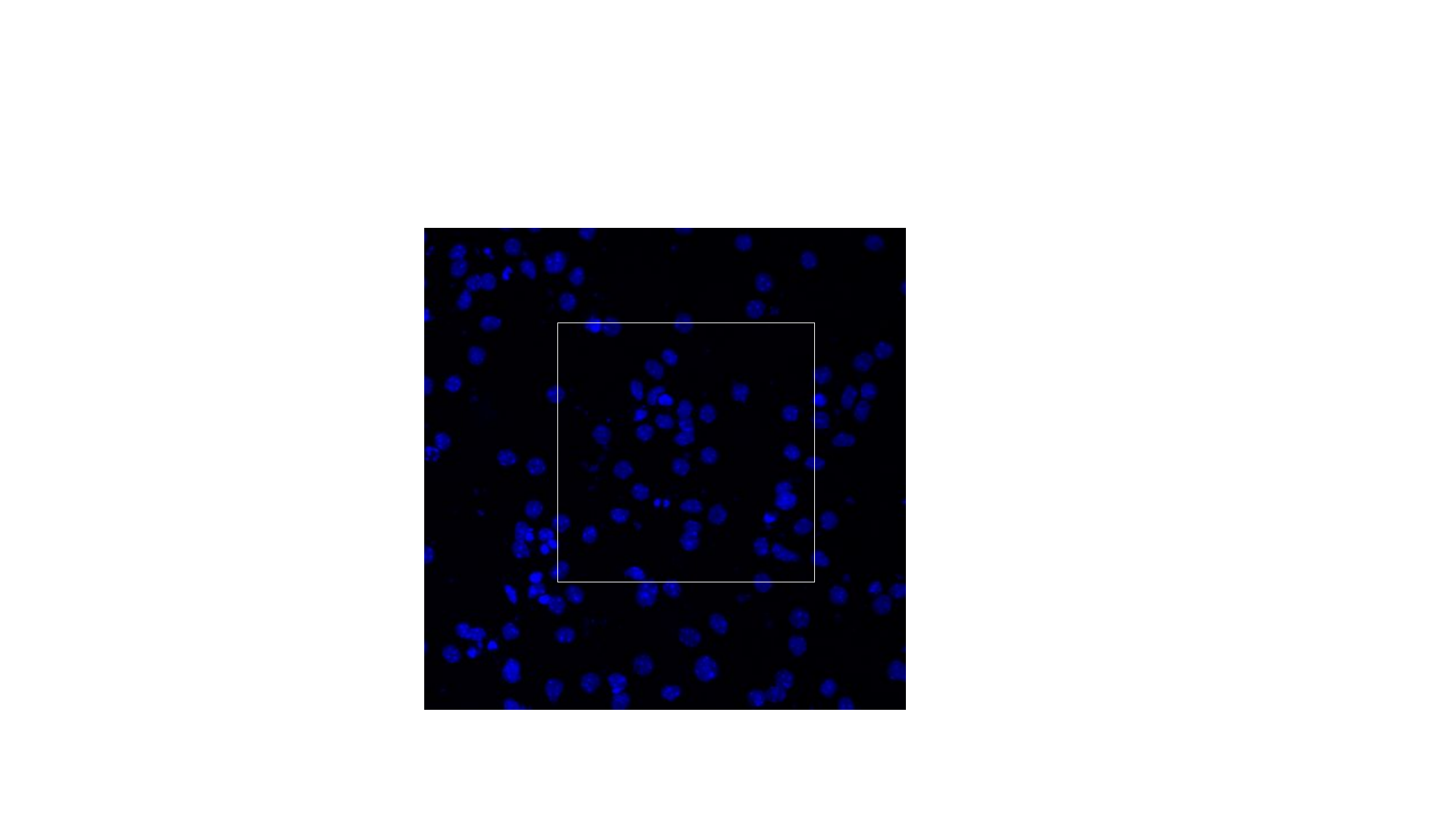

## Slide 49
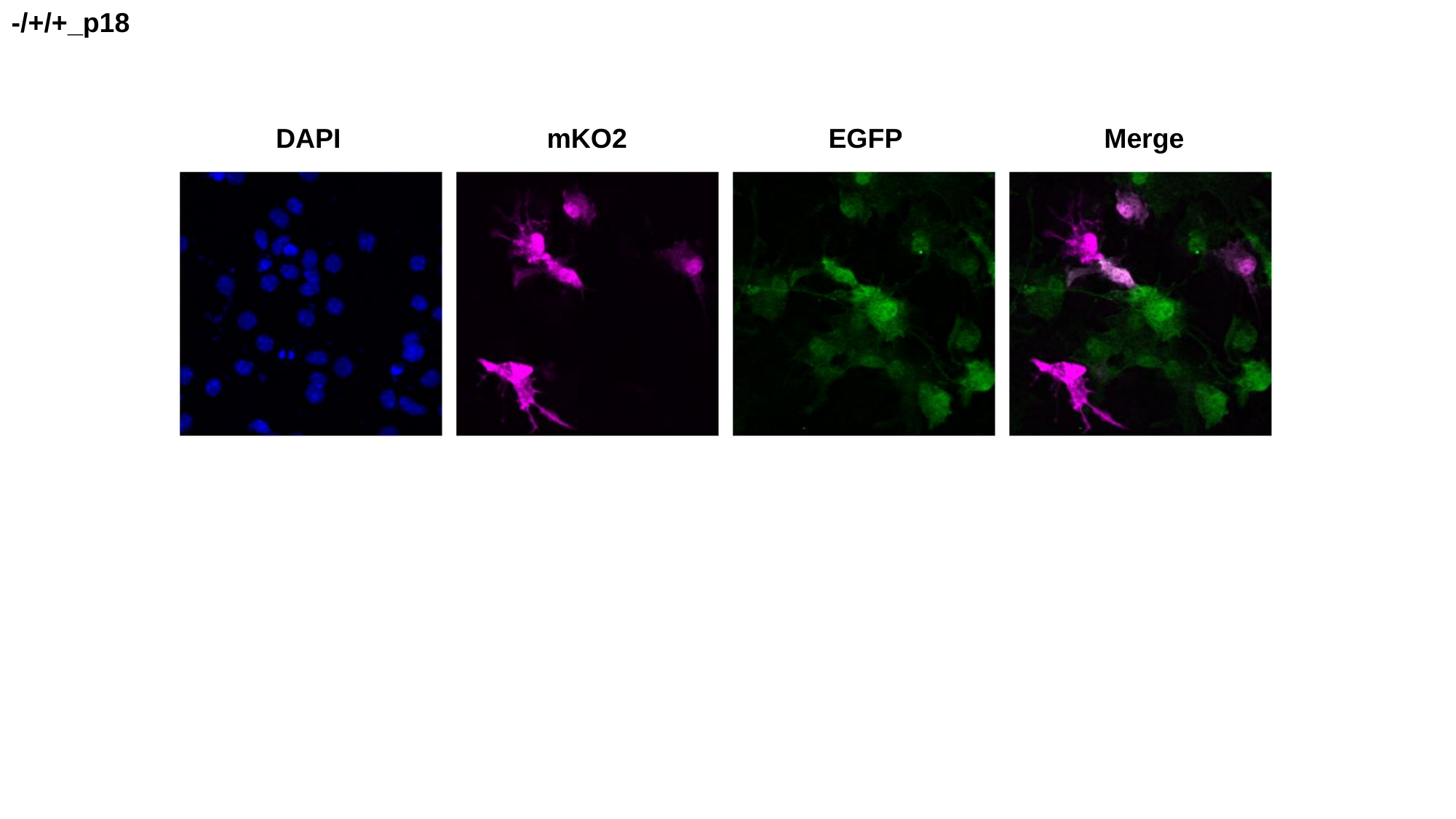

-/+/+_p18
DAPI
mKO2
EGFP
Merge
