# Supplementary material for: Cyclin-dependent kinase inhibitor p18 regulates lineage transitions of excitatory neurons, astrocytes, and interneurons in the mouse cortex
Source: EMBO J. 2024 Dec 12;44(2):382–412. doi: 10.1038/s44318-024-00325-9 (PMC11730326; doi:10.1038/s44318-024-00325-9)
Supplement: Supplementary file 5 — Source data Fig. 3 [file 44318_2024_325_MOESM5_ESM.zip › 3E/3E_b,d,f,h.pptx]

## Slide 1
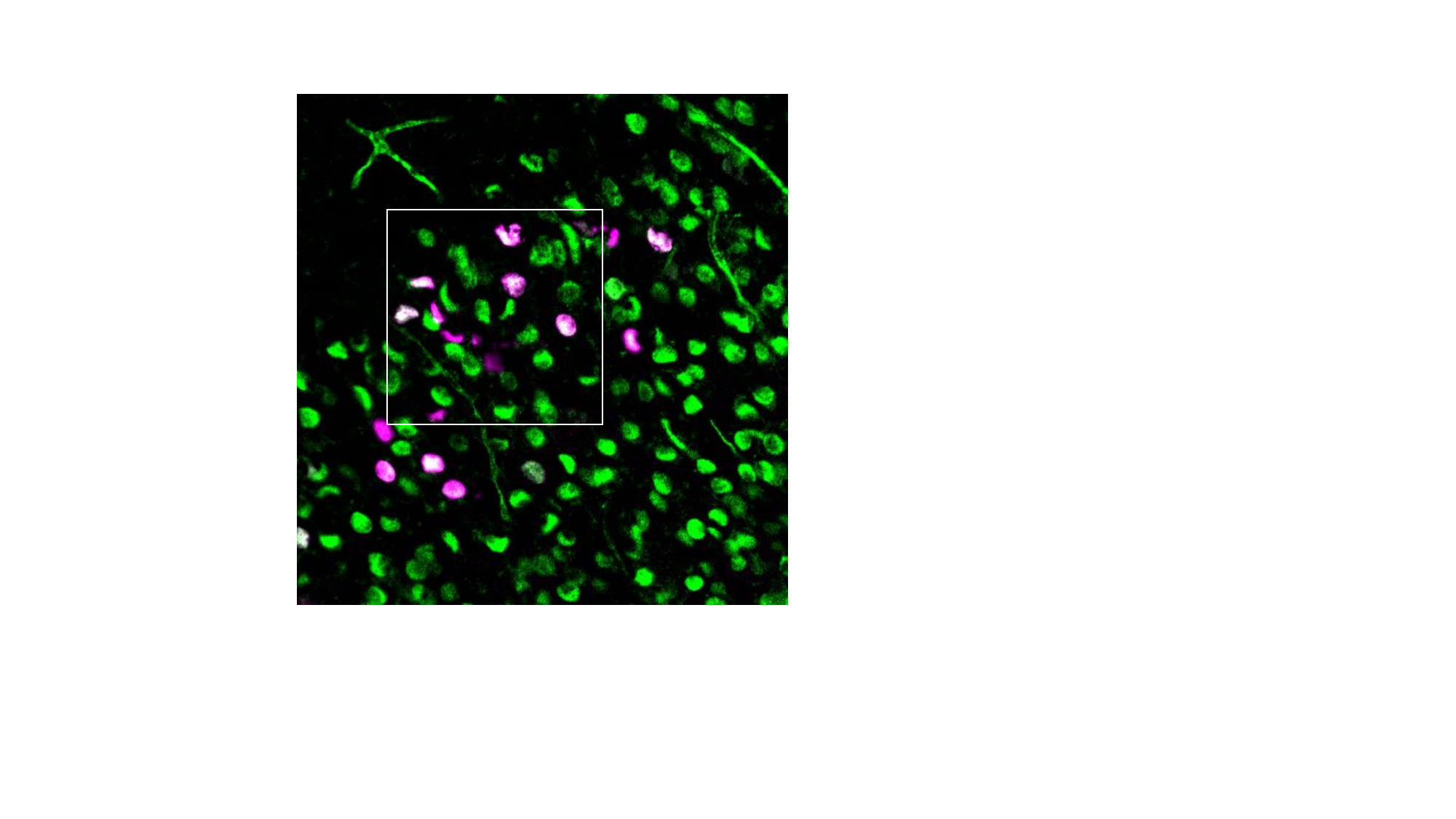

## Slide 2
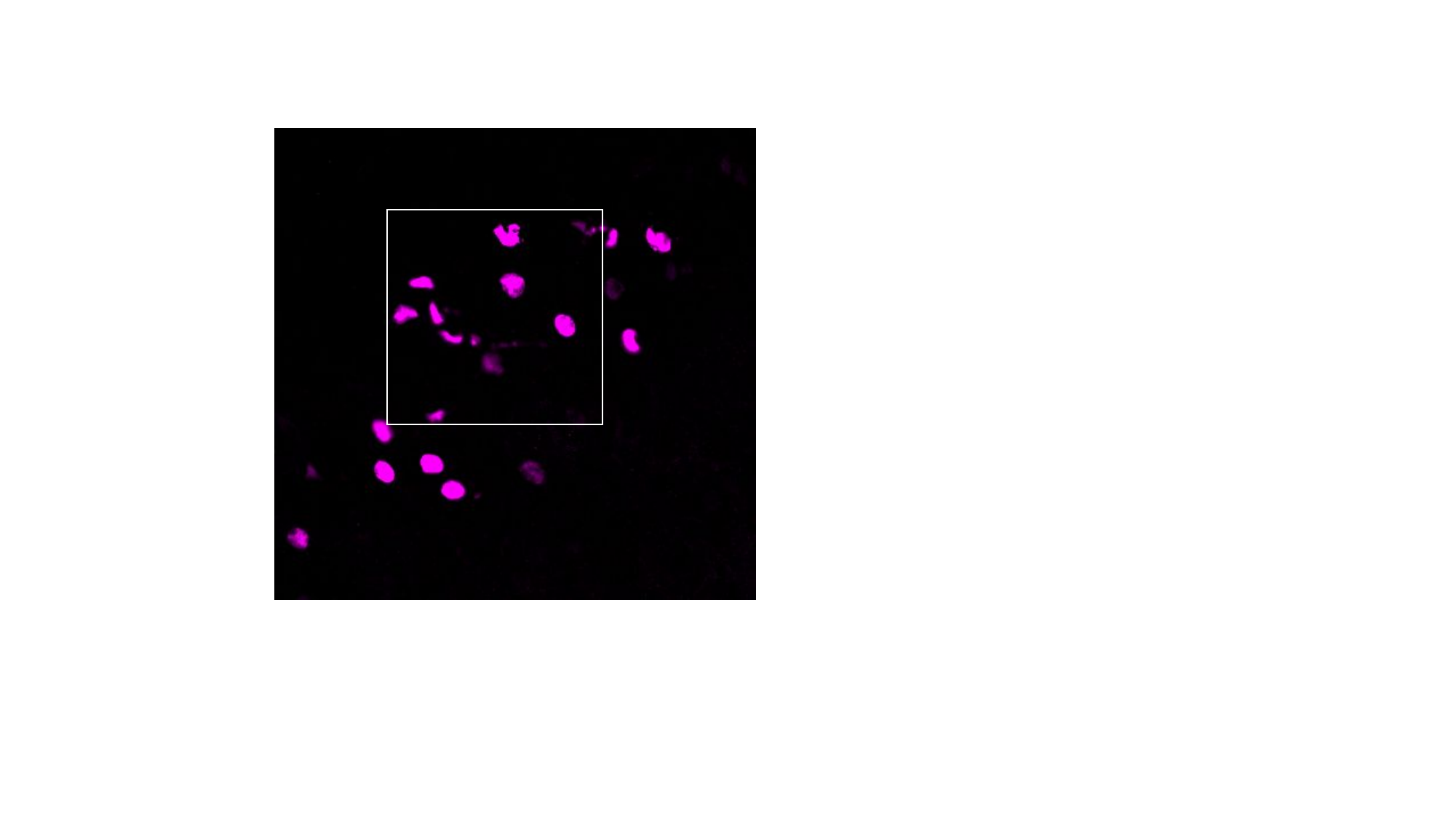

## Slide 3
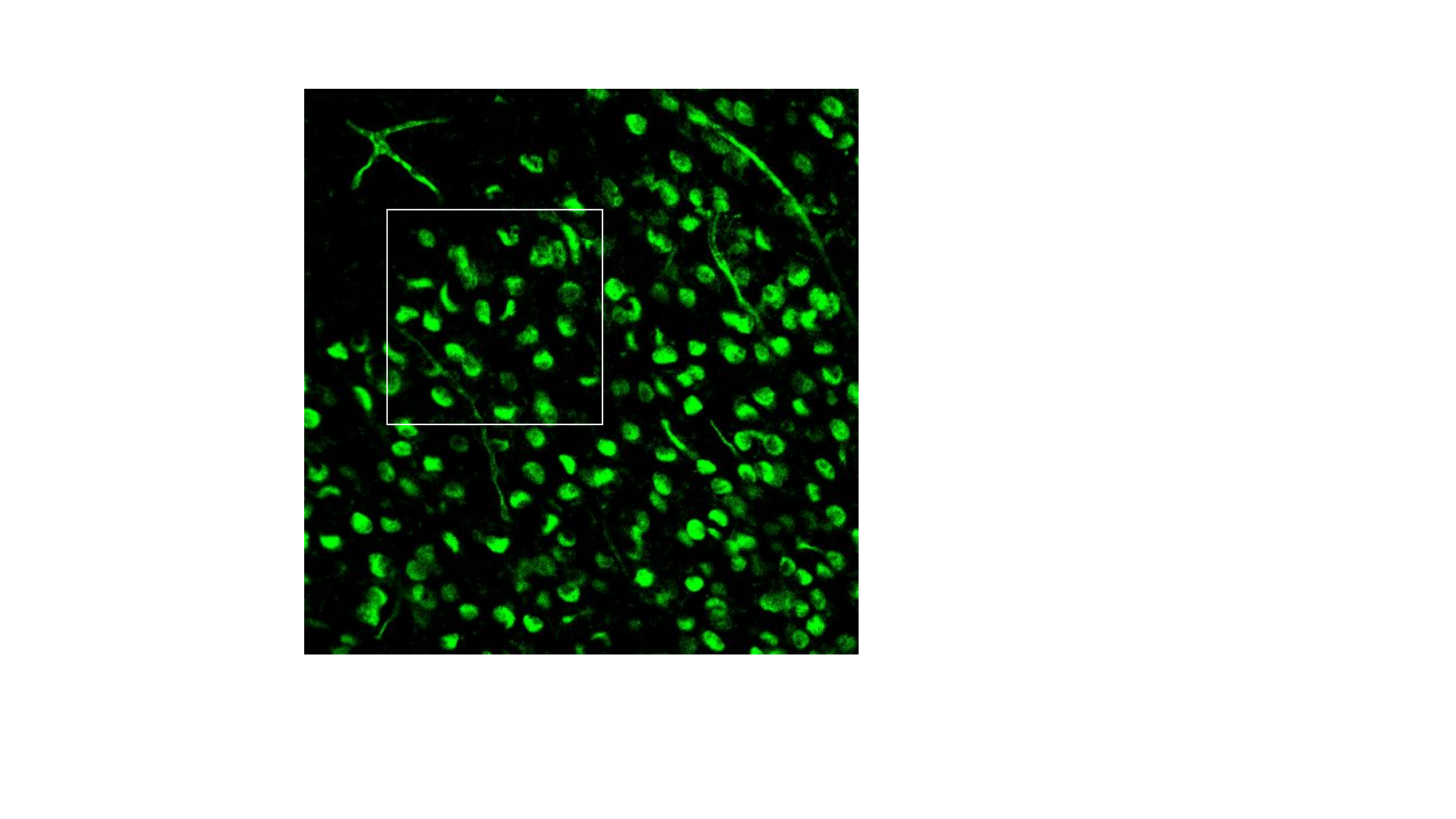

## Slide 4
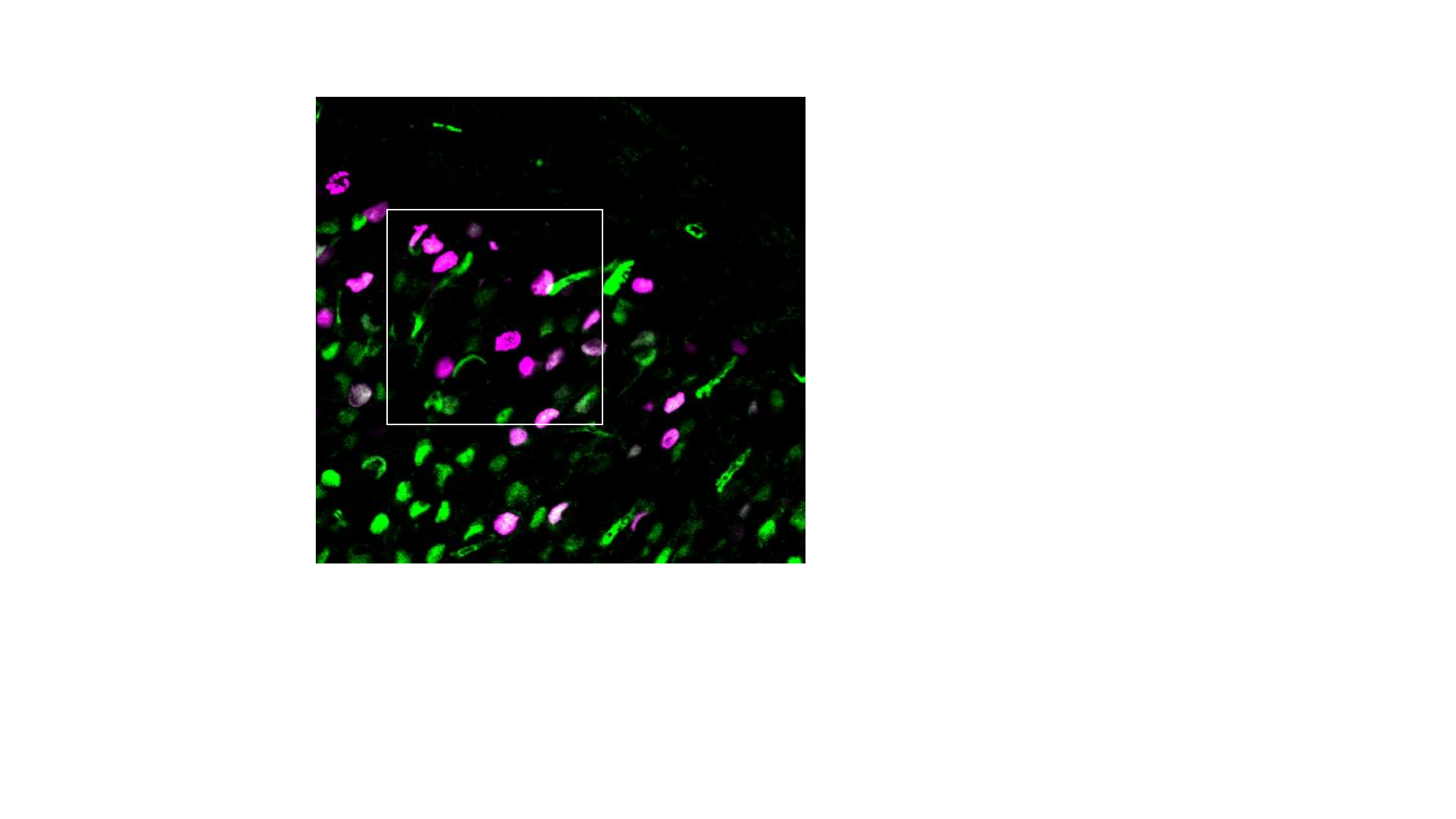

## Slide 5
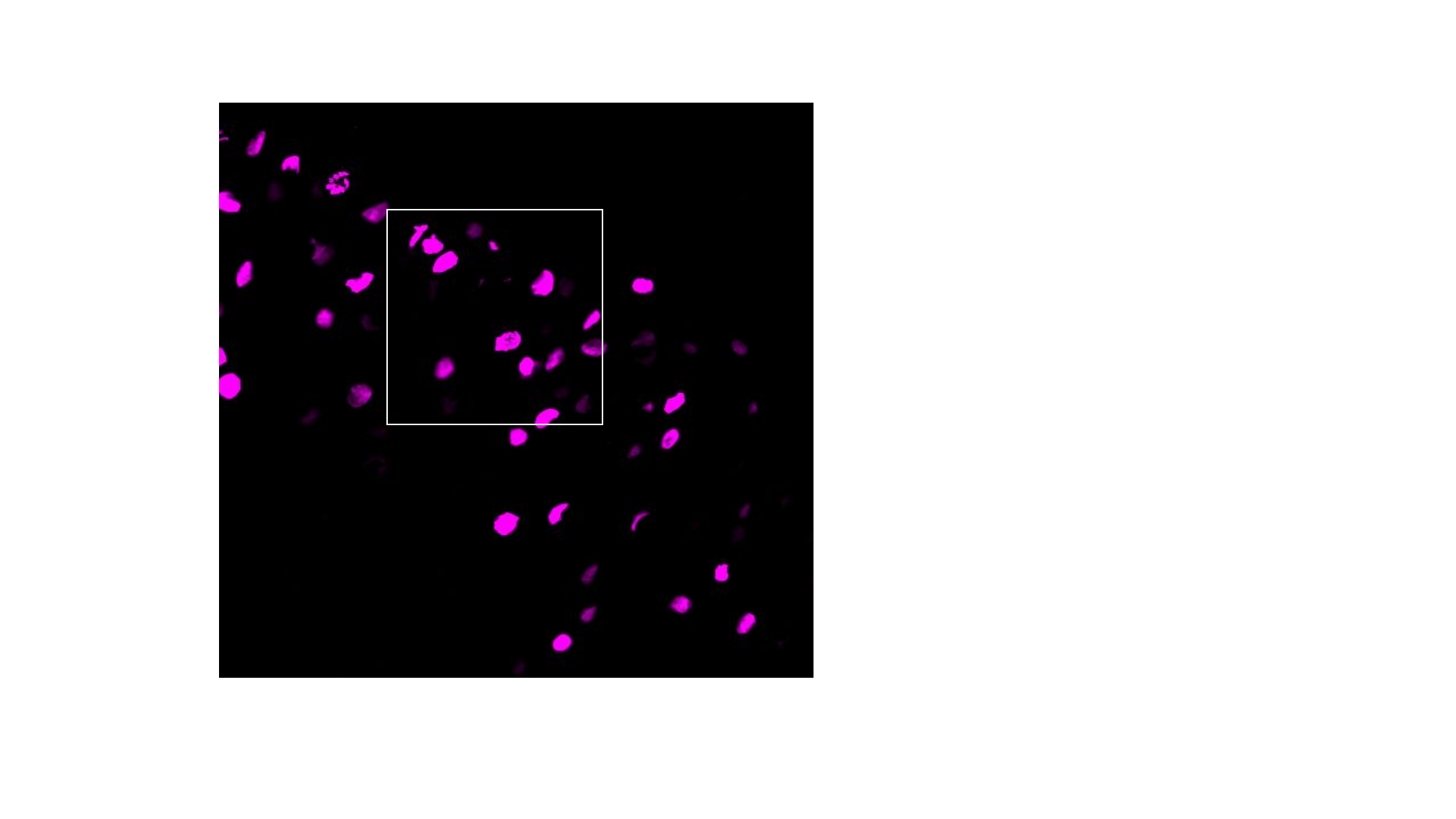

## Slide 6
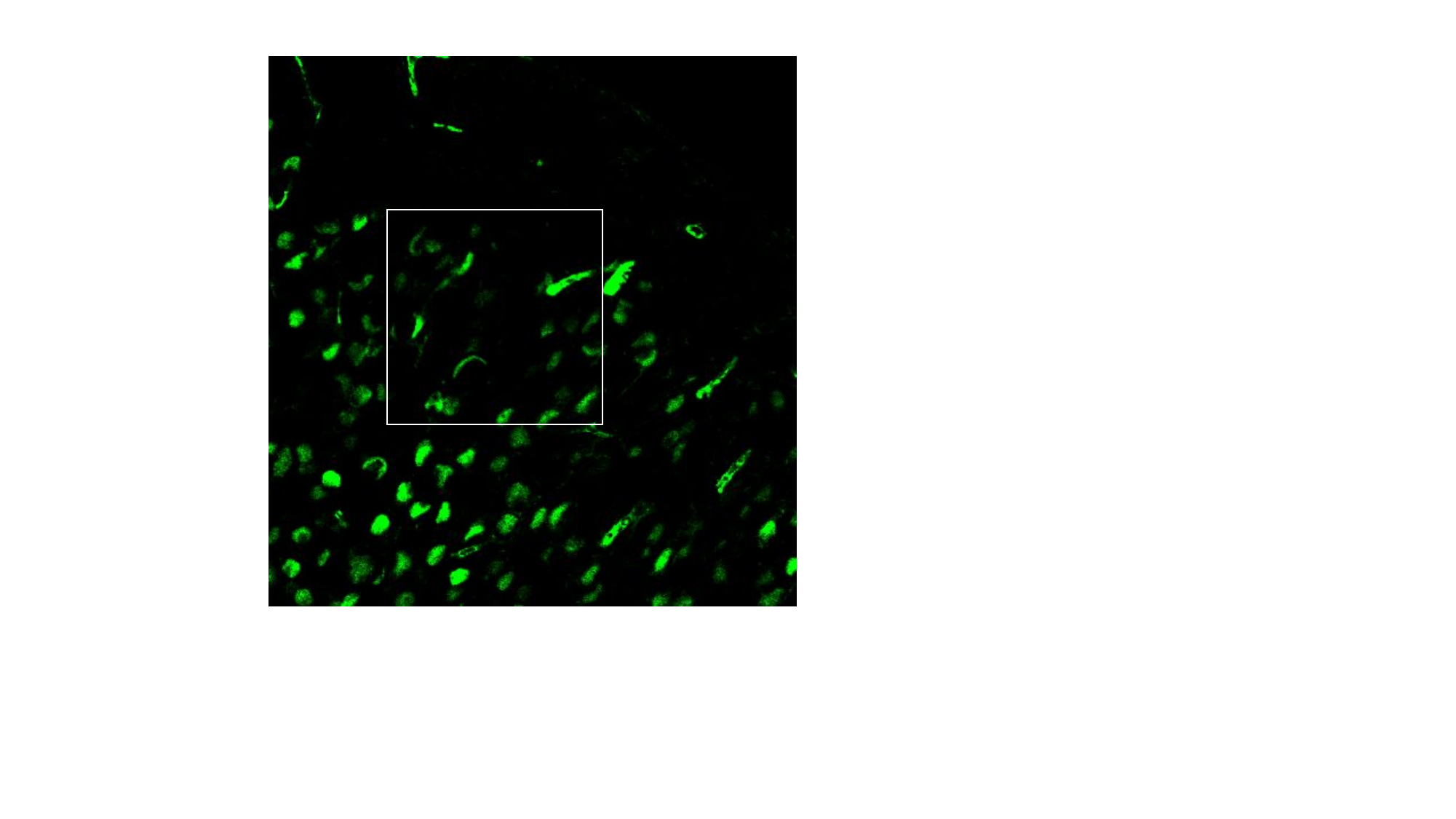

## Slide 7
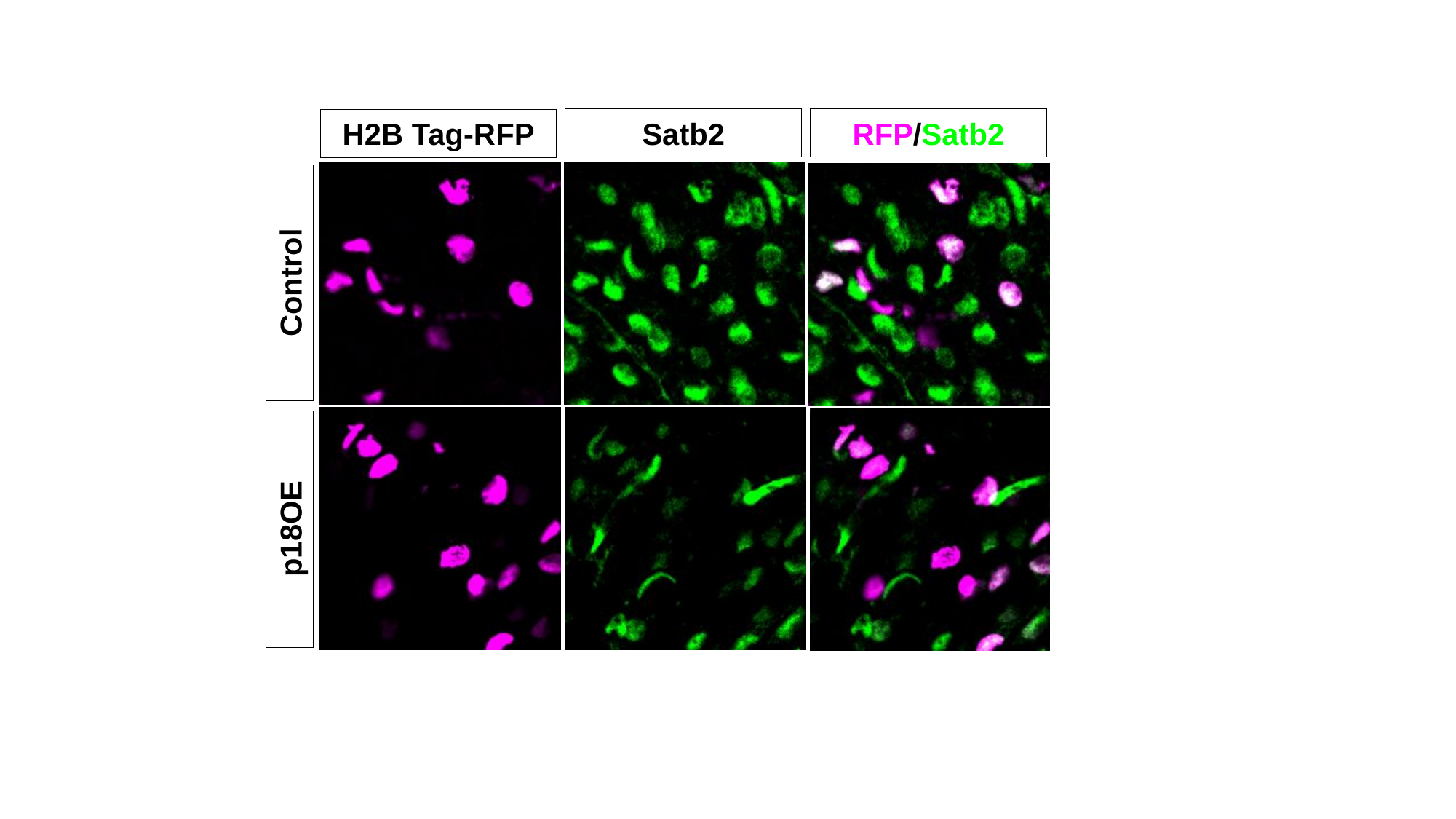

Satb2
RFP/Satb2
H2B Tag-RFP
Control
p18OE

## Slide 8
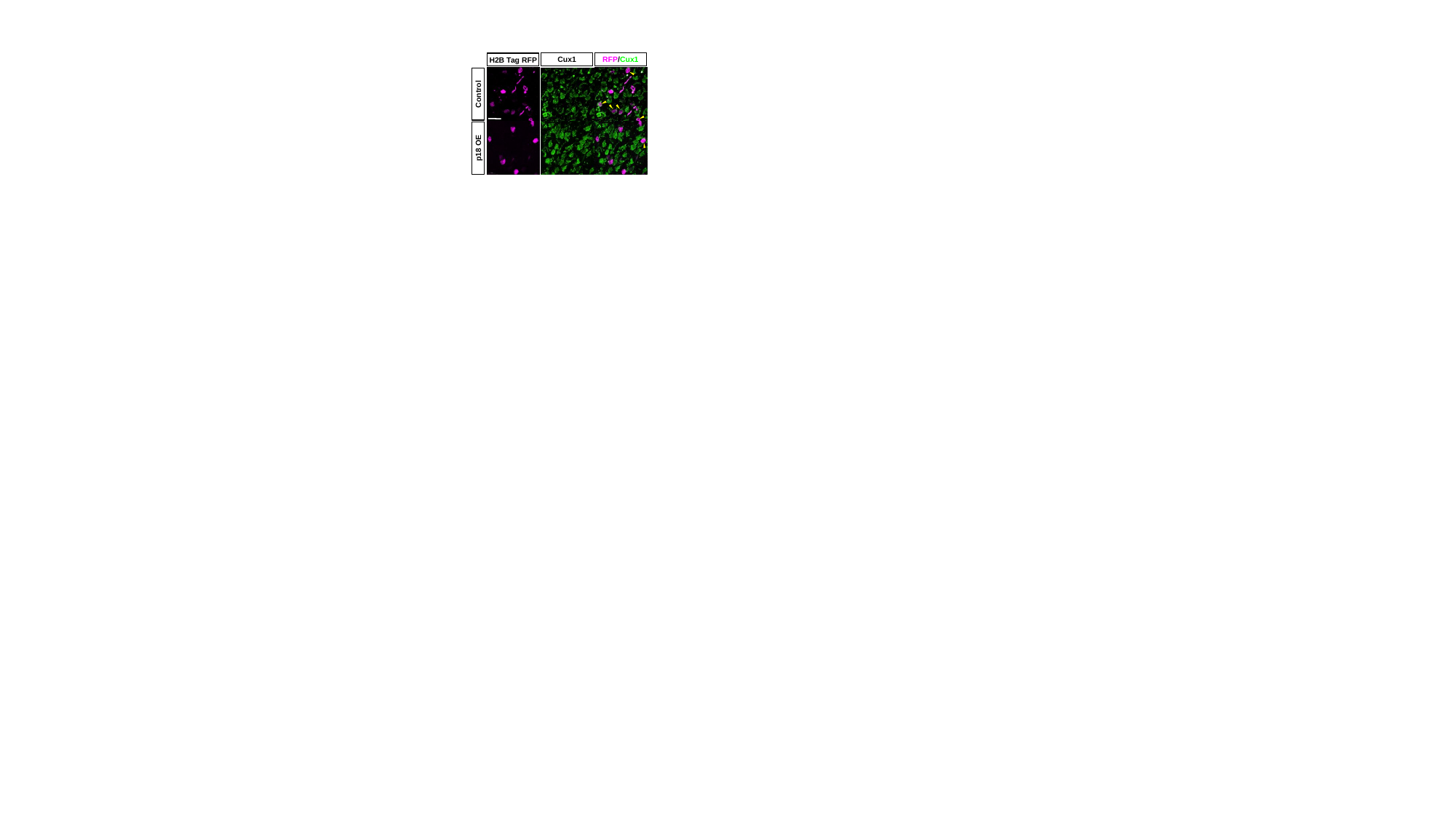

Cux1
RFP/Cux1
H2B Tag RFP
Control
p18 OE

## Slide 9
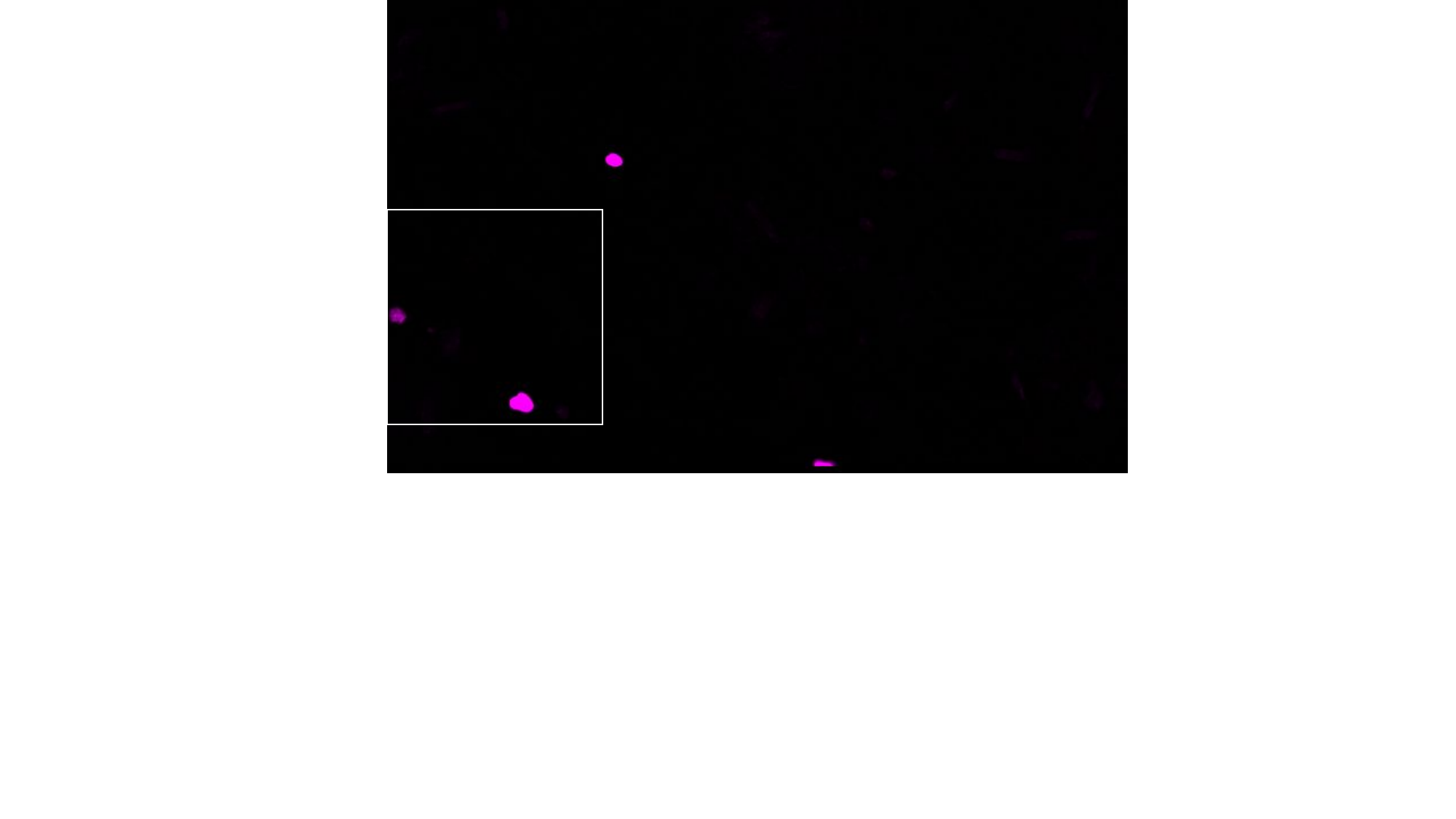

## Slide 10
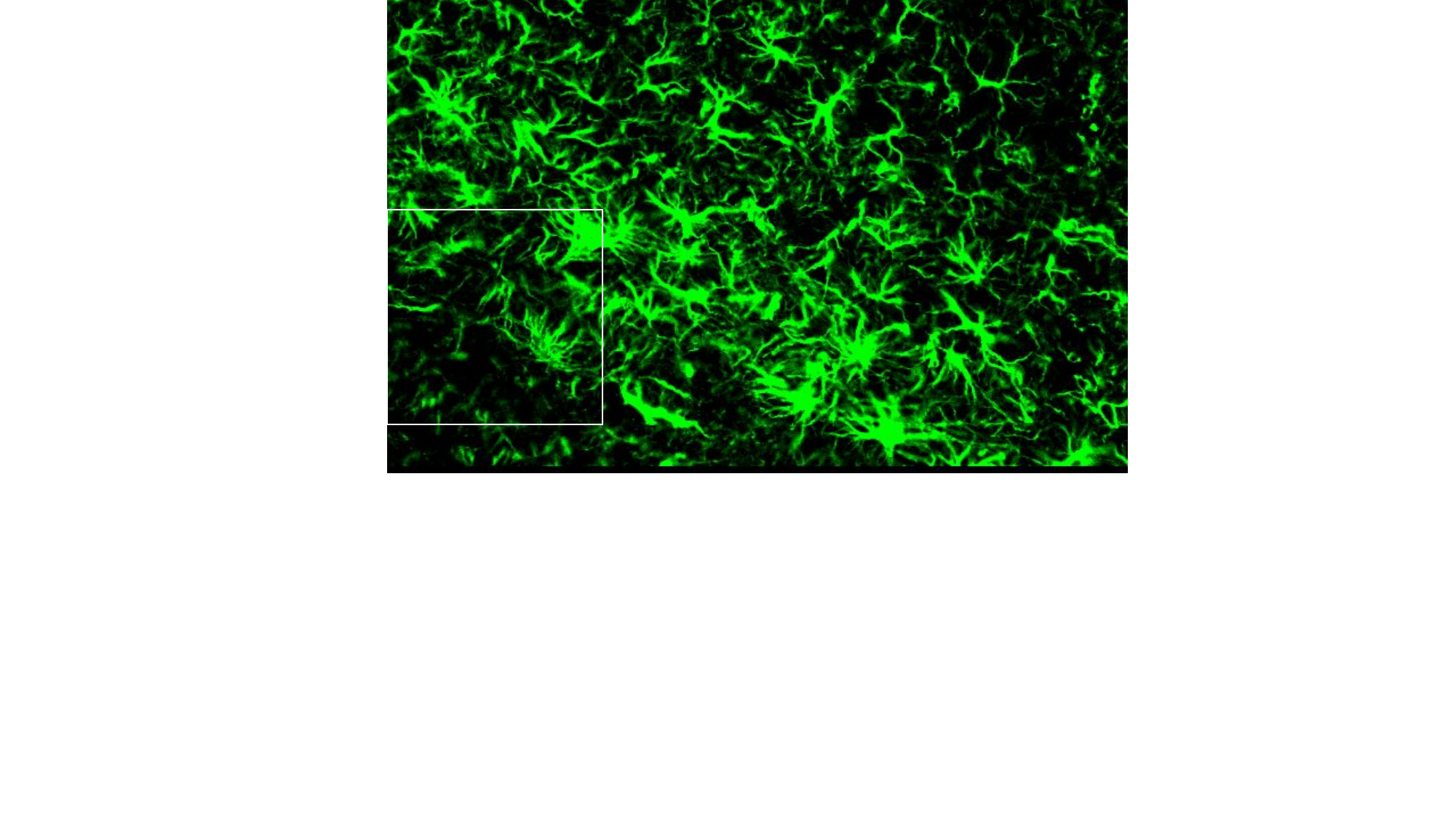

## Slide 11
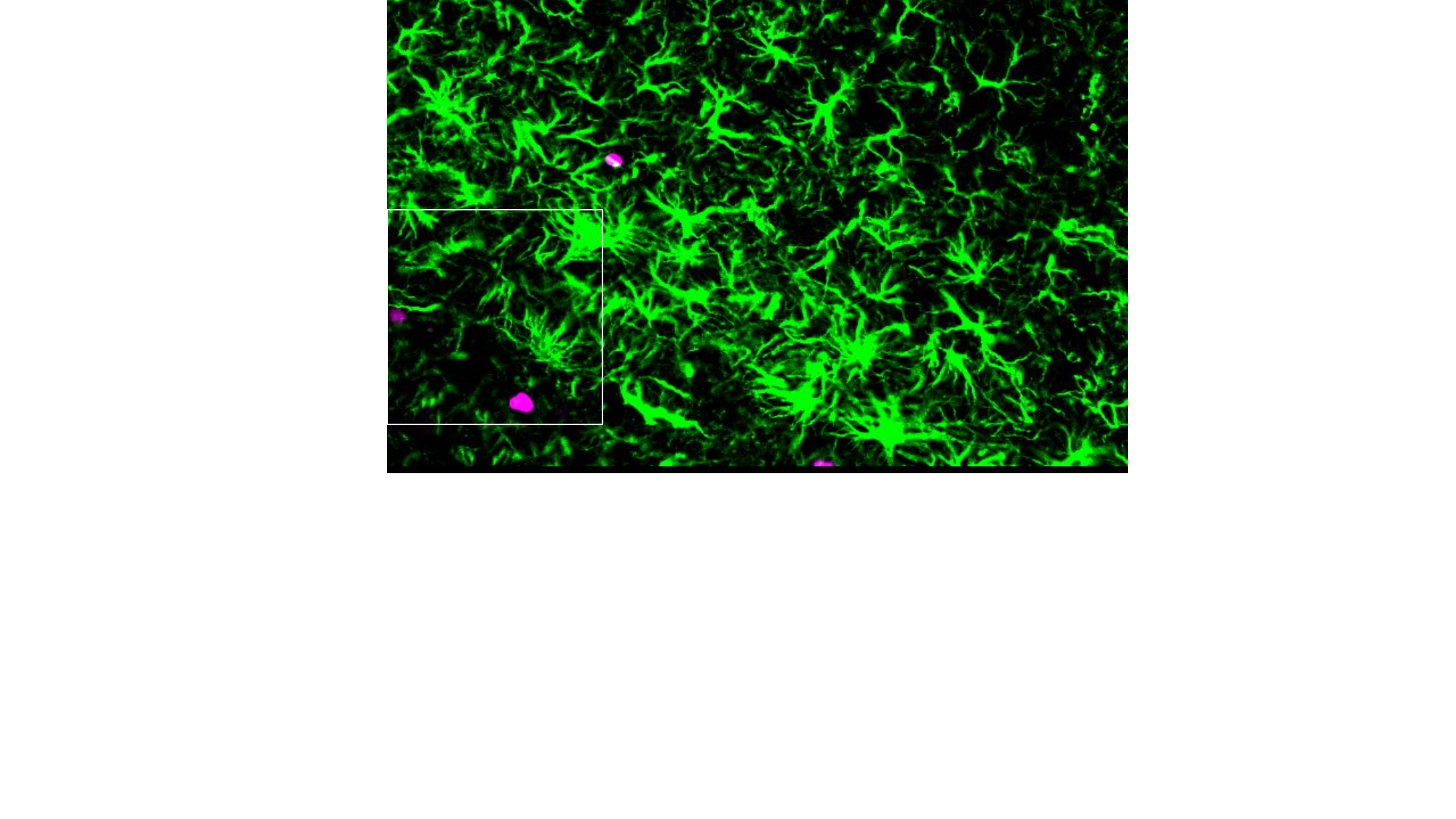

## Slide 12
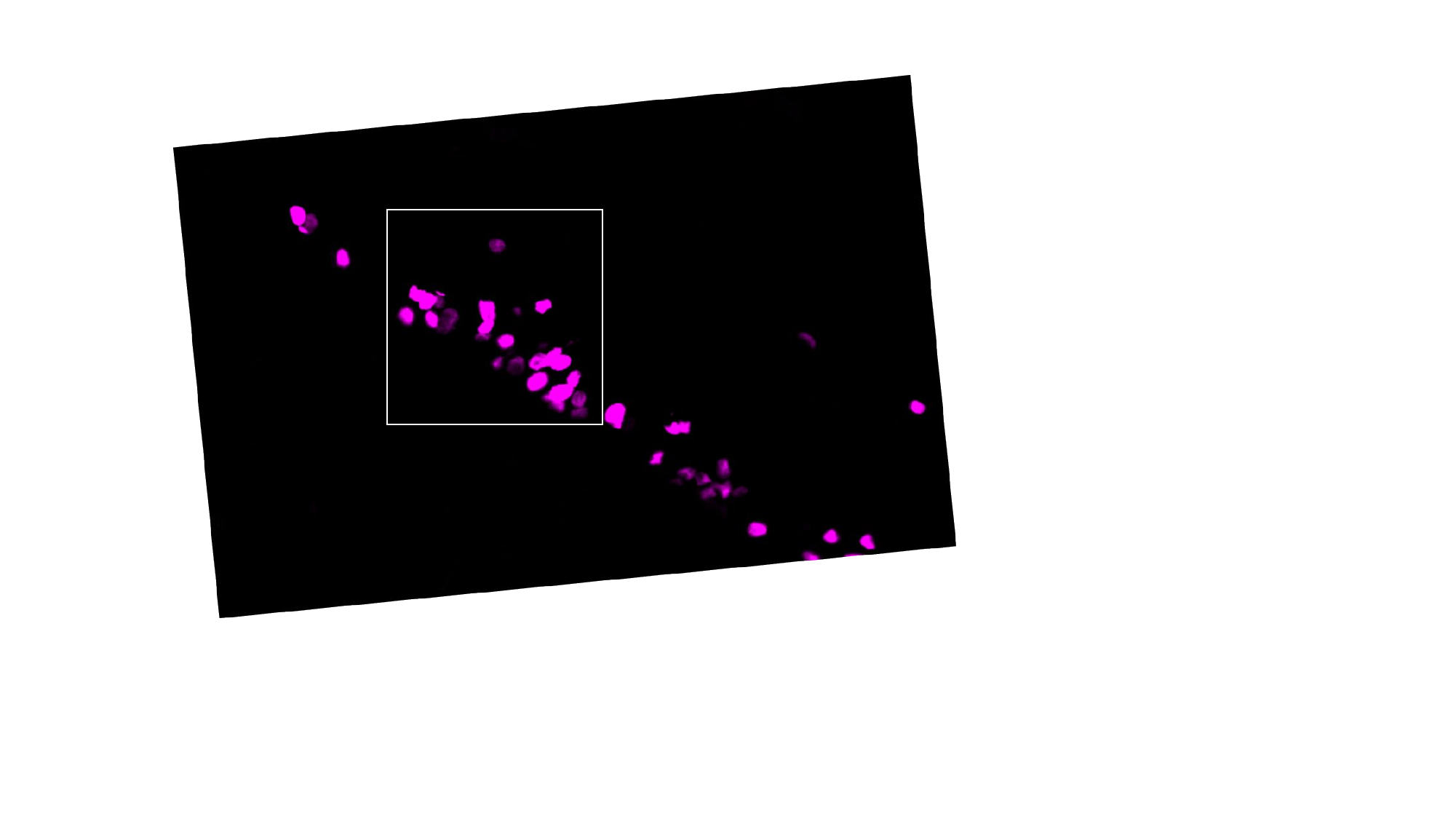

## Slide 13
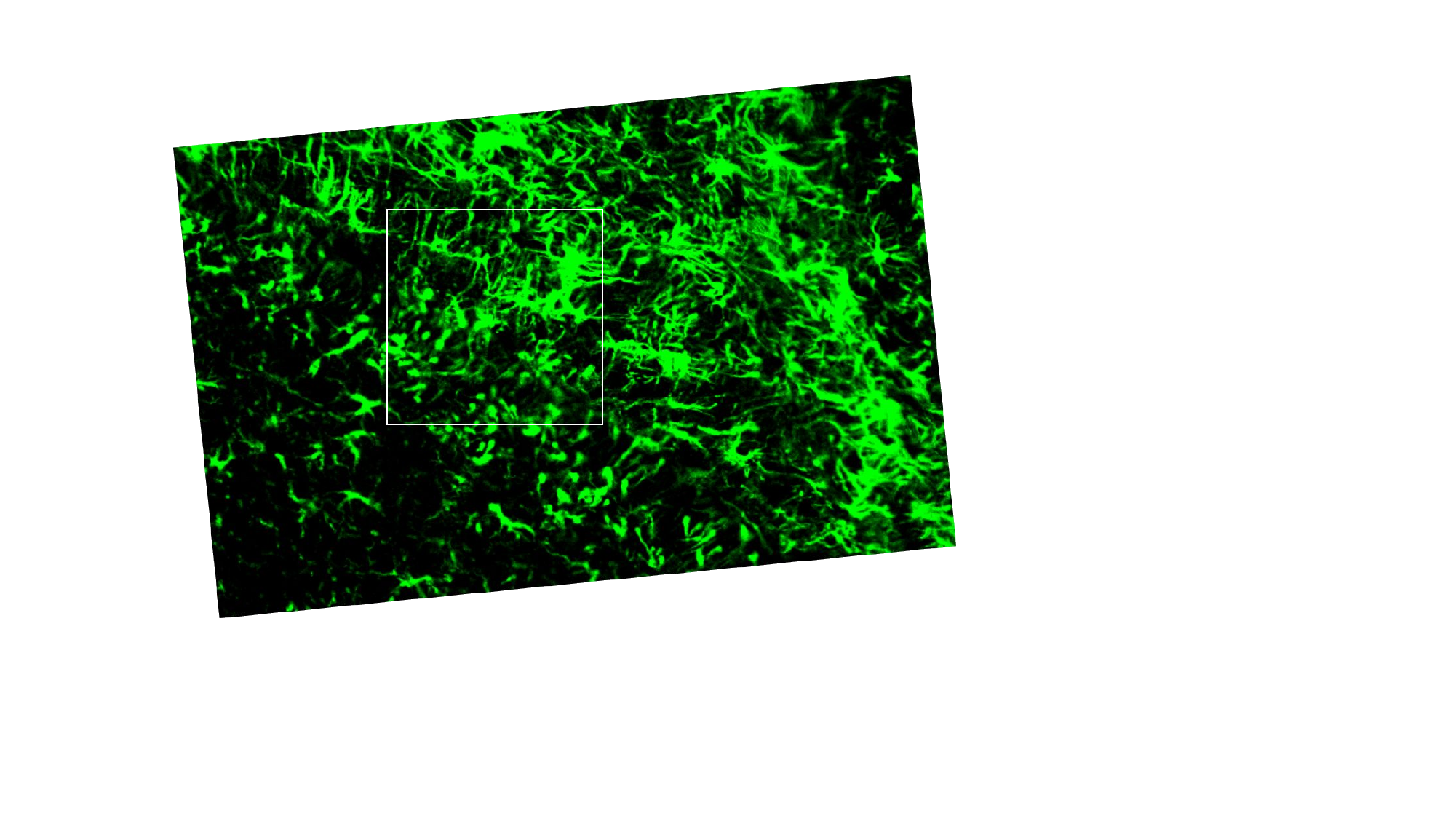

## Slide 14
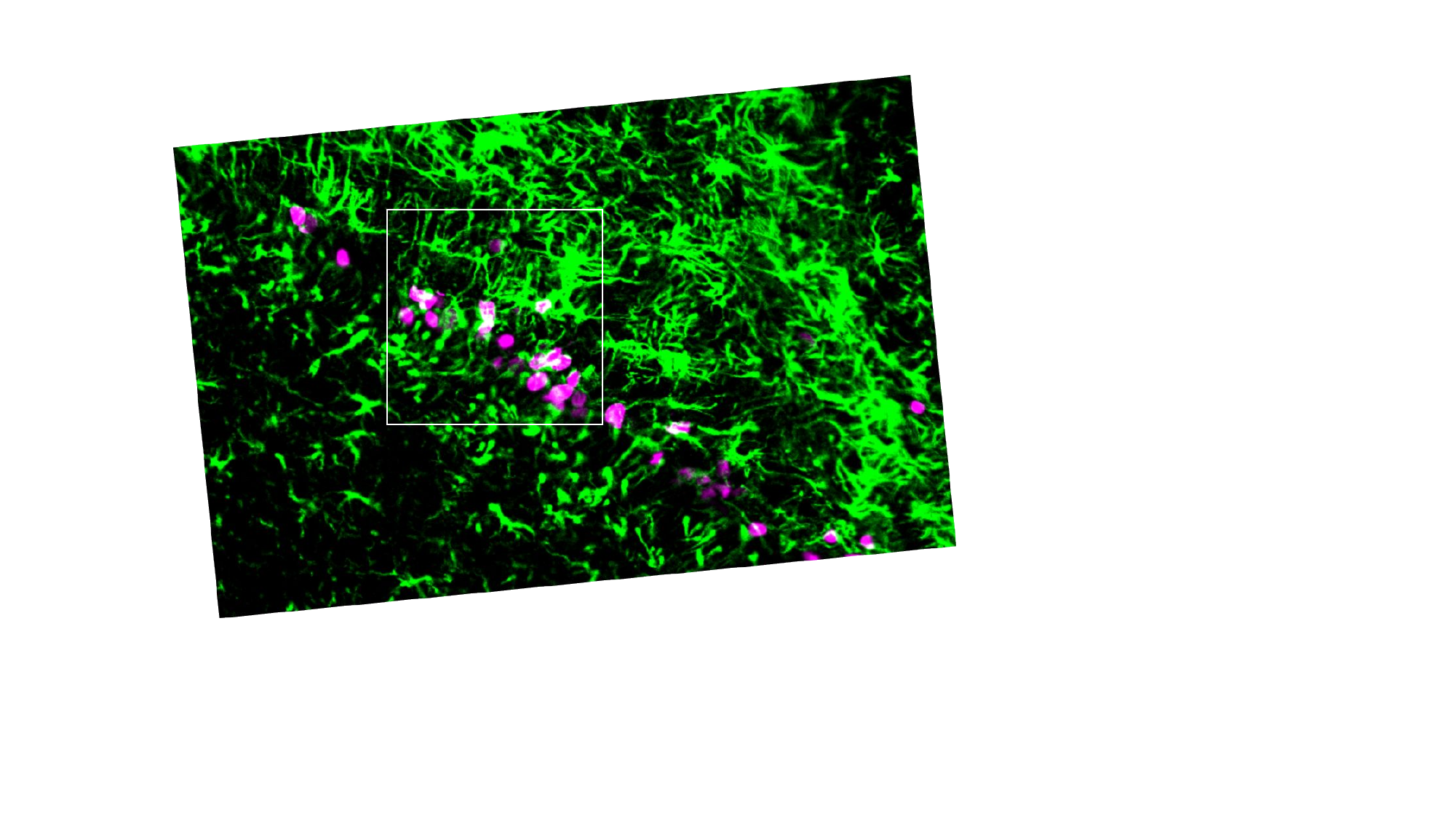

## Slide 15
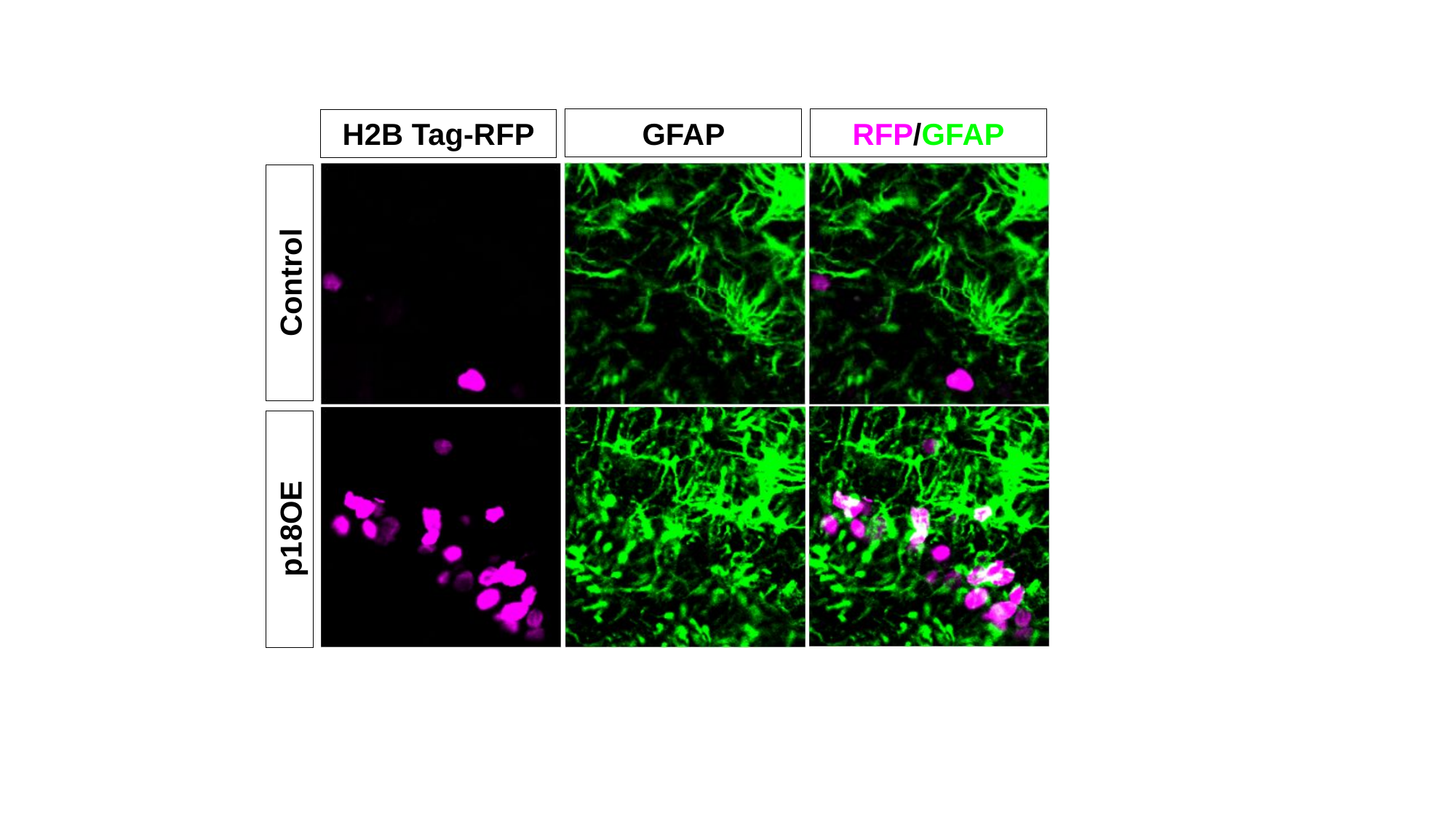

GFAP
RFP/GFAP
H2B Tag-RFP
Control
p18OE

## Slide 16
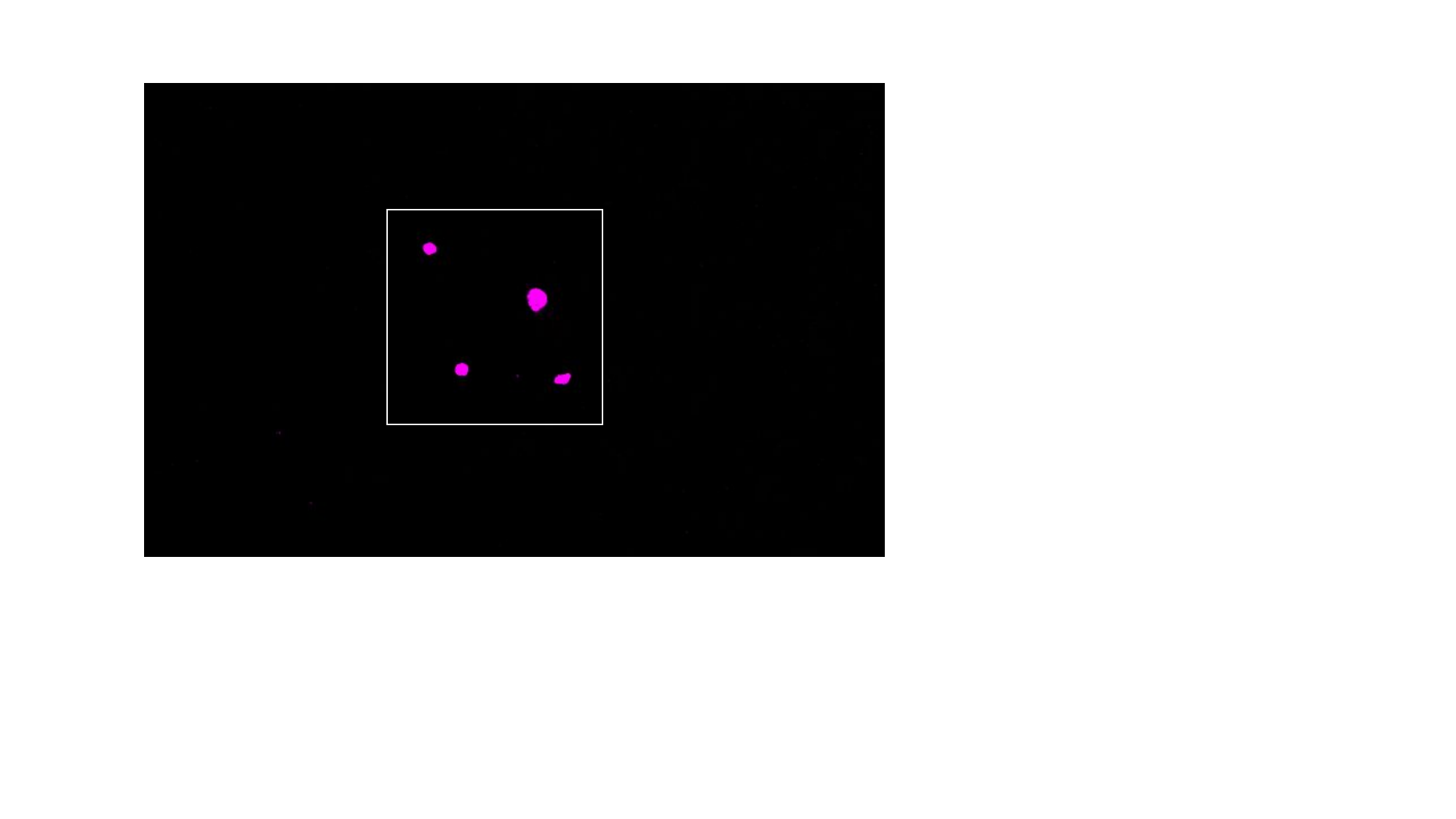

## Slide 17
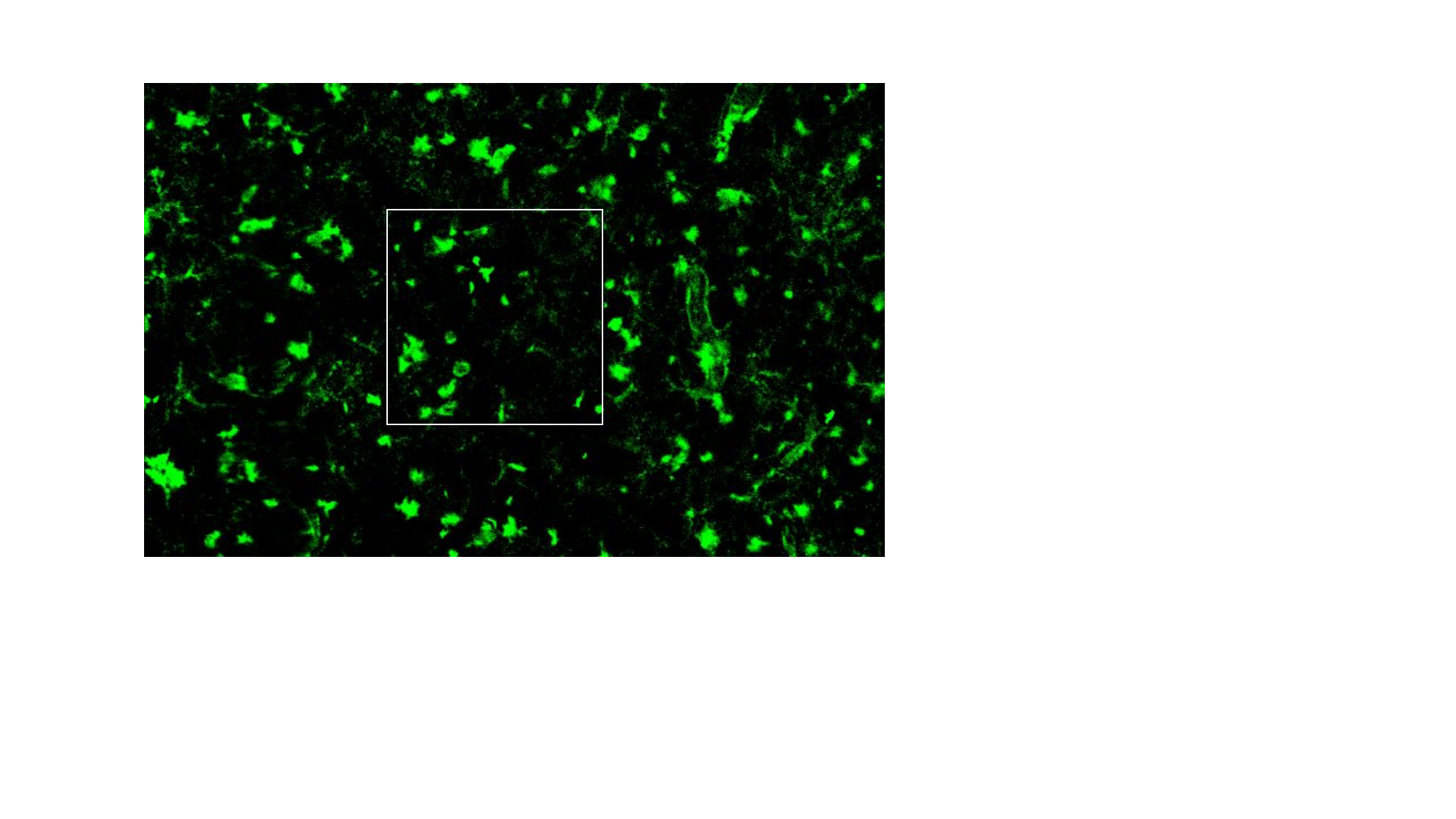

## Slide 18
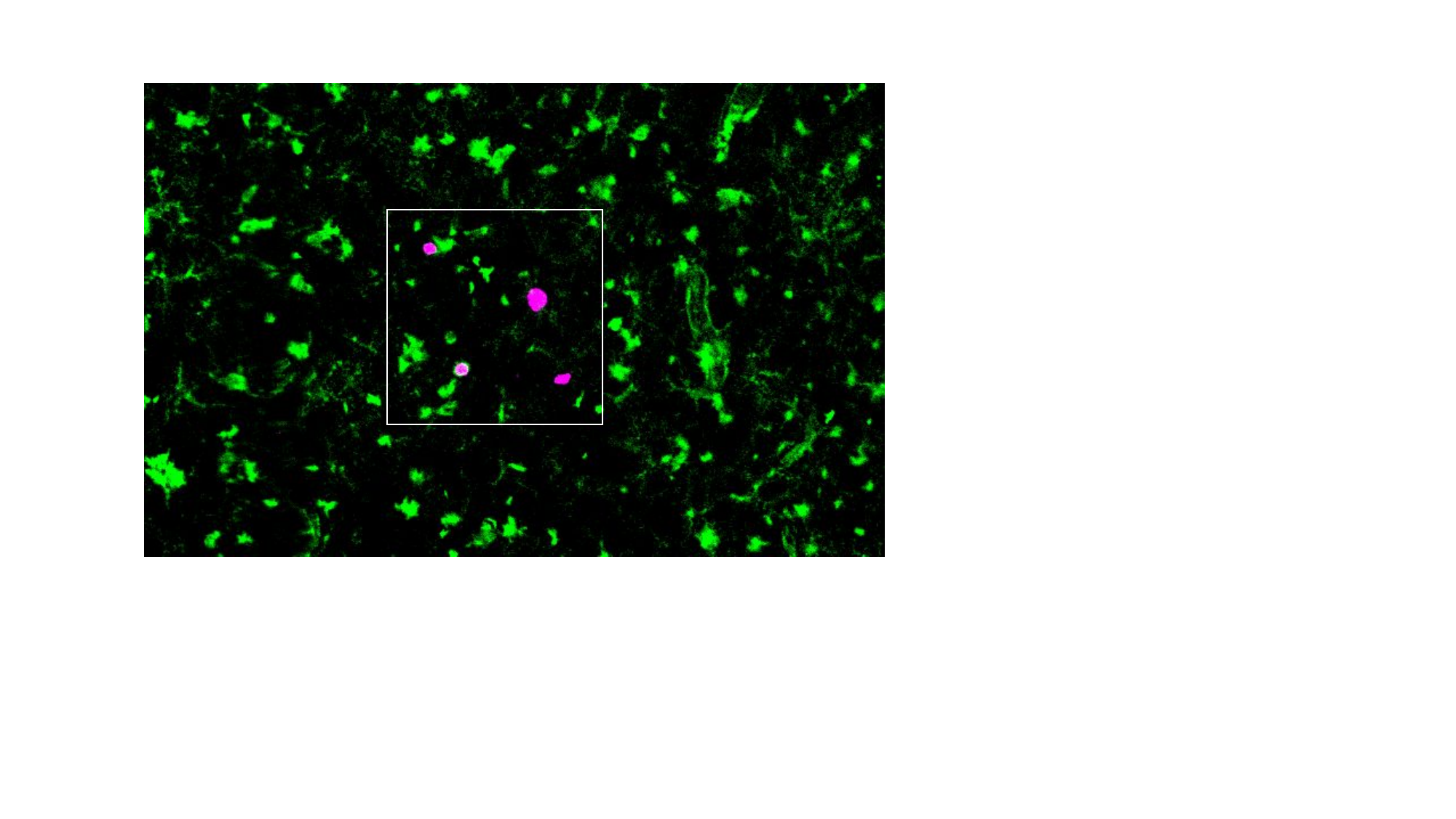

## Slide 19
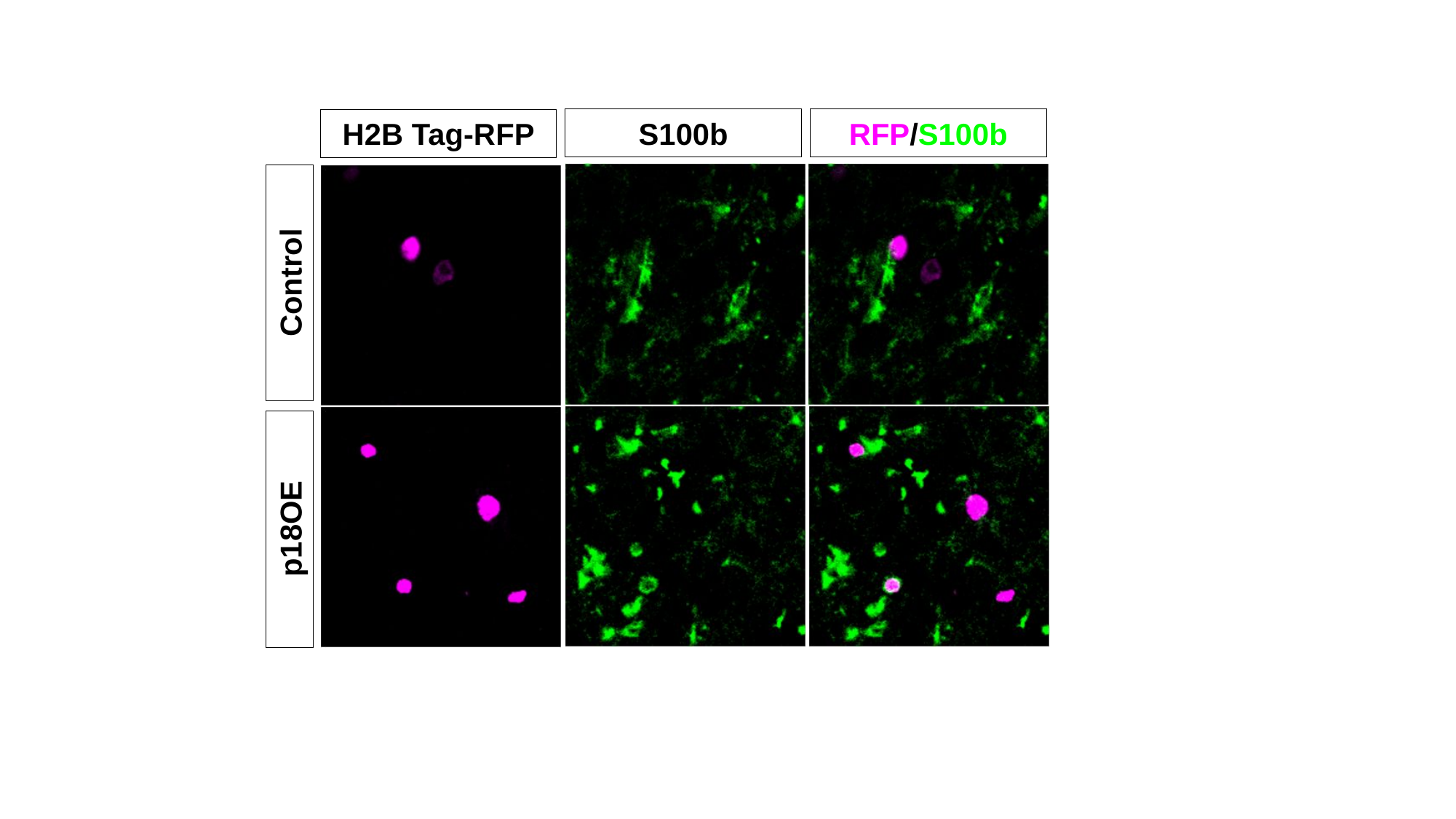

S100b
RFP/S100b
H2B Tag-RFP
Control
p18OE
