# Supplementary material for: Cyclin-dependent kinase inhibitor p18 regulates lineage transitions of excitatory neurons, astrocytes, and interneurons in the mouse cortex
Source: EMBO J. 2024 Dec 12;44(2):382–412. doi: 10.1038/s44318-024-00325-9 (PMC11730326; doi:10.1038/s44318-024-00325-9)
Supplement: Supplementary file 7 — Source data Fig. 5 [file 44318_2024_325_MOESM7_ESM.zip › 5B.pptx]

## Slide 1
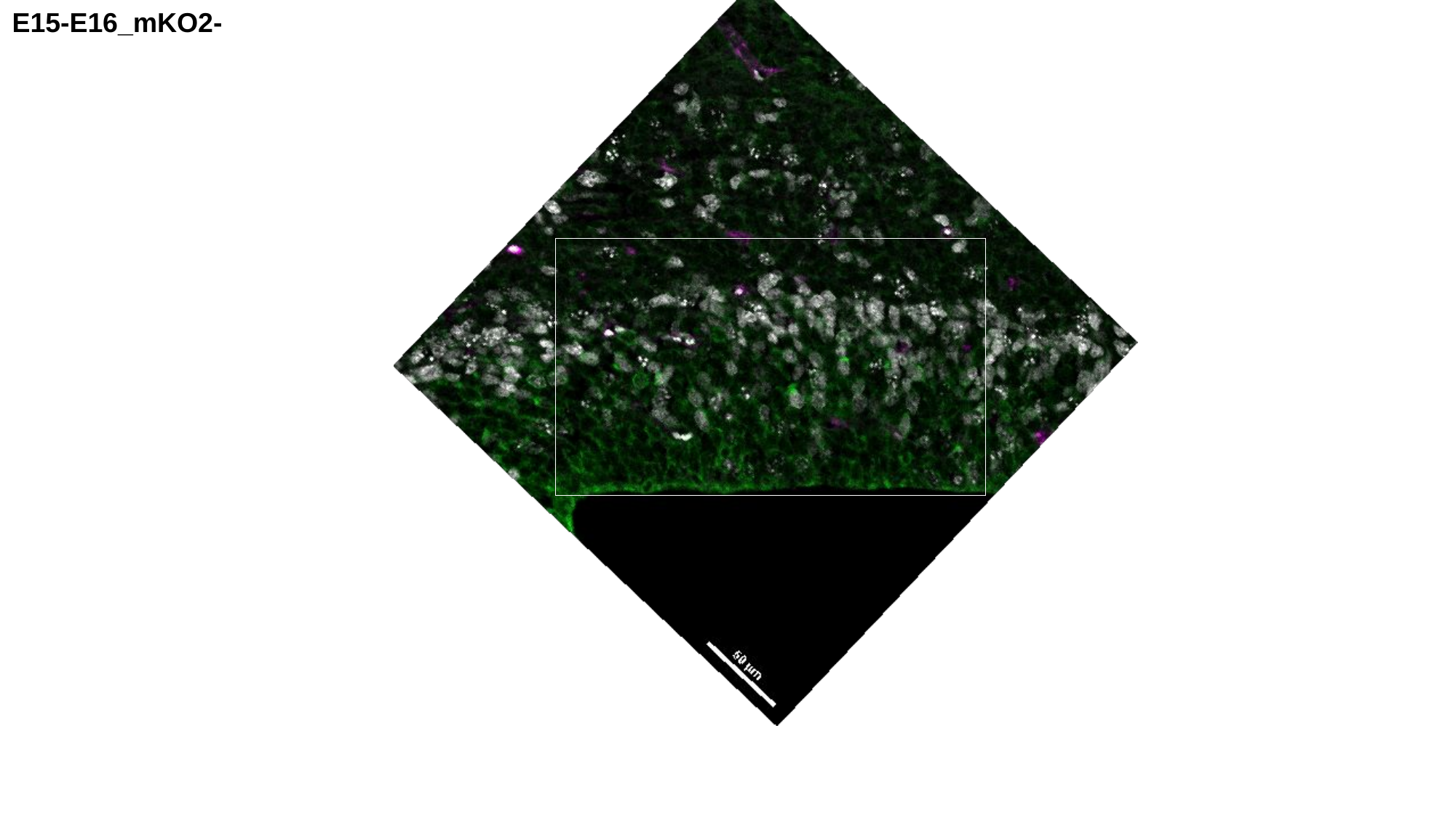

E15-E16_mKO2-

## Slide 2
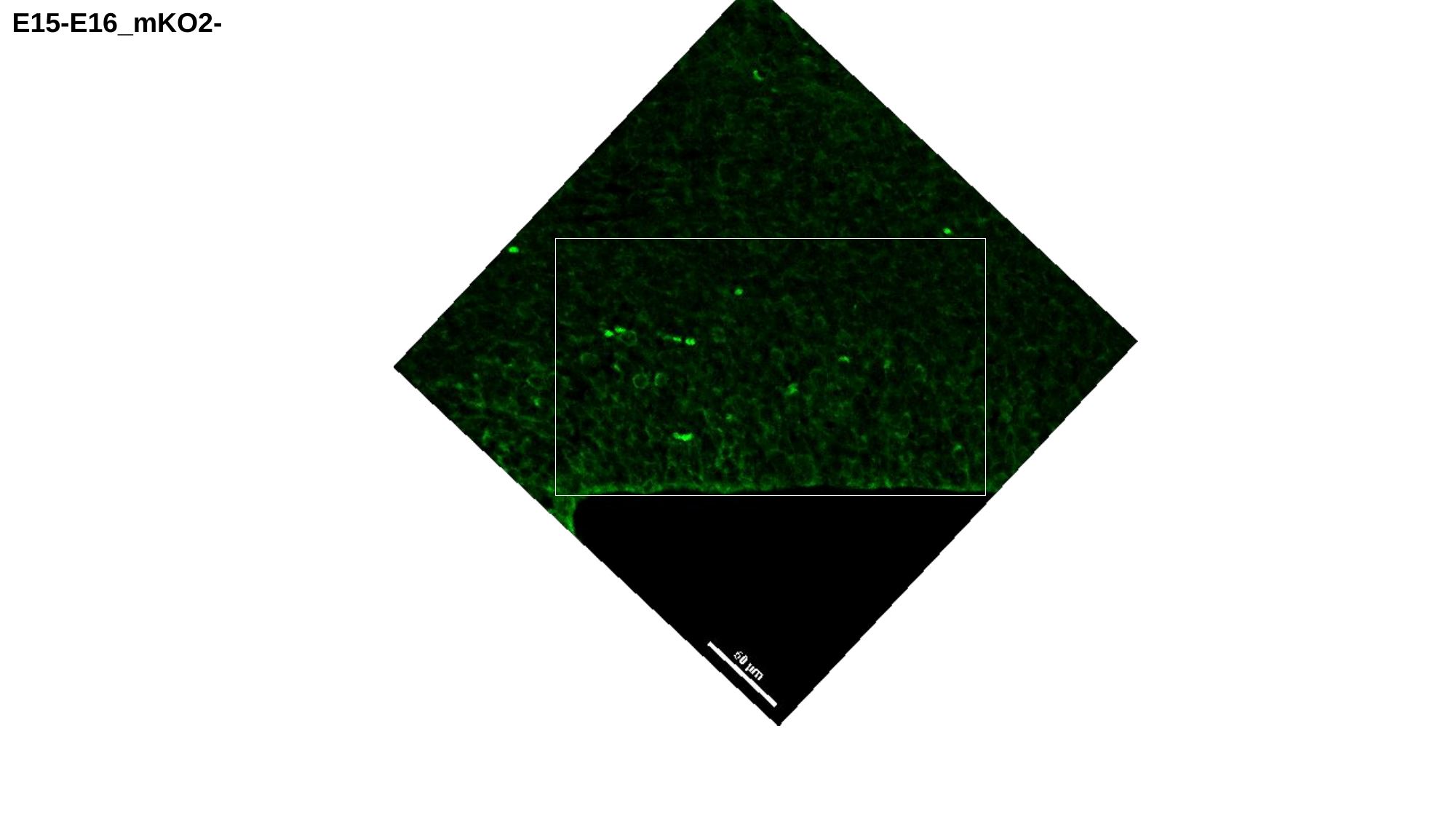

E15-E16_mKO2-

## Slide 3
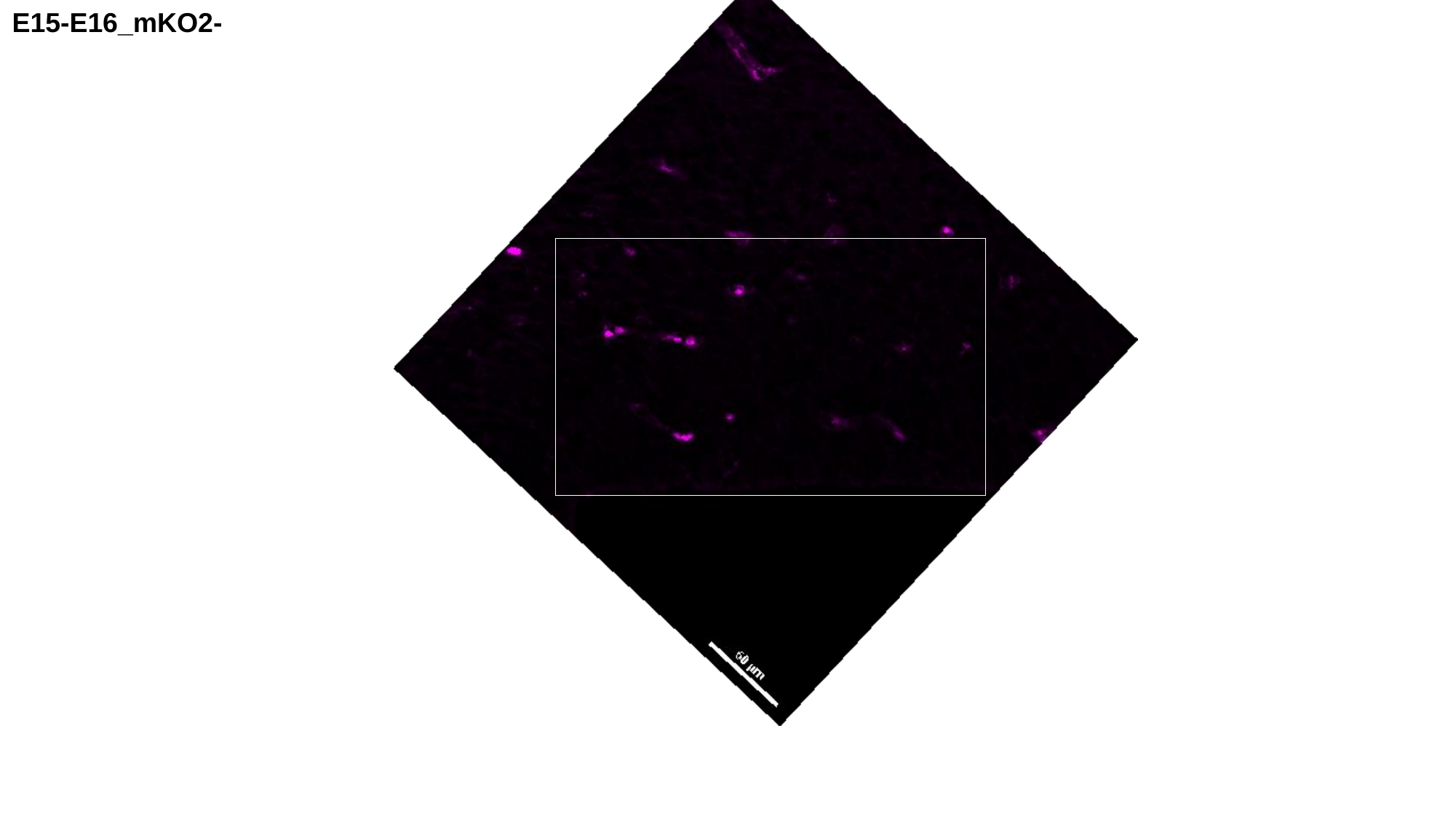

E15-E16_mKO2-

## Slide 4
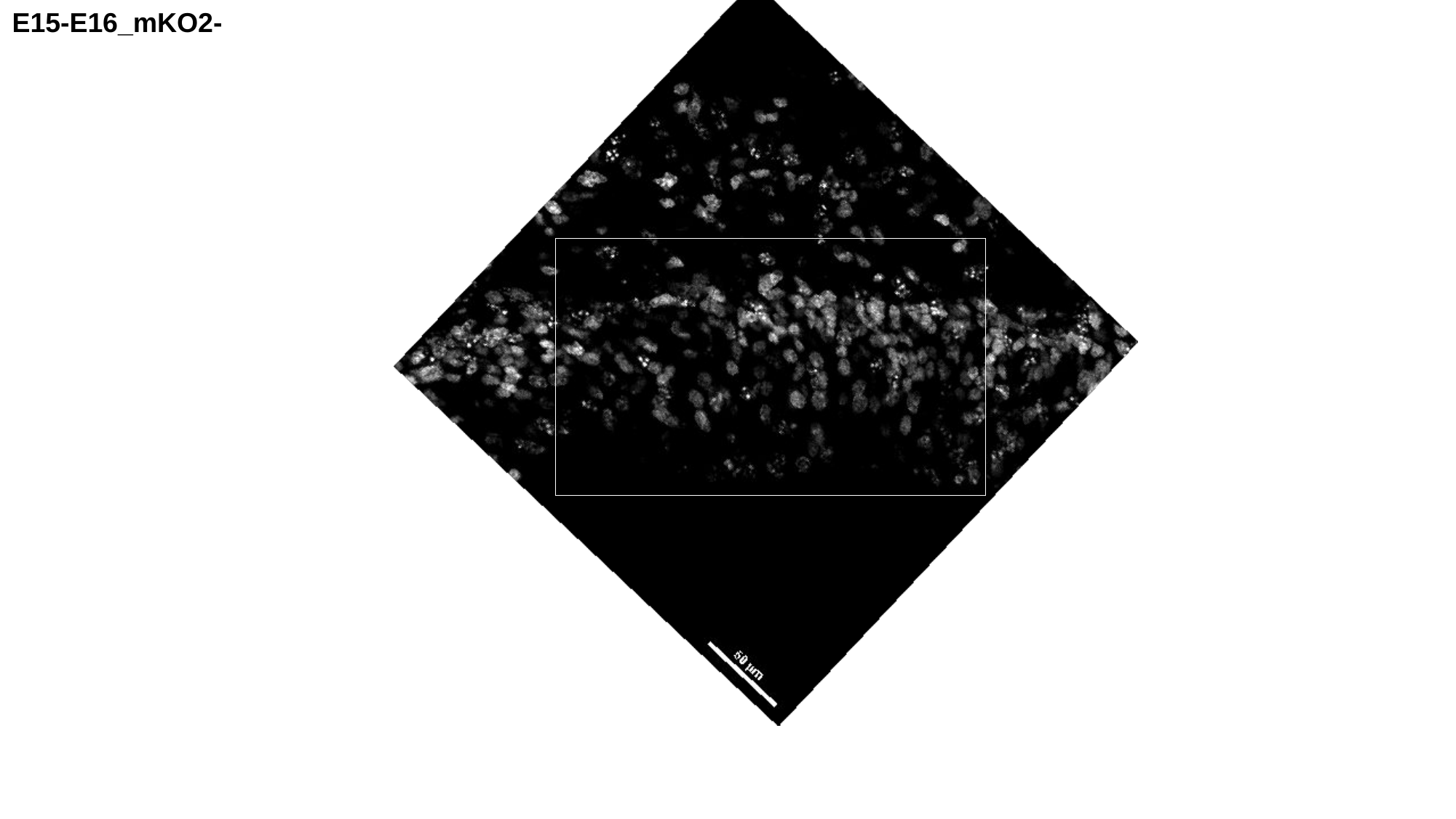

E15-E16_mKO2-

## Slide 5
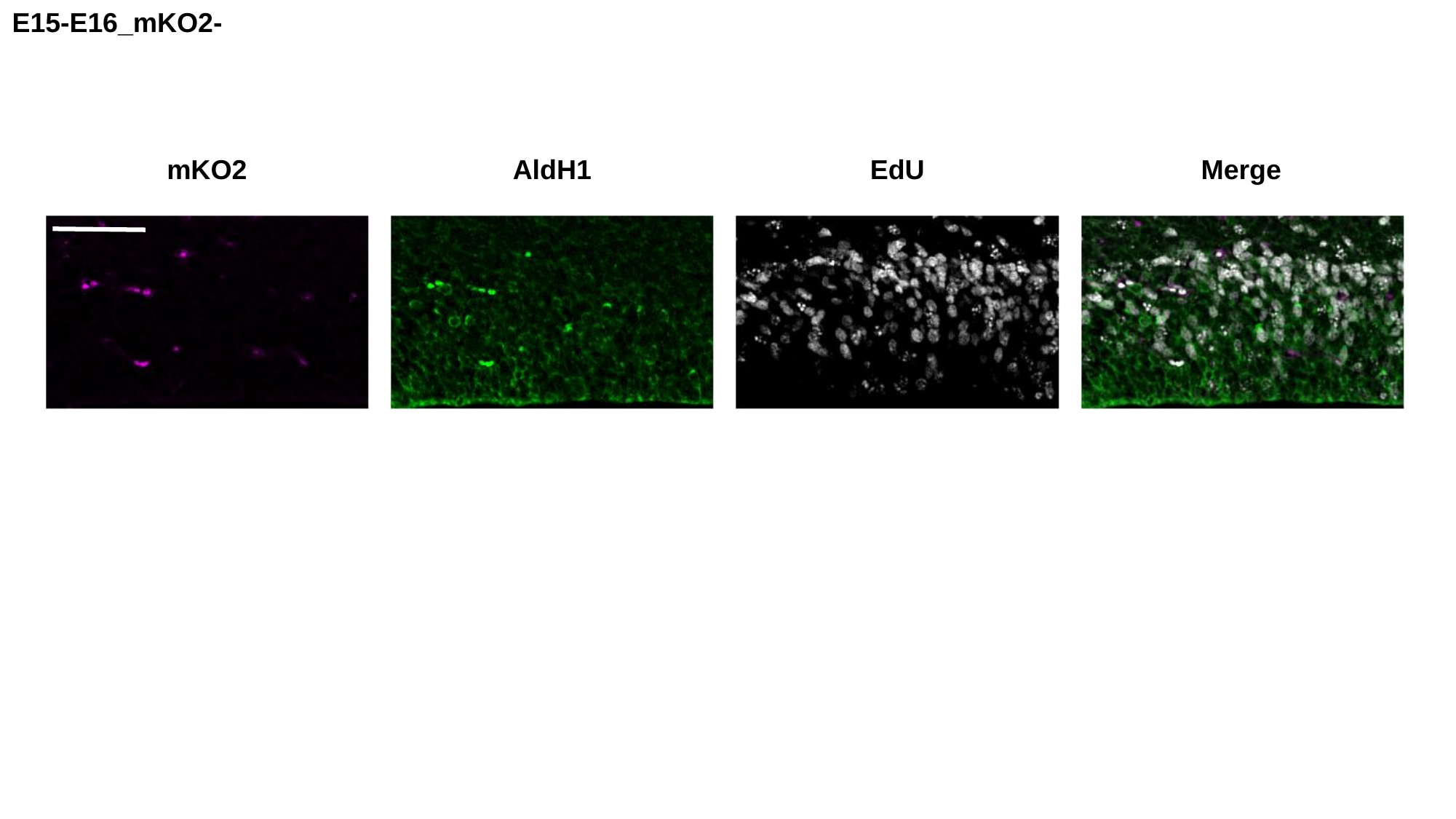

E15-E16_mKO2-
Merge
mKO2
AldH1
EdU

## Slide 6
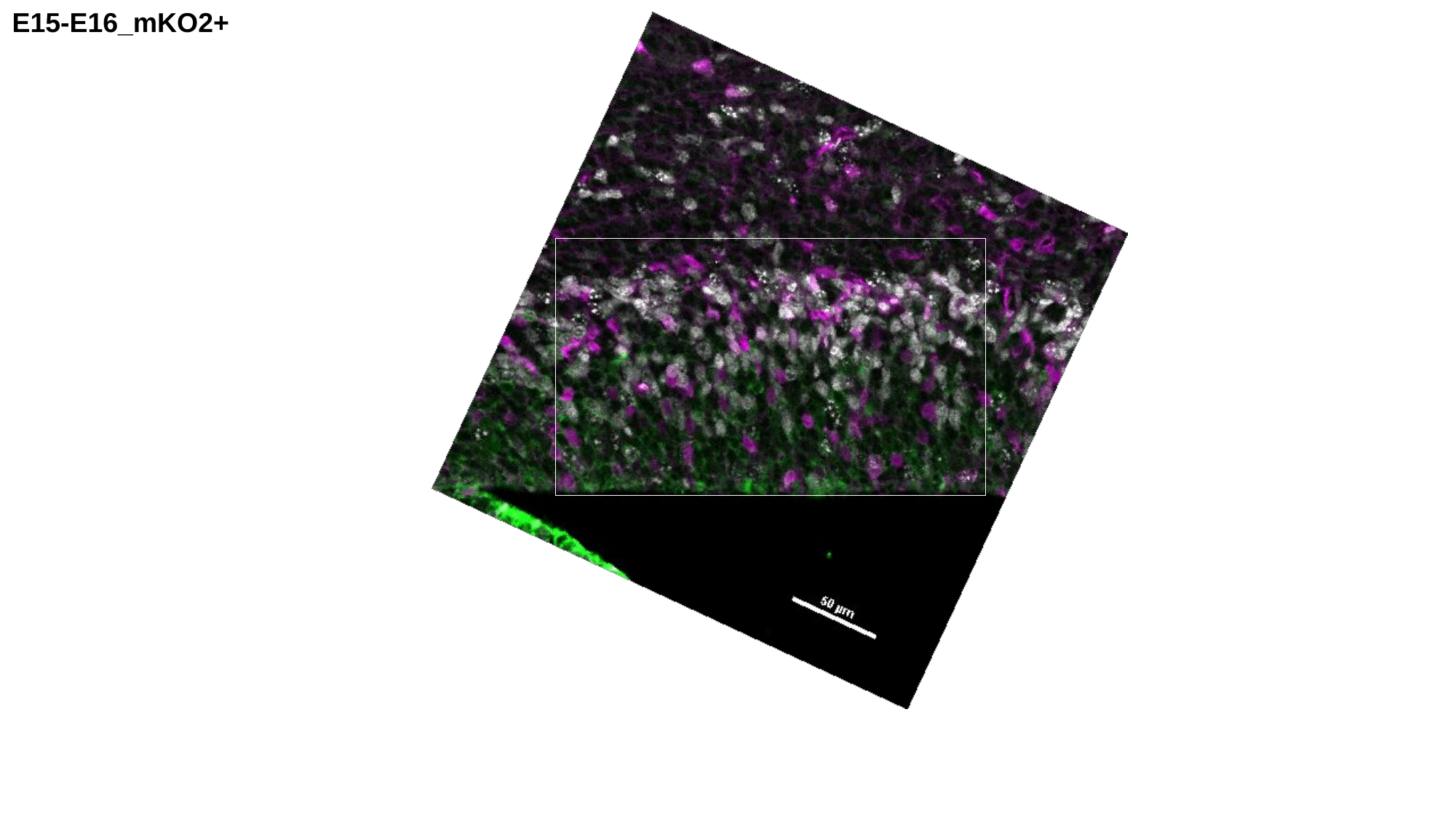

E15-E16_mKO2+

## Slide 7
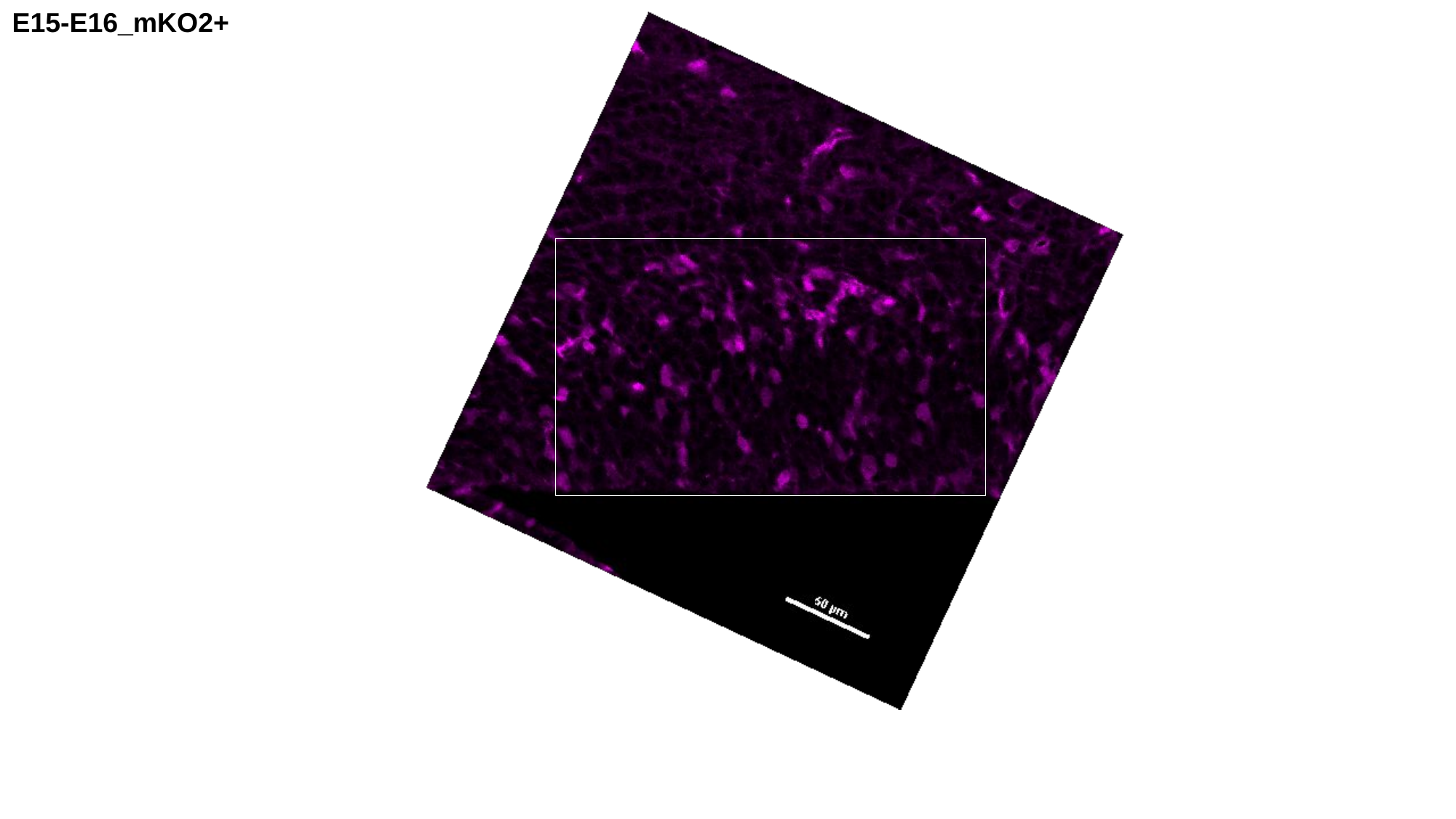

E15-E16_mKO2+

## Slide 8
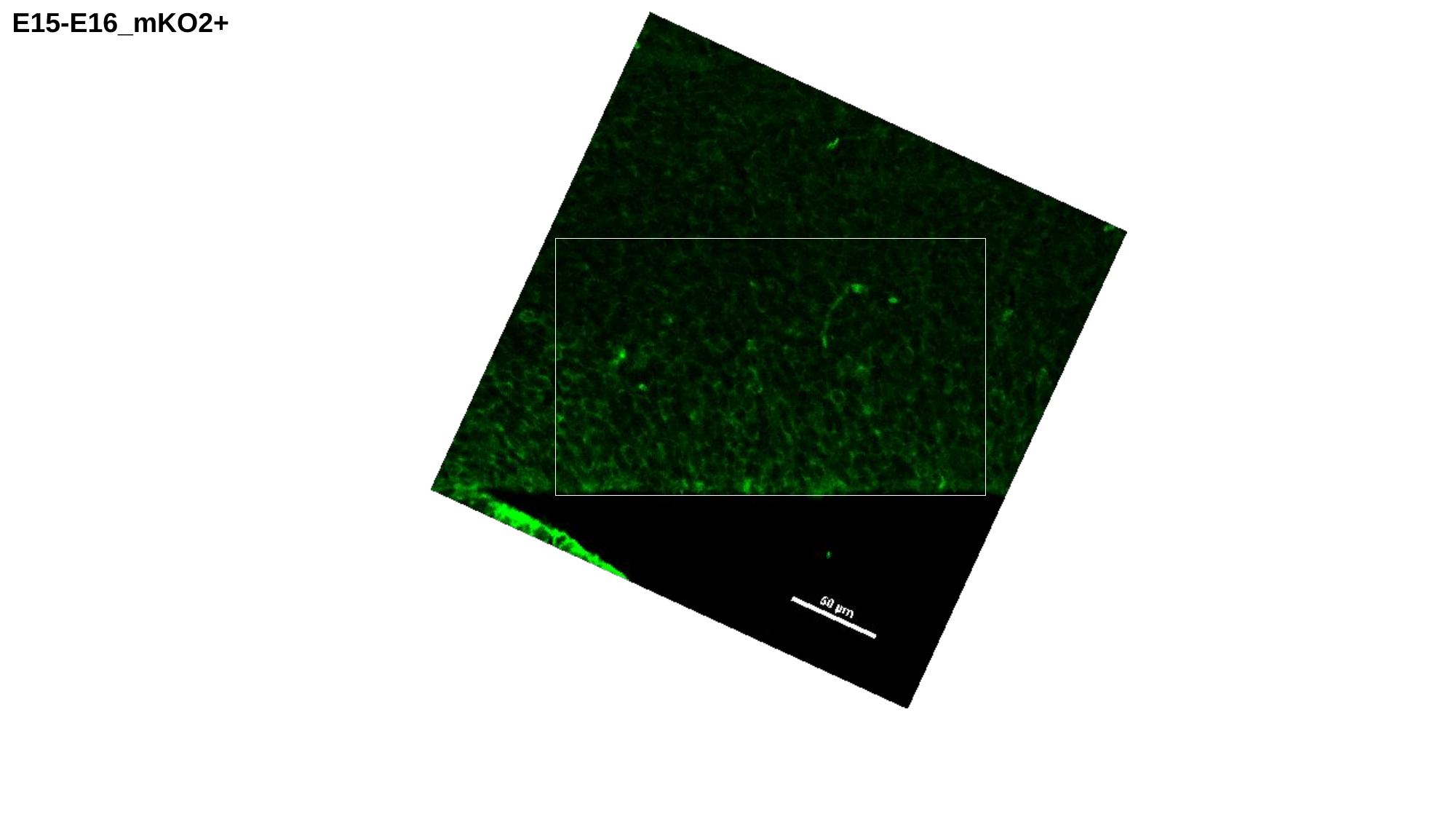

E15-E16_mKO2+

## Slide 9
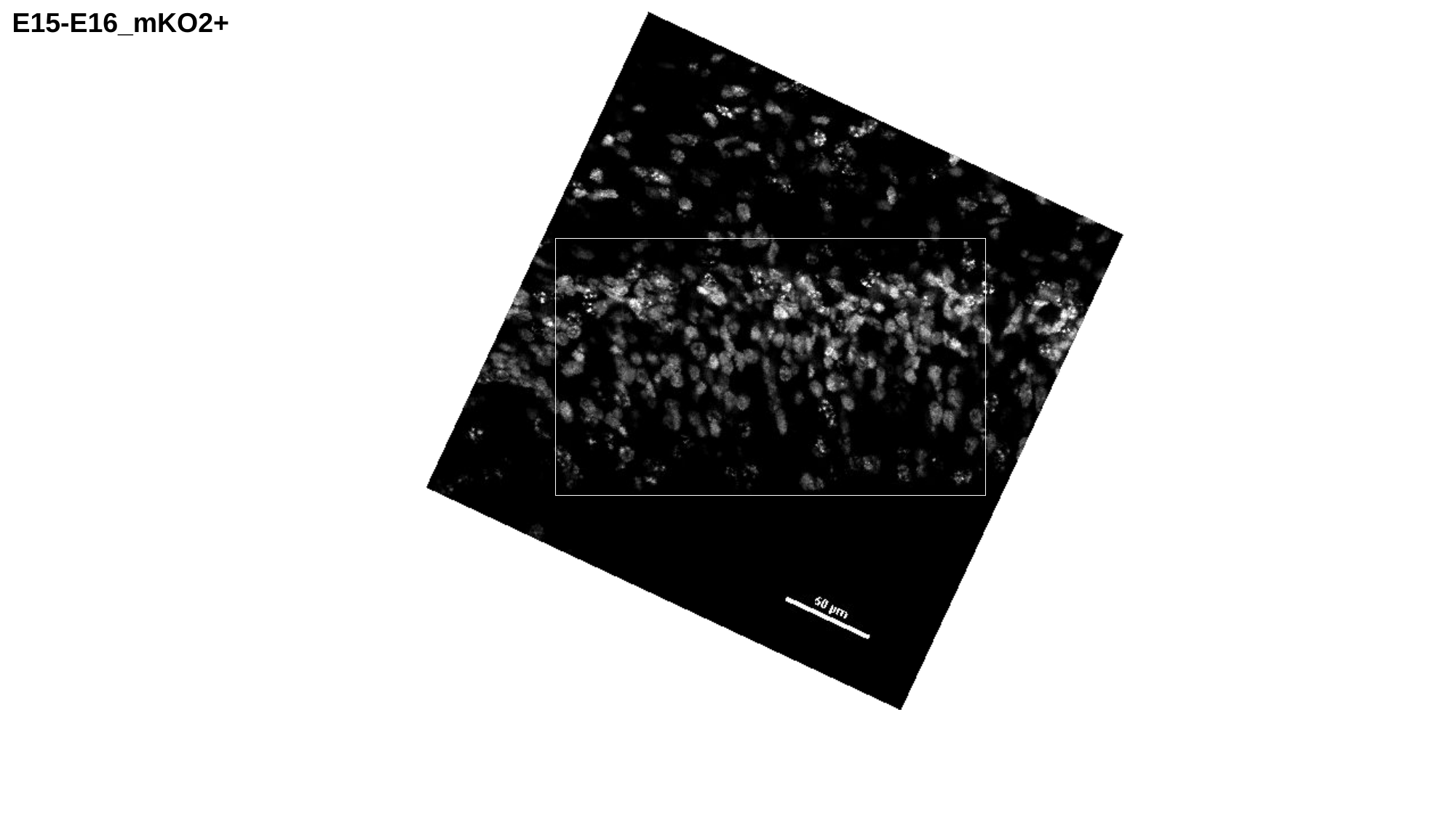

E15-E16_mKO2+

## Slide 10
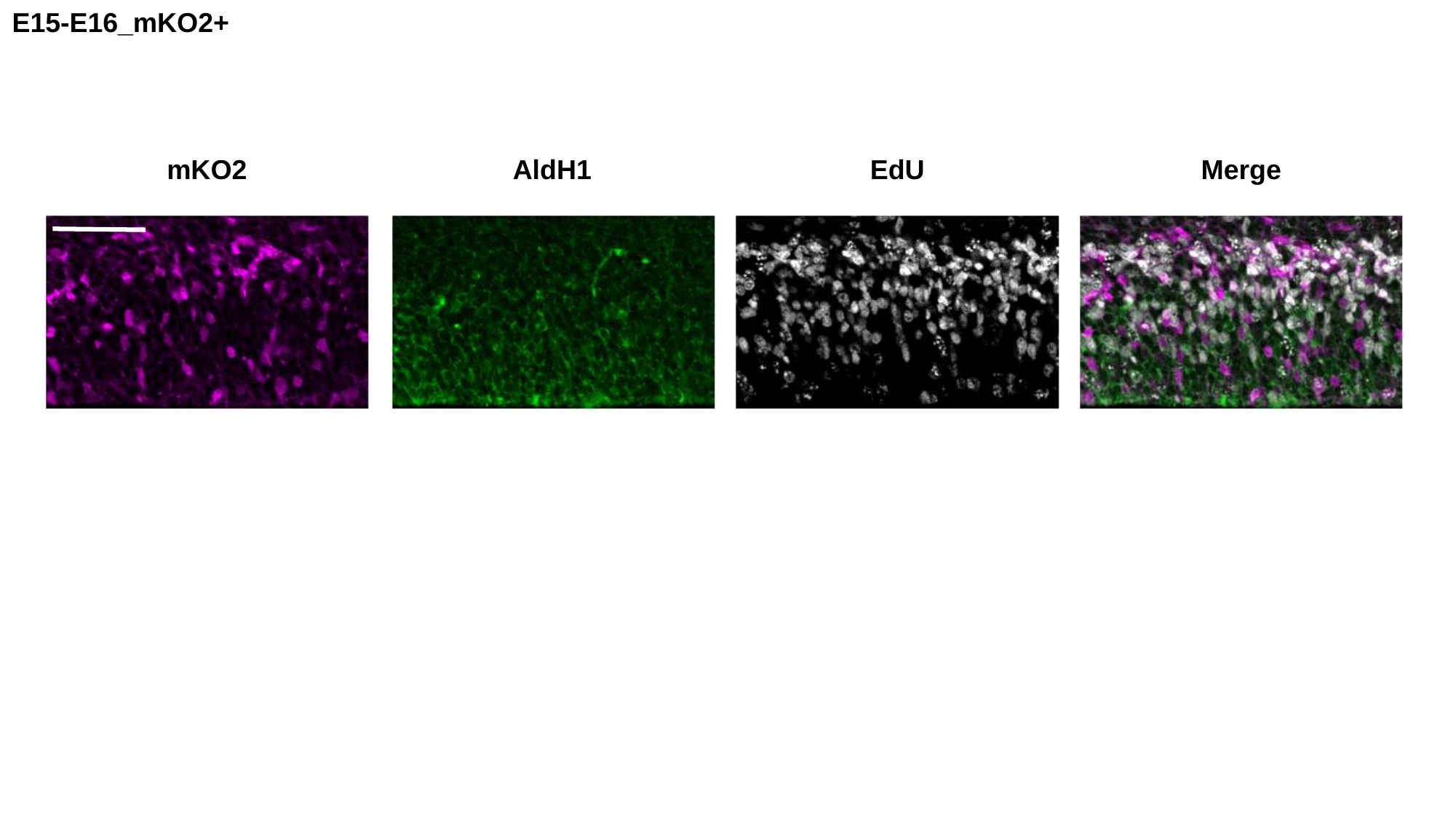

E15-E16_mKO2+
Merge
mKO2
AldH1
EdU

## Slide 11
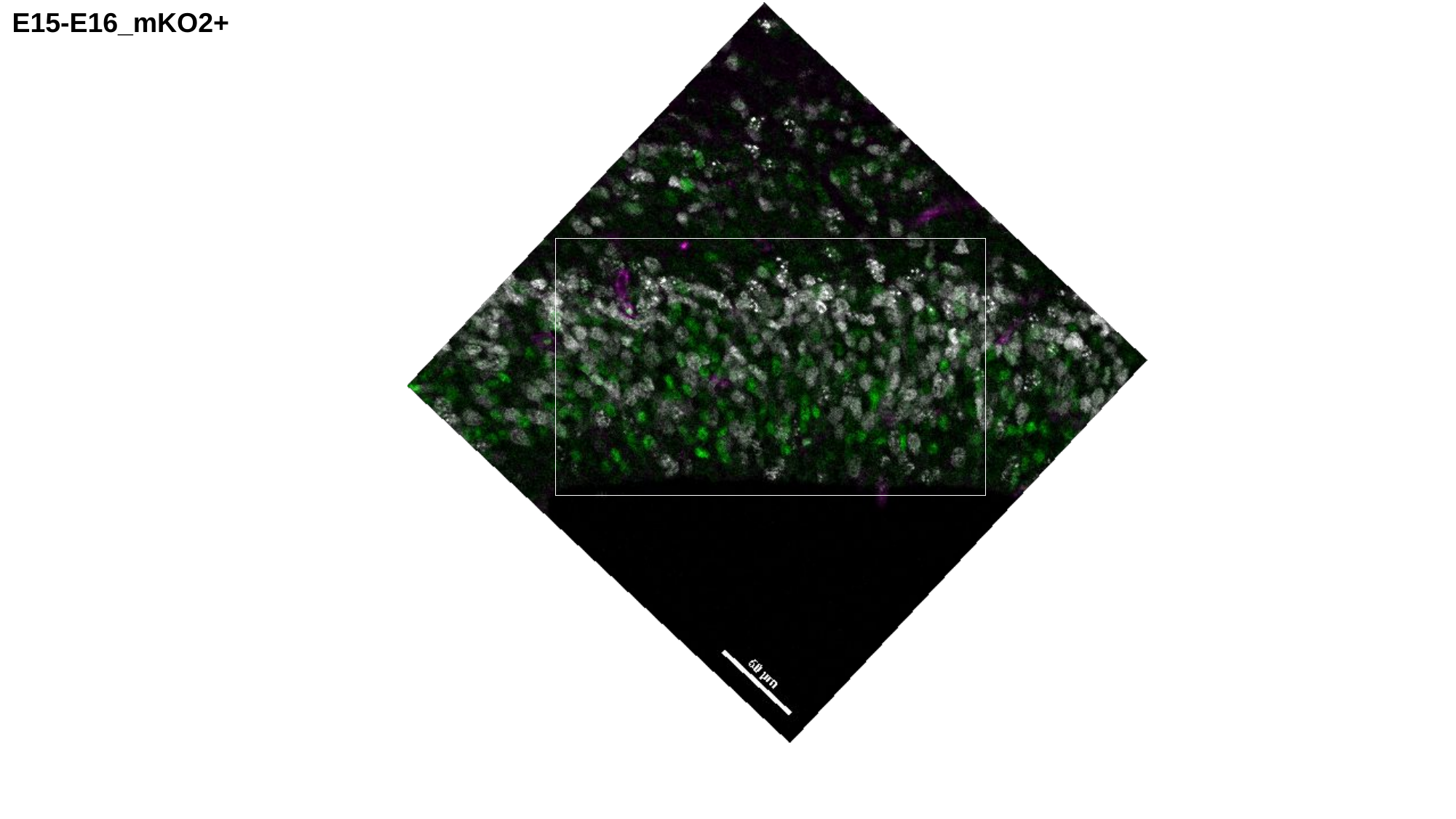

E15-E16_mKO2+

## Slide 12
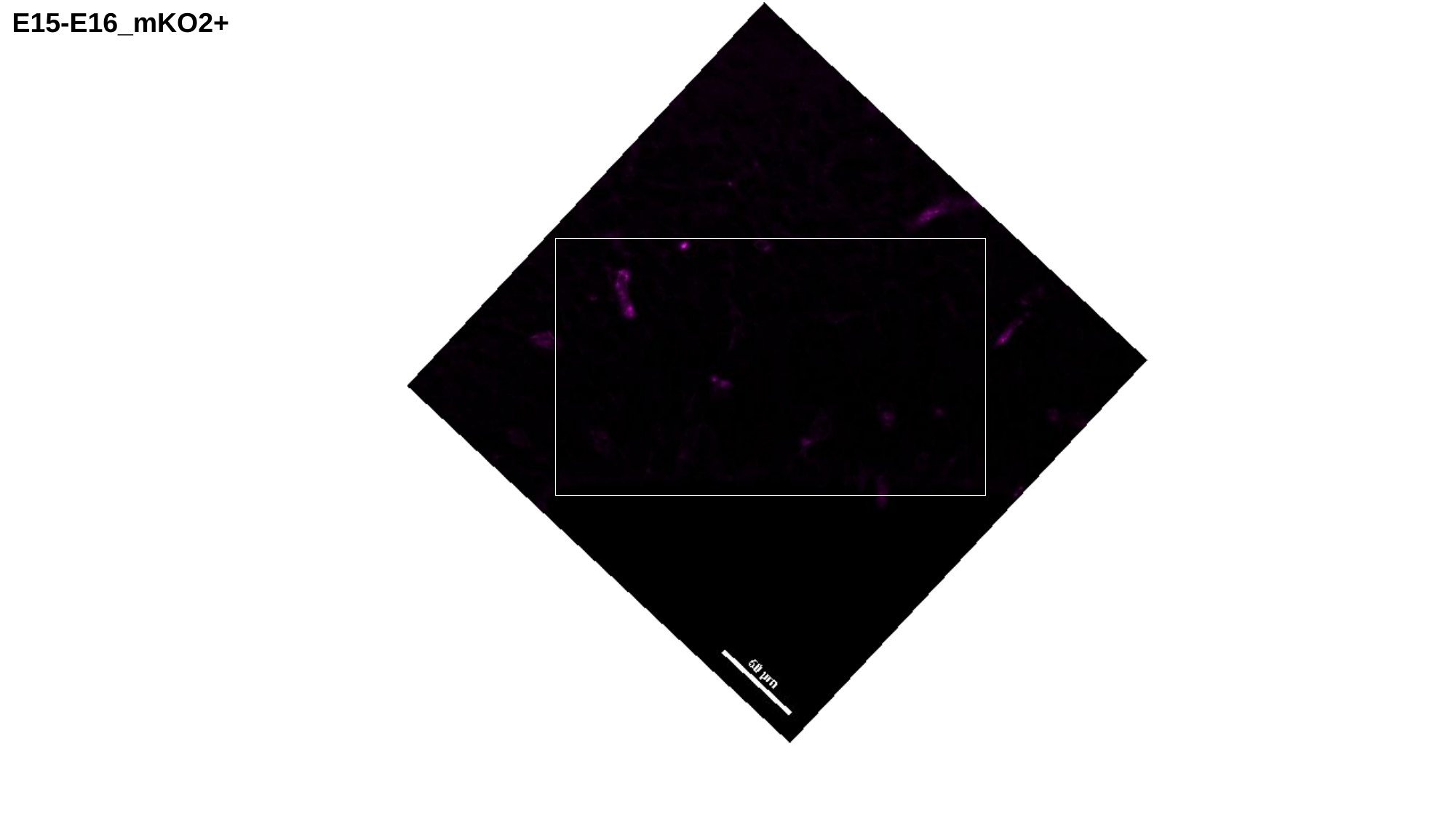

E15-E16_mKO2+

## Slide 13
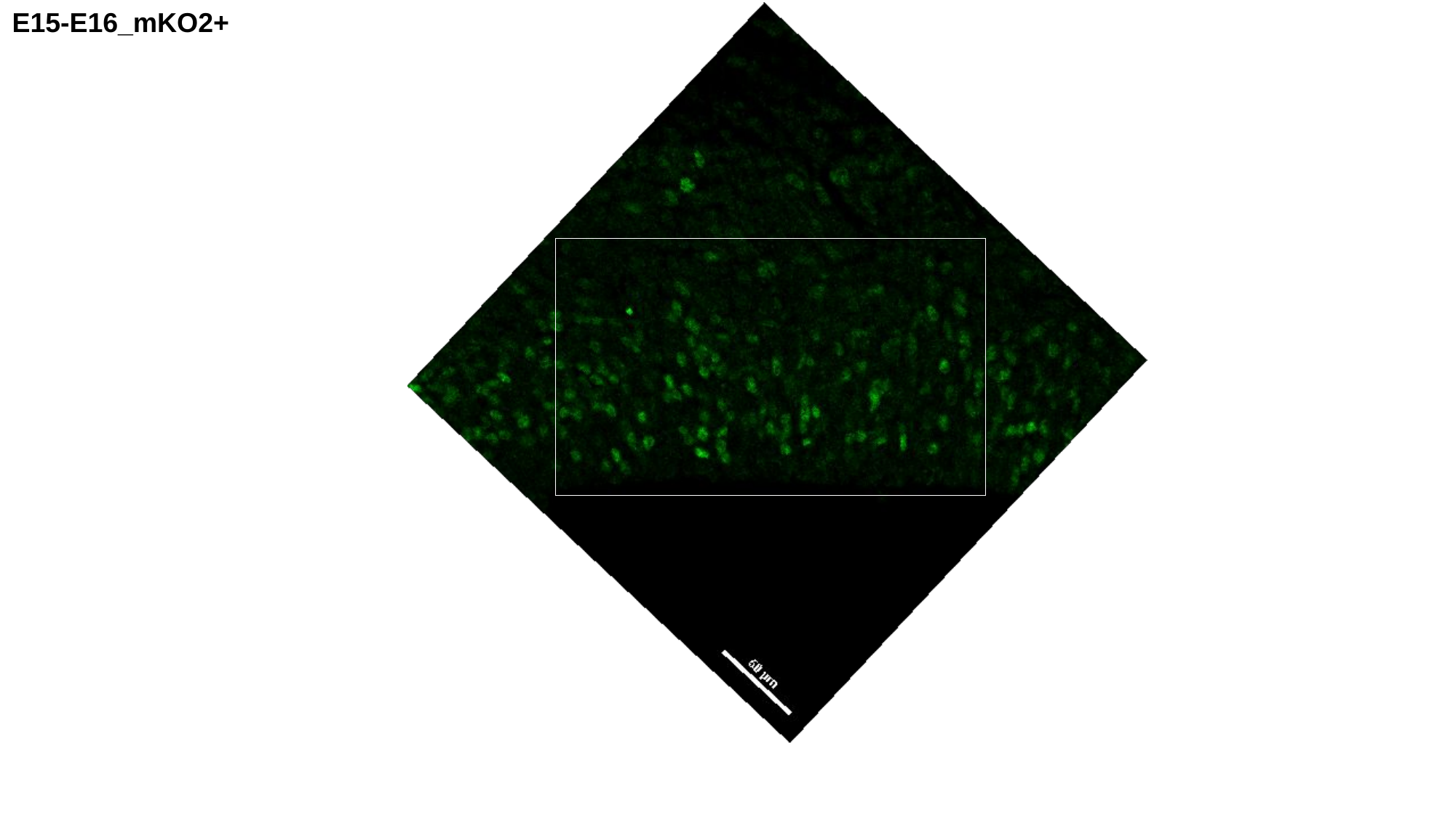

E15-E16_mKO2+

## Slide 14
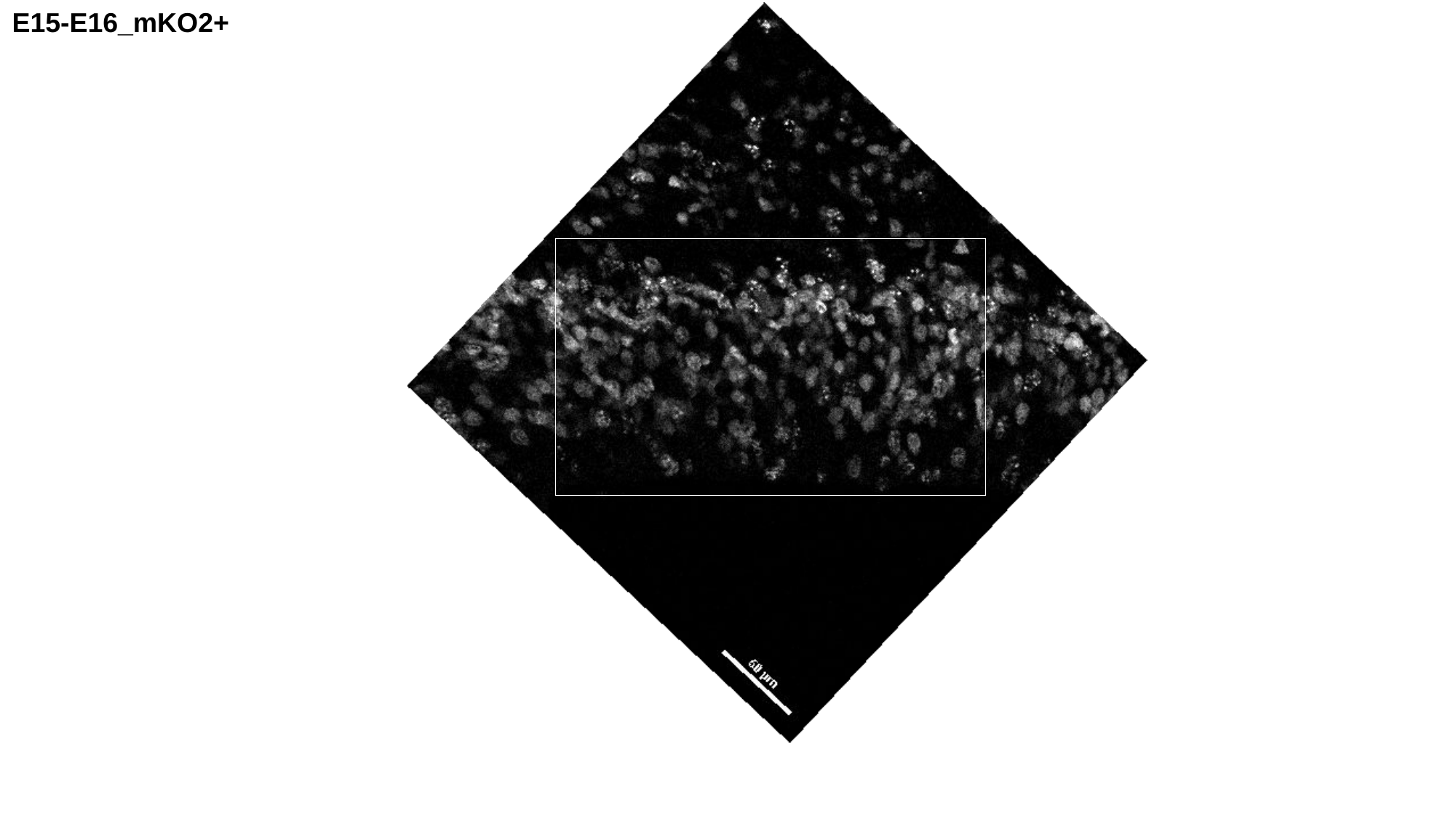

E15-E16_mKO2+

## Slide 15
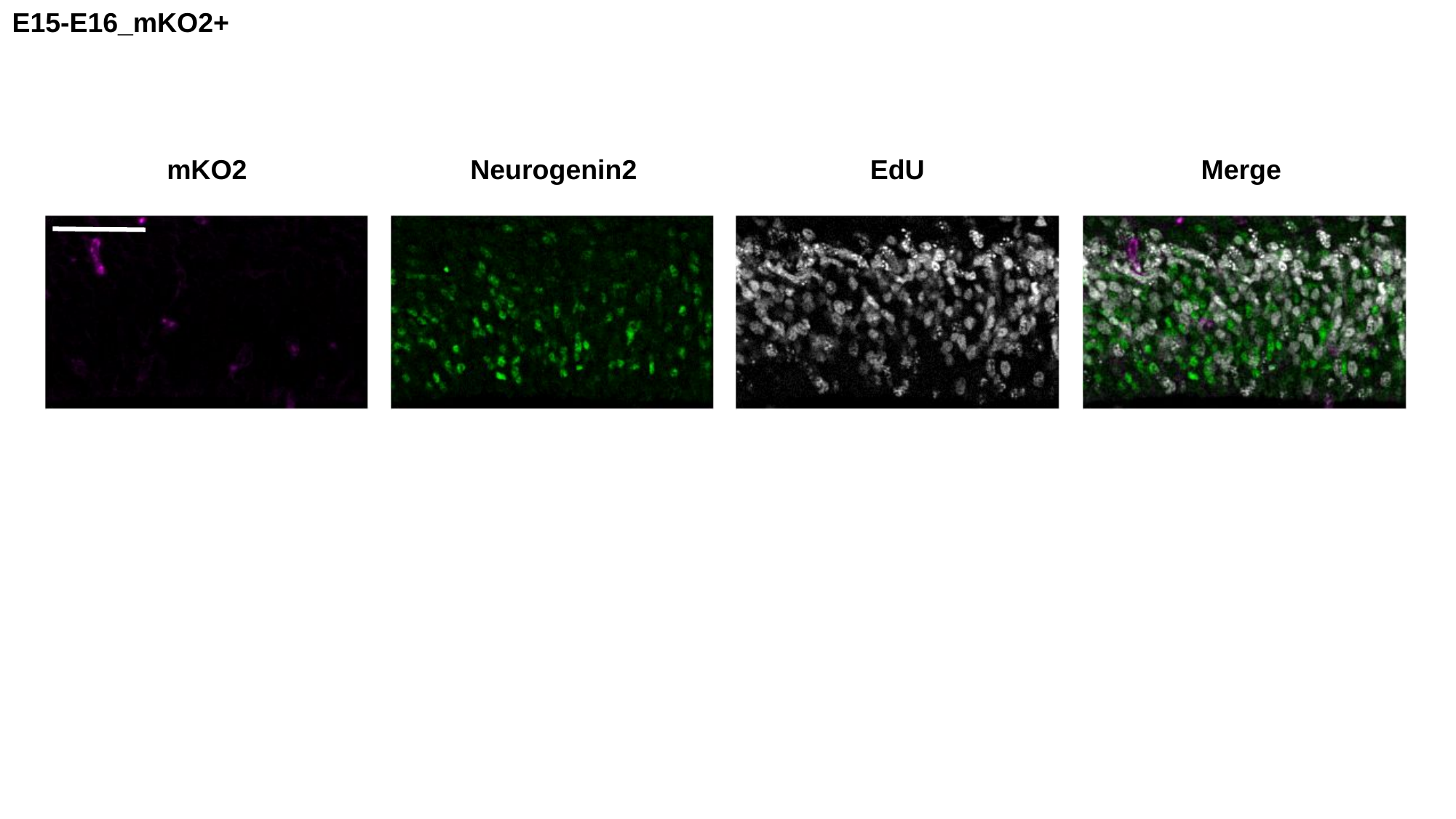

E15-E16_mKO2+
Neurogenin2
Merge
mKO2
EdU

## Slide 16
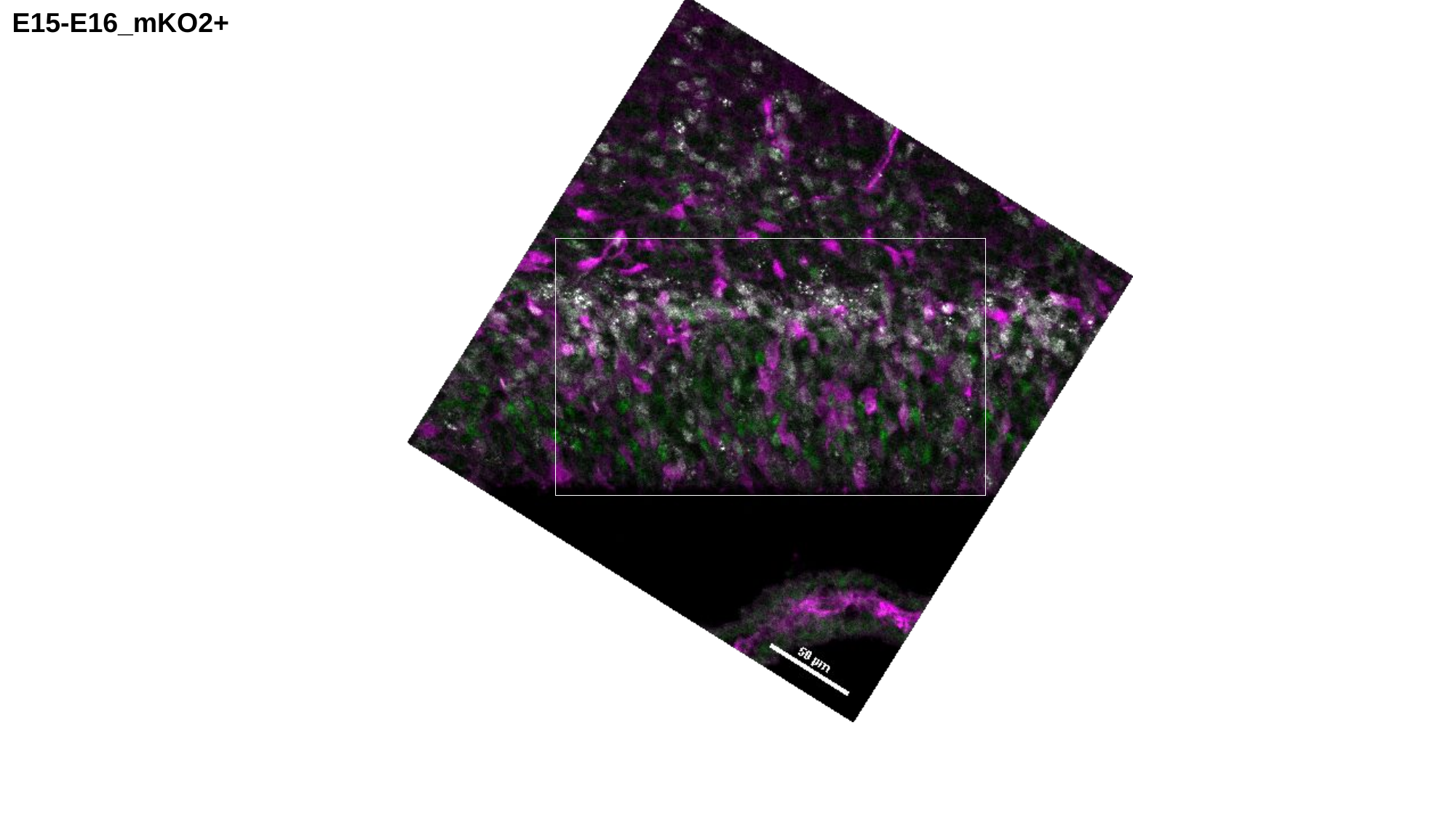

E15-E16_mKO2+

## Slide 17
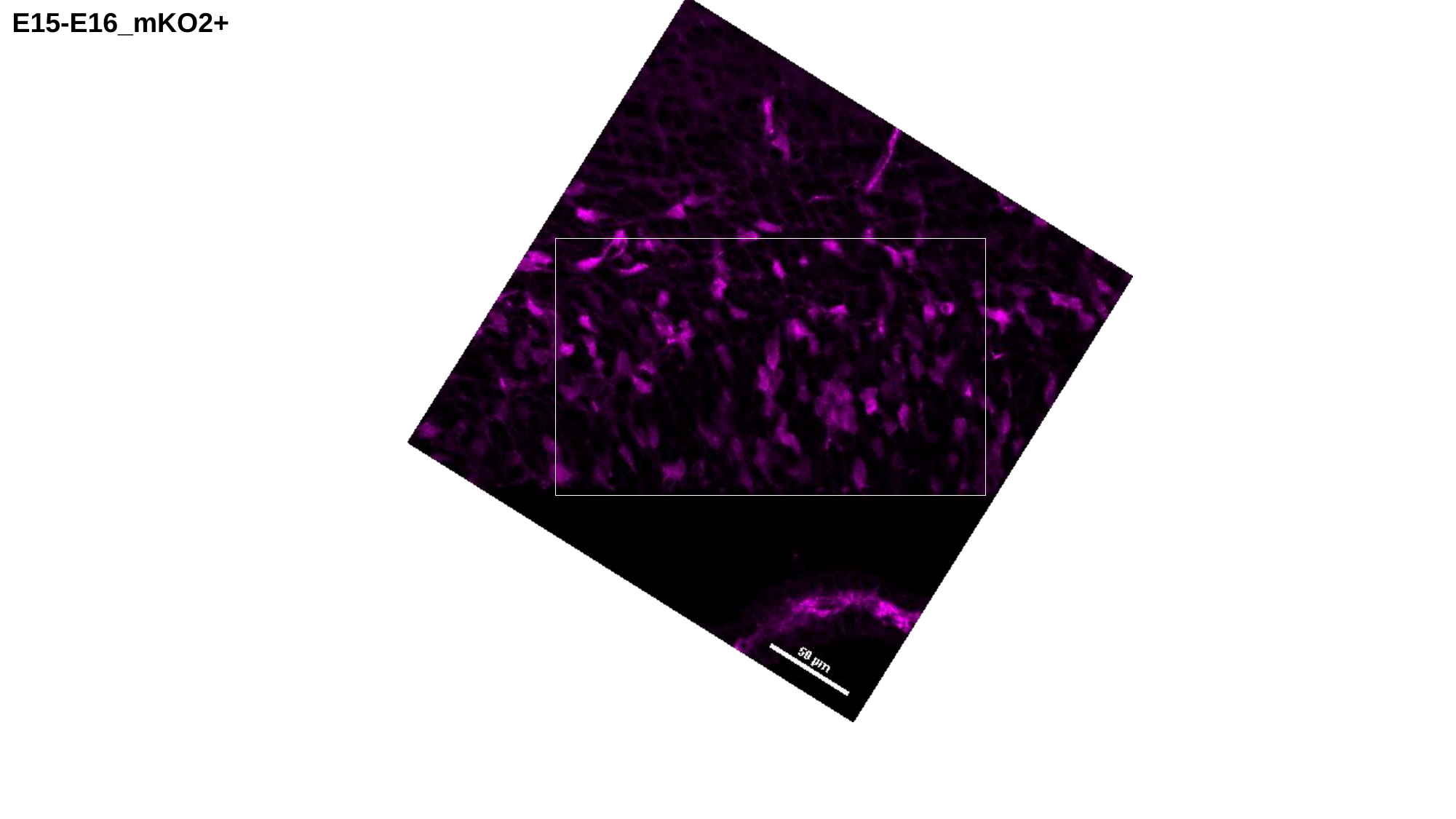

E15-E16_mKO2+

## Slide 18
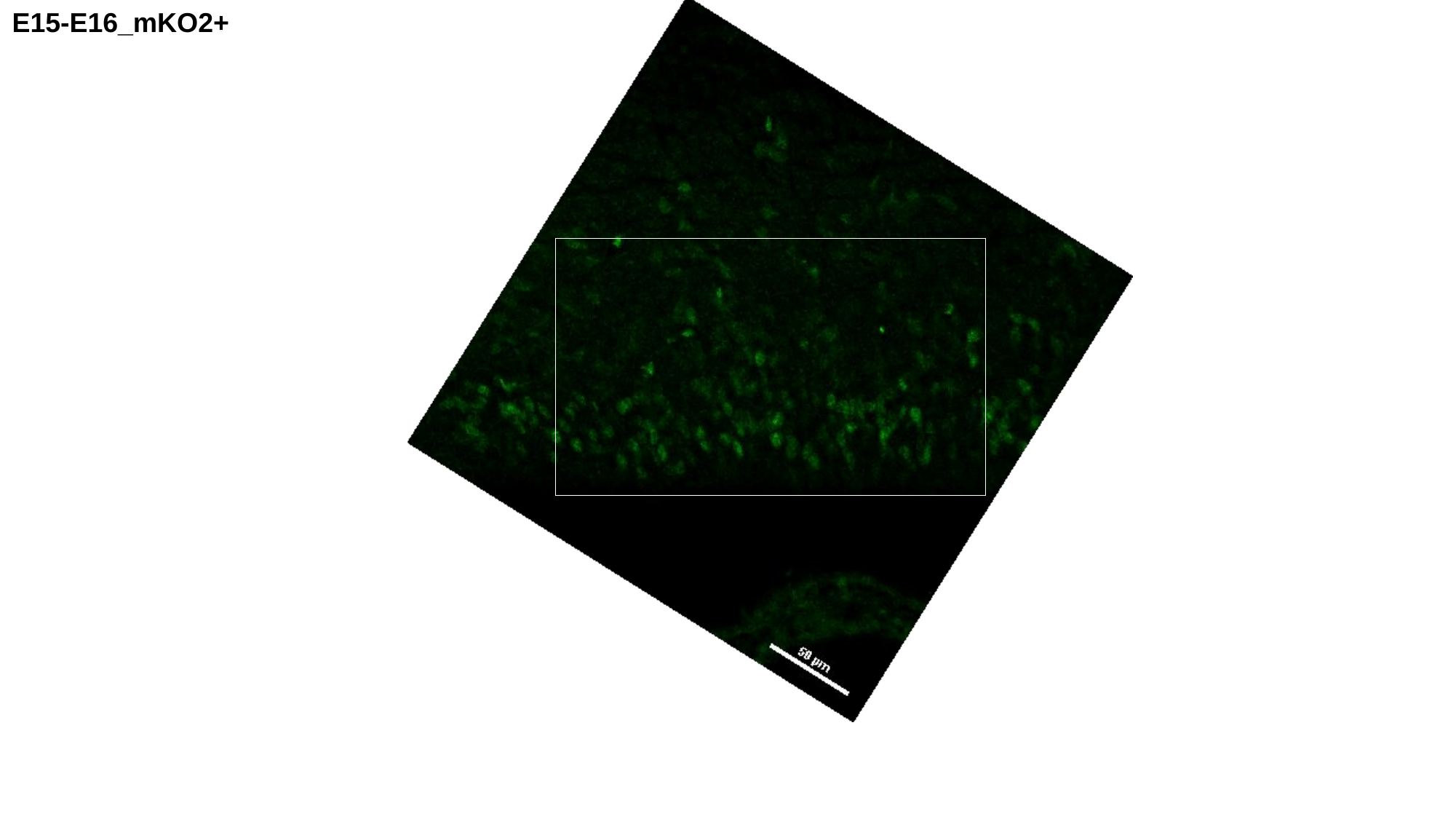

E15-E16_mKO2+

## Slide 19
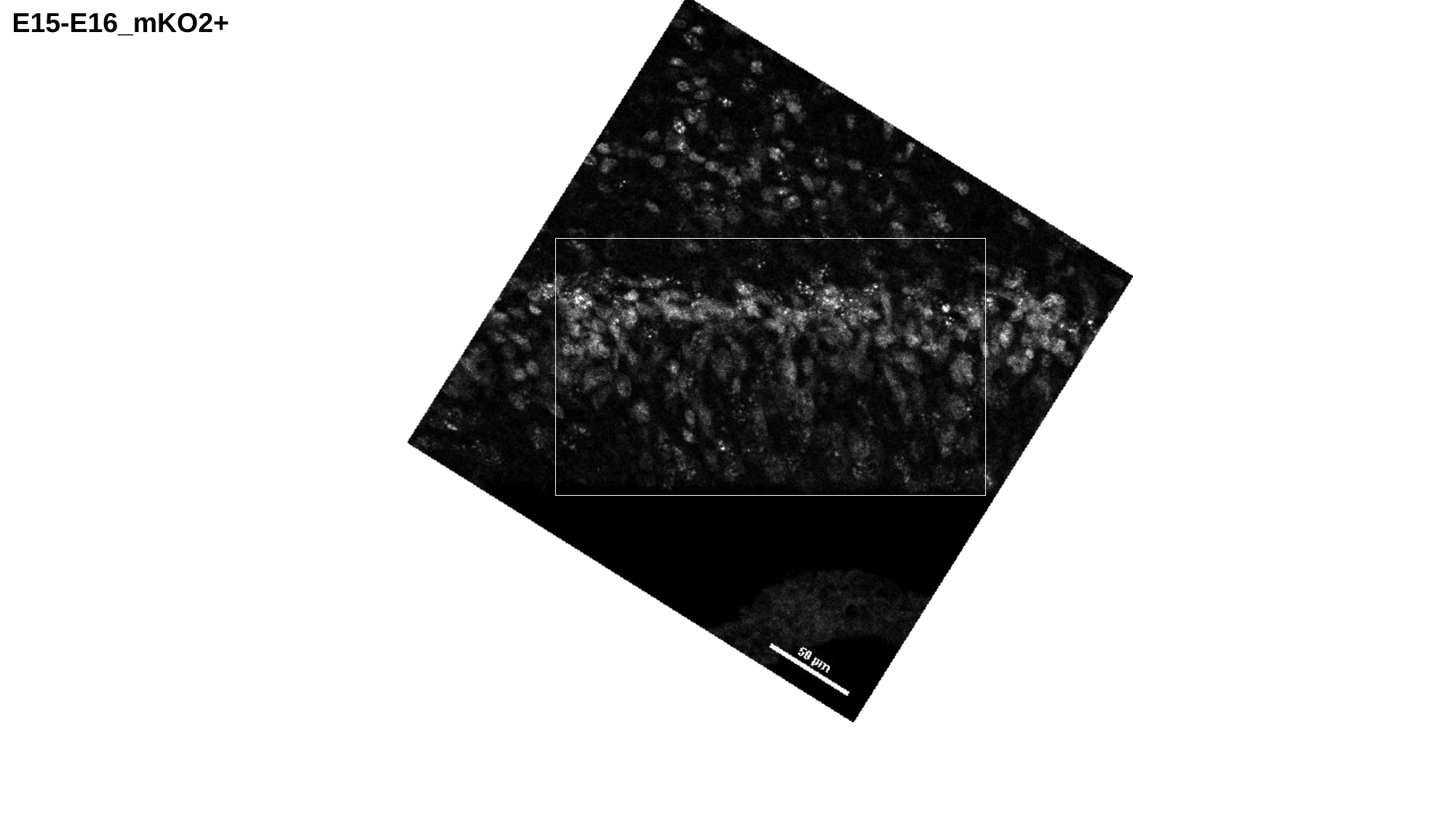

E15-E16_mKO2+

## Slide 20
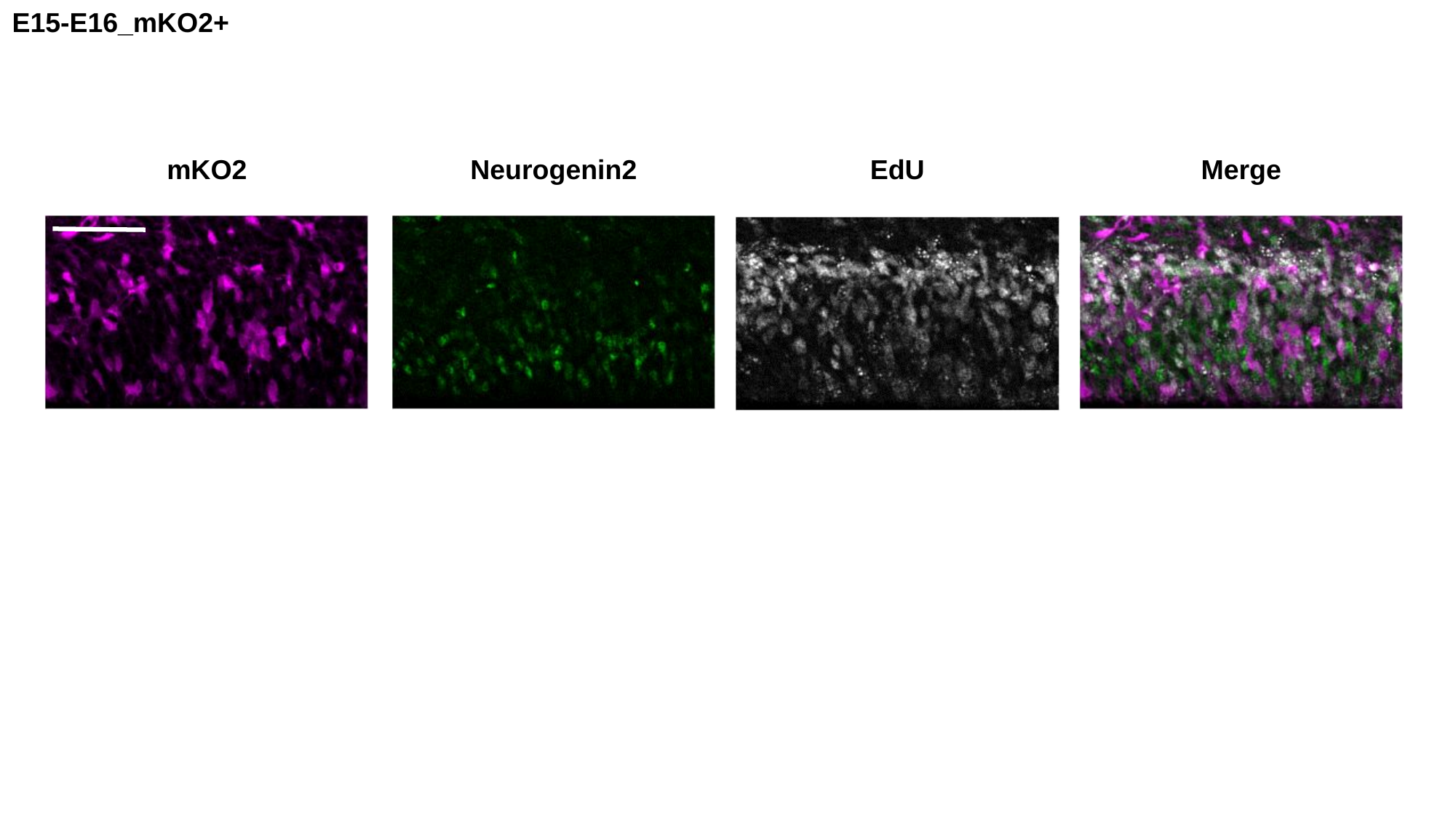

E15-E16_mKO2+
Neurogenin2
Merge
mKO2
EdU
