# Supplementary material for: Cyclin-dependent kinase inhibitor p18 regulates lineage transitions of excitatory neurons, astrocytes, and interneurons in the mouse cortex
Source: EMBO J. 2024 Dec 12;44(2):382–412. doi: 10.1038/s44318-024-00325-9 (PMC11730326; doi:10.1038/s44318-024-00325-9)
Supplement: Supplementary file 7 — Source data Fig. 5 [file 44318_2024_325_MOESM7_ESM.zip › 5D.pptx]

## Slide 1
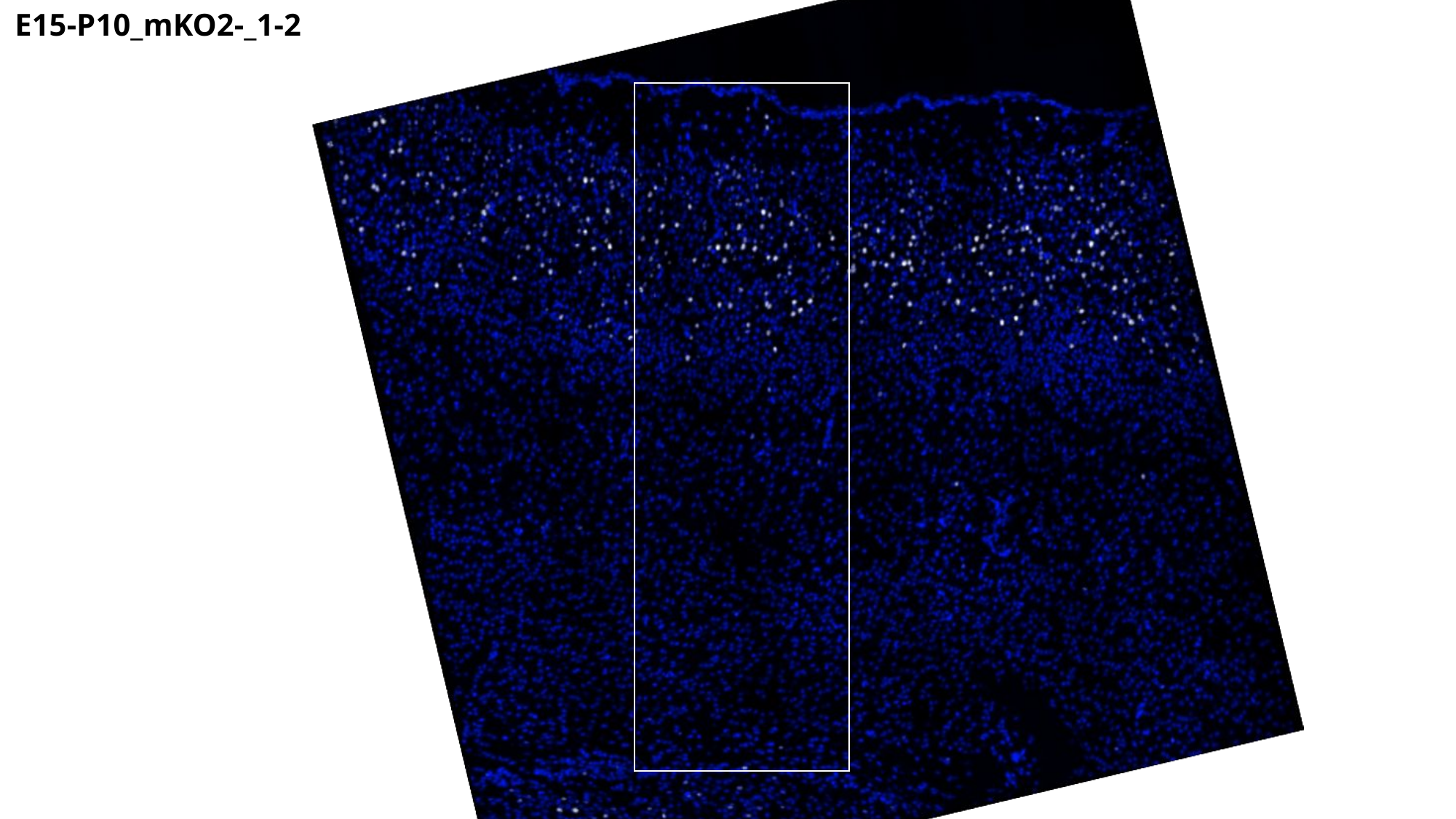

E15-P10_mKO2-_1-2

## Slide 2
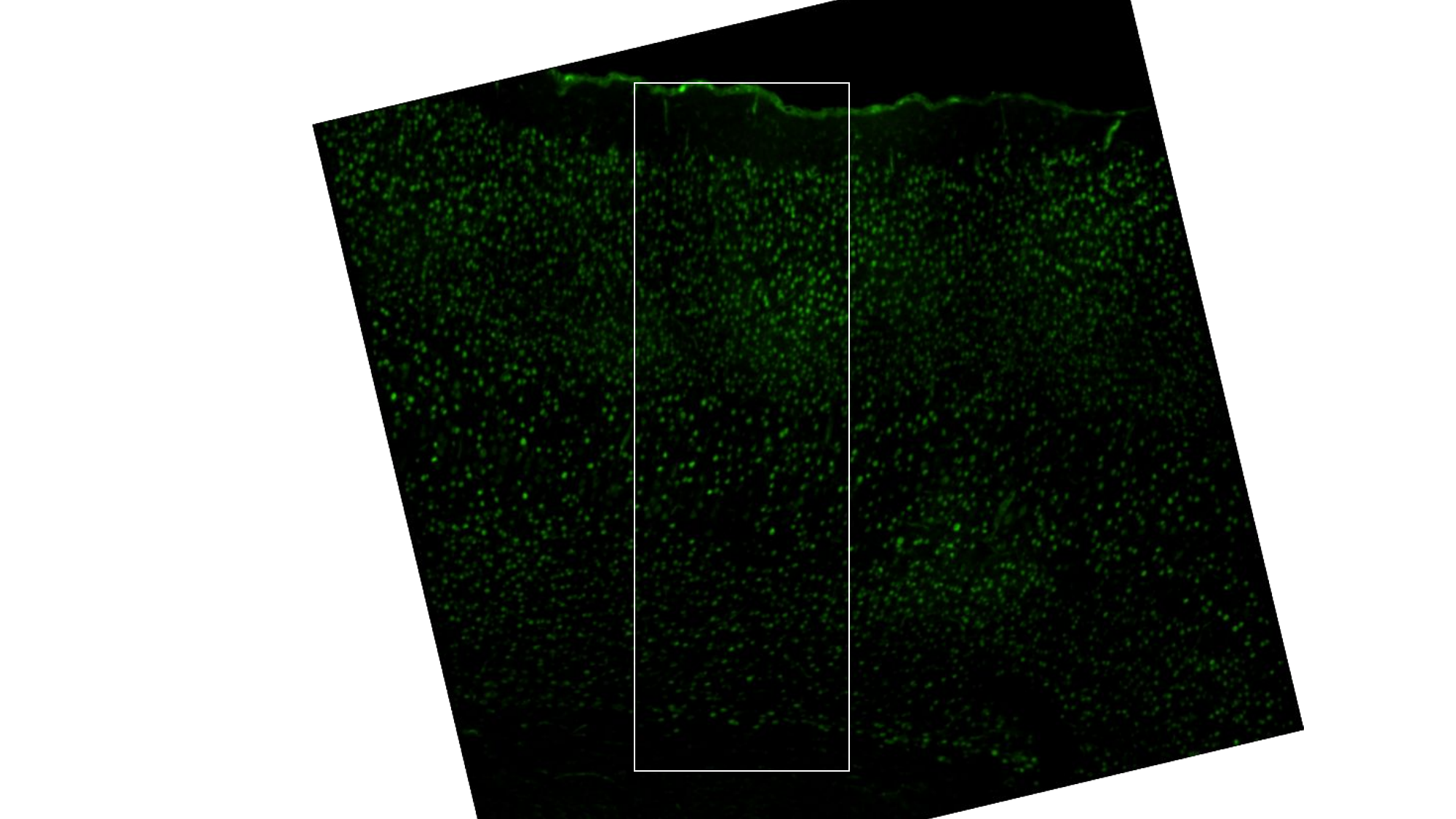

## Slide 3
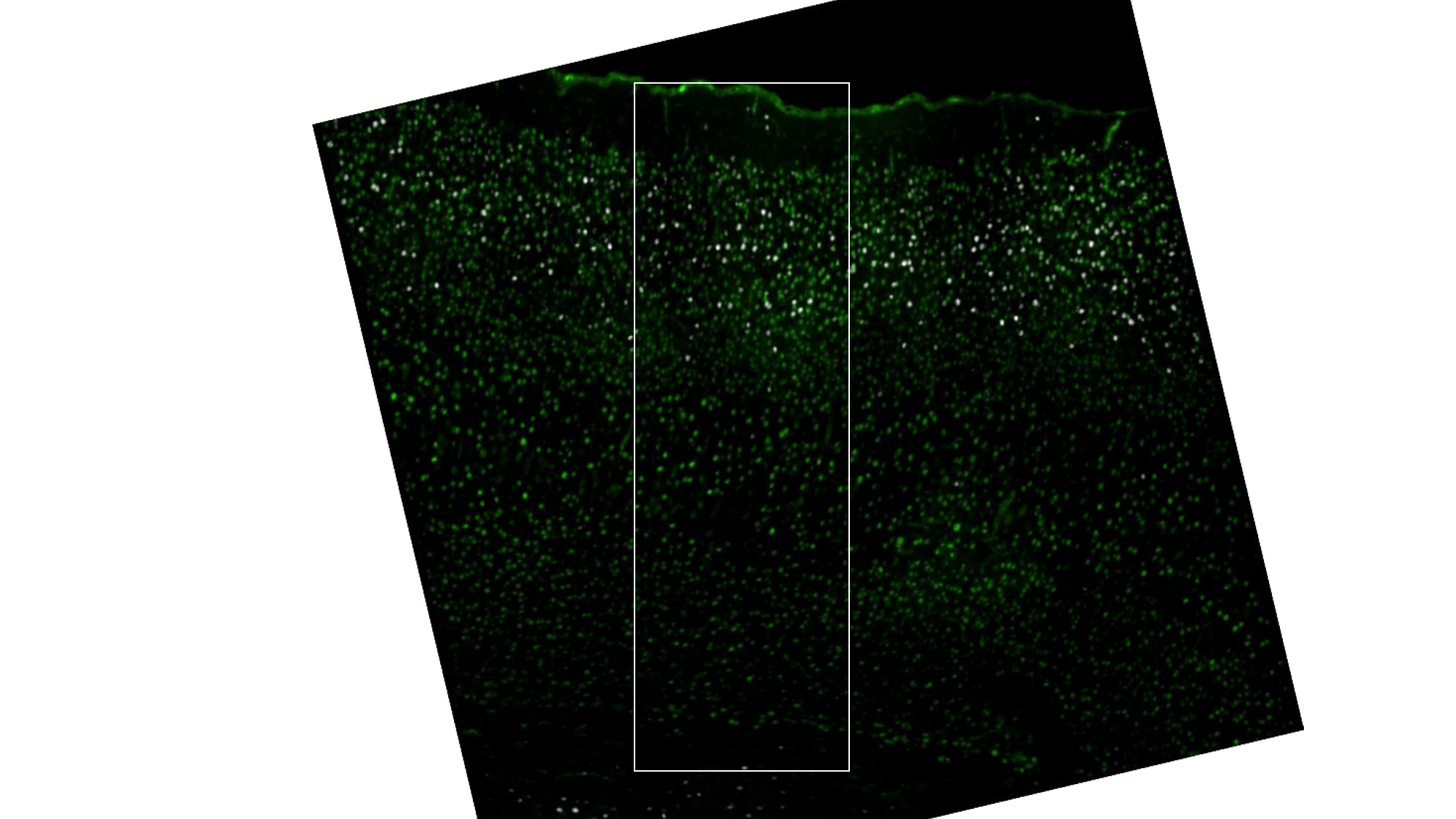

## Slide 4
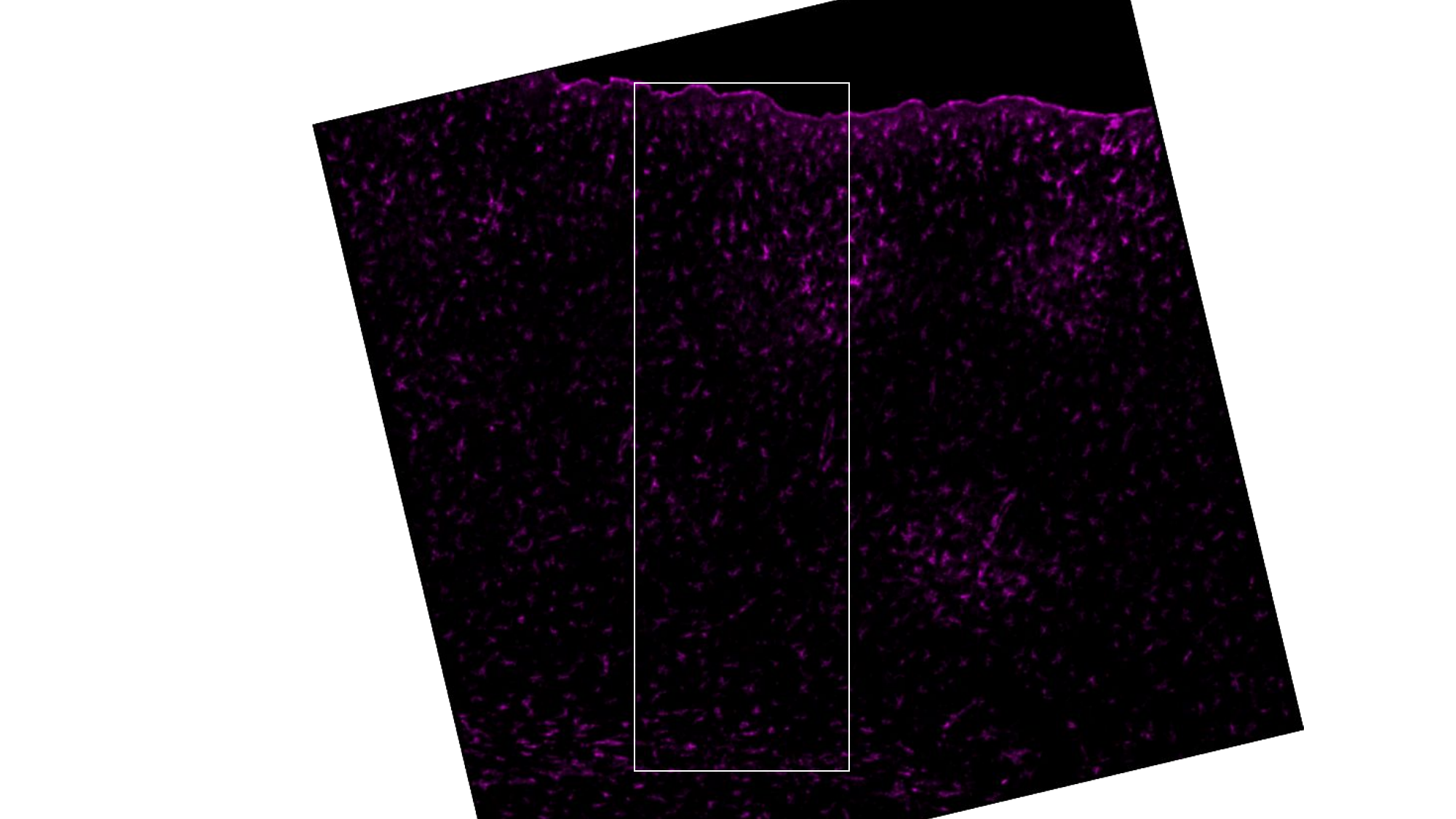

## Slide 5
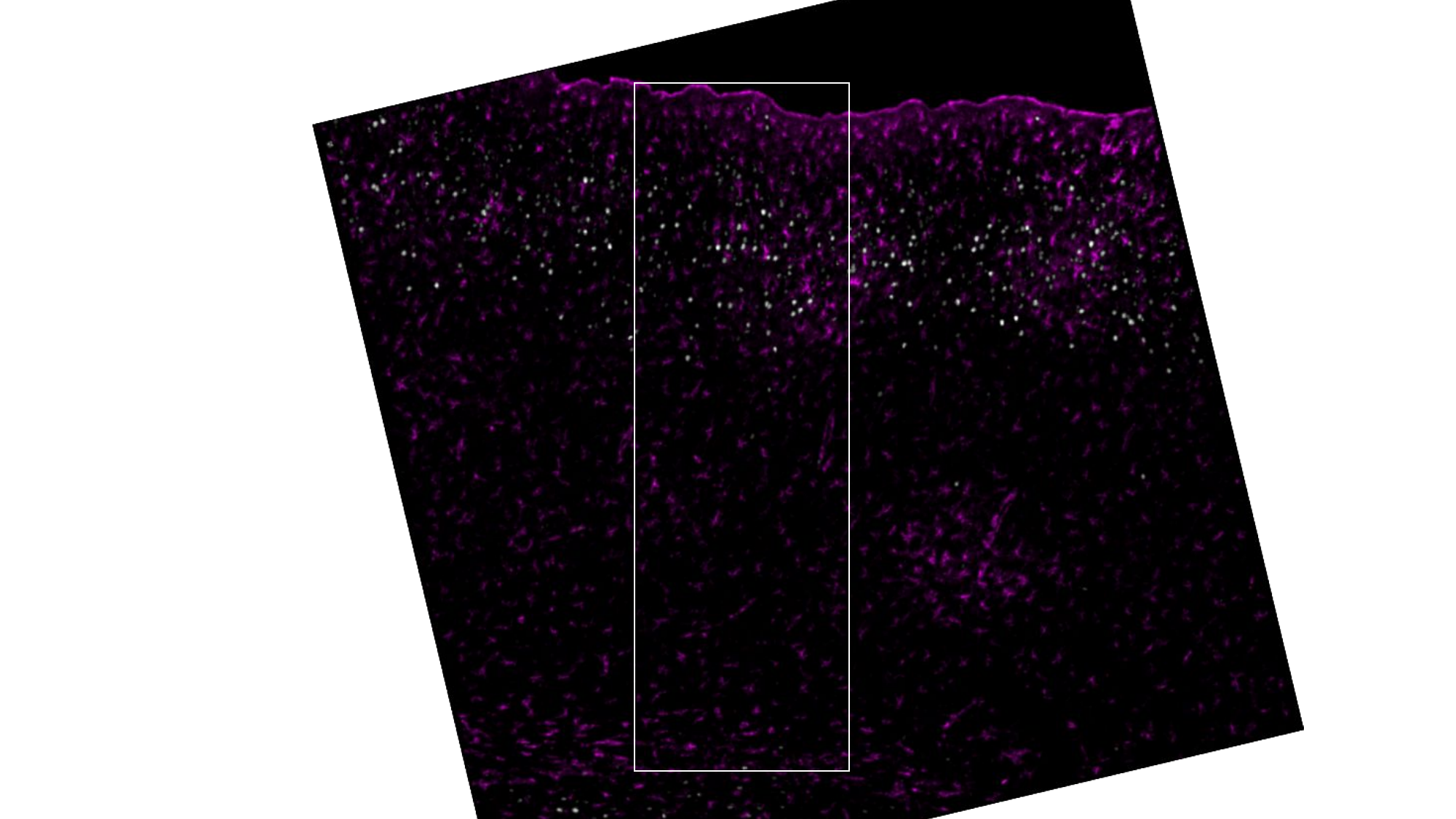

## Slide 6
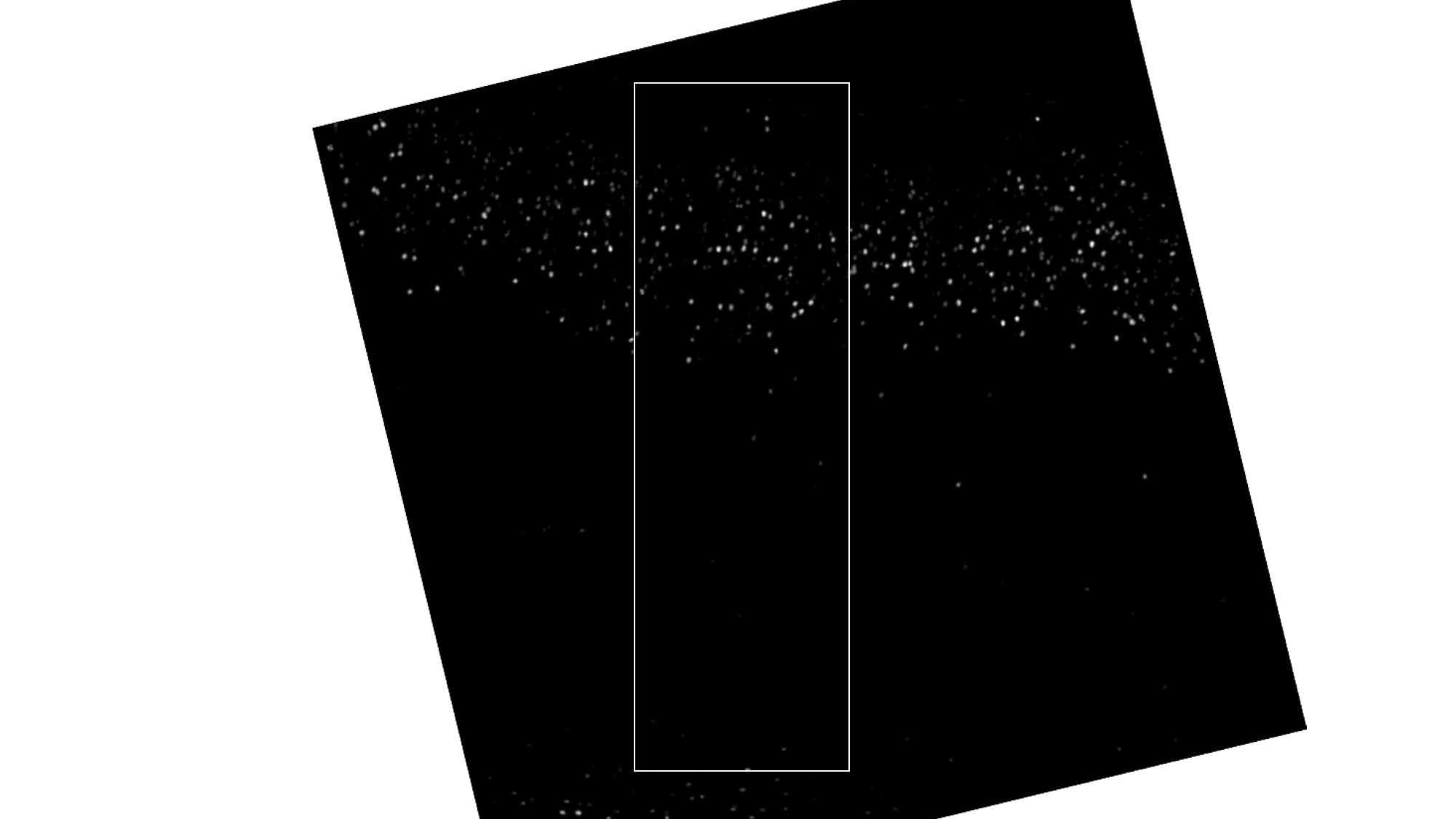

## Slide 7
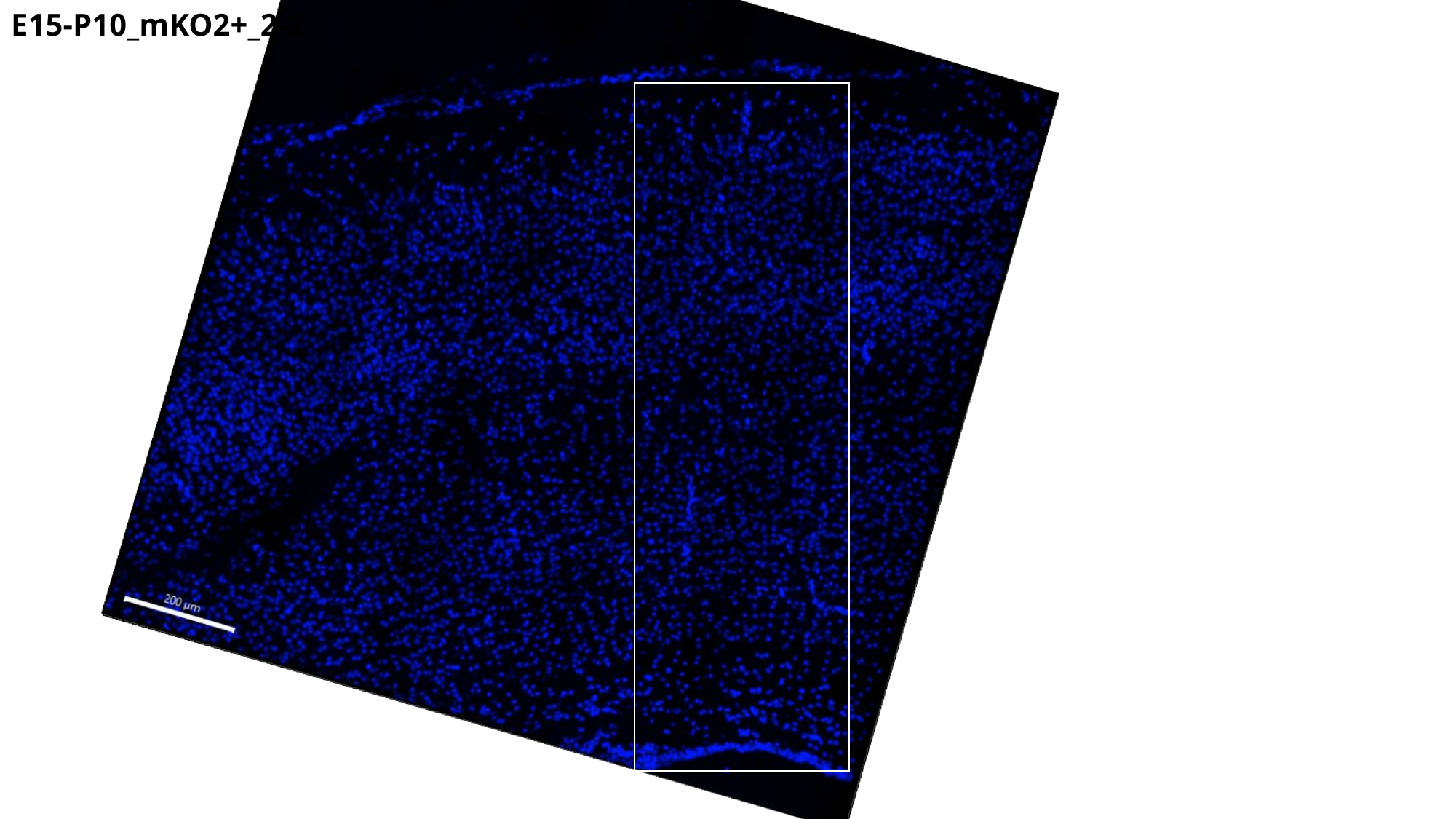

E15-P10_mKO2+_2-2

## Slide 8
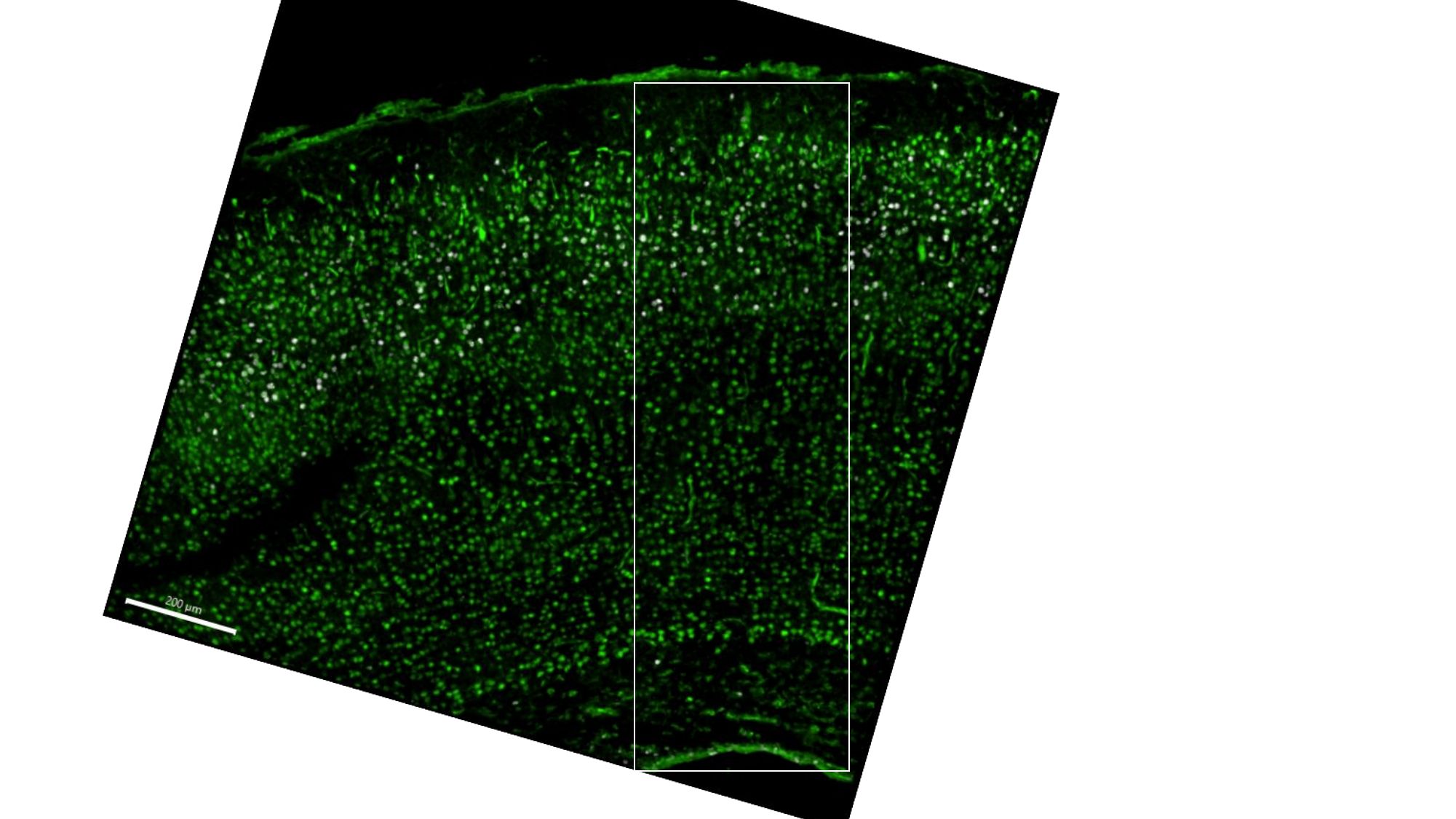

## Slide 9
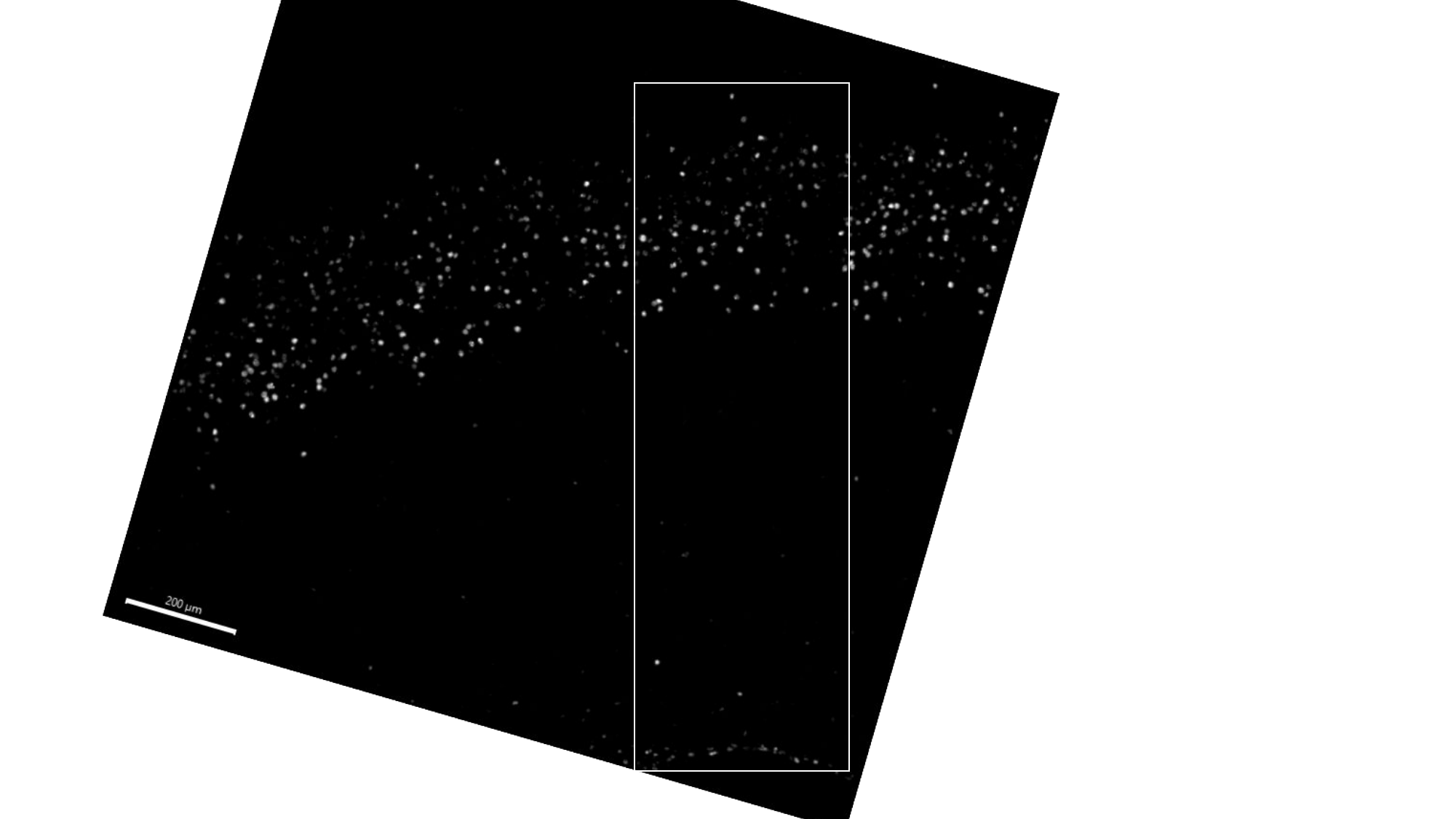

## Slide 10
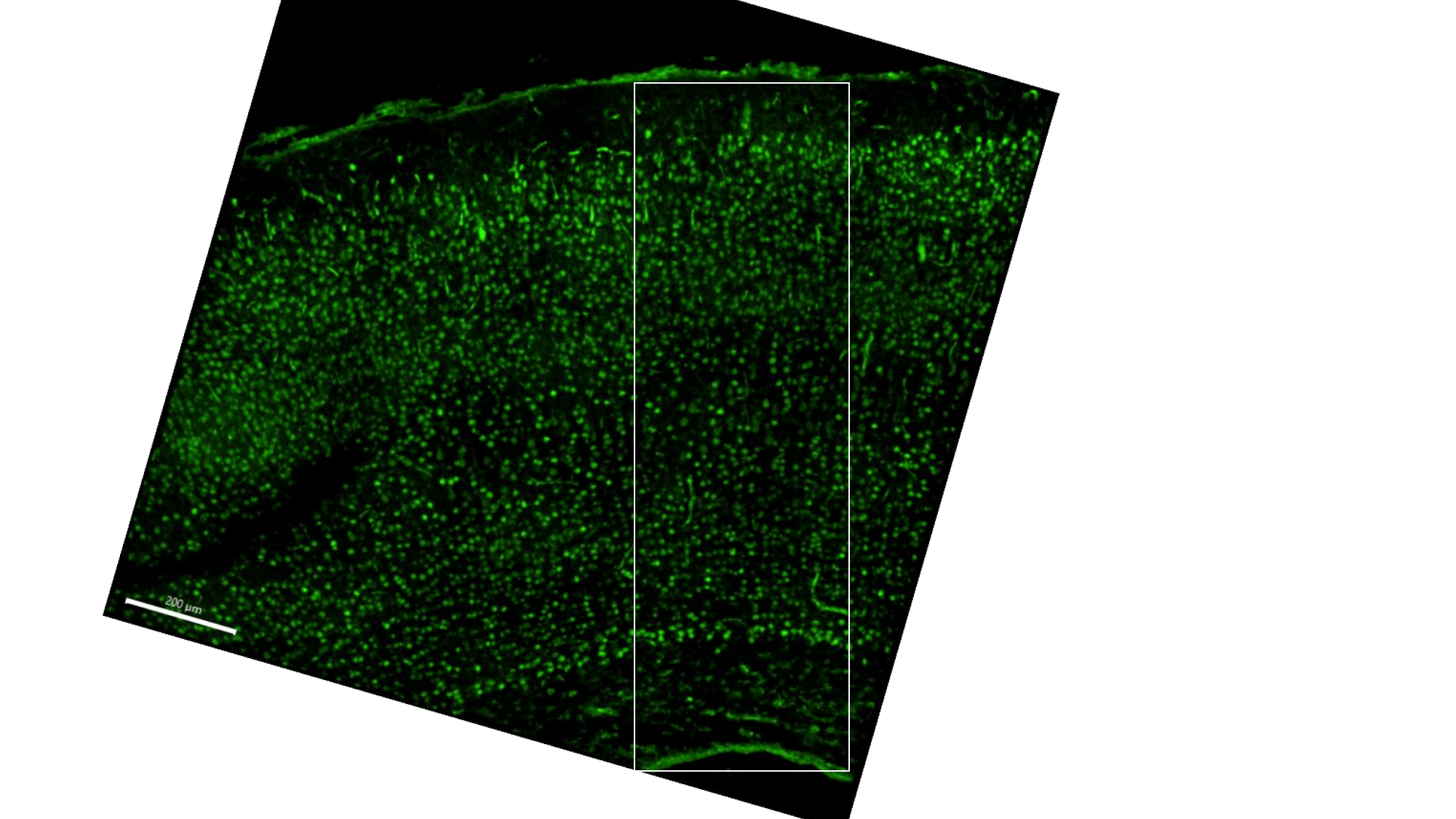

## Slide 11
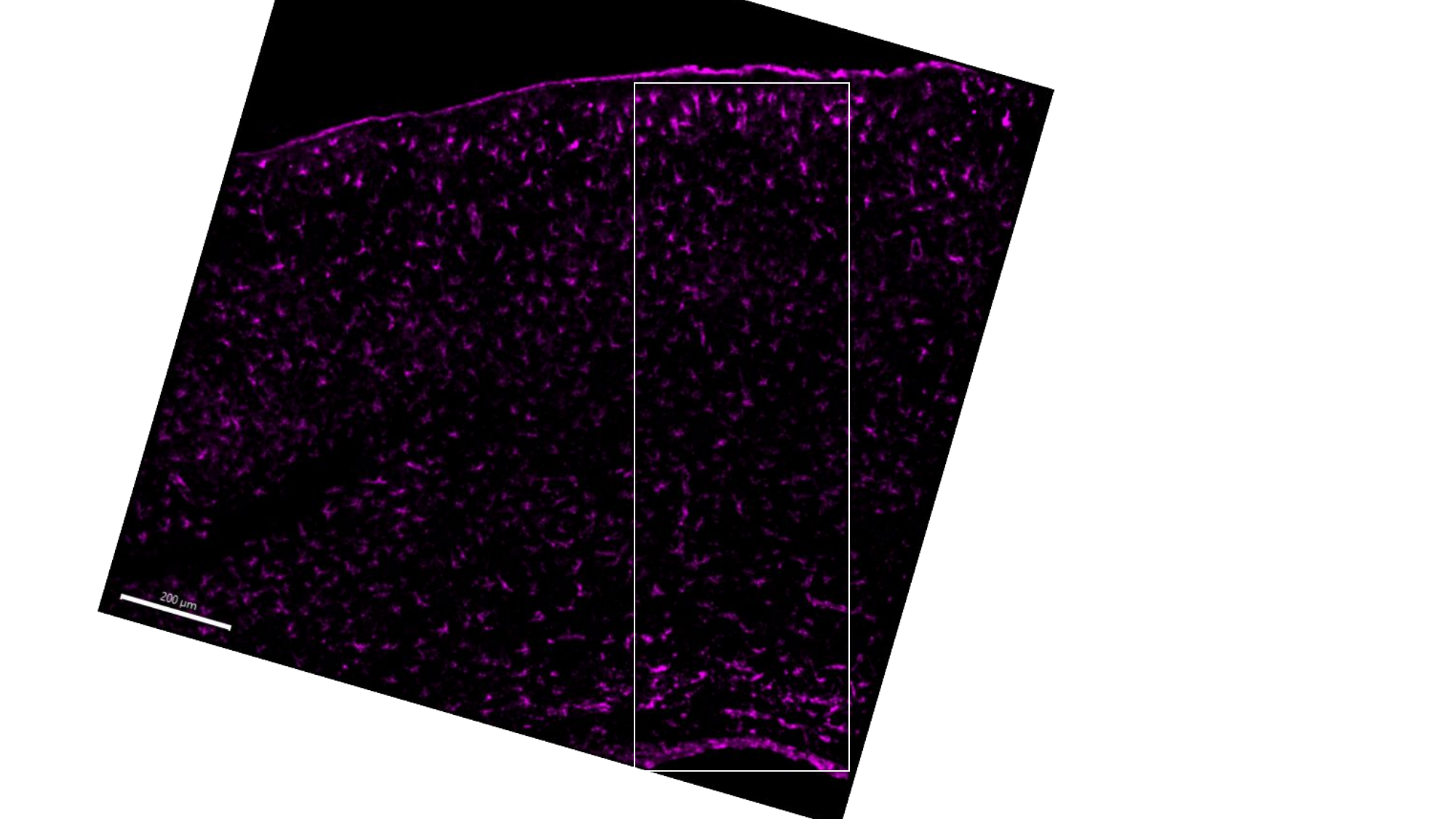

## Slide 12
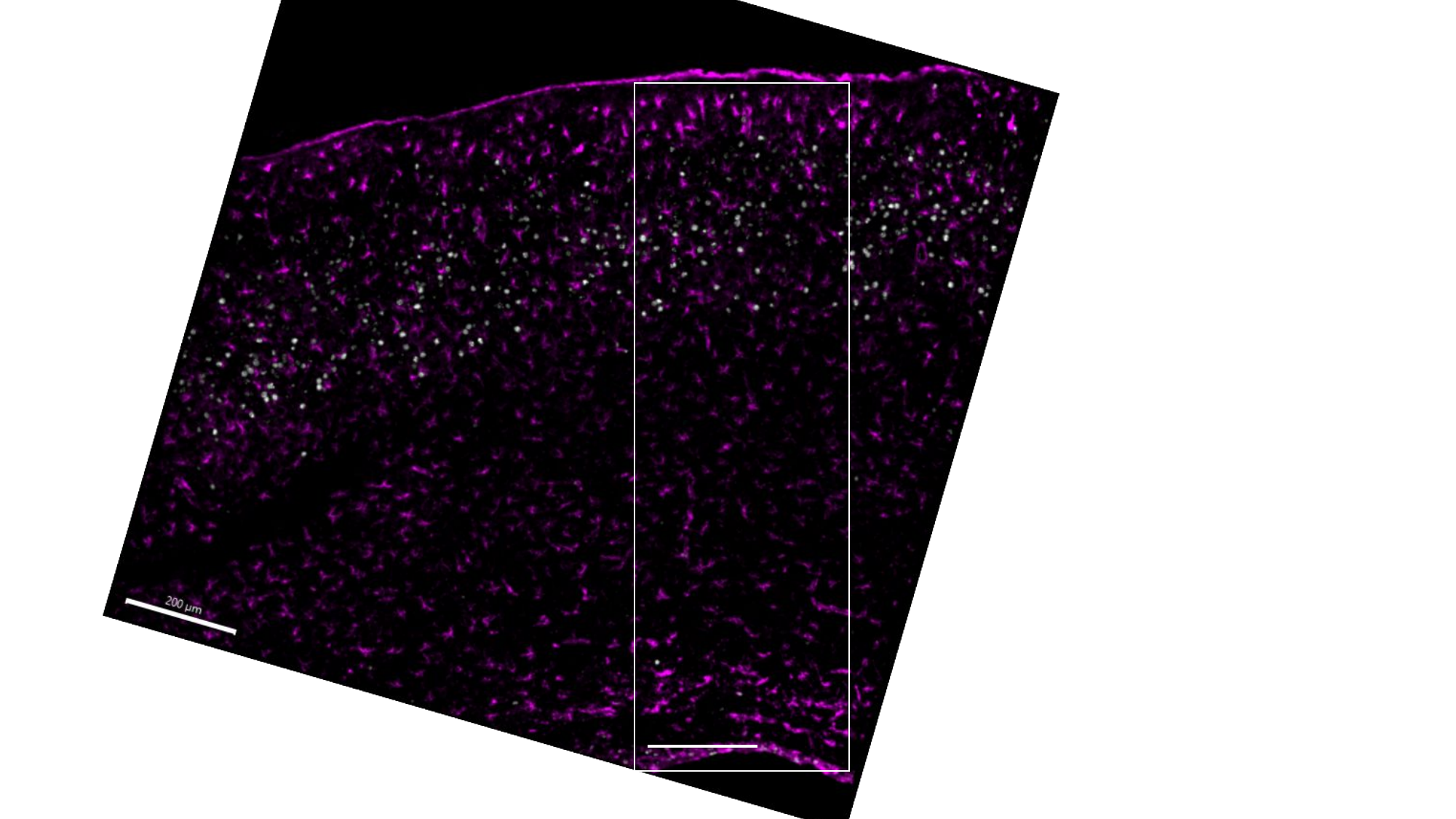

## Slide 13
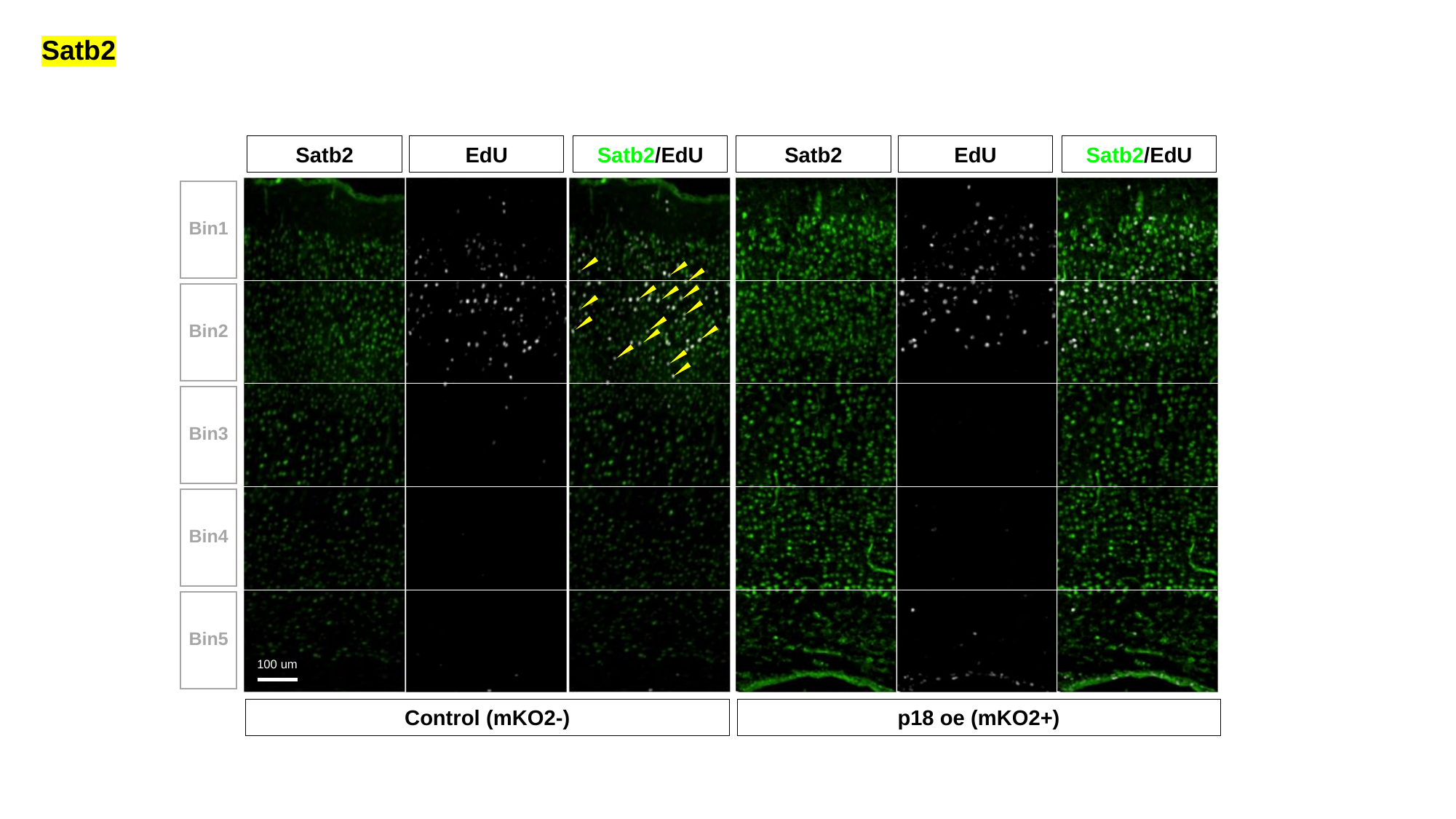

Satb2
Satb2
EdU
Satb2/EdU
Satb2
EdU
Satb2/EdU
Bin1
Bin2
Bin3
Bin4
Bin5
100 um
Control (mKO2-)
p18 oe (mKO2+)

## Slide 14
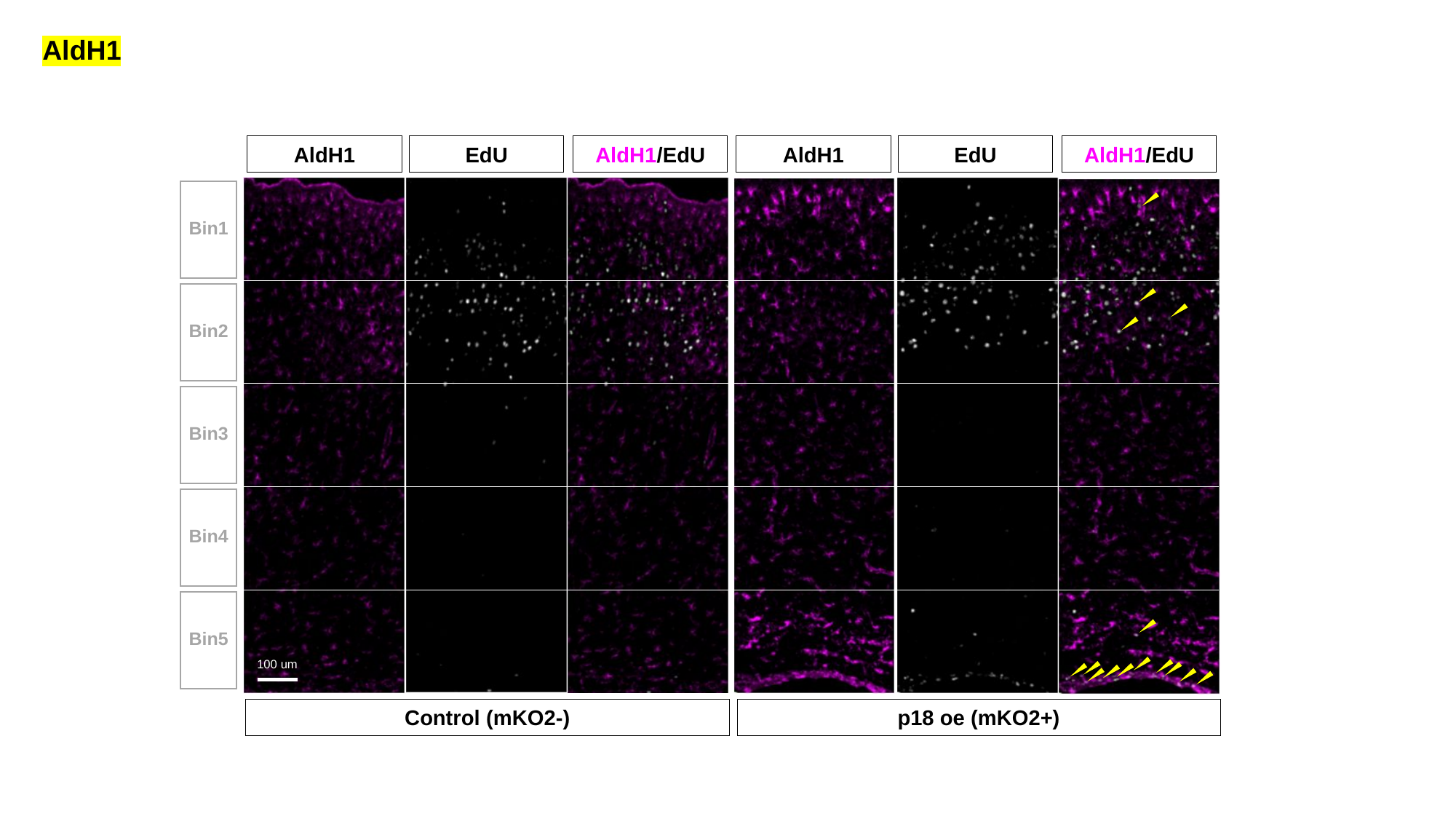

AldH1
AldH1
EdU
AldH1/EdU
AldH1
EdU
AldH1/EdU
100 um
Bin1
Bin2
Bin3
Bin4
Bin5
Control (mKO2-)
p18 oe (mKO2+)
