# Supplementary material for: Cyclin-dependent kinase inhibitor p18 regulates lineage transitions of excitatory neurons, astrocytes, and interneurons in the mouse cortex
Source: EMBO J. 2024 Dec 12;44(2):382–412. doi: 10.1038/s44318-024-00325-9 (PMC11730326; doi:10.1038/s44318-024-00325-9)
Supplement: Supplementary file 8 — Source data Fig. 6 [file 44318_2024_325_MOESM8_ESM.zip › GEO Accession viewer.pdf]

Series GSE265784

Status	**Private until Apr 23, 2027** **Private data, not to be shared or distributed without permission**
Title	Cyclin-dependent kinase inhibitors sequentially determine the neural-glial lineage boundaries in the developing cortex
Organism	Mus musculus
Experiment type	Expression profiling by high throughput sequencing
Summary	Neural stem cells (NSCs) can generate neurons and glia, but the regulatory mechanisms to invoke different lineages are largely unclear. Here, we addressed the role of cyclin-dependent kinase inhibitors (CDKIs) in the later stage of embryonic brain development. We found upregulations of p18 and p27 among CDKIs at the onset of astrocyte generation. Acute manipulation of p18 and p27 expressions in vivo revealed that the level of CDKIs at the transitional stage regulates the lineage switching between neurons and astrocytes. We generated a conditional knock-in mouse to induce p18 in NSCs. The transcriptome of micro-dissected tissue showed the enhanced activities of glial cell development and delta-notch signaling by the increased p18. Furthermore, Dlx2 induced olfactory bulb interneuron from multipotent intermediate cells with reduction of astrocyte in the late embryonic stage. Together, our results demonstrated the function of CDKIs in sequentially determining the boundary among different cellular lineages in the brain.
Overall design	To identify functional link between the CDKIs and the neural to astrocytic differentiation switching, we generated a conditional knock-in mouse containing a loxP-flanked stop sequence and p18-P2A-mKO2 in the ROSA26 locus.
Contributor(s)	Lee W, Kang B, Roh T, Kosodo Y
Citation missing	*Has this study been published? Please login to update or notify GEO. Note that private accession will be released, in accordance to guidelines.*
Submission date	Apr 24, 2024
Last update date	Apr 24, 2024
Contact name	Tae-Young Roh
Organization name	Ewha Womans University
Department	Life Sciences
Lab	Sysgem Genomics
Street address	52 Ewhayeodae-gil, Seodaemun-gu
City	Seoul
State/province	--- Select One ---
ZIP/Postal code	03760
Country	South Korea
Platforms (1)	GPL17021 Illumina HiSeq 2500 (Mus musculus)
Samples (12) More...	GSM8229150 E13.5, p18mKO2-, rep1<br>GSM8229151 E16.5, p18mKO2-, rep1<br>GSM8229152 E13.5, p18mKO2-, rep2

Relations

BioProject	PRJNA1104198

Supplementary file	Size	Download	File type/resource
GSE265784_RAW.tar		(http)(custom)	TAR (of RESULTS)

SRA Run Selector

Raw data are available in SRA
