# Supplementary material for: Cyclin-dependent kinase inhibitor p18 regulates lineage transitions of excitatory neurons, astrocytes, and interneurons in the mouse cortex
Source: EMBO J. 2024 Dec 12;44(2):382–412. doi: 10.1038/s44318-024-00325-9 (PMC11730326; doi:10.1038/s44318-024-00325-9)
Supplement: Supplementary file 9 — Source data Fig. 7 [file 44318_2024_325_MOESM9_ESM.zip › 7A/7A_a-e.pptx]

## Slide 1
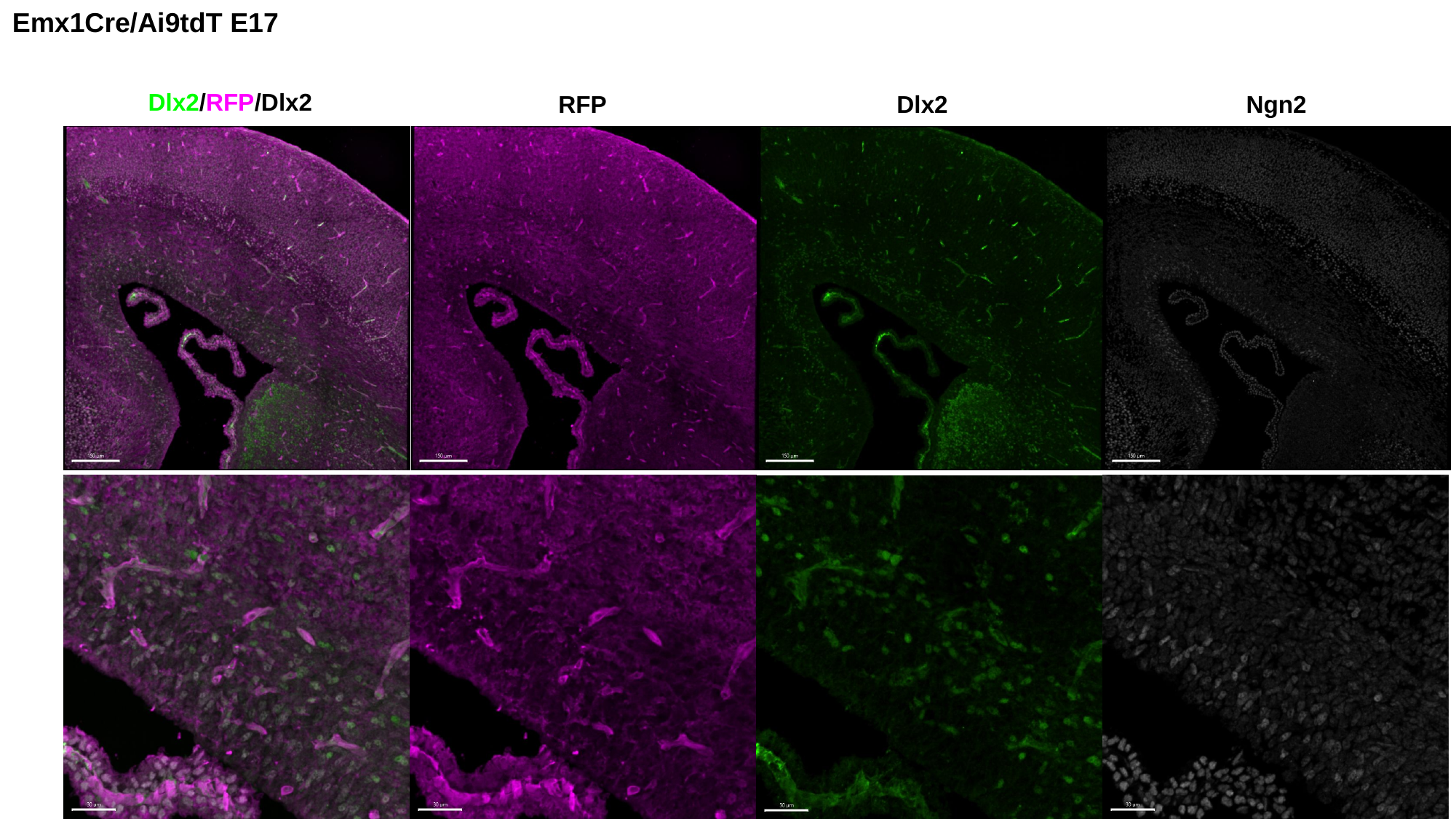

Emx1Cre/Ai9tdT E17
Dlx2/RFP/Dlx2
RFP
Dlx2
Ngn2

## Slide 2
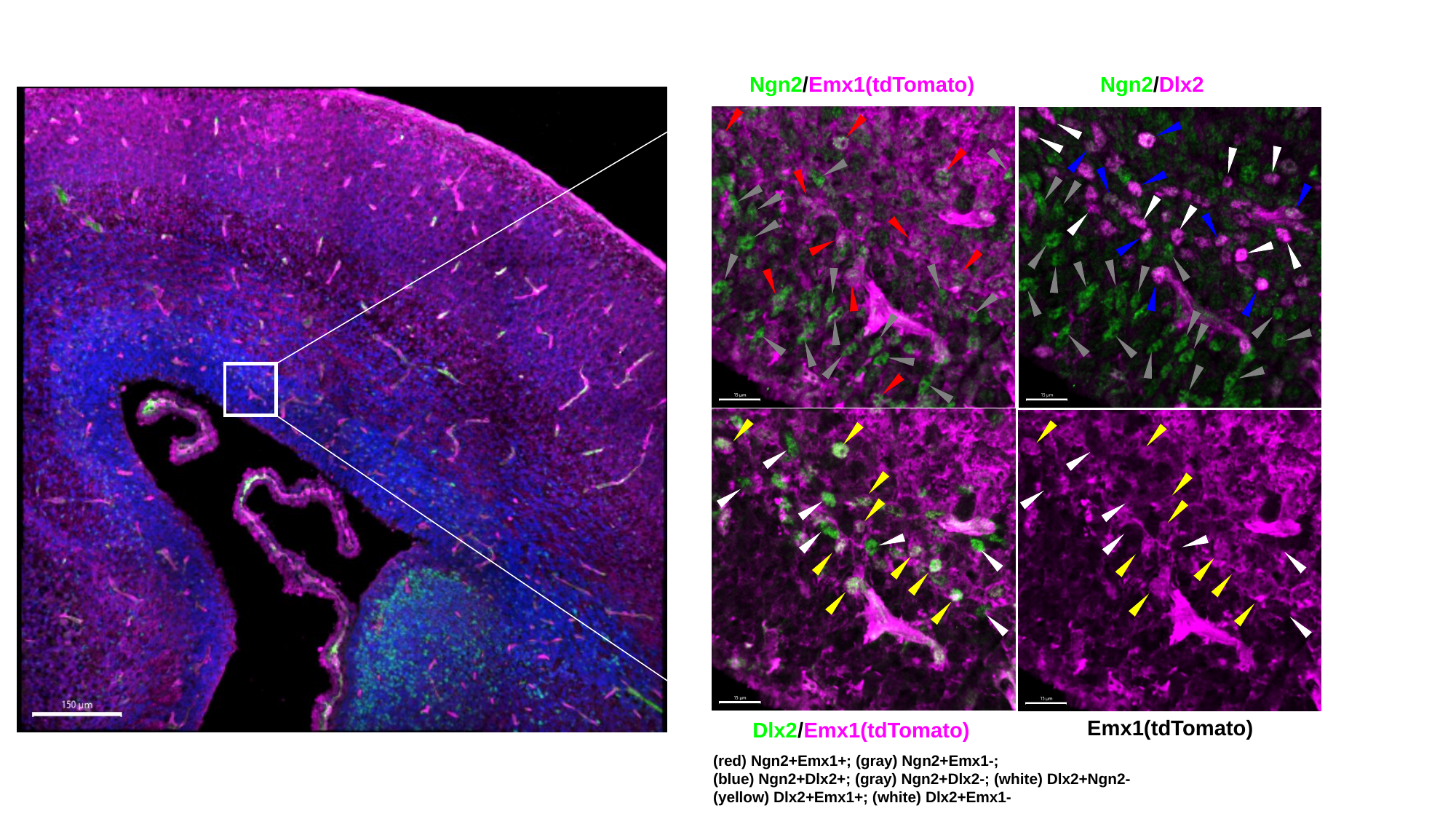

Ngn2/Dlx2
Ngn2/Emx1(tdTomato)
Dlx2
Emx1(tdTomato)
Dlx2/Emx1(tdTomato)
(red) Ngn2+Emx1+; (gray) Ngn2+Emx1-;
(blue) Ngn2+Dlx2+; (gray) Ngn2+Dlx2-; (white) Dlx2+Ngn2-
(yellow) Dlx2+Emx1+; (white) Dlx2+Emx1-

## Slide 3
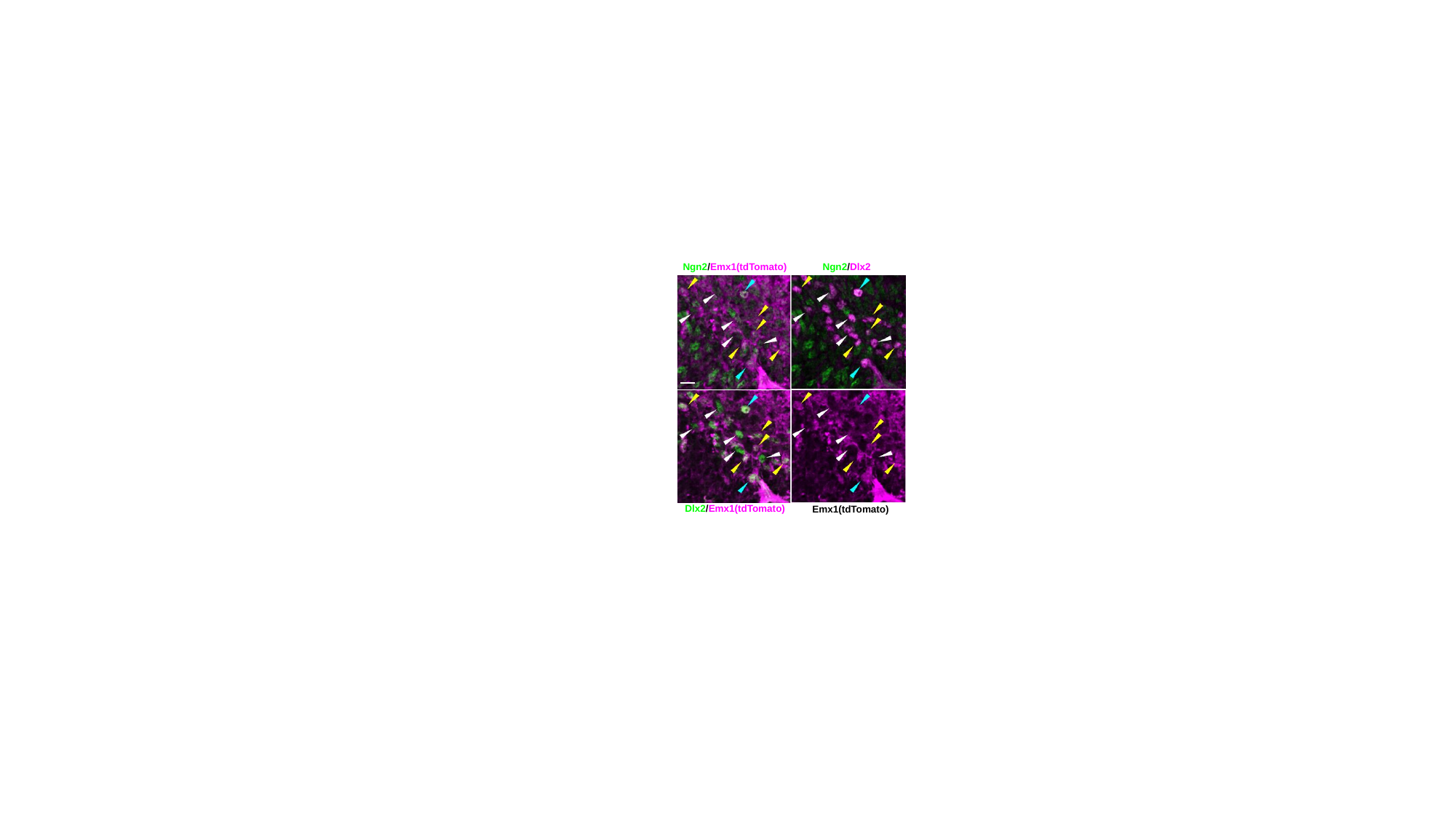

Ngn2/Dlx2
Ngn2/Emx1(tdTomato)
Dlx2
Dlx2/Emx1(tdTomato)
Emx1(tdTomato)
