# Supplementary material for: Cyclin-dependent kinase inhibitor p18 regulates lineage transitions of excitatory neurons, astrocytes, and interneurons in the mouse cortex
Source: EMBO J. 2024 Dec 12;44(2):382–412. doi: 10.1038/s44318-024-00325-9 (PMC11730326; doi:10.1038/s44318-024-00325-9)

## Slide 1
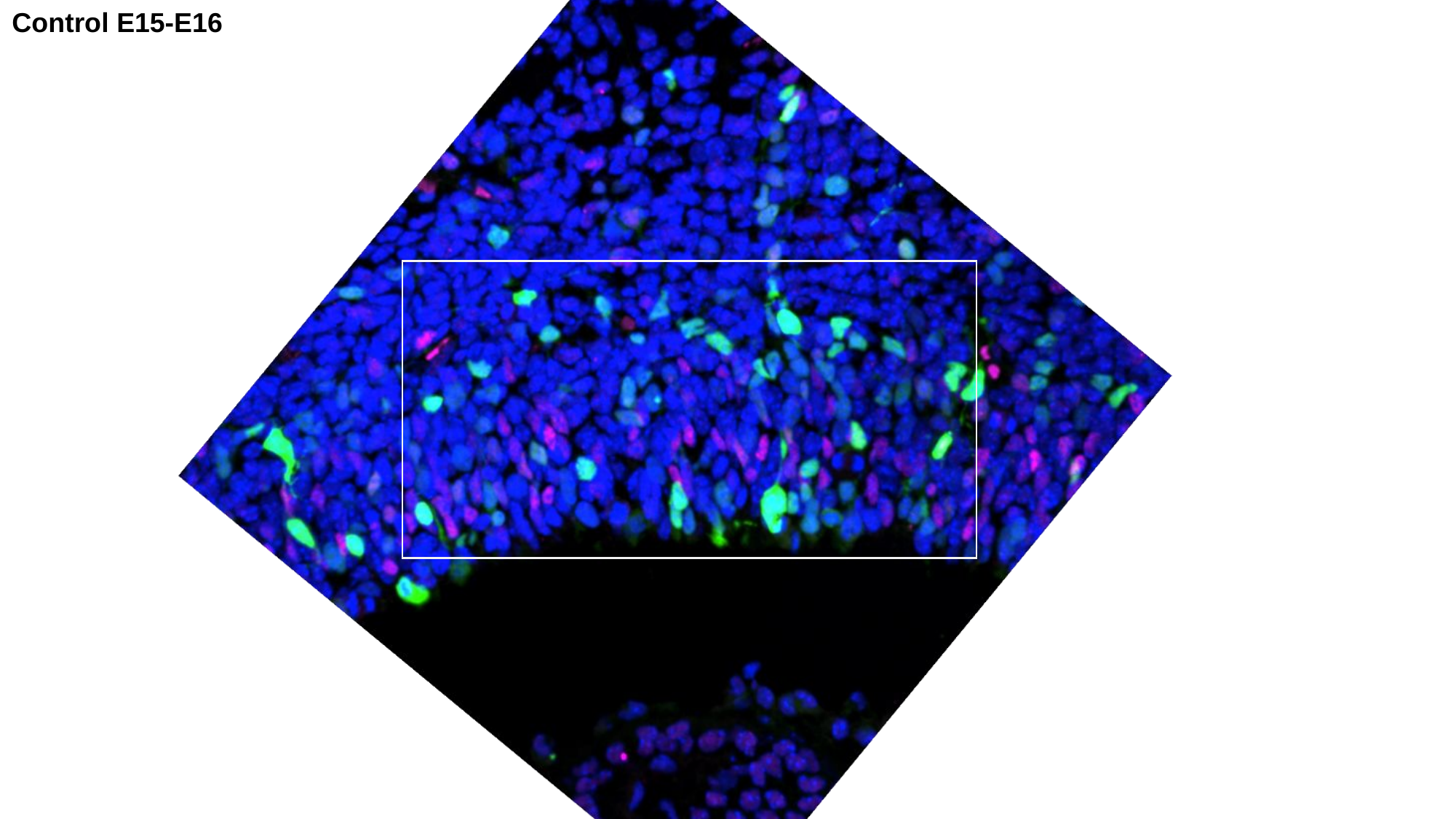

Control E15-E16

## Slide 2
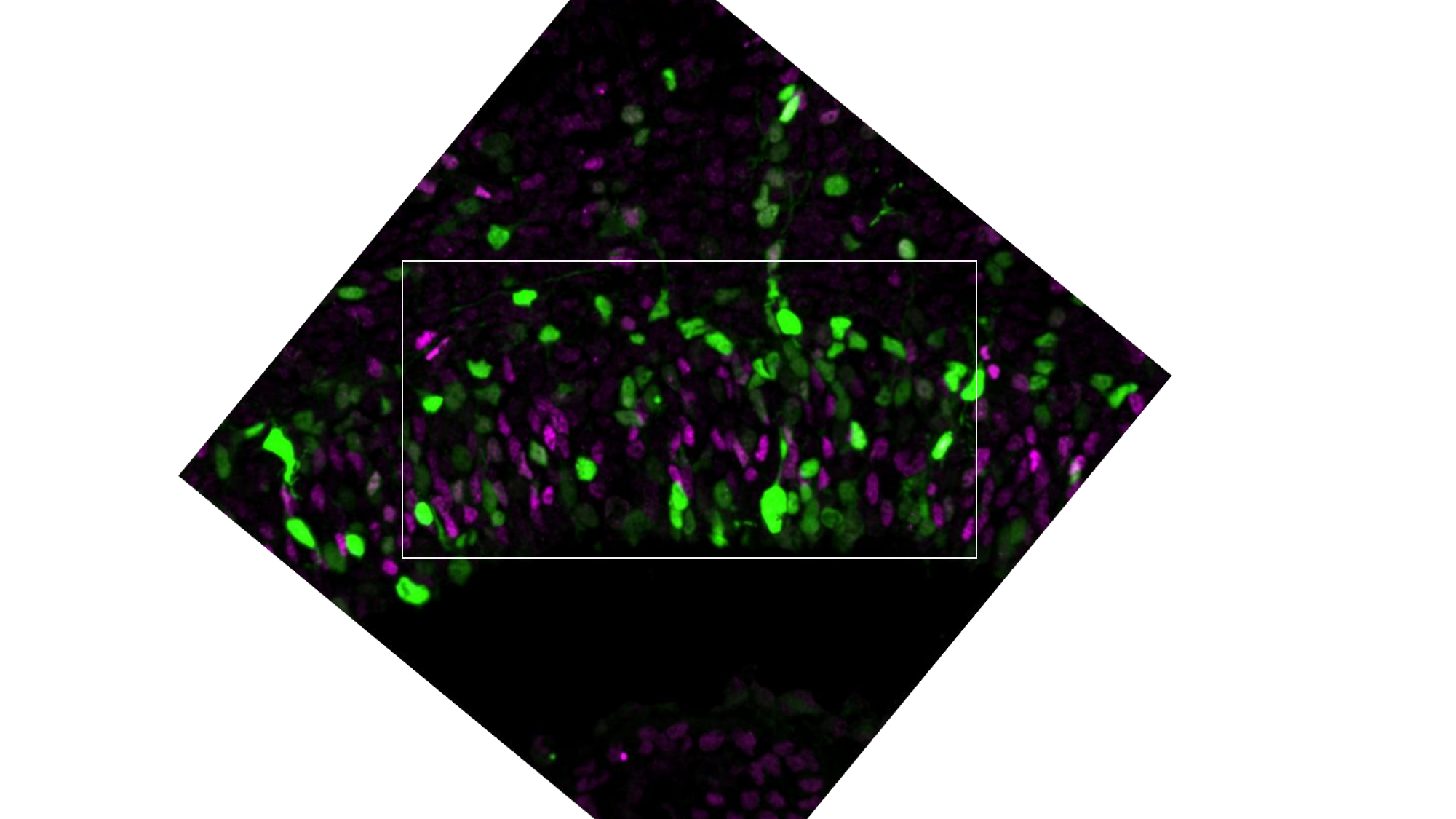

## Slide 3
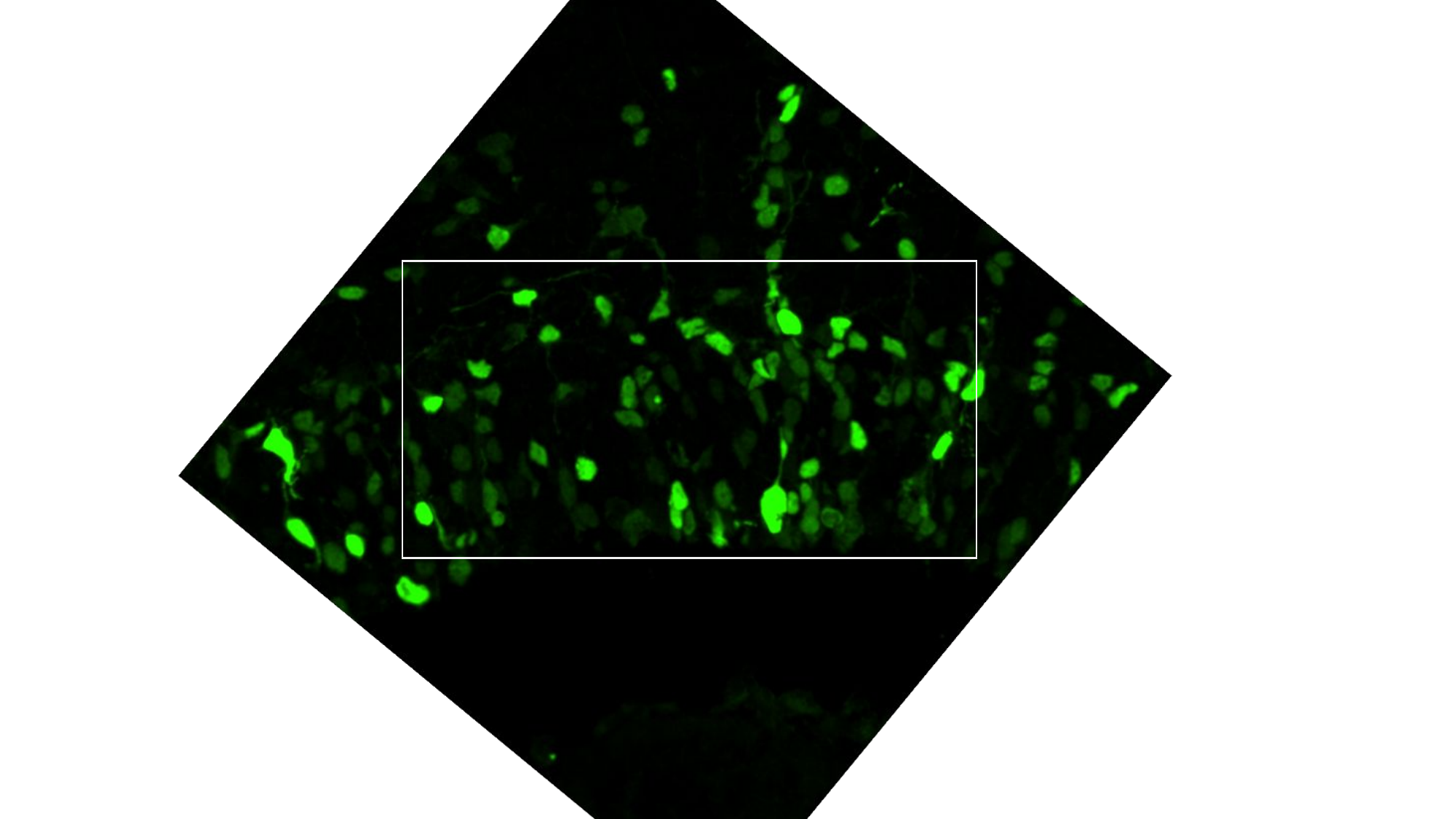

## Slide 4
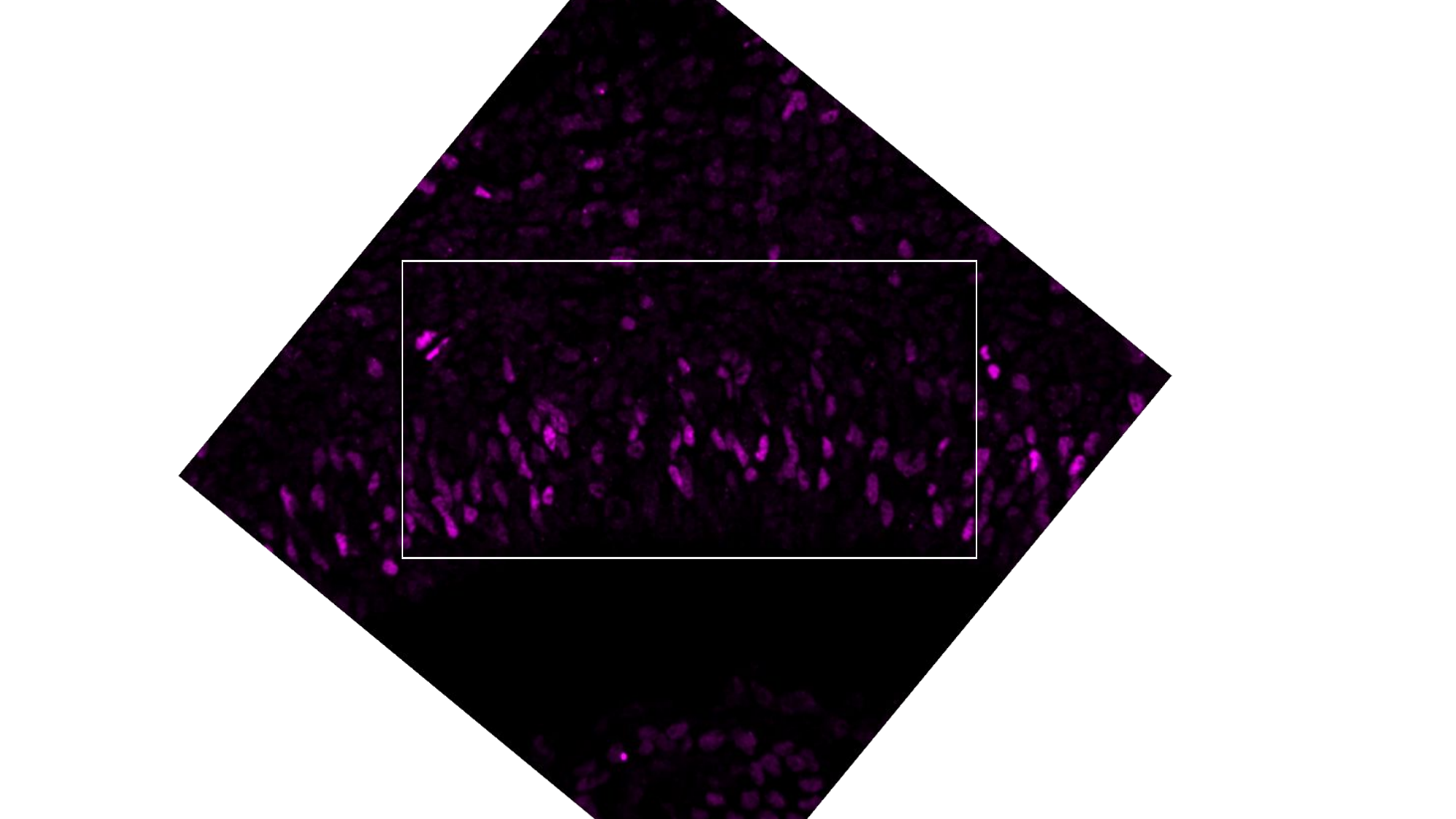

## Slide 5
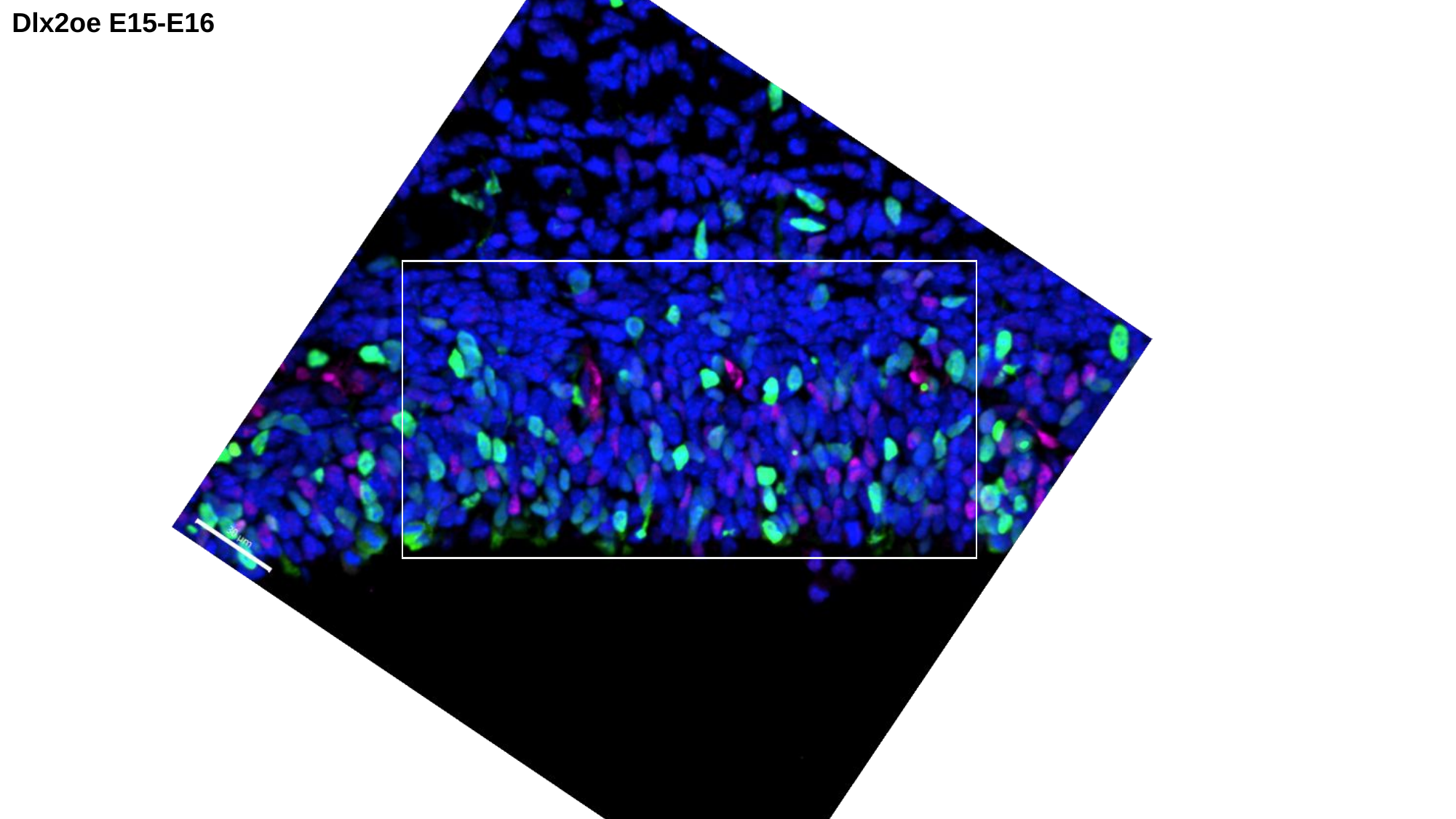

Dlx2oe E15-E16

## Slide 6
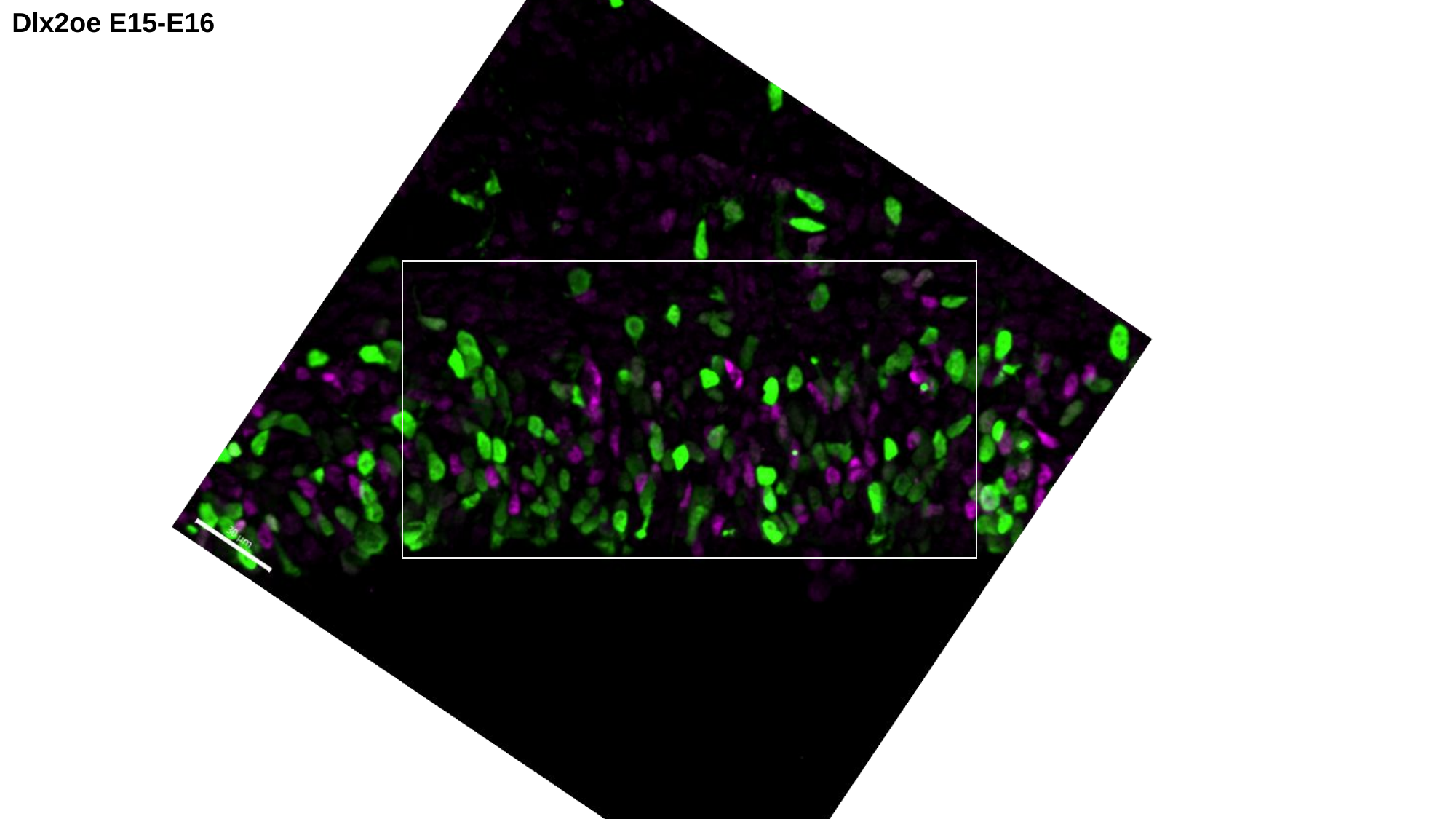

Dlx2oe E15-E16

## Slide 7
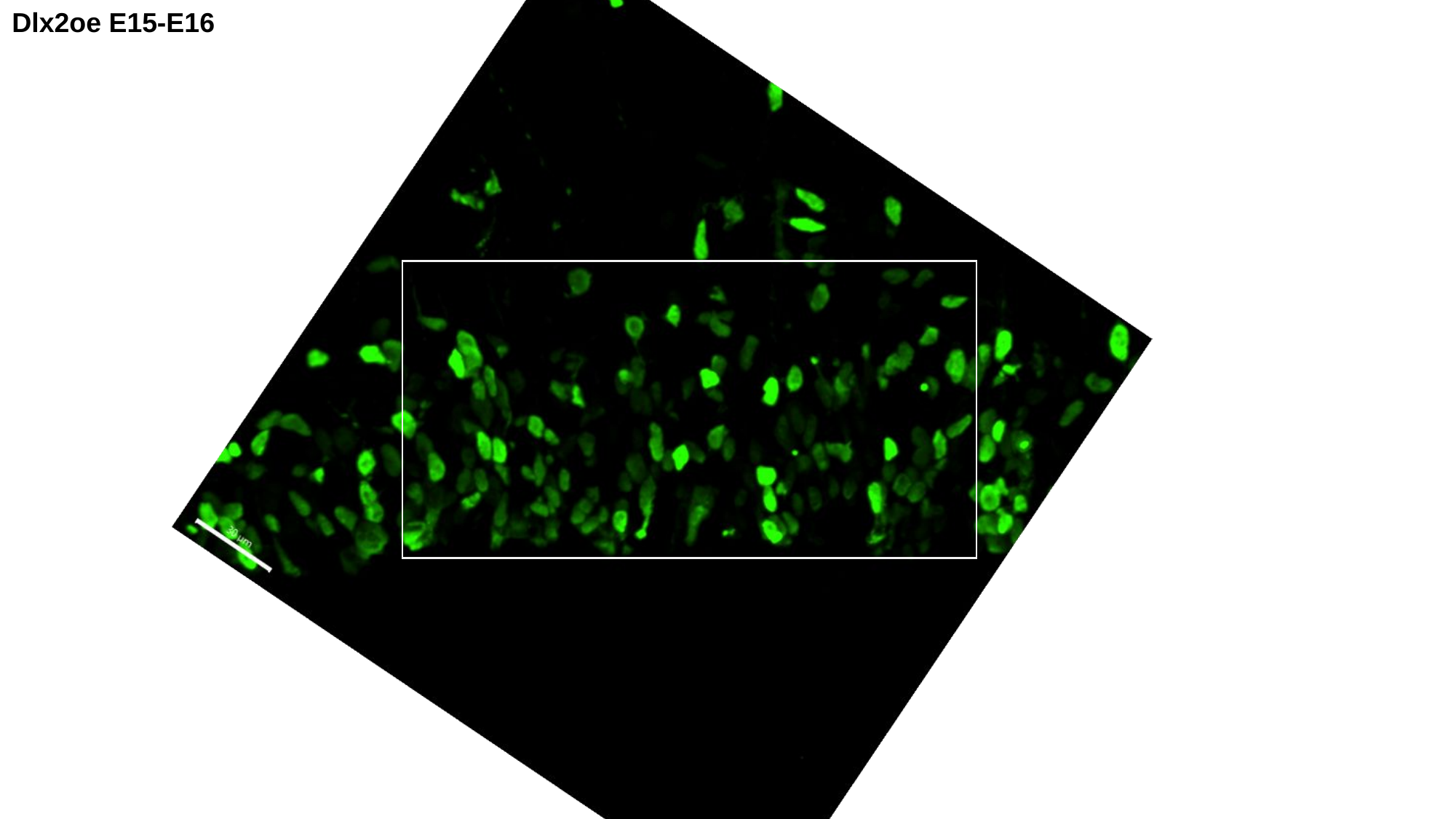

Dlx2oe E15-E16

## Slide 8
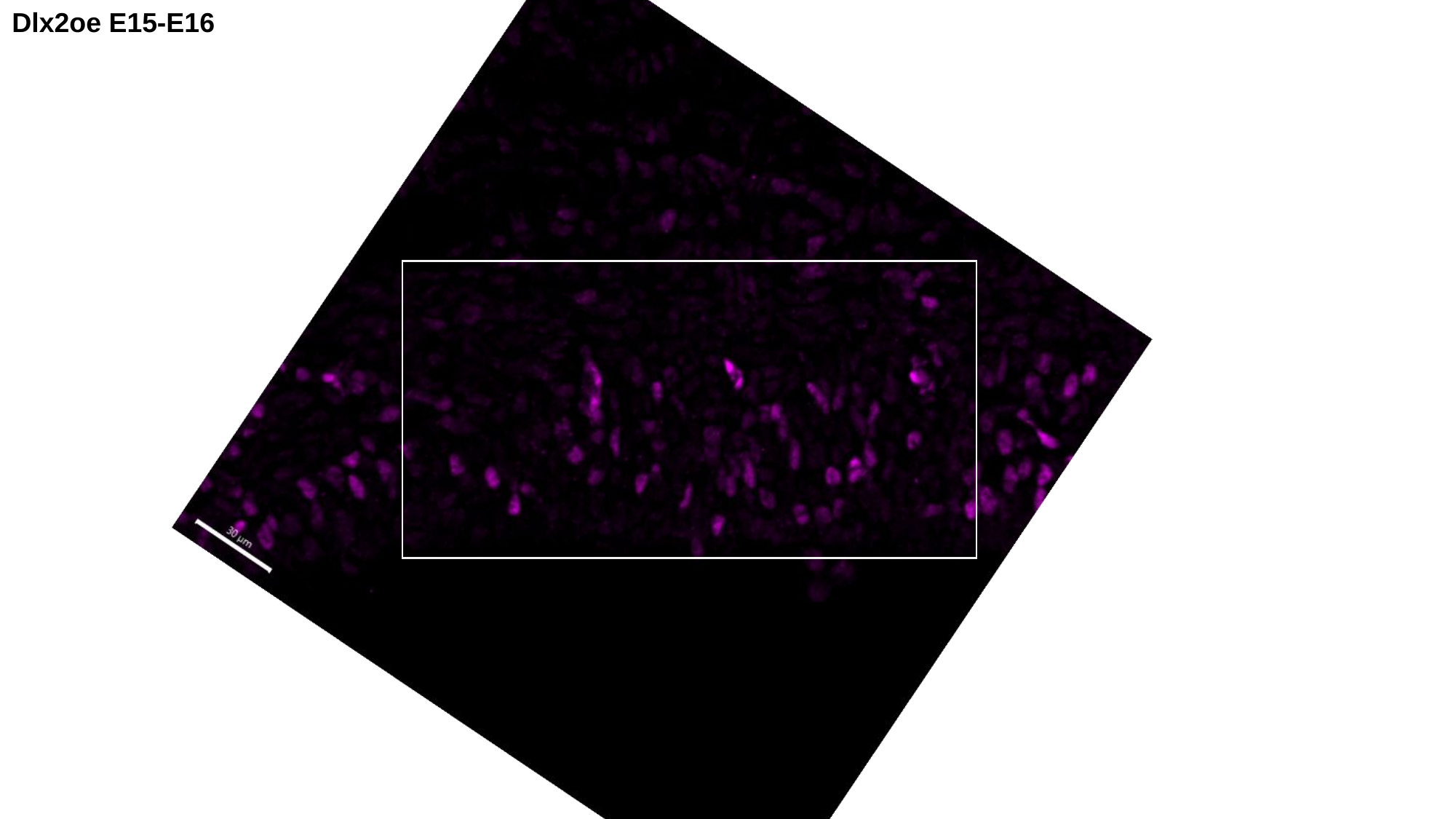

Dlx2oe E15-E16

## Slide 9
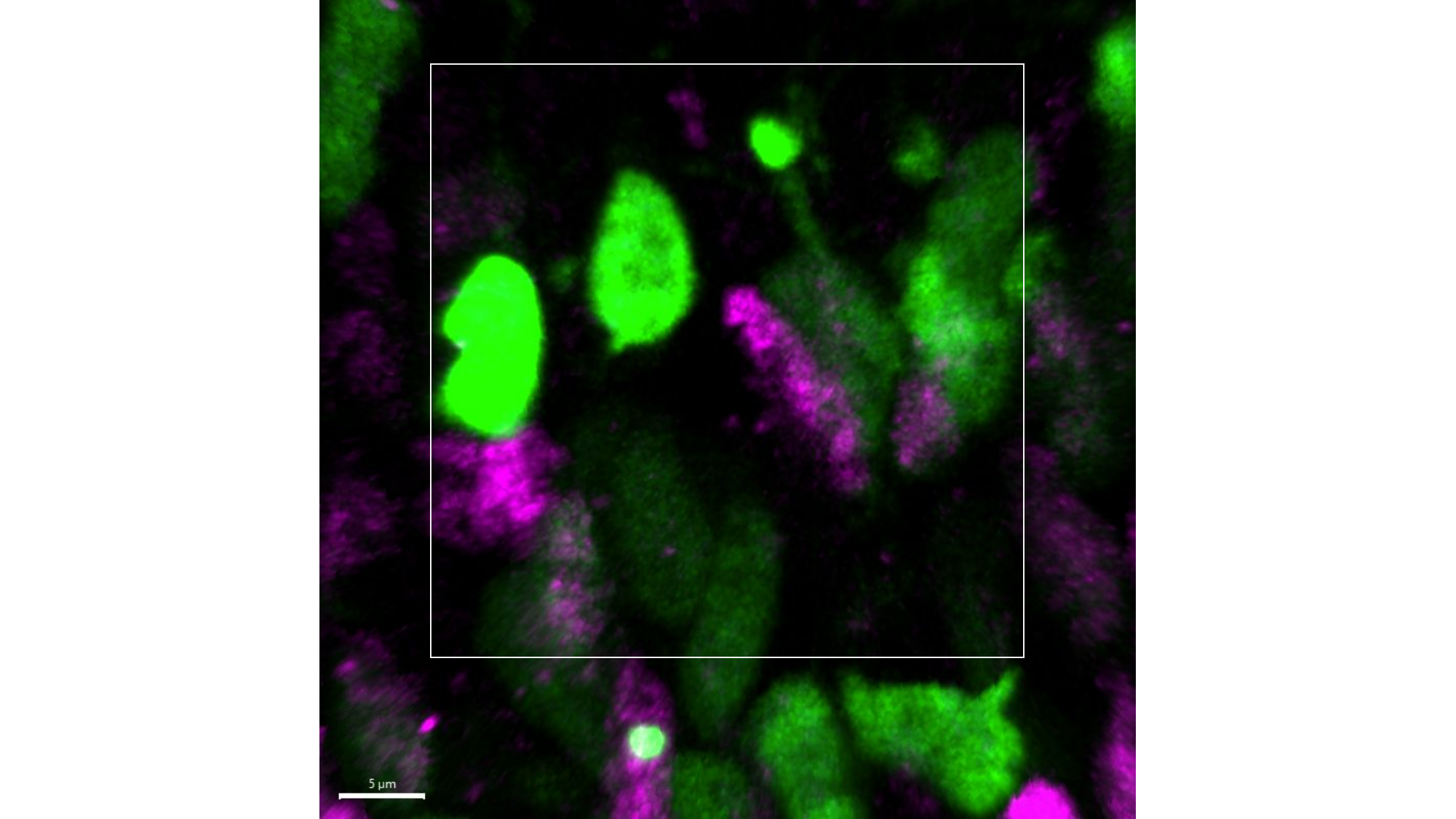

Supplement: Supplementary file 9 — Source data Fig. 7 [file 44318_2024_325_MOESM9_ESM.zip › 7D.pptx]
