# Supplementary material for: Cyclin-dependent kinase inhibitor p18 regulates lineage transitions of excitatory neurons, astrocytes, and interneurons in the mouse cortex
Source: EMBO J. 2024 Dec 12;44(2):382–412. doi: 10.1038/s44318-024-00325-9 (PMC11730326; doi:10.1038/s44318-024-00325-9)

## Slide 1
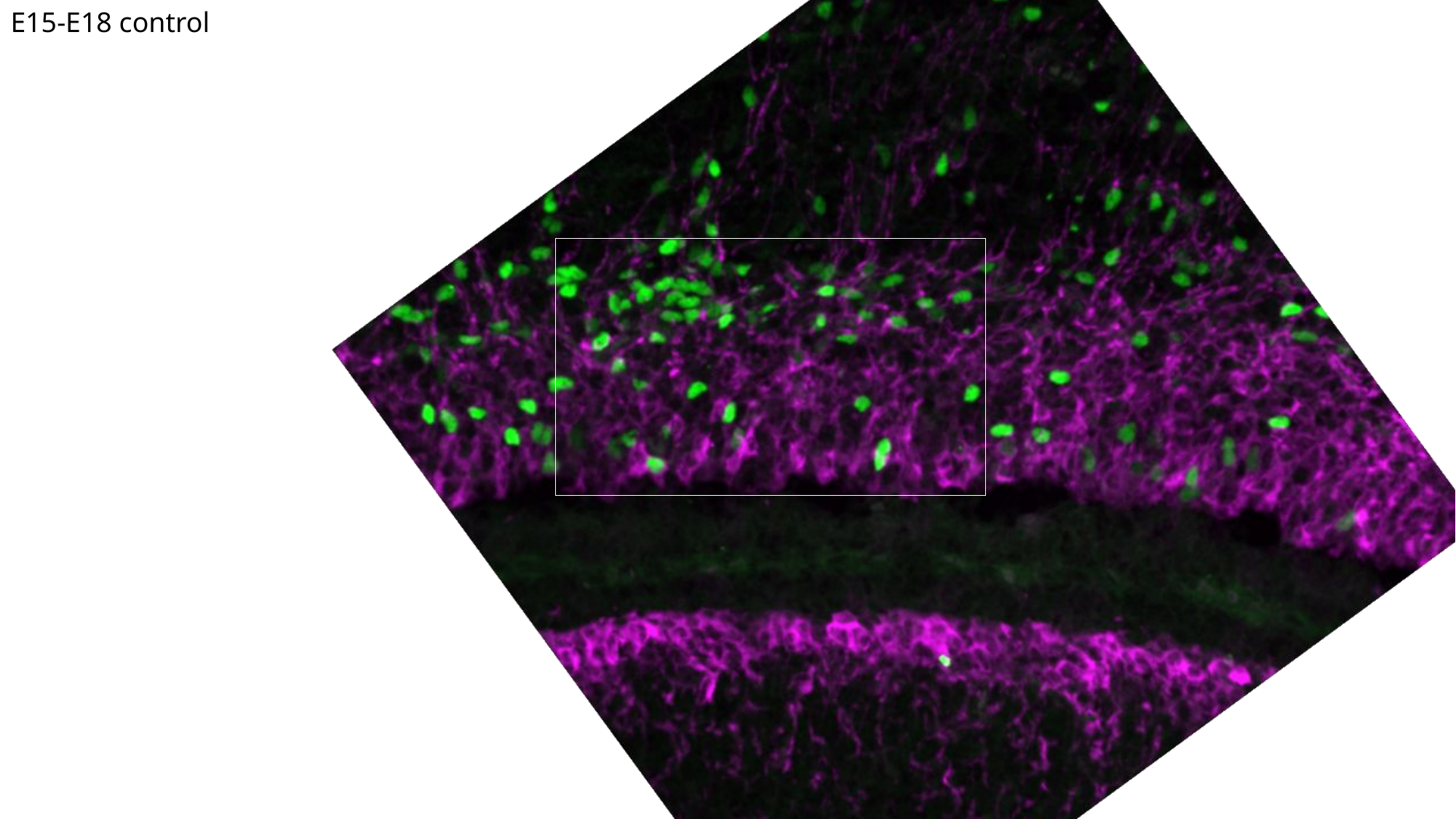

E15-E18 control

## Slide 2
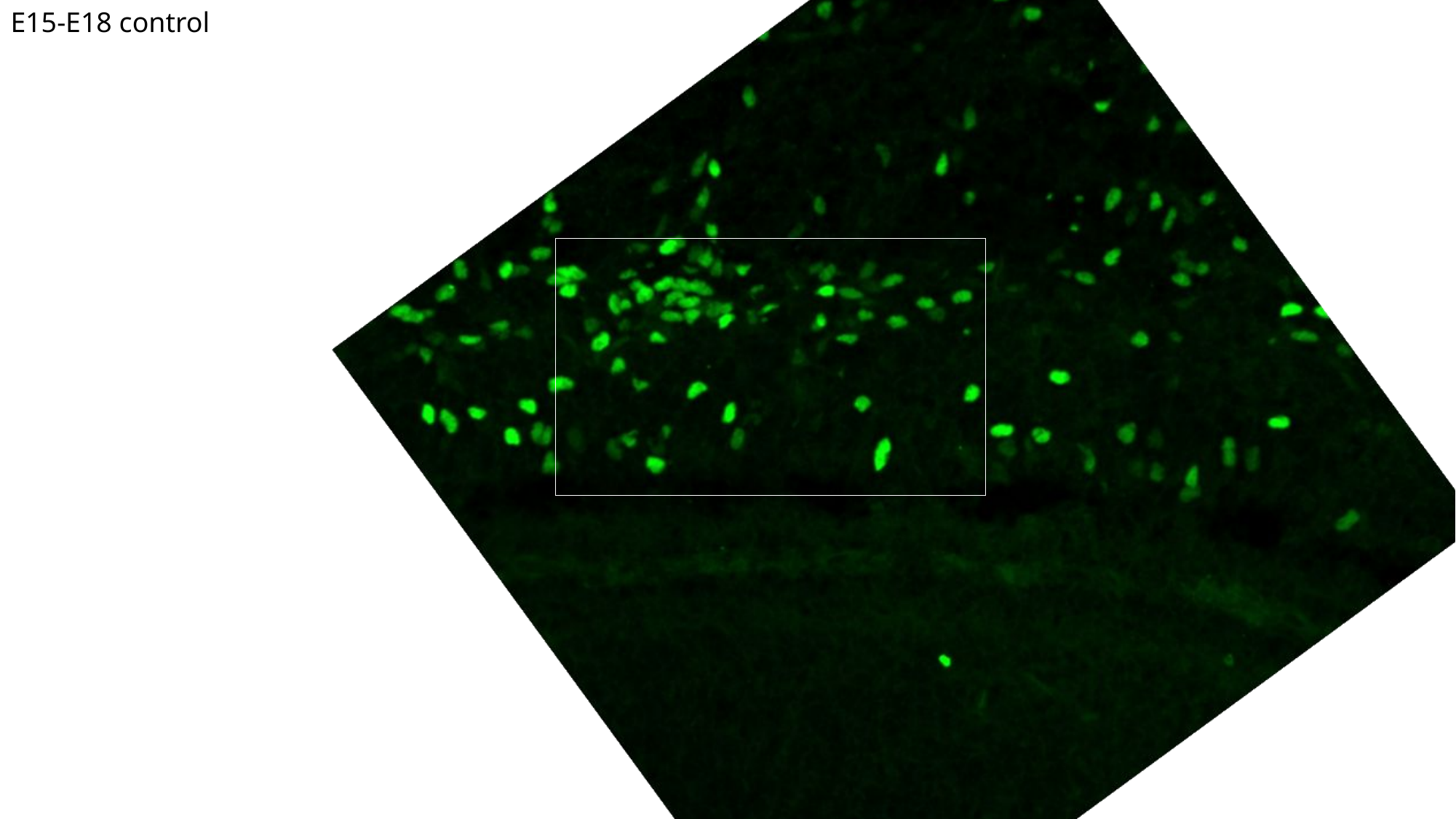

E15-E18 control

## Slide 3
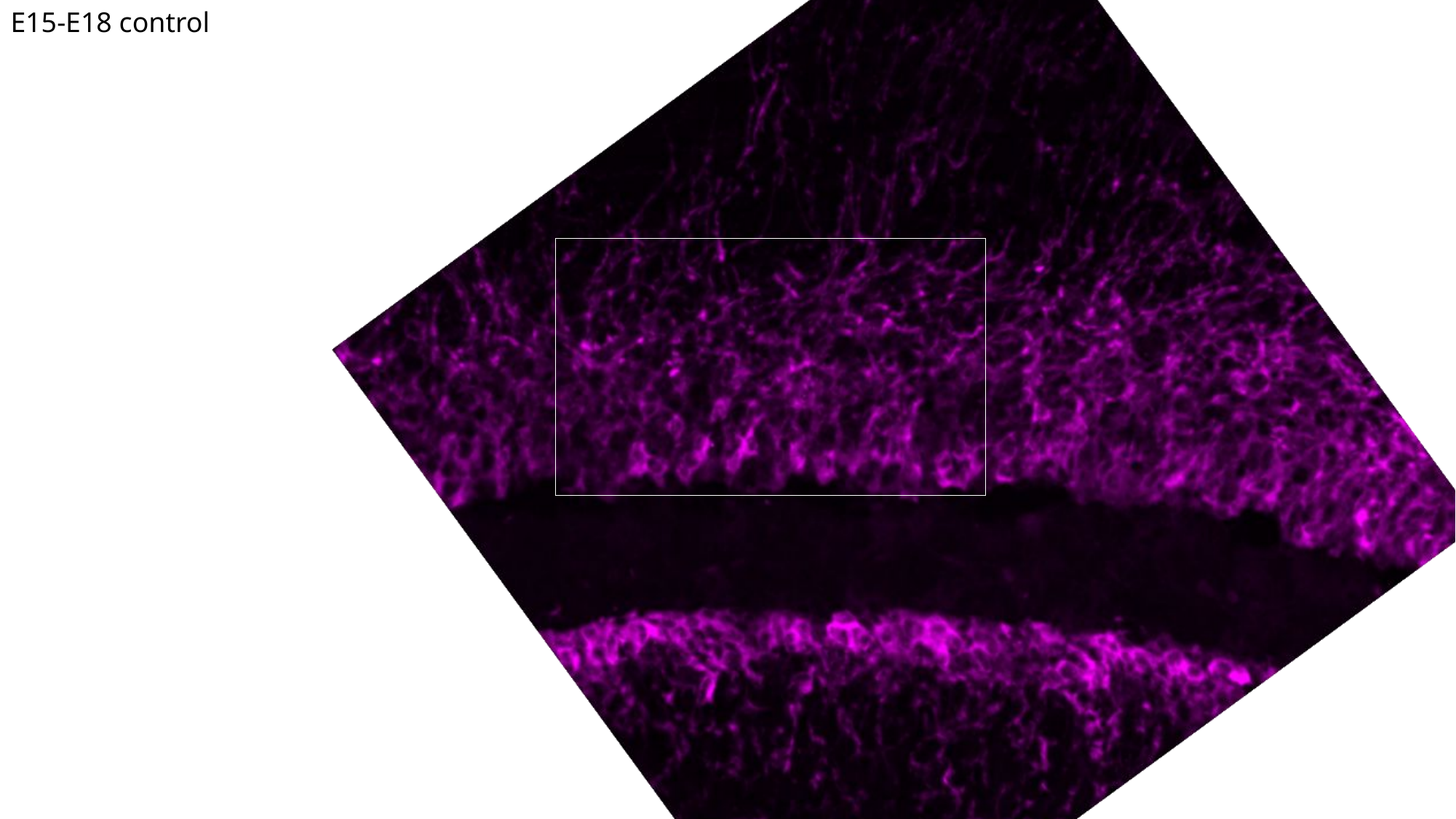

E15-E18 control

## Slide 4
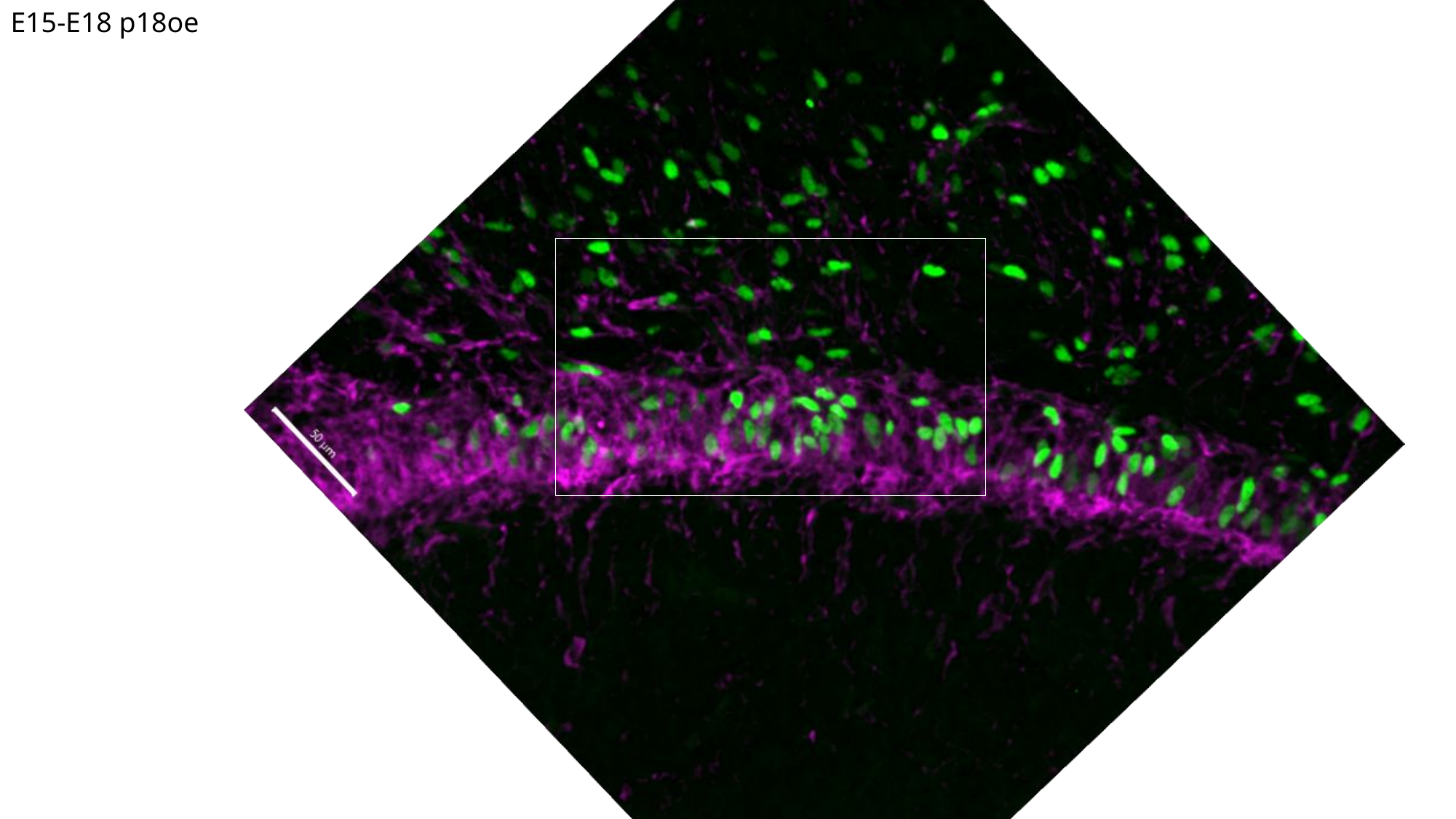

E15-E18 p18oe

## Slide 5
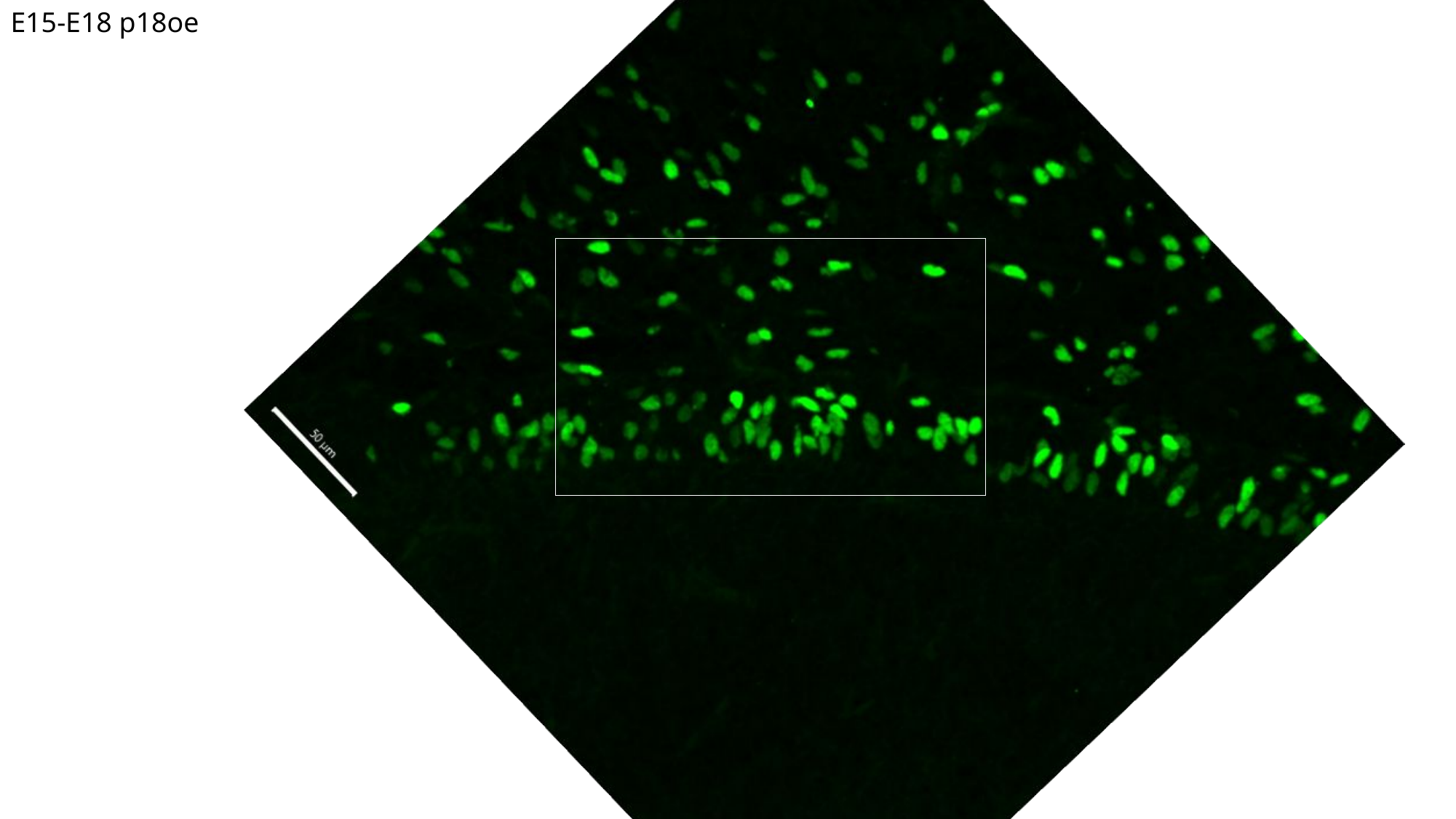

E15-E18 p18oe

## Slide 6
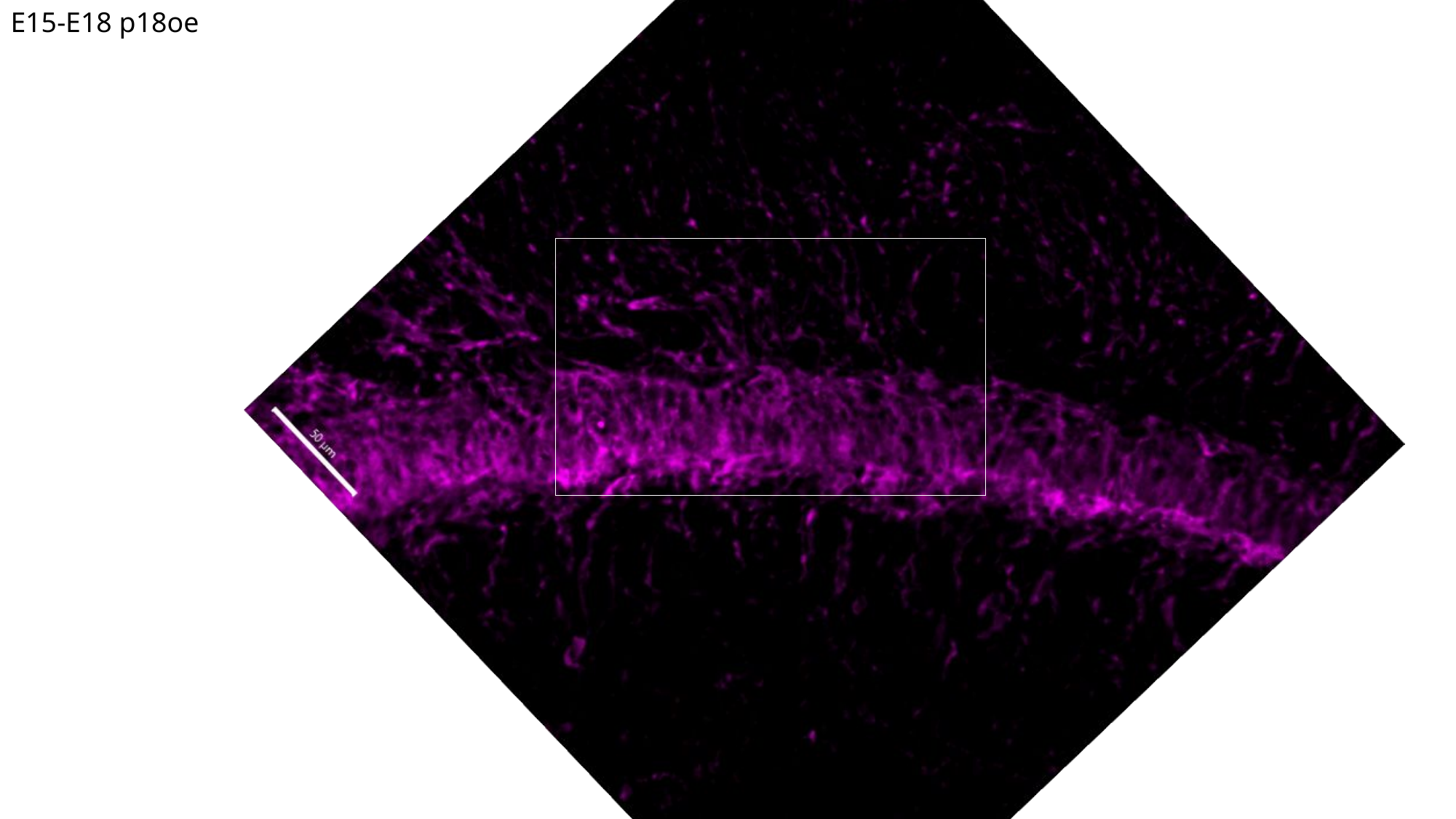

E15-E18 p18oe

## Slide 7
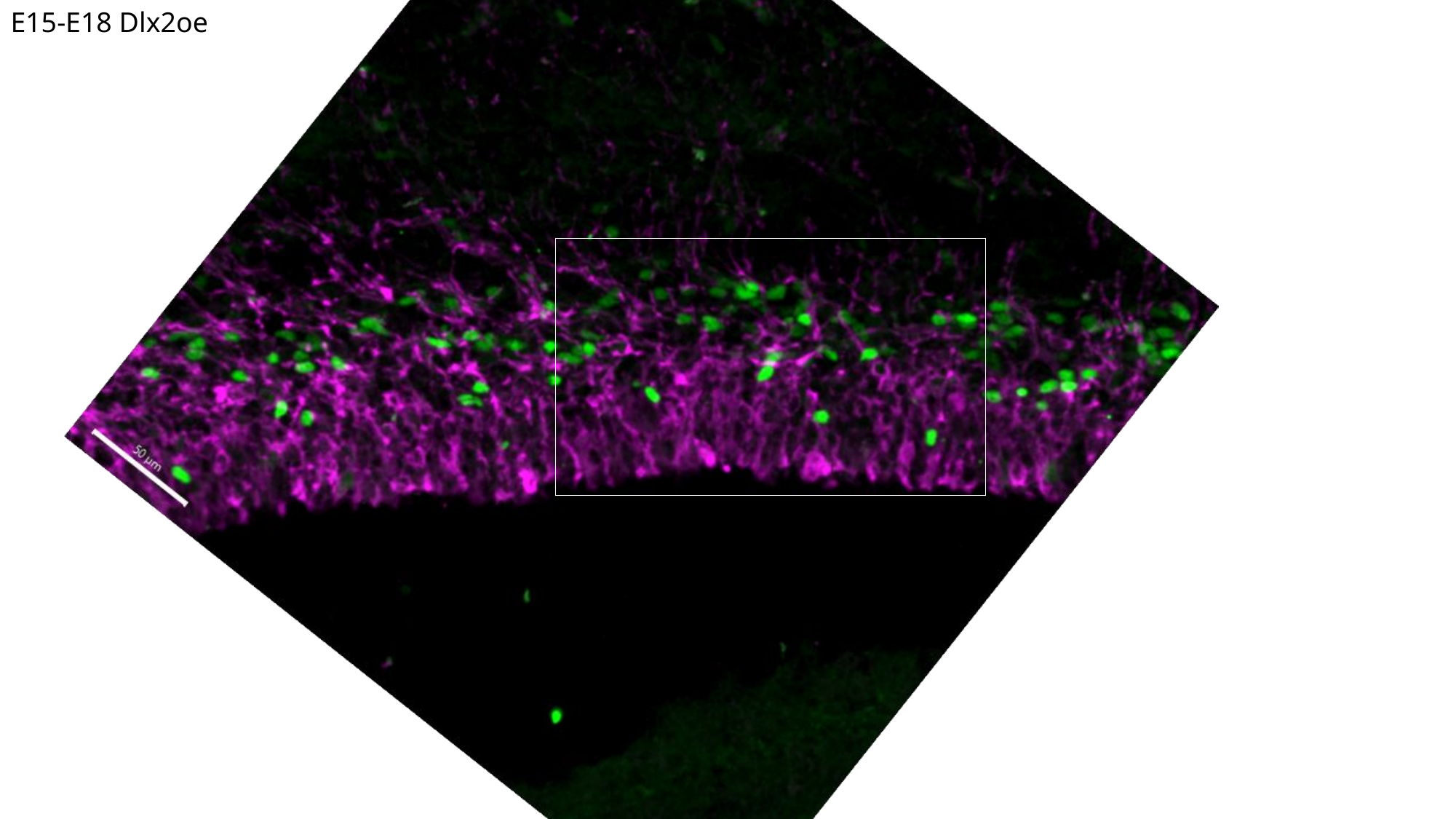

E15-E18 Dlx2oe

## Slide 8
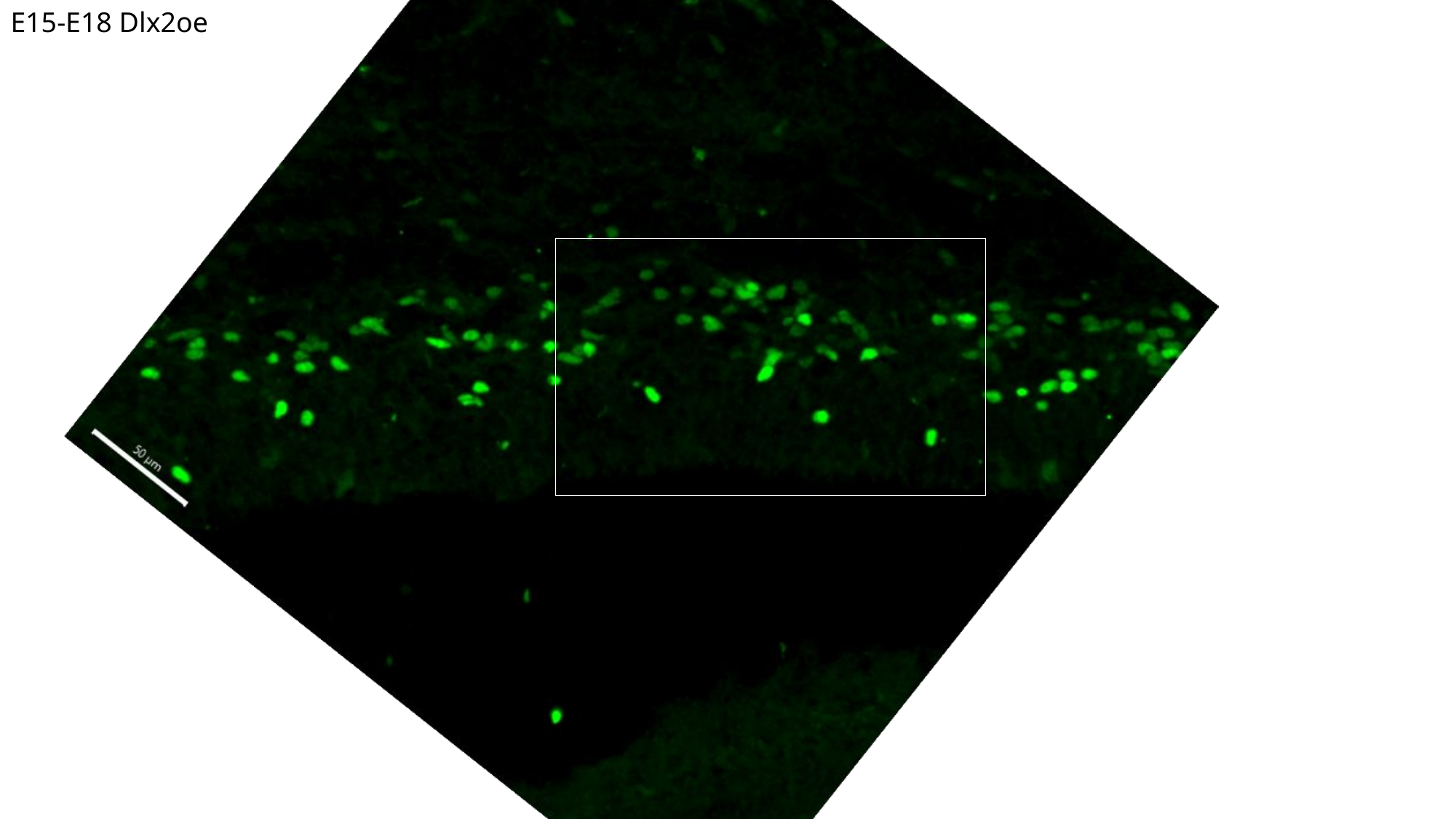

E15-E18 Dlx2oe

## Slide 9
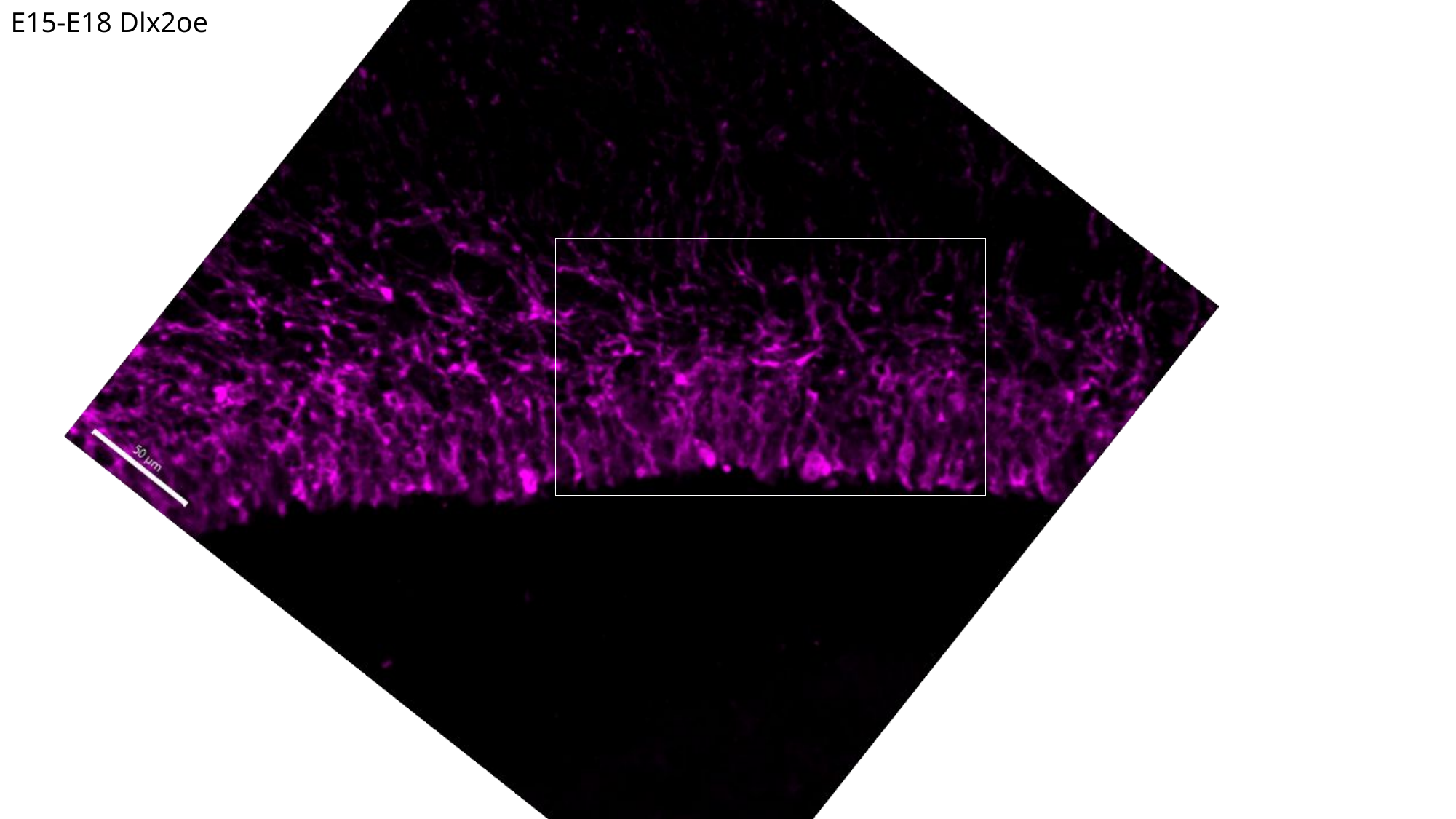

E15-E18 Dlx2oe

## Slide 10
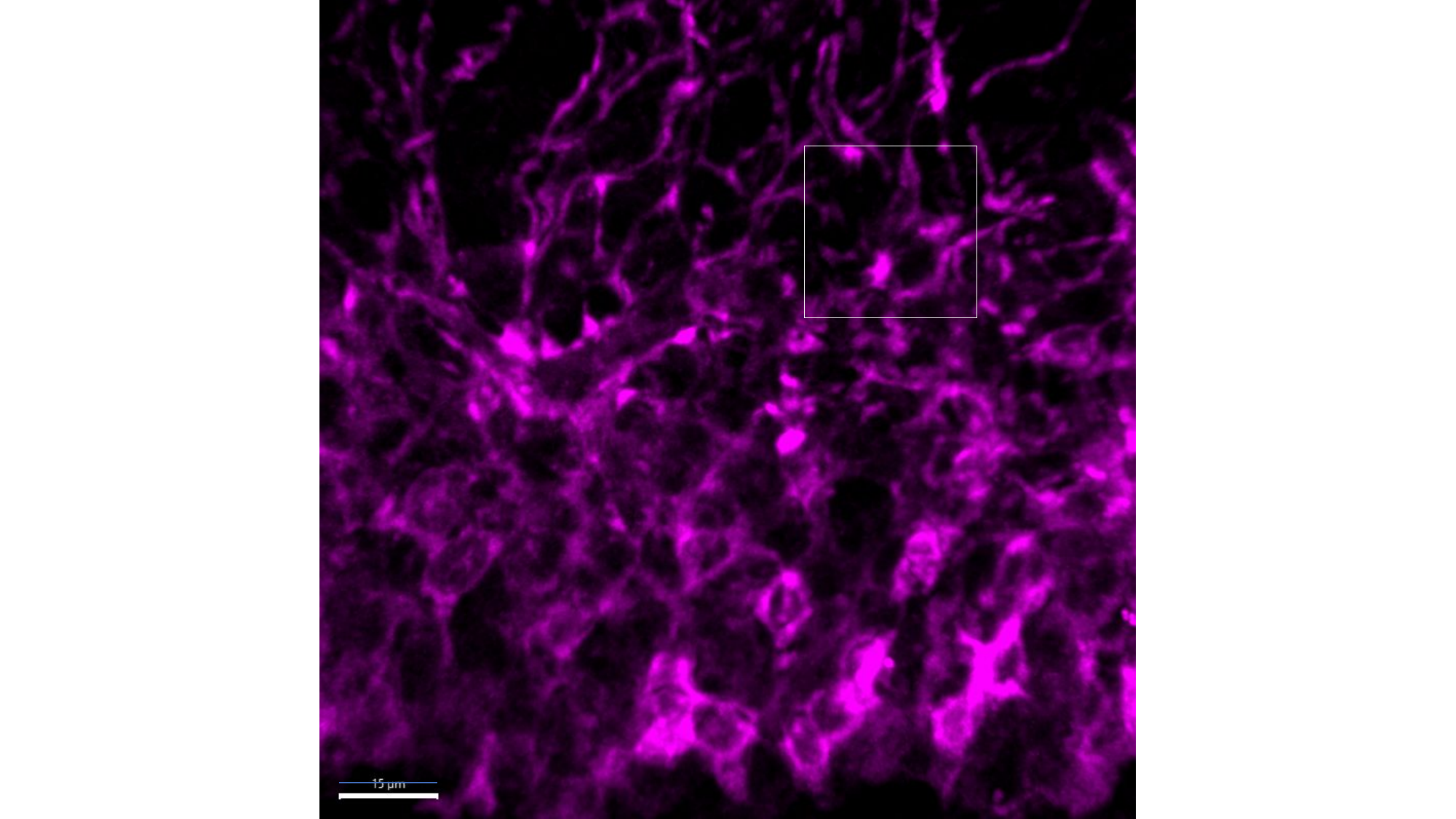

## Slide 11
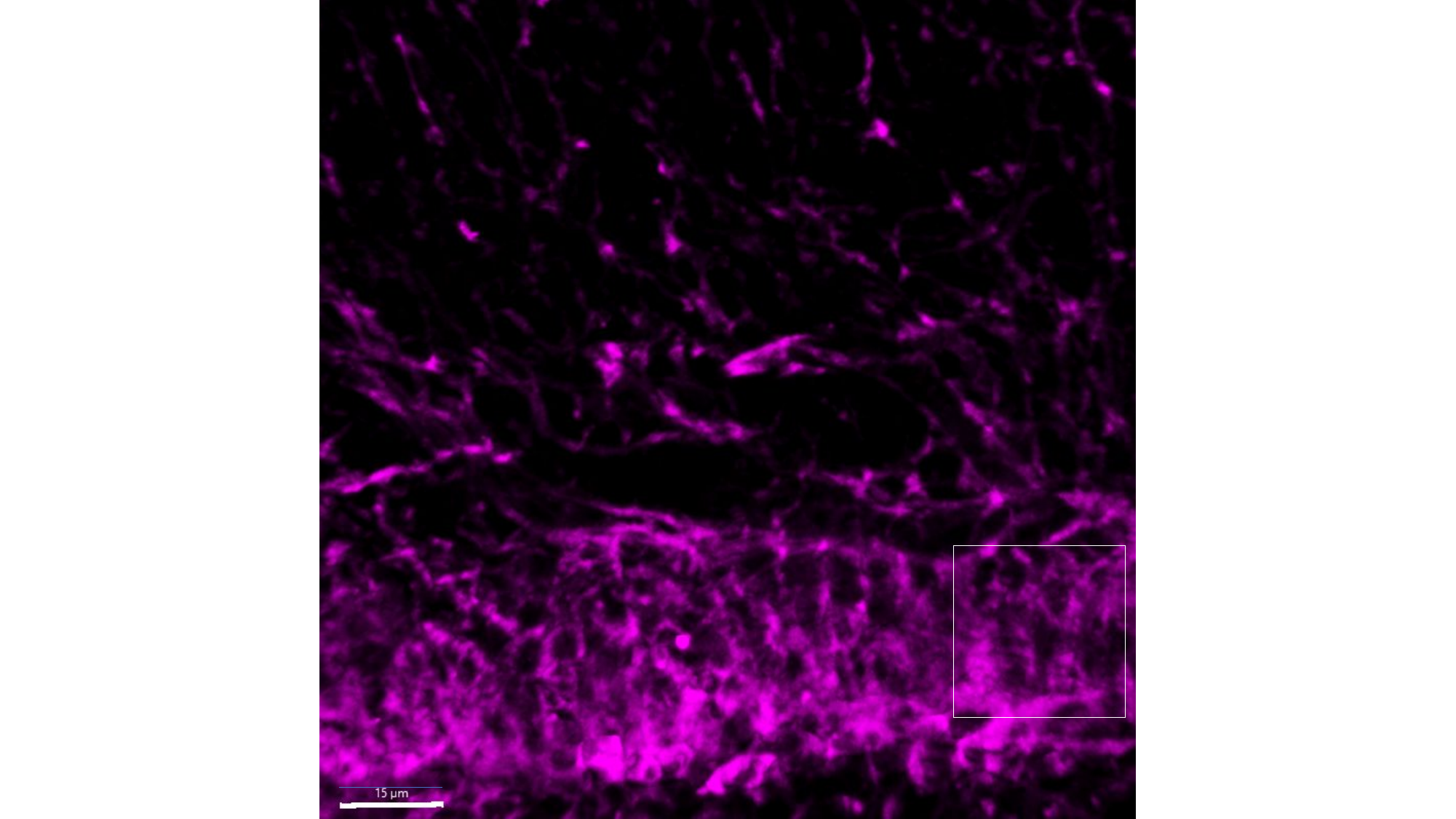

## Slide 12
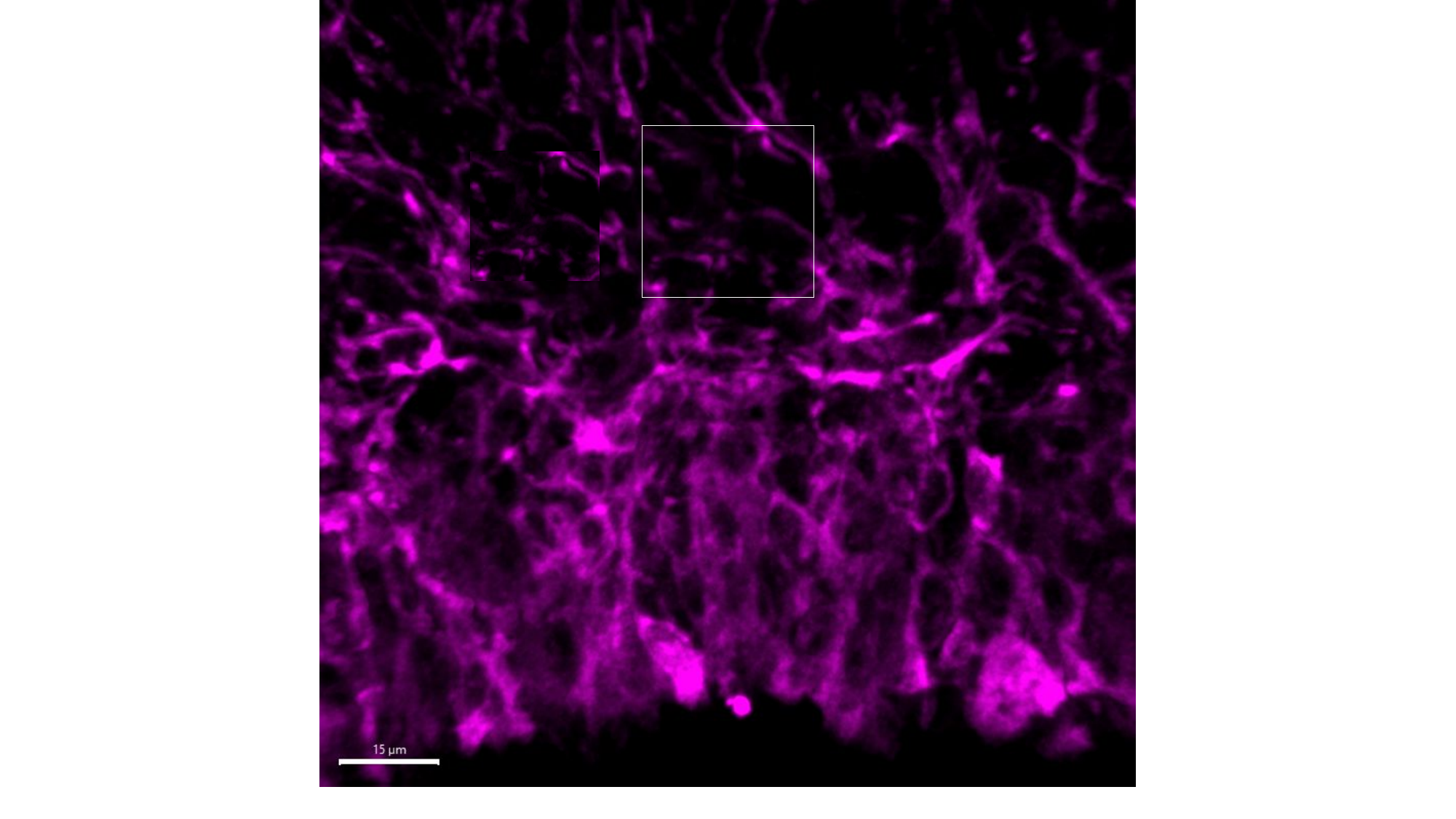

Supplement: Supplementary file 10 — Source data Fig. 8 [file 44318_2024_325_MOESM10_ESM.zip › 8A.pptx]
