# Supplementary material for: Cyclin-dependent kinase inhibitor p18 regulates lineage transitions of excitatory neurons, astrocytes, and interneurons in the mouse cortex
Source: EMBO J. 2024 Dec 12;44(2):382–412. doi: 10.1038/s44318-024-00325-9 (PMC11730326; doi:10.1038/s44318-024-00325-9)
Supplement: Supplementary file 10 — Source data Fig. 8 [file 44318_2024_325_MOESM10_ESM.zip › 8G.pptx]

## Slide 1
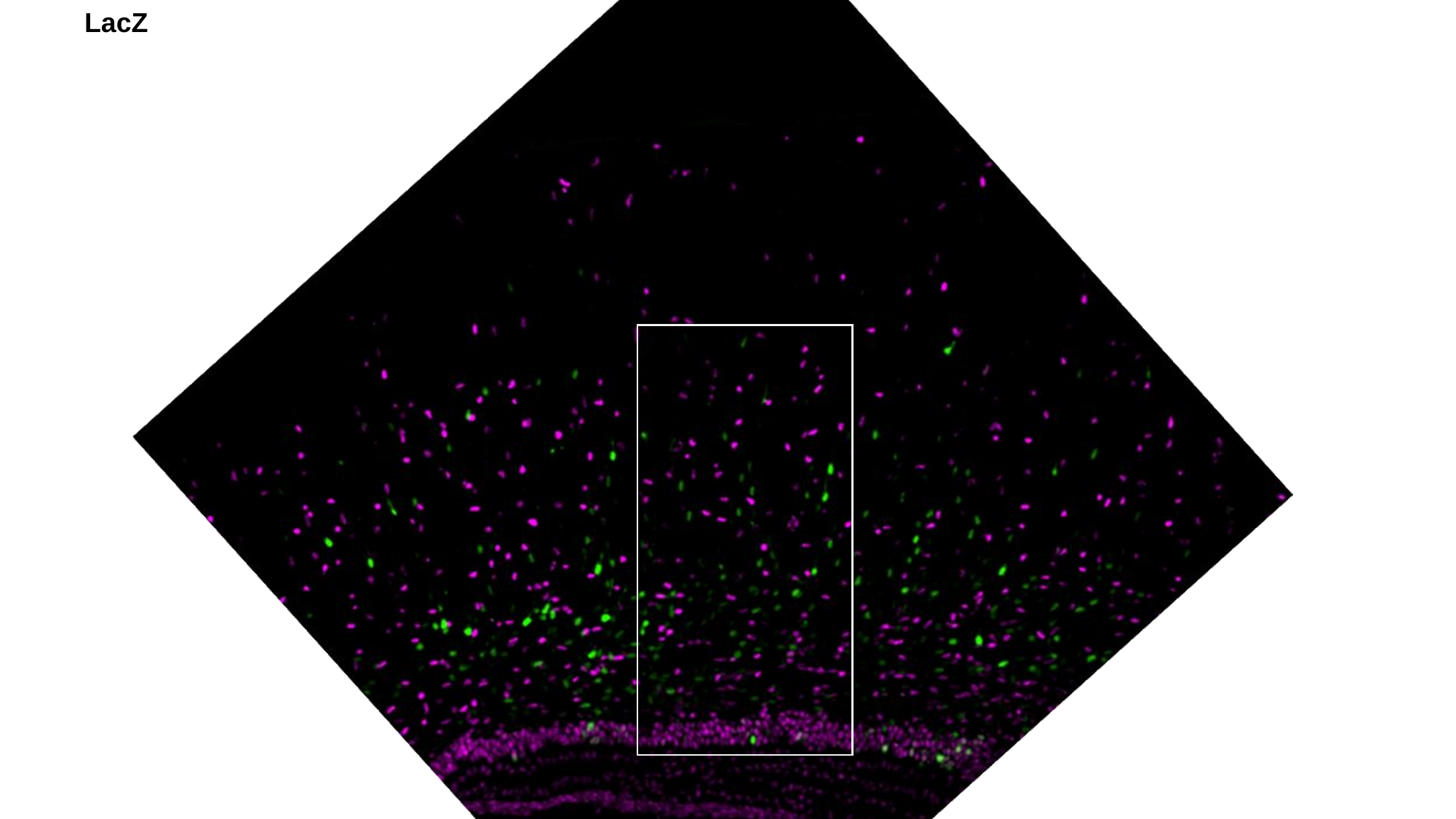

LacZ

## Slide 2
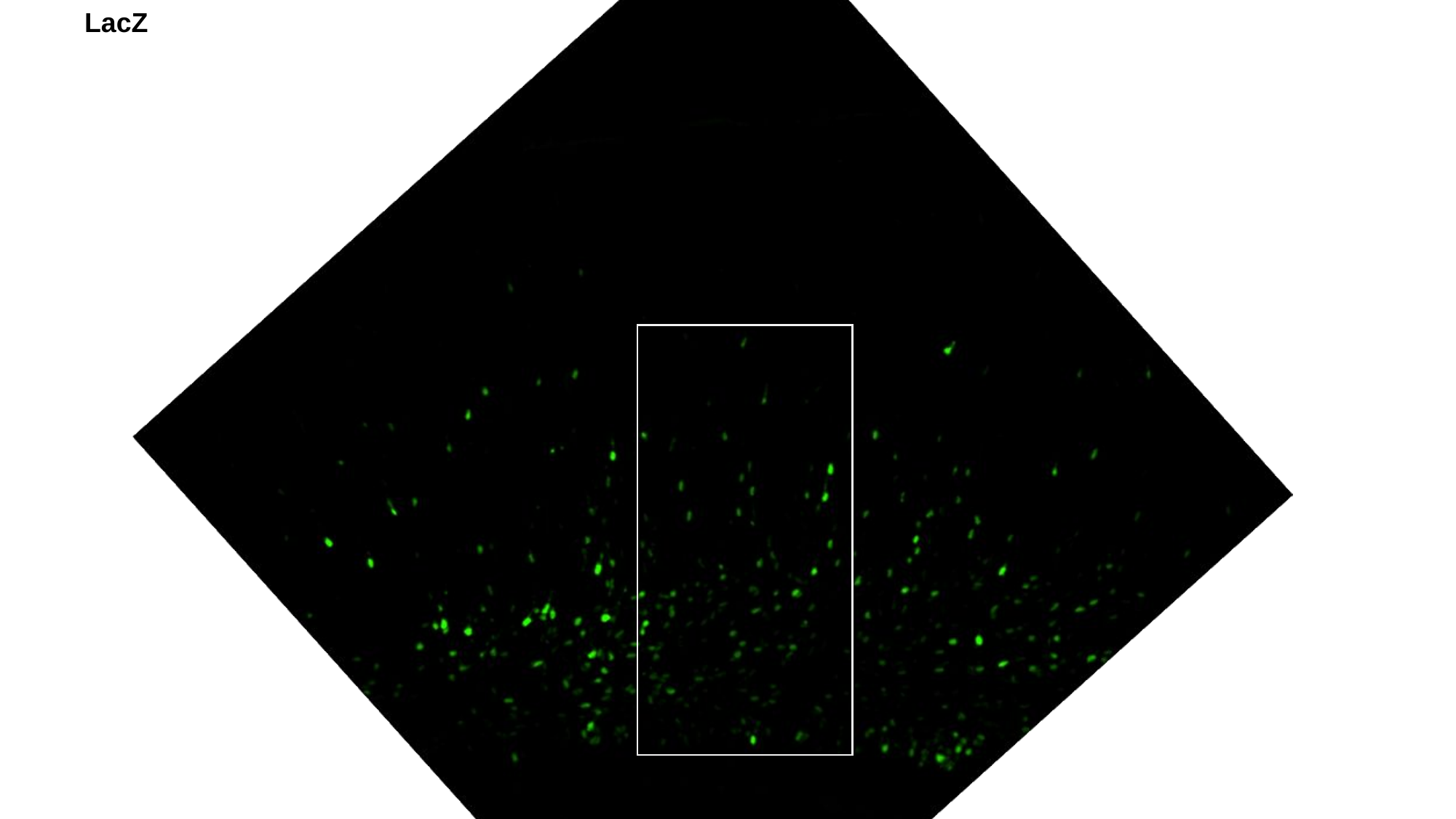

LacZ

## Slide 3
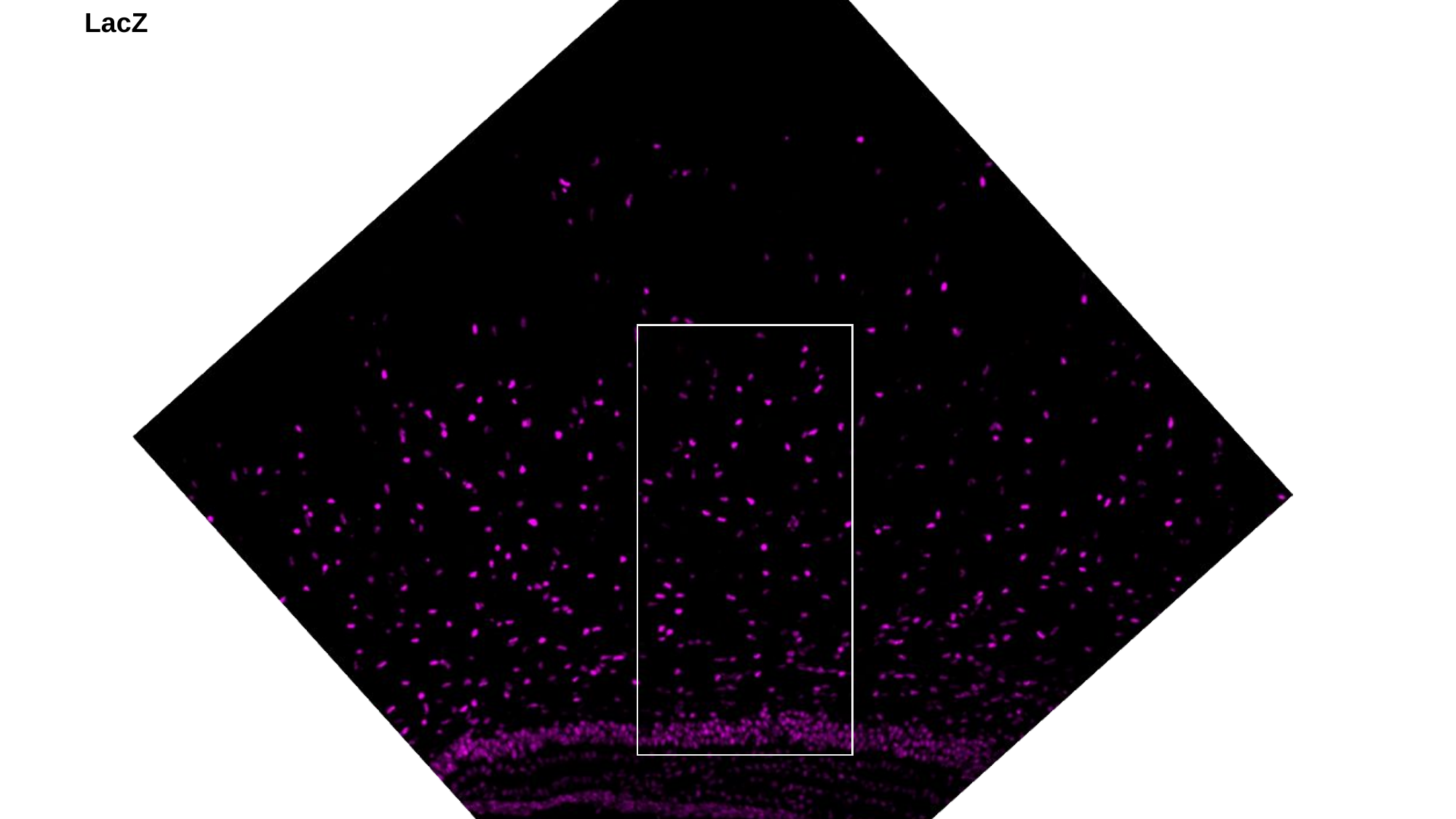

LacZ

## Slide 4
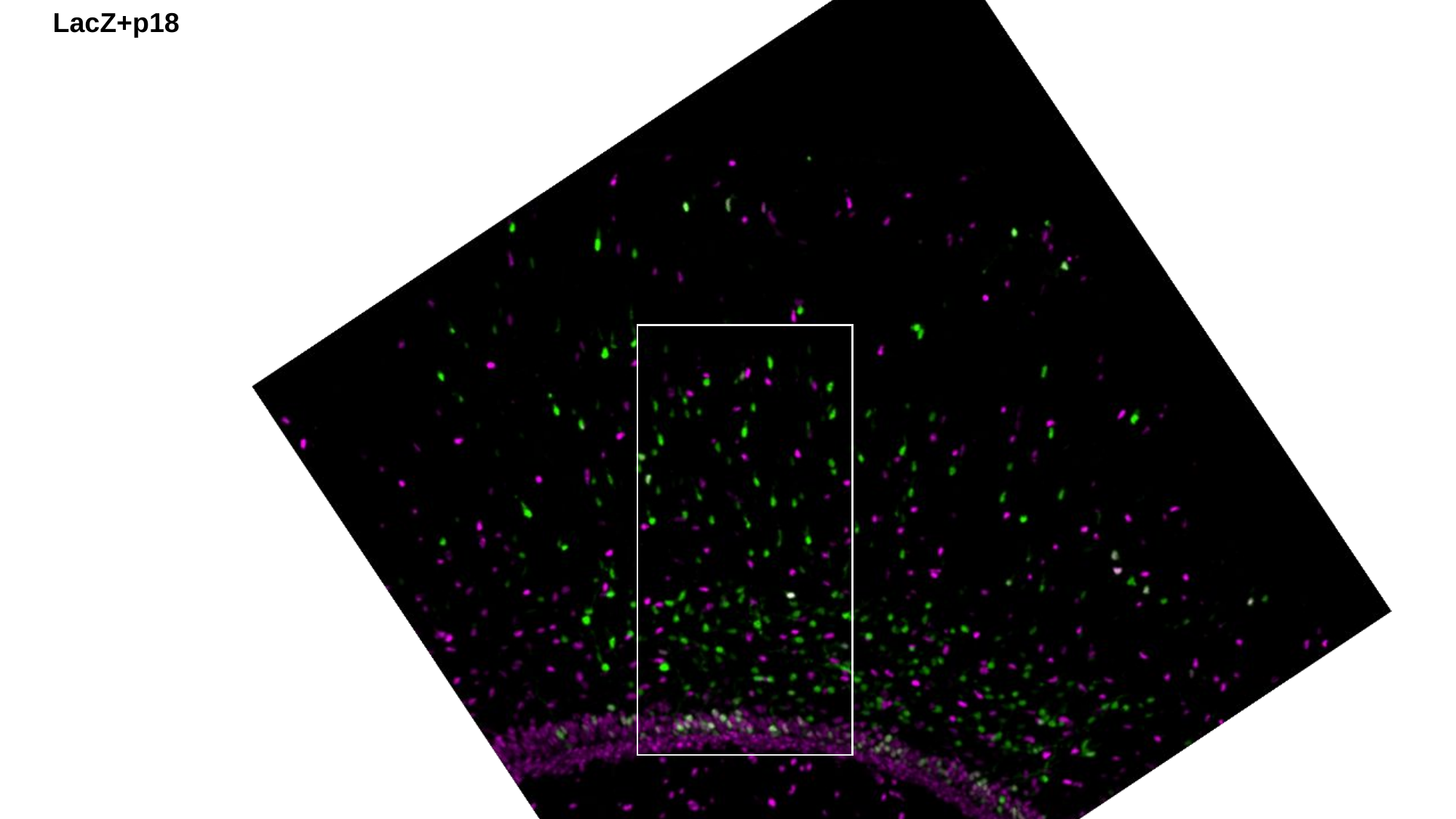

LacZ+p18

## Slide 5
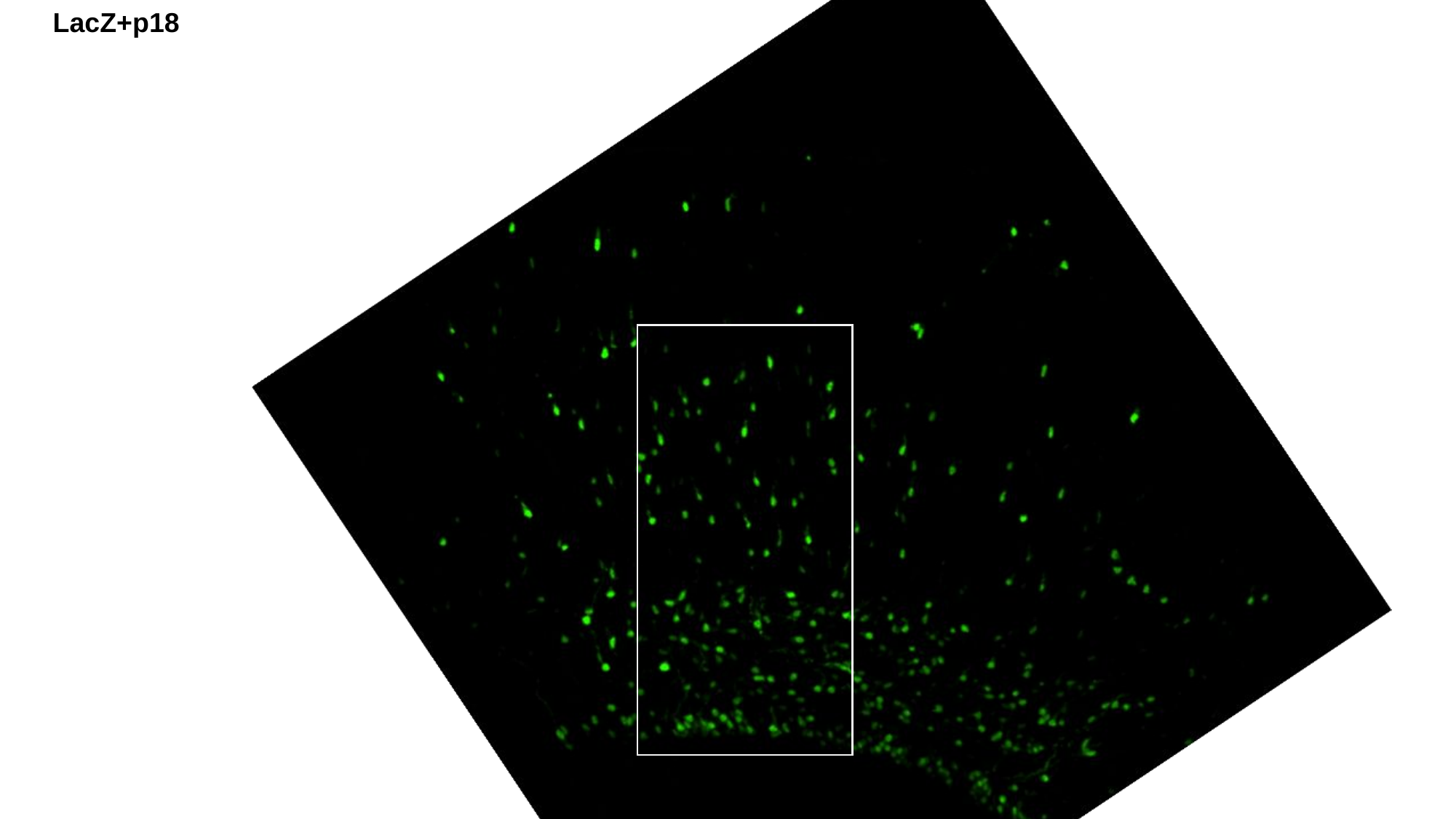

LacZ+p18

## Slide 6
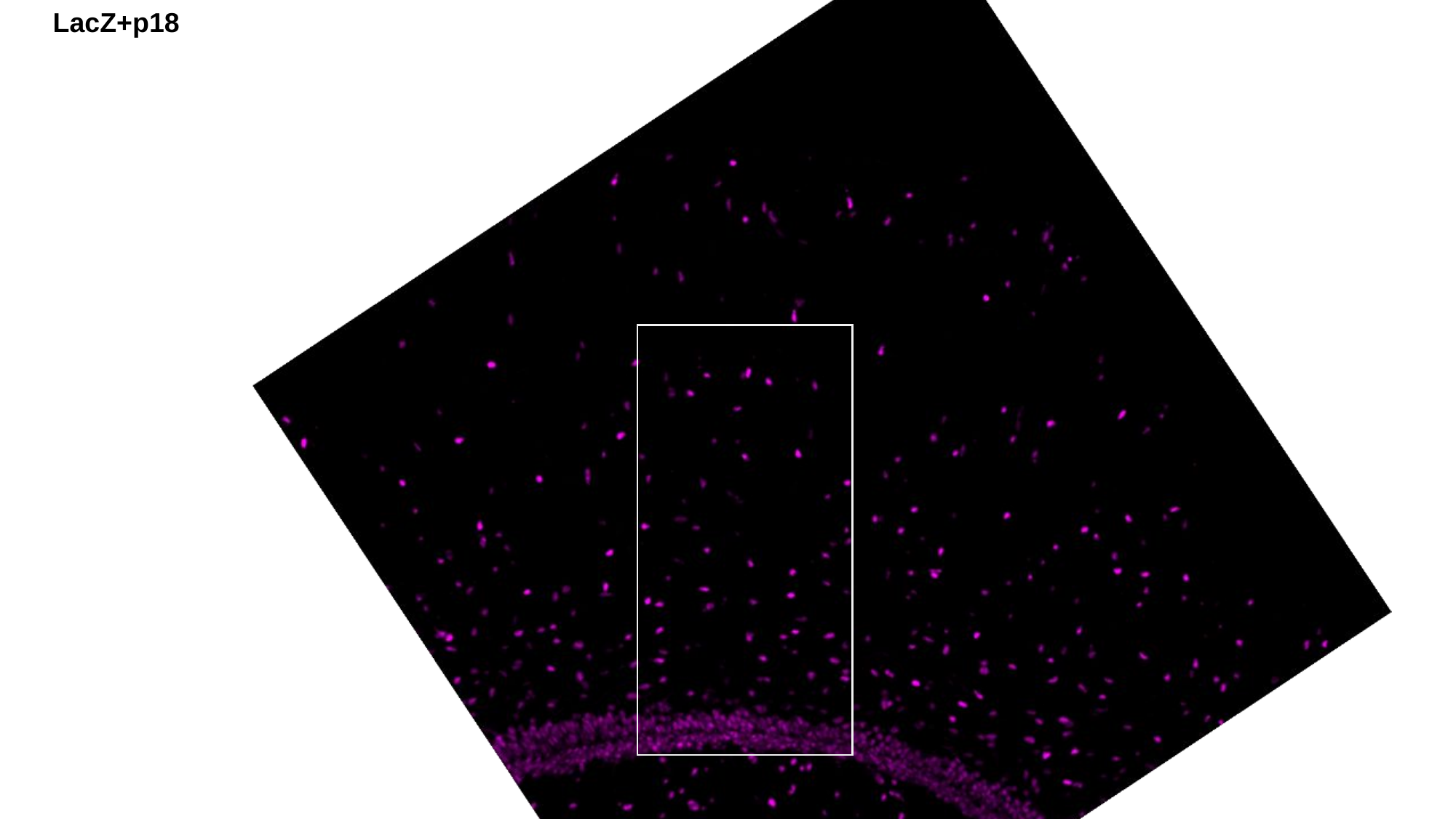

LacZ+p18

## Slide 7
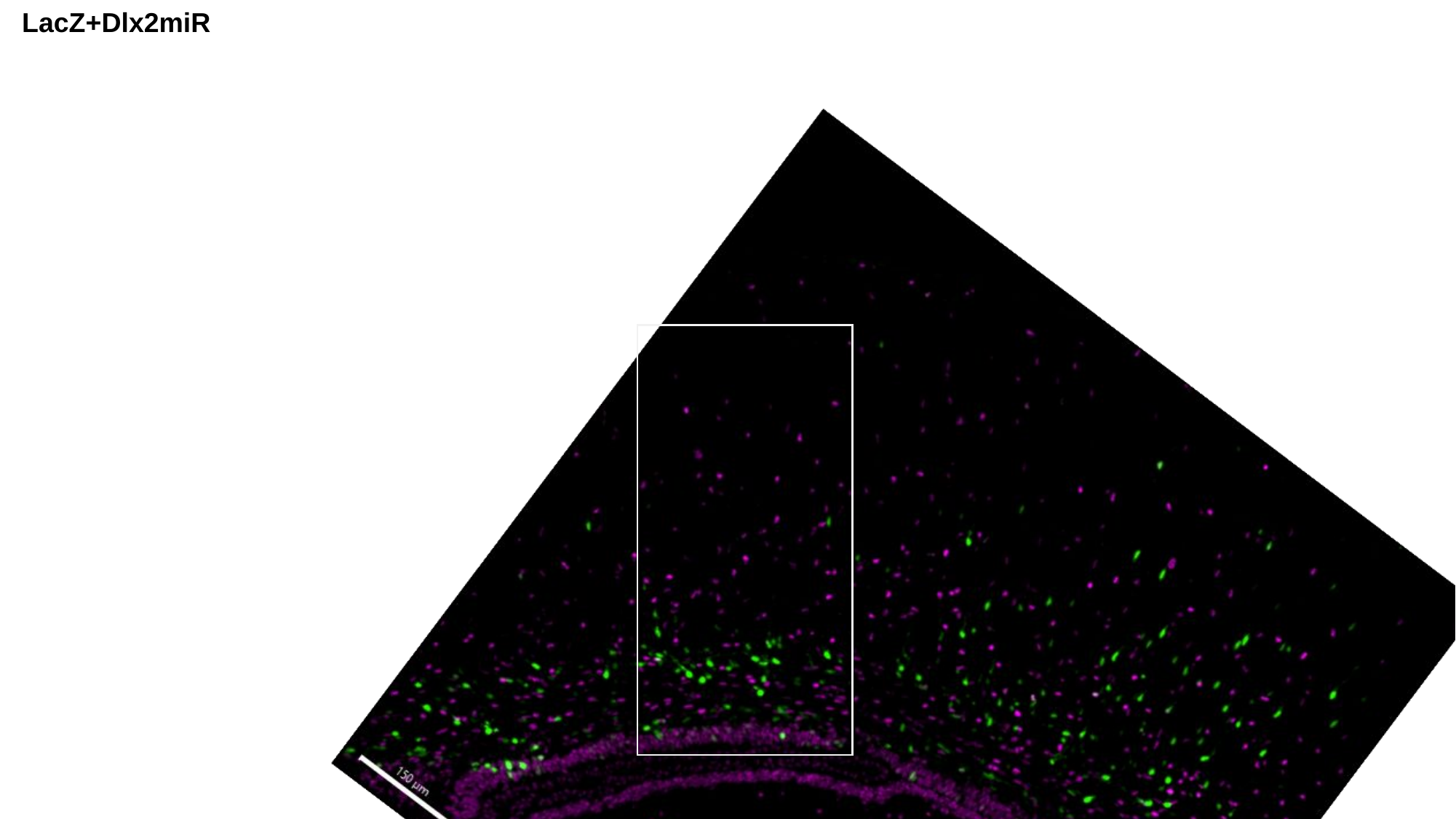

LacZ+Dlx2miR

## Slide 8
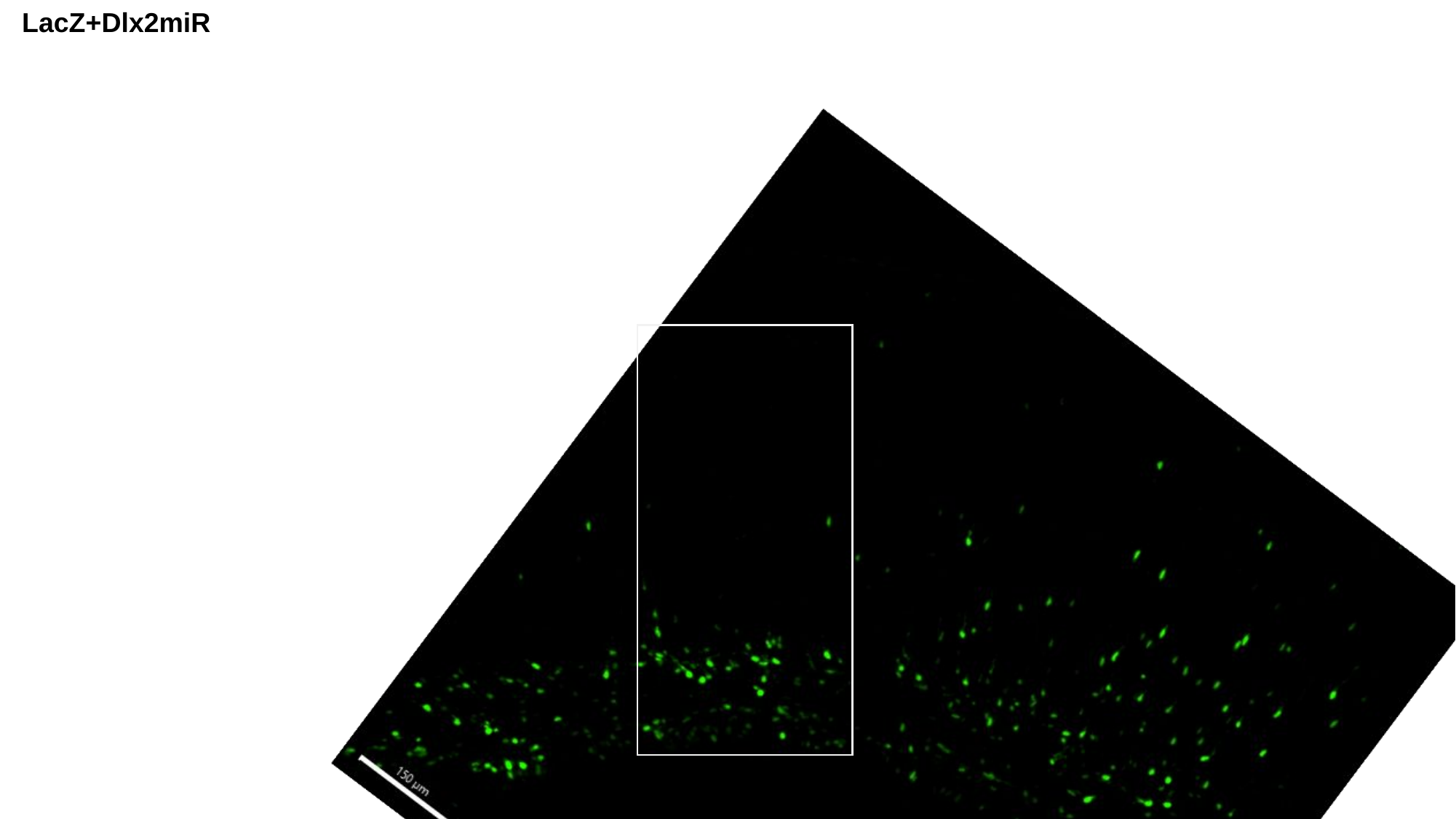

LacZ+Dlx2miR

## Slide 9
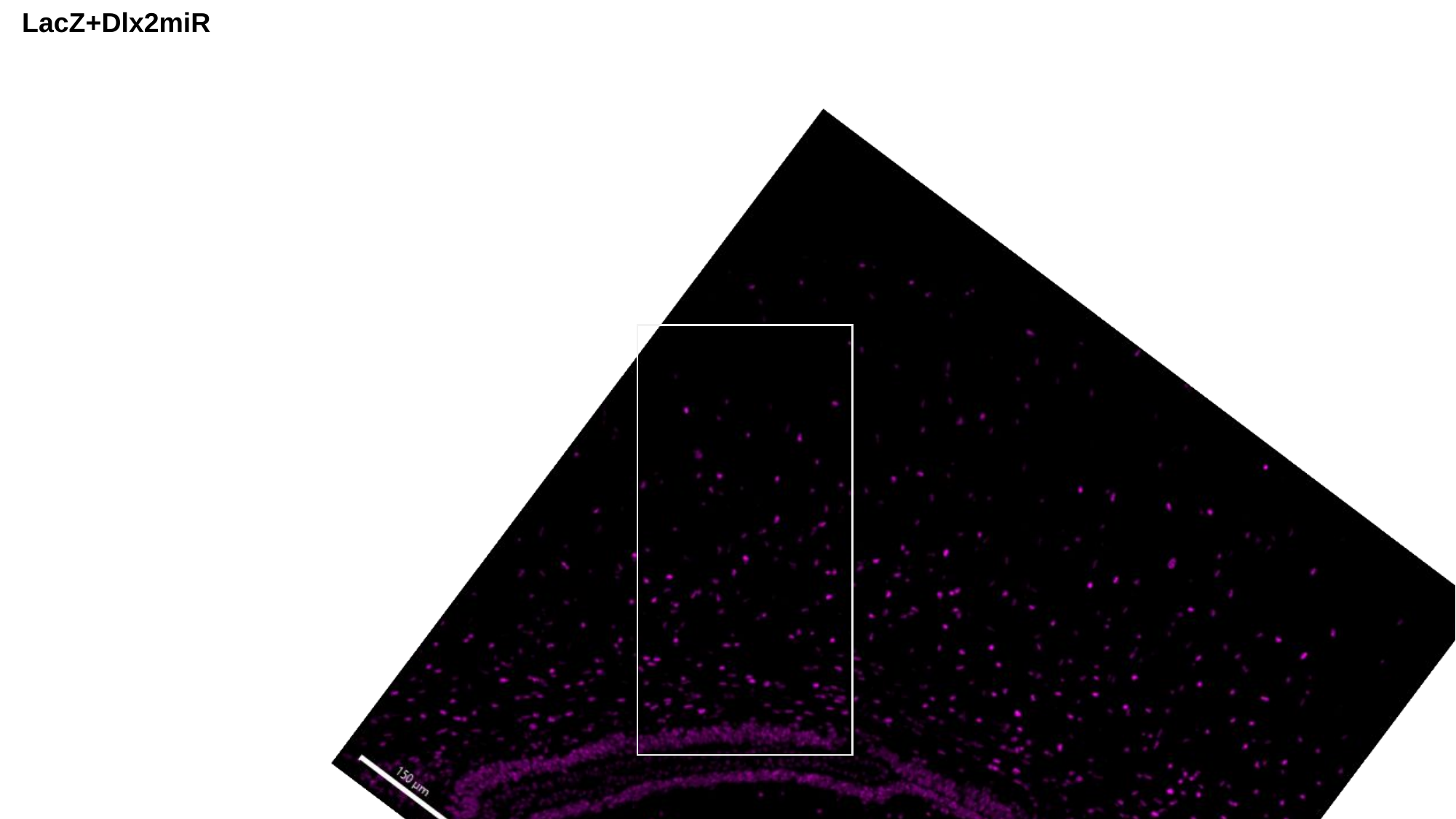

LacZ+Dlx2miR

## Slide 10
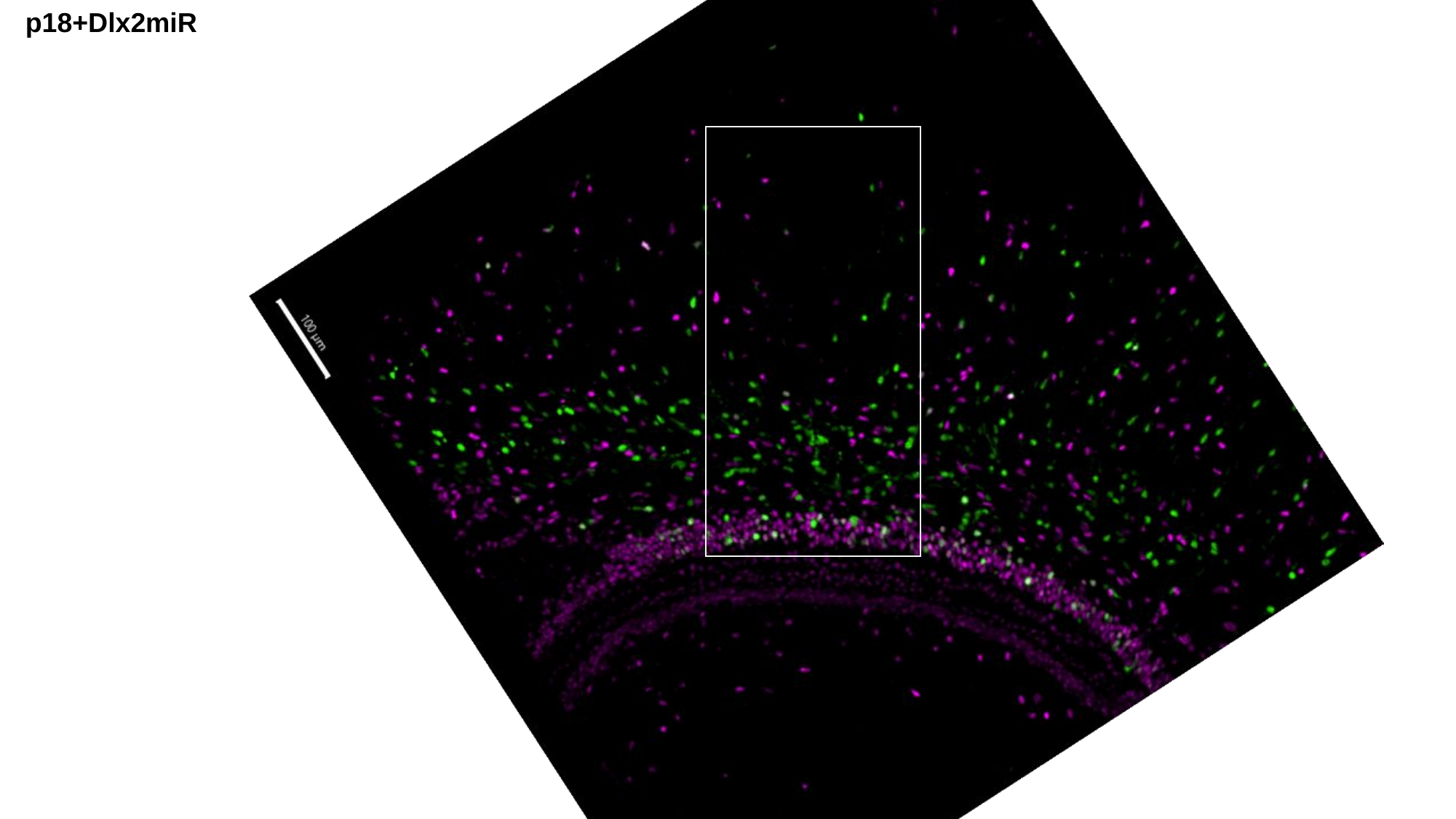

p18+Dlx2miR

## Slide 11
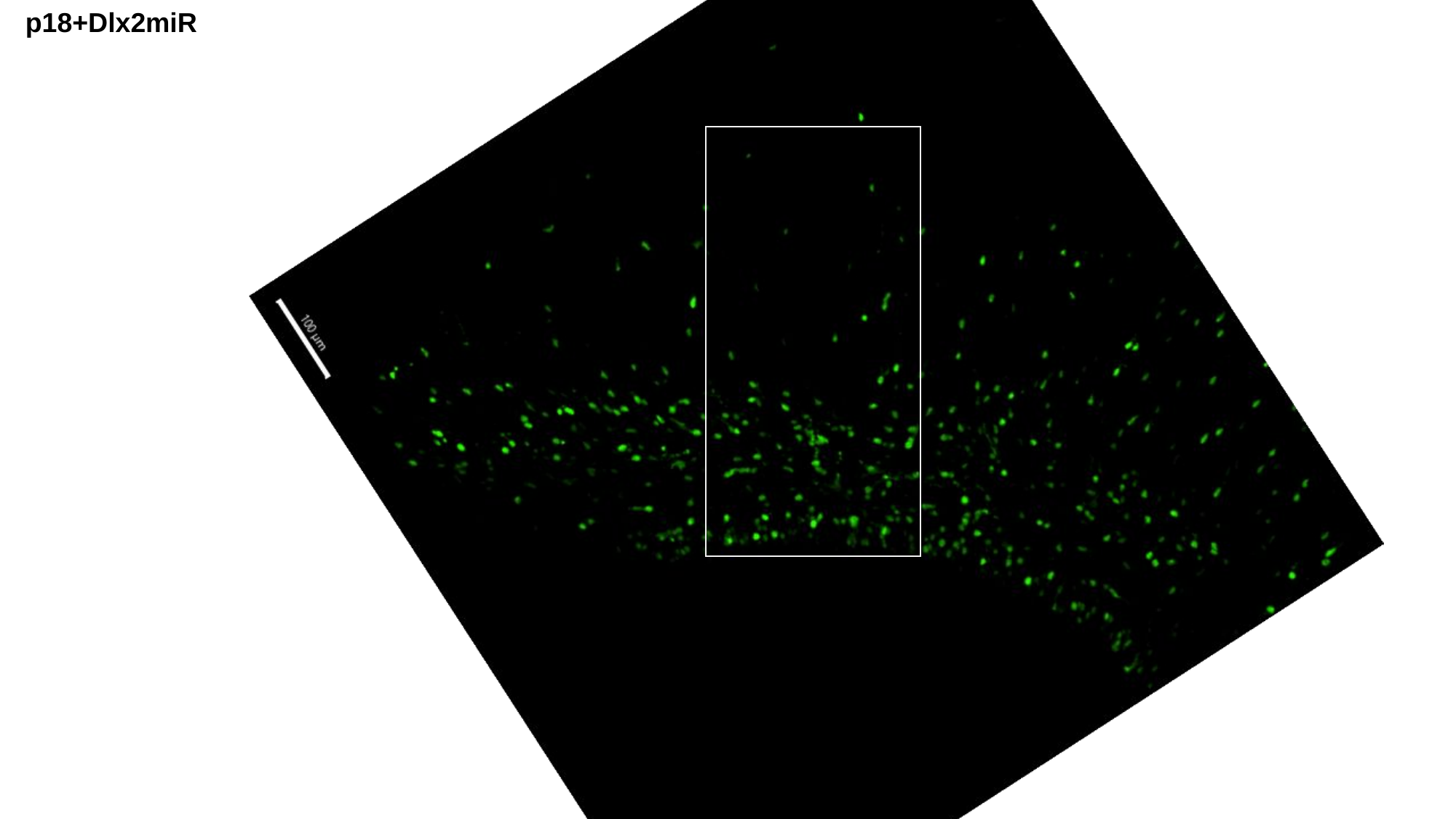

p18+Dlx2miR

## Slide 12
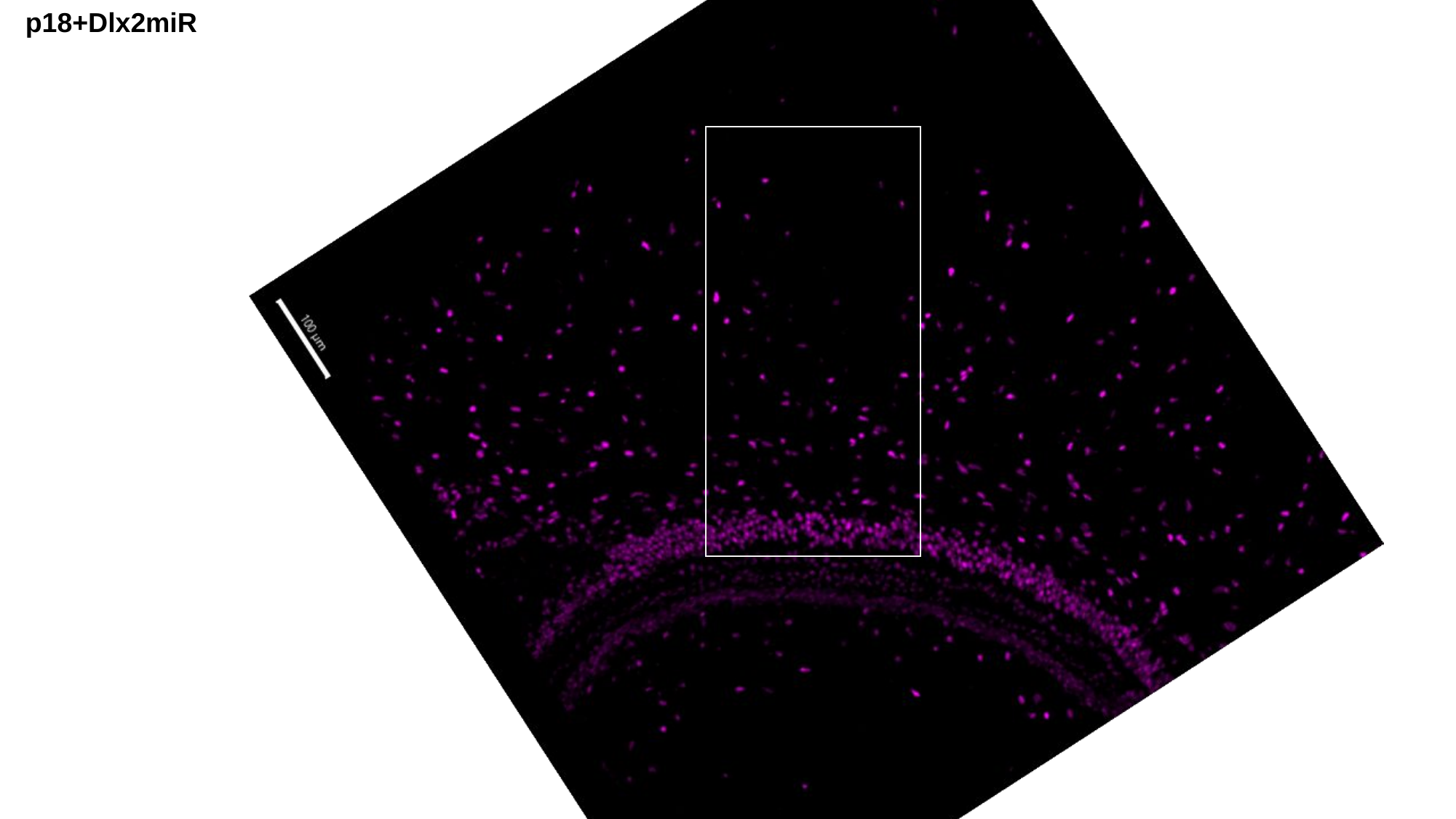

p18+Dlx2miR
