# Supplementary material for: Cyclin-dependent kinase inhibitor p18 regulates lineage transitions of excitatory neurons, astrocytes, and interneurons in the mouse cortex
Source: EMBO J. 2024 Dec 12;44(2):382–412. doi: 10.1038/s44318-024-00325-9 (PMC11730326; doi:10.1038/s44318-024-00325-9)

## Slide 1
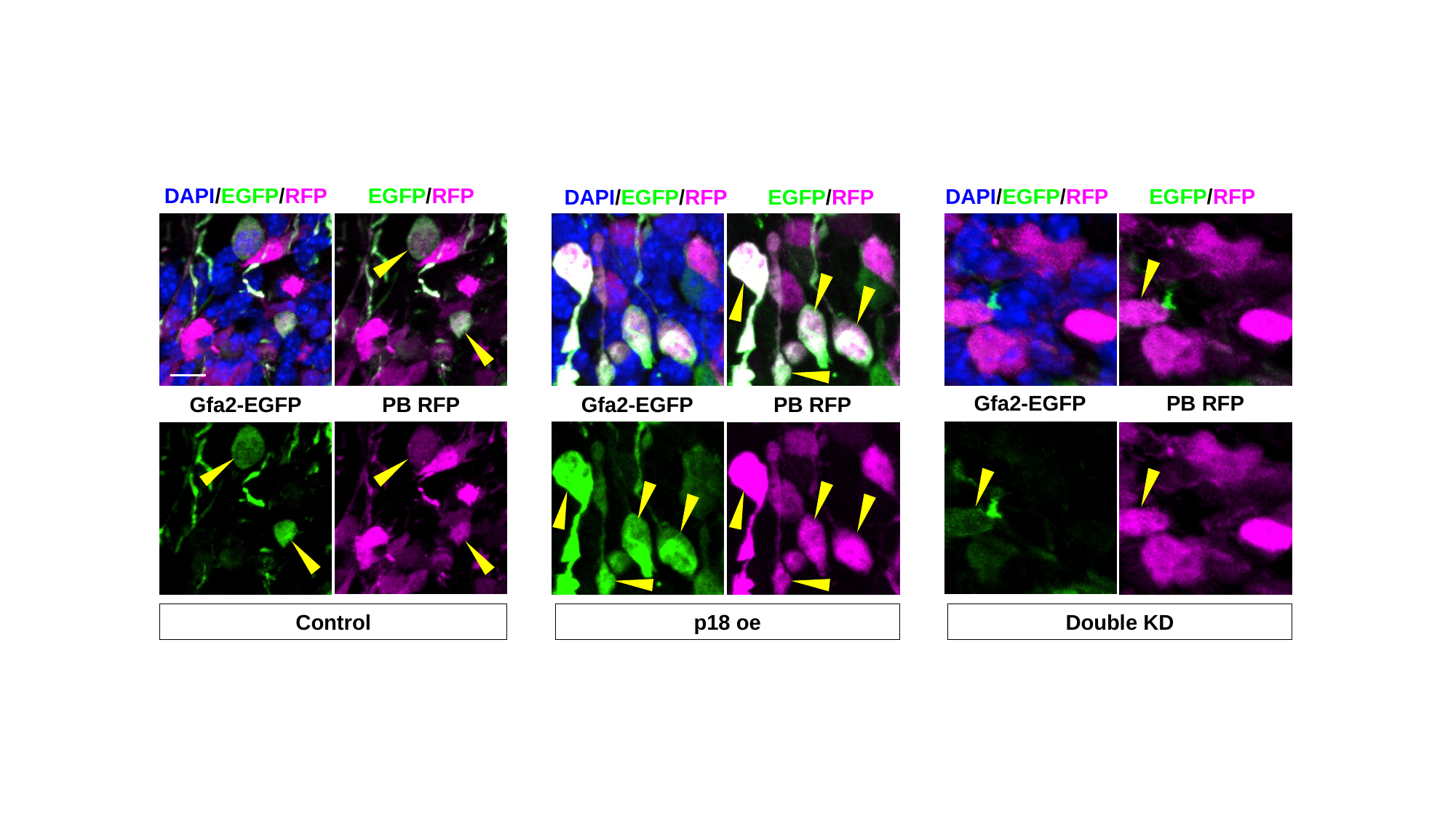

DAPI/EGFP/RFP
EGFP/RFP
DAPI/EGFP/RFP
EGFP/RFP
DAPI/EGFP/RFP
EGFP/RFP
Gfa2-EGFP
PB RFP
Gfa2-EGFP
PB RFP
Gfa2-EGFP
PB RFP
p18 oe
Double KD
Control

## Slide 2
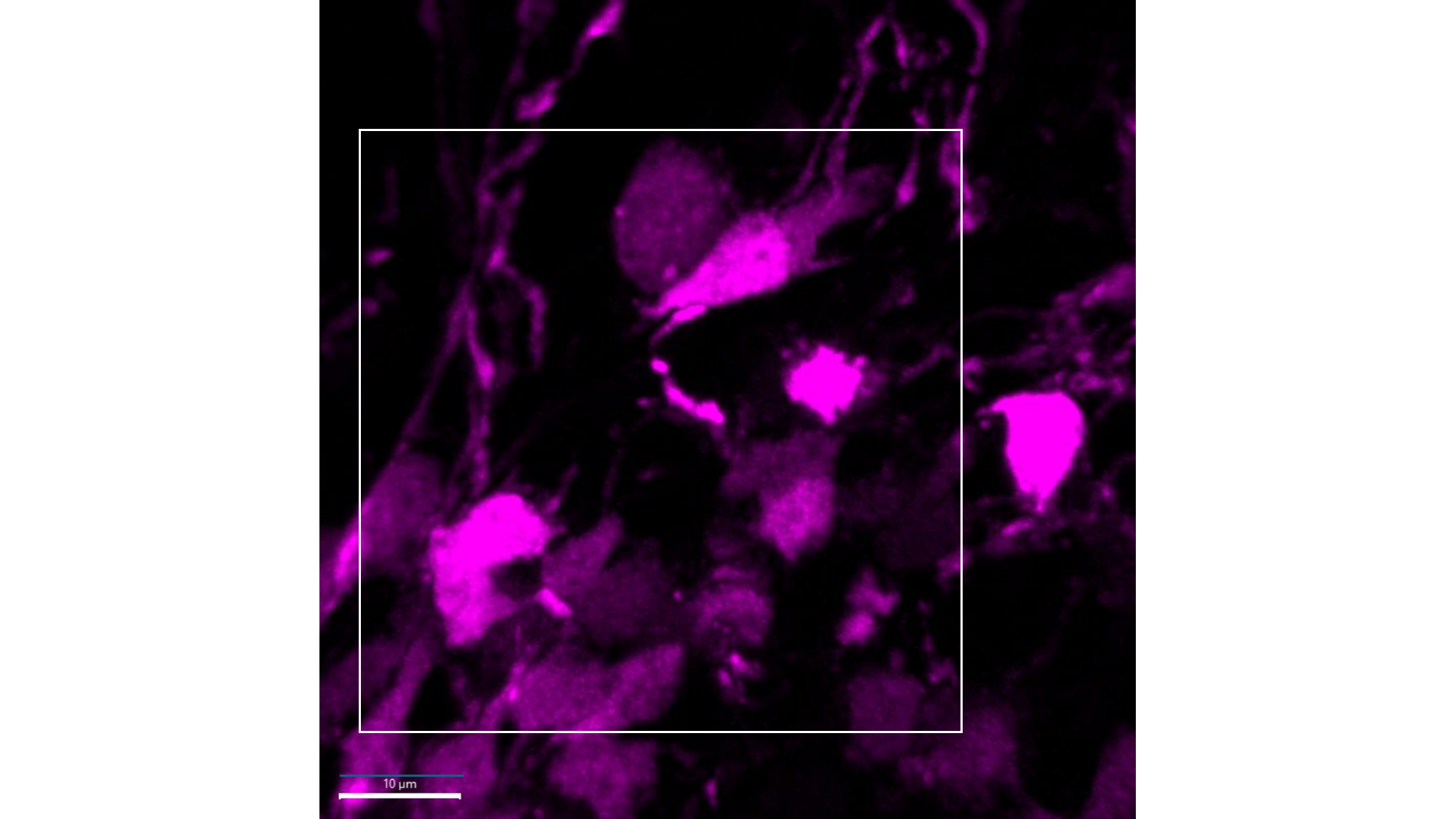

## Slide 3
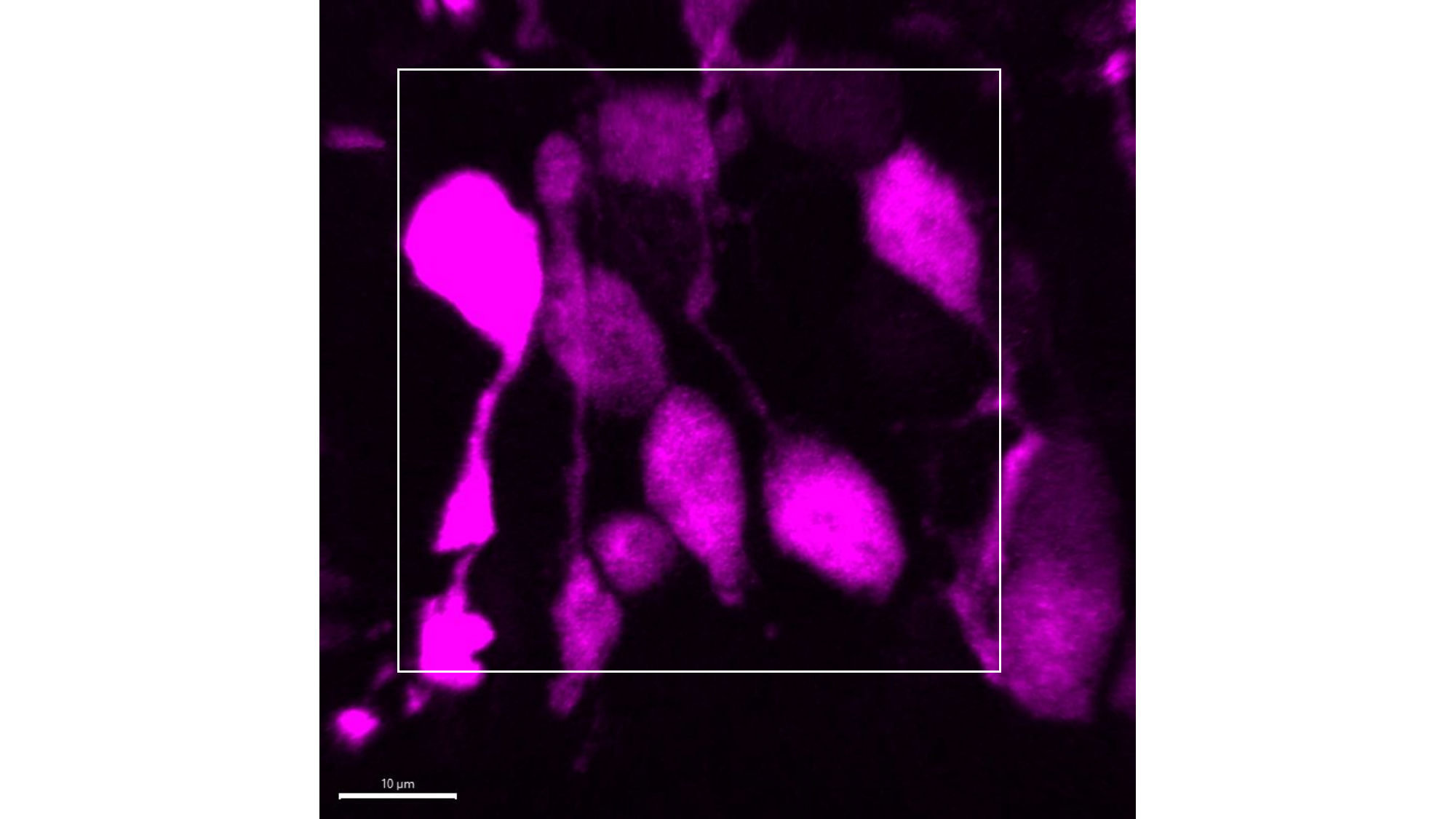

## Slide 4
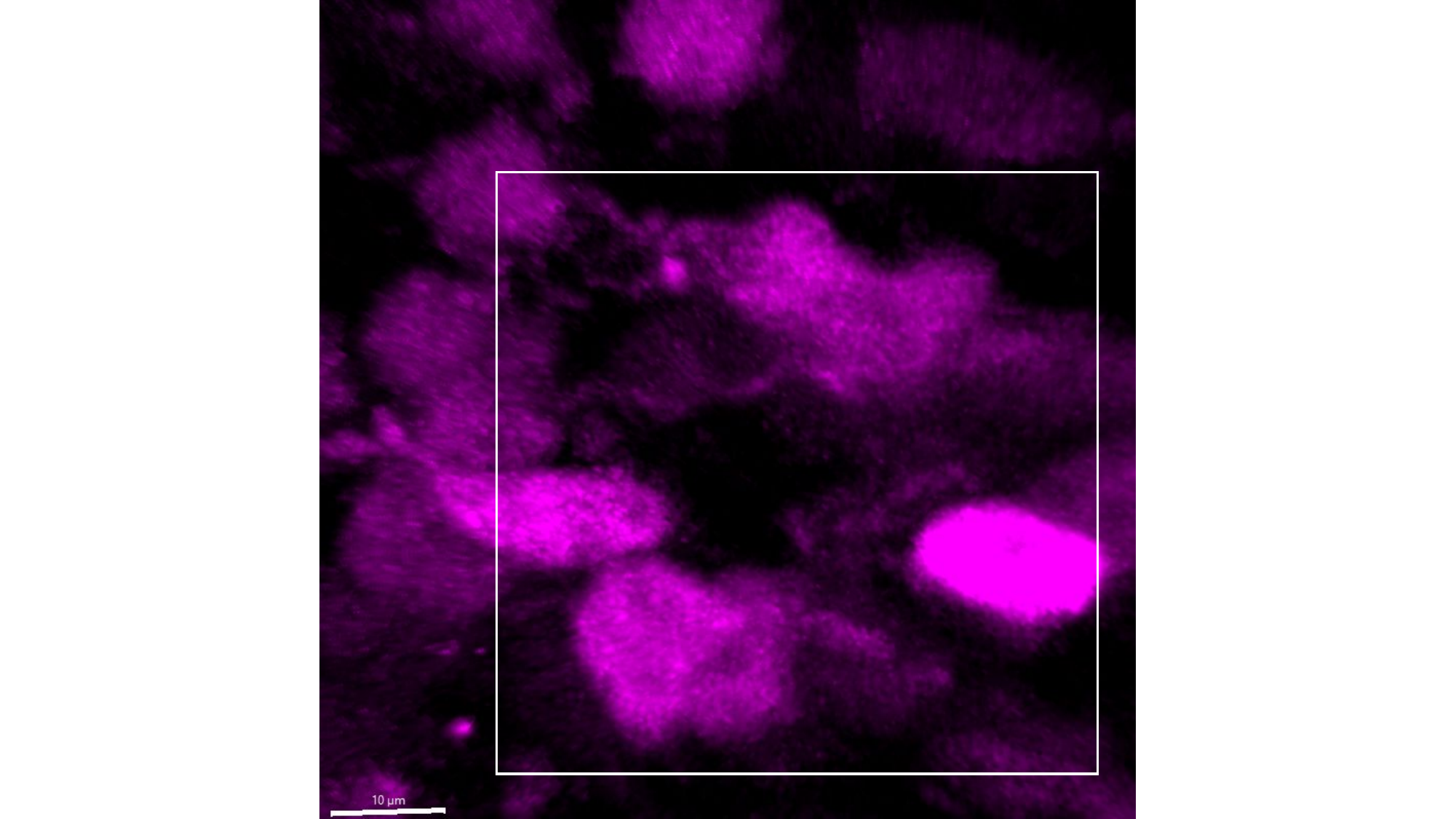

Supplement: Supplementary file 11 — Source data Fig. 9 [file 44318_2024_325_MOESM11_ESM.zip › 9A/9A_b-d.pptx]
