# Supplementary material for: Cyclin-dependent kinase inhibitor p18 regulates lineage transitions of excitatory neurons, astrocytes, and interneurons in the mouse cortex
Source: EMBO J. 2024 Dec 12;44(2):382–412. doi: 10.1038/s44318-024-00325-9 (PMC11730326; doi:10.1038/s44318-024-00325-9)

## Slide 1
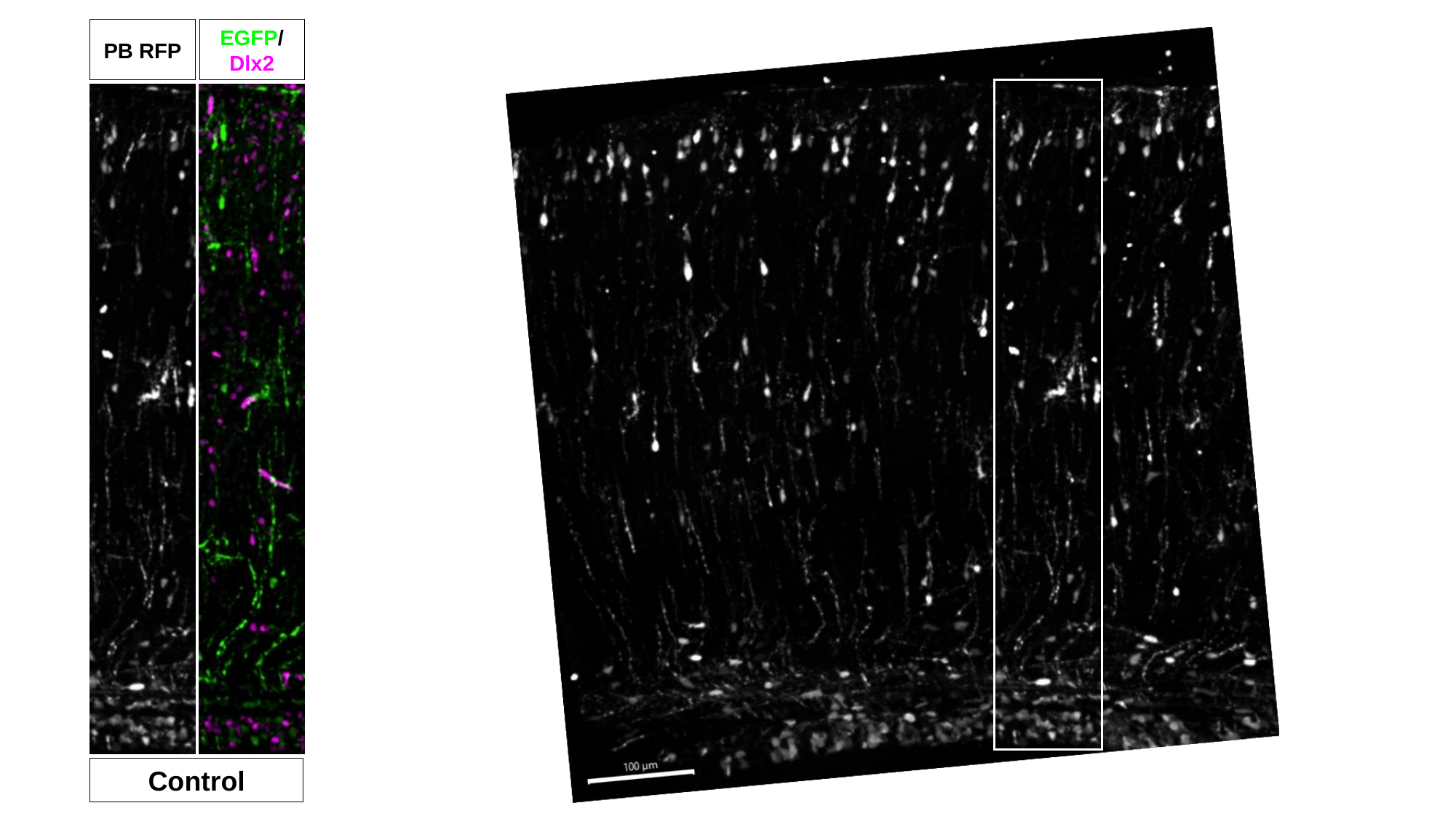

PB RFP
EGFP/
Dlx2
Control

## Slide 2
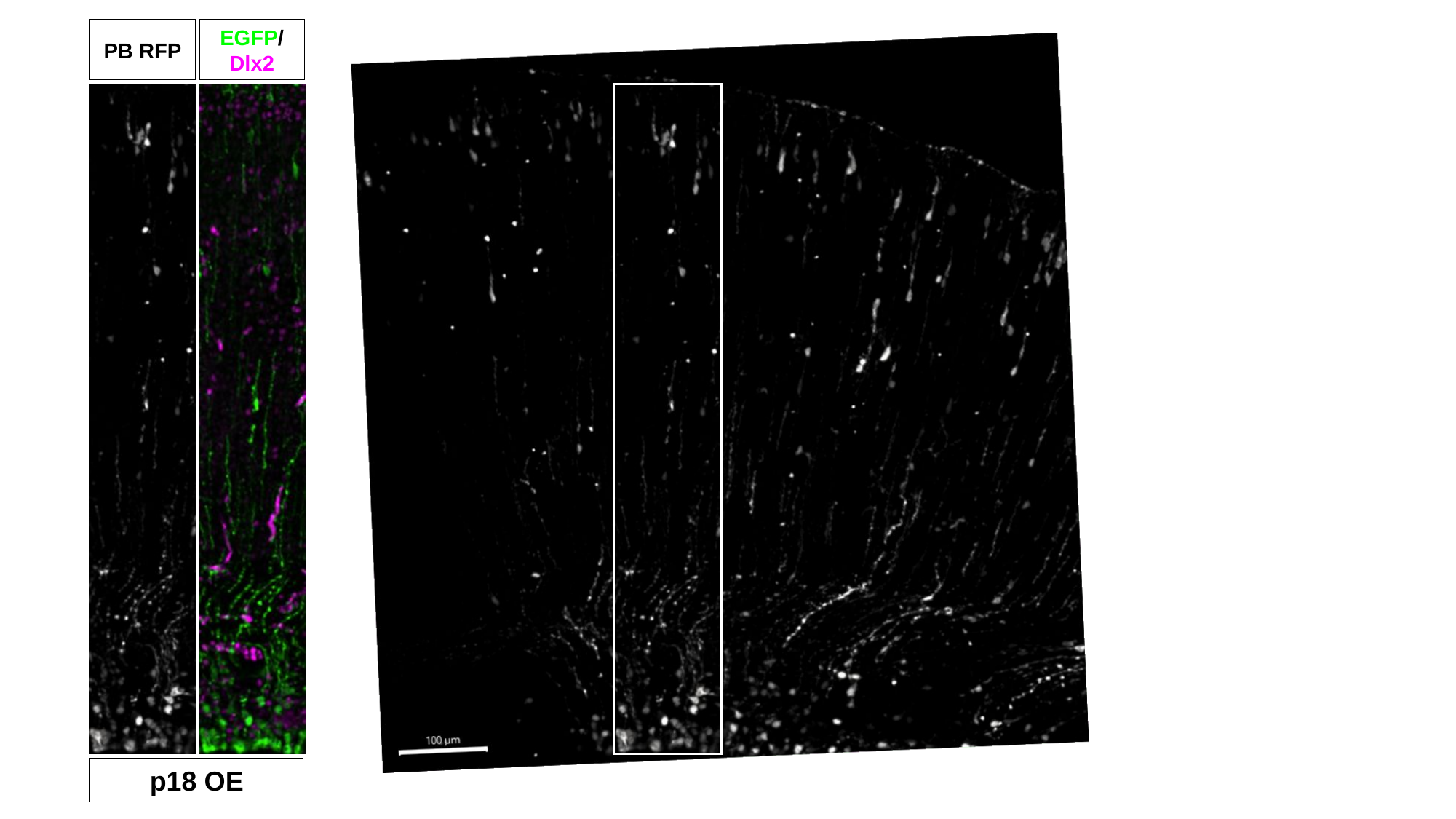

PB RFP
EGFP/
Dlx2
p18 OE

## Slide 3
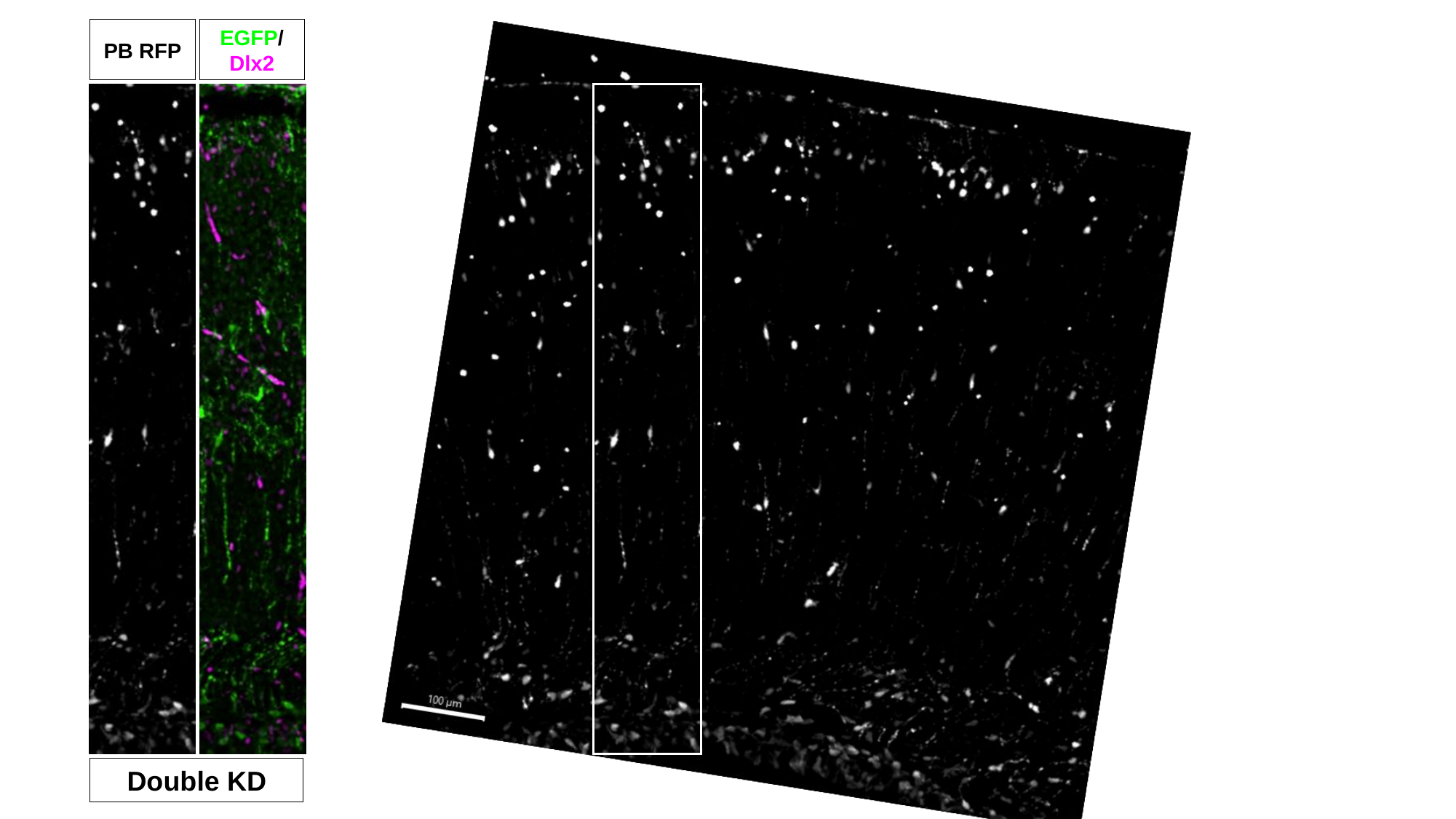

PB RFP
EGFP/
Dlx2
Double KD

## Slide 4
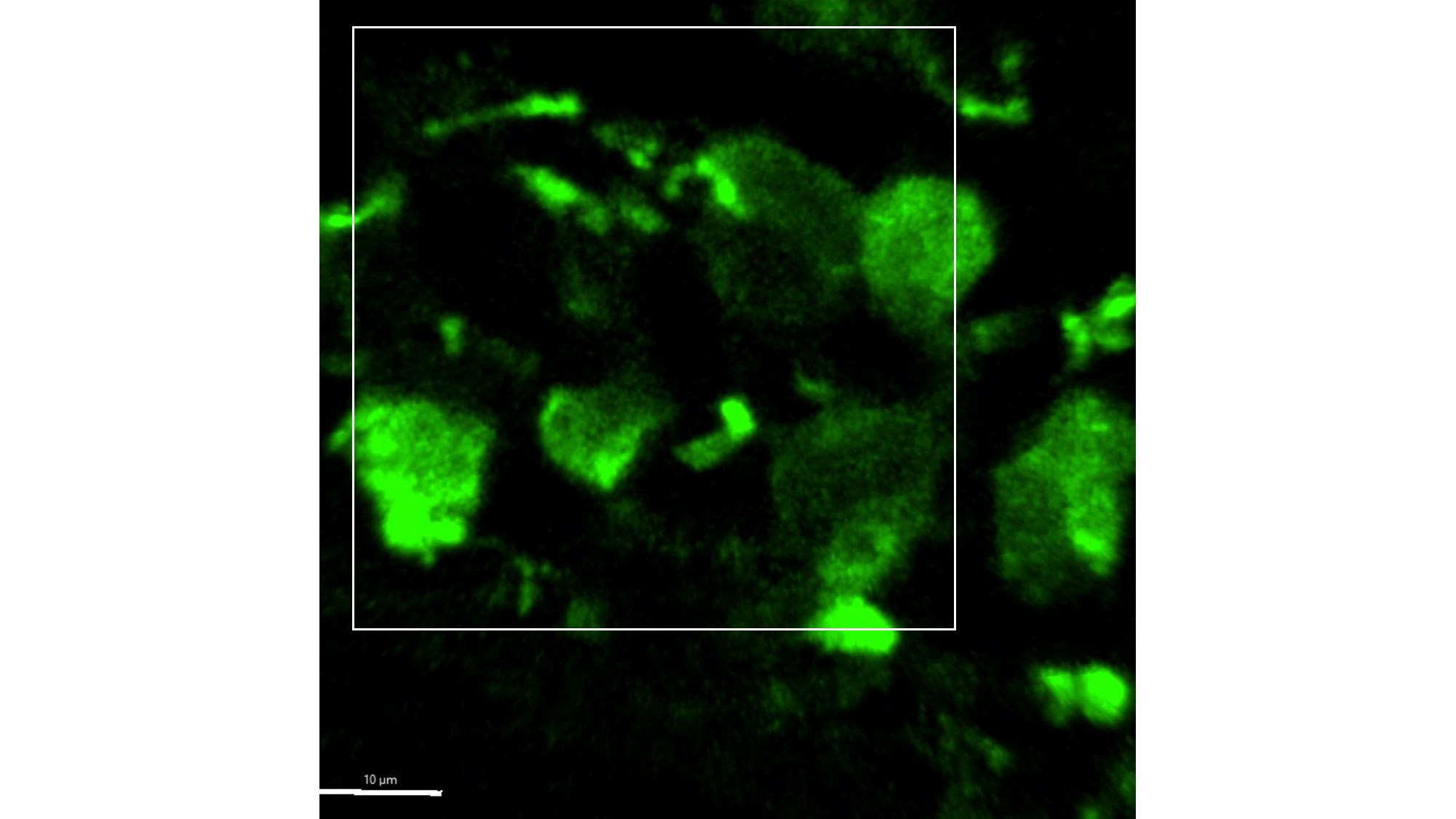

## Slide 5
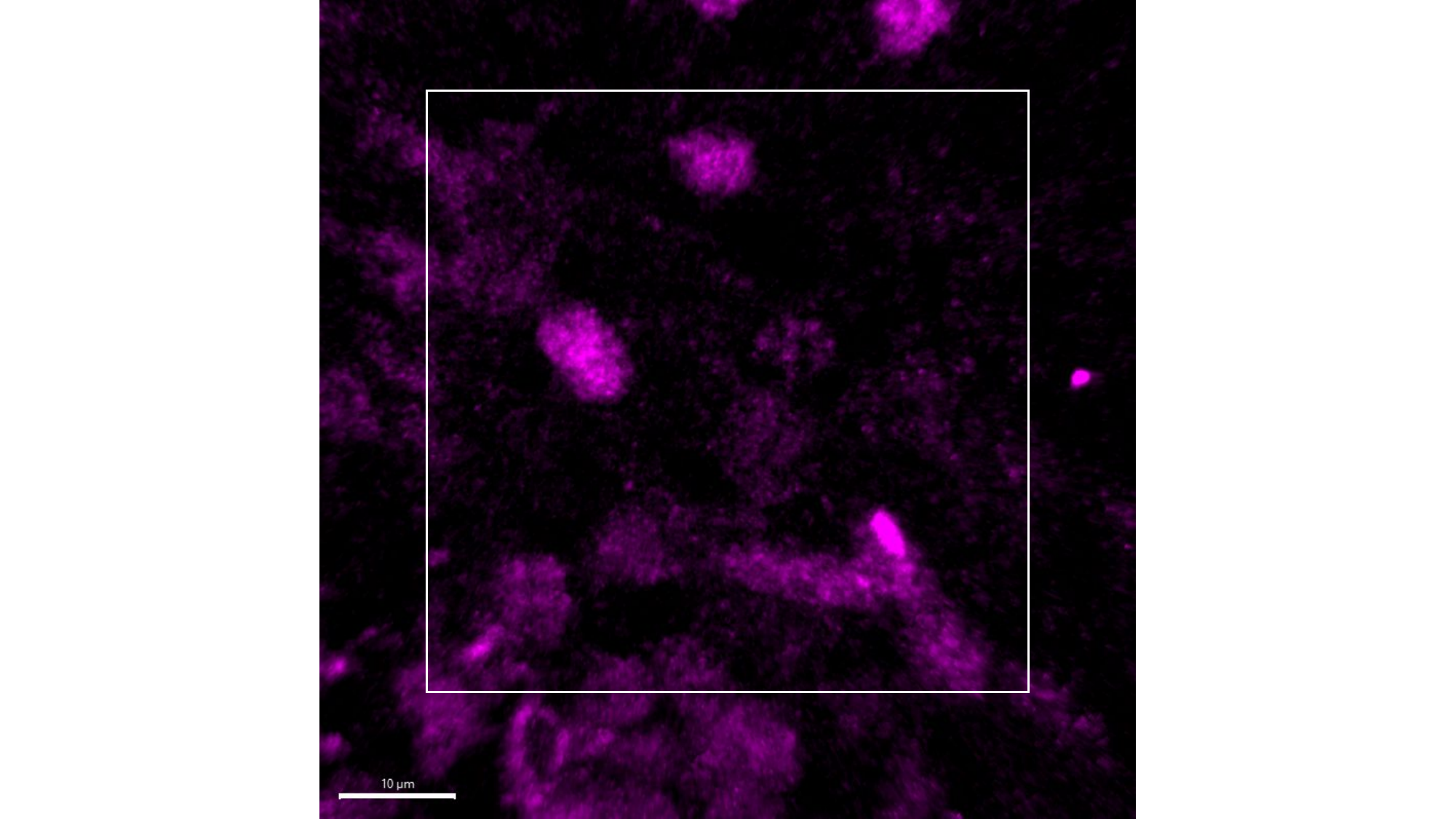

## Slide 6
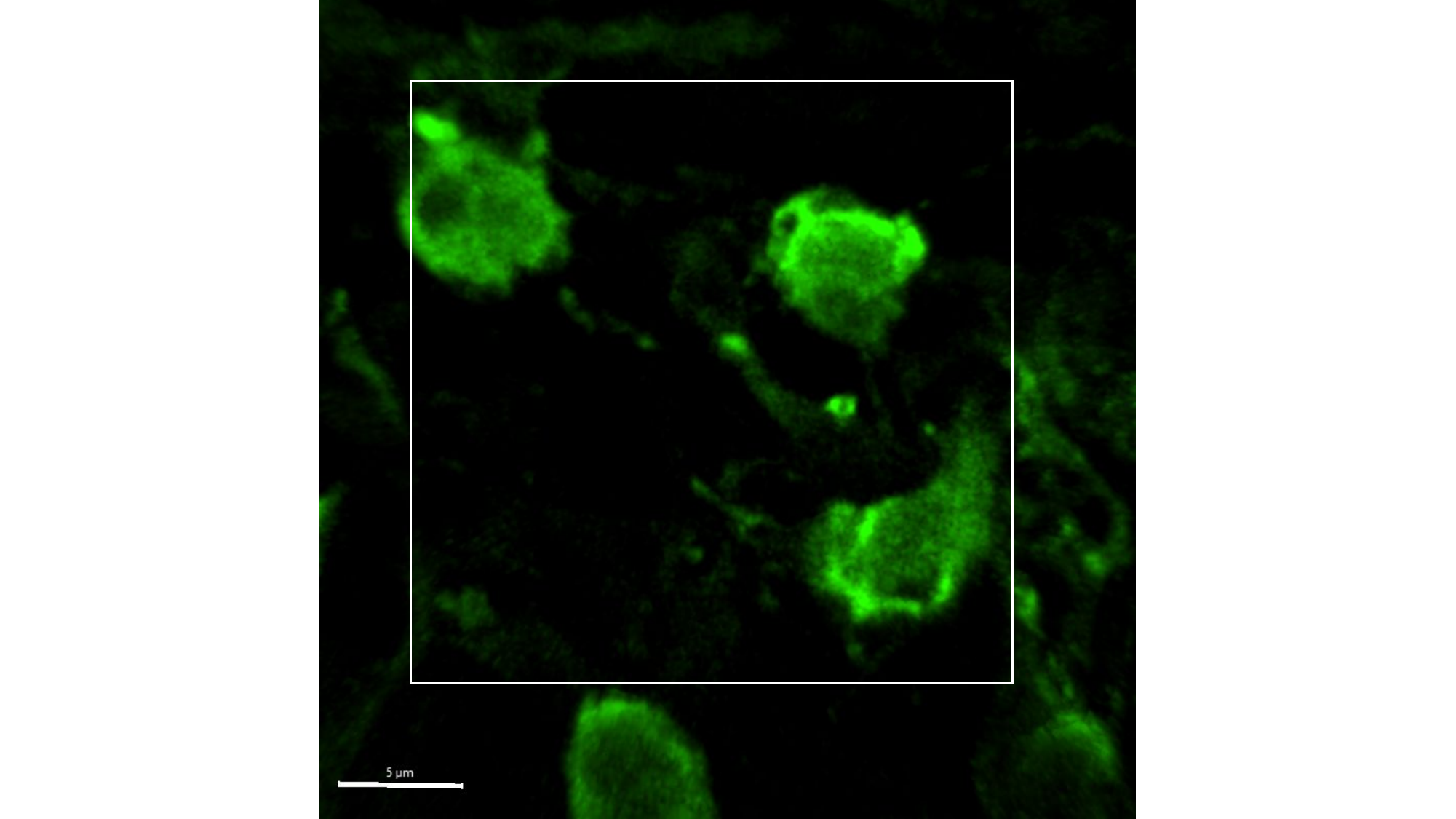

Supplement: Supplementary file 11 — Source data Fig. 9 [file 44318_2024_325_MOESM11_ESM.zip › 9D/9D_a-b.pptx]
