# Supplementary material for: Cyclin-dependent kinase inhibitor p18 regulates lineage transitions of excitatory neurons, astrocytes, and interneurons in the mouse cortex
Source: EMBO J. 2024 Dec 12;44(2):382–412. doi: 10.1038/s44318-024-00325-9 (PMC11730326; doi:10.1038/s44318-024-00325-9)
Supplement: Supplementary file 11 — Source data Fig. 9 [file 44318_2024_325_MOESM11_ESM.zip › 9C.pptx]

## Slide 1
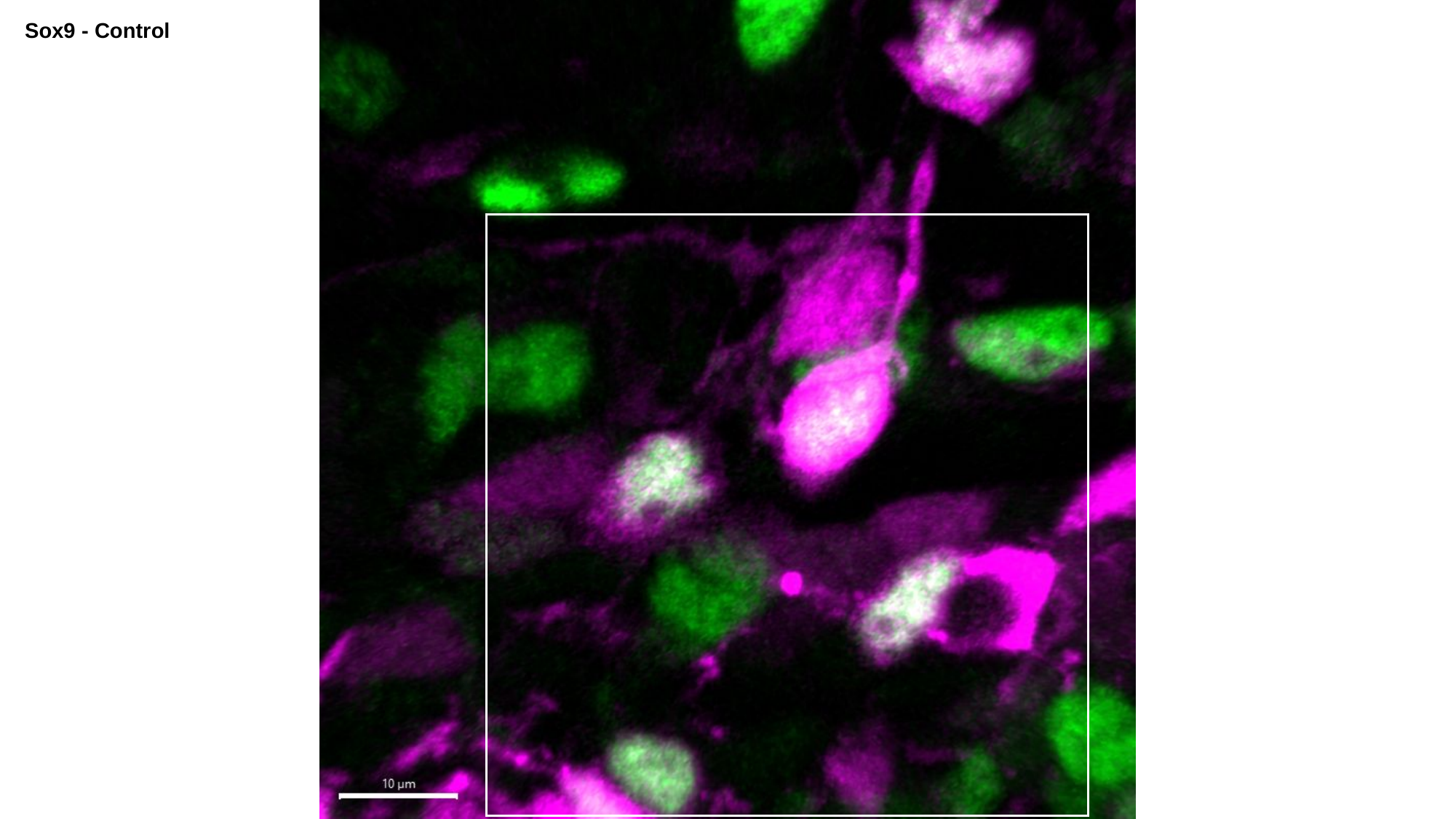

Sox9 - Control

## Slide 2
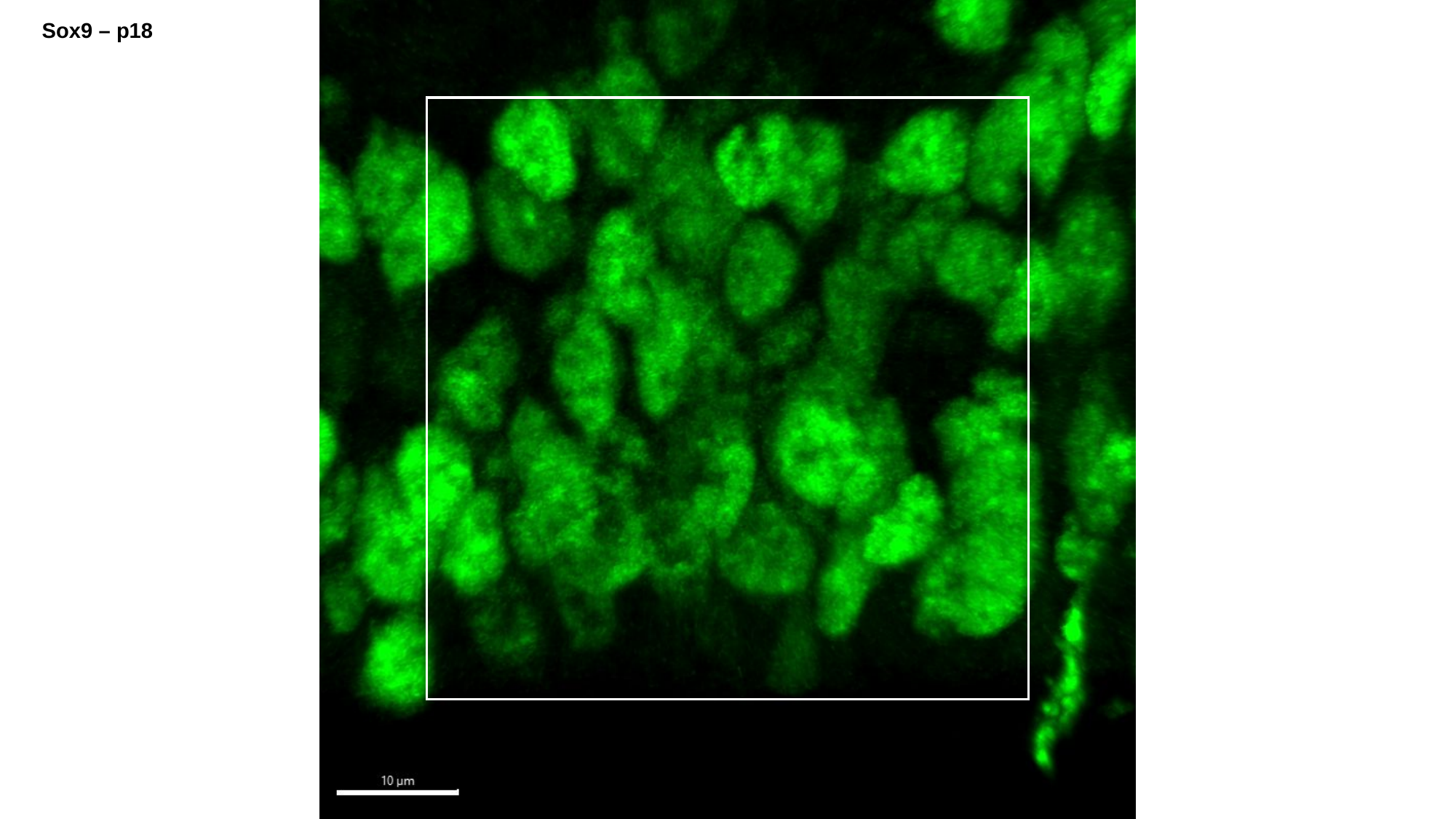

Sox9 – p18

## Slide 3
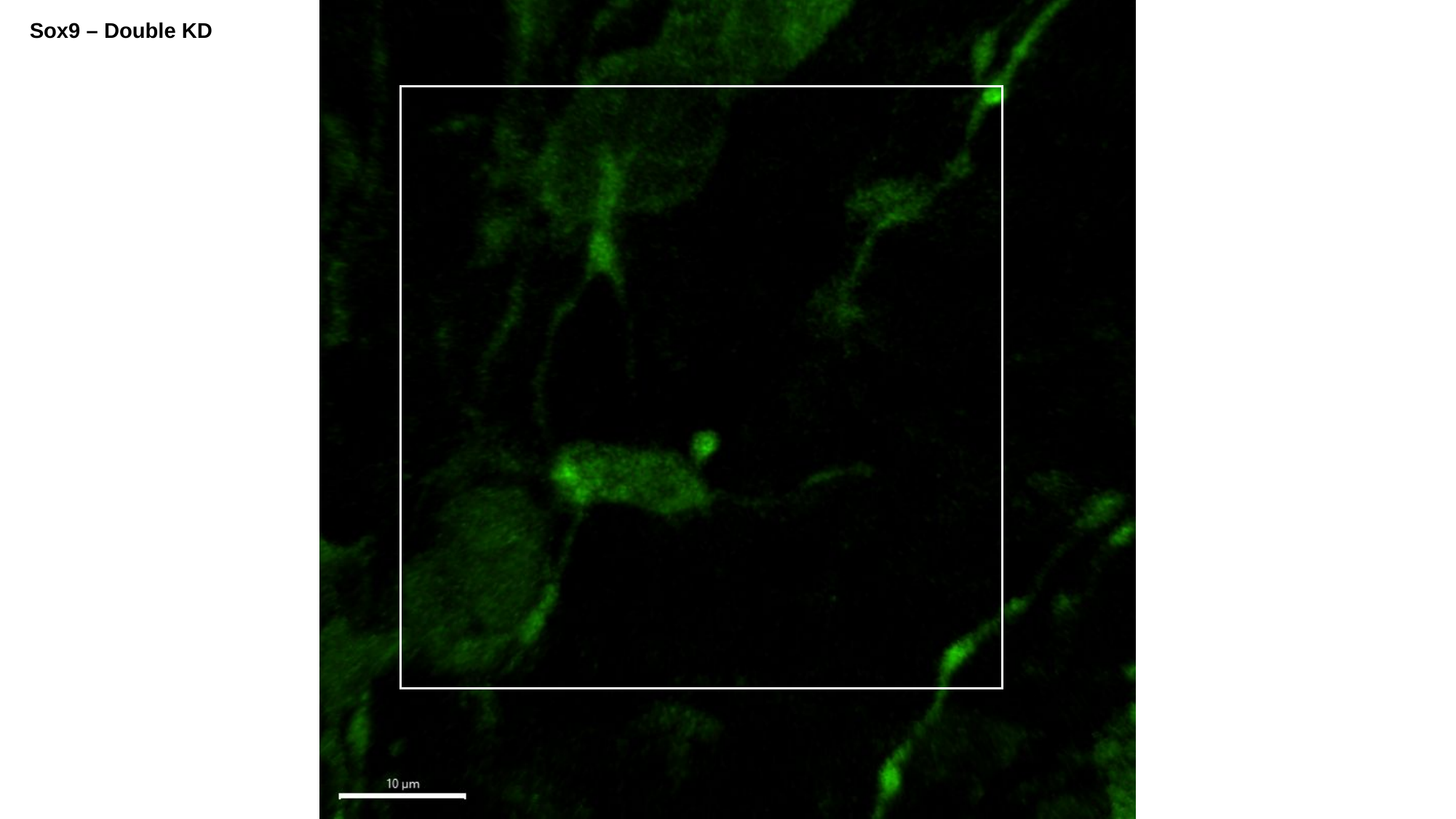

Sox9 – Double KD

## Slide 4
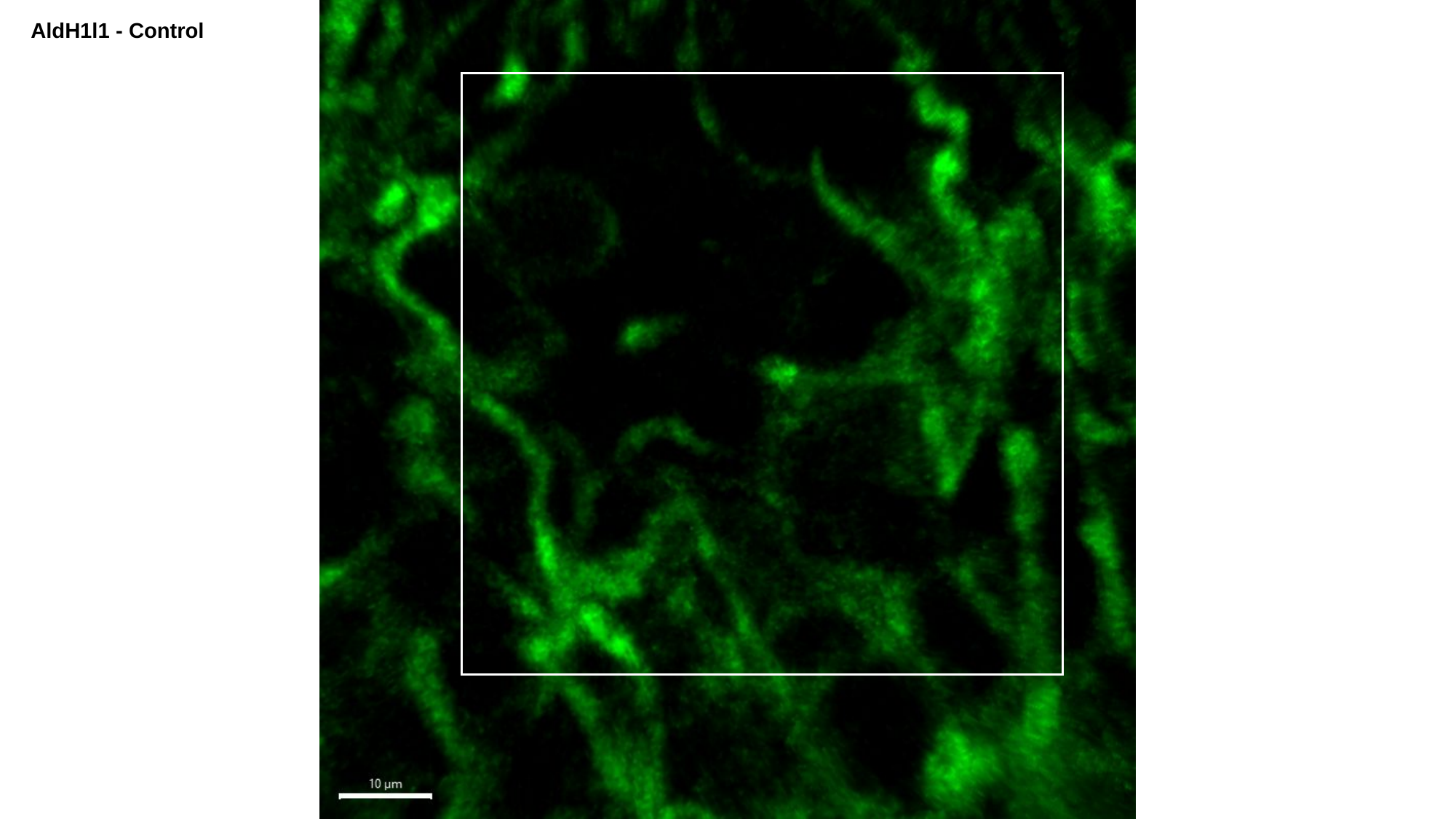

AldH1l1 - Control

## Slide 5
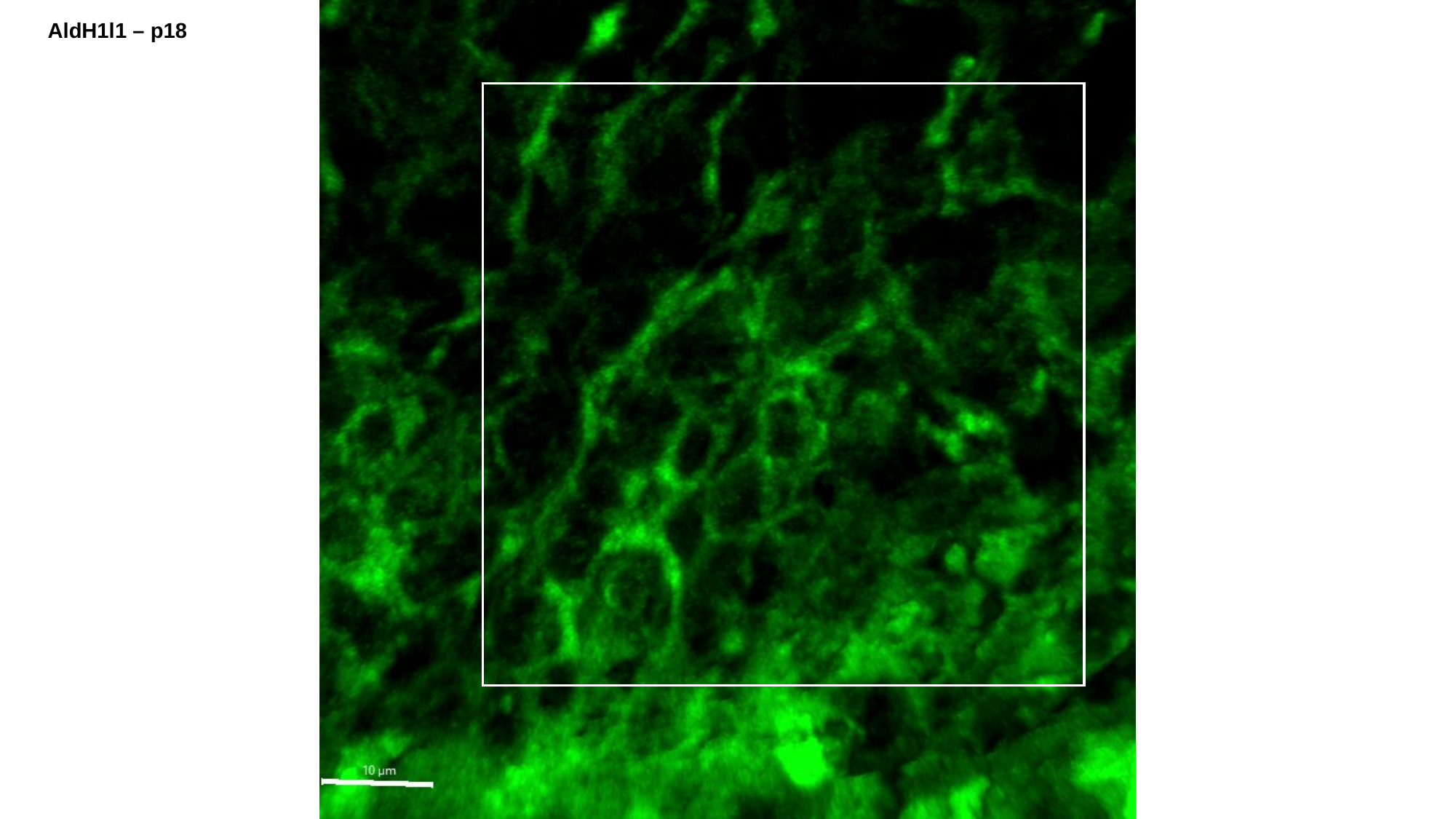

AldH1l1 – p18

## Slide 6
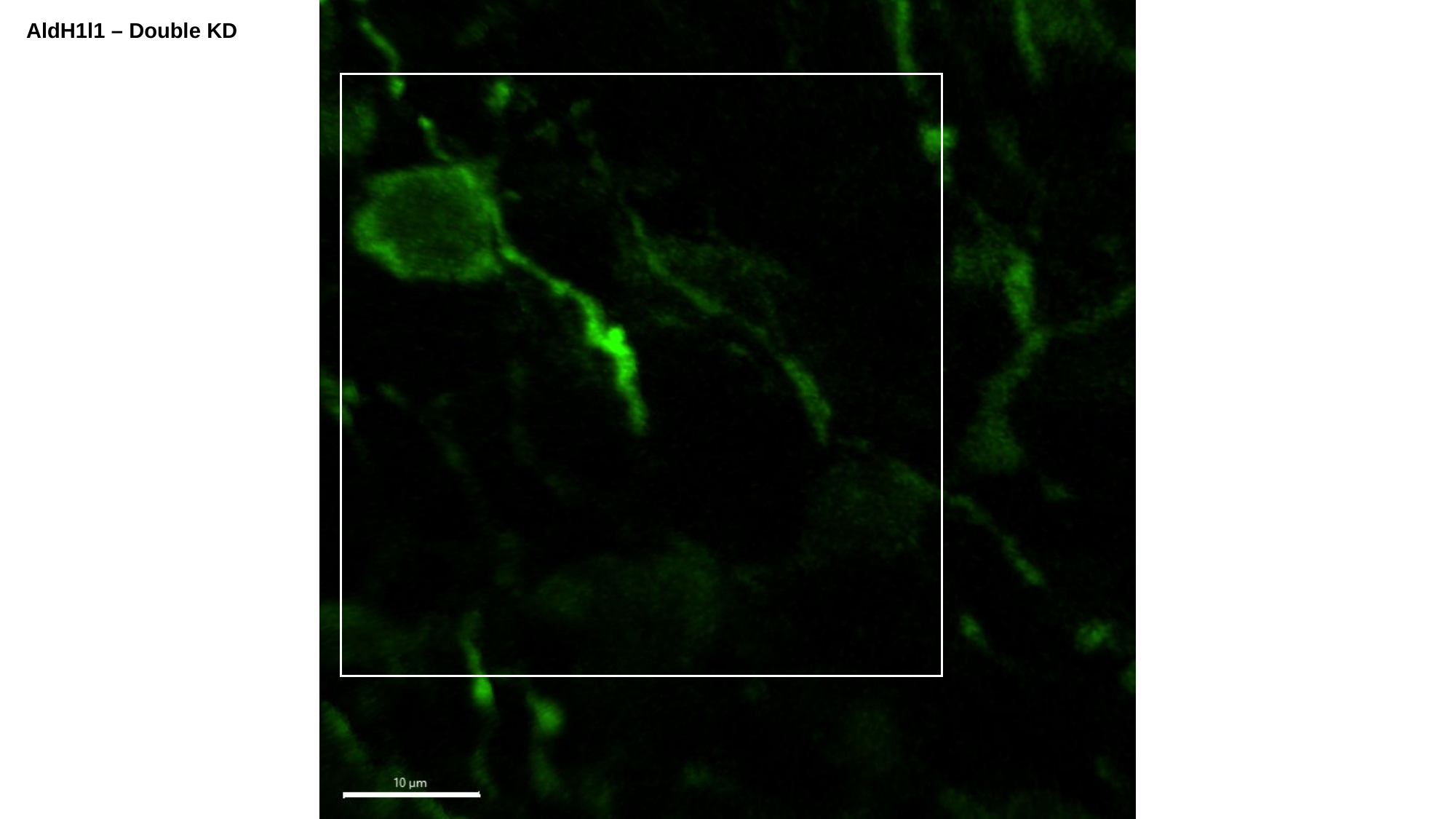

AldH1l1 – Double KD
