# Supplementary material for: Cyclin-dependent kinase inhibitor p18 regulates lineage transitions of excitatory neurons, astrocytes, and interneurons in the mouse cortex
Source: EMBO J. 2024 Dec 12;44(2):382–412. doi: 10.1038/s44318-024-00325-9 (PMC11730326; doi:10.1038/s44318-024-00325-9)
Supplement: Supplementary file 12 — Expanded View Figures [file 44318_2024_325_MOESM12_ESM.pdf]

Expanded View Figures

Figure EV1. The expression level of CDKIs, confirmation of markers, and sample preparation by laser microdissection (LMD).

(**A**) The expression level of CDKIs in RGCs in the developing mouse cortex (reanalysis of La Manno et al, 2021). The dot plot represents the expression level of CDKIs in the population of RGCs from La Manno et al scRNA-seq data. The dot size and color correspond to the proportion of cells that express the gene and the average level (log2-normalized count) of expressing cells. Arrows indicate higher expression at the onset of the gliogenesis stage (around E15-16). (**B**) Confirmation of the specificity of p18 and p27 antibodies. The control vector (a, b, g, h), p18 (c, d, i, j), and p27 (e, f, k, l) were transfected into mouse embryonic fibroblasts (MEFs). Subsequently, immunostaining using p18 (a-f) and p27 (g-l) antibodies was performed on the fixed cells to confirm that there were no cross-reactions between p18 and p27. Bar, 20 µm. (**C**) Sections of the embryonic mouse brains at E14.5 (a–c) and E15.5 (d–f) were immunostained with p18 and PECAM (a marker of a blood vessel) antibodies to confirm that bright dot structures responding to the p18 antibody were blood cells (arrowheads). Bar, 50 µm. (**D**) Confirmation of the specificity of the *Aldh1l1*-EGFP and AldH1l1 antibodies. (a–i) Sections of the *AldH1l1*-EGFP mouse brains at E17.5 (a–c), P0 (d–f), and P10 (g–i) were immunostained with AldH1l1 antibody to confirm that *AldH1l1*-EGFP expression reflects endogenous AldH1l1 protein expression. Bar, 50 µm. (j) Quantification of the immunostaining ($n = 3$ brains each). One-way ANOVA with Tukey's test; error bars show mean ± SEM. No statistically significant differences were detected. (**E**) LMD from mouse brain tissue. (a) Images before and after LMD of freshly frozen mouse brain sections at E13.5–17.5. (b) Quality check of RNA using TapeStation after preparation from mouse brain tissue by LMD (E13.5–17.5).

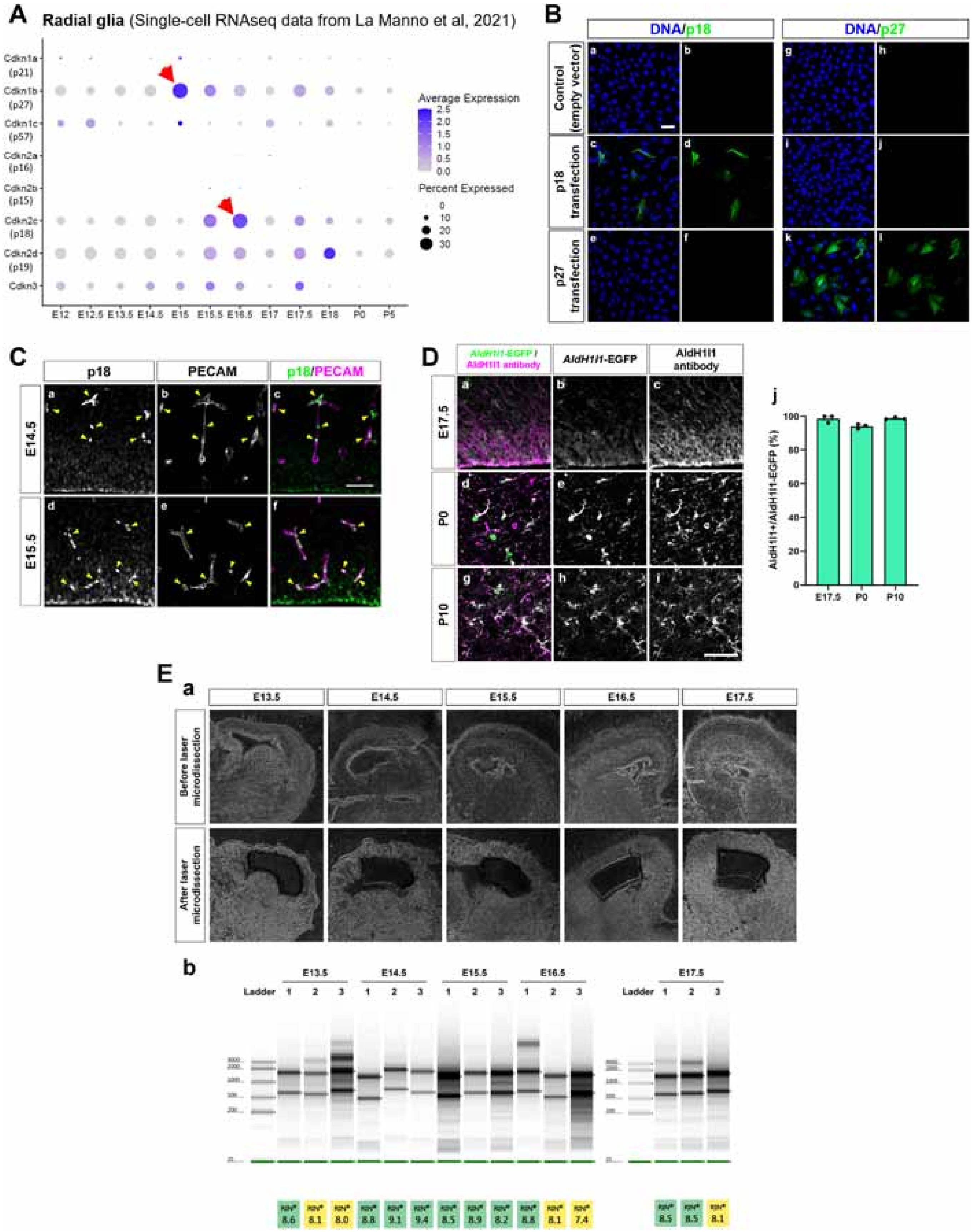
A
Radial glia (Single-cell RNAseq data from La Manno et al, 2021)
Cdkn1a (p21)
Cdkn1b (p27)
Cdkn1c (p57)
Cdkn2a (p16)
Cdkn2b (p15)
Cdkn2c (p18)
Cdkn2d (p19)
Cdkn3
E12 E12.5 E13.5 E14.5 E15 E15.5 E16.5 E17 E17.5 E18 P0 P5
Average Expression
2.5 2.0 1.5 1.0 0.5 0.0
Percent Expressed
0 10 20 30
B
DNA/p18
DNA/p27
Control (empty vector)
p18 transfection
p27 transfection
C
p18 PECAM p18/PECAM
E14.5
E15.5
D
AldH1l1-EGFP / AldH1l1 antibody
AldH1l1-EGFP
AldH1l1 antibody
E17.5
P0
P10
j
AldH1l1+/AldH1l1-EGFP (%)
E17.5 P0 P10
E
a
E13.5 E14.5 E15.5 E16.5 E17.5
Before laser microdissection
After laser microdissection
b
Ladder
1 2 3
RIN*
8.6 8.1 8.0 8.8 9.1 9.4 8.5 8.9 8.2 8.8 8.1 7.4
8.5 8.5 8.1

Figure EV2. Characterizations of p18 and p27 OE.

(**A**) Cell cycle inhibition in p18 and p27 OE brains. (a) Plasmids to induce OE of p18 and p27 mixed with pCAG-H2B-EGFP were delivered at E14.5. EdU was administered 2 h before fixation at E15.5. White squares (i-vi) are magnified images of I-III. Arrows; the border between electroporated and nonelectroporated regions. Bars, 200 μm in I-III, and 100 μm in ii, iv, and vi. (b) Quantification of EdU-positive cells among H2B-EGFP-positive cells ($n = 3$ brains each). One-way ANOVA with Tukey's test; error bars show mean ± SEM. From top to bottom, ***$P = 0.00000039$, ***$P = 0.00000120$. (**B**) Distribution of the *AldH1l1*-EGFP-positive cells with p18 OE. (a–d) Plasmids to induce OE of p18 mixed with pCAG-H2B-TagRFP were delivered at E15.5 of the *AldH1l1*-EGFP mice and then fixed at E16.5 (a), 17.5 (b), 18.5 (c), and P0 (d). Positions of the H2B-TagRFP-positive cells from the ventricular surface were measured. The bars indicate the ratio of H2B-TagRFP-positive cells in each zone among all zones ($n = 3$ brains each). Two-tailed *t* test; error bars show mean ± SEM. From top to bottom, **$P = 0.00160483$ (c), **$P = 0.00782095$, *$P = 0.01564190$ (d). (e) Representative images of the entire span of the dorsal cortex used for the quantification in (d (P0)). The cropped images close to the ventricular surface are presented in the main Fig. 2F (P0). Bar, 50 μm. (**C**) Expressions of astrocyte markers (Sox9 and GFAP) near the ventricular surface by p18 OE. Plasmids to induce OE of p18 mixed with pCAG-H2B-TagRFP were delivered at E15.5 and then fixed at P0. Sections were stained using Sox9 (a) or GFAP (b) antibody. Arrowheads; double-positive cells to Sox9 (a) or GFAP (b) and H2B-TagRFP. Bar, 10 μm. Sox9 (c) or GFAP (d) -positive cells among H2B-TagRFP-positive cells were quantified ($n = 3$ brains). Two-tailed *t*-test; error bars show mean ± SEM. **$p = 0.00288849$ (c), **$P = 0.00379764$ (d). (**D**) Effects of p18 OE on oligodendrocyte differentiation. (a) Plasmids to induce OE of p18 mixed with pCAG-H2B-TagRFP were delivered at E15.5 of the *AldH1l1*-EGFP mice and then fixed at P0. Sections were stained using PDGFR-alpha and Sox10 antibodies to recognize the oligodendrocyte lineage. Bar, 50 μm. (b) Quantification of Sox10-, PDGFR-alpha-, and *AldH1l1*-EGFP-positive cells among H2B-TagRFP-positive cells ($n = 3$–4 brains each). Two-tailed *t* test; error bars show mean ± SEM. **$P = 0.00153132$.

© The Author(s)

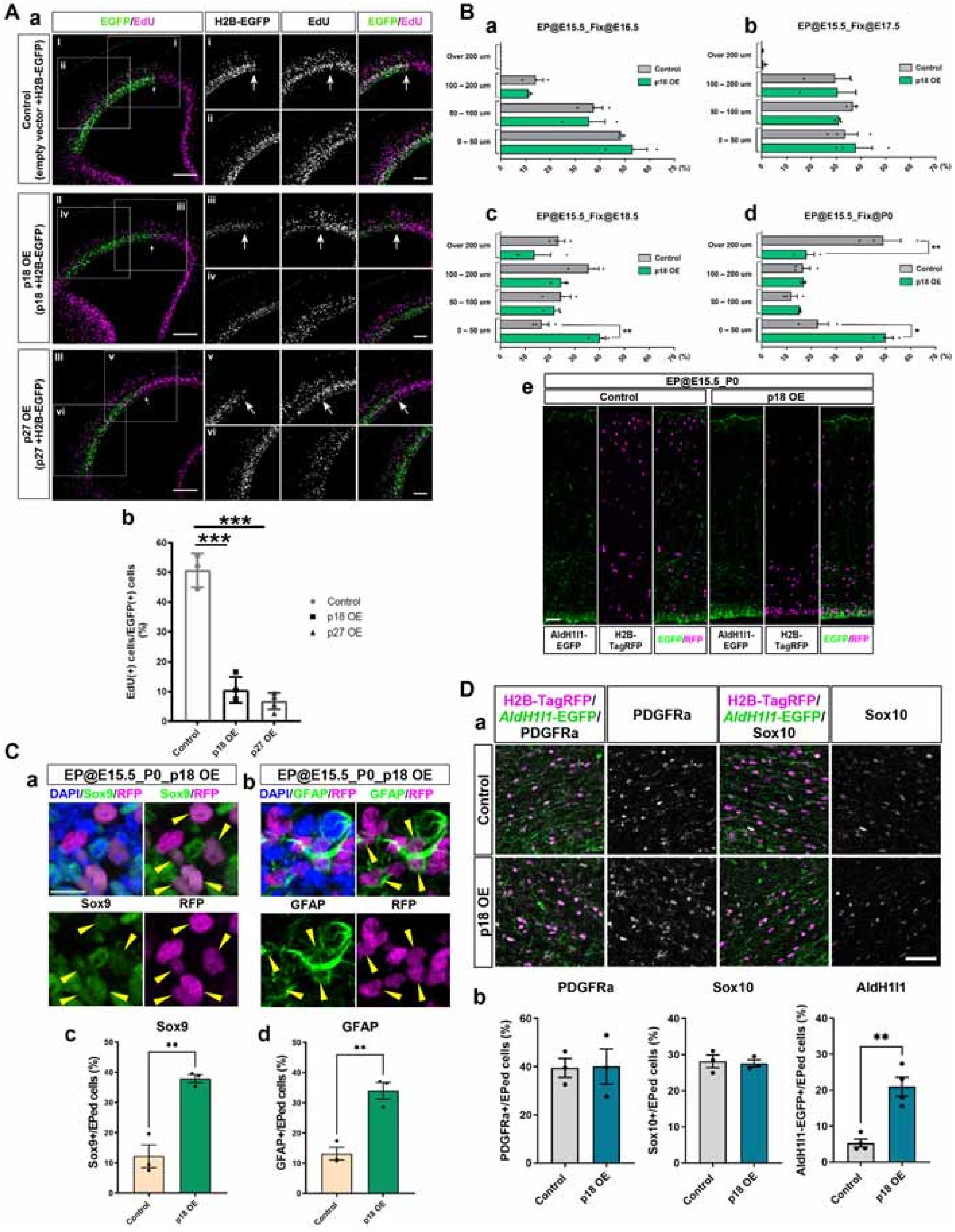
A
a
EGFP/EdU
H2B-EGFP
EdU
EGFP/EdU
Control (empty vector +H2B-EGFP)
p18 OE (p18 +H2B-EGFP)
p27 OE (p27 +H2B-EGFP)
b
EdU(+) cells/EGFP(+) cells (%)
Control
p18 OE
p27 OE
***
B
a
EP@E15.5_Fix@E16.5
b
EP@E15.5_Fix@E17.5
c
EP@E15.5_Fix@E18.5
d
EP@E15.5_Fix@P0
Over 200 um
100 ~ 200 um
50 ~ 100 um
0 ~ 50 um
Control
p18 OE
e
EP@E15.5_P0
Control
p18 OE
AldH1l1-EGFP
H2B-TagRFP
EGFP/RFP
C
a
EP@E15.5_P0_p18 OE
DAPI/Sox9/RFP
Sox9/RFP
Sox9
RFP
b
EP@E15.5_P0_p18 OE
DAPI/GFAP/RFP
GFAP/RFP
GFAP
RFP
c
Sox9
Sox9+/EPed cells (%)
d
GFAP
GFAP+/EPed cells (%)
**
Control
p18 OE
D
a
H2B-TagRFP/AldH1l1-EGFP/PDGFRa
PDGFRa
H2B-TagRFP/AldH1l1-EGFP/Sox10
Sox10
Control
p18 OE
b
PDGFRa
Sox10
AldH1l1
PDGFRa+/EPed cells (%)
Sox10+/EPed cells (%)
AldH1l1-EGFP+/EPed cells (%)

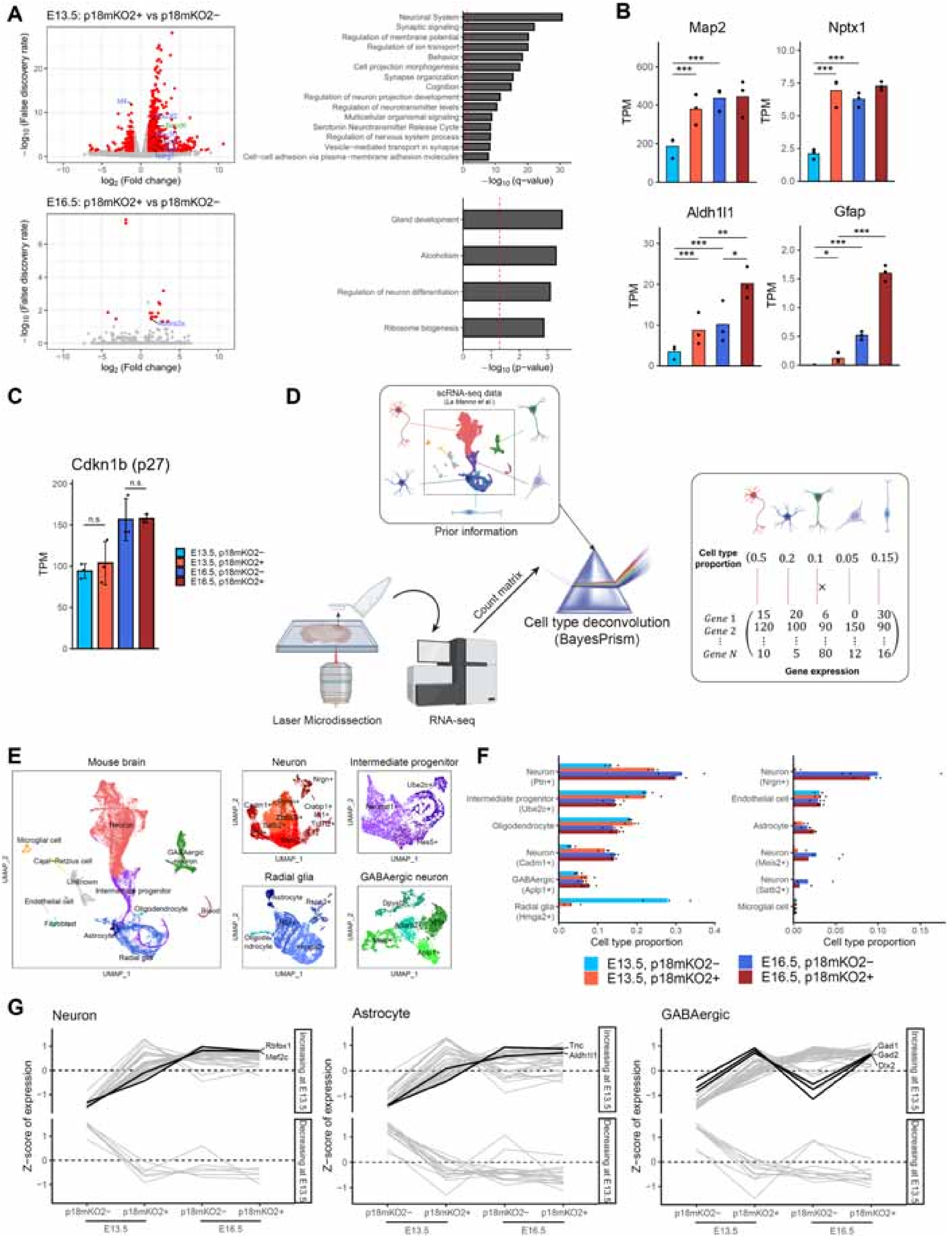

A
E13.5: p18mKO2+ vs p18mKO2−
−log10 (False discovery rate)
log2 (Fold change)
Neuronal System
Synaptic signaling
Regulation of membrane potential
Regulation of ion transport
Behavior
Cell projection morphogenesis
Synapse organization
Cognition
Regulation of neuron projection development
Regulation of neurotransmitter levels
Multicellular organismal signaling
Serotonin Neurotransmitter Release Cycle
Regulation of nervous system process
Vesicle−mediated transport in synapse
Cell−cell adhesion via plasma−membrane adhesion molecules
−log10 (q−value)
E16.5: p18mKO2+ vs p18mKO2−
Gland development
Alcoholism
Regulation of neuron differentiation
Ribosome biogenesis
−log10 (p−value)
B
Map2
Nptx1
Aldh1l1
Gfap
TPM
C
Cdkn1b (p27)
TPM
n.s.
E13.5, p18mKO2−
E13.5, p18mKO2+
E16.5, p18mKO2−
E16.5, p18mKO2+
D
scRNA-seq data
Prior information
Count matrix
Cell type deconvolution
(BayesPrism)
Cell type proportion
Gene 1
Gene 2
Gene N
Gene expression
Laser Microdissection
RNA-seq
E
Mouse brain
Neuron
Intermediate progenitor
Radial glia
GABAergic neuron
UMAP_1
UMAP_2
F
Cell type proportion
E13.5, p18mKO2−
E13.5, p18mKO2+
E16.5, p18mKO2−
E16.5, p18mKO2+
G
Neuron
Astrocyte
GABAergic
Z−score of expression
Increasing at E13.5
Decreasing at E13.5
p18mKO2−
p18mKO2+
E13.5
E16.5

© The Author(s)

Figure EV3. Cell type deconvolution by BayesPrism.

(**A**) Differential expression analysis comparing mKO2- (Control) and mKO2+ (p18 OE) at each stage. At E13.5 and E16.5, 1,211 and 273 DEGs were identified, respectively. There were changes in the expression levels of genes related to "regulation of neural differentiation" at E13.5, whereas no terms related to brain development were found at E16.5. (**B**) The expression levels of neural markers (*Map2* and *Nptx1*) and astrocyte markers (*Aldh1l1* and *Gfap*) in mKO2- and mKO2+ mice at E13.5 and E16.5. FDR was calculated by DESeq2. From top to bottom, left to right, ****P* = = 1.44e-11, ****P* = 7.15e-7 (Map2), ****P* = 3.63e-11, ****P* = 1.28e-10 (Nptx1), ***P* = 0.000234, ****P* = 1.05e-9, ****P* = 6.14e-7, **P* = 0.00120 (AldH1l1), ****P* = 1.36e-5, ****P* = 1.58e-5, **P* = 0.00936 (Gfap). (**C**) The expression levels of *p27* (*Cdkn1b*) in mKO2- and mKO2+ mice at E13.5 and E16.5 ($n = 3$ for each sample). FDR was calculated by DESeq2; error bars show mean ± SD. No statistically significant differences were detected. (**D**) Schema of the analysis pipeline for the cell type deconvolution. (**E**) UMAPs of scRNA-seq from the developing mouse brain (La Manno et al, 2021). Subtypes of Neuron (*Mapt* + , *Ly6h* +), Intermediate progenitor (*Eomes* +), Radial glia (*Nes* + , *Vim* +) and GABAergic neuron (*Gad1* + , *Gad2* +) were indicated by their marker genes. (**F**) Full deconvolution result using BayesPrism. Each dot represents a replicate of each sample. (**G**) The expression level of the top 50 variable genes found in Fig. 6F at E13.5 and E16.5. Representative genes are indicated by a thick black line.

 © The Author(s)

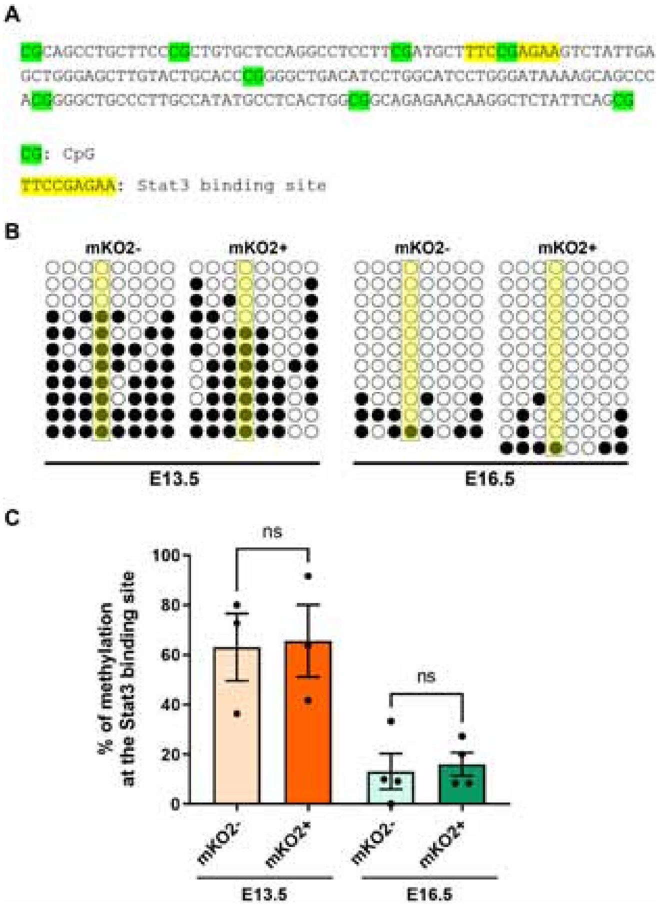

Figure EV4. Bisulfite sequencing analysis to investigate the DNA methylation status.

(**A**) Positions of CpG (green) and the Stat3 binding site at the promoter region of GFAP (yellow) investigated in a previous study (Takizawa et al, 2001). (**B**) Representative results showing methylated sites (black) among CpG positions (circle) in the cortical VZ of p18-P2A-mKO2-negative or -positive mice. Tamoxifen was administered to pregnant R26R-p18-P2A-mKO2/Nes-CreER^{T2} mice at E12.5 and 15.5, and then embryonic brains were dissected at E13.5 and 16.5. After the preparation of fresh brain sections, the genomic DNA from the VZ isolated by LMD was prepared for bisulfite assay. The yellow color indicates the Stat3 binding site. (**C**) Quantification of the methylation status at the Stat3 binding site at E13.5 and E16.5 in mKO2- and mKO2+ mice ($n = 3$ for E13.5 and 4 for E16.5 brains). Two-tailed t-test; error bars show mean ± SEM. No statistically significant differences were detected.
